# Identification of Potential Candidates with Antimicrobial Activity Against Antibiotic-Resistant *Staphylococcus aureus* Strains: A Hierarchical Bioinformatics Approach

**DOI:** 10.3390/ijms27062736

**Published:** 2026-03-17

**Authors:** Aderaldo Viegas da Silva, Kelton Luís Belém dos Santos, Lana Patrícia de Oliveira Barros Pinto de Oliveira, Luciana Sampaio Lima, Francy Mendes Nogueira Cardoso, Marcella Caroline Sampaio Vieira Carvalho, Ryan da Silva Ramos, Jorddy N. Cruz, Njogu Mark Kimani, Joaquín María Campos, Cleydson Breno Rodrigues dos Santos

**Affiliations:** 1 Graduate Program in Biotechnology and Biodiversity—Network BIONORTE, Federal University of Amapá, Macapá 68903-419, Brazil; aderaldosilva14@gmail.com (A.V.d.S.); kelton.belem@unifap.br (K.L.B.d.S.); francymnc35@gmail.com (F.M.N.C.); 2 Laboratory of Modeling and Computational Chemistry, Department of Biological and Health Sciences, Federal University of Amapá, Macapá 68902-280, Brazil; lanaoliveira2013@gmail.com (L.P.d.O.B.P.d.O.); lucianasampaio@unifap.br (L.S.L.); marcellacarvalhounifap@gmail.com (M.C.S.V.C.); ryanquimico@hotmail.com (R.d.S.R.); jorddynevescruz@gmail.com (J.N.C.); 3 Department of Physical Sciences, University of Embu, Embu P.O. Box 6-60100, Kenya; njogu.mark@embuni.ac.ke; 4 Department of Pharmaceutical and Organic Chemistry, Faculty of Pharmacy, Institute of Biosanitary Research ibs. GRANADA, University of Granada, 18071 Granada, Spain; jmcampos@ugr.es

**Keywords:** molecular docking, virtual screening, antimicrobial resistance, *Staphylococcus aureus*, computational drug discovery

## Abstract

Antibiotic resistance among several bacteria is a warning sign that reinforces the need for research to identify new compounds that are effective against resistant strains. In this sense, bioinformatics stands out as an excellent tool for identifying drug candidates by using computational methodologies to detect compounds with potential biological activity. Two pivot compounds (QNZ and 0Y5) with biological activity against *Staphylococcus aureus* were selected. A virtual screening was performed in the MolPort database with a Tanimoto index of 0.5, resulting in 20,000 compounds, 10,000 compounds for each template. Then, methodologies were applied to calculate pharmacokinetic and toxicological parameters using Discovery Studio software; molecular docking via DockThor; lethal dose via ProTOX; lipophilicity, solubility, and Lipinski parameters via SwissADME; in silico prediction of bacterial activity via Way2Drug; theoretical synthetic accessibility via SwissADME and AMBIT-SA; and, finally, molecular dynamics simulations via AMBER 18. After the entire methodological process, 10 compounds were identified with potential results according to the criteria adopted in this study and with possible antimicrobial activity against resistant bacterial strains of *S. aureus*. Our theoretical findings suggest 10 potential candidates with possible antimicrobial activity against *S. aureus* and other genera and species of bacteria as these compounds presented excellent results using the proposed methodology. Certainly, more in vitro and in vivo study steps are necessary.

## 1. Introduction

Bacterial infections affect humanity in several ways, causing diseases directly in humans [1,2] or affecting functional areas of society, such as agriculture and health systems [3]. Human diseases originating from virulent bacterial strains can trigger anything from mild infections (pimples and skin boils) to severe systemic infections, such as pneumonia, meningitis, endocarditis, toxic shock syndrome and septicemia [4].

Antibiotic resistance is a growing global problem with major impacts on public health. Restrictions on treatment protocols and threats to the longevity of available drugs are obvious issues already debated in health systems around the world [5,6,7]. According to the World Economic Forum Global Risks Report, bacterial resistance is identified as a major threat to human health in the coming years [6]. Furthermore, the World Health Organization highlights that, in recent years, the antibiotics developed have not sufficiently addressed the problem of bacterial resistance [8].

Among the antibiotic-resistant bacteria posing risks to public health is *S. aureus*, a Gram-positive coccus arranged in grape-like clusters. It preferentially colonizes the external mucosa but can become an opportunistic pathogen in humans, triggering various systemic infections and septicemia. Its virulence is attributed to surface proteins, enzymes and toxins that cause damage to host tissues. Antimicrobial resistance makes *S. aureus* a prominent agent of hospital infections, mainly due to the lethality of patients colonized by staphylococci [9]. The estimated lethality for an infection by *S. aureus* is estimated at 10% to 30%, with age being the most consistent factor for this lethality [10]. For years, beta-lactam antibiotics, a class to which penicillin and its derivatives belong, were used as a treatment for *S. aureus*. These bacterial strains with antibiotic resistance are given the generic name of methicillin-resistant *S. aureus* (MRSA), meaning not only resistance to methicillin but broad resistance to several antibiotics [11,12].

Designing and developing new drugs is an activity that demands time, resources, and numerous tests. Biological tests aim to confirm that a given pharmacological substance is effective and functional against a given organism or disease and that this efficiency should not result in major adverse effects on the host. These steps demand time, financial and human resources [13]. Thus, rational drug design is a supporting tool for biotechnology, where technology and the power of computational tools make it possible to replace time-consuming preclinical biological tests, in addition to enabling substances to be tested in a virtual environment without posing biological or chemical risks to researchers [14,15].

Recent advances in X-ray crystallography and nuclear magnetic resonance have been crucial for understanding protein structures in biological systems, enabling computational resources to simulate and calculate the processes involved in molecular interactions and interactions with biological receptors, thus bringing the results of computational approaches closer to what actually happens in reality at the atomic and molecular level [16,17].

Therefore, the aim of this study was to identify potential candidates with inhibitory activity for the enzymes PBP2a (PDB ID: 4CJN) and thymidylate kinase (PDB ID: 4GSY) from two pivot compounds with biological activity, QNZ and 0Y5, through a bioinformatics approach. The QNZ compound has, as its proposed mechanism of action, the ability to bind to the allosteric site of the PBP2a enzyme. Its intermolecular interactions in this region promote a conformational change of the active site (site of the catalytic reaction of the peptidoglycans of the bacterial cell wall) of the enzyme. The allosteric site functions as a regulatory site to the active site, so compounds with affinity for this region will trigger enzymatic inhibition and death of the bacterial cell, worsening the interruption of the cell wall reaction cascade [18].

For compound 0Y5, the authors provide, as a mechanism of action, the competitive binding of this compound to the catalytic site of phosphorylation of deoxythymidine monophosphate (dTMP) into deoxythymidine diphosphate (dTDP) using adenosine triphosphate (ATP) as a phosphate donor substrate. This conversion of dTMP into dTDP is essential for the synthesis of deoxythymidine triphosphate (dTTP), an essential compound for the polymerization of deoxyribonucleic acid (DNA). By preventing the synthesis of dTTP, the ability of the bacterial cell to replicate its DNA is interrupted, preventing cell divisions and promoting bacterial inhibition [19].

## 2. Results and Discussion

### 2.1. Selection of Compounds Based on the Tanimoto Index

The pivot compounds were used as templates to search for similar compounds in the MolPort database. The index adopted for this study was 0.5 (50%) of similarity to the pivot compounds. Table 1 illustrates the number of hits obtained in the Tanimoto search with an index ranging from 0.5 to 1.

### 2.2. Pharmacokinetic and Toxicological Prediction of Compounds Selected via Discovery Studio

#### 2.2.1. Pharmacokinetic Prediction

**Figure 1:**
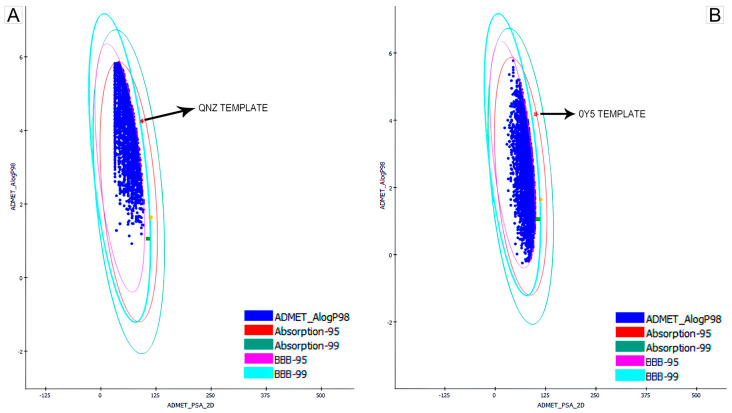
In (**A**)-top 4496 compounds screened from QNZ inside ellipses; in (**B**)-top 3256 compounds screened from 0Y5 inside ellipses. The yellow square represents Oxacillin; the green square represents Methicillin.

The first pharmacokinetic filter used to screen promising compounds was the ellipse plot provided by Discovery Studio when calculating ADMET properties. As specified by the software manual, the compounds (represented by the blue dots) that are within the four highlighted ellipses are the chemical molecules with the greatest potential to be used for the oral administration route. Figure 1 shows the initial screening of the bases obtained from QNZ and 0Y5. The commercial compounds oxacillin and methicillin were also used as pharmacological references for tabulating the results [20]. In the graph, they are represented by the yellow and green symbols, respectively.

The compounds grouped within the four ellipses demonstrate greater potential for oral administration, which is the intended form of administration for this study and in future stages of in vitro/in vivo development. All the compounds that were left out of the ellipses were eliminated from the database and did not proceed to the next stages of this study. Figure 1 illustrates the locations of the compounds that proceeded to the next stages.

This step resulted in the elimination of a total of 5504 screened compounds from QNZ and 6744 compounds from 0Y5.

After subtracting the compounds eliminated by ellipses, the remaining products were screened in the other filters for solubility, blood–brain barrier penetration, CYP2D6, hepatotoxicity, intestinal absorption and binding to blood plasma proteins. When calculating these properties, Discovery Studio returns a value and specifies with a caption what this value means. The comparison parameters for the results are the values of the pivot compounds, from which those molecules that have a similar or better value were selected. Values outside this inclusion criterion resulted in the elimination of that compound from the database.

Due to the high number of molecules remaining after subtracting the ellipses (4496 for QNZ and 3256 for 0Y5), and to prioritize formatting, Table 2 shows only the values obtained for the pivot compounds and the two selected commercial compounds (methicillin and oxacillin) that were the parameters used as inclusion criteria in the selection of candidates with the potential to become new drugs to combat resistant *S. aureus* strains; see Supplementary Materials of the results obtained of molecules selected in the pharmacokinetic step from the compounds QNZ and 0Y5.

As can be seen, for solubility, QNZ and 0Y5 presented a value of 2, which represents low solubility. Therefore, the selected compounds were those that obtained a value of 2 and a value of 3. When compared to commercial compounds, it is clear that methicillin and oxacillin have solubility 3. Therefore, the compounds selected for the solubility criterion were those with the same value as the pivot compounds and the same value as already approved drugs. The reason for this choice is precisely the biological activity that all compounds have against the bacterium *S. aureus*.

For the blood–brain barrier (BBB) penetration attribute, both pivot compounds presented a value of 4, which represents an undefined result in which the result of the calculation used by the software configures a value that exceeds the statistical confidence intervals for passage or not through the blood–brain barrier by the compound investigated. The selected compounds were those that obtained values 0 (very high penetrance); 1 (high penetrance) and 2 (medium penetrance).

The BBB is a structure that protects the Central Nervous System (CNS) and regulates the exchange of substances present in the blood with the brain and spinal cord. It is a barrier with extremely restricted permeability, in addition to containing enzymes with the capacity to degrade several substances that often do not penetrate the brain in their entirety [21]. Crossing this membrane is a pharmacological challenge; many drugs are unable to penetrate this protective layer, which makes it difficult to treat bacterial brain infections. Experimental studies are already investigating mechanisms to alter BBB permeability using ultrasound, thus enabling increased efficiency in the treatment of bacterial infections that affect the CNS by enabling better passage of drugs to the brain [22]. These experiments will enable the development of new antibiotics with high efficiency in reaching the Central Nervous System, without undergoing metabolism in the process.

When dealing with *S. aureus*, there are clinical cases of meningitis caused by these bacteria [23], so the choice of compounds with the potential to cross the BBB is based on the premise of enabling future new drug candidates with the potential to treat these serious infections in medicine.

For CYP2D6 inhibition, both template and commercial compounds presented false results, i.e., there is no inhibition of this enzyme family. All the compounds that presented the true result were eliminated from the database. Investigations on CYP2D6 for pharmacology are important because there is a direct impact on the analysis of drug metabolism when investigating these polymorphic enzymes, with 20% of drugs worldwide used for oncological treatments, viral/bacterial infections and cardiopathologies being metabolized by the CYP2D6 enzyme family. Therefore, screening compounds that will not cause enzyme inhibition is fundamental and means ensuring that there will be no bioaccumulation in tissues, thus causing organ toxicity and changes in the metabolic capacities of the drugs under investigation [24].

Hepatotoxicity was positive only for compound 0Y5 among the chosen model compounds, indicating the need for more refined studies before this compound can become an approved drug. QNZ has a negative value for hepatotoxicity; therefore, molecules with positive values were eliminated from the sample group screened using QNZ.

Hepatotoxicity caused by drugs can trigger drug-induced cirrhosis and compromise liver function. Drug-induced liver injury (DILI) is an adverse reaction with the potential to cause liver failure and even death. DILI should be considered in the research processes for new drugs, whether in computational, clinical studies, or even in drugs already approved and marketed [25]. Intestinal absorption was shown to have a value of 0, which corresponds to good absorption. All the compounds screened from QNZ and 0Y5 have a value of 0 for their intestinal absorption.

Finally, binding to blood plasma proteins reflects the ability to circulate and trigger the desired effect since this binding of blood plasma proteins can influence a slower release and availability of the compound in the circulation since this binding allows the compound to momentarily “escape” from metabolism and can provide protection against oxidative stress of the host during its transport so that the compound will be protected by being connected to the plasma protein. On the other hand, this measure reinforces the need for attention regarding doses that may cause a toxic effect to the drug since these bonds with blood plasma proteins are not definitive and can be reversed, thus allowing the concentration of the drug bound to the proteins to reach toxic doses when released. Future stages of in vitro testing will be essential to elucidate this mechanism and how the promising compounds screened will behave, especially when it comes to the proposed development of antibiotics [26].

QNZ has a positive value for binding to blood plasma proteins, while 0Y5 showed a negative value. In comparison to commercial compounds, only oxacillin shows positivity in binding to plasma proteins. All the compounds screened from QNZ and 0Y5 were selected with a positive value for binding to blood plasma proteins.

After applying all the pharmacokinetic filters and eliminating compounds that did not meet the chosen criteria, the result of promising compounds that met the requirements was 610 compounds for QNZ and 165 compounds for 0Y5.

#### 2.2.2. Toxicological Prediction

Discovery Studio’s toxicological prediction is based on algorithms developed and validated to investigate the Quantitative Structure–Toxicity Relationship (QSTR) and to analyze specific toxicological endpoints based only on the analysis of the 2D structure of the compounds entered for screening.

Table 3 presents the values obtained for the template and commercial compounds, which were used for screening the compounds. Once again, to prioritize formatting, since a total of 775 compounds were analyzed for toxicology, Table 3 presents the inclusion criteria that guided the selection of compounds with the best toxicological values. It is worth reiterating that screening always starts with the selection of properties similar to those of the template or commercial compounds, or with superior properties; see the Supplementary Materials of the results obtained for molecules selected in the toxicological step from the compounds QNZ and 0Y5.

Substances under investigation to become officially approved and commercially available drugs may have a carcinogenic effect directly linked to their therapeutic function or as an undesirable consequence of their action [27]. It is essential that investigations into the potential carcinogenicity of future drug candidates are carried out, mainly to ensure that the therapeutic effect of treating a bacterial infection does not trigger the development of a neoplasm.

A systematic review study with a meta-analysis guided by Petrelli (2019) [28] revealed a total of 17 neoplastic conditions incident on eight classes of antibiotics, namely: beta-lactams, cephalosporins, macrolides, tetracyclines, fluoroquinolones, nitrofurantoins, sulfonamides and nitroimidazoles. In an analysis involving 7,947,270 individual patients from 25 observational studies, antibiotic use was associated with an 18% increase in cancer, mainly in lung, hematologic, pancreatic and genital tract cancers. One of the possible hypotheses that explain this ability to trigger neoplasia is precisely that antibiotics negatively affect the symbiotic bacterial microbiota of the human gastrointestinal tract. Since these bacteria have associations with the immune system and prevention against infections by external pathogenic bacteria, prolonged use and high doses of antibiotics may therefore be carcinogenic.

Therefore, it is important to highlight that future in vitro stages of this research will follow the most rigid and specific protocols to investigate the potential neoplastic effect of the promising compounds found in cell colonies. In our in silico results, all the compounds that presented non-carcinogenic, non-mutagenic and non-toxic values were selected.

After completing the pharmacokinetic and toxicological screening, from the initial database of 10,000 molecules for each selected template compound, 77 compounds remained screened from QNZ and 46 compounds screened from 0Y5. These final selected compounds proceeded to molecular docking, where the top five compounds for each target were selected based on affinity energy.

### 2.3. Molecular Docking for Biological Targets

**Figure 2:**
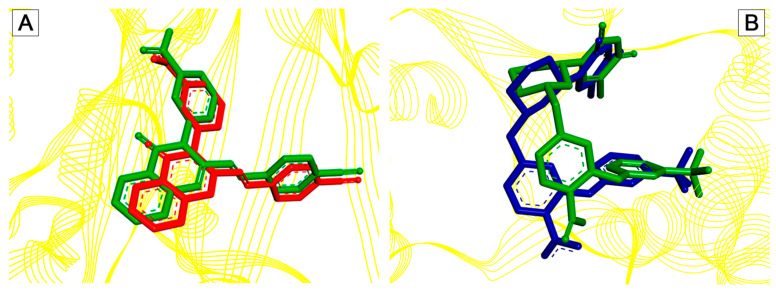
In (**A**) QNZ with RMSD of 0.795Å (green is the crystallographic conformation and red is the redocking conformation) and ΔG = −8080 Kcal/mol; in (**B**) 0Y5 with RMSD of 1.9313 Å (green is the crystallographic conformation and blue is the redocking conformation) and ΔG = −9147 Kcal/mol.

To validate the molecular docking simulation, the redocking methodology was chosen, which consists of removing the crystallographic ligand from the protein structure and “replacing” it through docking. This method serves to demonstrate the ability of the software algorithm used to replicate the same crystallographic conformation of the ligand deposited in the PDB. If the root mean square deviation (RMSD) is less than 2 Å between the crystallographic conformation and the ligand docked via docking, then the protocol is properly validated, and the docking of the selected ligands can be performed [29]. Figure 2 indicates the RMSD obtained in the redocking of the pivot compounds QNZ and 0Y5, respectively.

#### 2.3.1. Molecular Docking in the PBP2a Enzyme

The QNZ activity site consists of the allosteric site of the PBP2a protein. Allosteric sites have the property of influencing the conformational change and regular functioning of the enzyme’s active site [30]. Thus, the same effect is expected from the compounds screened from QNZ, causing the inhibition of the PBP2a enzyme and the consequent death of the bacteria as there was no synthesis of the cell wall, an essential structure for the survival of *S. aureus*. This entire process was possible thanks to the biological activity of the compounds in the cavity of the allosteric site of the PBP2a enzyme, where inhibition of this site is essential to address the problem of antibiotic resistance [31]. Table 4 illustrates the top five compounds with the best affinity energy, as well as the amino acid residues that presented interaction and the affinity energy in Kcal/mol.

The PBP2a enzyme is composed of chains A and B. The allosteric site is located in chain B. Compound CAV01 presented the lowest affinity energy and a total of five interactions with residues, with one bond corresponding to a residue of the crystallographic interaction of QNZ. The interactions recorded were hydrophobic, being of the carbon hydrogen type (B.ILE144 and B.ASP295); Pi-Cation (B.LYS273); Pi–Pi T-shaped (B.TYR297) and Pi-Alkyl (A.ILE309), this compound being the only one to present interaction with a residue of chain A.

The compounds that presented hydrogen bonding, all with the residue B.ILE144, were the compounds CAV02, CAV03, CAV04 and CAV05. The interactions recorded with the residue B.TYR297 were of the Pi–Pi T-shaped type for all the compounds, with the exception of CAV02 and CAV03, which presented amide-Pi Stacked bonds with this residue.

Intermolecular interactions between drugs and their sites of action are essential to trigger the desired biological effect. These interactions can be hydrophobic, conventional hydrogen bonds and, in rare cases, covalent bonds, making it possible to trigger effects that can cause conformational changes in proteins, inhibition of catalytic cavities and permanent inactivation of enzyme function [32,33,34]. Therefore, since the interactions with the amino acid residues were like those of the template compound QNZ, as well as the affinity energy of the top five compounds was better, it is expected that the hypothesis of causing a biological effect similar to QNZ was observed in the top five compounds obtained via molecular docking. This finding becomes promising given the fact that the top five compounds for screening from QNZ have better pharmacokinetic and toxicological parameters than the template compound, in addition to a better affinity energy.

#### 2.3.2. Molecular Docking in the TMK Enzyme of *S. aureus*

The catalytic site of 0Y5 corresponds to the site of reaction and transformation of deoxythymidine monophosphate (dMTP), so the compound acts with an antagonistic effect, binding to the site and preventing the cascade of the compound essential for DNA synthesis from occurring [35,36]. Therefore, the same inhibitory effect is estimated with the compounds screened in this study given the binding to the catalytic site by the screened compounds. Table 5 illustrates the top five compounds with the best affinity energy, the interactions of the compounds with the amino acid residues and the affinity energy in Kcal/mol for the compounds screened from 0Y5.

For the compounds coupled to the bacterial TMK, it is evident that the template molecule presented the second best affinity energy with the enzyme, so this finding is in agreement with the studies of Martínez-Botella [19], demonstrating the high affinity that compound 0Y5 has with the TMK of *S. aureus*. In terms of the overall ranking of the compounds screened from 0Y5, this study found only one compound with affinity energy lower than the template molecule for the bacterial TMK target.

The compound that presented the lowest affinity energy was GAV01, with an estimated energy of −9.362 Kcal/mol, with seven interaction hits, of which four correspond to the same interactions as the crystallographic 0Y5 originally deposited in the PDB.

The compounds that presented the highest number of interaction hits, with the exception of the template molecule that has 11 hits, are the compounds: GAV04 with nine hits, of which six correspond to crystallographic interactions, all of which are hydrophobic; GAV02 and GAV03 both with eight hits, of which five correspond to crystallographic interactions. In addition, both interact through hydrogen bonding with the residue A.ARG105, being the only two compounds to present conventional hydrogen bonding. The top five compounds presented good binding affinity values, in addition to interactions with amino acid residues close to the template compound 0Y5.

**Figure 3:**
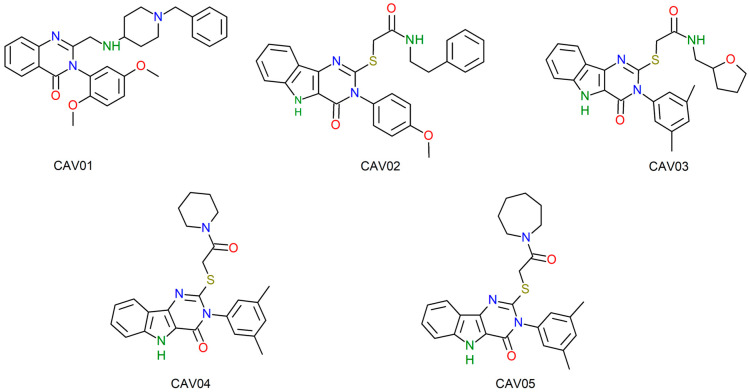
Compounds CAV01, CAV02, CAV03, CAV04 and CAV05 sorted from pivot compound QNZ.

**Figure 4:**
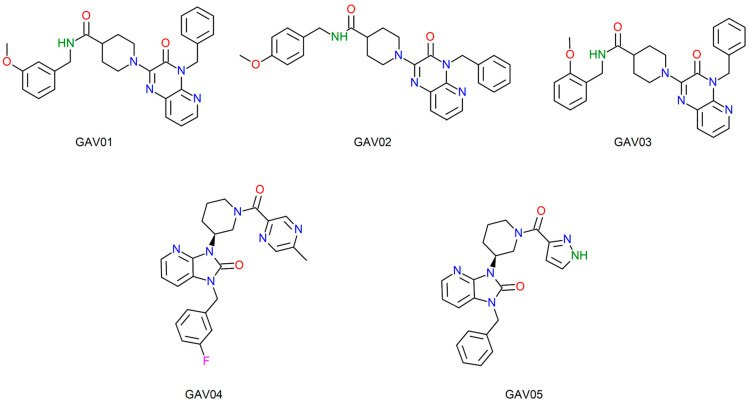
Compounds GAV01, GAV02, GAV03, GAV04 and GAV05 sorted from pivot compound 0Y5.

**Figure 5:**
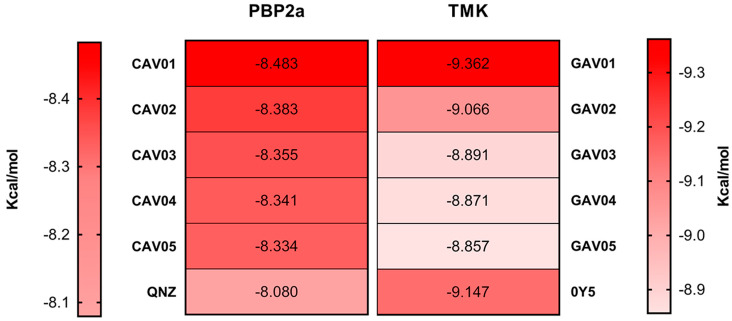
Heatmap of docking affinity energies for the top-ranked compounds against PBP2a and thymidylate kinase (TMK).

Figure 3 and Figure 4 illustrate the two-dimensional structures of the top five compounds for each biological target adopted. Finally, Figure 5 presents the heatmap of the compounds and their respective molecular targets, highlighting the affinity energy obtained. The more negative, the better. The heatmap provides an overview to observe the variations in affinity energy between compounds and their respective targets. Although the screening strategy was based on similarity to reference ligands, resulting in structurally related compounds, the selected candidates are not strictly redundant. The compounds selected via MolPort exhibit variations in functional group composition and physicochemical properties. A comprehensive analysis of structural diversity or structure–activity relationships was beyond the scope of this study and is planned as part of future lead compound optimization efforts.

### 2.4. Lethal Dose and Toxicity Class of the Top 5 Compounds Obtained by Molecular Docking in Both Screening Fronts

Toxic doses (LD_50_) are often expressed by the value known as LD_50_, which represents the median lethal dose and is indicated in milligrams of substance per kilogram of body weight (mg/kg). The LD_50_ corresponds to the amount of substance required to cause the death of 50% of a population of organisms exposed to the toxic agent. This value is used in toxicology studies to measure the degree of danger of a substance and is essential for risk assessment and determination of safe exposure limits for drug candidate substances [36]. Table 6 shows the LD_50_ values obtained and the toxicity class that the screened compounds presented.

The pivot compounds QNZ and 0Y5 have toxicity Class 4, with LD_50_ values of 450 and 1000 mg/kg, respectively. When compared to the approved antibiotics methicillin and oxacillin, there is evidence of a decrease in the level of toxicity since both are classified as Classes 5 and 6. According to [37], Class 4 is described as “harmful if ingested”, reinforced by the lethal dose that will serve as support for conducting in vitro tests to establish safe dosages, minimizing toxicity to the host. As specified by Grandjean [38], modern toxicology investigates and is concerned with the hazards and chemical exposures that drugs can cause. Every substance, to some degree, presents toxicity; therefore, the safety parameters for effective biological effects are linked to the dosage that will enable treatment or a toxic effect, respecting the concept that the difference between poison and an antidote is the dosage.

In the findings of this study, all the compounds screened for the two molecular targets presented toxicity Class 4 and LD_50_ values ranging from 677 mg/kg (CAV02) to a maximum of 1200 mg/kg (GAV01, GAV02 and GAV03), which is a satisfactory result and with parameters close to the pivot compounds. Future studies are necessary to identify analogues of these compounds with a higher toxicity class (Class 5 or Class 6), increasingly seeking safe dosages in the process of developing new compounds with potential antibiotic activity and bringing the new chemical entities closer to the standards of drugs that have already been approved and are marketed.

### 2.5. Lipophilicity, Water Solubility and Lipinski’s Parameters Lipophilicity Values, Water Solubility Values, and Lipinskiy Parameters

Lipophilicity studies use the partition coefficient between n-octanol and water (o/w). This parameter establishes how lipophilic a molecule is. Lipophilicity is directly related to the tendency of a compound to decompose in nonpolar and polar environments. An increase in lipophilicity can trigger an increase in permeability, protein binding and molecular distribution volume. When treating bacterial infections, compounds need to cross cell membranes (with lipid composition) to reach their targets and trigger their effects [39,40,41].

The SwissADME webserver provides five prediction models for LogP values and, in the end, establishes a Consensus LogP value, which means the average obtained between the values of the five models: iLOGP, XLOGP3, WLOGP, MLOGP, and SILICOS-IT [38,41]. Thus, the analysis becomes more concise when interpreting the results of the LogP consensus.

The pivot compounds presented values of 3.61 and 3.35 for QNZ and 0Y5. Among the compounds screened from QNZ, the lowest value was 3.74 for compound CAV03 and the highest was CAV05 with a value of 4.49.

In the screening front from 0Y5, the lowest value was 2.01 for GAV05 and 3.25 was the highest value with compound GAV01.

**Figure 6:**
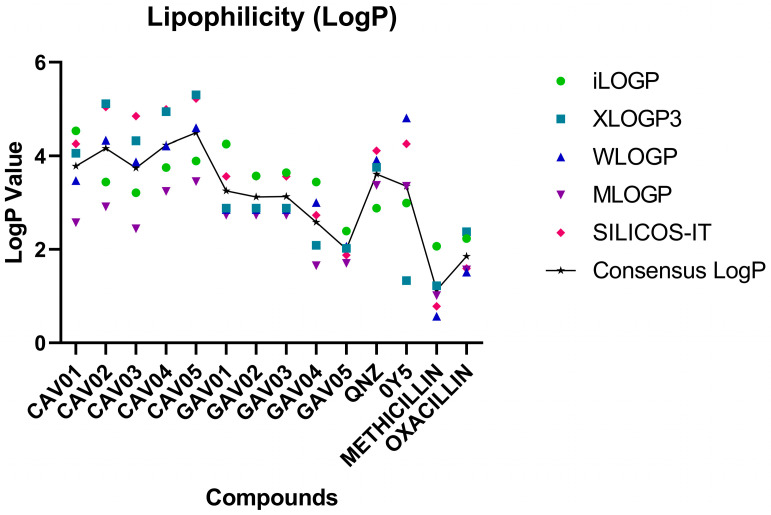
Lipophilicity (LogP) profiles of the top five screened compounds and reference antibiotics estimated using multiple computational models (iLOGP, XLOGP3, WLOGP, MLOGP, and SILICOS-IT) and their consensus values.

The maximum value for good lipophilicity is considered to be 5.00 [42,43]. Therefore, all the screened compounds comply with this range and can be considered fat-soluble compounds with acceptable values for use as drugs [44,45]. For lipophilicity, see Figure 6.

Aqueous solubility appears to be an important requirement for drug candidates to be administered orally as this descriptor demonstrates the ability of the active substance to be administered in a low volume [46]. Thus, compounds are classified according to their solubility when the following parameters are observed: between −2 and −4 good solubility; between −4 and −6 indicate moderate solubility; and values greater than −6 indicate poor solubility [47].

**Figure 7:**
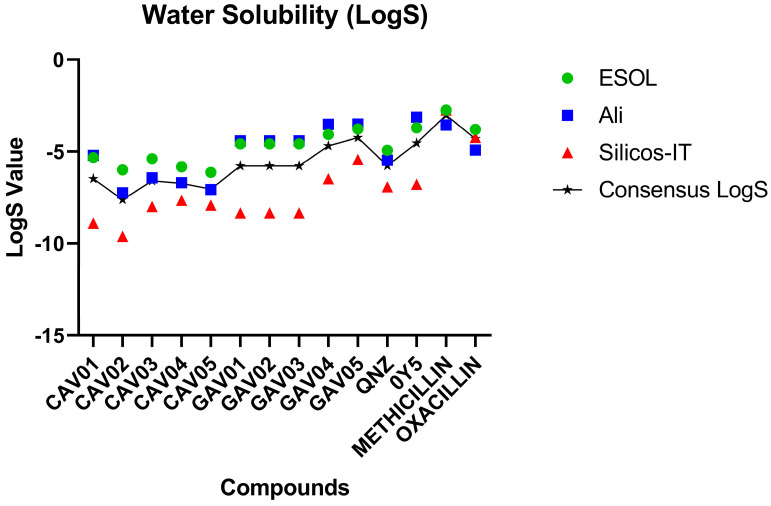
Water solubility (LogS) profiles of the top five screened compounds and reference antibiotics calculated using ESOL, Ali, Silicos-IT models, and their consensus values.

See Figure 7 for the water solubility parameters.

Both pivot compounds were classified as having good solubility. In this sense, there were no screened compounds with good solubility values, with the exception of the commercial compound methicillin. Compounds with moderate solubility were found in all top five compounds screened from 0Y5 (GAV01, GAV02, GAV03, GAV04 and GAV05). All the compounds screened from QNZ (CAV01, CAV02, CAV03, CAV04 and CAV05) were classified as having poor solubility as they presented values above −6.

Although the predicted aqueous solubility of the selected compounds was classified as poor, this parameter was not considered exclusionary at the hit identification stage as solubility optimization is routinely addressed during subsequent lead optimization and formulation steps. After the synthesis of the molecules, it is considered that this methodological proposal may contribute to the development of suitable solutions by medicinal chemists based on their solubility properties considering future in vivo assays, with the purpose of validating the computational methods employed.

Lipinski’s rule (also called the rule of five “RO5”) is an important set of physicochemical parameters to be evaluated for compounds with the potential possibility of being administered orally. The rule of five establishes that a good compound to be administered orally and have good bioavailability must have: molar mass (MM) ≤ 500, LogP ≤ 5; hydrogen donor group (GDH) ≤ 5 and hydrogen acceptor group (GAH) ≤ 10. The rule of five receives this name not because there are five parameters but because the values of the parameters are multiples of five [43].

Table 7 demonstrates the RO5 parameters calculated via SwissADME for the top five compounds of each target.

With the exception of the template compound 0Y5, which violated the parameter of having a molar mass (MM) greater than 500 g/mol, all the screened compounds are within the standardized ranges of the Lipinski rule. Therefore, they are classified as promising compounds for oral administration.

### 2.6. In Silico Antibacterial Activity Prediction of Top 5 Compounds from Both Molecular Targets

The AntiBac-Pred tool allows predictions to be made about whether a chemical compound has the ability to inhibit the growth of one or more than 353 species of bacteria available in the AntiBac-Pred algorithm. When the desired compound is entered to make the prediction, the result is displayed indicating which type of bacteria the inhibitory activity may occur in and the confidence value. There is no minimum confidence value to consider the potential inhibitory activity; however, a higher confidence value demonstrates that the chemical compound has a greater probability of being active and causing inhibition of the indicated bacterial strains according to the calculation performed by AntiBac-Pred [48,49].

Table 8 demonstrates the results obtained for predicting the antibacterial activity of the top five compounds for the target PBP2a. To validate the algorithm, the commercial compounds methicillin and oxacillin were inserted, which are known to have biological activity against a certain diversity of *S. aureus* strains.

As observed, the antibacterial prediction for the drugs methicillin and oxacillin indicates inhibitory activity against *S. aureus* strains, emphasizing the resistant subtype *S. aureus* subsp. *aureus* RN4220, thus demonstrating the predictive specificity of AntiBac-Pred to indicate possible antibacterial activity.

Regarding the five main compounds selected from QNZ, it is observed that all five molecules showed predicted antibacterial activity against some type or subtype of staphylococcal bacteria. It is recognized that the reliability values are low when compared to commercial antibiotics; however, the AntiBac-Pred tool is being used not as a way to confirm biological activity but rather to guide which bacterial strains future experimental stages of this study should focus on, in addition to resistant S. aureus strains. In this sense, compounds CAV02, CAV03, CAV04 and CAV05 stand out, for which possible activity was recorded for the resistant bacterial subtype *S. aureus* subsp. *aureus* MW2. Other types of staphylococcal bacteria recorded were *S. haemolyticus* (CAV01); *S. intermedius* (CAV02) and *S. sciuri* (CAV03). Thus, new perspectives can be developed for this study in order to investigate the potential of these compounds against other types of staphylococcal bacteria, not only *S. aureus*. These results obtained for the top five compounds screened from QNZ are fundamental to guide the future experimental stages.

Compounds CAV02, CAV03, CAV04, and CAV05 showed potential for activity against staphylococcal bacterial strains. It is essential to perform biological tests to investigate these activities in bacterial colonies, as well as to establish dosages and minimum inhibitory concentration (MIC) values.

The compound CAV01 may be tested against other strains of bacteria of medical importance, as indicated by the possible activity of AntiBac-Pred, and it will also be tested against resistant strains of *S. aureus*.

For the compounds screened from 0Y5 onwards, it is noted that there was no explicit result for potential activity against resistant *S. aureus*, as shown for the compounds screened from QNZ. However, there is a record of potential activity, in general, against the genus *Staphylococcus*, represented by all five molecules analyzed, which allows us to investigate the possibility of antibacterial activity against antibiotic-sensitive strains.

Experimental studies with these compounds are essential to check this activity, as well as the possibility of inhibiting resistant strains, since the result “*Sthapylococcus* sp.” is a term that refers to several types of bacteria belonging to the *Staphylococcus* genus, which may include resistant strains. Table 9 illustrates the results obtained for the top five compounds for the TMK target screened from 0Y5.

Expanding the analysis, it was noted that there was potential biological activity for other types of bacteria that are not of the *Staphylococcus* genus. Compounds GAV01, GAV02 and GAV03 showed predicted activity for the bacterial types RESISTANT *Propionibacterium acnes* and RESISTANT *Bacteroides thetaiotaomicron*. Compound GAV04 was the only one to record potential activity against the types of mycobacteria: *Mycobacterium tuberculosis*, *M. tuberculosis* H37Rv and RESISTANT *M. tuberculosis.* These bacteria cause tuberculosis.

Finally, compounds GAV04 and GAV05 demonstrated potential inhibitory activity against the *Staphylococcus lugdunensis* strain.

In the screening of compounds from pivot 0Y5, the fact that no explicit record of potential activity against resistant *S. aureus* was obtained will not result in the elimination of these compounds from future studies since this result suggests the possibility of identifying and selecting substances with potential activity against other bacteria of medical importance, not only *S. aureus*, which was the initial proposal of this study. Furthermore, there was an indication of potential activity against the genus, represented by the field *Staphylococcus sp*., which will allow testing of these compounds against strains of *S, aureus* as they belong to the indicated genus.

All five compounds for each molecular target showed significant results in the context of an in silico study, drawing attention to the potential antibacterial activity indicated. Experimental studies will be the next important step for the research group that conducted this work and may result in new biotechnological products of pharmaceutical interest and patent registration. The compounds discovered in this work are already in the acquisition process, and new studies from the research group will be published in the future.

Although auxiliary in silico predictions suggested potential interactions with additional bacterial species, these observations should be interpreted as exploratory hypotheses. The present study is focused on *S. aureus*, and any broader-spectrum implications require further computational and experimental validation. The experimental steps in this study will be directed toward the inhibition of resistant strains of *S. aureus,* highlighting compounds CAV02, CAV03, CAV04 and CAV05 and their potential against resistant *S. aureus* subsp. *aureus* MW2.

### 2.7. Prediction of Synthetic Accessibility of the Top 5 Screened Compounds

Table 10 specifies the values obtained for synthetic accessibility. The SwissADME establishes values from 1 (very easy) to 10 (very difficult). For AMBIT-SA the values are: score ≥ 50 (easy); 10 < score ≤ 49 (medium) and score ≤ 10 (difficult).

These findings demonstrate that all the compounds obtained at the end of the virtual screening have an accessibility level classified as “easy” by both tools used for the calculation. In a possible scenario of large-scale compound synthesis, synthetic accessibility classified as “easy” is an excellent factor for laboratory-scale chemical synthesis [40].

### 2.8. Molecular Dynamics Simulations

**Figure 8:**
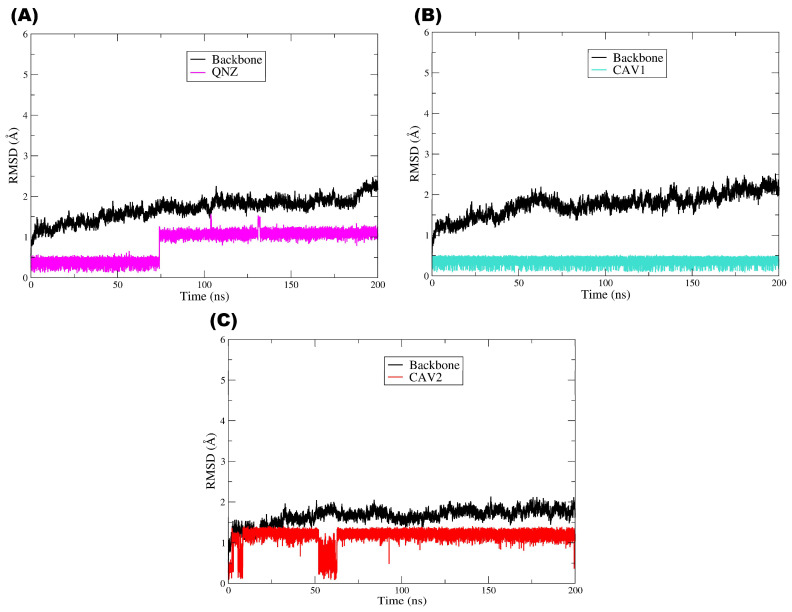
RMSD of compounds QNZ, CAV1, and CAV2 over 200 ns of simulation in PBP2a (PDB: 4CJN). In (**A**): RMSD result of the pivot compound QNZ; In (**B**): RMSD result of the compound CAV1; In (**C**): RMSD result of the compound CAV2.

To assess the conformational stability of the complexes and the persistence of binding interactions over time, 200 ns molecular dynamics simulations were performed for the target protein PBP2a in complex with the pivot compound QNZ and the candidate compounds CAV1 and CAV2. This approach complements the molecular docking results by enabling the evaluation of whether the ligands remain stably accommodated within the binding site under dynamic conditions [50]. As illustrated by the RMSD plot (Figure 8), all the systems reached a structural equilibrium consistent with overall stability throughout the simulations. The RMSD of the PBP2a main chain (Cα) (black line) remained low (approximately ≈ 1.0–2.5 Å) after a brief initial equilibration phase, with no evidence of abrupt significant global conformational changes, although a slight tendency toward a gradual increase over time was observed. This behavior indicates that the presence of each ligand did not destabilize the protein structure, which remained structurally intact throughout the entire molecular dynamics trajectory.

With respect to the ligands, the pivot compound QNZ (magenta) displayed low RMSD values at the beginning of the simulation (≈0.2–0.5 Å) and, at approximately ≈70–80 ns, showed a step-like transition, thereafter oscillating around ≈1.0–1.3 Å until the end of the trajectory, with occasional peaks. This profile is indicative of a conformational rearrangement/realignment of the ligand within the binding site followed by stabilization in a new regime, a phenomenon commonly observed when a ligand explores alternative conformations while remaining associated with the target. Importantly, variations in ligand RMSD do not necessarily imply loss of affinity or dissociation but may instead reflect dynamic conformational adaptations within the binding site [51,52].

Compound CAV1 (cyan line) exhibited a highly stable and low RMSD profile throughout the simulation after a brief initial phase of conformational accommodation. The maintenance of RMSD values within a relatively constant range (≈0.2–0.6 Å) suggests that the ligand remained well fitted within the binding site, displaying a lower degree of structural fluctuation. This behavior is frequently associated with more persistent and energetically favorable binding modes, in agreement with previous molecular docking results [53,54].

Similarly, compound CAV2 (red line) also exhibited dynamic stability after the initial adaptation period, although with fluctuations at a slightly higher plateau than CAV1, remaining predominantly around ≈1.0–1.4 Å. A transient event involving a more pronounced decrease was also observed at approximately ≈50–60 ns, followed by a return to the previous plateau, with no evidence of continuous drift. Nevertheless, the absence of persistent abrupt deviations or a continuous increasing trend in RMSD indicates that the ligand maintained its association with the binding site throughout the simulation, a feature consistent with structurally stable complexes in molecular dynamics studies [55,56].

Overall, the plot demonstrates that all the systems reached a dynamic equilibrium regime characterized by controlled RMSD fluctuations over time. The stability of the protein structure, together with the maintenance of ligand RMSD profiles without a progressive increase compatible with instability, suggest that the protein–ligand complexes are structurally stable under the simulated conditions. A comparative analysis of the ligands further indicates that CAV1 displayed the most constrained profile (lowest RMSD), whereas CAV2 remained stable at a moderate plateau and QNZ exhibited a late rearrangement followed by stabilization, in agreement with the results obtained in the previous molecular docking and computational screening steps, thereby reinforcing the internal consistency of the adopted methodology [54].

Taken together, the RMSD analysis throughout the molecular dynamics simulations indicates that the evaluated protein–ligand complexes exhibit structural stability consistent with well-equilibrated systems from both the protein and ligand perspectives. The maintenance of low conformational deviations in the main chain suggests that ligand binding does not induce relevant structural perturbations in the protein, whereas the RMSD profiles observed for QNZ, CAV1, and CAV2 reflect the dynamic conformational adaptations expected in physiologically simulated environments, with no evidence of dissociation or complex instability. As widely discussed in the literature, moderate fluctuations in ligand RMSD are common and are often associated with the intrinsic flexibility of the molecules and conformational exploration within the binding site rather than indicating loss of affinity or interaction failure. In this context, the RMSD results obtained in this study reinforce the internal consistency of the computational screening workflow, corroborating the molecular docking findings and the favorable pharmacokinetic profiles previously observed. Thus, although limited to the in silico scope, the MD data provide additional support for the selection of CAV1 and CAV2 as promising candidates for future experimental investigations, in line with contemporary molecular dynamics-based computer-aided drug discovery approaches [57].

**Figure 9:**
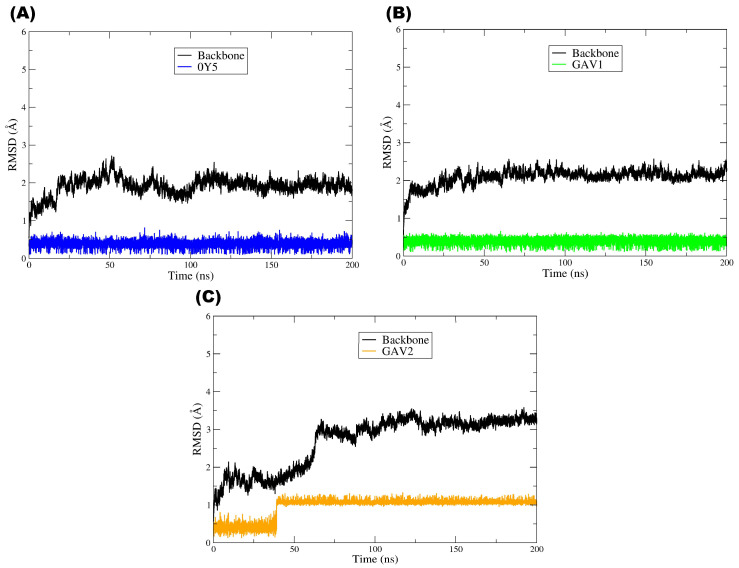
RMSD of compounds 0Y5, GAV01, and GAV02 over 200 ns of simulation in TMK (PDB: 4GSY). (**A**): RMSD result of the pivot compound 0Y5; (**B**): RMSD result of the compound GAV1; (**C**): RMSD result of the compound GAV2.

Figure 9 shows the RMSD plot corresponding to the molecular dynamics (MD) simulations of the TMK target (PDB: 4GSY), illustrating the temporal evolution of the structural deviation of the protein main chain (black line) and of ligands 0Y5 (blue), GAV01 (green), and GAV02 (orange) over 200 ns of simulation. The black line, corresponding to the RMSD of the protein main chain, exhibited low and relatively constant values throughout the simulation, with small fluctuations around ≈1–2.5 Å, although in the system with GAV02 a more pronounced increase in backbone RMSD was observed from approximately ≈60–70 ns onward, reaching a new plateau around ≈3.0–3.5 Å. This indicates that the overall TMK structure remained stable throughout the MD trajectory, showing globally continuous behavior and no persistent abrupt transitions in the systems with 0Y5 and GAV01, whereas the complex with GAV02 suggests a global conformational rearrangement followed by stabilization in a higher RMSD regime [58].

With respect to the pivot compound 0Y5 (blue line), a low and stable RMSD profile was observed throughout the 200 ns simulation, with only small fluctuations after the initial accommodation phase, remaining predominantly around ≈0.2–0.6 Å. This behavior suggests that the ligand remained firmly associated with the TMK catalytic site throughout the simulation, reflecting the high affinity previously reported for this compound in experimental and computational studies. The conformational stability of 0Y5 during the molecular dynamics simulations is consistent with its role as the structural and functional reference in this study, serving as a comparative parameter for the selected compounds [19].

Compound GAV01 (green line) exhibited RMSD values similar to those of the pivot ligand 0Y5, stabilizing at approximately ≈0.2–0.6 Å after the initial nanoseconds of the simulation. Despite these discrete fluctuations, the RMSD remained within a relatively constant range over time, with no trends toward progressive increase or abrupt instability events. This profile suggests that the ligand remained accommodated within the binding site while exploring local/fine conformational adjustments, a behavior frequently observed for ligands that retain a persistent binding mode [54].

In turn, compound GAV02 (orange line) displayed distinct behavior, with a step-like transition occurring at approximately ≈35–45 ns. Initially, the RMSD oscillated around ≈0.2–0.6 Å and, after this transition, stabilized at a new level around ≈1.0–1.3 Å, maintaining moderate fluctuations until the end of the simulation. This behavior indicates ligand rearrangement/realignment within the catalytic site followed by stabilization, suggesting dynamic adaptation of the compound within a flexible protein environment. However, the absence of a continuous or irreversible increase in RMSD suggests that these fluctuations do not necessarily correspond to ligand dissociation from the catalytic site but rather to dynamic adaptation of the compound within a flexible protein environment. Previous studies have shown that larger ligands or those with multiple rotational degrees of freedom may display moderately elevated RMSD values while still maintaining relevant interactions with the biological target [59].

Overall, the plot indicates that all the systems reached a dynamic equilibrium regime characterized by controlled RMSD fluctuations over time. The structural stability of the TMK main chain, together with ligand retention within the binding site throughout the simulation, suggest that the protein–ligand complexes are dynamically stable under the simulated conditions, particularly 0Y5 and GAV01, which maintained low and stable ligand RMSD, and GAV02, which stabilized after a ligand step transition and a backbone readjustment into a higher RMSD regime.

**Figure 10:**
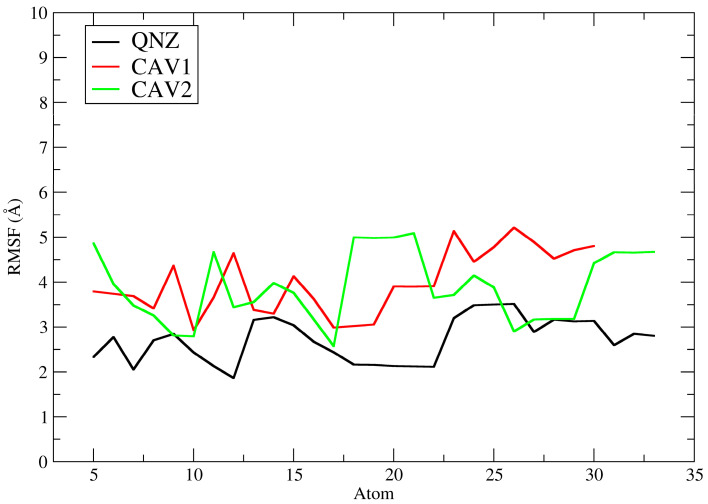
Ligand root mean square fluctuation (L-RMSF) of ligands QNZ, CAV1, and CAV2 for the PBP2a target (PDB: 4CJN).

Figure 10 depicts the ligand root mean square fluctuation (L-RMSF) of ligands QNZ, CAV1, and CAV2 for the PBP2a target (PDB: 4CJN). The L-RMSF plot describes the root mean square fluctuation of each ligand atom for QNZ (black), CAV1 (red), and CAV2 (green) throughout the molecular dynamics simulations, allowing assessment of the internal flexibility of the ligands when complexed with the allosteric site of PBP2a. Unlike RMSD, which provides a global measure of structural displacement, RMSF offers local and detailed information on which regions of the ligand exhibit greater or lesser mobility during the simulation [56].

In general, the pivot compound QNZ presented the lowest RMSF values across nearly its entire molecular structure, with fluctuations reaching approximately ≈2.0 to 3.5 Å and moderate peaks in specific regions. This behavior indicates lower intramolecular conformational flexibility, suggesting that different fragments of QNZ explored a more restricted conformational space while remaining associated with the allosteric site. This profile is consistent with ligands that, although biologically active, retain greater relative rigidity and a lower propensity for extensive dynamic rearrangements within the protein cavity [60].

In contrast, compound CAV1 exhibited higher RMSF values than QNZ, with fluctuations predominantly concentrated between ≈3.0 and 5.2 Å across almost all its atoms, including more pronounced peaks in specific segments. This pattern suggests greater intramolecular flexibility and less restricted conformational behavior during the simulation, indicating that the ligand remained well accommodated within the binding site yet with a greater degree of internal freedom. According to the literature, higher ligand RMSF values may reflect greater mobility of peripheral moieties, often more solvent-exposed, even in stable complexes, particularly when combined with favorable affinity profiles obtained in molecular docking studies [53].

**Figure 11:**
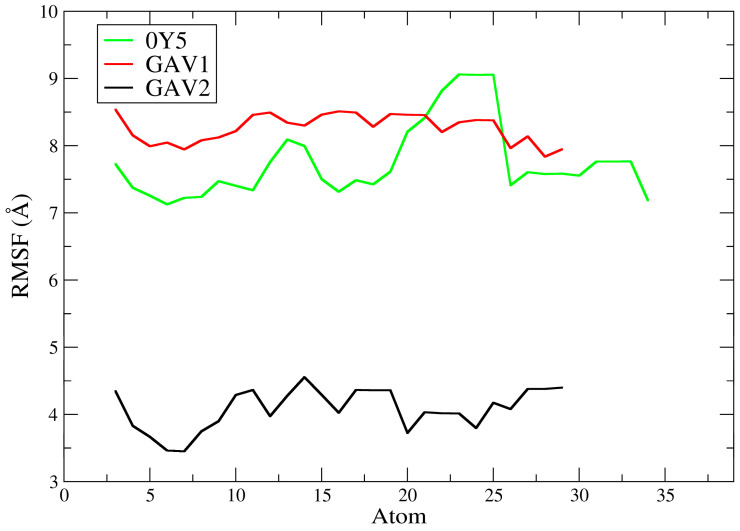
Ligand root mean square fluctuation (L-RMSF) of ligands 0Y5, GAV01, and GAV02 for the TMK target (PDB: 4GSY).

Compound CAV2 showed a globally similar profile to that of CAV1 and higher than that of QNZ, with RMSF values generally ranging from ≈2.8 to 5.1 Å (Figure 11). Notably, however, CAV2 exhibited a marked increase in one specific region (approximately atoms ≈18–21), reaching values close to ≈5.0–5.1 Å, followed by a reduction to more moderate ranges. This profile indicates that CAV2 displayed moderate to high flexibility, with certain more mobile regions, possibly associated with more exposed functional groups or with fewer stabilizing interactions with the protein. Nevertheless, the observed pattern suggests that the ligand maintained a relatively stable conformation within the allosteric site throughout the simulation, in agreement with the RMSD results.

A direct comparison among the three ligands reveals that QNZ displayed the lowest internal fluctuations, whereas CAV1 and CAV2 exhibited greater internal fluctuations, indicating more mobile conformational behavior during the molecular dynamics simulations. This finding is particularly relevant in the context of the computational screening conducted in this study since these compounds were selected based on more favorable docking affinity energies and advantageous pharmacokinetic and toxicological profiles. Thus, the L-RMSF data suggest that the screened compounds not only maintain relevant interactions with PBP2a but also exhibit greater intramolecular mobility than the pivot compound, which may reflect local conformational adaptations within the binding site without necessarily implying loss of complex stability.

The pivot compound 0Y5 exhibited high RMSF values, predominantly around ≈7.1–9.1 Å across its entire molecular structure. A more pronounced increase was observed approximately between atoms ≈20 and 26, reaching a plateau close to ≈9.0–9.1 Å, followed by a reduction to a plateau around ≈7.2–7.8 Å in the terminal portions of the ligand. This profile indicates high intramolecular flexibility during the simulation, suggesting that the ligand may explore multiple internal conformations while remaining accommodated within the TMK catalytic site, with substantial fluctuations. This finding does not necessarily contradict the statement that ligand 0Y5 has high affinity for the bacterial enzyme [19] since more mobile peripheral regions may coexist with stable core interactions and should be interpreted together with the profile observed in the RMSD analysis.

In contrast, compound GAV01 also presented high RMSF values but with a smaller amplitude of variation than 0Y5, remaining mostly between ≈7.8 and 8.6 Å, with relatively continuous fluctuations and no extremely pronounced abrupt peaks. This pattern indicates a high degree of conformational flexibility, suggesting that different ligand fragments explored multiple conformations during the dynamics. Such behavior may be associated with the presence of more exposed functional groups or a reduced number of stabilizing interactions with residues in the TMK catalytic site. As described in the literature, highly flexible ligands may exhibit elevated RMSF values while remaining associated with the target, reflecting dynamic adaptation within the enzymatic cavity [57].

Compound GAV02 exhibited substantially more restricted behavior, with RMSF values predominantly around ≈3.4–4.6 Å across almost its entire molecular structure without increasing into the 7–9 Å range observed for 0Y5 and GAV01. This profile suggests that, although the ligand still displayed internal mobility, it was globally less flexible than GAV01 and 0Y5, maintaining moderate fluctuations across the analyzed atoms. Such behavior is frequently observed in ligands that retain a stable structural core within the binding site, while peripheral groups explore alternative conformations of smaller amplitude without compromising the overall association with the protein [54].

A comparative analysis among the three ligands indicates that GAV02 exhibited the most restricted dynamic behavior (lowest L-RMSF), whereas GAV01 and 0Y5 displayed the greatest intramolecular flexibility, with 0Y5 showing the largest localized increase (≈atoms 20–26). These findings are consistent with the RMSD data discussed previously, in which 0Y5 and GAV01 exhibited low and stable RMSD, whereas GAV02 showed ligand rearrangement with a step transition and an increase in backbone RMSD to a higher regime, indicating that internal mobility (L-RMSF) and global stability (RMSD) may capture distinct aspects of the dynamic behavior of the complex. Taken together, the L-RMSF results suggest that the selected compounds remained associated with the TMK catalytic site, although they differed substantially in their degree of internal flexibility during the simulation.

It is important to note, however, that lower RMSF values do not necessarily imply greater biological activity, but rather indicate more restricted dynamic behavior under the simulated conditions. Therefore, the L-RMSF results should be interpreted as complementary evidence of the ligand’s intramolecular conformational flexibility within the protein–ligand complex, contributing to the rational prioritization of candidates in in silico studies, without extrapolation beyond the computational scope of the present work [57]. It is worth emphasizing that efficacy and biological activity studies will be conducted in future in vitro and in vivo phases of this research. Our computational results reinforce the predicted selection of these ligands for future experimental stages, further supporting the role of the computational approach in saving resources and directing future investigations toward the molecules with the best results through the in silico approach.

**Figure 12:**
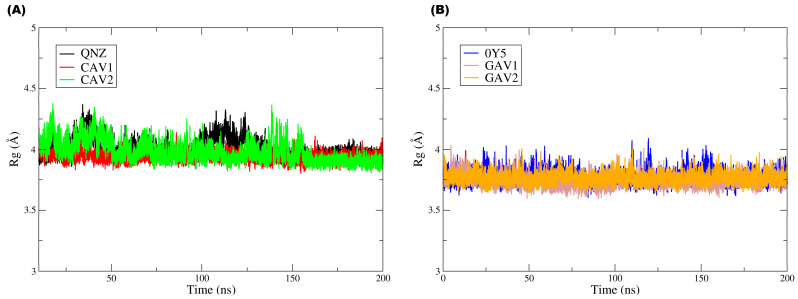
Radius of gyration of the compounds for each target. (**A**): Represents the radius of gyration of compounds QNZ, CAV1, and CAV2; (**B**): Represents the radius of gyration of compounds 0Y5, GAV1, and GAV2.

Figure 12 groups the Rg plots for both targets and their respective ligands, with Figure 12A corresponding to QNZ, CAV1, and CAV2 and Figure 12B corresponding to 0Y5, GAV01, and GAV02.

In Figure 12B, it can be observed that the three ligands displayed very similar Rg values (around ≈3.9–4.2 Å), with moderate fluctuations over 200 ns and no continuous drift or persistent abrupt changes. Compound QNZ did not exhibit the lowest mean Rg values; on the contrary, it showed moderate fluctuations and discrete episodes of transient increase (approximately around ≈100–120 ns), reaching values close to ≈4.2 Å, suggesting small temporary conformational expansions during the simulation. Compound CAV1 (red) generally displayed the most constrained profile, with slightly lower Rg values (≈3.9–4.0 Å) and lower fluctuation amplitude, indicating a more compact and stable overall conformation over time. Compound CAV2 (green) maintained Rg values within the same general range but with greater variability and more frequent transient peaks than CAV1, reflecting episodes of reversible conformational expansion. The absence of prolonged maintenance of these peaks suggests that such events correspond to adaptive rearrangements, with no evidence of persistent ligand destabilization.

Ligands 0Y5, GAV01, and GAV02 also exhibited similar Rg values (≈3.6–4.0 Å), with moderate fluctuations and no trend toward progressive increase throughout the trajectory. Ligand 0Y5 (blue) was not the one with the lowest Rg values; rather, it oscillated around ≈3.7–3.9 Å, with occasional peaks approaching ≈4.0 Å, suggesting slight variation in overall compactness over time. Compound GAV01 (gray) tended to present the lowest mean values and the most stable profile, remaining predominantly around ≈3.6–3.8 Å, which is compatible with greater overall compactness. Compound GAV02 (orange) exhibited values very similar to those of GAV01, with slightly more evident fluctuations in some segments but without standing out as having the highest mean Rg in the set, indicating moderate and reversible global conformational flexibility.

The Rg results indicate that none of the ligands exhibited progressive loss of structural compactness during the molecular dynamics simulations, reinforcing that the fluctuations observed in the other metrics reflect natural and reversible conformational adaptations under dynamic conditions rather than structural destabilization.

**Figure 13:**
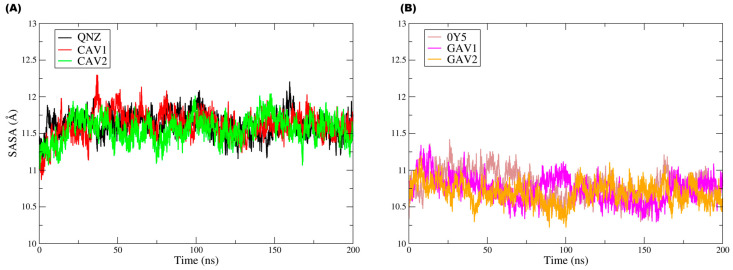
Solvent-accessible surface area (SASA) of the compounds for each target. (**A**): Solvent Accessible Surface Area QNZ, CAV1, and CAV2; (**B**): Solvent Accessible Surface Area 0Y5, GAV1, and GAV2.

Figure 13 shows the unified solvent-accessible surface area (SASA) plot.

The analysis of the solvent-accessible surface area (SASA) throughout the simulation provides complementary information on solvent exposure and on possible global conformational rearrangements along the trajectory, allowing evaluation of the ligand surface area accessible to water molecules.

For the ligands in the PBP2a complexes (QNZ, CAV1, and CAV2), the SASA profiles reveal that the systems remained globally stable in terms of solvent exposure, with no continuous increasing or decreasing trends that would indicate structural opening events or conformational collapse. An initial increase in SASA was observed during the first nanoseconds, followed by stabilization at a relatively constant plateau, with moderate fluctuations throughout the 200 ns simulation. Approximately, the values were concentrated mainly around ≈11.2–11.9 Å, with occasional peaks near ≈12.0–12.2 Å, particularly for CAV1 and QNZ in some intervals. The moderate fluctuations in solvent-accessible area are consistent with local rearrangements and transient reorientation of exposed surfaces without compromising the overall structural organization or protein–ligand association. The SASA values oscillated within well-defined ranges, suggesting that the observed variations reflect natural dynamic conformational adjustments of the system in solution rather than persistent structural instability, as corroborated by the RMSD and L-RMSF data.

Likewise, for the TMK complexes (0Y5, GAV1, and GAV2), the SASA analysis indicated stability with respect to solvent exposure, with no evidence of pronounced structural opening or conformational collapse throughout the trajectory. In panel (B), the SASA values remained predominantly around ≈10.4–11.1 Å, with moderate fluctuations and no continuous drift. 0Y5 tended to occupy slightly higher values during part of the trajectory, especially in the early-to-middle simulation period, whereas GAV1 and GAV2 displayed comparable oscillations, with small transient depressions, for example around ≈80–110 ns, before returning to the mean plateau. In both plots, the SASA values remained confined within well-defined intervals, suggesting that the observed variations correspond to transient and reversible conformational rearrangements typical of biologically relevant systems in aqueous environments.

Taken together, the molecular dynamics data provide an integrated and consistent view of the dynamic behavior of the evaluated complexes. The low conformational deviations of the protein main chain indicate that ligand binding does not compromise the overall structural stability of the studied targets, whereas the ligand RMSD and RMSF profiles reflect dynamic conformational adaptations that are compatible with well-equilibrated systems, with no evidence of dissociation from the binding sites. In addition, the SASA profiles demonstrate that solvent exposure remained stable within narrow ranges, corroborating the absence of pronounced global conformational changes during the trajectories. Although differences in the degree of flexibility and interaction patterns were observed among the compounds, ligands CAV1 and CAV2, as well as GAV1 and GAV2, maintained continuous association with their respective functional sites, showing data consistent with the criteria adopted in the computational screening. Thus, within the limitations inherent to exclusively in silico studies, the results obtained corroborate that these ligands display satisfactory structural and dynamic properties, making them suitable for future in vitro and in vivo experimental evaluation, where their biological activity and pharmacological profiles may be properly validated.

Table 11 and Table 12 present the binding affinity energy (MM/GBSA) values for the ligands evaluated in complexes with PBP2a and TMK, calculated from the molecular dynamics simulations. In all cases, negative ΔGbinding values were observed, indicating favorable ligand association with their respective targets. For the PBP2a set, the predicted affinity ranking (most favorable → least favorable) was QNZ (−31.48 kcal/mol) > CAV2 (−29.43 kcal/mol) > CAV1 (−27.10 kcal/mol). For the TMK set, the hierarchy was 0Y5 (−26.81 kcal/mol) > GAV1 (−25.32 kcal/mol) > GAV2 (−23.46 kcal/mol).

Table 11 presents the affinity energy values for ligands QNZ, CAV1, and CAV2 in complex with PBP2a (PDB: 4CJN), calculated by MM/GBSA. The analyzed energetic components include van der Waals interactions (ΔEvdW), electrostatic energy (ΔEele), polar solvation energy (ΔGGB), nonpolar solvation energy (ΔGNP), and total binding energy (ΔGbinding).

For QNZ, a marked contribution from van der Waals interactions was observed (ΔEvdW = −36.50 kcal/mol), together with electrostatic interactions (ΔEele = −12.80 kcal/mol), partially offset by the polar solvation penalty (ΔGGB = +22.10 kcal/mol). Nonpolar solvation contributed favorably (ΔGNP = −4.28 kcal/mol), resulting in ΔGbinding = −31.48 kcal/mol. This profile indicates that the energetic gain is predominantly vdW-driven, with electrostatic reinforcement, whereas the polar term (ΔGGB) imposes a consistent penalty, as expected in GB models.

For CAV2, ΔGbinding remained close to that of the pivot compound (ΔGbinding = −29.43 kcal/mol), supported by strongly favorable van der Waals interactions (ΔEvdW = −34.80 kcal/mol) and a relevant electrostatic contribution (ΔEele = −11.20 kcal/mol). As observed for QNZ, there was a substantial polar penalty (ΔGGB = +20.05 kcal/mol), partially compensated by the nonpolar term (ΔGNP = −3.48 kcal/mol). In the overall balance, CAV2 exhibited robust complex stabilization, mainly through packing/dispersion (vdW), but with a slightly less intense nonpolar contribution and somewhat weaker attractive interactions than QNZ, explaining its less negative ΔGbinding.

For CAV1, the total binding energy was the least favorable of the set (ΔGbinding = −27.10 kcal/mol). This result arises mainly from weaker attractive contributions (ΔEvdW = −31.45 kcal/mol; ΔEele = −9.51 kcal/mol) and a less favorable nonpolar term (ΔGNP = −2.90 kcal/mol) despite presenting the lowest polar solvation penalty among the three (ΔGGB = +16.76 kcal/mol). Thus, the limiting factor for CAV1 is the lower overall efficiency of dispersion and electrostatic contacts rather than an excessive polar cost.

Table 12 presents the affinity energy values for ligands 0Y5, GAV1, and GAV2 in complex with TMK (PDB: 4GSY), calculated by MM/GBSA, considering the same components (ΔEvdW, ΔEele, ΔGGB, ΔGNP, and ΔGbinding).

For 0Y5, the most negative ΔGbinding of the TMK set was observed (ΔGbinding = −26.81 kcal/mol), driven by attractive van der Waals (ΔEvdW = −30.90 kcal/mol) and electrostatic contributions (ΔEele = −9.80 kcal/mol), as well as a favorable nonpolar contribution (ΔGNP = −3.21 kcal/mol) despite a relatively larger polar penalty (ΔGGB = +17.10 kcal/mol). This energetic balance suggests that, also in TMK, the driving force is predominantly vdW, with an additional electrostatic contribution that is partially counteracted by polar desolvation.

For GAV1, ΔGbinding was close to that of 0Y5 (ΔGbinding = −25.32 kcal/mol), with slightly less intense attractive contributions (ΔEvdW = −29.60 kcal/mol; ΔEele = −9.10 kcal/mol) and a less favorable nonpolar term (ΔGNP = −2.62 kcal/mol) but with reduced polar penalty (ΔGGB = +16.00 kcal/mol). This balance explains the moderate difference relative to the pivot ligand.

For GAV2, the least negative ΔGbinding was observed (ΔGbinding = −23.46 kcal/mol), mainly reflecting reduced attractive contributions (ΔEvdW = −27.80 kcal/mol; ΔEele = −8.40 kcal/mol) and a less intense ΔGNP (ΔGNP = −2.66 kcal/mol) despite the lowest polar penalty in the set (ΔGGB = +15.40 kcal/mol). Therefore, the reduction in vdW/electrostatic contributions predominates in the balance and governs the lower energetic favorability of GAV2.

Comparatively between the targets, the PBP2a complexes exhibited more negative ΔGbinding values (QNZ: −31.48; CAV2: −29.43; CAV1: −27.10 kcal/mol) than the TMK complexes (0Y5: −26.81; GAV1: −25.32; GAV2: −23.46 kcal/mol), suggesting a more pronounced relative energetic preference in the PBP2a set under the same MM/GBSA formalism. This difference is consistent with stronger vdW contributions in PBP2a (ΔEvdW up to −36.50 kcal/mol) compared with TMK (ΔEvdW up to −30.90 kcal/mol), whereas PBP2a also imposes larger polar penalties (ΔGGB up to +22.10 kcal/mol) than TMK (ΔGGB up to +17.10 kcal/mol), reflecting a higher desolvation cost.

The MM/GBSA results reinforce that the affinity hierarchy is governed primarily by ΔEvdW (packing/dispersion) and, secondarily, by ΔEele and ΔGNP, whereas ΔGGB acts as a counterbalancing penalty in all cases. Within the limitations inherent to MM/GBSA, and considering that moderate differences (≈1–3 kcal/mol) should be interpreted with caution, the energetic data support the prioritization of CAV2 (for PBP2a) and GAV1 (for TMK) as promising candidates as they combine more favorable ΔGbinding with dynamic profiles previously compatible with stable complexes (RMSD/RMSF/Rg/SASA), with no evidence of global instability in the analyzed trajectories.

### 2.9. Limitations and Future Perspectives

This study was designed as an in silico screening and prioritization workflow to identify promising chemical entities with potential inhibitory activity against PBP2a and *S. aureus* thymidylate kinase. Although extensive molecular dynamics simulations were performed to assess the stability and dynamic behavior of the selected protein–ligand complexes, some limitations inherent to computational studies must be acknowledged.

Firstly, no experimental validation was conducted within the scope of this work. Therefore, the binding affinities, interaction patterns, and predicted stability profiles should be interpreted as hypothesis-generating results requiring confirmation through in vitro and in vivo assays. Secondly, selectivity against non-target bacterial or human proteins was not evaluated and represents an important aspect for future investigations during the lead compound optimization stages.

Despite these limitations, the integrated computational framework employed here provides a rational and cost-effective strategy to constrain the chemical space and guide experimental efforts. Future studies will focus on experimental validation and selectivity characterization to further assess the therapeutic potential of the identified candidates.

## 3. Materials and Methods

### 3.1. Template Compound Selection

Potential new compounds to be screened via virtual screening were based on structural similarity with the selected pivot compounds based on the assumption that “similar molecules exert similar pharmacological activities” [61,62].

**Figure 14:**
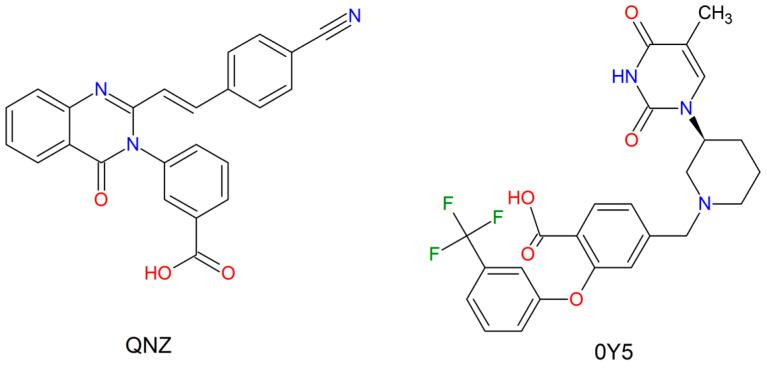
2D structure and conformation of pivotal compounds extracted from the Protein Data Bank.

The selected pivot compounds were: (E)-3-(2-(4-cyanostyryl)-4-oxoquinazolin-3(4H)-yl)benzoic acid and 4-((3-(5-Methyl-2,4-dioxo-3,4-dihydropyrimidin-1(2H)-yl)-piperidin-1-yl)methyl)-2-(3-(trifluoromethyl)phenoxy)benzoic acid. In this study the compounds had their IUPAC nomenclatures abbreviated to QNZ and 0Y5, respectively, for better reading. Since these two chemical entities are crystallized together with the target proteins selected for this study, the conformation adopted as a template for screening in the chosen database was the bioactive conformation resulting from the X-ray crystallography of the target structures deposited in the Protein Data Bank (PDB), as shown in (Figure 14).

### 3.2. Virtual Screening of New Compounds with Potential Antimicrobial Activity

Virtual screening consists of searching for new drug candidates based on existing databases, directing the search towards the desired properties (potency, selectivity, and favorable pharmacokinetic and toxicological properties), in addition to enabling the elimination of undesirable properties (inactive, reactive, toxic, and inappropriate pharmacokinetic effects) [63].

The Tanimoto index was adopted to search for new candidates, and this screening algorithm searches for the similarity of compounds in an online database or downloaded to a local computer, where the algorithm was going to identify which structural regions of the template compound had an equivalence with the compounds deposited in the database on a scale ranging from 0 to 1, where, the closer to 1, the greater the similarity between the template compound and the compounds in the database. The Tanimoto index is based on the XYZ coordinates of the atoms, resulting in a percentage of similarity when obtaining the results. The database selected for this study was the MolPort company (Beacon, NY, USA). The criterion for choosing this database is that virtual screening was going to allow the identification of synthesized compounds available in stock for purchase and continuation for in vitro tests after the computational prediction steps of the present study. The MolPort database (https://www.molport.com/shop/index (accessed on 6 September 2024)) contains a library with more than 51 million compounds.

The compounds were obtained using the “Structure Search” tool on the MolPort website, with the server limited to a maximum of the top 10,000 entries. The results are automatic according to the server’s algorithm, always listing the 10,000 best compounds according to the selected search criteria. In this study, the Tanimoto index of 0.5 (50%) of similarity to the pivot compounds (QNZ and 0Y5) was adopted. In the end, 10,000 compounds were obtained for each template molecule, resulting in a bank of 20,000 compounds for this study, which were downloaded from MolPort in .sdf format.

### 3.3. Pharmacokinetic and Toxicological Prediction of Compounds Obtained from Virtual Screening

The initial screening for this study was performed using Discovery Studio software version 16.0.1. In this initial stage, the 20,000 compounds obtained via MolPort were entered into Discovery Studio and their ADMET (absorption, distribution, metabolism, excretion and toxicity) descriptors were obtained. The calculated properties were: aqueous solubility, penetration of the blood–brain barrier, binding to the CYP2D6 enzyme, hepatotoxicity, intestinal absorption and binding to plasma protein. The inclusion criteria for selecting promising compounds were based on the values of the template molecules, seeking to select those compounds with similar or better pharmacokinetic properties. Molecules that did not meet the inclusion criteria were removed from the database and did not proceed to the next stage.

The compounds selected within the pharmacokinetic inclusion criteria proceeded to the calculation of toxicological properties in Discovery Studio, with the criteria calculated according to the standard Discovery specification: Topkat Mouse NTP—Mouse Male and Female; Topkat Rat NTP—Rat Male and Female; Topkat Mouse FDA—Mouse Male and Female; Topkat Rat FDA—Male and Female; Topkat Woe; Topkat Ames and Topkat DTP.

### 3.4. Molecular Docking of the Screened Compounds

**Figure 15:**
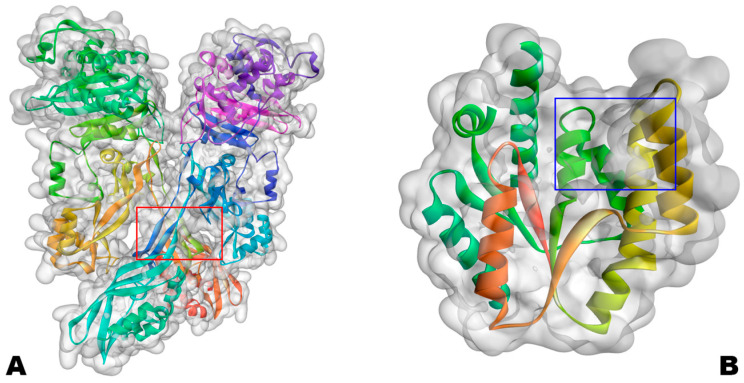
In (**A**)—PBP2a (PDB ID: 4CJN); the red rectangle represents the site of action of QNZ; in (**B**)—TMK (PDB ID: 4GSY); the blue rectangle represents the site of action of 0Y5.

Targets selected for molecular docking were two proteins from the enzyme class: PBP2a (PDB ID: 4CJN) and VRSA (PDB ID: 4GSY). These proteins are penicillin binding protein 2a and thymidylate kinase, respectively; see Figure 15.

The selection of penicillin-binding protein 2a (PBP2a) and thymidylate kinase (TMK) as molecular targets was guided by their biological relevance in antibiotic resistance and bacterial survival. PBP2a is a key determinant of methicillin resistance in *Staphylococcus aureus*, enabling cell wall biosynthesis even in the presence of β-lactam antibiotics. In contrast, TMK is an essential enzyme in the de novo synthesis of thymidine monophosphate, playing a critical role in DNA replication and cell proliferation. By targeting two functionally distinct yet essential enzymes, the screening strategy aimed to explore innovative chemical structures in complementary bacterial pathways, thus increasing the relevance and translational potential of investigating antibacterial potentials for these enzymes fundamental to bacterial survival [18,19,64].

Interactions with amino acid residues were compared with crystallographic pivot molecules in the receptors since the coupled compounds are in the protein’s biological interaction cavity where the biological effect was observed. Visualization of intermolecular interactions was performed using Discovery Studio software.

In preparation for molecular docking, the biological targets PDB ID: 4CJN (PBP2a) and PDB ID: 4GSY (TMK) were downloaded in .pdb format and read in the Swiss-PdbViewer 4.1.0 software. This software has an automatic tool that analyzes the amino acid chains of the files and corrects any missing amino acids in the chain. Among the targets used, only 4GSY was found to have missing amino acid residues in the .pdb file, and this absence was corrected using the Swiss-PdbViewer 4.1.0. Target 4CJN had all its amino acid residues intact and did not require correction [65,66].

Afterward, the targets had their three-dimensional structures optimized to the lowest energy conformation using the UCSF Chimera 1.18 software, using the software’s default parameters according to the developer’s protocol [67]. Finally, the protonation states of the amino acid residues were corrected using the APBS-PDB2PQR web server (https://server.poissonboltzmann.org/pdb2pqr (accessed on 6 September 2024)), with the protonation settings adopted: pH of 7.0 for the PBP2a target and 7.5 for TMK; for both targets, PROPKA was used to calculate the protonation of the residues, and the force field assigned was PARSE [68].

The compounds selected via pharmacokinetic and toxicological screening were drawn and pre-optimized in 3D using the Freeware ACD/ChemSketch 12.0 software [69]. The next step for the ligands was optimization in the Molecular Mechanics database using the MM+ method in the HyperChem Plus 8.0.8 software. The ligands, now ready to be sent to the DockThor web server, were saved in .mol2 format. Docking simulations were performed with 24 independent runs per ligand, generating 10 poses per run. Ligands were treated as flexible, while the receptor was kept rigid, and the best-ranked pose according to binding energy was selected for analysis.

For molecular docking, the DockThor webserver (https://dockthor.lncc.br/v2/ (accessed on 6 September 2024)) was used. The docking affinity data were tabulated in order from the lowest to the highest affinity energy [70,71,72]. Table 13 specifies the settings adopted for the molecular docking protocol. At the end of the molecular docking methodological stage, the 5 best compounds were selected based on the best affinity energy. At the end of the molecular docking stage, the five best compounds were selected based on the best affinity energy

As a nomenclature criterion for the top 5 compounds with the best affinity energy for each of the two molecular targets, the nominal code was adopted: the top 5 compounds for the PBP2a target (PDB ID: 4CJN) received the code 4CAV; the top 5 compounds for the TMK target (PDB ID: 4GSY) received the code 4GAV. For a complete list of all compounds selected in the pharmacokinetic/toxicological analysis that proceeded to molecular docking, see the Supplementary Materials.

### 3.5. Lethal Dose and Toxicity Class of the Top 5 Compounds Obtained via Molecular Docking

After completing the screening of the database with the 10,000 MolPort compounds screened for each molecular target, the top 5 molecules for each target studied had their parameters for toxicity class and lethal dose obtained via the ProTox webserver [73,74].

### 3.6. Lipophilicity, Water Solubility and Lipinski’s Parameters

To obtain the dissolution parameters in fat and water of the selected promising compounds, the SwissADME tool (http://www.swissadme.ch/ (accessed on 16 July 2025)) was used [40].

Lipinski parameters, also known as the rule of five, are physicochemical descriptors that allow us to assess whether a substance has good oral bioavailability. Thus, the substance must present the following parameters: molecular weight less than 500 g/mol, LogP (lipophilicity) less than five, a maximum of five hydrogen donor groups and a maximum of ten bond acceptor groups, thus presenting satisfactory values for intestinal permeability [44]. Lipinski descriptors were obtained via the SwissADME webserver [40].

### 3.7. Prediction of Antibacterial Activity in Silico

For the prediction of antibacterial activity in silico, the AntiBac Pred tool from the PASS Online WebServer (https://way2drug.com/PassOnline/definition.php (accessed on 16 July 2025)) was used. This tool indicates the degree of confidence of the inhibition of a compound against different strains of bacteria [61,75].

### 3.8. Synthetic Accessibility Prediction

For synthetic accessibility, the SwissADME [40] and AMBIT-SA [42] tools were used. The heats obtained for synthetic accessibility were 1–10 for SwissADME and 1–100 for AMBIT-SA. Synthetic accessibility is a fundamental step in drug development, especially regarding large-scale production of compounds.

### 3.9. Molecular Dynamics

RESP charges for the compounds were derived at the HF/6-31G [76] level of theory, as previously reported. The ligand parameters were generated with Antechamber and assigned using the General AMBER Force Field (GAFF) [77]. Molecular dynamics (MD) simulations were carried out with AMBER 18 [78,79,80]. The tLEaP module was used to add missing hydrogen atoms to the protein structures, and the proteins were described with the ff14SB force field [81]. The systems were solvated with TIP3P [82] water molecules in a periodic truncated octahedral box. A 12 Å cutoff was applied in all directions. Counterions were added to neutralize the systems.

Energy minimization was performed using the sander.MPI module. First, water molecules and ions were minimized using 2000 cycles of steepest descent followed by 3000 cycles of conjugate gradient. Next, receptor–ligand hydrogen atoms were minimized using 4000 steps of steepest descent and 3000 steps of conjugate gradient. In the third stage, hydrogen atoms, water molecules, and ions were further minimized using 2500 steps of steepest descent and 3500 steps of conjugate gradient. Finally, the entire system was minimized using 3000 steps of steepest descent followed by 3 steps of conjugate gradient.

The complexes were then heated to 300 K in five stages over a total of 500 ps. During the first four stages, a harmonic force constant of 25 kcal mol^−1^ Å^−2^ was applied to restrain heavy atoms; in the final stage, the restraint force constant was reduced to zero. The systems were subsequently equilibrated by running 5 ns of MD at 300 K without any restraints. Production MD simulations were then performed for a total of 200 ns.

Electrostatic interactions were treated using the Particle Mesh Ewald (PME) [83] method, and bonds involving hydrogen atoms were constrained using the SHAKE algorithm [84]. Temperature control was achieved with a Langevin thermostat [85] using a collision frequency of 2 ps^−1^.

### 3.10. Free Energy Calculations

The free energy of each complex was obtained from the last 5 ns of the trajectory corresponding to 500 snapshots. In the MM-GBSA approach, binding free energy is calculated from the free energy of a linker interacting with a receptor to form the complex [86,87]. Equation (1) is related to this phenomenon:

ΔG = ΔG – ΔG − ΔG(1)

In each state, the free energy is calculated through the following expression:

ΔG = ΔG + ΔG − TΔS(2)

ΔEMM is the energy of the total molecular mechanics in the gas phase, ΔGsolv is the free energy of solvation, and TΔS is the entropy of the system.

EMM represents the sum of the internal energy contributions (ΔEinternal, sum of the binding energies, angles and dihedrals), eletrostatic interactions (ΔEelectrostatic), and contributions of van der Waals (ΔEvdw), according to the equation:

ΔE = ΔE + ΔE + ΔE(3)

The free energy of solvation (ΔGsolv), in Equation (3), is composed of polar (ΔGGB) and nonpolar (ΔGSASA) contributions. Polar contributions are approximated by the Generalized Born (GB) method, and the nonpolar contributions are determined from the calculation of the solvent-accessible surface area (SASA):

ΔG = ΔG + ΔG(4)

## 4. Conclusions

In this study, complementary virtual in silico screening strategies were employed using the key compounds QNZ and 0Y5, both previously reported as exhibiting biological activity against *Staphylococcus aureus*. From an initial molecule bank comprising approximately 20,000 compounds, a hierarchical computational workflow integrating similarity-based screening, molecular docking, pharmacokinetic and toxicological predictions, and molecular dynamics simulations was applied to identify potential inhibitors of PBP2a and thymidylate kinase (TMK) enzymes.

This approach resulted in the selection of five promising compounds for each molecular target, totaling ten candidate molecules with favorable in silico profiles. ADMET analyses highlighted compounds with physicochemical and pharmacokinetic properties that are compatible with oral administration, while toxicological predictions indicated the absence of significant carcinogenic or toxic risks for the screened candidates, in contrast to the key compounds, which showed some degree of predicted toxicity. Molecular modeling allowed for the prioritization of the best-ranked compounds based on binding affinity, exhibiting the most favorable predicted binding energies for PBP2a and TMK, respectively.

To overcome the limitations of molecular modeling as a static approach, molecular dynamics simulations were performed, providing a dynamic assessment of protein–ligand stability, interaction persistence, and conformational behavior over time. The results demonstrated that the selected compounds maintained stable interactions with their respective targets, exhibiting dynamic behavior comparable to or more stable than that of the main ligands under simulated physiological conditions. Furthermore, lipophilicity and aqueous solubility profiles, along with Lipinski parameters and toxicity class predictions, corroborated the suitability of these compounds as promising early-stage candidates.

Finally, auxiliary predictions using AntiBac-Pred suggested potential antibacterial activity against resistant strains of *S. aureus* and other clinically relevant bacteria. However, these predictions are intended only for hypothesis generation to guide experimental prioritization. In general, the conclusions of this study are based exclusively on computational analyses and should be interpreted accordingly. Experimental validation through in vitro and in vivo assays, as well as selectivity characterization, is necessary to confirm the biological activity and therapeutic potential of the identified compounds. Nevertheless, the integrated in silico structure presented here provides a rational strategy that seeks to optimize cost and investment for the identification of promising compounds and supports the advancement of these candidates towards future experimental investigations.

## Figures and Tables

**Table 1:** Hit registration of pivot compounds at different Tanimoto indices in the MolPort database.

Database	Tanimoto Index				
QNZ-PDB ID: 4CJN	0.5	0.6	0.7	0.8	0.9
MolPort	10,000	5999	1614	275	7
0Y5-PDB ID: 4GSY	0.5	0.6	0.7	0.8	0.9
MolPort	10,000	553	7	0	0

This table summarizes the number of compounds retrieved from the MolPort database based on structural similarity searches using the pivot compounds QNZ (PBP2a, PDB ID: 4CJN) and 0Y5 (TMK, PDB ID: 4GSY). Similarity was evaluated using Tanimoto indices ranging from 0.5 to 0.9. Higher Tanimoto values correspond to increased structural similarity to the pivot compounds, resulting in progressively fewer retrieved hits. This analysis illustrates the effect of similarity stringency on the size of the screened chemical space for each molecular target.

**Table 2:** Pharmacokinetic filter values obtained for template and commercial compounds.

Parameters	QNZ	0Y5	Methicillin	Oxacillin
Solubility ^1^	2	2	3	3
Blood-Cephalic Barrier ^2^	4	4	3	4
Cyp2d6 ^3^	False	False	False	False
Hepatotoxicity ^4^	False	True	False	False
Intestinal Absorption ^5^	0	0	0	0
Blood Plasma Protein Binding ^6^	True	False	False	True

^1^ Solubility: 0—extremely low, 1—very low but possible, 2—low; 3—good, 4—excellent, 5—very soluble and 6—out of the standards; ^2^ blood-cephalic barrier: 0—very high penetrance; 1—high penetrance; 2—average penetrance; 3—low penetrance and 4—indefinite. ^3^ CYP2D6: false—noninhibition of the CYP2D6 enzyme and true—inhibition of the CYP2D6 enzyme; ^4^ hepatotoxicity: false—for non-hepatotoxic compounds and true—for hepatotoxic compounds; ^5^ intestinal absorption: 0—good absorption; 1—moderate absorption; 2—low absorption and 3—very low absorption; ^6^ blood plasma protein binding: false—non-binding with plasma proteins and true—binding to blood plasma proteins.

**Table 3:** Toxicological properties of template and commercial compounds.

Parameters		Compounds			
		QNZ	0Y5	METHICILLIN	OXACILLIN
NTP Mouse	Female	Non-Carcinogenic	Carcinogenic	Non-Carcinogenic	Non-Carcinogenic
	Male	Non-Carcinogenic	Carcinogenic	Non-Carcinogenic	Non-Carcinogenic
NTP Rat	Female	Non-Carcinogenic	Non-Carcinogenic	Non-Carcinogenic	Non-Carcinogenic
	Male	Non-Carcinogenic	Non-Carcinogenic	Non-Carcinogenic	Non-Carcinogenic
Mouse FDA	Female	Non-Carcinogenic	Single Carcinogen	Non-Carcinogenic	Non-Carcinogenic
	Male	Single Carcinogen	Single Carcinogen	Non-Carcinogenic	Non-Carcinogenic
RAT FDA	Female	Non-Carcinogenic	Non-Carcinogenic	Non-Carcinogenic	Non-Carcinogenic
	Male	Single Carcinogen	Non-Carcinogenic	Non-Carcinogenic	Non-Carcinogenic
WOE		Carcinogenic	Non-Carcinogenic	Non-Carcinogenic	Non-Carcinogenic
AMES		Non-Mutagenic	Non-Mutagenic	Non-Mutagenic	Non-Mutagenic
DTP		Toxic	Non-Toxic	Non-Toxic	Non-Toxic

This table presents the predicted toxicological profiles of the pivot compounds QNZ and 0Y5 in comparison with the clinically used antibiotics methicillin and oxacillin. Toxicity endpoints were evaluated using in silico prediction models and include carcinogenicity assessments based on National Toxicology Program (NTP) mouse and rat models, U.S. Food and Drug Administration (FDA) mouse and rat models, weight-of-evidence (WOE) classification, AMES mutagenicity test prediction, and Developmental Toxicity Potential (DTP). The results are reported separately for male and female models when applicable. These predictions are intended to provide an early-stage safety overview and do not replace experimental toxicological validation.

**Table 4:** Interaction of the top five screened compounds and the pivot ligand with amino acid residues of the allosteric site of PBP2a from *S. aureus*.

Compounds	B.TYR105	B.ILE144	B.GLU145	B.ASN146	B.LYS273	B.GLU294	B.ASP295	B.TYR297	A.ILE309	B.LYS316	Interaction Hits	Binding Energy (Kcal/mol)
CAV01											5	−8.483
CAV02											4	−8.383
CAV03											5	−8.355
CAV04											4	−8.341
CAV05											5	−8.334
QNZ											5	−8.080
Types of chemical interactions												
Carbon Hydrogen; Pi-Cation; Pi-Pi T-Shaped; Pi-Alkyl; Conv. Hydrogen; Pi-Sigma; Amide-Pi Stacked												

This table presents a color-coded description of the molecular interactions established between the five main selected compounds (CAV01–CAV05) and the pivot compound QNZ with key amino acid residues of the allosteric site of penicillin-binding protein 2a (PBP2a). Each colored cell indicates the presence and type of interaction formed between a given compound and a specific residue during molecular docking analysis. The interaction types are represented by the following color scheme: carbon–hydrogen bond (light green), π-cation interaction (orange), T-shaped π–π interaction (pink), π-alkyl interaction (magenta), conventional hydrogen bond (dark green), π-sigma interaction (yellow), and stacked amide-π interaction (blue). The “Interactions Detected” column represents the total number of distinct residue interactions observed for each compound, while the “Binding Energy” column reports the corresponding docking affinity (kcal/mol). This interaction map provides a comparative overview of interaction patterns and highlights the similarities and differences between the analyzed compounds and the main ligand within the allosteric site of PBP2a.

**Table 5:** Interaction of the top five screened compounds and the pivot ligand with amino acid residues of the catalytic site of thymidylate kinase from *Staphylococcus aureus*.

Compounds	A.GLU11	A.GLU37	A.PRO38	A.GLY44	A.ARG48	A.VAL51	A.LEU52	A.LEU65	A.PHE66	A.SER69	A.ARG70	A.ARG92	A.SER96	A.SER97	A.TYR100	A.GLN101	A.ARG105	A.ASP156	Interaction Hits	Binding Energy (Kcal/mol)
GAV01																			7	−9.362
0Y5																			11	−9.147
GAV02																			8	−9.066
GAV03																			8	−8.891
GAV04																			9	−8.871
GAV05																			6	−8.857
Types of chemical interactions																				
Carbon Hydrogen; Pi-Pi T-Shaped; Pi-Alkyl; Conv. Hydrogen; Pi-Anion; Halogen (Fluorine)																				

This table presents the color-coded interaction, illustrating the molecular interactions formed between the five main selected compounds (GAV01–GAV05) and the pivot compound 0Y5 with key amino acid residues of the catalytic site of *Staphylococcus aureus* thymidylate kinase (TMK). Each colored cell denotes the presence and type of interaction between a given compound and a specific residue, as identified through molecular coupling analysis. The interaction types are represented by the following color scheme: carbon–hydrogen bond (light green), T-shaped π–π interaction (pink), π-alkyl interaction (magenta), conventional hydrogen bond (dark green), π-anion interaction (yellow), and halogen (fluorine) bond (blue). The “Interactions Detected” column indicates the total number of residue interactions detected for each compound, while the “Bonding Energy” column reports the corresponding coupling affinity values (kcal/mol). This interaction map allows for a direct comparison of interaction patterns, highlighting similarities and differences between the analyzed compounds and the pivot ligand within the TMK catalytic site.

**Table 6:** LD_50_ and toxicity class of the top 5 compounds with the best affinity energy via molecular docking.

Chemical Compounds		LD_50_ (mg/kg)	Toxicity Class
QNZ Template Screening	CAV01	982	4
	CAV02	677	4
	CAV03	883	4
	CAV04	883	4
	CAV05	883	4
0Y5 Template Screening	GAV01	1200	4
	GAV02	1200	4
	GAV03	1200	4
	GAV04	1000	4
	GAV05	1000	4
Reference and Commercial Compounds	QNZ	450	4
	0Y5	1000	4
	Methicillin	2880	5
	Oxacillin	6500	6

Class 1: fatal if swallowed (LD_50_ ≤ 5); Class 2: fatal if swallowed (5 < LD_50_ ≤ 50); Class 3: toxic if swallowed (50 < LD_50_ ≤ 300); Class 4: harmful if swallowed (300 < LD_50_ ≤ 2000); Class 5: may be harmful if swallowed (2000 < LD_50_ ≤ 5000); Class 6: non-toxic (LD_50_ > 5000).

**Table 7:** Lipinski’s parameters of top 5 compounds for each molecular target.

Compound	^1^ MM	^2^ LogP (o/w)	^3^ GDH	^4^ GAH
CAV01	484.59 g/mol	3.78	1	6
CAV02	484.57 g/mol	4.16	2	4
CAV03	462.56 g/mol	3.74	2	4
CAV04	446.56 g/mol	4.23	1	3
CAV05	460.59 g/mol	4.49	1	3
GAV01	483.56 g/mol	3.25	1	5
GAV02	483.56 g/mol	3.12	1	5
GAV03	483.56 g/mol	3.13	1	5
GAV04	446.48 g/mol	2.58	0	6
GAV05	402.45 g/mol	2.01	1	4
QNZ	393.39 g/mol	3.61	1	5
0Y5	503.47 g/mol	3.35	2	9
Methicillin	380.42 g/mol	1.13	2	6
Oxacillin	401.44 g/mol	1.85	2	6

MM ^1^ = molar mass; LogP ^2^ = water and octanol coefficient; GDH ^3^ = hydrogen donor group; GAH ^4^ = hydrogen acceptor group.

**Table 8:** In silico prediction of antibacterial activity for the top five screened compounds derived from QNZ using AntiBac-Pred.

Antibacterial Activity via Antibac-Pred			
Structure	Bacterial Strain	Confidence	Chembl ID
Methicillin	Resistant *S. aureus* subsp. *aureus* RN4220	0.8780	Chembl2366906
	*S. aureus*	0.3437	Chembl352
	*S. aureus* subsp. *aureus* RN4220	0.1901	Chembl2366906
	Resistant *S. aureus*	0.0318	Chembl352
Oxacillin	Resistant *S. aureus* subsp. *aureus* RN4220	0.9482	Chembl2366906
	*S. aureus*	0.3977	Chembl352
	*Staphylococcus* sp.	0.0406	Chembl612243
QNZ	*S. haemolyticus*	0.5909	Chembl612507
	*S. aureus*	0.0422	Chembl352
CAV01	*S. haemolyticus*	0.1212	Chembl612507
	*Staphylococcus* sp.	0.0875	Chembl612243
CAV02	*S. intermedius*	0.0907	Chembl614422
	*Staphylococcus* sp.	0.0759	Chembl612243
	Resistant *S. aureus* subsp. *aureus* MW2	0.0685	Chembl612531
CAV03	Resistant *S. simulans*	0.0923	Chembl612425
	Resistant *S. aureus* subsp. *aureus* MW2	0.0264	Chembl612531
	*S. sciuri*	0.0133	Chembl613150
CAV04	Resistant *S. aureus* subsp. *aureus* MW2	0.1025	Chembl612531
CAV05	Resistant *S. aureus* subsp. *aureus* MW2	0.1025	Chembl612531

This table summarizes the predicted antibacterial activity of the top five screened compounds derived from the pivot compound QNZ, as evaluated using the AntiBac-Pred platform. The predicted bacterial strains, confidence scores, and corresponding Chembl identifiers are reported. The confidence values represent probabilistic predictions generated by the model and are intended solely to support hypothesis generation and experimental prioritization rather than to indicate confirmed antibacterial activity.

**Table 9:** In silico prediction of antibacterial activity for the top five screened compounds derived from 0Y5 using AntiBac-Pred.

Antibacterial Activity via Antibac-Pred			
Structure	Bacterial Strain	Confidence	Chembl ID
Methicillin	Resistant *S. aureus* subsp. *aureus* RN4220	0.8780	Chembl2366906
	*S. aureus*	0.3437	Chembl352
	*S. aureus* subsp. *aureus* RN4220	0.1901	Chembl2366906
	Resistant *S. aureus*	0.0318	Chembl352
Oxacillin	Resistant *S. aureus* subsp. *aureus* RN4220	0.9482	Chembl2366906
	*S. aureus*	0.3977	Chembl352
	*Staphylococcus* sp.	0.0406	Chembl612243
0Y5	*S. lugdunensis*	0.8220	Chembl613303
	*S. haemolyticus*	0.2411	Chembl612507
	*S. aureus*	0.1733	Chembl352
	Resistant *S. aureus*	0.1450	Chembl352
	*Staphylococcus* sp.	0.1050	Chembl612243
	Resistant *S. aureus* subsp. *aureus* RN4220	0.0842	Chembl2366906
	*S. intermedius*	0.0312	Chembl614422
GAV01	Resistant *Propionibacterium acnes*	0.2622	Chembl612639
	Resistant *Bacteroides thetaiotaomicron*	0.0433	Chembl614412
	*Staphylococcus* sp.	0.0110	Chembl612243
GAV02	Resistant *P. acnes*	0.2715	Chembl612639
	Resistant *B. thetaiotaomicron*	0.0642	Chembl614412
	*Staphylococcus* sp.	0.0286	Chembl612243
GAV03	Resistant *P. acnes*	0.2715	Chembl612639
	Resistant *B. thetaiotaomicron*	0.0642	Chembl614412
	*S. aureus*	0.0140	Chembl352
	*Staphylococcus* sp.	0.0017	Chembl612243
GAV04	*S. lugdunensis*	0.1923	Chembl613303
	*Staphylococcus* sp.	0.1885	Chembl612243
	*Mycobacterium tuberculosis*	0.0159	Chembl360
	*M. tuberculosis* H37Rv	0.0127	Chembl2111188
	Resistant *M. tuberculosis*	0.0062	Chembl360
GAV05	*Staphylococcus* sp.	0.2875	Chembl612243
	*S. lugdunensis*	0.0807	Chembl613303

This table presents the predicted antibacterial activity of the top five screened compounds derived from the pivot compound 0Y5, as evaluated using the AntiBac-Pred platform. The predicted bacterial strains, confidence scores, and corresponding Chembl identifiers are reported. The confidence values reflect probabilistic model outputs and are intended to support hypothesis generation and experimental prioritization rather than to represent confirmed antibacterial efficacy.

**Table 10:** Theoretical accessibility of screened compounds.

Compound	Synthetic Accessibility Score	
	SwissADME ^(a)^	AMBIT-SA ^(b)^
CAV01	3.85	65.339
CAV02	3.40	63.051
CAV03	3.91	63.654
CAV04	3.36	64.925
CAV05	3.50	64.843
GAV01	3.59	64.724
GAV02	3.57	64.194
GAV03	3.60	59.187
GAV04	3.75	62.261
GAV05	3.48	61.200
QNZ	3.10	68.702
0Y5	3.95	60.403

^(a)^ SwissADME: 1 (very easy) to 10 (very difficult). ^(b)^ AMBIT-SA: score ≥ 50 (easy); 10 < score ≤ 49 (medium) and score ≤ 10 (difficult).

**Table 11:** Results of binding free energies (ΔGbind) and their components for the complexes formed by QNZ, CAV1, and CAV2.

Molecules	ΔEvdW	ΔEele	ΔGGB	ΔGNP	ΔGbinding
QNZ	−36.5	−12.8	22.1	−4.28	−31.48
CAV1	−31.45	−9.51	16.76	−2.9	−27.10
CAV2	−34.8	−11.2	20.05	−3.48	−29.43

**Table 12:** Results of binding free energies (ΔGbind) and their components for the complexes formed by 0Y5, GAV1, and GAV2.

Molecules	ΔEvdW	ΔEele	ΔGGB	ΔGNP	ΔGbinding
0Y5	−30.9	−9.8	17.1	−3.21	−26.81
GAV1	−29.6	−9.1	16	−2.62	−25.32
GAV2	−27.8	−8.4	15.4	−2.66	−23.46

**Table 13:** Molecular docking protocol settings used for PBP2a and TMK targets.

Receptor	Ligand	Grid Center Coordinates	Grid Box Dimensions
Penicillin-binding protein (PBP2a-MRSA) PDB ID: 4CJN	QNZ	x = 8.794	x = 10.000 Å
		y = −2.416	y = 16.838 Å
		z = −68.591	z = 11.817 Å
Thymidylate kinase enzyme (TMK) PDB ID: 4GSY	0Y5	x = 6.464	x = 15.000 Å
		y = 3.061	y = 16.000 Å
		z = 28.995	z = 15.000Å

This table summarizes the molecular docking parameters employed for the two selected targets, penicillin-binding protein 2a (PBP2a, PDB ID: 4CJN) and thymidylate kinase (TMK, PDB ID: 4GSY). The reference pivot ligands, grid center coordinates (x, y, z), and grid box dimensions (Å) used to define the docking search space are reported for each protein. These parameters were selected to adequately cover the respective binding sites during docking simulations.

## Data Availability

Data is contained within the article and Supplementary Materials.

## Supplementary Materials

The following supporting information can be downloaded at: https://www.mdpi.com/article/10.3390/ijms27062736/s1.
