# Supplementary material for: Identification of Potential Candidates with Antimicrobial Activity Against Antibiotic-Resistant Staphylococcus aureus Strains: A Hierarchical Bioinformatics Approach
Source: Int J Mol Sci. 2026 Mar 17;27(6):2736. doi: 10.3390/ijms27062736 (PMC13026990; doi:10.3390/ijms27062736)

International Journal of *Molecular Sciences*

MDPI

Identification of potential candidates with antimicrobial activity against antibiotic-resistant *Staphylococcus aureus* strains: a hierarchical Bioinformatics approach

Aderaldo Viegas da Silva [1], Kelton Luís Belém dos Santos [1,2], Lana Patrícia de Oliveira Barros Pinto de Oliveira [2], Luciana Sampaio Lima[2], Francy Mendes Nogueira Cardoso [1,2], Marcella Caroline Sampaio Vieira Carvalho [2], Ryan da Silva Ramos [2], Jorddy N. Cruz[2], Njogu Mark Kimani [3], Joaquín María Campos [4], Cleydson Breno Rodrigues dos Santos [1,2]*

1 Graduate Program in Biotechnology and Biodiversity-Network BIONORTE, Federal University of Amapá, Macapá 68903-419, Brazil. aderaldosilva14@gmail.com (A.V.d.S)
2 Laboratory of Modeling and Computational Chemistry, Department of Biological and Health Sciences, Federal University of Amapá, Macapá 68902-280, Brazil. lanaoliveira2013@gmail.com (L.P.d.O.B.P.d.O)
3 Department of Physical Sciences, University of Embu, Embu P.O. Box 6-60100, Kenya
4 Department of Pharmaceutical and Organic Chemistry, Faculty of Pharmacy, Institute of Biosanitary Research ibs. GRANADA, University of Granada, 18071 Granada, Spain; jmcampos@ugr.es

* **Correspondence: breno@unifap.br (C.B.R.S)**

Abstract

Antibiotic resistance among several bacteria is a warning sign that reinforces the need for research to identify new compounds effective against resistant strains. In this sense, Bioinformatics stands out as an excellent tool for identifying drug candidates by using computational methodologies to detect compounds with potential biological activity. Two pivot compounds (QNZ and 0Y5) with biological activity against *Staphylococcus aureus* were selected. A virtual screening was performed in the Molport database with a Tanimoto index of 0.5, resulting in 20,000 compounds, 10,000 compounds for each template. Then, methodologies were applied to calculate pharmacokinetic and toxicological parameters using Discovery Studio software; molecular docking via Dockthor; lethal dose via ProTOX; lipophilicity, solubility, and Lipinsky parameters via SwissADME; *in silico* prediction of bacterial activity via Way2Drug; theoretical synthetic accessibility via SwissADME and AMBIT-SA; and finally, molecular dynamics simulations via AMBER 18. After the entire methodological process, 10 compounds were identified with potential results according to the criteria adopted in this study and with possible antimicrobial activity against resistant bacterial strains of *S. aureus*. Our theoretical findings suggest 10 potential candidates with possible antimicrobial activity against *S. aureus* and other genus and species of bacteria, as these compounds presented excellent results using the proposed methodology. Certainly, more *in vitro* and *in vivo* study steps are necessary.

Keywords: Molecular docking, Virtual screening, Antimicrobial resistance, *Staphylococcus aureus*, Computational drug discovery

Supplementary Material

Table S1: List of all compounds selected in the pharmacokinetic step from QNZ

COMPOUNDS	PARAMETERS					
	SOLUBILITY	BLOOD-CEPHALIC BARRIER	CYP2D6	HEPATOTOXICITY	INTESTINAL ABSORPTION	BLOOD PLASMA PROTEIN BINDING
MolPort-002-558-192	2	1	false	false	0	true
MolPort-000-720-419	2	1	false	false	0	true
MolPort-002-870-436	2	1	false	false	0	true
MolPort-000-163-155	2	1	false	false	0	true
MolPort-003-183-873	2	1	false	false	0	true
MolPort-001-954-672	2	1	false	false	0	true
MolPort-002-007-476	2	1	false	false	0	true
MolPort-002-180-646	2	1	false	false	0	true
MolPort-002-551-995	2	1	false	false	0	true
MolPort-001-510-405	2	2	false	false	0	true
MolPort-001-979-695	2	2	false	false	0	true
MolPort-001-532-007	2	1	false	false	0	true
MolPort-007-920-205	2	1	false	false	0	true
MolPort-002-589-813	2	1	false	false	0	true
MolPort-000-714-337	2	1	false	false	0	true
MolPort-001-915-783	2	1	false	false	0	true
MolPort-001-532-008	2	1	false	false	0	true
MolPort-002-546-645	2	1	false	false	0	true
MolPort-001-496-238	2	1	false	false	0	true
MolPort-001-896-800	2	1	false	false	0	true
MolPort-002-628-237	2	1	false	false	0	true
MolPort-001-954-692	2	1	false	false	0	true
MolPort-000-773-166	2	1	false	false	0	true
MolPort-000-825-045	2	1	false	false	0	true
MolPort-001-525-277	2	1	false	false	0	true
MolPort-001-496-325	2	2	false	false	0	true
MolPort-002-547-544	2	2	false	false	0	true
MolPort-002-870-434	2	1	false	false	0	true
MolPort-007-920-119	2	1	false	false	0	true
MolPort-001-508-852	2	1	false	false	0	true
MolPort-002-621-023	2	1	false	false	0	true
MolPort-007-920-017	2	1	false	false	0	true
MolPort-002-536-784	2	1	false	false	0	true
MolPort-001-965-773	2	2	false	false	0	true
MolPort-001-954-675	2	1	false	false	0	true

COMPOUNDS	PARAMETERS					
	SOLUBILITY	BLOOD-CEPHALIC BARRIER	CYP2D6	HEPATOTOXICITY	INTESTINAL ABSORPTION	BLOOD PLASMA PROTEIN BINDING
MolPort-002-609-250	2	1	false	false	0	true
MolPort-001-959-871	2	1	false	false	0	true
MolPort-001-959-867	2	1	false	false	0	true
MolPort-001-532-006	2	1	false	false	0	true
MolPort-002-574-982	2	1	false	false	0	true
MolPort-001-965-774	2	1	false	false	0	true
MolPort-001-495-138	2	1	false	false	0	true
MolPort-002-118-352	2	1	false	false	0	true
MolPort-002-206-746	2	1	false	false	0	true
MolPort-002-589-492	2	1	false	false	0	true
MolPort-002-101-060	2	1	false	false	0	true
MolPort-001-959-881	2	1	false	false	0	true
MolPort-007-919-958	2	1	false	false	0	true
MolPort-007-920-002	2	1	false	false	0	true
MolPort-002-169-312	2	1	false	false	0	true
MolPort-002-007-475	2	1	false	false	0	true
MolPort-001-508-865	2	1	false	false	0	true
MolPort-001-508-851	2	1	false	false	0	true
MolPort-007-920-116	2	1	false	false	0	true
MolPort-002-007-477	2	1	false	false	0	true
MolPort-039-019-470	2	1	false	false	0	true
MolPort-007-920-267	2	1	false	false	0	true
MolPort-001-959-879	2	1	false	false	0	true
MolPort-007-555-957	2	1	false	false	0	true
MolPort-002-007-464	2	1	false	false	0	true
MolPort-002-323-575	2	1	false	false	0	true
MolPort-002-180-651	2	1	false	false	0	true
MolPort-007-920-273	2	1	false	false	0	true
MolPort-002-118-932	2	0	false	false	0	true
MolPort-002-548-479	2	1	false	false	0	true
MolPort-002-007-480	2	1	false	false	0	true
MolPort-000-848-625	2	1	false	false	0	true
MolPort-000-728-326	2	1	false	false	0	true
MolPort-002-085-489	2	1	false	false	0	true
MolPort-000-852-679	2	1	false	false	0	true
MolPort-001-961-882	2	1	false	false	0	true

COMPOUNDS	PARAMETERS					
	SOLUBILITY	BLOOD-CEPHALIC BARRIER	CYP2D6	HEPATOTOXICITY	INTESTINAL ABSORPTION	BLOOD PLASMA PROTEIN BINDING
MolPort-001-965-777	2	1	false	false	0	true
MolPort-002-629-556	2	1	false	false	0	true
MolPort-000-699-823	2	1	false	false	0	true
MolPort-000-744-347	2	1	false	false	0	true
MolPort-000-788-016	2	1	false	false	0	true
MolPort-002-559-360	3	2	false	false	0	true
MolPort-000-918-134	2	2	false	false	0	true
MolPort-000-901-854	2	1	false	false	0	true
MolPort-002-004-020	2	1	false	false	0	true
MolPort-002-001-167	2	1	false	false	0	true
MolPort-002-709-662	2	1	false	false	0	true
MolPort-001-848-862	2	1	false	false	0	true
MolPort-010-785-755	2	1	false	false	0	true
MolPort-003-378-239	2	2	false	false	0	true
MolPort-000-829-777	2	1	false	false	0	true
MolPort-000-837-815	2	1	false	false	0	true
MolPort-007-975-257	2	1	false	false	0	true
MolPort-010-693-336	2	1	false	false	0	true
MolPort-003-145-972	2	1	false	false	0	true
MolPort-002-207-256	2	0	false	false	0	true
MolPort-001-496-066	2	1	false	false	0	true
MolPort-005-930-626	2	2	false	false	0	true
MolPort-044-636-245	2	2	false	false	0	true
MolPort-047-584-956	2	1	false	false	0	true
MolPort-003-146-028	2	1	false	false	0	true
MolPort-000-827-808	2	0	false	false	0	true
MolPort-001-891-972	2	1	false	false	0	true
MolPort-003-145-994	2	1	false	false	0	true
MolPort-010-785-744	2	1	false	false	0	true
MolPort-000-914-692	2	2	false	false	0	true
MolPort-005-926-713	2	1	false	false	0	true
MolPort-003-145-991	2	1	false	false	0	true
MolPort-010-785-757	2	1	false	false	0	true
MolPort-000-848-982	2	1	false	false	0	true
MolPort-000-914-546	2	1	false	false	0	true
MolPort-003-378-231	2	1	false	false	0	true

COMPOUNDS	PARAMETERS					
	SOLUBILITY	BLOOD-CEPHALIC BARRIER	CYP2D6	HEPATOTOXICITY	INTESTINAL ABSORPTION	BLOOD PLASMA PROTEIN BINDING
MolPort-008-430-067	2	2	false	false	0	true
MolPort-010-693-332	2	1	false	false	0	true
MolPort-002-571-906	2	1	false	false	0	true
MolPort-002-601-462	2	0	false	false	0	true
MolPort-003-146-003	2	1	false	false	0	true
MolPort-003-145-976	2	1	false	false	0	true
MolPort-002-578-419	2	1	false	false	0	true
MolPort-000-220-846	2	1	false	false	0	true
MolPort-000-833-148	2	0	false	false	0	true
MolPort-003-146-018	2	1	false	false	0	true
MolPort-000-736-044	2	0	false	false	0	true
MolPort-007-975-276	2	1	false	false	0	true
MolPort-002-001-477	2	1	false	false	0	true
MolPort-003-145-921	2	1	false	false	0	true
MolPort-009-736-599	2	1	false	false	0	true
MolPort-006-810-336	2	2	false	false	0	true
MolPort-005-976-468	2	2	false	false	0	true
MolPort-003-378-236	2	1	false	false	0	true
MolPort-002-164-221	2	2	false	false	0	true
MolPort-005-980-064	3	2	false	false	0	true
MolPort-001-633-020	2	1	false	false	0	true
MolPort-002-321-066	2	1	false	false	0	true
MolPort-010-785-756	2	1	false	false	0	true
MolPort-000-743-391	2	0	false	false	0	true
MolPort-001-532-277	2	1	false	false	0	true
MolPort-002-571-014	2	1	false	false	0	true
MolPort-003-145-920	2	1	false	false	0	true
MolPort-000-836-068	2	1	false	false	0	true
MolPort-010-785-753	2	1	false	false	0	true
MolPort-000-918-432	2	1	false	false	0	true
MolPort-003-157-184	2	1	false	false	0	true
MolPort-008-314-299	2	2	false	false	0	true
MolPort-008-313-898	2	2	false	false	0	true
MolPort-003-145-992	2	2	false	false	0	true
MolPort-003-145-971	2	1	false	false	0	true
MolPort-002-511-359	2	1	false	false	0	true

COMPOUNDS	PARAMETERS					
	SOLUBILITY	BLOOD-CEPHALIC BARRIER	CYP2D6	HEPATOTOXICITY	INTESTINAL ABSORPTION	BLOOD PLASMA PROTEIN BINDING
MolPort-004-954-334	2	1	false	false	0	true
MolPort-005-969-844	2	2	false	false	0	true
MolPort-002-248-203	2	1	false	false	0	true
MolPort-002-165-886	2	2	false	false	0	true
MolPort-002-083-854	2	1	false	false	0	true
MolPort-019-870-074	2	1	false	false	0	true
MolPort-004-843-552	2	1	false	false	0	true
MolPort-003-145-999	2	1	false	false	0	true
MolPort-002-545-055	2	2	false	false	0	true
MolPort-001-532-276	2	1	false	false	0	true
MolPort-005-919-095	2	2	false	false	0	true
MolPort-002-666-277	2	1	false	false	0	true
MolPort-009-736-639	2	1	false	false	0	true
MolPort-000-918-408	2	1	false	false	0	true
MolPort-001-979-697	2	1	false	false	0	true
MolPort-001-509-028	2	1	false	false	0	true
MolPort-004-843-553	2	1	false	false	0	true
MolPort-000-614-768	2	1	false	false	0	true
MolPort-000-735-987	2	0	false	false	0	true
MolPort-007-975-293	2	2	false	false	0	true
MolPort-007-975-296	2	2	false	false	0	true
MolPort-003-157-228	2	1	false	false	0	true
MolPort-046-425-730	2	2	false	false	0	true
MolPort-001-027-281	2	1	false	false	0	true
MolPort-003-145-924	2	1	false	false	0	true
MolPort-002-801-957	2	1	false	false	0	true
MolPort-000-858-693	2	1	false	false	0	true
MolPort-001-633-725	2	1	false	false	0	true
MolPort-019-931-888	2	1	false	false	0	true
MolPort-001-650-474	2	1	false	false	0	true
MolPort-000-717-569	2	1	false	false	0	true
MolPort-002-288-462	2	1	false	false	0	true
MolPort-001-953-531	2	1	false	false	0	true
MolPort-002-094-226	2	1	false	false	0	true
MolPort-002-665-311	2	1	false	false	0	true
MolPort-001-965-869	2	1	false	false	0	true

COMPOUNDS	PARAMETERS					
	SOLUBILITY	BLOOD-CEPHALIC BARRIER	CYP2D6	HEPATOTOXICITY	INTESTINAL ABSORPTION	BLOOD PLASMA PROTEIN BINDING
MolPort-002-273-873	2	1	false	false	0	true
MolPort-000-711-767	2	1	false	false	0	true
MolPort-000-051-566	2	1	false	false	0	true
MolPort-003-145-923	2	1	false	false	0	true
MolPort-002-801-956	2	2	false	false	0	true
MolPort-000-733-030	2	1	false	false	0	true
MolPort-001-965-870	2	1	false	false	0	true
MolPort-004-843-547	2	1	false	false	0	true
MolPort-000-740-406	3	2	false	false	0	true
MolPort-006-811-446	2	2	false	false	0	true
MolPort-046-685-647	2	1	false	false	0	true
MolPort-002-665-556	2	2	false	false	0	true
MolPort-002-666-947	2	2	false	false	0	true
MolPort-000-051-570	2	1	false	false	0	true
MolPort-047-584-980	2	1	false	false	0	true
MolPort-016-899-843	2	1	false	false	0	true
MolPort-001-494-053	2	2	false	false	0	true
MolPort-001-913-400	2	1	false	false	0	true
MolPort-000-708-378	2	1	false	false	0	true
MolPort-001-911-947	2	1	false	false	0	true
MolPort-002-620-454	2	1	false	false	0	true
MolPort-002-624-244	2	1	false	false	0	true
MolPort-008-333-219	2	1	false	false	0	true
MolPort-009-736-608	2	2	false	false	0	true
MolPort-004-843-563	2	1	false	false	0	true
MolPort-002-842-374	2	1	false	false	0	true
MolPort-051-578-906	2	2	false	false	0	true
MolPort-001-983-441	2	1	false	false	0	true
MolPort-002-003-292	2	1	false	false	0	true
MolPort-008-342-958	2	2	false	false	0	true
MolPort-001-996-158	2	1	false	false	0	true
MolPort-002-668-721	2	2	false	false	0	true
MolPort-008-342-790	2	2	false	false	0	true
MolPort-007-579-353	2	1	false	false	0	true
MolPort-003-146-175	2	2	false	false	0	true
MolPort-008-344-861	2	1	false	false	0	true

COMPOUNDS	PARAMETERS					
	SOLUBILITY	BLOOD-CEPHALIC BARRIER	CYP2D6	HEPATOTOXICITY	INTESTINAL ABSORPTION	BLOOD PLASMA PROTEIN BINDING
MolPort-002-512-634	3	2	false	false	0	true
MolPort-002-014-881	2	2	false	false	0	true
MolPort-000-845-119	3	2	false	false	0	true
MolPort-001-939-889	2	1	false	false	0	true
MolPort-008-344-505	2	2	false	false	0	true
MolPort-001-903-023	2	1	false	false	0	true
MolPort-008-339-617	2	2	false	false	0	true
MolPort-002-579-834	2	1	false	false	0	true
MolPort-010-799-254	2	1	false	false	0	true
MolPort-002-589-840	2	2	false	false	0	true
MolPort-005-911-135	2	2	false	false	0	true
MolPort-008-342-117	2	2	false	false	0	true
MolPort-005-972-868	2	2	false	false	0	true
MolPort-001-966-733	2	1	false	false	0	true
MolPort-001-910-024	3	2	false	false	0	true
MolPort-001-496-362	2	1	false	false	0	true
MolPort-001-965-875	2	1	false	false	0	true
MolPort-010-799-253	2	1	false	false	0	true
MolPort-002-086-428	3	2	false	false	0	true
MolPort-004-843-565	2	1	false	false	0	true
MolPort-000-051-571	2	1	false	false	0	true
MolPort-002-272-211	2	2	false	false	0	true
MolPort-010-961-340	2	2	false	false	0	true
MolPort-000-715-666	2	1	false	false	0	true
MolPort-046-418-807	2	1	false	false	0	true
MolPort-002-299-028	2	1	false	false	0	true
MolPort-002-274-066	2	1	false	false	0	true
MolPort-001-987-421	2	1	false	false	0	true
MolPort-002-568-297	2	1	false	false	0	true
MolPort-002-556-215	2	0	false	false	0	true
MolPort-002-274-818	2	2	false	false	0	true
MolPort-001-954-654	2	1	false	false	0	true
MolPort-002-275-050	2	0	false	false	0	true
MolPort-001-903-770	2	2	false	false	0	true
MolPort-002-917-210	2	1	false	false	0	true
MolPort-016-900-298	2	1	false	false	0	true

COMPOUNDS	PARAMETERS					
	SOLUBILITY	BLOOD-CEPHALIC BARRIER	CYP2D6	HEPATOTOXICITY	INTESTINAL ABSORPTION	BLOOD PLASMA PROTEIN BINDING
MolPort-002-085-159	2	1	false	false	0	true
MolPort-001-890-755	3	2	false	false	0	true
MolPort-000-727-887	2	2	false	false	0	true
MolPort-003-146-173	2	2	false	false	0	true
MolPort-004-843-566	2	1	false	false	0	true
MolPort-001-496-363	2	1	false	false	0	true
MolPort-002-559-455	2	1	false	false	0	true
MolPort-010-961-343	2	2	false	false	0	true
MolPort-001-996-160	2	1	false	false	0	true
MolPort-006-505-380	2	2	false	false	0	true
MolPort-002-090-886	3	2	false	false	0	true
MolPort-000-743-235	2	1	false	false	0	true
MolPort-010-961-306	2	1	false	false	0	true
MolPort-002-581-442	2	0	false	false	0	true
MolPort-002-665-396	2	1	false	false	0	true
MolPort-010-961-339	2	1	false	false	0	true
MolPort-002-123-410	2	1	false	false	0	true
MolPort-002-547-850	2	1	false	false	0	true
MolPort-001-908-029	2	1	false	false	0	true
MolPort-003-146-172	2	2	false	false	0	true
MolPort-000-839-745	2	1	false	false	0	true
MolPort-001-576-890	2	0	false	false	0	true
MolPort-016-910-415	2	2	false	false	0	true
MolPort-047-917-180	2	2	false	false	0	true
MolPort-051-552-482	2	2	false	false	0	true
MolPort-002-274-146	2	2	false	false	0	true
MolPort-007-788-818	2	2	false	false	0	true
MolPort-007-909-672	2	2	false	false	0	true
MolPort-008-344-602	2	2	false	false	0	true
MolPort-010-661-933	2	1	false	false	0	true
MolPort-001-509-029	2	1	false	false	0	true
MolPort-002-169-310	2	1	false	false	0	true
MolPort-000-703-827	2	1	false	false	0	true
MolPort-000-700-352	2	1	false	false	0	true
MolPort-001-958-478	2	0	false	false	0	true
MolPort-010-961-334	2	2	false	false	0	true

COMPOUNDS	PARAMETERS					
	SOLUBILITY	BLOOD-CEPHALIC BARRIER	CYP2D6	HEPATOTOXICITY	INTESTINAL ABSORPTION	BLOOD PLASMA PROTEIN BINDING
MolPort-010-661-935	2	1	false	false	0	true
MolPort-002-021-656	2	1	false	false	0	true
MolPort-007-915-803	2	1	false	false	0	true
MolPort-001-959-866	2	1	false	false	0	true
MolPort-001-958-474	2	1	false	false	0	true
MolPort-016-910-276	2	2	false	false	0	true
MolPort-010-661-929	2	1	false	false	0	true
MolPort-001-991-949	3	2	false	false	0	true
MolPort-010-796-861	2	2	false	false	0	true
MolPort-001-964-357	2	2	false	false	0	true
MolPort-002-214-591	3	1	false	false	0	true
MolPort-010-961-300	2	1	false	false	0	true
MolPort-010-661-930	2	1	false	false	0	true
MolPort-010-961-330	2	1	false	false	0	true
MolPort-001-987-422	2	1	false	false	0	true
MolPort-002-568-512	2	1	false	false	0	true
MolPort-007-608-380	2	2	false	false	0	true
MolPort-010-661-932	2	1	false	false	0	true
MolPort-002-635-550	2	1	false	false	0	true
MolPort-000-700-766	2	1	false	false	0	true
MolPort-006-037-677	2	1	false	false	0	true
MolPort-001-849-093	2	1	false	false	0	true
MolPort-000-830-872	2	1	false	false	0	true
MolPort-001-944-355	2	0	false	false	0	true
MolPort-001-997-751	2	1	false	false	0	true
MolPort-010-661-936	2	1	false	false	0	true
MolPort-002-541-996	2	1	false	false	0	true
MolPort-046-426-686	2	1	false	false	0	true
MolPort-002-296-939	2	1	false	false	0	true
MolPort-007-579-483	2	1	false	false	0	true
MolPort-000-829-844	2	1	false	false	0	true
MolPort-001-991-948	2	1	false	false	0	true
MolPort-010-961-333	2	1	false	false	0	true
MolPort-000-739-421	2	1	false	false	0	true
MolPort-016-910-263	2	2	false	false	0	true
MolPort-009-713-376	2	1	false	false	0	true

COMPOUNDS	PARAMETERS					
	SOLUBILITY	BLOOD-CEPHALIC BARRIER	CYP2D6	HEPATOTOXICITY	INTESTINAL ABSORPTION	BLOOD PLASMA PROTEIN BINDING
MolPort-002-286-502	2	1	false	false	0	true
MolPort-007-903-342	2	2	false	false	0	true
MolPort-010-961-301	2	1	false	false	0	true
MolPort-010-961-325	2	1	false	false	0	true
MolPort-000-828-199	2	1	false	false	0	true
MolPort-001-994-711	2	1	false	false	0	true
MolPort-009-713-356	2	1	false	false	0	true
MolPort-002-587-461	2	1	false	false	0	true
MolPort-008-338-409	2	2	false	false	0	true
MolPort-002-016-205	2	1	false	false	0	true
MolPort-009-713-357	2	1	false	false	0	true
MolPort-002-660-993	2	2	false	false	0	true
MolPort-002-284-368	2	1	false	false	0	true
MolPort-047-898-129	2	2	false	false	0	true
MolPort-000-827-812	2	1	false	false	0	true
MolPort-007-915-467	2	1	false	false	0	true
MolPort-001-994-767	3	2	false	false	0	true
MolPort-010-961-267	2	1	false	false	0	true
MolPort-016-910-264	2	2	false	false	0	true
MolPort-004-832-102	2	1	false	false	0	true
MolPort-003-029-485	2	2	false	false	0	true
MolPort-001-991-950	2	1	false	false	0	true
MolPort-001-944-340	2	0	false	false	0	true
MolPort-001-905-481	2	1	false	false	0	true
MolPort-003-965-540	2	1	false	false	0	true
MolPort-007-598-724	2	2	false	false	0	true
MolPort-002-017-868	2	1	false	false	0	true
MolPort-004-832-105	2	1	false	false	0	true
MolPort-001-616-095	2	1	false	false	0	true
MolPort-009-713-353	2	2	false	false	0	true
MolPort-003-107-534	2	2	false	false	0	true
MolPort-010-961-328	2	1	false	false	0	true
MolPort-008-334-857	2	1	false	false	0	true
MolPort-001-994-712	2	1	false	false	0	true
MolPort-000-736-082	2	1	false	false	0	true
MolPort-008-335-634	2	2	false	false	0	true

COMPOUNDS	PARAMETERS					
	SOLUBILITY	BLOOD-CEPHALIC BARRIER	CYP2D6	HEPATOTOXICITY	INTESTINAL ABSORPTION	BLOOD PLASMA PROTEIN BINDING
MolPort-002-296-975	2	1	false	false	0	true
MolPort-010-661-872	2	1	false	false	0	true
MolPort-010-661-873	2	1	false	false	0	true
MolPort-010-961-329	2	1	false	false	0	true
MolPort-001-682-952	2	2	false	false	0	true
MolPort-010-661-865	2	1	false	false	0	true
MolPort-010-661-927	2	2	false	false	0	true
MolPort-007-579-520	2	1	false	false	0	true
MolPort-003-029-486	2	1	false	false	0	true
MolPort-010-661-867	2	1	false	false	0	true
MolPort-010-961-319	2	1	false	false	0	true
MolPort-002-180-644	2	1	false	false	0	true
MolPort-002-590-294	2	1	false	false	0	true
MolPort-001-997-748	2	1	false	false	0	true
MolPort-019-716-570	2	1	false	false	0	true
MolPort-003-029-079	2	1	false	false	0	true
MolPort-003-591-751	2	2	false	false	0	true
MolPort-004-950-004	2	1	false	false	0	true
MolPort-010-961-358	2	1	false	false	0	true
MolPort-010-661-866	2	2	false	false	0	true
MolPort-007-675-172	2	1	false	false	0	true
MolPort-002-283-274	2	0	false	false	0	true
MolPort-002-088-505	2	1	false	false	0	true
MolPort-002-578-447	2	1	false	false	0	true
MolPort-006-318-220	2	1	false	false	0	true
MolPort-010-661-874	2	1	false	false	0	true
MolPort-010-661-911	2	1	false	false	0	true
MolPort-008-316-249	2	2	false	false	0	true
MolPort-003-965-528	2	1	false	false	0	true
MolPort-007-598-725	2	2	false	false	0	true
MolPort-007-675-446	2	1	false	false	0	true
MolPort-010-961-347	2	1	false	false	0	true
MolPort-002-662-127	2	1	false	false	0	true
MolPort-004-892-984	2	1	false	false	0	true
MolPort-001-536-634	2	2	false	false	0	true
MolPort-002-508-339	2	1	false	false	0	true

COMPOUNDS	PARAMETERS					
	SOLUBILITY	BLOOD-CEPHALIC BARRIER	CYP2D6	HEPATOTOXICITY	INTESTINAL ABSORPTION	BLOOD PLASMA PROTEIN BINDING
MolPort-009-766-872	2	0	false	false	0	true
MolPort-007-913-267	2	1	false	false	0	true
MolPort-002-510-828	2	2	false	false	0	true
MolPort-002-123-700	2	1	false	false	0	true
MolPort-008-300-237	2	1	false	false	0	true
MolPort-010-961-360	2	1	false	false	0	true
MolPort-000-853-802	2	1	false	false	0	true
MolPort-003-029-080	2	1	false	false	0	true
MolPort-002-238-872	2	0	false	false	0	true
MolPort-003-029-498	2	1	false	false	0	true
MolPort-009-713-335	2	2	false	false	0	true
MolPort-010-961-349	2	1	false	false	0	true
MolPort-000-705-655	2	2	false	false	0	true
MolPort-002-510-933	2	1	false	false	0	true
MolPort-004-832-103	2	1	false	false	0	true
MolPort-000-837-477	2	1	false	false	0	true
MolPort-010-961-352	2	1	false	false	0	true
MolPort-010-961-354	2	2	false	false	0	true
MolPort-002-097-491	2	2	false	false	0	true
MolPort-007-675-198	2	1	false	false	0	true
MolPort-001-975-233	2	1	false	false	0	true
MolPort-010-961-314	2	2	false	false	0	true
MolPort-010-961-361	2	1	false	false	0	true
MolPort-003-029-082	2	1	false	false	0	true
MolPort-000-714-703	2	1	false	false	0	true
MolPort-001-844-431	2	2	false	false	0	true
MolPort-046-868-290	2	2	false	false	0	true
MolPort-010-961-262	2	2	false	false	0	true
MolPort-000-734-010	2	1	false	false	0	true
MolPort-008-328-249	2	2	false	false	0	true
MolPort-009-713-358	2	1	false	false	0	true
MolPort-000-657-398	2	1	false	false	0	true
MolPort-010-661-877	2	2	false	false	0	true
MolPort-000-035-396	2	2	false	false	0	true
MolPort-001-997-749	2	1	false	false	0	true
MolPort-009-710-101	2	2	false	false	0	true

COMPOUNDS	PARAMETERS					
	SOLUBILITY	BLOOD-CEPHALIC BARRIER	CYP2D6	HEPATOTOXICITY	INTESTINAL ABSORPTION	BLOOD PLASMA PROTEIN BINDING
MolPort-010-961-271	2	1	false	false	0	true
MolPort-000-047-065	2	1	false	false	0	true
MolPort-003-029-081	2	2	false	false	0	true
MolPort-008-314-885	2	2	false	false	0	true
MolPort-002-029-886	2	2	false	false	0	true
MolPort-003-029-497	2	1	false	false	0	true
MolPort-000-444-067	2	1	false	false	0	true
MolPort-000-839-149	2	1	false	false	0	true
MolPort-002-510-826	2	1	false	false	0	true
MolPort-003-029-416	2	1	false	false	0	true
MolPort-007-888-983	2	2	false	false	0	true
MolPort-009-701-491	2	2	false	false	0	true
MolPort-010-961-258	2	1	false	false	0	true
MolPort-000-635-510	2	1	false	false	0	true
MolPort-000-051-577	2	1	false	false	0	true
MolPort-009-713-368	2	1	false	false	0	true
MolPort-007-675-181	2	1	false	false	0	true
MolPort-016-910-006	2	2	false	false	0	true
MolPort-002-005-903	3	2	false	false	0	true
MolPort-000-847-632	2	1	false	false	0	true
MolPort-000-035-384	2	1	false	false	0	true
MolPort-002-276-151	2	1	false	false	0	true
MolPort-010-806-836	2	1	false	false	0	true
MolPort-003-029-355	2	2	false	false	0	true
MolPort-007-675-436	2	1	false	false	0	true
MolPort-005-917-410	2	2	false	false	0	true
MolPort-002-158-064	2	1	false	false	0	true
MolPort-002-508-835	2	2	false	false	0	true
MolPort-007-675-439	2	1	false	false	0	true
MolPort-003-872-842	2	2	false	false	0	true
MolPort-001-994-765	2	1	false	false	0	true
MolPort-009-713-329	2	1	false	false	0	true
MolPort-002-296-958	2	2	false	false	0	true
MolPort-005-917-695	2	2	false	false	0	true
MolPort-000-035-380	2	1	false	false	0	true
MolPort-010-961-263	2	2	false	false	0	true

COMPOUNDS	PARAMETERS					
	SOLUBILITY	BLOOD-CEPHALIC BARRIER	CYP2D6	HEPATOTOXICITY	INTESTINAL ABSORPTION	BLOOD PLASMA PROTEIN BINDING
MolPort-000-635-515	2	1	false	false	0	true
MolPort-009-713-327	2	2	false	false	0	true
MolPort-010-961-256	2	1	false	false	0	true
MolPort-002-592-406	2	1	false	false	0	true
MolPort-023-300-288	2	1	false	false	0	true
MolPort-004-892-994	2	1	false	false	0	true
MolPort-002-661-167	2	1	false	false	0	true
MolPort-004-892-989	2	2	false	false	0	true
MolPort-002-534-290	2	2	false	false	0	true
MolPort-003-060-212	2	2	false	false	0	true
MolPort-046-842-331	2	1	false	false	0	true
MolPort-007-635-930	2	2	false	false	0	true
MolPort-010-796-862	2	1	false	false	0	true
MolPort-004-000-769	2	1	false	false	0	true
MolPort-005-925-188	2	2	false	false	0	true
MolPort-000-087-187	2	1	false	false	0	true
MolPort-002-665-802	2	2	false	false	0	true
MolPort-009-713-323	2	2	false	false	0	true
MolPort-002-534-180	2	2	false	false	0	true
MolPort-009-713-350	2	2	false	false	0	true
MolPort-007-784-038	2	2	false	false	0	true
MolPort-006-816-719	2	2	false	false	0	true
MolPort-003-029-493	2	2	false	false	0	true
MolPort-000-819-385	2	1	false	false	0	true
MolPort-007-595-219	2	2	false	false	0	true
MolPort-010-961-259	2	1	false	false	0	true
MolPort-010-661-898	2	1	false	false	0	true
MolPort-010-661-871	2	1	false	false	0	true
MolPort-003-029-106	2	2	false	false	0	true
MolPort-010-806-830	2	1	false	false	0	true
MolPort-010-961-251	2	1	false	false	0	true
MolPort-007-677-228	2	2	false	false	0	true
MolPort-007-675-437	2	1	false	false	0	true
MolPort-005-921-169	2	1	false	false	0	true
MolPort-007-608-233	2	1	false	false	0	true
MolPort-010-806-841	2	2	false	false	0	true

COMPOUNDS	PARAMETERS					
	SOLUBILITY	BLOOD-CEPHALIC BARRIER	CYP2D6	HEPATOTOXICITY	INTESTINAL ABSORPTION	BLOOD PLASMA PROTEIN BINDING
MolPort-001-857-227	2	1	false	false	0	true
MolPort-010-661-897	2	1	false	false	0	true
MolPort-009-710-097	2	2	false	false	0	true
MolPort-002-534-229	3	2	false	false	0	true
MolPort-004-892-990	3	2	false	false	0	true
MolPort-007-675-434	2	1	false	false	0	true
MolPort-003-010-674	2	1	false	false	0	true
MolPort-010-961-250	2	2	false	false	0	true
MolPort-010-661-945	2	1	false	false	0	true
MolPort-010-806-849	2	2	false	false	0	true
MolPort-007-675-501	2	1	false	false	0	true
MolPort-010-661-895	2	2	false	false	0	true
MolPort-000-820-027	2	1	false	false	0	true
MolPort-044-218-403	2	1	false	false	0	true
MolPort-005-918-368	2	2	false	false	0	true
MolPort-007-675-424	2	2	false	false	0	true
MolPort-003-029-230	2	2	false	false	0	true
MolPort-008-325-339	2	2	false	false	0	true
MolPort-009-713-367	2	1	false	false	0	true
MolPort-010-806-835	2	1	false	false	0	true
MolPort-008-325-428	2	2	false	false	0	true
MolPort-000-635-508	2	1	false	false	0	true
MolPort-002-314-621	2	2	false	false	0	true
MolPort-000-087-139	2	2	false	false	0	true
MolPort-010-661-892	2	1	false	false	0	true
MolPort-010-961-257	2	1	false	false	0	true
MolPort-009-713-337	2	2	false	false	0	true
MolPort-007-675-425	2	1	false	false	0	true
MolPort-010-661-940	2	1	false	false	0	true
MolPort-010-961-376	2	1	false	false	0	true
MolPort-010-661-891	2	1	false	false	0	true
MolPort-009-766-971	2	0	false	false	0	true
MolPort-010-961-346	2	2	false	false	0	true
MolPort-002-512-852	2	1	false	false	0	true
MolPort-000-641-822	2	2	false	false	0	true
MolPort-010-661-894	2	1	false	false	0	true

COMPOUNDS	PARAMETERS					
	SOLUBILITY	BLOOD-CEPHALIC BARRIER	CYP2D6	HEPATOTOXICITY	INTESTINAL ABSORPTION	BLOOD PLASMA PROTEIN BINDING
MolPort-002-272-187	2	1	false	false	0	true
MolPort-003-029-502	2	2	false	false	0	true
MolPort-007-675-445	2	1	false	false	0	true
MolPort-007-675-447	2	1	false	false	0	true
MolPort-007-909-700	2	1	false	false	0	true
MolPort-008-325-405	2	2	false	false	0	true
MolPort-000-635-518	2	1	false	false	0	true
MolPort-004-892-988	2	1	false	false	0	true
MolPort-002-661-402	2	1	false	false	0	true
MolPort-007-675-208	2	1	false	false	0	true
MolPort-007-675-476	2	2	false	false	0	true
MolPort-010-961-261	2	2	false	false	0	true
MolPort-000-051-585	2	1	false	false	0	true
MolPort-000-819-177	2	1	false	false	0	true
MolPort-002-661-554	2	1	false	false	0	true
MolPort-006-421-577	2	1	false	false	0	true
MolPort-003-029-129	2	2	false	false	0	true
MolPort-007-675-450	2	1	false	false	0	true
MolPort-006-505-334	2	2	false	false	0	true
MolPort-000-035-393	2	1	false	false	0	true
MolPort-007-675-413	2	2	false	false	0	true
MolPort-007-734-561	2	1	false	false	0	true
MolPort-009-713-331	2	1	false	false	0	true
MolPort-000-635-507	2	2	false	false	0	true
MolPort-006-386-336	2	1	false	false	0	true
MolPort-002-090-650	2	1	false	false	0	true
MolPort-008-325-352	2	1	false	false	0	true
MolPort-007-677-224	2	2	false	false	0	true
MolPort-000-637-240	2	2	false	false	0	true
MolPort-010-806-753	2	2	false	false	0	true
MolPort-002-668-995	2	1	false	false	0	true
MolPort-007-598-740	2	2	false	false	0	true
MolPort-007-675-106	2	2	false	false	0	true
MolPort-010-961-367	2	1	false	false	0	true
MolPort-000-635-519	2	2	false	false	0	true
MolPort-007-582-340	2	1	false	false	0	true

COMPOUNDS	PARAMETERS					
	SOLUBILITY	BLOOD-CEPHALIC BARRIER	CYP2D6	HEPATOTOXICITY	INTESTINAL ABSORPTION	BLOOD PLASMA PROTEIN BINDING
MolPort-009-713-363	2	1	false	false	0	true
MolPort-009-713-324	2	1	false	false	0	true
MolPort-002-227-231	2	1	false	false	0	true
MolPort-010-814-757	2	2	false	false	0	true
MolPort-006-421-574	2	1	false	false	0	true
MolPort-000-635-523	2	1	false	false	0	true
MolPort-007-734-570	2	1	false	false	0	true
MolPort-008-317-301	2	2	false	false	0	true
MolPort-008-325-404	2	2	false	false	0	true
MolPort-010-806-840	2	1	false	false	0	true
MolPort-007-675-462	2	2	false	false	0	true
MolPort-007-608-238	2	1	false	false	0	true
MolPort-000-726-045	2	2	false	false	0	true
MolPort-001-958-480	2	1	false	false	0	true
MolPort-003-107-500	2	2	false	false	0	true
MolPort-009-713-328	2	1	false	false	0	true
MolPort-002-775-342	2	1	false	false	0	true
MolPort-009-713-369	2	1	false	false	0	true
MolPort-007-675-438	2	1	false	false	0	true
MolPort-001-954-699	2	1	false	false	0	true
MolPort-002-169-299	2	1	false	false	0	true
MolPort-000-635-516	2	1	false	false	0	true
MolPort-009-713-346	2	2	false	false	0	true
MolPort-005-509-773	2	2	false	false	0	true
MolPort-010-961-369	2	1	false	false	0	true
MolPort-000-635-525	2	2	false	false	0	true
MolPort-010-961-391	2	1	false	false	0	true
MolPort-000-040-197	2	1	false	false	0	true
MolPort-000-819-162	2	1	false	false	0	true
MolPort-002-273-130	2	1	false	false	0	true
MolPort-007-677-227	2	2	false	false	0	true
MolPort-001-892-669	2	1	false	false	0	true
MolPort-010-961-342	2	2	false	false	0	true

Table S2: List of all compounds selected in the toxicological stage from QNZ

COMPOUNDS	PARAMETERS										
	NTP Mouse		NTP Rat		Mouse FDA		Rat FDA		WOE	AMES	DTP
	Female	Male	Female	Male	Female	Male	Female	Male			
MolPort-000-728-326	Non-Carcinogen	Non-Carcinogen	Non-Carcinogen	Non-Carcinogen	Non-Carcinogen	Non-Carcinogen	Non-Carcinogen	Non-Carcinogen	Non-Carcinogen	Non-Mutagen	Non-Toxic
MolPort-000-744-347	Non-Carcinogen	Non-Carcinogen	Non-Carcinogen	Non-Carcinogen	Non-Carcinogen	Non-Carcinogen	Non-Carcinogen	Non-Carcinogen	Non-Carcinogen	Non-Mutagen	Non-Toxic
MolPort-002-709-662	Non-Carcinogen	Non-Carcinogen	Non-Carcinogen	Non-Carcinogen	Non-Carcinogen	Non-Carcinogen	Non-Carcinogen	Non-Carcinogen	Non-Carcinogen	Non-Mutagen	Non-Toxic
MolPort-007-975-257	Non-Carcinogen	Non-Carcinogen	Non-Carcinogen	Non-Carcinogen	Non-Carcinogen	Non-Carcinogen	Non-Carcinogen	Non-Carcinogen	Non-Carcinogen	Non-Mutagen	Non-Toxic
MolPort-001-532-277	Non-Carcinogen	Non-Carcinogen	Non-Carcinogen	Non-Carcinogen	Non-Carcinogen	Non-Carcinogen	Non-Carcinogen	Non-Carcinogen	Non-Carcinogen	Non-Mutagen	Non-Toxic
MolPort-010-785-753	Non-Carcinogen	Non-Carcinogen	Non-Carcinogen	Non-Carcinogen	Non-Carcinogen	Non-Carcinogen	Non-Carcinogen	Non-Carcinogen	Non-Carcinogen	Non-Mutagen	Non-Toxic
MolPort-001-979-697	Non-Carcinogen	Non-Carcinogen	Non-Carcinogen	Non-Carcinogen	Non-Carcinogen	Non-Carcinogen	Non-Carcinogen	Non-Carcinogen	Non-Carcinogen	Non-Mutagen	Non-Toxic
MolPort-007-975-293	Non-Carcinogen	Non-Carcinogen	Non-Carcinogen	Non-Carcinogen	Non-Carcinogen	Non-Carcinogen	Non-Carcinogen	Non-Carcinogen	Non-Carcinogen	Non-Mutagen	Non-Toxic
MolPort-001-633-725	Non-Carcinogen	Non-Carcinogen	Non-Carcinogen	Non-Carcinogen	Non-Carcinogen	Non-Carcinogen	Non-Carcinogen	Non-Carcinogen	Non-Carcinogen	Non-Mutagen	Non-Toxic
MolPort-019-931-888	Non-Carcinogen	Non-Carcinogen	Non-Carcinogen	Non-Carcinogen	Non-Carcinogen	Non-Carcinogen	Non-Carcinogen	Non-Carcinogen	Non-Carcinogen	Non-Mutagen	Non-Toxic
MolPort-002-288-462	Non-Carcinogen	Non-Carcinogen	Non-Carcinogen	Non-Carcinogen	Non-Carcinogen	Non-Carcinogen	Non-Carcinogen	Non-Carcinogen	Non-Carcinogen	Non-Mutagen	Non-Toxic
MolPort-002-665-311	Non-Carcinogen	Non-Carcinogen	Non-Carcinogen	Non-Carcinogen	Non-Carcinogen	Non-Carcinogen	Non-Carcinogen	Non-Carcinogen	Non-Carcinogen	Non-Mutagen	Non-Toxic
MolPort-001-965-869	Non-Carcinogen	Non-Carcinogen	Non-Carcinogen	Non-Carcinogen	Non-Carcinogen	Non-Carcinogen	Non-Carcinogen	Non-Carcinogen	Non-Carcinogen	Non-Mutagen	Non-Toxic
MolPort-001-965-870	Non-Carcinogen	Non-Carcinogen	Non-Carcinogen	Non-Carcinogen	Non-Carcinogen	Non-Carcinogen	Non-Carcinogen	Non-Carcinogen	Non-Carcinogen	Non-Mutagen	Non-Toxic
MolPort-001-913-400	Non-Carcinogen	Non-Carcinogen	Non-Carcinogen	Non-Carcinogen	Non-Carcinogen	Non-Carcinogen	Non-Carcinogen	Non-Carcinogen	Non-Carcinogen	Non-Mutagen	Non-Toxic
MolPort-008-333-219	Non-Carcinogen	Non-Carcinogen	Non-Carcinogen	Non-Carcinogen	Non-Carcinogen	Non-Carcinogen	Non-Carcinogen	Non-Carcinogen	Non-Carcinogen	Non-Mutagen	Non-Toxic
MolPort-002-003-292	Non-Carcinogen	Non-Carcinogen	Non-Carcinogen	Non-Carcinogen	Non-Carcinogen	Non-Carcinogen	Non-Carcinogen	Non-Carcinogen	Non-Carcinogen	Non-Mutagen	Non-Toxic
MolPort-002-668-721	Non-Carcinogen	Non-Carcinogen	Non-Carcinogen	Non-Carcinogen	Non-Carcinogen	Non-Carcinogen	Non-Carcinogen	Non-Carcinogen	Non-Carcinogen	Non-Mutagen	Non-Toxic
MolPort-008-344-861	Non-Carcinogen	Non-Carcinogen	Non-Carcinogen	Non-Carcinogen	Non-Carcinogen	Non-Carcinogen	Non-Carcinogen	Non-Carcinogen	Non-Carcinogen	Non-Mutagen	Non-Toxic
MolPort-008-342-117	Non-Carcinogen	Non-Carcinogen	Non-Carcinogen	Non-Carcinogen	Non-Carcinogen	Non-Carcinogen	Non-Carcinogen	Non-Carcinogen	Non-Carcinogen	Non-Mutagen	Non-Toxic
MolPort-016-900-298	Non-Carcinogen	Non-Carcinogen	Non-Carcinogen	Non-Carcinogen	Non-Carcinogen	Non-Carcinogen	Non-Carcinogen	Non-Carcinogen	Non-Carcinogen	Non-Mutagen	Non-Toxic
MolPort-016-910-415	Non-Carcinogen	Non-Carcinogen	Non-Carcinogen	Non-Carcinogen	Non-Carcinogen	Non-Carcinogen	Non-Carcinogen	Non-Carcinogen	Non-Carcinogen	Non-Mutagen	Non-Toxic
MolPort-002-274-146	Non-Carcinogen	Non-Carcinogen	Non-Carcinogen	Non-Carcinogen	Non-Carcinogen	Non-Carcinogen	Non-Carcinogen	Non-Carcinogen	Non-Carcinogen	Non-Mutagen	Non-Toxic
MolPort-008-344-602	Non-Carcinogen	Non-Carcinogen	Non-Carcinogen	Non-Carcinogen	Non-Carcinogen	Non-Carcinogen	Non-Carcinogen	Non-Carcinogen	Non-Carcinogen	Non-Mutagen	Non-Toxic
MolPort-002-021-656	Non-Carcinogen	Non-Carcinogen	Non-Carcinogen	Non-Carcinogen	Non-Carcinogen	Non-Carcinogen	Non-Carcinogen	Non-Carcinogen	Non-Carcinogen	Non-Mutagen	Non-Toxic
MolPort-001-964-357	Non-Carcinogen	Non-Carcinogen	Non-Carcinogen	Non-Carcinogen	Non-Carcinogen	Non-Carcinogen	Non-Carcinogen	Non-Carcinogen	Non-Carcinogen	Non-Mutagen	Non-Toxic
MolPort-007-608-380	Non-Carcinogen	Non-Carcinogen	Non-Carcinogen	Non-Carcinogen	Non-Carcinogen	Non-Carcinogen	Non-Carcinogen	Non-Carcinogen	Non-Carcinogen	Non-Mutagen	Non-Toxic
MolPort-001-997-751	Non-Carcinogen	Non-Carcinogen	Non-Carcinogen	Non-Carcinogen	Non-Carcinogen	Non-Carcinogen	Non-Carcinogen	Non-Carcinogen	Non-Carcinogen	Non-Mutagen	Non-Toxic
MolPort-000-739-421	Non-Carcinogen	Non-Carcinogen	Non-Carcinogen	Non-Carcinogen	Non-Carcinogen	Non-Carcinogen	Non-Carcinogen	Non-Carcinogen	Non-Carcinogen	Non-Mutagen	Non-Toxic

COMPOUNDS	PARAMETERS										
	NTP Mouse		NTP Rat		Mouse FDA		Rat FDA		WOE	AMES	DTP
	Female	Male	Female	Male	Female	Male	Female	Male			
MolPort-009-713-376	Non-Carcinogen	Non-Carcinogen	Non-Carcinogen	Non-Carcinogen	Non-Carcinogen	Non-Carcinogen	Non-Carcinogen	Non-Carcinogen	Non-Carcinogen	Non-Mutagen	Non-Toxic
MolPort-009-713-357	Non-Carcinogen	Non-Carcinogen	Non-Carcinogen	Non-Carcinogen	Non-Carcinogen	Non-Carcinogen	Non-Carcinogen	Non-Carcinogen	Non-Carcinogen	Non-Mutagen	Non-Toxic
MolPort-047-898-129	Non-Carcinogen	Non-Carcinogen	Non-Carcinogen	Non-Carcinogen	Non-Carcinogen	Non-Carcinogen	Non-Carcinogen	Non-Carcinogen	Non-Carcinogen	Non-Mutagen	Non-Toxic
MolPort-004-832-102	Non-Carcinogen	Non-Carcinogen	Non-Carcinogen	Non-Carcinogen	Non-Carcinogen	Non-Carcinogen	Non-Carcinogen	Non-Carcinogen	Non-Carcinogen	Non-Mutagen	Non-Toxic
MolPort-003-965-540	Non-Carcinogen	Non-Carcinogen	Non-Carcinogen	Non-Carcinogen	Non-Carcinogen	Non-Carcinogen	Non-Carcinogen	Non-Carcinogen	Non-Carcinogen	Non-Mutagen	Non-Toxic
MolPort-002-017-868	Non-Carcinogen	Non-Carcinogen	Non-Carcinogen	Non-Carcinogen	Non-Carcinogen	Non-Carcinogen	Non-Carcinogen	Non-Carcinogen	Non-Carcinogen	Non-Mutagen	Non-Toxic
MolPort-004-832-105	Non-Carcinogen	Non-Carcinogen	Non-Carcinogen	Non-Carcinogen	Non-Carcinogen	Non-Carcinogen	Non-Carcinogen	Non-Carcinogen	Non-Carcinogen	Non-Mutagen	Non-Toxic
MolPort-008-334-857	Non-Carcinogen	Non-Carcinogen	Non-Carcinogen	Non-Carcinogen	Non-Carcinogen	Non-Carcinogen	Non-Carcinogen	Non-Carcinogen	Non-Carcinogen	Non-Mutagen	Non-Toxic
MolPort-007-579-520	Non-Carcinogen	Non-Carcinogen	Non-Carcinogen	Non-Carcinogen	Non-Carcinogen	Non-Carcinogen	Non-Carcinogen	Non-Carcinogen	Non-Carcinogen	Non-Mutagen	Non-Toxic
MolPort-003-029-079	Non-Carcinogen	Non-Carcinogen	Non-Carcinogen	Non-Carcinogen	Non-Carcinogen	Non-Carcinogen	Non-Carcinogen	Non-Carcinogen	Non-Carcinogen	Non-Mutagen	Non-Toxic
MolPort-000-853-802	Non-Carcinogen	Non-Carcinogen	Non-Carcinogen	Non-Carcinogen	Non-Carcinogen	Non-Carcinogen	Non-Carcinogen	Non-Carcinogen	Non-Carcinogen	Non-Mutagen	Non-Toxic
MolPort-003-029-080	Non-Carcinogen	Non-Carcinogen	Non-Carcinogen	Non-Carcinogen	Non-Carcinogen	Non-Carcinogen	Non-Carcinogen	Non-Carcinogen	Non-Carcinogen	Non-Mutagen	Non-Toxic
MolPort-009-713-335	Non-Carcinogen	Non-Carcinogen	Non-Carcinogen	Non-Carcinogen	Non-Carcinogen	Non-Carcinogen	Non-Carcinogen	Non-Carcinogen	Non-Carcinogen	Non-Mutagen	Non-Toxic
MolPort-004-832-103	Non-Carcinogen	Non-Carcinogen	Non-Carcinogen	Non-Carcinogen	Non-Carcinogen	Non-Carcinogen	Non-Carcinogen	Non-Carcinogen	Non-Carcinogen	Non-Mutagen	Non-Toxic
MolPort-001-844-431	Non-Carcinogen	Non-Carcinogen	Non-Carcinogen	Non-Carcinogen	Non-Carcinogen	Non-Carcinogen	Non-Carcinogen	Non-Carcinogen	Non-Carcinogen	Non-Mutagen	Non-Toxic
MolPort-000-734-010	Non-Carcinogen	Non-Carcinogen	Non-Carcinogen	Non-Carcinogen	Non-Carcinogen	Non-Carcinogen	Non-Carcinogen	Non-Carcinogen	Non-Carcinogen	Non-Mutagen	Non-Toxic
MolPort-009-713-358	Non-Carcinogen	Non-Carcinogen	Non-Carcinogen	Non-Carcinogen	Non-Carcinogen	Non-Carcinogen	Non-Carcinogen	Non-Carcinogen	Non-Carcinogen	Non-Mutagen	Non-Toxic
MolPort-000-657-398	Non-Carcinogen	Non-Carcinogen	Non-Carcinogen	Non-Carcinogen	Non-Carcinogen	Non-Carcinogen	Non-Carcinogen	Non-Carcinogen	Non-Carcinogen	Non-Mutagen	Non-Toxic
MolPort-000-047-065	Non-Carcinogen	Non-Carcinogen	Non-Carcinogen	Non-Carcinogen	Non-Carcinogen	Non-Carcinogen	Non-Carcinogen	Non-Carcinogen	Non-Carcinogen	Non-Mutagen	Non-Toxic
MolPort-003-029-416	Non-Carcinogen	Non-Carcinogen	Non-Carcinogen	Non-Carcinogen	Non-Carcinogen	Non-Carcinogen	Non-Carcinogen	Non-Carcinogen	Non-Carcinogen	Non-Mutagen	Non-Toxic
MolPort-009-701-491	Non-Carcinogen	Non-Carcinogen	Non-Carcinogen	Non-Carcinogen	Non-Carcinogen	Non-Carcinogen	Non-Carcinogen	Non-Carcinogen	Non-Carcinogen	Non-Mutagen	Non-Toxic
MolPort-000-051-577	Non-Carcinogen	Non-Carcinogen	Non-Carcinogen	Non-Carcinogen	Non-Carcinogen	Non-Carcinogen	Non-Carcinogen	Non-Carcinogen	Non-Carcinogen	Non-Mutagen	Non-Toxic
MolPort-009-713-368	Non-Carcinogen	Non-Carcinogen	Non-Carcinogen	Non-Carcinogen	Non-Carcinogen	Non-Carcinogen	Non-Carcinogen	Non-Carcinogen	Non-Carcinogen	Non-Mutagen	Non-Toxic
MolPort-016-910-006	Non-Carcinogen	Non-Carcinogen	Non-Carcinogen	Non-Carcinogen	Non-Carcinogen	Non-Carcinogen	Non-Carcinogen	Non-Carcinogen	Non-Carcinogen	Non-Mutagen	Non-Toxic
MolPort-002-005-903	Non-Carcinogen	Non-Carcinogen	Non-Carcinogen	Non-Carcinogen	Non-Carcinogen	Non-Carcinogen	Non-Carcinogen	Non-Carcinogen	Non-Carcinogen	Non-Mutagen	Non-Toxic
MolPort-003-872-842	Non-Carcinogen	Non-Carcinogen	Non-Carcinogen	Non-Carcinogen	Non-Carcinogen	Non-Carcinogen	Non-Carcinogen	Non-Carcinogen	Non-Carcinogen	Non-Mutagen	Non-Toxic
MolPort-001-994-765	Non-Carcinogen	Non-Carcinogen	Non-Carcinogen	Non-Carcinogen	Non-Carcinogen	Non-Carcinogen	Non-Carcinogen	Non-Carcinogen	Non-Carcinogen	Non-Mutagen	Non-Toxic
MolPort-009-713-329	Non-Carcinogen	Non-Carcinogen	Non-Carcinogen	Non-Carcinogen	Non-Carcinogen	Non-Carcinogen	Non-Carcinogen	Non-Carcinogen	Non-Carcinogen	Non-Mutagen	Non-Toxic
MolPort-002-592-406	Non-Carcinogen	Non-Carcinogen	Non-Carcinogen	Non-Carcinogen	Non-Carcinogen	Non-Carcinogen	Non-Carcinogen	Non-Carcinogen	Non-Carcinogen	Non-Mutagen	Non-Toxic
MolPort-023-300-288	Non-Carcinogen	Non-Carcinogen	Non-Carcinogen	Non-Carcinogen	Non-Carcinogen	Non-Carcinogen	Non-Carcinogen	Non-Carcinogen	Non-Carcinogen	Non-Mutagen	Non-Toxic

COMPOUNDS	PARAMETERS										
	NTP Mouse		NTP Rat		Mouse FDA		Rat FDA		WOE	AMES	DTP
	Female	Male	Female	Male	Female	Male	Female	Male			
MolPort-007-635-930	Non-Carcinogen	Non-Carcinogen	Non-Carcinogen	Non-Carcinogen	Non-Carcinogen	Non-Carcinogen	Non-Carcinogen	Non-Carcinogen	Non-Carcinogen	Non-Mutagen	Non-Toxic
MolPort-000-087-187	Non-Carcinogen	Non-Carcinogen	Non-Carcinogen	Non-Carcinogen	Non-Carcinogen	Non-Carcinogen	Non-Carcinogen	Non-Carcinogen	Non-Carcinogen	Non-Mutagen	Non-Toxic
MolPort-003-029-106	Non-Carcinogen	Non-Carcinogen	Non-Carcinogen	Non-Carcinogen	Non-Carcinogen	Non-Carcinogen	Non-Carcinogen	Non-Carcinogen	Non-Carcinogen	Non-Mutagen	Non-Toxic
MolPort-005-921-169	Non-Carcinogen	Non-Carcinogen	Non-Carcinogen	Non-Carcinogen	Non-Carcinogen	Non-Carcinogen	Non-Carcinogen	Non-Carcinogen	Non-Carcinogen	Non-Mutagen	Non-Toxic
MolPort-010-661-897	Non-Carcinogen	Non-Carcinogen	Non-Carcinogen	Non-Carcinogen	Non-Carcinogen	Non-Carcinogen	Non-Carcinogen	Non-Carcinogen	Non-Carcinogen	Non-Mutagen	Non-Toxic
MolPort-003-029-230	Non-Carcinogen	Non-Carcinogen	Non-Carcinogen	Non-Carcinogen	Non-Carcinogen	Non-Carcinogen	Non-Carcinogen	Non-Carcinogen	Non-Carcinogen	Non-Mutagen	Non-Toxic
MolPort-009-713-337	Non-Carcinogen	Non-Carcinogen	Non-Carcinogen	Non-Carcinogen	Non-Carcinogen	Non-Carcinogen	Non-Carcinogen	Non-Carcinogen	Non-Carcinogen	Non-Mutagen	Non-Toxic
MolPort-008-325-405	Non-Carcinogen	Non-Carcinogen	Non-Carcinogen	Non-Carcinogen	Non-Carcinogen	Non-Carcinogen	Non-Carcinogen	Non-Carcinogen	Non-Carcinogen	Non-Mutagen	Non-Toxic
MolPort-006-421-577	Non-Carcinogen	Non-Carcinogen	Non-Carcinogen	Non-Carcinogen	Non-Carcinogen	Non-Carcinogen	Non-Carcinogen	Non-Carcinogen	Non-Carcinogen	Non-Mutagen	Non-Toxic
MolPort-000-035-393	Non-Carcinogen	Non-Carcinogen	Non-Carcinogen	Non-Carcinogen	Non-Carcinogen	Non-Carcinogen	Non-Carcinogen	Non-Carcinogen	Non-Carcinogen	Non-Mutagen	Non-Toxic
MolPort-002-090-650	Non-Carcinogen	Non-Carcinogen	Non-Carcinogen	Non-Carcinogen	Non-Carcinogen	Non-Carcinogen	Non-Carcinogen	Non-Carcinogen	Non-Carcinogen	Non-Mutagen	Non-Toxic
MolPort-008-325-352	Non-Carcinogen	Non-Carcinogen	Non-Carcinogen	Non-Carcinogen	Non-Carcinogen	Non-Carcinogen	Non-Carcinogen	Non-Carcinogen	Non-Carcinogen	Non-Mutagen	Non-Toxic
MolPort-007-608-238	Non-Carcinogen	Non-Carcinogen	Non-Carcinogen	Non-Carcinogen	Non-Carcinogen	Non-Carcinogen	Non-Carcinogen	Non-Carcinogen	Non-Carcinogen	Non-Mutagen	Non-Toxic
MolPort-009-713-369	Non-Carcinogen	Non-Carcinogen	Non-Carcinogen	Non-Carcinogen	Non-Carcinogen	Non-Carcinogen	Non-Carcinogen	Non-Carcinogen	Non-Carcinogen	Non-Mutagen	Non-Toxic
MolPort-001-954-699	Non-Carcinogen	Non-Carcinogen	Non-Carcinogen	Non-Carcinogen	Non-Carcinogen	Non-Carcinogen	Non-Carcinogen	Non-Carcinogen	Non-Carcinogen	Non-Mutagen	Non-Toxic
MolPort-002-169-299	Non-Carcinogen	Non-Carcinogen	Non-Carcinogen	Non-Carcinogen	Non-Carcinogen	Non-Carcinogen	Non-Carcinogen	Non-Carcinogen	Non-Carcinogen	Non-Mutagen	Non-Toxic
MolPort-002-273-130	Non-Carcinogen	Non-Carcinogen	Non-Carcinogen	Non-Carcinogen	Non-Carcinogen	Non-Carcinogen	Non-Carcinogen	Non-Carcinogen	Non-Carcinogen	Non-Mutagen	Non-Toxic
MolPort-010-961-342	Non-Carcinogen	Non-Carcinogen	Non-Carcinogen	Non-Carcinogen	Non-Carcinogen	Non-Carcinogen	Non-Carcinogen	Non-Carcinogen	Non-Carcinogen	Non-Mutagen	Non-Toxic

Table S3: Molecular docking of the compounds selected in the toxicological screening from QNZ, the Top5 are those mentioned in the study

ID	COMPOUNDS	BINDING ENERGY(Kcal/mol)
**4CAV01**	Molport-002-274-146	-8.483
**4CAV02**	Molport-003-029-230	-8.383
**4CAV03**	Molport-003-029-106	-8.355
**4CAV04**	Molport-003-029-079	-8.341
**4CAV05**	Molport-003-029-080	-8.334
4CAV06	Molport-019-931-888	-8.260
4CAV07	Molport-005-921-169	-8.243
4CAV08	Molport-002-665-311	-8.242
4CAV09	Molport-008-344-602	-8.173
4CAV10	Molport-008-344-861	-8.164
4CAV11	Molport-001-965-870	-8.133
4CAV12	Molport-001-954-699	-8.124
4CAV13	Molport-003-965-540	-8.120
4CAV14	Molport-002-169-299	-8.099
4CAV15	Molport-009-701-491	-8.095
4CAV16	Molport-004-832-102	-8.084
4CAV17	Molport-000-035-393	-8.084
**Template**	QNZ	-8.080
4CAV18	Molport-007-975-257	-8.076
4CAV19	Molport-000-739-421	-8.073
4CAV20	Molport-002-668-721	-8.066
4CAV21	Molport-002-003-292	-8.059
4CAV22	Molport-001-965-869	-8.027
4CAV23	Molport-023-300-288	-8.013
4CAV24	Molport-002-021-656	-8.004
4CAV25	Molport-007-579-520	-7.977
4CAV26	Molport-006-421-577	-7.976
4CAV27	Molport-001-964-357	-7.970
4CAV28	Molport-008-325-352	-7.969
4CAV29	Molport-002-592-406	-7.950

ID	COMPOUNDS	BINDING ENERGY(Kcal/mol)
4CAV30	Molport-000-734-010	-7.936
4CAV31	Molport-010-785-753	-7.932
4CAV32	Molport-047-898-129	-7.928
4CAV33	Molport-002-017-868	-7.921
4CAV34	Molport-010-661-897	-7.919
4CAV35	Molport-004-832-105	-7.899
4CAV36	Molport-007-608-238	-7.899
4CAV37	Molport-001-994-765	-7.885
4CAV38	Molport-007-975-293	-7.877
4CAV39	Molport-000-087-187	-7.872
4CAV40	Molport-016-910-415	-7.849
4CAV41	Molport-016-910-006	-7.837
4CAV42	Molport-002-090-650	-7.819
4CAV43	Molport-002-288-462	-7.802
4CAV44	Molport-000-051-577	-7.792
4CAV45	Molport-004-832-103	-7.785
4CAV46	Molport-016-900-298	-7.771
4CAV47	Molport-008-333-219	-7.761
4CAV48	Molport-002-273-130	-7.758
4CAV49	Molport-007-635-930	-7.727
4CAV50	Molport-003-029-416	-7.720
4CAV51	Molport-003-872-842	-7.682
4CAV52	Molport-001-913-400	-7.672
4CAV53	Molport-000-657-398	-7.660
4CAV54	Molport-009-713-335	-7.631
4CAV55	Molport-001-997-751	-7.628
4CAV56	Molport-009-713-369	-7.619
4CAV57	Molport-009-713-368	-7.607
4CAV58	Molport-009-713-329	-7.594
4CAV59	Molport-008-334-857	-7.562
4CAV60	Molport-008-342-117	-7.551
4CAV61	Molport-000-047-065	-7.545

ID	COMPOUNDS	BINDING ENERGY(Kcal/mol)
4CAV62	Molport-000-728-326	-7.539
4CAV63	Molport-009-713-376	-7.522
4CAV64	Molport-009-713-357	-7.521
4CAV65	Molport-001-979-697	-7.520
4CAV66	Molport-010-961-342	-7.516
4CAV67	Molport-001-633-725	-7.503
4CAV68	Molport-002-005-903	-7.492
4CAV69	Molport-000-853-802	-7.483
4CAV70	Molport-008-325-405	-7.462
4CAV71	Molport-009-713-358	-7.443
4CAV72	Molport-009-713-337	-7.423
4CAV73	Molport-001-532-277	-7.393
4CAV74	Molport-000-744-347	-7.385
4CAV75	Molport-007-608-380	-7.364
4CAV76	Molport-002-709-662	-7.315
4CAV77	Molport-001-844-431	-7.261

Figure S1: Interaction with amino acid residues of the Top5 compounds screened from QNZ

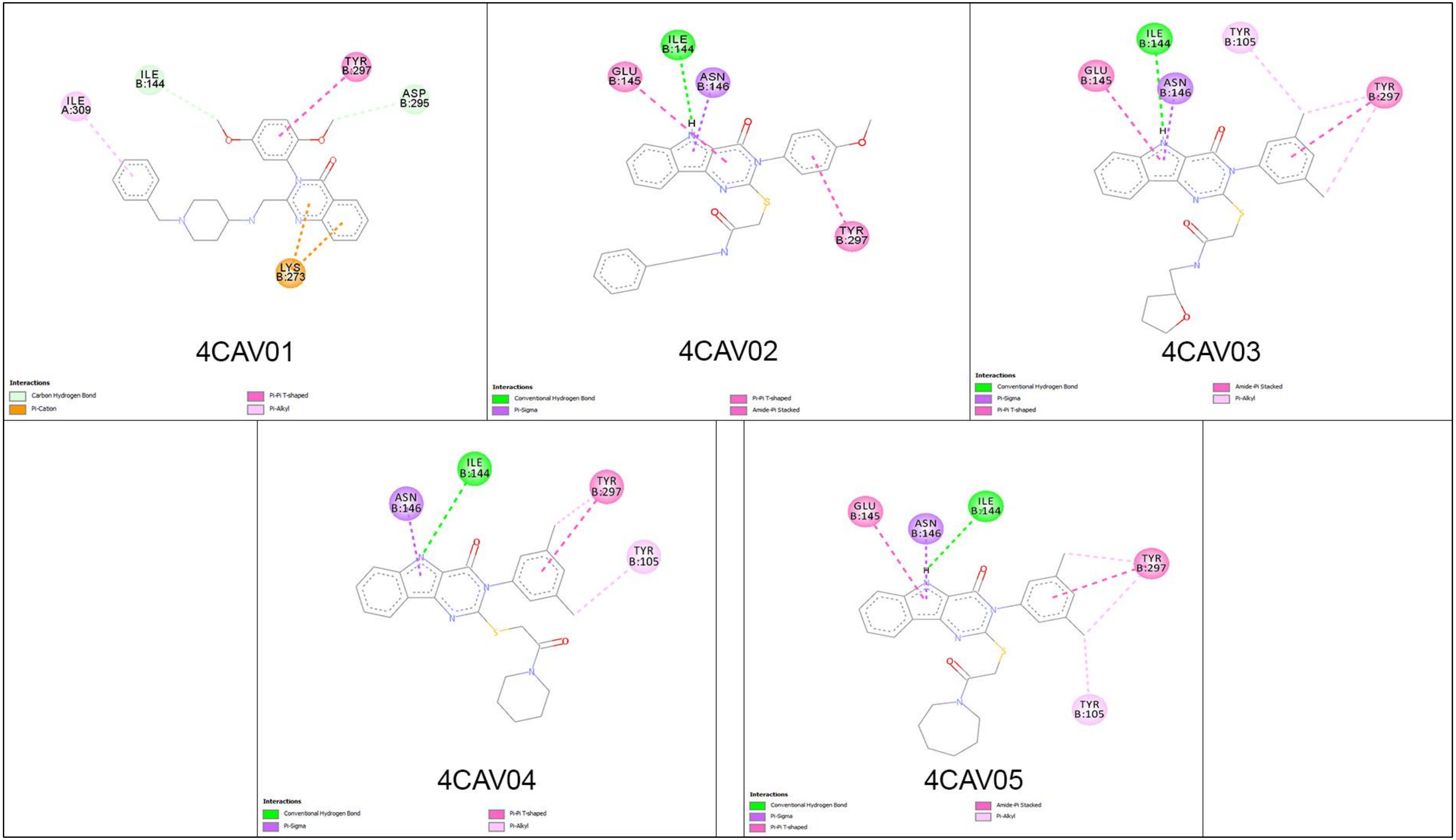

Table S4: List of all compounds selected in the pharmacokinetic step of 0Y5

COMPOUNDS	PARAMETERS					
	SOLUBILITY	BLOOD-CEPHALIC BARRIER	CYP2D6	HEPATOTOXICITY	INTESTINAL ABSORPTION	BLOOD PLASMA PROTEIN BINDING
MolPort-046-839-344	3	2	false	false	0	true
MolPort-000-134-945	3	3	false	false	0	true
MolPort-000-134-723	3	3	false	false	0	true
MolPort-016-664-393	3	3	false	false	0	true
MolPort-016-699-781	3	2	false	false	0	true
MolPort-042-617-134	3	3	false	false	0	true
MolPort-019-720-423	3	3	false	false	0	true
MolPort-016-718-488	3	3	false	false	0	true
MolPort-005-059-930	4	3	false	false	0	true
MolPort-016-664-399	3	3	false	false	0	true
MolPort-019-720-501	3	3	false	false	0	true
MolPort-016-664-403	3	3	false	false	0	true
MolPort-005-013-046	4	3	false	false	0	true
MolPort-016-668-568	3	2	false	false	0	true
MolPort-005-032-127	3	2	false	false	0	true
MolPort-009-715-125	3	3	false	false	0	true
MolPort-016-668-550	3	3	false	false	0	true
MolPort-019-720-425	3	3	false	false	0	true
MolPort-005-026-461	3	2	false	false	0	true
MolPort-000-134-790	3	3	false	false	0	true
MolPort-016-699-632	3	3	false	false	0	true
MolPort-028-854-465	3	3	false	false	0	true
MolPort-016-662-953	3	3	false	false	0	true
MolPort-016-666-791	3	3	false	false	0	true
MolPort-028-609-066	3	3	false	false	0	true
MolPort-044-309-641	3	3	false	false	0	true
MolPort-044-313-041	3	3	false	false	0	true
MolPort-005-083-030	3	3	false	false	0	true
MolPort-047-674-574	3	3	false	false	0	true
MolPort-019-720-421	3	3	false	false	0	true
MolPort-005-036-359	3	2	false	false	0	true
MolPort-047-210-340	3	2	false	false	0	true
MolPort-005-022-477	3	2	false	false	0	true
MolPort-016-664-391	3	3	false	false	0	true
MolPort-003-125-711	3	3	false	false	0	true
MolPort-039-102-749	3	3	false	false	0	true
MolPort-016-668-547	3	3	false	false	0	true

COMPOUNDS	PARAMETERS					
	SOLUBILITY	BLOOD-CEPHALIC BARRIER	CYP2D6	HEPATOTOXICITY	INTESTINAL ABSORPTION	BLOOD PLASMA PROTEIN BINDING
MolPort-005-071-503	3	3	false	false	0	true
MolPort-005-016-924	3	3	false	false	0	true
MolPort-016-668-567	3	2	false	false	0	true
MolPort-005-046-014	3	2	false	false	0	true
MolPort-019-720-482	3	3	false	false	0	true
MolPort-016-664-392	3	3	false	false	0	true
MolPort-005-052-452	3	3	false	false	0	true
MolPort-028-609-119	3	3	false	false	0	true
MolPort-046-839-431	3	3	false	false	0	true
MolPort-000-820-749	3	3	false	false	0	true
MolPort-005-010-547	3	2	false	false	0	true
MolPort-005-038-231	3	2	false	false	0	true
MolPort-004-999-739	3	3	false	false	0	true
MolPort-005-043-958	3	2	false	false	0	true
MolPort-046-601-735	3	3	false	false	0	true
MolPort-044-312-579	3	3	false	false	0	true
MolPort-044-313-085	3	3	false	false	0	true
MolPort-005-006-000	3	3	false	false	0	true
MolPort-005-059-909	3	3	false	false	0	true
MolPort-046-177-951	3	2	false	false	0	true
MolPort-008-329-796	3	3	false	false	0	true
MolPort-009-715-127	3	3	false	false	0	true
MolPort-005-005-087	3	2	false	false	0	true
MolPort-039-103-052	3	3	false	false	0	true
MolPort-005-003-108	3	3	false	false	0	true
MolPort-005-037-492	3	3	false	false	0	true
MolPort-016-666-561	3	3	false	false	0	true
MolPort-051-704-722	3	3	false	false	0	true
MolPort-046-192-370	3	3	false	false	0	true
MolPort-005-012-831	3	2	false	false	0	true
MolPort-005-000-676	3	3	false	false	0	true
MolPort-016-699-684	3	3	false	false	0	true
MolPort-005-041-135	3	3	false	false	0	true
MolPort-010-746-765	3	3	false	false	0	true
MolPort-010-710-830	3	3	false	false	0	true
MolPort-051-592-148	3	2	false	false	0	true
MolPort-005-012-547	3	2	false	false	0	true
MolPort-005-092-473	3	2	false	false	0	true
MolPort-005-074-134	3	3	false	false	0	true

COMPOUNDS	PARAMETERS					
	SOLUBILITY	BLOOD-CEPHALIC BARRIER	CYP2D6	HEPATOTOXICITY	INTESTINAL ABSORPTION	BLOOD PLASMA PROTEIN BINDING
MolPort-039-105-887	3	2	false	false	0	true
MolPort-005-058-122	3	2	false	false	0	true
MolPort-008-283-589	3	2	false	false	0	true
MolPort-016-721-702	3	3	false	false	0	true
MolPort-010-746-700	3	3	false	false	0	true
MolPort-044-352-089	4	3	false	false	0	true
MolPort-005-063-722	3	3	false	false	0	true
MolPort-005-064-757	3	2	false	false	0	true
MolPort-007-674-419	3	3	false	false	0	true
MolPort-016-655-372	3	2	false	false	0	true
MolPort-005-031-705	3	2	false	false	0	true
MolPort-005-046-277	3	2	false	false	0	true
MolPort-039-104-467	3	2	false	false	0	true
MolPort-016-666-570	3	3	false	false	0	true
MolPort-039-122-085	3	2	false	false	0	true
MolPort-047-128-768	3	3	false	false	0	true
MolPort-009-706-524	3	3	false	false	0	true
MolPort-005-039-945	3	2	false	false	0	true
MolPort-005-101-024	3	3	false	false	0	true
MolPort-047-189-580	3	3	false	false	0	true
MolPort-005-012-554	3	3	false	false	0	true
MolPort-005-003-595	3	2	false	false	0	true
MolPort-005-006-865	3	3	false	false	0	true
MolPort-008-378-815	3	2	false	false	0	true
MolPort-002-669-875	3	2	false	false	0	true
MolPort-027-714-412	3	3	false	false	0	true
MolPort-016-666-796	3	3	false	false	0	true
MolPort-016-665-240	3	2	false	false	0	true
MolPort-002-663-534	3	3	false	false	0	true
MolPort-051-496-301	3	3	false	false	0	true
MolPort-020-227-141	3	3	false	false	0	true
MolPort-010-710-829	3	3	false	false	0	true
MolPort-005-069-159	3	3	false	false	0	true
MolPort-010-746-674	3	3	false	false	0	true
MolPort-010-976-150	3	2	false	false	0	true
MolPort-044-313-981	3	3	false	false	0	true
MolPort-016-668-431	3	3	false	false	0	true
MolPort-005-027-302	3	2	false	false	0	true
MolPort-047-183-756	3	3	false	false	0	true

COMPOUNDS	PARAMETERS					
	SOLUBILITY	BLOOD-CEPHALIC BARRIER	CYP2D6	HEPATOTOXICITY	INTESTINAL ABSORPTION	BLOOD PLASMA PROTEIN BINDING
MolPort-000-850-276	3	3	false	false	0	true
MolPort-044-638-272	3	2	false	false	0	true
MolPort-016-666-563	3	3	false	false	0	true
MolPort-002-649-964	3	2	false	false	0	true
MolPort-005-075-173	3	3	false	false	0	true
MolPort-005-043-977	3	3	false	false	0	true
MolPort-039-196-636	3	3	false	false	0	true
MolPort-028-599-998	3	3	false	false	0	true
MolPort-019-699-937	3	3	false	false	0	true
MolPort-007-629-455	3	3	false	false	0	true
MolPort-044-311-675	3	3	false	false	0	true
MolPort-016-642-774	3	3	false	false	0	true
MolPort-009-715-110	3	2	false	false	0	true
MolPort-005-029-917	3	3	false	false	0	true
MolPort-010-746-682	3	3	false	false	0	true
MolPort-005-001-709	3	3	false	false	0	true
MolPort-010-647-705	3	3	false	false	0	true
MolPort-042-675-531	3	3	false	false	0	true
MolPort-042-675-529	3	3	false	false	0	true
MolPort-046-601-741	3	2	false	false	0	true
MolPort-005-020-225	3	2	false	false	0	true
MolPort-009-715-108	3	3	false	false	0	true
MolPort-004-992-801	3	3	false	false	0	true
MolPort-005-000-171	3	3	false	false	0	true
MolPort-039-112-879	3	3	false	false	0	true
MolPort-047-661-718	3	3	false	false	0	true
MolPort-005-036-593	3	2	false	false	0	true
MolPort-005-089-476	3	2	false	false	0	true
MolPort-016-655-366	3	2	false	false	0	true
MolPort-005-007-020	3	2	false	false	0	true
MolPort-005-057-861	3	3	false	false	0	true
MolPort-047-373-313	3	3	false	false	0	true
MolPort-009-715-106	3	3	false	false	0	true
MolPort-046-601-847	3	3	false	false	0	true
MolPort-005-059-677	3	3	false	false	0	true
MolPort-000-791-076	3	3	false	false	0	true
MolPort-016-666-568	3	3	false	false	0	true
MolPort-047-011-115	3	3	false	false	0	true
MolPort-003-122-319	3	3	false	false	0	true

COMPOUNDS	PARAMETERS					
	SOLUBILITY	BLOOD-CEPHALIC BARRIER	CYP2D6	HEPATOTOXICITY	INTESTINAL ABSORPTION	BLOOD PLASMA PROTEIN BINDING
MolPort-005-020-529	3	3	false	false	0	true
MolPort-005-033-956	3	2	false	false	0	true
MolPort-010-710-806	3	3	false	false	0	true
MolPort-047-503-527	3	3	false	false	0	true
MolPort-047-182-226	3	3	false	false	0	true
MolPort-010-741-771	3	3	false	false	0	true
MolPort-002-861-891	3	3	false	false	0	true
MolPort-019-720-091	3	3	false	false	0	true
MolPort-010-663-222	3	3	false	false	0	true
MolPort-010-911-006	3	3	false	false	0	true
MolPort-010-901-286	3	2	false	false	0	true

Table S5: List of all compounds selected in the toxicological stage from 0Y5

COMPOUNDS	PARAMETERS										
	NTP Mouse		NTP Rat		Mouse FDA		Rat FDA		WOE	AMES	DTP
	Female	Male	Female	Male	Female	Male	Female	Male			
MolPort-042-617-134	Non-Carcinogen	Non-Carcinogen	Non-Carcinogen	Non-Carcinogen	Non-Carcinogen	Non-Carcinogen	Non-Carcinogen	Non-Carcinogen	Non-Carcinogen	Non-Mutagen	Non-Toxic
MolPort-019-720-501	Non-Carcinogen	Non-Carcinogen	Non-Carcinogen	Non-Carcinogen	Non-Carcinogen	Non-Carcinogen	Non-Carcinogen	Non-Carcinogen	Non-Carcinogen	Non-Mutagen	Non-Toxic
MolPort-016-668-568	Non-Carcinogen	Non-Carcinogen	Non-Carcinogen	Non-Carcinogen	Non-Carcinogen	Non-Carcinogen	Non-Carcinogen	Non-Carcinogen	Non-Carcinogen	Non-Mutagen	Non-Toxic
MolPort-019-720-425	Non-Carcinogen	Non-Carcinogen	Non-Carcinogen	Non-Carcinogen	Non-Carcinogen	Non-Carcinogen	Non-Carcinogen	Non-Carcinogen	Non-Carcinogen	Non-Mutagen	Non-Toxic
MolPort-028-854-465	Non-Carcinogen	Non-Carcinogen	Non-Carcinogen	Non-Carcinogen	Non-Carcinogen	Non-Carcinogen	Non-Carcinogen	Non-Carcinogen	Non-Carcinogen	Non-Mutagen	Non-Toxic
MolPort-044-309-641	Non-Carcinogen	Non-Carcinogen	Non-Carcinogen	Non-Carcinogen	Non-Carcinogen	Non-Carcinogen	Non-Carcinogen	Non-Carcinogen	Non-Carcinogen	Non-Mutagen	Non-Toxic
MolPort-044-313-041	Non-Carcinogen	Non-Carcinogen	Non-Carcinogen	Non-Carcinogen	Non-Carcinogen	Non-Carcinogen	Non-Carcinogen	Non-Carcinogen	Non-Carcinogen	Non-Mutagen	Non-Toxic
MolPort-003-125-711	Non-Carcinogen	Non-Carcinogen	Non-Carcinogen	Non-Carcinogen	Non-Carcinogen	Non-Carcinogen	Non-Carcinogen	Non-Carcinogen	Non-Carcinogen	Non-Mutagen	Non-Toxic
MolPort-046-839-431	Non-Carcinogen	Non-Carcinogen	Non-Carcinogen	Non-Carcinogen	Non-Carcinogen	Non-Carcinogen	Non-Carcinogen	Non-Carcinogen	Non-Carcinogen	Non-Mutagen	Non-Toxic
MolPort-000-820-749	Non-Carcinogen	Non-Carcinogen	Non-Carcinogen	Non-Carcinogen	Non-Carcinogen	Non-Carcinogen	Non-Carcinogen	Non-Carcinogen	Non-Carcinogen	Non-Mutagen	Non-Toxic
MolPort-005-043-958	Non-Carcinogen	Non-Carcinogen	Non-Carcinogen	Non-Carcinogen	Non-Carcinogen	Non-Carcinogen	Non-Carcinogen	Non-Carcinogen	Non-Carcinogen	Non-Mutagen	Non-Toxic
MolPort-046-601-735	Non-Carcinogen	Non-Carcinogen	Non-Carcinogen	Non-Carcinogen	Non-Carcinogen	Non-Carcinogen	Non-Carcinogen	Non-Carcinogen	Non-Carcinogen	Non-Mutagen	Non-Toxic
MolPort-044-312-579	Non-Carcinogen	Non-Carcinogen	Non-Carcinogen	Non-Carcinogen	Non-Carcinogen	Non-Carcinogen	Non-Carcinogen	Non-Carcinogen	Non-Carcinogen	Non-Mutagen	Non-Toxic
MolPort-044-313-085	Non-Carcinogen	Non-Carcinogen	Non-Carcinogen	Non-Carcinogen	Non-Carcinogen	Non-Carcinogen	Non-Carcinogen	Non-Carcinogen	Non-Carcinogen	Non-Mutagen	Non-Toxic
MolPort-046-177-951	Non-Carcinogen	Non-Carcinogen	Non-Carcinogen	Non-Carcinogen	Non-Carcinogen	Non-Carcinogen	Non-Carcinogen	Non-Carcinogen	Non-Carcinogen	Non-Mutagen	Non-Toxic
MolPort-008-329-796	Non-Carcinogen	Non-Carcinogen	Non-Carcinogen	Non-Carcinogen	Non-Carcinogen	Non-Carcinogen	Non-Carcinogen	Non-Carcinogen	Non-Carcinogen	Non-Mutagen	Non-Toxic
MolPort-005-037-492	Non-Carcinogen	Non-Carcinogen	Non-Carcinogen	Non-Carcinogen	Non-Carcinogen	Non-Carcinogen	Non-Carcinogen	Non-Carcinogen	Non-Carcinogen	Non-Mutagen	Non-Toxic
MolPort-046-192-370	Non-Carcinogen	Non-Carcinogen	Non-Carcinogen	Non-Carcinogen	Non-Carcinogen	Non-Carcinogen	Non-Carcinogen	Non-Carcinogen	Non-Carcinogen	Non-Mutagen	Non-Toxic
MolPort-005-012-831	Non-Carcinogen	Non-Carcinogen	Non-Carcinogen	Non-Carcinogen	Non-Carcinogen	Non-Carcinogen	Non-Carcinogen	Non-Carcinogen	Non-Carcinogen	Non-Mutagen	Non-Toxic
MolPort-010-710-830	Non-Carcinogen	Non-Carcinogen	Non-Carcinogen	Non-Carcinogen	Non-Carcinogen	Non-Carcinogen	Non-Carcinogen	Non-Carcinogen	Non-Carcinogen	Non-Mutagen	Non-Toxic
MolPort-051-592-148	Non-Carcinogen	Non-Carcinogen	Non-Carcinogen	Non-Carcinogen	Non-Carcinogen	Non-Carcinogen	Non-Carcinogen	Non-Carcinogen	Non-Carcinogen	Non-Mutagen	Non-Toxic
MolPort-005-074-134	Non-Carcinogen	Non-Carcinogen	Non-Carcinogen	Non-Carcinogen	Non-Carcinogen	Non-Carcinogen	Non-Carcinogen	Non-Carcinogen	Non-Carcinogen	Non-Mutagen	Non-Toxic
MolPort-008-283-589	Non-Carcinogen	Non-Carcinogen	Non-Carcinogen	Non-Carcinogen	Non-Carcinogen	Non-Carcinogen	Non-Carcinogen	Non-Carcinogen	Non-Carcinogen	Non-Mutagen	Non-Toxic
MolPort-044-352-089	Non-Carcinogen	Non-Carcinogen	Non-Carcinogen	Non-Carcinogen	Non-Carcinogen	Non-Carcinogen	Non-Carcinogen	Non-Carcinogen	Non-Carcinogen	Non-Mutagen	Non-Toxic
MolPort-005-063-722	Non-Carcinogen	Non-Carcinogen	Non-Carcinogen	Non-Carcinogen	Non-Carcinogen	Non-Carcinogen	Non-Carcinogen	Non-Carcinogen	Non-Carcinogen	Non-Mutagen	Non-Toxic
MolPort-007-674-419	Non-Carcinogen	Non-Carcinogen	Non-Carcinogen	Non-Carcinogen	Non-Carcinogen	Non-Carcinogen	Non-Carcinogen	Non-Carcinogen	Non-Carcinogen	Non-Mutagen	Non-Toxic
MolPort-039-104-467	Non-Carcinogen	Non-Carcinogen	Non-Carcinogen	Non-Carcinogen	Non-Carcinogen	Non-Carcinogen	Non-Carcinogen	Non-Carcinogen	Non-Carcinogen	Non-Mutagen	Non-Toxic
MolPort-009-706-524	Non-Carcinogen	Non-Carcinogen	Non-Carcinogen	Non-Carcinogen	Non-Carcinogen	Non-Carcinogen	Non-Carcinogen	Non-Carcinogen	Non-Carcinogen	Non-Mutagen	Non-Toxic
MolPort-005-012-554	Non-Carcinogen	Non-Carcinogen	Non-Carcinogen	Non-Carcinogen	Non-Carcinogen	Non-Carcinogen	Non-Carcinogen	Non-Carcinogen	Non-Carcinogen	Non-Mutagen	Non-Toxic
MolPort-002-663-534	Non-Carcinogen	Non-Carcinogen	Non-Carcinogen	Non-Carcinogen	Non-Carcinogen	Non-Carcinogen	Non-Carcinogen	Non-Carcinogen	Non-Carcinogen	Non-Mutagen	Non-Toxic
MolPort-010-710-829	Non-Carcinogen	Non-Carcinogen	Non-Carcinogen	Non-Carcinogen	Non-Carcinogen	Non-Carcinogen	Non-Carcinogen	Non-Carcinogen	Non-Carcinogen	Non-Mutagen	Non-Toxic
MolPort-005-069-159	Non-Carcinogen	Non-Carcinogen	Non-Carcinogen	Non-Carcinogen	Non-Carcinogen	Non-Carcinogen	Non-Carcinogen	Non-Carcinogen	Non-Carcinogen	Non-Mutagen	Non-Toxic
MolPort-044-313-981	Non-Carcinogen	Non-Carcinogen	Non-Carcinogen	Non-Carcinogen	Non-Carcinogen	Non-Carcinogen	Non-Carcinogen	Non-Carcinogen	Non-Carcinogen	Non-Mutagen	Non-Toxic
MolPort-016-668-431	Non-Carcinogen	Non-Carcinogen	Non-Carcinogen	Non-Carcinogen	Non-Carcinogen	Non-Carcinogen	Non-Carcinogen	Non-Carcinogen	Non-Carcinogen	Non-Mutagen	Non-Toxic
MolPort-000-850-276	Non-Carcinogen	Non-Carcinogen	Non-Carcinogen	Non-Carcinogen	Non-Carcinogen	Non-Carcinogen	Non-Carcinogen	Non-Carcinogen	Non-Carcinogen	Non-Mutagen	Non-Toxic
MolPort-002-649-964	Non-Carcinogen	Non-Carcinogen	Non-Carcinogen	Non-Carcinogen	Non-Carcinogen	Non-Carcinogen	Non-Carcinogen	Non-Carcinogen	Non-Carcinogen	Non-Mutagen	Non-Toxic
MolPort-005-075-173	Non-Carcinogen	Non-Carcinogen	Non-Carcinogen	Non-Carcinogen	Non-Carcinogen	Non-Carcinogen	Non-Carcinogen	Non-Carcinogen	Non-Carcinogen	Non-Mutagen	Non-Toxic
MolPort-046-601-847	Non-Carcinogen	Non-Carcinogen	Non-Carcinogen	Non-Carcinogen	Non-Carcinogen	Non-Carcinogen	Non-Carcinogen	Non-Carcinogen	Non-Carcinogen	Non-Mutagen	Non-Toxic
MolPort-000-791-076	Non-Carcinogen	Non-Carcinogen	Non-Carcinogen	Non-Carcinogen	Non-Carcinogen	Non-Carcinogen	Non-Carcinogen	Non-Carcinogen	Non-Carcinogen	Non-Mutagen	Non-Toxic
MolPort-003-122-319	Non-Carcinogen	Non-Carcinogen	Non-Carcinogen	Non-Carcinogen	Non-Carcinogen	Non-Carcinogen	Non-Carcinogen	Non-Carcinogen	Non-Carcinogen	Non-Mutagen	Non-Toxic
MolPort-005-033-956	Non-Carcinogen	Non-Carcinogen	Non-Carcinogen	Non-Carcinogen	Non-Carcinogen	Non-Carcinogen	Non-Carcinogen	Non-Carcinogen	Non-Carcinogen	Non-Mutagen	Non-Toxic

COMPOUNDS	PARAMETERS										
	NTP Mouse		NTP Rat		Mouse FDA		Rat FDA		WOE	AMES	DTP
	Female	Male	Female	Male	Female	Male	Female	Male			
MolPort-010-710-806	Non-Carcinogen	Non-Carcinogen	Non-Carcinogen	Non-Carcinogen	Non-Carcinogen	Non-Carcinogen	Non-Carcinogen	Non-Carcinogen	Non-Carcinogen	Non-Mutagen	Non-Toxic
MolPort-002-861-891	Non-Carcinogen	Non-Carcinogen	Non-Carcinogen	Non-Carcinogen	Non-Carcinogen	Non-Carcinogen	Non-Carcinogen	Non-Carcinogen	Non-Carcinogen	Non-Mutagen	Non-Toxic
MolPort-019-720-091	Non-Carcinogen	Non-Carcinogen	Non-Carcinogen	Non-Carcinogen	Non-Carcinogen	Non-Carcinogen	Non-Carcinogen	Non-Carcinogen	Non-Carcinogen	Non-Mutagen	Non-Toxic
MolPort-010-663-222	Non-Carcinogen	Non-Carcinogen	Non-Carcinogen	Non-Carcinogen	Non-Carcinogen	Non-Carcinogen	Non-Carcinogen	Non-Carcinogen	Non-Carcinogen	Non-Mutagen	Non-Toxic
MolPort-010-911-006	Non-Carcinogen	Non-Carcinogen	Non-Carcinogen	Non-Carcinogen	Non-Carcinogen	Non-Carcinogen	Non-Carcinogen	Non-Carcinogen	Non-Carcinogen	Non-Mutagen	Non-Toxic

Table S6: Molecular docking of the compounds selected in the toxicological screening from 0Y5, the Top5 are those mentioned in the study

ID	COMPOUNDS	BINDING ENERGY(Kcal/mol)
**4GAV01**	Molport-010-710-830	-9.362
**Template**	0Y5	-9.147
**4GAV02**	Molport-010-710-806	-9.066
**4GAV03**	Molport-010-710-829	-8.891
**4GAV04**	Molport-044-309-641	-8.871
**4GAV05**	Molport-044-313-041	-8.857
4GAV06	Molport-044-313-981	-8.782
4GAV07	Molport-000-791-076	-8.699
4GAV08	Molport-016-668-568	-8.627
4GAV09	Molport-007-674-419	-8.549
4GAV10	Molport-044-313-085	-8.462
4GAV11	Molport-046-839-431	-8.449
4GAV12	Molport-010-663-222	-8.351
4GAV13	Molport-044-352-089	-8.350
4GAV14	Molport-008-329-796	-8.295
4GAV15	Molport-009-706-524	-8.291
4GAV16	Molport-005-074-134	-8.265
4GAV17	Molport-005-012-554	-8.256
4GAV18	Molport-005-075-173	-8.189
4GAV19	Molport-005-063-722	-8.152
4GAV20	Molport-005-012-831	-8.146
4GAV21	Molport-000-820-749	-8.137
4GAV22	Molport-046-601-735	-8.132
4GAV23	Molport-002-649-964	-8.107
4GAV24	Molport-003-122-319	-8.102
4GAV25	Molport-005-033-956	-8.094
4GAV26	Molport-010-911-006	-8.073
4GAV27	Molport-051-592-148	-8.067
4GAV28	Molport-003-125-711	-7.966
4GAV29	Molport-028-854-465	-7.953

ID	COMPOUNDS	BINDING ENERGY(Kcal/mol)
4GAV30	Molport-046-601-847	-7.881
4GAV31	Molport-042-617-134	-7.870
4GAV32	Molport-002-663-534	-7.778
4GAV33	Molport-002-861-891	-7.740
4GAV34	Molport-046-177-951	-7.729
4GAV35	Molport-005-037-492	-7.719
4GAV36	Molport-008-283-589	-7.710
4GAV37	Molport-019-720-091	-7.705
4GAV38	Molport-019-720-501	-7.700
4GAV39	Molport-019-720-425	-7.694
4GAV40	Molport-044-312-579	-7.557
4GAV41	Molport-005-069-159	-7.537
4GAV42	Molport-005-043-958	-7.514
4GAV43	Molport-039-104-467	-7.507
4GAV44	Molport-016-668-431	-7.471
4GAV45	Molport-000-850-276	-7.230
4GAV46	Molport-046-192-370	-7.177

Figure S2: Interaction with amino acid residues of the Top5 compounds screened from 0Y5

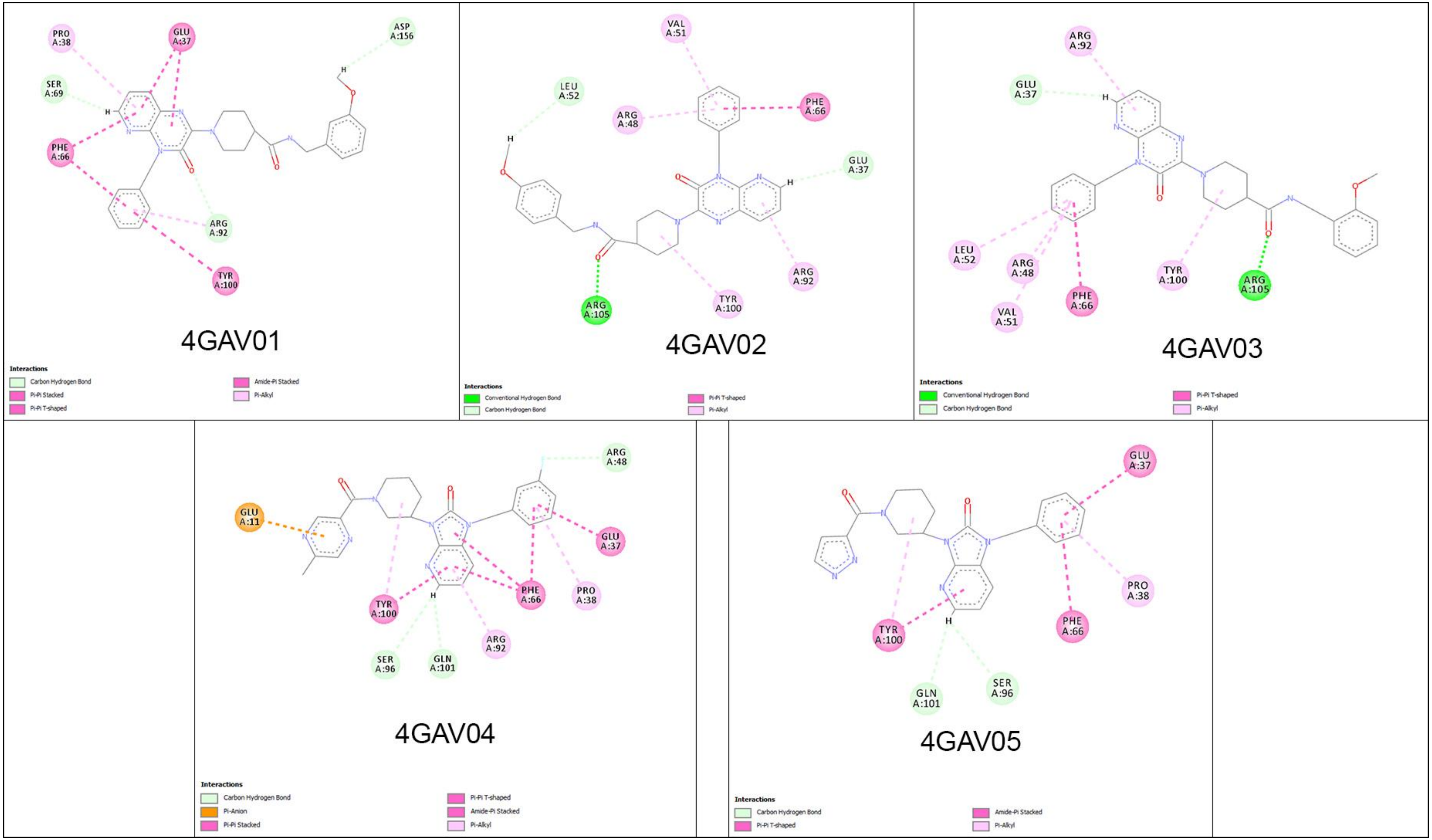

Supplement: Supplementary file 1 [file ijms-27-02736-s001.zip › Supplementary_AVS.pdf]
