# Supplementary material for: Identification of Potential Candidates with Antimicrobial Activity Against Antibiotic-Resistant Staphylococcus aureus Strains: A Hierarchical Bioinformatics Approach
Source: Int J Mol Sci. 2026 Mar 17;27(6):2736. doi: 10.3390/ijms27062736 (PMC13026990; doi:10.3390/ijms27062736)
Supplement: Supplementary file 1 [file ijms-27-02736-s001.zip › Top5_0Y5_ToxicityReports.pdf]

Molecule

TOPKAT_Aerobic_Biodegradability

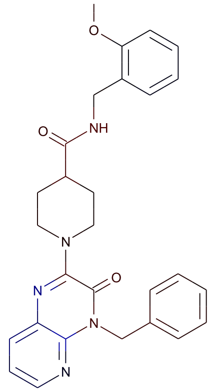

$C_{28}H_{29}N_5O_3$
Molecular Weight: 483.56156
ALogP: 2.942
Rotatable Bonds: 7
Acceptors: 6
Donors: 1

Structural Similar Compounds

Name	Rhodamine_B	2,5-Cyclohexadiene-1,4-dione,_bis(O-benzoyloxime)	9,10-Anthracenedione,_1-amino-2-(4-bromophenoxy)-4-hydroxy-
Structure	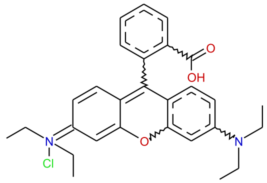	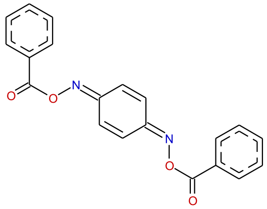	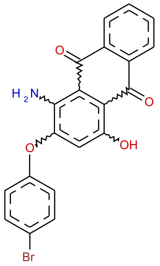
Actual Endpoint	Non-Degradable	Non-Degradable	Non-Degradable
Predicted Endpoint	Non-Degradable	Non-Degradable	Non-Degradable
Distance	0.796	0.822	0.841
Reference	Environmental Toxicology & Chemistry 18(9), 1763-1768, 1999.	Environmental Toxicology & Chemistry 18(9), 1763-1768, 1999.	Environmental Toxicology & Chemistry 18(9), 1763-1768, 1999.

Model Prediction

Prediction: Degradable
Probability: 0.499
Enrichment: 1.14
Bayesian Score: 0.185
Mahalanobis Distance: 17.2
Mahalanobis Distance p-value: 6.85e-020

Prediction: Positive if the Bayesian score is above the estimated best cutoff value from minimizing the false positive and false negative rate.
Probability: The esimated probability that the sample is in the positive category. This assumes that the Bayesian score follows a normal distribution and is different from the prediction using a cutoff.
Enrichment: An estimate of enrichment, that is, the increased likelihood (versus random) of this sample being in the category.
Bayesian Score: The standard Laplacian-modified Bayesian score.
Mahalanobis Distance: The Mahalanobis distance (MD) is the distance to the center of the training data. The larger the MD, the less trustworthy the prediction.
Mahalanobis Distance p-value: The p-value gives the fraction of training data with an MD greater than or equal to the one for the given sample, assuming normally distributed data. The smaller the p-value, the less trustworthy the prediciton. For highly non-normal X properties (e.g., fingerprints), the MD p-value is wildly inaccurate.

Model Applicability

Unknown features are fingerprint features in the query molecule, but not found or appearing too infrequently in the training set.

1. OPS PC6 out of range. Value: 6.7424. Training min, max, SD, explained variance: -5.6927, 6.627, 1.955, 0.0439.
2. OPS PC14 out of range. Value: -5.657. Training min, max, SD, explained variance: -4.3372, 4.2674, 1.364, 0.0214.
3. OPS PC16 out of range. Value: 5.7753. Training min, max, SD, explained variance: -3.0094, 4.2513, 1.289, 0.0191.

Feature Contribution

Top features for positive contribution				
**Fingerprint**	**Bit/Smiles**	**Feature Structure**	**Score**	**Degradable in training set**

SCFP_12	1256995004	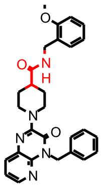<br>[*]NC(=O)C([*])[*]	0.54	23 out of 32
SCFP_12	-1272798659	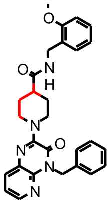<br>[*]CCC([*])[*]	0.518	160 out of 234
SCFP_12	1311071855	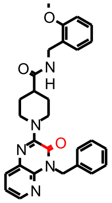<br>[*]C(=O)[*]	0.461	173 out of 268

Top Features for negative contribution

Fingerprint	Bit/Smiles	Feature Structure	Score	Degradable in training set
SCFP_12	6	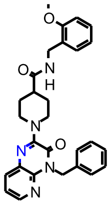<br>[*]N=[*]	-1.02	2 out of 18
SCFP_12	-1377141613	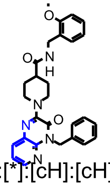<br>[*][c]1:[*]:[cH]:[cH]:[cH]:[c]:1N=[*]	-0.964	0 out of 4

SCFP_12	1851000357	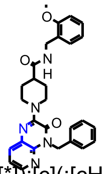 [*][c](:[*]):[c](:[cH]:[*])N=[*]	-0.964	0 out of 4

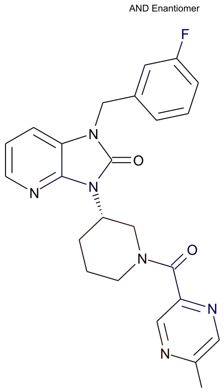

$C_{24}H_{23}FN_6O_2$
Molecular Weight: 446.47682
ALogP: 2.244
Rotatable Bonds: 4
Acceptors: 5
Donors: 0

Structural Similar Compounds

Name	Tetrachlorvinphos	Stirofos	2,5-Cyclohexadiene-1,4-dione,_bis(O-benzoyloxime)
Structure	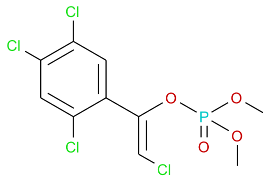	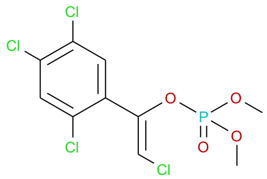	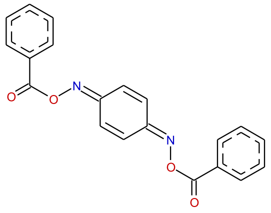
Actual Endpoint	Non-Degradable	Non-Degradable	Non-Degradable
Predicted Endpoint	Non-Degradable	Non-Degradable	Non-Degradable
Distance	0.691	0.691	0.731
Reference	Environmental Toxicology & Chemistry 18(9), 1763-1768, 1999.	Environmental Toxicology & Chemistry 18(9), 1763-1768, 1999.	Environmental Toxicology & Chemistry 18(9), 1763-1768, 1999.

Model Prediction

Prediction: Non-Degradable
Probability: 0.36
Enrichment: 0.825
Bayesian Score: -2.82
Mahalanobis Distance: 12.8
Mahalanobis Distance p-value: 5.55e-006

Prediction: Positive if the Bayesian score is above the estimated best cutoff value from minimizing the false positive and false negative rate.
Probability: The esimated probability that the sample is in the positive category. This assumes that the Bayesian score follows a normal distribution and is different from the prediction using a cutoff.
Enrichment: An estimate of enrichment, that is, the increased likelihood (versus random) of this sample being in the category.
Bayesian Score: The standard Laplacian-modified Bayesian score.
Mahalanobis Distance: The Mahalanobis distance (MD) is the distance to the center of the training data. The larger the MD, the less trustworthy the prediction.
Mahalanobis Distance p-value: The p-value gives the fraction of training data with an MD greater than or equal to the one for the given sample, assuming normally distributed data. The smaller the p-value, the less trustworthy the prediciton. For highly non-normal X properties (e.g., fingerprints), the MD p-value is wildly inaccurate.

Model Applicability

Unknown features are fingerprint features in the query molecule, but not found or appearing too infrequently in the training set.

1. All properties and OPS components are within expected ranges.

Feature Contribution

Top features for positive contribution

Fingerprint	Bit/Smiles	Feature Structure	Score	Degradable in training set
SCFP_12	-1343150366	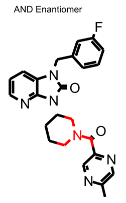 [*]CN(C[*])C(=[*])[*]	0.561	10 out of 13

SCFP_12	-1272798659	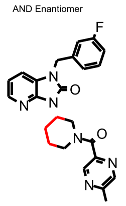<br>[*]CCC([*])[*]	0.518	160 out of 234
SCFP_12	1194442465	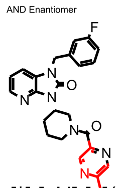<br>[*][c]1:[*]:[cH]:[c](C):n:[cH]:1	0.504	2 out of 2

Top Features for negative contribution

Fingerprint	Bit/Smiles	Feature Structure	Score	Degradable in training set
SCFP_12	26	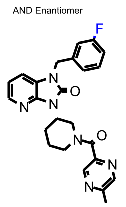<br>[*]F	-1.62	0 out of 10
SCFP_12	-827073191	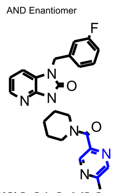<br>[*]C(=[*])[c]1:[cH]:[*]:[c]([*]):[cH]:n:1	-0.594	0 out of 2
SCFP_12	1256786467	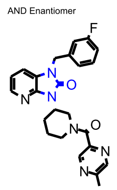<br>[*]N1[*]:[*]N([*])C1=O	-0.541	1 out of 6

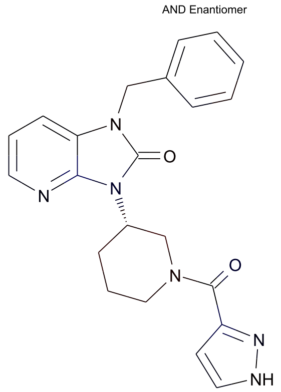

$C_{22}H_{22}N_6O_2$
Molecular Weight: 402.44907
ALogP: 2.501
Rotatable Bonds: 4
Acceptors: 4
Donors: 1

Structural Similar Compounds

Name	Benzeneacetic_acid,_4-chloro-.alpha.-(4-chlorophenyl)-.alpha.-hydroxy-,_ethyl_ester	Bicyclo_2.2.1_hept-5-ene-2,3-dicarboxylic_acid,_1,4,5,6,7,7-hexachloro-	Stirofos
Structure	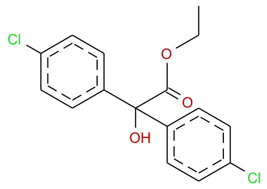	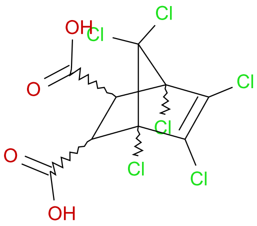	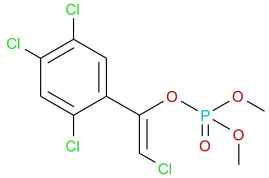
Actual Endpoint	Non-Degradable	Non-Degradable	Non-Degradable
Predicted Endpoint	Non-Degradable	Non-Degradable	Non-Degradable
Distance	0.704	0.739	0.749
Reference	Environmental Toxicology & Chemistry 18(9), 1763-1768, 1999.	Environmental Toxicology & Chemistry 18(9), 1763-1768, 1999.	Environmental Toxicology & Chemistry 18(9), 1763-1768, 1999.

Model Prediction

Prediction: Non-Degradable
Probability: 0.479
Enrichment: 1.1
Bayesian Score: -0.234
Mahalanobis Distance: 12
Mahalanobis Distance p-value: 0.000365

Prediction: Positive if the Bayesian score is above the estimated best cutoff value from minimizing the false positive and false negative rate.
Probability: The esimated probability that the sample is in the positive category. This assumes that the Bayesian score follows a normal distribution and is different from the prediction using a cutoff.
Enrichment: An estimate of enrichment, that is, the increased likelihood (versus random) of this sample being in the category.
Bayesian Score: The standard Laplacian-modified Bayesian score.
Mahalanobis Distance: The Mahalanobis distance (MD) is the distance to the center of the training data. The larger the MD, the less trustworthy the prediction.
Mahalanobis Distance p-value: The p-value gives the fraction of training data with an MD greater than or equal to the one for the given sample, assuming normally distributed data. The smaller the p-value, the less trustworthy the prediciton. For highly non-normal X properties (e.g., fingerprints), the MD p-value is wildly inaccurate.

Model Applicability

Unknown features are fingerprint features in the query molecule, but not found or appearing too infrequently in the training set.

1. All properties and OPS components are within expected ranges.

Feature Contribution

Top features for positive contribution

Fingerprint	Bit/Smiles	Feature Structure	Score	Degradable in training set
SCFP_12	-1343150366	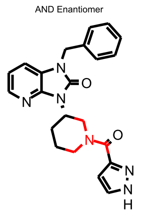 [*]CN(C[*])C(=[*])[*]	0.561	10 out of 13

SCFP_12	-1272798659	AND Enantiomer 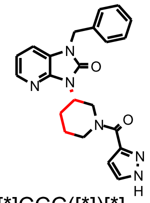 [*]CCC([*])[*]	0.518	160 out of 234
SCFP_12	1311071855	AND Enantiomer 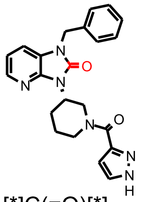 [*]C(=O)[*]	0.461	173 out of 268
**Top Features for negative contribution**				
**Fingerprint**	**Bit/Smiles**	**Feature Structure**	**Score**	**Degradable in training set**
SCFP_12	1257084377	AND Enantiomer 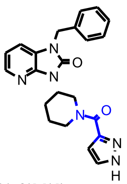 [*]N([*])C(=O)[c](:[*]):[*]	-0.541	1 out of 6
SCFP_12	1256786467	AND Enantiomer 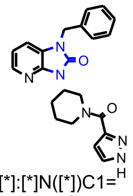 [*]N1[*]:[*]N([*])C1=O	-0.541	1 out of 6
SCFP_12	149003983	AND Enantiomer 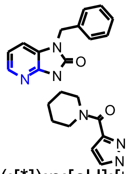 [*][c](:[*]):n:[cH]:[*]	-0.497	8 out of 34

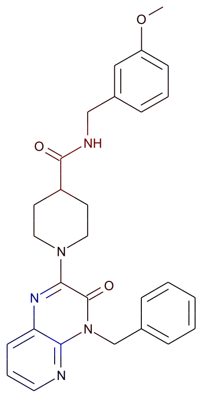

$C_{28}H_{29}N_5O_3$
Molecular Weight: 483.56156
ALogP: 2.942
Rotatable Bonds: 7
Acceptors: 6
Donors: 1

Structural Similar Compounds

Name	Rhodamine_B	2,5-Cyclohexadiene-1,4-dione,_bis(O-benzoyloxime)	9,10-Anthracenedione,_1-amino-2-(4-bromophenoxy)-4-hydroxy-
Structure	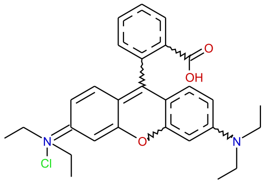	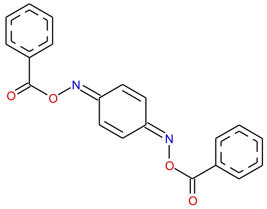	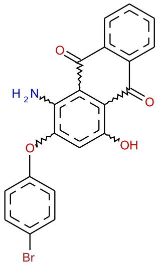
Actual Endpoint	Non-Degradable	Non-Degradable	Non-Degradable
Predicted Endpoint	Non-Degradable	Non-Degradable	Non-Degradable
Distance	0.796	0.823	0.841
Reference	Environmental Toxicology & Chemistry 18(9), 1763-1768, 1999.	Environmental Toxicology & Chemistry 18(9), 1763-1768, 1999.	Environmental Toxicology & Chemistry 18(9), 1763-1768, 1999.

Model Prediction

Prediction: Degradable
Probability: 0.507
Enrichment: 1.16
Bayesian Score: 0.357
Mahalanobis Distance: 16.4
Mahalanobis Distance p-value: 9.94e-017

Prediction: Positive if the Bayesian score is above the estimated best cutoff value from minimizing the false positive and false negative rate.
Probability: The esimated probability that the sample is in the positive category. This assumes that the Bayesian score follows a normal distribution and is different from the prediction using a cutoff.
Enrichment: An estimate of enrichment, that is, the increased likelihood (versus random) of this sample being in the category.
Bayesian Score: The standard Laplacian-modified Bayesian score.
Mahalanobis Distance: The Mahalanobis distance (MD) is the distance to the center of the training data. The larger the MD, the less trustworthy the prediction.
Mahalanobis Distance p-value: The p-value gives the fraction of training data with an MD greater than or equal to the one for the given sample, assuming normally distributed data. The smaller the p-value, the less trustworthy the prediciton. For highly non-normal X properties (e.g., fingerprints), the MD p-value is wildly inaccurate.

Model Applicability

Unknown features are fingerprint features in the query molecule, but not found or appearing too infrequently in the training set.

1. OPS PC14 out of range. Value: -5.1447. Training min, max, SD, explained variance: -4.3372, 4.2674, 1.364, 0.0214.
2. OPS PC16 out of range. Value: 5.2982. Training min, max, SD, explained variance: -3.0094, 4.2513, 1.289, 0.0191.

Feature Contribution

Top features for positive contribution

Fingerprint	Bit/Smiles	Feature Structure	Score	Degradable in training set

SCFP_12	1256995004	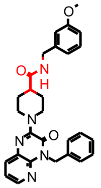 [*]NC(=O)C([*])[*]	0.54	23 out of 32
SCFP_12	-1272798659	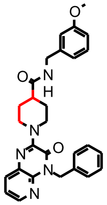 [*]CCC([*])[*]	0.518	160 out of 234
SCFP_12	1311071855	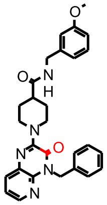 [*]C(=O)[*]	0.461	173 out of 268

Top Features for negative contribution

**Fingerprint**	**Bit/Smiles**	**Feature Structure**	**Score**	**Degradable in training set**
SCFP_12	6	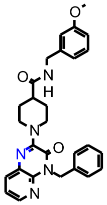 [*]N=[*]	-1.02	2 out of 18
SCFP_12	-1377141613	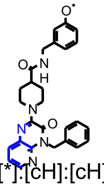 [*][c]1:[*]:[cH]:[cH]:[cH]:[c]:1N=[*]	-0.964	0 out of 4

SCFP_12	1851000357	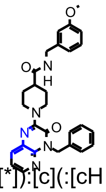 [*][c](:[*]):[c](:[cH]:[*])N=[*]	-0.964	0 out of 4

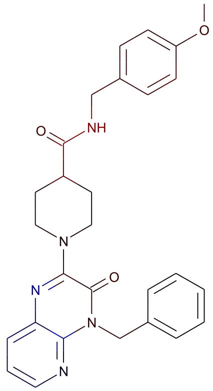

$C_{28}H_{29}N_5O_3$
Molecular Weight: 483.56156
ALogP: 2.942
Rotatable Bonds: 7
Acceptors: 6
Donors: 1

Structural Similar Compounds

Name	Rhodamine_B	2,5-Cyclohexadiene-1,4-dione,_bis(O-benzoyloxime)	9,10-Anthracenedione,_1-amino-2-(4-bromophenoxy)-4-hydroxy-
Structure	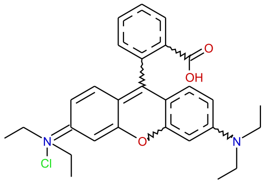	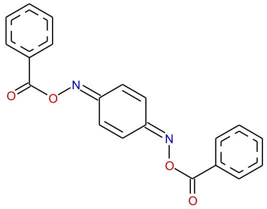	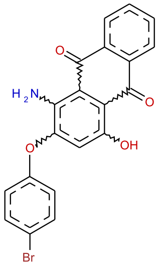
Actual Endpoint	Non-Degradable	Non-Degradable	Non-Degradable
Predicted Endpoint	Non-Degradable	Non-Degradable	Non-Degradable
Distance	0.796	0.823	0.841
Reference	Environmental Toxicology & Chemistry 18(9), 1763-1768, 1999.	Environmental Toxicology & Chemistry 18(9), 1763-1768, 1999.	Environmental Toxicology & Chemistry 18(9), 1763-1768, 1999.

Model Prediction

Prediction: Degradable
Probability: 0.548
Enrichment: 1.26
Bayesian Score: 1.2
Mahalanobis Distance: 16.4
Mahalanobis Distance p-value: 9.94e-017

Prediction: Positive if the Bayesian score is above the estimated best cutoff value from minimizing the false positive and false negative rate.
Probability: The esimated probability that the sample is in the positive category. This assumes that the Bayesian score follows a normal distribution and is different from the prediction using a cutoff.
Enrichment: An estimate of enrichment, that is, the increased likelihood (versus random) of this sample being in the category.
Bayesian Score: The standard Laplacian-modified Bayesian score.
Mahalanobis Distance: The Mahalanobis distance (MD) is the distance to the center of the training data. The larger the MD, the less trustworthy the prediction.
Mahalanobis Distance p-value: The p-value gives the fraction of training data with an MD greater than or equal to the one for the given sample, assuming normally distributed data. The smaller the p-value, the less trustworthy the prediciton. For highly non-normal X properties (e.g., fingerprints), the MD p-value is wildly inaccurate.

Model Applicability

Unknown features are fingerprint features in the query molecule, but not found or appearing too infrequently in the training set.

1. OPS PC14 out of range. Value: -5.1447. Training min, max, SD, explained variance: -4.3372, 4.2674, 1.364, 0.0214.
2. OPS PC16 out of range. Value: 5.2982. Training min, max, SD, explained variance: -3.0094, 4.2513, 1.289, 0.0191.

Feature Contribution

Top features for positive contribution

Fingerprint	Bit/Smiles	Feature Structure	Score	Degradable in training set

SCFP_12	1256995004	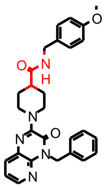 [*]NC(=O)C([*])[*]	0.54	23 out of 32
SCFP_12	-1272798659	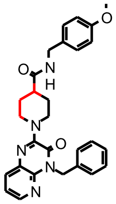 [*]CCC([*])[*]	0.518	160 out of 234
SCFP_12	1311071855	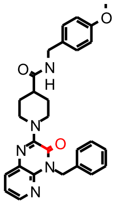 [*]C(=O)[*]	0.461	173 out of 268

Top Features for negative contribution

Fingerprint	Bit/Smiles	Feature Structure	Score	Degradable in training set
SCFP_12	6	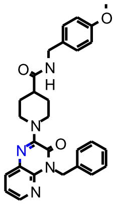 [*]N=[*]	-1.02	2 out of 18
SCFP_12	1851000357	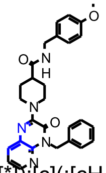 [*][c](:[*]):[c](:[cH]:[*])N=[*]	-0.964	0 out of 4

SCFP_12	-1377141613	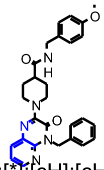 [*][c]1:[*]:[cH]:[cH]:[cH]:[c]:1N=[*]	-0.964	0 out of 4

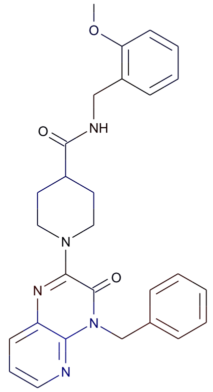

$C_{28}H_{29}N_5O_3$
Molecular Weight: 483.56156
ALogP: 2.942
Rotatable Bonds: 7
Acceptors: 6
Donors: 1

Model Prediction

Prediction: Non-Mutagen
Probability: 0.622
Enrichment: 1.11
Bayesian Score: -4.42
Mahalanobis Distance: 15.1
Mahalanobis Distance p-value: 7.24e-013

Prediction: Positive if the Bayesian score is above the estimated best cutoff value from minimizing the false positive and false negative rate.
Probability: The esimated probability that the sample is in the positive category. This assumes that the Bayesian score follows a normal distribution and is different from the prediction using a cutoff.
Enrichment: An estimate of enrichment, that is, the increased likelihood (versus random) of this sample being in the category.
Bayesian Score: The standard Laplacian-modified Bayesian score.
Mahalanobis Distance: The Mahalanobis distance (MD) is the distance to the center of the training data. The larger the MD, the less trustworthy the prediction.
Mahalanobis Distance p-value: The p-value gives the fraction of training data with an MD greater than or equal to the one for the given sample, assuming normally distributed data. The smaller the p-value, the less trustworthy the prediciton. For highly non-normal X properties (e.g., fingerprints), the MD p-value is wildly inaccurate.

Structural Similar Compounds

Name	83621-06-1	MORICIZINE	DILTIAZEM
Structure	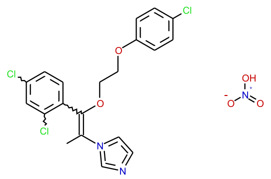	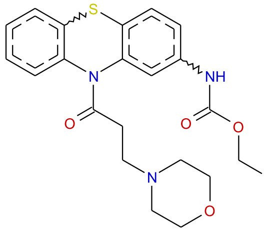	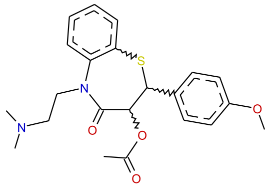
Actual Endpoint	Non-Mutagen	Non-Mutagen	Non-Mutagen
Predicted Endpoint	Non-Mutagen	Non-Mutagen	Non-Mutagen
Distance	0.575	0.580	0.593
Reference	Kazius et. al., J. Med. Chem. (2005) 48, 312-320	PDR 1994	PDR 1994

Model Applicability

Unknown features are fingerprint features in the query molecule, but not found or appearing too infrequently in the training set.

1. OPS PC35 out of range. Value: 6.1618. Training min, max, SD, explained variance: -6.6231, 5.2872, 0.925, 0.0091.

Feature Contribution

Top features for positive contribution				
**Fingerprint**	**Bit/Smiles**	**Feature Structure**	**Score**	**Mutagen in training set**
SCFP_12	1575781215	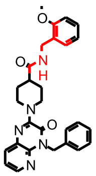 [*]C(=[*])NC[c](:[cH]:[*]):[c]([*]):[*]	0.517	16 out of 16

SCFP_12	-1211234921	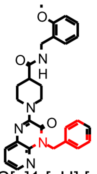 [*]N([*])C[c]1:[cH]:[cH]:[*]:[cH]:[cH]:1	0.514	15 out of 15
SCFP_12	1632957125	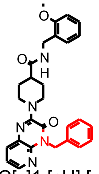 [*]N([*])C[c]1:[cH]:[cH]:[cH]:[cH]:[cH]:1	0.487	9 out of 9

Top Features for negative contribution

Fingerprint	Bit/Smiles	Feature Structure	Score	Mutagen in training set
SCFP_12	1190896200	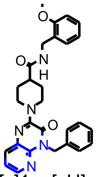 [*]N([*])[c]1:n:[cH]:[cH]:[*]:[c]:1[*]	-1.49	0 out of 6
SCFP_12	1205795299	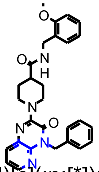 [*]N([*])[c](:n:[*]):[c]([*]):[*]	-1.22	2 out of 16
SCFP_12	-1043310069	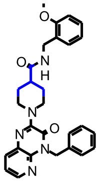 [*]CC(C[*])C(=[*])[*]	-0.989	44 out of 210

#UNDEFINED

TOPKAT_Ames_Mutagenicity

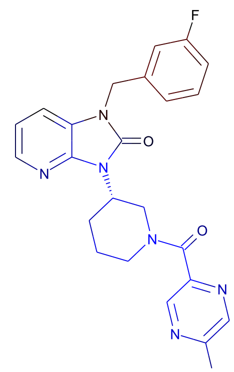

$C_{24}H_{23}FN_6O_2$

Molecular Weight: 446.47682

ALogP: 2.244

Rotatable Bonds: 4

Acceptors: 5

Donors: 0

Structural Similar Compounds

Name	105149-00-6	81840-15-5	BUSPIRONE HCL
Structure	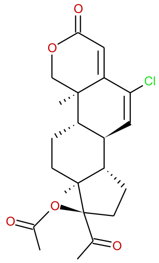	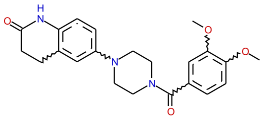	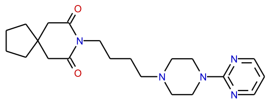
Actual Endpoint	Non-Mutagen	Non-Mutagen	Non-Mutagen
Predicted Endpoint	Non-Mutagen	Non-Mutagen	Non-Mutagen
Distance	0.589	0.595	0.613
Reference	Kazius et. al., J. Med. Chem. (2005) 48, 312-320	Kazius et. al., J. Med. Chem. (2005) 48, 312-320	PDR 1994

Model Applicability

Unknown features are fingerprint features in the query molecule, but not found or appearing too infrequently in the training set.

1. All properties and OPS components are within expected ranges.

Model Prediction

Prediction: Non-Mutagen

Probability: 0.246

Enrichment: 0.44

Bayesian Score: -13

Mahalanobis Distance: 13.4

Mahalanobis Distance p-value: 2.66e-007

Prediction: Positive if the Bayesian score is above the estimated best cutoff value from minimizing the false positive and false negative rate.
Probability: The esimated probability that the sample is in the positive category. This assumes that the Bayesian score follows a normal distribution and is different from the prediction using a cutoff.
Enrichment: An estimate of enrichment, that is, the increased likelihood (versus random) of this sample being in the category.
Bayesian Score: The standard Laplacian-modified Bayesian score.
Mahalanobis Distance: The Mahalanobis distance (MD) is the distance to the center of the training data. The larger the MD, the less trustworthy the prediction.
Mahalanobis Distance p-value: The p-value gives the fraction of training data with an MD greater than or equal to the one for the given sample, assuming normally distributed data. The smaller the p-value, the less trustworthy the prediciton. For highly non-normal X properties (e.g., fingerprints), the MD p-value is wildly inaccurate.

Feature Contribution

Top features for positive contribution

Fingerprint	Bit/Smiles	Feature Structure	Score	Mutagen in training set
SCFP_12	-1211234921	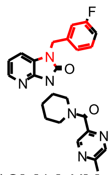 [*]N([*])C[c]1:[cH]:[cH]:[*]:[cH]:[cH]:1	0.514	15 out of 15

SCFP_12	1632957125	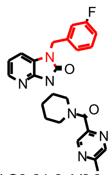 [*]N([*])C[c]1:[cH]:[cH]:[cH]:[cH]:[cH]:1	0.487	9 out of 9
SCFP_12	-971326317	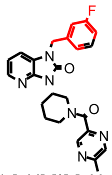 [*]C[c]1:[cH]:[*]:[cH]:[c](F):[cH]:1	0.337	2 out of 2

Top Features for negative contribution

Fingerprint	Bit/Smiles	Feature Structure	Score	Mutagen in training set
SCFP_12	1190896200	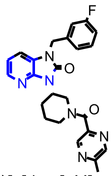 [*]N([*])[c]1:n:[cH]:[cH]:[*]:[c]:1[*]	-1.49	0 out of 6
SCFP_12	1205795299	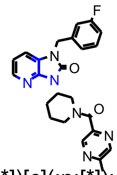 [*]N([*])[c](:n:[*]):[c]([*]):[*]	-1.22	2 out of 16
SCFP_12	306578635	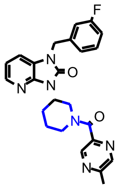 [*]C(=[*])N1C[*]CCC1	-1.09	3 out of 19

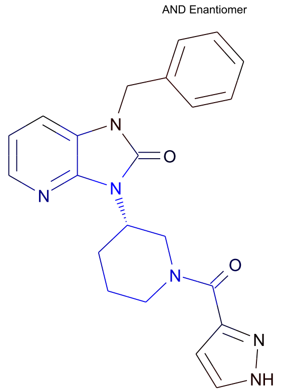

$C_{22}H_{22}N_6O_2$
Molecular Weight: 402.44907
ALogP: 2.501
Rotatable Bonds: 4
Acceptors: 4
Donors: 1

Model Prediction

Prediction: Non-Mutagen
Probability: 0.463
Enrichment: 0.829
Bayesian Score: -8.34
Mahalanobis Distance: 11.9
Mahalanobis Distance p-value: 0.00118

Prediction: Positive if the Bayesian score is above the estimated best cutoff value from minimizing the false positive and false negative rate.
Probability: The esimated probability that the sample is in the positive category. This assumes that the Bayesian score follows a normal distribution and is different from the prediction using a cutoff.
Enrichment: An estimate of enrichment, that is, the increased likelihood (versus random) of this sample being in the category.
Bayesian Score: The standard Laplacian-modified Bayesian score.
Mahalanobis Distance: The Mahalanobis distance (MD) is the distance to the center of the training data. The larger the MD, the less trustworthy the prediction.
Mahalanobis Distance p-value: The p-value gives the fraction of training data with an MD greater than or equal to the one for the given sample, assuming normally distributed data. The smaller the p-value, the less trustworthy the prediciton. For highly non-normal X properties (e.g., fingerprints), the MD p-value is wildly inaccurate.

Structural Similar Compounds

Name	81840-15-5	112022-07-8	112022-06-7
Structure	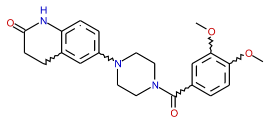	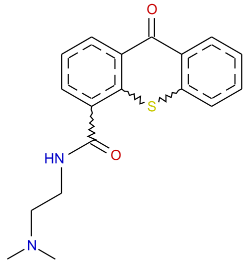	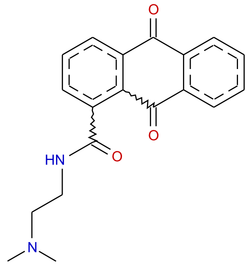
Actual Endpoint	Non-Mutagen	Mutagen	Mutagen
Predicted Endpoint	Non-Mutagen	Mutagen	Mutagen
Distance	0.545	0.578	0.582
Reference	Kazius et. al., J. Med. Chem. (2005) 48, 312-320	Kazius et. al., J. Med. Chem. (2005) 48, 312-320	Kazius et. al., J. Med. Chem. (2005) 48, 312-320

Model Applicability

Unknown features are fingerprint features in the query molecule, but not found or appearing too infrequently in the training set.

1. All properties and OPS components are within expected ranges.

Feature Contribution

Top features for positive contribution

Fingerprint	Bit/Smiles	Feature Structure	Score	Mutagen in training set
SCFP_12	-1211234921	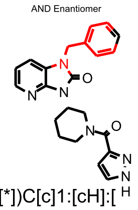 [*]N([*])C[c]1:[cH]:[cH]:[*]:[cH]:[cH]:1	0.514	15 out of 15

SCFP_12	1632957125	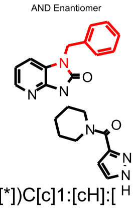 [*]N([*])C[c]1:[cH]:[cH]:[cH]:[cH]:[cH]:1	0.487	9 out of 9
SCFP_12	-587479743	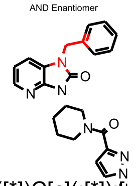 [*]N([*])C[c](:[*]):[*]	0.281	20 out of 26

Top Features for negative contribution

Fingerprint	Bit/Smiles	Feature Structure	Score	Mutagen in training set
SCFP_12	1190896200	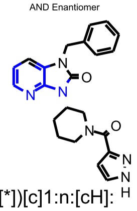 [*]N([*])[c]1:n:[cH]:[cH]:[*]:[c]:1[*]	-1.49	0 out of 6
SCFP_12	1205795299	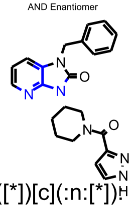 [*]N([*])[c](:n:[*]):[c]([*]):[*]	-1.22	2 out of 16
SCFP_12	306578635	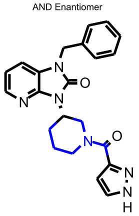 [*]C(=[*])N1C[*]CCC1	-1.09	3 out of 19

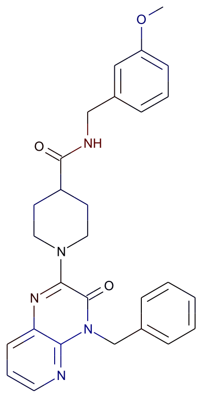

$C_{28}H_{29}N_5O_3$
Molecular Weight: 483.56156
ALogP: 2.942
Rotatable Bonds: 7
Acceptors: 6
Donors: 1

Model Prediction

Prediction: Non-Mutagen
Probability: 0.598
Enrichment: 1.07
Bayesian Score: -5.1
Mahalanobis Distance: 15.1
Mahalanobis Distance p-value: 7.24e-013

Prediction: Positive if the Bayesian score is above the estimated best cutoff value from minimizing the false positive and false negative rate.
Probability: The esimated probability that the sample is in the positive category. This assumes that the Bayesian score follows a normal distribution and is different from the prediction using a cutoff.
Enrichment: An estimate of enrichment, that is, the increased likelihood (versus random) of this sample being in the category.
Bayesian Score: The standard Laplacian-modified Bayesian score.
Mahalanobis Distance: The Mahalanobis distance (MD) is the distance to the center of the training data. The larger the MD, the less trustworthy the prediction.
Mahalanobis Distance p-value: The p-value gives the fraction of training data with an MD greater than or equal to the one for the given sample, assuming normally distributed data. The smaller the p-value, the less trustworthy the prediciton. For highly non-normal X properties (e.g., fingerprints), the MD p-value is wildly inaccurate.

Structural Similar Compounds

Name	83621-06-1	MORICIZINE	DILTIAZEM
Structure	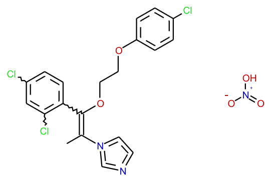	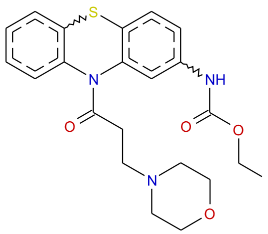	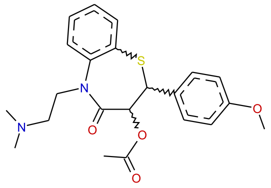
Actual Endpoint	Non-Mutagen	Non-Mutagen	Non-Mutagen
Predicted Endpoint	Non-Mutagen	Non-Mutagen	Non-Mutagen
Distance	0.573	0.580	0.591
Reference	Kazius et. al., J. Med. Chem. (2005) 48, 312-320	PDR 1994	PDR 1994

Model Applicability

Unknown features are fingerprint features in the query molecule, but not found or appearing too infrequently in the training set.

1. OPS PC35 out of range. Value: 6.1618. Training min, max, SD, explained variance: -6.6231, 5.2872, 0.925, 0.0091.

Feature Contribution

Top features for positive contribution

Fingerprint	Bit/Smiles	Feature Structure	Score	Mutagen in training set
SCFP_12	1575781215	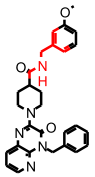 [*]C(=[*])NC[c](:[cH]:[*]):[c]([*]):[*]	0.517	16 out of 16

SCFP_12	-1211234921	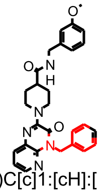 [*]N([*])C[c]1:[cH]:[cH]:[*]:[cH]:[cH]:1	0.514	15 out of 15
SCFP_12	44775464	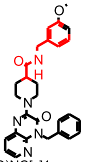 [*]C([*])C(=O)NC[c]1:[cH]:[cH]:[*]:[c]([*]):[cH]:1	0.514	15 out of 15

Top Features for negative contribution

Fingerprint	Bit/Smiles	Feature Structure	Score	Mutagen in training set
SCFP_12	1190896200	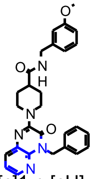 [*]N([*])[c]1:n:[cH]:[cH]:[*]:[c]:1[*]	-1.49	0 out of 6
SCFP_12	1205795299	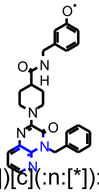 [*]N([*])[c](:n:[*]):[c]([*]):[*]	-1.22	2 out of 16
SCFP_12	-1632615624	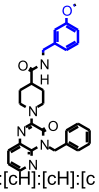 [*]C[c]1:[cH]:[cH]:[cH]:[c](O[*]):[cH]:1	-1.07	5 out of 29

#UNDEFINED

TOPKAT_Ames_Mutagenicity

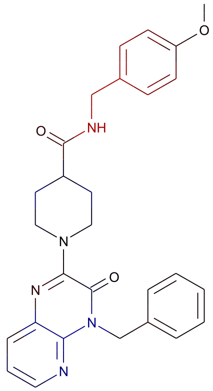

$C_{28}H_{29}N_5O_3$
Molecular Weight: 483.56156
ALogP: 2.942
Rotatable Bonds: 7
Acceptors: 6
Donors: 1

Model Prediction

Prediction: Non-Mutagen
Probability: 0.689
Enrichment: 1.23
Bayesian Score: -2.17
Mahalanobis Distance: 15.1
Mahalanobis Distance p-value: 7.24e-013

Prediction: Positive if the Bayesian score is above the estimated best cutoff value from minimizing the false positive and false negative rate.
Probability: The esimated probability that the sample is in the positive category. This assumes that the Bayesian score follows a normal distribution and is different from the prediction using a cutoff.
Enrichment: An estimate of enrichment, that is, the increased likelihood (versus random) of this sample being in the category.
Bayesian Score: The standard Laplacian-modified Bayesian score.
Mahalanobis Distance: The Mahalanobis distance (MD) is the distance to the center of the training data. The larger the MD, the less trustworthy the prediction.
Mahalanobis Distance p-value: The p-value gives the fraction of training data with an MD greater than or equal to the one for the given sample, assuming normally distributed data. The smaller the p-value, the less trustworthy the prediciton. For highly non-normal X properties (e.g., fingerprints), the MD p-value is wildly inaccurate.

Structural Similar Compounds

Name	83621-06-1	MORICIZINE	DILTIAZEM
Structure	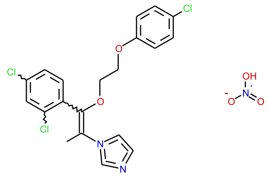	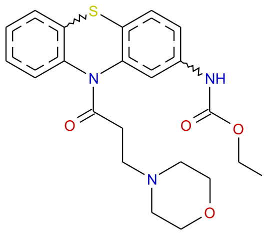	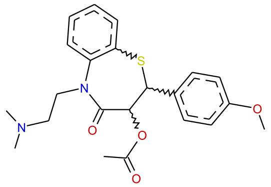
Actual Endpoint	Non-Mutagen	Non-Mutagen	Non-Mutagen
Predicted Endpoint	Non-Mutagen	Non-Mutagen	Non-Mutagen
Distance	0.573	0.580	0.586
Reference	Kazius et. al., J. Med. Chem. (2005) 48, 312-320	PDR 1994	PDR 1994

Model Applicability

Unknown features are fingerprint features in the query molecule, but not found or appearing too infrequently in the training set.

1. OPS PC35 out of range. Value: 6.1618. Training min, max, SD, explained variance: -6.6231, 5.2872, 0.925, 0.0091.

Feature Contribution

Top features for positive contribution				
**Fingerprint**	**Bit/Smiles**	**Feature Structure**	**Score**	**Mutagen in training set**
SCFP_12	1575781215	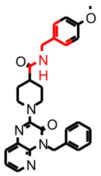 [*]C(=[*])NC[c](:[cH]:[*]):[c]([*]):[*]	0.517	16 out of 16

SCFP_12	44775464	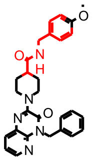 [*]C([*])C(=O)NC[c]1:[cH]:[cH]:[*]:[c]([*]):[cH]:1	0.514	15 out of 15
SCFP_12	-1211234921	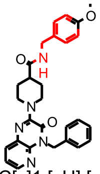 [*]N([*])C[c]1:[cH]:[cH]:[*]:[cH]:[cH]:1	0.514	15 out of 15
**Top Features for negative contribution**				
**Fingerprint**	**Bit/Smiles**	**Feature Structure**	**Score**	**Mutagen in training set**
SCFP_12	1190896200	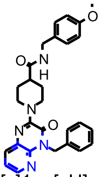 [*]N([*])[c]1:n:[cH]:[cH]:[*]:[c]:1[*]	-1.49	0 out of 6
SCFP_12	1205795299	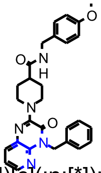 [*]N([*])[c](:n:[*]):[c]([*]):[*]	-1.22	2 out of 16
SCFP_12	-1043310069	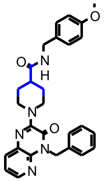 [*]CC(C[*])C(=[*])[*]	-0.989	44 out of 210

Molecule

TOPKAT_Developmental_Toxicity_Potential

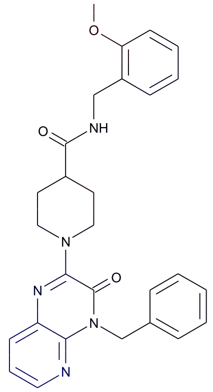

$C_{28}H_{29}N_5O_3$

Molecular Weight: 483.56156

ALogP: 2.942

Rotatable Bonds: 7

Acceptors: 6

Donors: 1

Structural Similar Compounds

Name	Acetemetacin	Ketoconazole	Beclomethasone Dipropionate
Structure	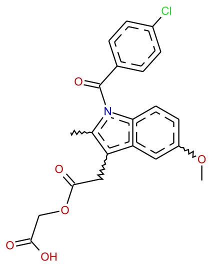	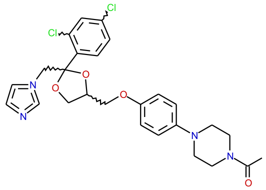	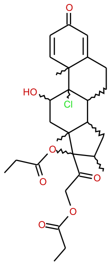
Actual Endpoint	Non-Toxic	Toxic	Toxic
Predicted Endpoint	Non-Toxic	Toxic	Toxic
Distance	0.594	0.609	0.627
Reference	Oyo Yakuri 22(6):777-786; 1981	Kiso to Rinsho 18:1433-1448; 1984	Oyo Yakuri 18(6):1021-1038; 1979

Model Prediction

Prediction: Non-Toxic

Probability: 0.417

Enrichment: 0.793

Bayesian Score: -3.83

Mahalanobis Distance: 12.4

Mahalanobis Distance p-value: 5.87e-006

Prediction: Positive if the Bayesian score is above the estimated best cutoff value from minimizing the false positive and false negative rate.
Probability: The esimated probability that the sample is in the positive category. This assumes that the Bayesian score follows a normal distribution and is different from the prediction using a cutoff.
Enrichment: An estimate of enrichment, that is, the increased likelihood (versus random) of this sample being in the category.
Bayesian Score: The standard Laplacian-modified Bayesian score.
Mahalanobis Distance: The Mahalanobis distance (MD) is the distance to the center of the training data. The larger the MD, the less trustworthy the prediction.
Mahalanobis Distance p-value: The p-value gives the fraction of training data with an MD greater than or equal to the one for the given sample, assuming normally distributed data. The smaller the p-value, the less trustworthy the prediciton. For highly non-normal X properties (e.g., fingerprints), the MD p-value is wildly inaccurate.

Model Applicability

Unknown features are fingerprint features in the query molecule, but not found or appearing too infrequently in the training set.

1. OPS PC22 out of range. Value: -2.536. Training min, max, SD, explained variance: -2.4788, 2.7503, 0.9196, 0.0124.

Feature Contribution

Top features for positive contribution

Fingerprint	Bit/Smiles	Feature Structure	Score	Toxic in training set
SCFP_6	591469355	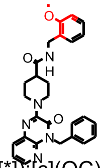 [*][c](:[*]):[c](OC):[cH]:[*]	0.411	10 out of 12

SCFP_6	2088704928	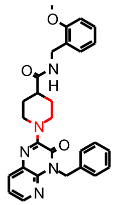 [*]CCN([*])[*]	0.303	38 out of 53
SCFP_6	136239834	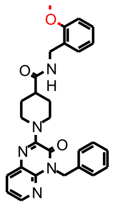 [*]OC	0.242	23 out of 34
**Top Features for negative contribution**				
**Fingerprint**	**Bit/Smiles**	**Feature Structure**	**Score**	**Toxic in training set**
SCFP_6	698986342	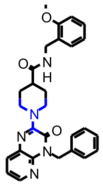 [*]CN(C[*])C(=[*])[*]	-0.718	0 out of 2
SCFP_6	-758850909	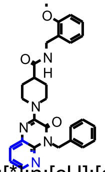 [*][c]1:[*]:n:[cH]:[cH]:[cH]:1	-0.646	2 out of 9
SCFP_6	1652428703	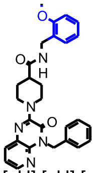 [*]O[c]1:[cH]:[cH]:[cH]:[cH]:[c]:1[*]	-0.438	1 out of 4

#UNDEFINED

TOPKAT_Developmental_Toxicity_Potential

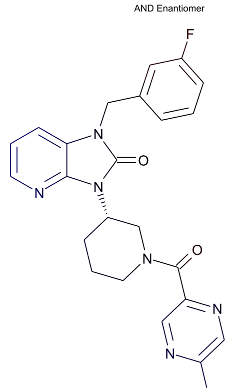

$C_{24}H_{23}FN_6O_2$
Molecular Weight: 446.47682
ALogP: 2.244
Rotatable Bonds: 4
Acceptors: 5
Donors: 0

Structural Similar Compounds

Name	Zopiclone	Pirenzepine	Rotenone
Structure	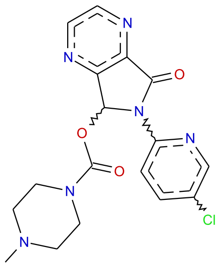	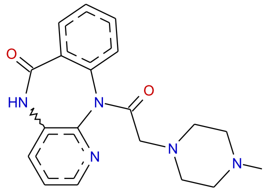	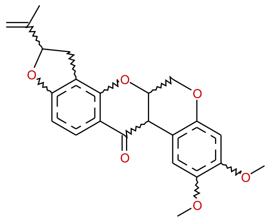
Actual Endpoint	Toxic	Non-Toxic	Toxic
Predicted Endpoint	Toxic	Non-Toxic	Toxic
Distance	0.618	0.659	0.666
Reference	Preclin Rep Cent Inst Exp Anim 9:145-156; 1983	Iyakuhin Kenkyu 11:424-436; 1980	J Toxicol Environ Health 10:111-119; 1982

Model Prediction

Prediction: Non-Toxic
Probability: 0.44
Enrichment: 0.837
Bayesian Score: -3.07
Mahalanobis Distance: 10.4
Mahalanobis Distance p-value: 0.00803

Prediction: Positive if the Bayesian score is above the estimated best cutoff value from minimizing the false positive and false negative rate.
Probability: The esimated probability that the sample is in the positive category. This assumes that the Bayesian score follows a normal distribution and is different from the prediction using a cutoff.
Enrichment: An estimate of enrichment, that is, the increased likelihood (versus random) of this sample being in the category.
Bayesian Score: The standard Laplacian-modified Bayesian score.
Mahalanobis Distance: The Mahalanobis distance (MD) is the distance to the center of the training data. The larger the MD, the less trustworthy the prediction.
Mahalanobis Distance p-value: The p-value gives the fraction of training data with an MD greater than or equal to the one for the given sample, assuming normally distributed data. The smaller the p-value, the less trustworthy the prediciton. For highly non-normal X properties (e.g., fingerprints), the MD p-value is wildly inaccurate.

Model Applicability

Unknown features are fingerprint features in the query molecule, but not found or appearing too infrequently in the training set.

1. All properties and OPS components are within expected ranges.

Feature Contribution

Top features for positive contribution				
Fingerprint	Bit/Smiles	Feature Structure	Score	Toxic in training set
SCFP_6	1257084377	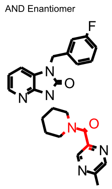 [*]N([*])C(=O)[c](:[*]):[*]	0.362	14 out of 18

SCFP_6	-783770208	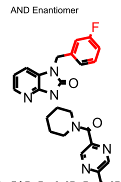 [*][c]1:[*]:[cH]:[cH]:[c](F):[cH]:1	0.322	4 out of 5
SCFP_6	-1410969850	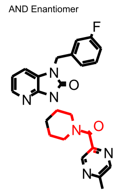 [*][C@H]1[*]CCN(C1)C(=O)[c](:[*]):[*]	0.271	1 out of 1

Top Features for negative contribution

Fingerprint	Bit/Smiles	Feature Structure	Score	Toxic in training set
SCFP_6	-758850909	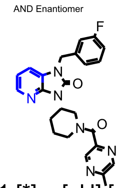 [*][c]1:[*]:n:[cH]:[cH]:[cH]:1	-0.646	2 out of 9
SCFP_6	2109374332	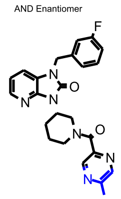 [*]:[cH]:[c](C):n:[*]	-0.446	3 out of 10
SCFP_6	1194442465	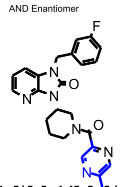 [*][c]1:[*]:[cH]:[c](C):n:[cH]:1	-0.438	1 out of 4

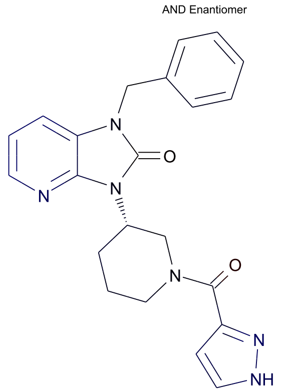

$C_{22}H_{22}N_6O_2$
Molecular Weight: 402.44907
ALogP: 2.501
Rotatable Bonds: 4
Acceptors: 4
Donors: 1

Structural Similar Compounds

Name	Clebopride Malate	Tiaramide .HCl (Free base form)	Quinine
Structure	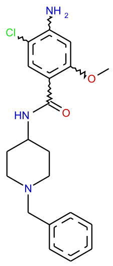	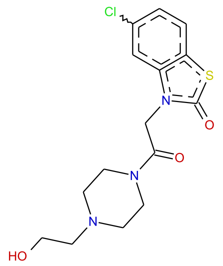	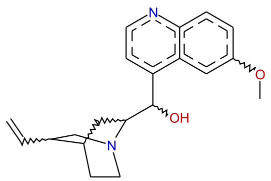
Actual Endpoint	Non-Toxic	Toxic	Toxic
Predicted Endpoint	Non-Toxic	Toxic	Toxic
Distance	0.591	0.598	0.606
Reference	Kiso to Rinsho 16:5649-5660; 1982	Arzneimittelforschung 23(4):504-8; 1973	Biol Neonat 36(5-6):273-6; 1979

Model Applicability

Unknown features are fingerprint features in the query molecule, but not found or appearing too infrequently in the training set.

1. All properties and OPS components are within expected ranges.

Feature Contribution

Top features for positive contribution

Fingerprint	Bit/Smiles	Feature Structure	Score	Toxic in training set
SCFP_6	1257084377	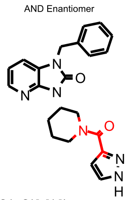 [*]N([*])C(=O)[c](:[*]):[*]	0.362	14 out of 18

Model Prediction

Prediction: Non-Toxic
Probability: 0.404
Enrichment: 0.768
Bayesian Score: -4.28
Mahalanobis Distance: 9.59
Mahalanobis Distance p-value: 0.0553

Prediction: Positive if the Bayesian score is above the estimated best cutoff value from minimizing the false positive and false negative rate.
Probability: The esimated probability that the sample is in the positive category. This assumes that the Bayesian score follows a normal distribution and is different from the prediction using a cutoff.
Enrichment: An estimate of enrichment, that is, the increased likelihood (versus random) of this sample being in the category.
Bayesian Score: The standard Laplacian-modified Bayesian score.
Mahalanobis Distance: The Mahalanobis distance (MD) is the distance to the center of the training data. The larger the MD, the less trustworthy the prediction.
Mahalanobis Distance p-value: The p-value gives the fraction of training data with an MD greater than or equal to the one for the given sample, assuming normally distributed data. The smaller the p-value, the less trustworthy the prediciton. For highly non-normal X properties (e.g., fingerprints), the MD p-value is wildly inaccurate.

SCFP_6	-1410969850	AND Enantiomer 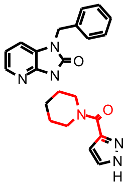 [*][C@H]1[*]CCN(C1)C(=O)[c](:[*]):[*]	0.271	1 out of 1
SCFP_6	1190896200	AND Enantiomer 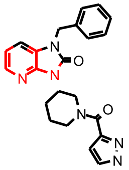 [*]N([*])[c]1:n:[cH]:[cH]:[*]:[c]:1[*]	0.153	2 out of 3
**Top Features for negative contribution**				
**Fingerprint**	**Bit/Smiles**	**Feature Structure**	**Score**	**Toxic in training set**
SCFP_6	-758850909	AND Enantiomer 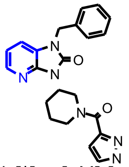 [*][c]1:[*]:n:[cH]:[cH]:[cH]:1	-0.646	2 out of 9
SCFP_6	149212520	AND Enantiomer 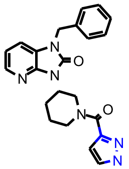 [*][c]1:[*]:[*]:[nH]:n:1	-0.448	5 out of 16
SCFP_6	240509252	AND Enantiomer 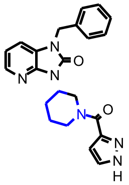 [*][C@@H]1[*]N([*])CCC1	-0.438	1 out of 4

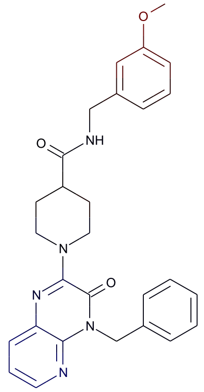

$C_{28}H_{29}N_5O_3$
Molecular Weight: 483.56156
ALogP: 2.942
Rotatable Bonds: 7
Acceptors: 6
Donors: 1

Structural Similar Compounds

Name	Acetametacin	Ketoconazole	Beclomethasone Dipropionate
Structure	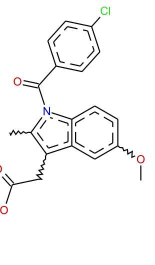	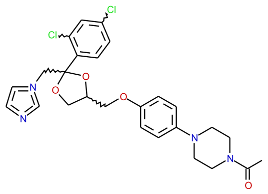	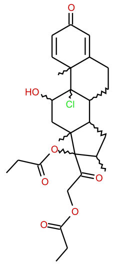
Actual Endpoint	Non-Toxic	Toxic	Toxic
Predicted Endpoint	Non-Toxic	Toxic	Toxic
Distance	0.590	0.605	0.627
Reference	Oyo Yakuri 22(6):777-786; 1981	Kiso to Rinsho 18:1433-1448; 1984	Oyo Yakuri 18(6):1021-1038; 1979

Model Prediction

Prediction: Non-Toxic
Probability: 0.458
Enrichment: 0.871
Bayesian Score: -2.52
Mahalanobis Distance: 12.4
Mahalanobis Distance p-value: 5.87e-006

Prediction: Positive if the Bayesian score is above the estimated best cutoff value from minimizing the false positive and false negative rate.
Probability: The esimated probability that the sample is in the positive category. This assumes that the Bayesian score follows a normal distribution and is different from the prediction using a cutoff.
Enrichment: An estimate of enrichment, that is, the increased likelihood (versus random) of this sample being in the category.
Bayesian Score: The standard Laplacian-modified Bayesian score.
Mahalanobis Distance: The Mahalanobis distance (MD) is the distance to the center of the training data. The larger the MD, the less trustworthy the prediction.
Mahalanobis Distance p-value: The p-value gives the fraction of training data with an MD greater than or equal to the one for the given sample, assuming normally distributed data. The smaller the p-value, the less trustworthy the prediciton. For highly non-normal X properties (e.g., fingerprints), the MD p-value is wildly inaccurate.

Model Applicability

Unknown features are fingerprint features in the query molecule, but not found or appearing too infrequently in the training set.

1. OPS PC22 out of range. Value: -2.536. Training min, max, SD, explained variance: -2.4788, 2.7503, 0.9196, 0.0124.

Feature Contribution

Top features for positive contribution

Fingerprint	Bit/Smiles	Feature Structure	Score	Toxic in training set
SCFP_6	1237755852	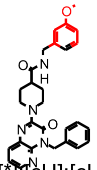 [*][c]1:[*]:[cH]:[cH]:[c](OC):[cH]:1	0.453	8 out of 9

SCFP_6	591469355	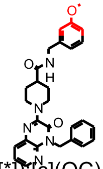 [*][c](:[*]):[c](OC):[cH]:[*]	0.411	10 out of 12
SCFP_6	2088704928	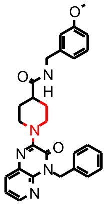 [*]CCN([*])[*]	0.303	38 out of 53
**Top Features for negative contribution**				
**Fingerprint**	**Bit/Smiles**	**Feature Structure**	**Score**	**Toxic in training set**
SCFP_6	698986342	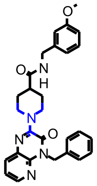 [*]CN(C[*])C(=[*])[*]	-0.718	0 out of 2
SCFP_6	-758850909	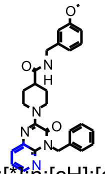 [*][c]1:[*]:n:[cH]:[cH]:[cH]:1	-0.646	2 out of 9
SCFP_6	-103134799	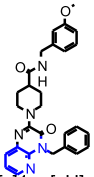 [*]N([*])[c]1:n:[cH]:[cH]:[cH]:[c]:1[*]	-0.422	0 out of 1

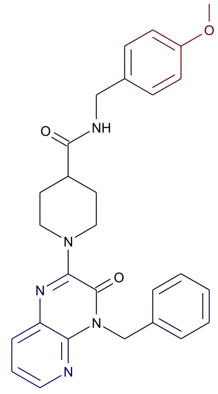

$C_{28}H_{29}N_5O_3$

Molecular Weight: 483.56156

ALogP: 2.942

Rotatable Bonds: 7

Acceptors: 6

Donors: 1

Model Prediction

Prediction: Non-Toxic

Probability: 0.46

Enrichment: 0.875

Bayesian Score: -2.46

Mahalanobis Distance: 12.4

Mahalanobis Distance p-value: 5.87e-006

Prediction: Positive if the Bayesian score is above the estimated best cutoff value from minimizing the false positive and false negative rate.
Probability: The esimated probability that the sample is in the positive category. This assumes that the Bayesian score follows a normal distribution and is different from the prediction using a cutoff.
Enrichment: An estimate of enrichment, that is, the increased likelihood (versus random) of this sample being in the category.
Bayesian Score: The standard Laplacian-modified Bayesian score.
Mahalanobis Distance: The Mahalanobis distance (MD) is the distance to the center of the training data. The larger the MD, the less trustworthy the prediction.
Mahalanobis Distance p-value: The p-value gives the fraction of training data with an MD greater than or equal to the one for the given sample, assuming normally distributed data. The smaller the p-value, the less trustworthy the prediciton. For highly non-normal X properties (e.g., fingerprints), the MD p-value is wildly inaccurate.

Structural Similar Compounds

Name	Acemetacin	Ketoconazole	Beclomethasone Dipropionate
Structure	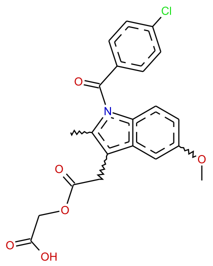	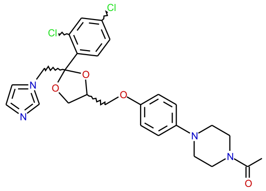	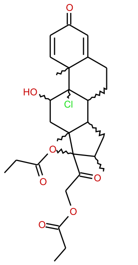
Actual Endpoint	Non-Toxic	Toxic	Toxic
Predicted Endpoint	Non-Toxic	Toxic	Toxic
Distance	0.589	0.605	0.627
Reference	Oyo Yakuri 22(6):777-786; 1981	Kiso to Rinsho 18:1433-1448; 1984	Oyo Yakuri 18(6):1021-1038; 1979

Model Applicability

Unknown features are fingerprint features in the query molecule, but not found or appearing too infrequently in the training set.

1. OPS PC22 out of range. Value: -2.536. Training min, max, SD, explained variance: -2.4788, 2.7503, 0.9196, 0.0124.

Feature Contribution

Top features for positive contribution

Fingerprint	Bit/Smiles	Feature Structure	Score	Toxic in training set
SCFP_6	-1849095515	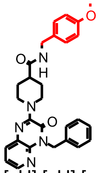 [*]C[c]1:[cH]:[cH]:[c](OC):[cH]:[cH]:1	0.478	4 out of 4

SCFP_6	1237755852	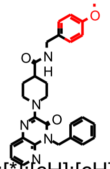 [*][c]1:[*]:[cH]:[cH]:[c](OC):[cH]:1	0.453	8 out of 9
SCFP_6	591469355	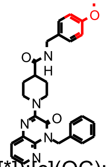 [*][c](:[*]):[c](OC):[cH]:[*]	0.411	10 out of 12

Top Features for negative contribution

Fingerprint	Bit/Smiles	Feature Structure	Score	Toxic in training set
SCFP_6	698986342	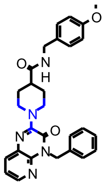 [*]CN(C[*])C(=[*])[*]	-0.718	0 out of 2
SCFP_6	-758850909	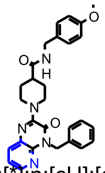 [*][c]1:[*]:n:[cH]:[cH]:[cH]:1	-0.646	2 out of 9
SCFP_6	-103134799	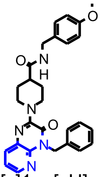 [*]N([*])[c]1:n:[cH]:[cH]:[cH]:[c]:1[*]	-0.422	0 out of 1

Molecule

TOPKAT_Mouse_Female_FDA_None_vs_Carcinogen

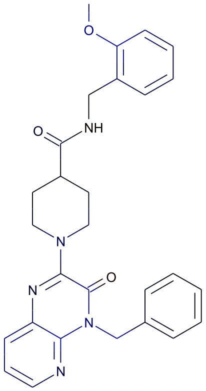

$C_{28}H_{29}N_5O_3$

Molecular Weight: 483.56156

ALogP: 2.942

Rotatable Bonds: 7

Acceptors: 6

Donors: 1

Structural Similar Compounds

Name	Moricizine	Fluticasone	Diltiazem
Structure	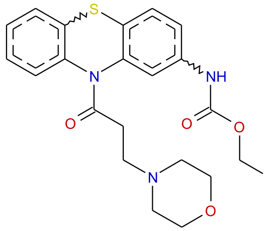	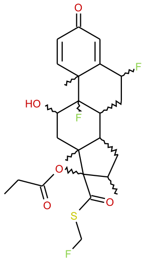	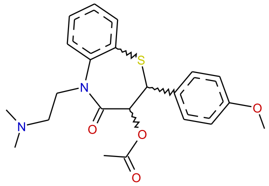
Actual Endpoint	Carcinogen	Non-Carcinogen	Non-Carcinogen
Predicted Endpoint	Carcinogen	Non-Carcinogen	Non-Carcinogen
Distance	0.583	0.602	0.627
Reference	US FDA (Centre for Drug Eval.& Res./Off. Testing & Res.) Sept. 1997	US FDA (Centre for Drug Eval.& Res./Off. Testing & Res.) Sept. 1997	US FDA (Centre for Drug Eval.& Res./Off. Testing & Res.) Sept. 1997

Model Prediction

Prediction: Non-Carcinogen

Probability: 0.209

Enrichment: 0.651

Bayesian Score: -8.02

Mahalanobis Distance: 13.3

Mahalanobis Distance p-value: 0.000183

Prediction: Positive if the Bayesian score is above the estimated best cutoff value from minimizing the false positive and false negative rate.
Probability: The esimated probability that the sample is in the positive category. This assumes that the Bayesian score follows a normal distribution and is different from the prediction using a cutoff.
Enrichment: An estimate of enrichment, that is, the increased likelihood (versus random) of this sample being in the category.
Bayesian Score: The standard Laplacian-modified Bayesian score.
Mahalanobis Distance: The Mahalanobis distance (MD) is the distance to the center of the training data. The larger the MD, the less trustworthy the prediction.
Mahalanobis Distance p-value: The p-value gives the fraction of training data with an MD greater than or equal to the one for the given sample, assuming normally distributed data. The smaller the p-value, the less trustworthy the prediciton. For highly non-normal X properties (e.g., fingerprints), the MD p-value is wildly inaccurate.

Model Applicability

Unknown features are fingerprint features in the query molecule, but not found or appearing too infrequently in the training set.

1. All properties and OPS components are within expected ranges.
2. Unknown ECFP_2 feature: -857146788: [*]CC(C[*])C(=[*])[*]

Feature Contribution

Top features for positive contribution

Fingerprint	Bit/Smiles	Feature Structure	Score	Carcinogen in training set
ECFP_6	769925792	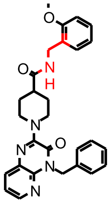 [*]NC[c](:[*]):[*]	0.617	2 out of 2

ECFP_6	2085698692	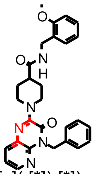 [*]C(=N[c](:[*]):[*])[*]	0.337	3 out of 6
ECFP_6	2106656448	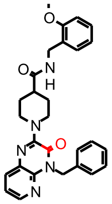 [*]C(=O)[*]	0.254	31 out of 77
**Top Features for negative contribution**				
**Fingerprint**	**Bit/Smiles**	**Feature Structure**	**Score**	**Carcinogen in training set**
ECFP_6	-661097313	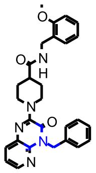 [*]CN(C(=[*])[*])[c](:[*]):[*]	-1.55	0 out of 12
ECFP_6	2013347047	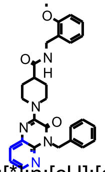 [*][c]1:[*]:n:[cH]:[cH]:[cH]:1	-0.805	0 out of 4
ECFP_6	1307307440	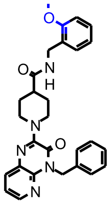 [*]:[c](:[*])OC	-0.558	4 out of 25

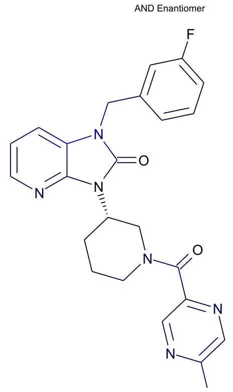

$C_{24}H_{23}FN_6O_2$
Molecular Weight: 446.47682
ALogP: 2.244
Rotatable Bonds: 4
Acceptors: 5
Donors: 0

Structural Similar Compounds

Name	Risperidone	Levocabstine	Buspirone
Structure	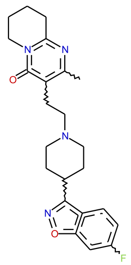	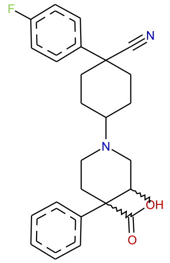	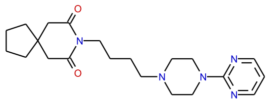
Actual Endpoint	Carcinogen	Carcinogen	Non-Carcinogen
Predicted Endpoint	Carcinogen	Carcinogen	Non-Carcinogen
Distance	0.587	0.614	0.629
Reference	US FDA (Centre for Drug Eval.& Res./Off. Testing & Res.) Sept. 1997	US FDA (Centre for Drug Eval.& Res./Off. Testing & Res.) Sept. 1997	US FDA (Centre for Drug Eval.& Res./Off. Testing & Res.) Sept. 1997

Model Prediction

Prediction: Non-Carcinogen
Probability: 0.212
Enrichment: 0.66
Bayesian Score: -8.56
Mahalanobis Distance: 15.8
Mahalanobis Distance p-value: 6.88e-009

Prediction: Positive if the Bayesian score is above the estimated best cutoff value from minimizing the false positive and false negative rate.
Probability: The esimated probability that the sample is in the positive category. This assumes that the Bayesian score follows a normal distribution and is different from the prediction using a cutoff.
Enrichment: An estimate of enrichment, that is, the increased likelihood (versus random) of this sample being in the category.
Bayesian Score: The standard Laplacian-modified Bayesian score.
Mahalanobis Distance: The Mahalanobis distance (MD) is the distance to the center of the training data. The larger the MD, the less trustworthy the prediction.
Mahalanobis Distance p-value: The p-value gives the fraction of training data with an MD greater than or equal to the one for the given sample, assuming normally distributed data. The smaller the p-value, the less trustworthy the prediciton. For highly non-normal X properties (e.g., fingerprints), the MD p-value is wildly inaccurate.

Model Applicability

Unknown features are fingerprint features in the query molecule, but not found or appearing too infrequently in the training set.

1. All properties and OPS components are within expected ranges.
2. Unknown ECFP_2 feature: 2077298510: [*]N([*])C(=O)[c](:[*]):[*]

Feature Contribution

Top features for positive contribution

Fingerprint	Bit/Smiles	Feature Structure	Score	Carcinogen in training set
ECFP_6	-1869628272	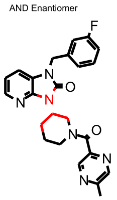 [*]CC(C[*])N([*])[*]	0.293	2 out of 4

ECFP_6	2106656448	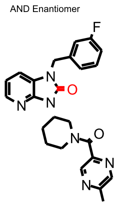 [*]C(=O)[*]	0.254	31 out of 77
ECFP_6	-1331450522	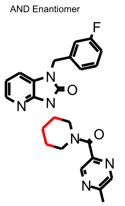 [*]CCC([*])[*]	0.238	25 out of 63

Top Features for negative contribution

Fingerprint	Bit/Smiles	Feature Structure	Score	Carcinogen in training set
ECFP_6	-661097313	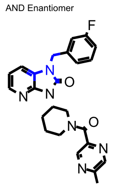 [*]CN(C(=[*])[*])[c](:[*]):[*]	-1.55	0 out of 12
ECFP_6	-1102925512	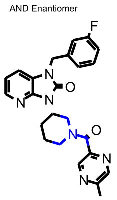 [*]CN(C[*])C(=[*])[*]	-0.805	0 out of 4
ECFP_6	2013347047	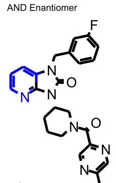 [*][c]1:[*]:n:[cH]:[cH]:[cH]:1	-0.805	0 out of 4

#UNDEFINED

TOPKAT_Mouse_Female_FDA_None_vs_Carcinogen

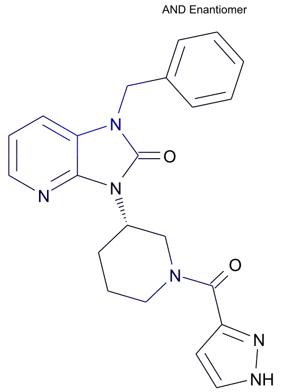

$C_{22}H_{22}N_6O_2$
Molecular Weight: 402.44907
ALogP: 2.501
Rotatable Bonds: 4
Acceptors: 4
Donors: 1

Model Prediction

Prediction: Non-Carcinogen
Probability: 0.206
Enrichment: 0.642
Bayesian Score: -7.14
Mahalanobis Distance: 14.7
Mahalanobis Distance p-value: 9.23e-007

Prediction: Positive if the Bayesian score is above the estimated best cutoff value from minimizing the false positive and false negative rate.
Probability: The esimated probability that the sample is in the positive category. This assumes that the Bayesian score follows a normal distribution and is different from the prediction using a cutoff.
Enrichment: An estimate of enrichment, that is, the increased likelihood (versus random) of this sample being in the category.
Bayesian Score: The standard Laplacian-modified Bayesian score.
Mahalanobis Distance: The Mahalanobis distance (MD) is the distance to the center of the training data. The larger the MD, the less trustworthy the prediction.
Mahalanobis Distance p-value: The p-value gives the fraction of training data with an MD greater than or equal to the one for the given sample, assuming normally distributed data. The smaller the p-value, the less trustworthy the prediciton. For highly non-normal X properties (e.g., fingerprints), the MD p-value is wildly inaccurate.

Structural Similar Compounds

Name	Levocabstine	Lansoprazole	Omeprazole
Structure	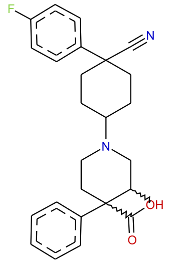	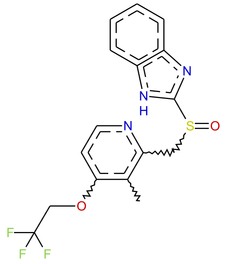	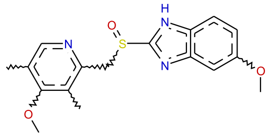
Actual Endpoint	Carcinogen	Carcinogen	Non-Carcinogen
Predicted Endpoint	Carcinogen	Carcinogen	Non-Carcinogen
Distance	0.536	0.627	0.632
Reference	US FDA (Centre for Drug Eval.& Res./Off. Testing & Res.) Sept. 1997	US FDA (Centre for Drug Eval.& Res./Off. Testing & Res.) Sept. 1997	US FDA (Centre for Drug Eval.& Res./Off. Testing & Res.) Sept. 1997

Model Applicability

Unknown features are fingerprint features in the query molecule, but not found or appearing too infrequently in the training set.

1. All properties and OPS components are within expected ranges.
2. Unknown ECFP_2 feature: 2077298510: [*]N([*])C(=O)[c](:[*]):[*]
3. Unknown ECFP_2 feature: 600440273: [*][c]1:[*]:[*]:[nH]:n:1
4. Unknown ECFP_2 feature: -954588747: [*]1:[*]:n:[nH]:[cH]:1

Feature Contribution

Top features for positive contribution				
**Fingerprint**	**Bit/Smiles**	**Feature Structure**	**Score**	**Carcinogen in training set**
ECFP_6	-1869628272	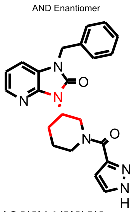 [*]CC(C[*])N([*])[*]	0.293	2 out of 4

ECFP_6	2106656448	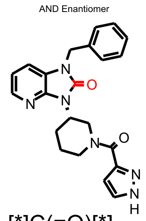 [*]C(=O)[*]	0.254	31 out of 77
ECFP_6	-152683720	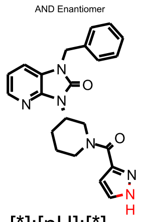 [*]:[nH]:[*]	0.245	7 out of 17
**Top Features for negative contribution**				
**Fingerprint**	**Bit/Smiles**	**Feature Structure**	**Score**	**Carcinogen in training set**
ECFP_6	-661097313	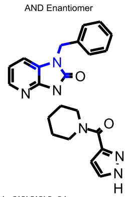 [*]CN(C(=[*])[*])[c](:[*]):[*]	-1.55	0 out of 12
ECFP_6	-1102925512	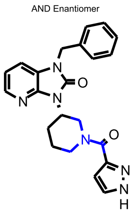 [*]CN(C[*])C(=[*])[*]	-0.805	0 out of 4
ECFP_6	2013347047	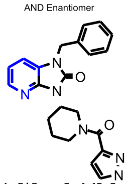 [*][c]1:[*]:n:[cH]:[cH]:[cH]:1	-0.805	0 out of 4

#UNDEFINED

TOPKAT_Mouse_Female_FDA_None_vs_Carcinogen

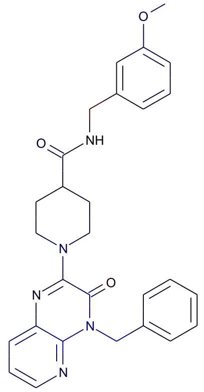

$C_{28}H_{29}N_5O_3$

Molecular Weight: 483.56156

ALogP: 2.942

Rotatable Bonds: 7

Acceptors: 6

Donors: 1

Model Prediction

Prediction: Non-Carcinogen

Probability: 0.205

Enrichment: 0.639

Bayesian Score: -6.71

Mahalanobis Distance: 16.6

Mahalanobis Distance p-value: 1.35e-010

Prediction: Positive if the Bayesian score is above the estimated best cutoff value from minimizing the false positive and false negative rate.
Probability: The esimated probability that the sample is in the positive category. This assumes that the Bayesian score follows a normal distribution and is different from the prediction using a cutoff.
Enrichment: An estimate of enrichment, that is, the increased likelihood (versus random) of this sample being in the category.
Bayesian Score: The standard Laplacian-modified Bayesian score.
Mahalanobis Distance: The Mahalanobis distance (MD) is the distance to the center of the training data. The larger the MD, the less trustworthy the prediction.
Mahalanobis Distance p-value: The p-value gives the fraction of training data with an MD greater than or equal to the one for the given sample, assuming normally distributed data. The smaller the p-value, the less trustworthy the prediciton. For highly non-normal X properties (e.g., fingerprints), the MD p-value is wildly inaccurate.

Structural Similar Compounds

Name	Moricizine	Fluticasone	Diltiazem
Structure	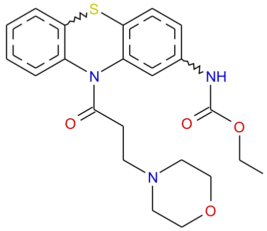	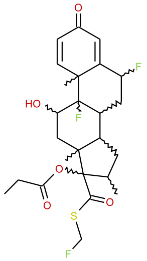	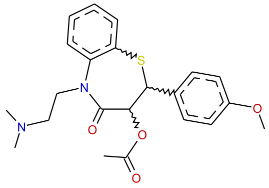
Actual Endpoint	Carcinogen	Non-Carcinogen	Non-Carcinogen
Predicted Endpoint	Carcinogen	Non-Carcinogen	Non-Carcinogen
Distance	0.580	0.602	0.620
Reference	US FDA (Centre for Drug Eval.& Res./Off. Testing & Res.) Sept. 1997	US FDA (Centre for Drug Eval.& Res./Off. Testing & Res.) Sept. 1997	US FDA (Centre for Drug Eval.& Res./Off. Testing & Res.) Sept. 1997

Model Applicability

Unknown features are fingerprint features in the query molecule, but not found or appearing too infrequently in the training set.

1. All properties and OPS components are within expected ranges.
2. Unknown ECFP_2 feature: -857146788: [*]CC(C[*])C(=[*])[*]

Feature Contribution

Top features for positive contribution

Fingerprint	Bit/Smiles	Feature Structure	Score	Carcinogen in training set
ECFP_6	769925792	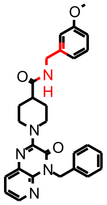 [*]NC[c](:[*]):[*]	0.617	2 out of 2

ECFP_6	-767769026	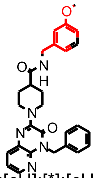 [*]C[c]1:[cH]:[*]:[cH]:[c](O[*]):[cH]:1	0.424	1 out of 1
ECFP_6	2068492356	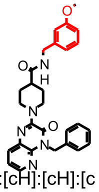 [*]C[c]1:[cH]:[cH]:[c H]:[c](O[*]):[cH]:1	0.424	1 out of 1
**Top Features for negative contribution**				
**Fingerprint**	**Bit/Smiles**	**Feature Structure**	**Score**	**Carcinogen in training set**
ECFP_6	-661097313	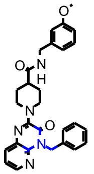 [*]CN(C(=[*])[*])[c](:[*]):[*]	-1.55	0 out of 12
ECFP_6	2013347047	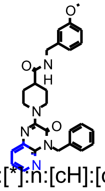 [*][c]1:[*]:n:[cH]:[c H]:[cH]:1	-0.805	0 out of 4
ECFP_6	2007300961	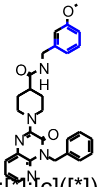 [*][c]1:[*]:[c]([*]): [cH]:[cH]:[cH]:1	-0.652	5 out of 34

#UNDEFINED

TOPKAT_Mouse_Female_FDA_None_vs_Carcinogen

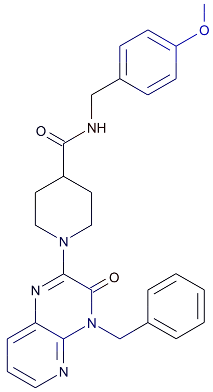

$C_{28}H_{29}N_5O_3$
Molecular Weight: 483.56156
ALogP: 2.942
Rotatable Bonds: 7
Acceptors: 6
Donors: 1

Model Prediction

Prediction: Non-Carcinogen
Probability: 0.209
Enrichment: 0.653
Bayesian Score: -8.19
Mahalanobis Distance: 12
Mahalanobis Distance p-value: 0.0105

Prediction: Positive if the Bayesian score is above the estimated best cutoff value from minimizing the false positive and false negative rate.
Probability: The esimated probability that the sample is in the positive category. This assumes that the Bayesian score follows a normal distribution and is different from the prediction using a cutoff.
Enrichment: An estimate of enrichment, that is, the increased likelihood (versus random) of this sample being in the category.
Bayesian Score: The standard Laplacian-modified Bayesian score.
Mahalanobis Distance: The Mahalanobis distance (MD) is the distance to the center of the training data. The larger the MD, the less trustworthy the prediction.
Mahalanobis Distance p-value: The p-value gives the fraction of training data with an MD greater than or equal to the one for the given sample, assuming normally distributed data. The smaller the p-value, the less trustworthy the prediciton. For highly non-normal X properties (e.g., fingerprints), the MD p-value is wildly inaccurate.

Structural Similar Compounds

Name	Moricizine	Fluticasone	Diltiazem
Structure	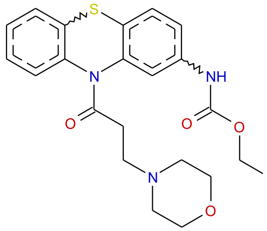	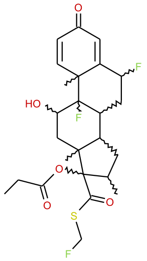	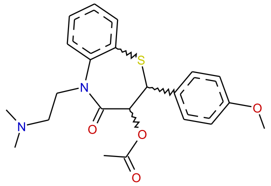
Actual Endpoint	Carcinogen	Non-Carcinogen	Non-Carcinogen
Predicted Endpoint	Carcinogen	Non-Carcinogen	Non-Carcinogen
Distance	0.582	0.602	0.605
Reference	US FDA (Centre for Drug Eval.& Res./Off. Testing & Res.) Sept. 1997	US FDA (Centre for Drug Eval.& Res./Off. Testing & Res.) Sept. 1997	US FDA (Centre for Drug Eval.& Res./Off. Testing & Res.) Sept. 1997

Model Applicability

Unknown features are fingerprint features in the query molecule, but not found or appearing too infrequently in the training set.

1. OPS PC20 out of range. Value: -3.4898. Training min, max, SD, explained variance: -3.1862, 4.4571, 1.28, 0.0167.
2. Unknown ECFP_2 feature: -857146788: [*]CC(C[*])C(=[*])[*]

Feature Contribution

Top features for positive contribution

Fingerprint	Bit/Smiles	Feature Structure	Score	Carcinogen in training set
ECFP_6	769925792	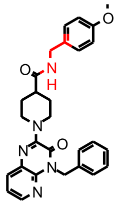 [*]NC[c](:[*]):[*]	0.617	2 out of 2

ECFP_6	110318898	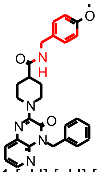 [*]NC[c]1:[cH]:[cH]:[*]:[cH]:[cH]:1	0.424	1 out of 1
ECFP_6	2085698692	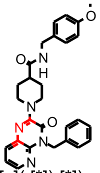 [*]C(=N[c](:[*]):[*])[*]	0.337	3 out of 6
**Top Features for negative contribution**				
**Fingerprint**	**Bit/Smiles**	**Feature Structure**	**Score**	**Carcinogen in training set**
ECFP_6	-661097313	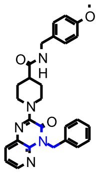 [*]CN(C(=[*])[*])[c](:[*]):[*]	-1.55	0 out of 12
ECFP_6	693720869	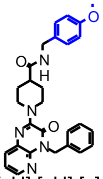 [*][c]1:[cH]:[cH]:[c](OC):[cH]:[cH]:1	-0.805	0 out of 4
ECFP_6	2013347047	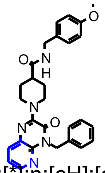 [*][c]1:[*]:n:[cH]:[cH]:[cH]:1	-0.805	0 out of 4

Molecule

TOPKAT_Mouse_Female_NTP

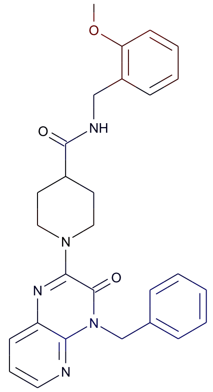

$C_{28}H_{29}N_5O_3$
Molecular Weight: 483.56156
ALogP: 2.942
Rotatable Bonds: 7
Acceptors: 6
Donors: 1

Structural Similar Compounds

Name	Curcumin	Rhodamine 6G	Rotenone
Structure	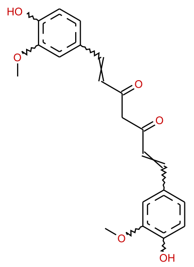	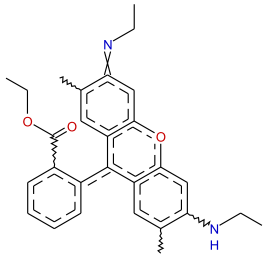	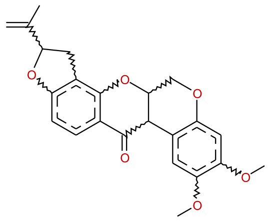
Actual Endpoint	Carcinogen	Non-Carcinogen	Non-Carcinogen
Predicted Endpoint	Carcinogen	Non-Carcinogen	Non-Carcinogen
Distance	0.704	0.732	0.779
Reference	NTP427	NTP/TR-364	NTP/TR-320

Model Prediction

Prediction: Non-Carcinogen
Probability: 0.371
Enrichment: 0.942
Bayesian Score: -4.5
Mahalanobis Distance: 12
Mahalanobis Distance p-value: 6.69e-007

Prediction: Positive if the Bayesian score is above the estimated best cutoff value from minimizing the false positive and false negative rate.
Probability: The esimated probability that the sample is in the positive category. This assumes that the Bayesian score follows a normal distribution and is different from the prediction using a cutoff.
Enrichment: An estimate of enrichment, that is, the increased likelihood (versus random) of this sample being in the category.
Bayesian Score: The standard Laplacian-modified Bayesian score.
Mahalanobis Distance: The Mahalanobis distance (MD) is the distance to the center of the training data. The larger the MD, the less trustworthy the prediction.
Mahalanobis Distance p-value: The p-value gives the fraction of training data with an MD greater than or equal to the one for the given sample, assuming normally distributed data. The smaller the p-value, the less trustworthy the prediciton. For highly non-normal X properties (e.g., fingerprints), the MD p-value is wildly inaccurate.

Model Applicability

Unknown features are fingerprint features in the query molecule, but not found or appearing too infrequently in the training set.

1. OPS PC18 out of range. Value: -2.9059. Training min, max, SD, explained variance: -2.8668, 4.1288, 1.043, 0.0181.
2. Unknown ECFP_2 feature: 671679640: [*]\N=C(\N([*])[*])/C(=[*])[*]
3. Unknown ECFP_2 feature: 2085698692: [*]C(=N[c](:[*]):[*])[*]
4. Unknown ECFP_2 feature: 1951894094: [*]CN(C[*])C(=[*])[*]
5. Unknown ECFP_2 feature: -81134287: [*]NC(=O)C([*])[*]
6. Unknown ECFP_2 feature: -44121127: [*]N([*])C[c](:[*]):[*]

Feature Contribution

Top features for positive contribution

Fingerprint	Bit/Smiles	Feature Structure	Score	Carcinogen in training set
ECFP_8	-1331450522	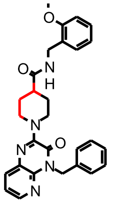 [*]CCC([*])[*]	0.48	9 out of 14

ECFP_8	1634699529	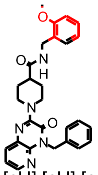 [*]O[c]1:[cH]:[cH]:[cH]:[*]:[c]:1[*]	0.477	3 out of 4
ECFP_8	-1961666573	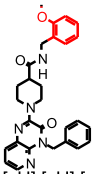 [*]O[c]1:[cH]:[cH]:[cH]:[cH]:[c]:1[*]	0.477	3 out of 4
**Top Features for negative contribution**				
**Fingerprint**	**Bit/Smiles**	**Feature Structure**	**Score**	**Carcinogen in training set**
ECFP_8	-1650219925	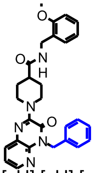 [*]C[c]1:[cH]:[cH]:[cH]:[cH]:[cH]:1	-0.856	1 out of 10
ECFP_8	-2024509555	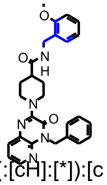 [*]C[c](:[cH]:[*]):[c]([*]):[*]	-0.748	0 out of 3
ECFP_8	-1897341097	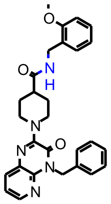 [*]N[*]	-0.555	10 out of 49

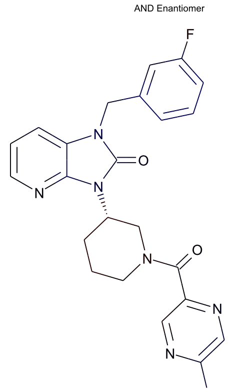

$C_{24}H_{23}FN_6O_2$
Molecular Weight: 446.47682
ALogP: 2.244
Rotatable Bonds: 4
Acceptors: 5
Donors: 0

Model Prediction

Prediction: Non-Carcinogen
Probability: 0.458
Enrichment: 1.16
Bayesian Score: -3.01
Mahalanobis Distance: 12.8
Mahalanobis Distance p-value: 1.07e-008

Prediction: Positive if the Bayesian score is above the estimated best cutoff value from minimizing the false positive and false negative rate.
Probability: The esimated probability that the sample is in the positive category. This assumes that the Bayesian score follows a normal distribution and is different from the prediction using a cutoff.
Enrichment: An estimate of enrichment, that is, the increased likelihood (versus random) of this sample being in the category.
Bayesian Score: The standard Laplacian-modified Bayesian score.
Mahalanobis Distance: The Mahalanobis distance (MD) is the distance to the center of the training data. The larger the MD, the less trustworthy the prediction.
Mahalanobis Distance p-value: The p-value gives the fraction of training data with an MD greater than or equal to the one for the given sample, assuming normally distributed data. The smaller the p-value, the less trustworthy the prediciton. For highly non-normal X properties (e.g., fingerprints), the MD p-value is wildly inaccurate.

Structural Similar Compounds

Name	Rotenone	Rotenone	3;3'-Dimethoxybenzidine-4-4'-diisocyanate
Structure	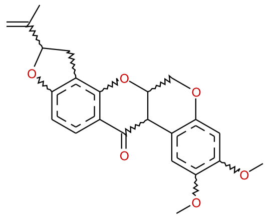	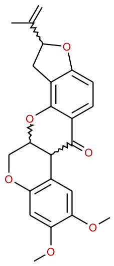	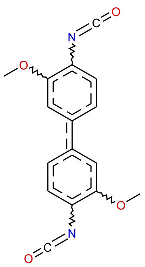
Actual Endpoint	Non-Carcinogen	Non-Carcinogen	Non-Carcinogen
Predicted Endpoint	Non-Carcinogen	Non-Carcinogen	Non-Carcinogen
Distance	0.670	0.670	0.746
Reference	NTP/TR-320	NTP320	NTP/TR-128

Model Applicability

Unknown features are fingerprint features in the query molecule, but not found or appearing too infrequently in the training set.

1. All properties and OPS components are within expected ranges.
2. Unknown ECFP_2 feature: -957084426: [*]C([*])N1C(=[*])[*][*]:[c]1:[*]
3. Unknown ECFP_2 feature: -1102925512: [*]CN(C[*])C(=[*])[*]
4. Unknown ECFP_2 feature: 2077298510: [*]N([*])C(=O)[c](:[*]):[*]
5. Unknown ECFP_2 feature: -1869628272: [*]CC(C[*])N([*])[*]
6. Unknown ECFP_2 feature: 1413420509: [*]C(=[*])[c](:[cH]:[*]):n:[*]
7. Unknown ECFP_2 feature: -44121127: [*]N([*])C[c](:[*]):[*]
8. Unknown ECFP_2 feature: -176686665: [*]:[cH]:[c](F):[cH]:[*]
9. Unknown ECFP_2 feature: 220735655: [*]:[c](:[*])F

Feature Contribution

Top features for positive contribution

Fingerprint	Bit/Smiles	Feature Structure	Score	Carcinogen in training set

ECFP_8	-1331450522	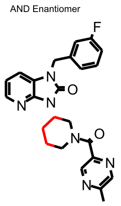 [*]CCC([*])[*]	0.48	9 out of 14
ECFP_8	-756348342	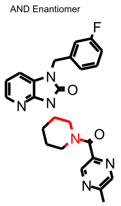 [*]C([*])CN([*])[*]	0.378	1 out of 1
ECFP_8	-709633021	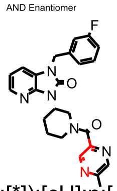 [*][c](:[*]):[cH]:n:[*]	0.351	2 out of 3

Top Features for negative contribution

Fingerprint	Bit/Smiles	Feature Structure	Score	Carcinogen in training set
ECFP_8	2007300961	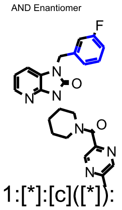 [*][c]1:[*]:[c]([*]):[cH]:[cH]:[cH]:1	-1.13	1 out of 14
ECFP_8	2146815437	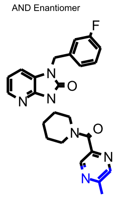 [*]:[cH]:[c](C):n:[*]	-0.555	0 out of 2

ECFP_8	1095683433	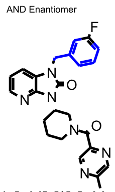 [*]C[c]1:[cH]:[*]:[cH]:[cH]:[cH]:1	-0.526	2 out of 11

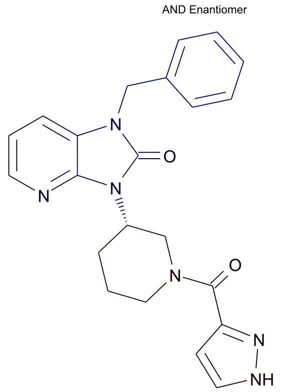

$C_{22}H_{22}N_6O_2$
Molecular Weight: 402.44907
ALogP: 2.501
Rotatable Bonds: 4
Acceptors: 4
Donors: 1

Model Prediction

Prediction: Non-Carcinogen

Probability: 0.466

Enrichment: 1.18

Bayesian Score: -2.86

Mahalanobis Distance: 10.7

Mahalanobis Distance p-value: 0.000264

Prediction: Positive if the Bayesian score is above the estimated best cutoff value from minimizing the false positive and false negative rate.
Probability: The esimated probability that the sample is in the positive category. This assumes that the Bayesian score follows a normal distribution and is different from the prediction using a cutoff.
Enrichment: An estimate of enrichment, that is, the increased likelihood (versus random) of this sample being in the category.
Bayesian Score: The standard Laplacian-modified Bayesian score.
Mahalanobis Distance: The Mahalanobis distance (MD) is the distance to the center of the training data. The larger the MD, the less trustworthy the prediction.
Mahalanobis Distance p-value: The p-value gives the fraction of training data with an MD greater than or equal to the one for the given sample, assuming normally distributed data. The smaller the p-value, the less trustworthy the prediciton. For highly non-normal X properties (e.g., fingerprints), the MD p-value is wildly inaccurate.

Structural Similar Compounds

Name	Chlorendic Acid	Chlorendic Acid	Acetohexamide
Structure	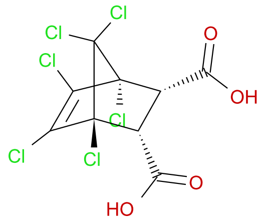	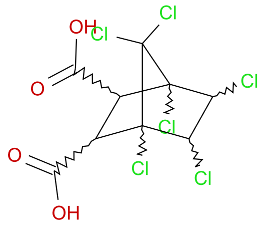	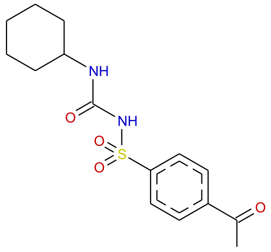
Actual Endpoint	Non-Carcinogen	Non-Carcinogen	Non-Carcinogen
Predicted Endpoint	Non-Carcinogen	Non-Carcinogen	Non-Carcinogen
Distance	0.667	0.671	0.683
Reference	NTP304	NTP/TR-304	NTP/TR-050

Model Applicability

Unknown features are fingerprint features in the query molecule, but not found or appearing too infrequently in the training set.

1. All properties and OPS components are within expected ranges.
2. Unknown ECFP_2 feature: -957084426: [*]C([*])N1C(=[*])[*][*]:[c]1:[*]
3. Unknown ECFP_2 feature: -1102925512: [*]CN(C[*])C(=[*])[*]
4. Unknown ECFP_2 feature: 2077298510: [*]N([*])C(=O)[c](:[*]):[*]
5. Unknown ECFP_2 feature: 1413420509: [*]C(=[*])[c](:[cH]:[*]):n:[*]
6. Unknown ECFP_2 feature: -1869628272: [*]CC(C[*])N([*])[*]
7. Unknown ECFP_2 feature: 600440273: [*][c]1:[*]:[*]:[nH]:n:1
8. Unknown ECFP_2 feature: -954588747: [*]1:[*]:n:[nH]:[cH]:1
9. Unknown ECFP_2 feature: -44121127: [*]N([*])C[c](:[*]):[*]
10. Unknown ECFP_2 feature: 1998023064: [*]1:[*]:[cH]:[cH]:[nH]:1

Feature Contribution

Top features for positive contribution				
**Fingerprint**	**Bit/Smiles**	**Feature Structure**	**Score**	**Carcinogen in training set**

ECFP_8	-1331450522	AND Enantiomer 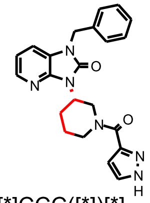 [*]CCC([*])[*]	0.48	9 out of 14
ECFP_8	-756348342	AND Enantiomer 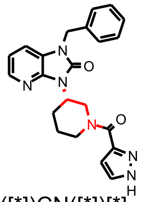 [*]C([*])CN([*])[*]	0.378	1 out of 1
ECFP_8	-152683720	AND Enantiomer 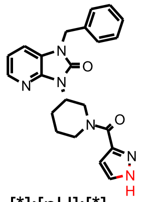 [*]:[nH]:[*]	0.351	2 out of 3
**Top Features for negative contribution**				
**Fingerprint**	**Bit/Smiles**	**Feature Structure**	**Score**	**Carcinogen in training set**
ECFP_8	-1650219925	AND Enantiomer 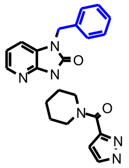 [*]C[c]1:[cH]:[cH]:[cH]:[cH]:[cH]:1	-0.856	1 out of 10
ECFP_8	1095683433	AND Enantiomer 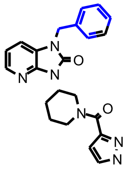 [*]C[c]1:[cH]:[*]:[cH]:[cH]:[cH]:1	-0.526	2 out of 11

ECFP_8	-677309799	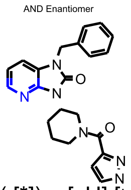 [*][c](:[*]):n:[cH]:[*]	-0.368	2 out of 9

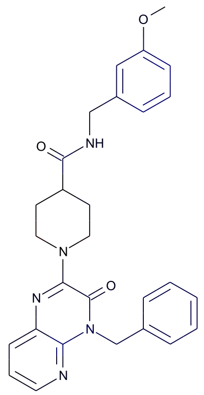

$C_{28}H_{29}N_5O_3$
Molecular Weight: 483.56156
ALogP: 2.942
Rotatable Bonds: 7
Acceptors: 6
Donors: 1

Model Prediction

Prediction: Non-Carcinogen
Probability: 0.189
Enrichment: 0.479
Bayesian Score: -7.37
Mahalanobis Distance: 13.4
Mahalanobis Distance p-value: 3.23e-010

Prediction: Positive if the Bayesian score is above the estimated best cutoff value from minimizing the false positive and false negative rate.
Probability: The esimated probability that the sample is in the positive category. This assumes that the Bayesian score follows a normal distribution and is different from the prediction using a cutoff.
Enrichment: An estimate of enrichment, that is, the increased likelihood (versus random) of this sample being in the category.
Bayesian Score: The standard Laplacian-modified Bayesian score.
Mahalanobis Distance: The Mahalanobis distance (MD) is the distance to the center of the training data. The larger the MD, the less trustworthy the prediction.
Mahalanobis Distance p-value: The p-value gives the fraction of training data with an MD greater than or equal to the one for the given sample, assuming normally distributed data. The smaller the p-value, the less trustworthy the prediciton. For highly non-normal X properties (e.g., fingerprints), the MD p-value is wildly inaccurate.

Structural Similar Compounds

Name	Curcumin	Rhodamine 6G	Rotenone
Structure	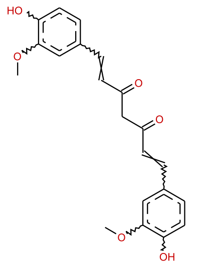	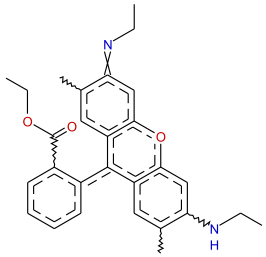	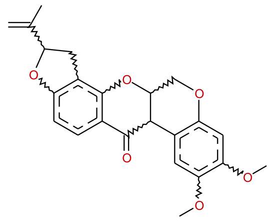
Actual Endpoint	Carcinogen	Non-Carcinogen	Non-Carcinogen
Predicted Endpoint	Carcinogen	Non-Carcinogen	Non-Carcinogen
Distance	0.708	0.730	0.782
Reference	NTP427	NTP/TR-364	NTP/TR-320

Model Applicability

Unknown features are fingerprint features in the query molecule, but not found or appearing too infrequently in the training set.

1. All properties and OPS components are within expected ranges.
2. Unknown ECFP_2 feature: 671679640: [*]\N=C(\N([*])[*])/C(=[*])[*]
3. Unknown ECFP_2 feature: 2085698692: [*]C(=N[c](:[*]):[*])[*]
4. Unknown ECFP_2 feature: 1951894094: [*]CN(C[*])C(=[*])[*]
5. Unknown ECFP_2 feature: -81134287: [*]NC(=O)C([*])[*]
6. Unknown ECFP_2 feature: -44121127: [*]N([*])C[c](:[*]):[*]

Feature Contribution

Top features for positive contribution				
**Fingerprint**	**Bit/Smiles**	**Feature Structure**	**Score**	**Carcinogen in training set**
ECFP_8	-1331450522	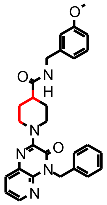 [*]CCC([*])[*]	0.48	9 out of 14

ECFP_8	769925792	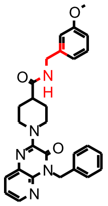 [*]NC[c](:[*]):[*]	0.378	1 out of 1
ECFP_8	2013347047	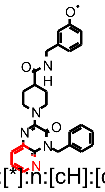 [*][c]1:[*]:n:[cH]:[cH]:[cH]:1	0.351	2 out of 3
**Top Features for negative contribution**				
**Fingerprint**	**Bit/Smiles**	**Feature Structure**	**Score**	**Carcinogen in training set**
ECFP_8	2007300961	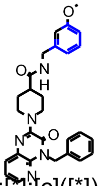 [*][c]1:[*]:[c]([*]):[cH]:[cH]:[cH]:1	-1.13	1 out of 14
ECFP_8	-1650219925	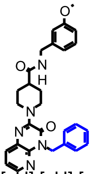 [*]C[c]1:[cH]:[cH]:[cH]:[cH]:[cH]:1	-0.856	1 out of 10
ECFP_8	-1897341097	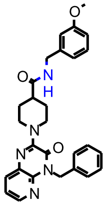 [*]N[*]	-0.555	10 out of 49

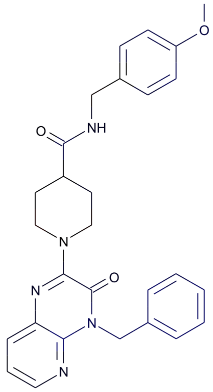

$C_{28}H_{29}N_5O_3$
Molecular Weight: 483.56156
ALogP: 2.942
Rotatable Bonds: 7
Acceptors: 6
Donors: 1

Structural Similar Compounds

Name	Curcumin	Rhodamine 6G	Rotenone
Structure	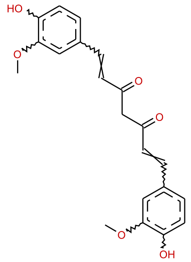	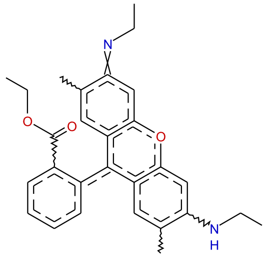	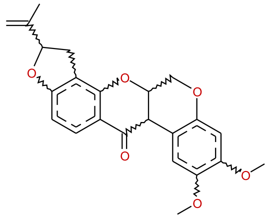
Actual Endpoint	Carcinogen	Non-Carcinogen	Non-Carcinogen
Predicted Endpoint	Carcinogen	Non-Carcinogen	Non-Carcinogen
Distance	0.711	0.732	0.784
Reference	NTP427	NTP/TR-364	NTP/TR-320

Model Prediction

Prediction: Non-Carcinogen
Probability: 0.229
Enrichment: 0.581
Bayesian Score: -6.7
Mahalanobis Distance: 12.9
Mahalanobis Distance p-value: 4.56e-009

Prediction: Positive if the Bayesian score is above the estimated best cutoff value from minimizing the false positive and false negative rate.
Probability: The esimated probability that the sample is in the positive category. This assumes that the Bayesian score follows a normal distribution and is different from the prediction using a cutoff.
Enrichment: An estimate of enrichment, that is, the increased likelihood (versus random) of this sample being in the category.
Bayesian Score: The standard Laplacian-modified Bayesian score.
Mahalanobis Distance: The Mahalanobis distance (MD) is the distance to the center of the training data. The larger the MD, the less trustworthy the prediction.
Mahalanobis Distance p-value: The p-value gives the fraction of training data with an MD greater than or equal to the one for the given sample, assuming normally distributed data. The smaller the p-value, the less trustworthy the prediciton. For highly non-normal X properties (e.g., fingerprints), the MD p-value is wildly inaccurate.

Model Applicability

Unknown features are fingerprint features in the query molecule, but not found or appearing too infrequently in the training set.

1. All properties and OPS components are within expected ranges.
2. Unknown ECFP_2 feature: 671679640: [*]\N=C(\N([*])[*])/C(=[*])[*]
3. Unknown ECFP_2 feature: 2085698692: [*]C(=N[c](:[*]):[*])[*]
4. Unknown ECFP_2 feature: 1951894094: [*]CN(C[*])C(=[*])[*]
5. Unknown ECFP_2 feature: -81134287: [*]NC(=O)C([*])[*]
6. Unknown ECFP_2 feature: -44121127: [*]N([*])C[c](:[*]):[*]

Feature Contribution

Top features for positive contribution

Fingerprint	Bit/Smiles	Feature Structure	Score	Carcinogen in training set
ECFP_8	-1331450522	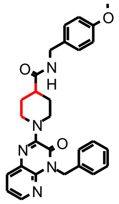 [*]CCC([*])[*]	0.48	9 out of 14

ECFP_8	769925792	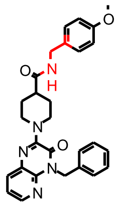<br>[*]NC[c](:[*]):[*]	0.378	1 out of 1
ECFP_8	2013347047	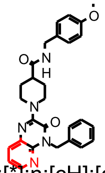<br>[*][c]1:[*]:n:[cH]:[c<br>H]:[cH]:1	0.351	2 out of 3

Top Features for negative contribution

Fingerprint	Bit/Smiles	Feature Structure	Score	Carcinogen in training set
ECFP_8	-1650219925	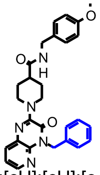<br>[*]C[c]1:[cH]:[cH]:[c<br>H]:[cH]:[cH]:1	-0.856	1 out of 10
ECFP_8	-1897341097	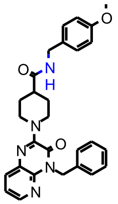<br>[*]N[*]	-0.555	10 out of 49
ECFP_8	-1271104377	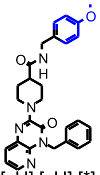<br>CO[c]1:[cH]:[cH]:[*]:<br>[cH]:[cH]:1	-0.555	0 out of 2

Molecule

TOPKAT_Mouse_Male_FDA_None_vs_Carcinogen

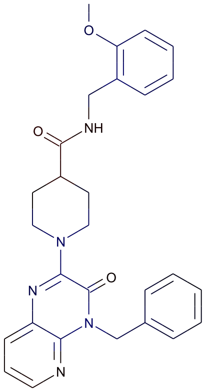

$C_{28}H_{29}N_5O_3$
Molecular Weight: 483.56156
ALogP: 2.942
Rotatable Bonds: 7
Acceptors: 6
Donors: 1

Structural Similar Compounds

Name	Moricizine	Fluticasone	Diltiazem
Structure	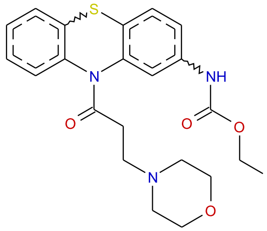	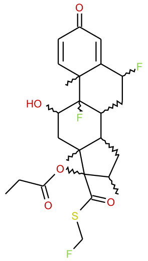	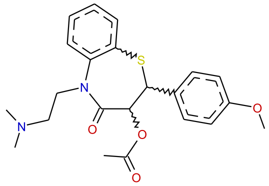
Actual Endpoint	Non-Carcinogen	Non-Carcinogen	Non-Carcinogen
Predicted Endpoint	Non-Carcinogen	Carcinogen	Non-Carcinogen
Distance	0.579	0.609	0.611
Reference	US FDA (Centre for Drug Eval.& Res./Off. Testing & Res.) Sept. 1997	US FDA (Centre for Drug Eval.& Res./Off. Testing & Res.) Sept. 1997	US FDA (Centre for Drug Eval.& Res./Off. Testing & Res.) Sept. 1997

Model Prediction

Prediction: Non-Carcinogen
Probability: 0.186
Enrichment: 0.633
Bayesian Score: -5.88
Mahalanobis Distance: 15.2
Mahalanobis Distance p-value: 3.02e-008

Prediction: Positive if the Bayesian score is above the estimated best cutoff value from minimizing the false positive and false negative rate.
Probability: The esimated probability that the sample is in the positive category. This assumes that the Bayesian score follows a normal distribution and is different from the prediction using a cutoff.
Enrichment: An estimate of enrichment, that is, the increased likelihood (versus random) of this sample being in the category.
Bayesian Score: The standard Laplacian-modified Bayesian score.
Mahalanobis Distance: The Mahalanobis distance (MD) is the distance to the center of the training data. The larger the MD, the less trustworthy the prediction.
Mahalanobis Distance p-value: The p-value gives the fraction of training data with an MD greater than or equal to the one for the given sample, assuming normally distributed data. The smaller the p-value, the less trustworthy the prediciton. For highly non-normal X properties (e.g., fingerprints), the MD p-value is wildly inaccurate.

Model Applicability

Unknown features are fingerprint features in the query molecule, but not found or appearing too infrequently in the training set.

1. All properties and OPS components are within expected ranges.

Feature Contribution

Top features for positive contribution

Fingerprint	Bit/Smiles	Feature Structure	Score	Carcinogen in training set
FCFP_6	566058135	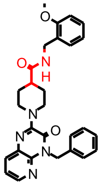 [*]NC(=O)C([*])[*]	0.447	17 out of 40

FCFP_6	-1043339860	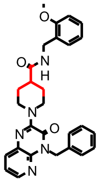 [*]CC(C[*])C(=[*])[*]	0.383	24 out of 61
FCFP_6	-1498803602	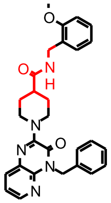 [*]CNC(=O)C(C[*])C[*]	0.271	1 out of 2

Top Features for negative contribution

Fingerprint	Bit/Smiles	Feature Structure	Score	Carcinogen in training set
FCFP_6	1743817318	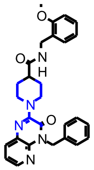 [*]\N=C(\N1CC[*]CC1)/ C(=[*])[*]	-0.582	0 out of 3
FCFP_6	-1553874037	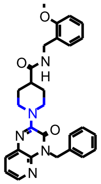 [*]CN(C[*])C(=[*])[*]	-0.45	5 out of 32
FCFP_6	-1474971978	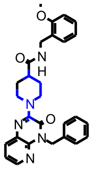 [*]C1[*]CN(CC1)C(=[*])[*]	-0.439	1 out of 8

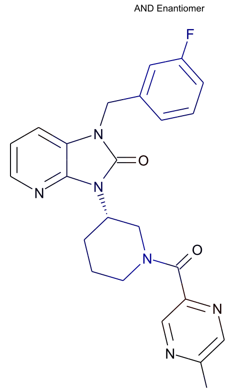

$C_{24}H_{23}FN_6O_2$
Molecular Weight: 446.47682
ALogP: 2.244
Rotatable Bonds: 4
Acceptors: 5
Donors: 0

Structural Similar Compounds

Name	Risperidone	Levocabstine	Buspirone
Structure	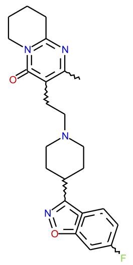	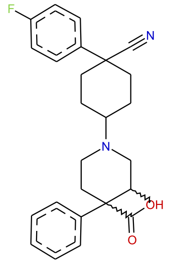	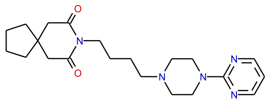
Actual Endpoint	Non-Carcinogen	Non-Carcinogen	Non-Carcinogen
Predicted Endpoint	Non-Carcinogen	Non-Carcinogen	Non-Carcinogen
Distance	0.587	0.618	0.619
Reference	US FDA (Centre for Drug Eval.& Res./Off. Testing & Res.) Sept. 1997	US FDA (Centre for Drug Eval.& Res./Off. Testing & Res.) Sept. 1997	US FDA (Centre for Drug Eval.& Res./Off. Testing & Res.) Sept. 1997

Model Applicability

Unknown features are fingerprint features in the query molecule, but not found or appearing too infrequently in the training set.

1. OPS PC30 out of range. Value: -2.7889. Training min, max, SD, explained variance: -2.5648, 3.3898, 0.9347, 0.0092.

Model Prediction

Prediction: Non-Carcinogen
Probability: 0.176
Enrichment: 0.596
Bayesian Score: -6.69
Mahalanobis Distance: 14.4
Mahalanobis Distance p-value: 1.41e-006

Prediction: Positive if the Bayesian score is above the estimated best cutoff value from minimizing the false positive and false negative rate.
Probability: The esimated probability that the sample is in the positive category. This assumes that the Bayesian score follows a normal distribution and is different from the prediction using a cutoff.
Enrichment: An estimate of enrichment, that is, the increased likelihood (versus random) of this sample being in the category.
Bayesian Score: The standard Laplacian-modified Bayesian score.
Mahalanobis Distance: The Mahalanobis distance (MD) is the distance to the center of the training data. The larger the MD, the less trustworthy the prediction.
Mahalanobis Distance p-value: The p-value gives the fraction of training data with an MD greater than or equal to the one for the given sample, assuming normally distributed data. The smaller the p-value, the less trustworthy the prediciton. For highly non-normal X properties (e.g., fingerprints), the MD p-value is wildly inaccurate.

Feature Contribution

Top features for positive contribution

Fingerprint	Bit/Smiles	Feature Structure	Score	Carcinogen in training set
FCFP_6	-1462709112	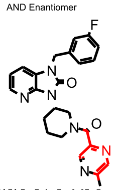 [*]C(=[*])[c]1:[cH]:[*]:[c]([*]):[cH]:n:1	0.367	5 out of 12

FCFP_6	-989213044	AND Enantiomer<br>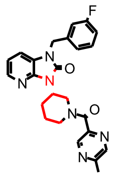<br>[*]N([*])[C@@H]1C[*]C<br>CC1	0.348	6 out of 15
FCFP_6	-1539132615	AND Enantiomer<br>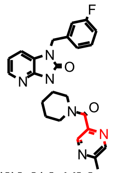<br>[*]C(=[*])[c](:[cH]:[<br>*]):n:[*]	0.328	19 out of 51
**Top Features for negative contribution**				
**Fingerprint**	**Bit/Smiles**	**Feature Structure**	**Score**	**Carcinogen in training set**
FCFP_6	-98332825	AND Enantiomer<br>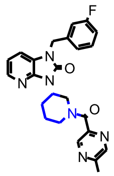<br>[*][C@@H]1[*]N([*])CC<br>C1	-0.793	1 out of 13
FCFP_6	-823454507	AND Enantiomer<br>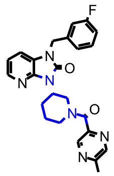<br>[*]N([*])[C@H]1CCCN(C<br>1)C(=[*])[*]	-0.719	0 out of 4
FCFP_6	-1553874037	AND Enantiomer<br>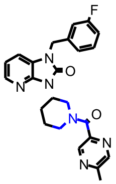<br>[*]CN(C[*])C(=[*])[*]	-0.45	5 out of 32

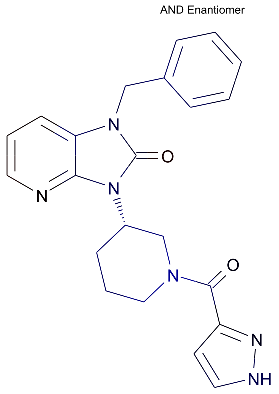

$C_{22}H_{22}N_6O_2$
Molecular Weight: 402.44907
ALogP: 2.501
Rotatable Bonds: 4
Acceptors: 4
Donors: 1

Model Prediction

Prediction: Non-Carcinogen
Probability: 0.177
Enrichment: 0.602
Bayesian Score: -6.57
Mahalanobis Distance: 13
Mahalanobis Distance p-value: 0.00029

Prediction: Positive if the Bayesian score is above the estimated best cutoff value from minimizing the false positive and false negative rate.
Probability: The esimated probability that the sample is in the positive category. This assumes that the Bayesian score follows a normal distribution and is different from the prediction using a cutoff.
Enrichment: An estimate of enrichment, that is, the increased likelihood (versus random) of this sample being in the category.
Bayesian Score: The standard Laplacian-modified Bayesian score.
Mahalanobis Distance: The Mahalanobis distance (MD) is the distance to the center of the training data. The larger the MD, the less trustworthy the prediction.
Mahalanobis Distance p-value: The p-value gives the fraction of training data with an MD greater than or equal to the one for the given sample, assuming normally distributed data. The smaller the p-value, the less trustworthy the prediciton. For highly non-normal X properties (e.g., fingerprints), the MD p-value is wildly inaccurate.

Structural Similar Compounds

Name	Levocabstine	Lansoprazole	Omeprazole
Structure	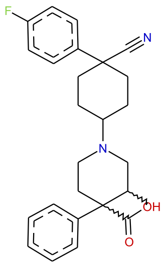	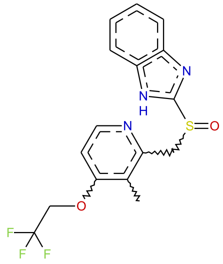	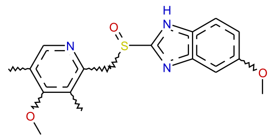
Actual Endpoint	Non-Carcinogen	Carcinogen	Non-Carcinogen
Predicted Endpoint	Non-Carcinogen	Carcinogen	Non-Carcinogen
Distance	0.552	0.603	0.605
Reference	US FDA (Centre for Drug Eval.& Res./Off. Testing & Res.) Sept. 1997	US FDA (Centre for Drug Eval.& Res./Off. Testing & Res.) Sept. 1997	US FDA (Centre for Drug Eval.& Res./Off. Testing & Res.) Sept. 1997

Model Applicability

Unknown features are fingerprint features in the query molecule, but not found or appearing too infrequently in the training set.

1. All properties and OPS components are within expected ranges.

Feature Contribution

Top features for positive contribution

Fingerprint	Bit/Smiles	Feature Structure	Score	Carcinogen in training set
FCFP_6	-989213044	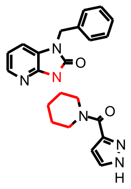 AND Enantiomer [*]N([*])[C@@H]1C[*]C CC1	0.348	6 out of 15

FCFP_6	-1539132615	AND Enantiomer 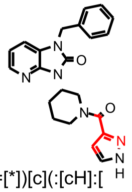 [*]C(=[*])[c](:[cH]:[*]):n:[*]	0.328	19 out of 51
FCFP_6	1390842262	AND Enantiomer 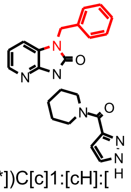 [*]N([*])C[c]1:[cH]:[cH]:[cH]:[cH]:[cH]:1	0.271	1 out of 2

Top Features for negative contribution

Fingerprint	Bit/Smiles	Feature Structure	Score	Carcinogen in training set
FCFP_6	-98332825	AND Enantiomer 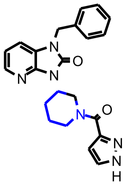 [*][C@@H]1[*]N([*])CCC1	-0.793	1 out of 13
FCFP_6	-823454507	AND Enantiomer 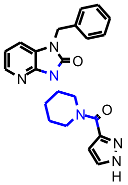 [*]N([*])[C@H]1CCCN(C1)C(=[*])[*]	-0.719	0 out of 4
FCFP_6	1618184456	AND Enantiomer 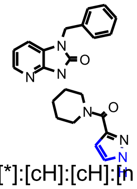 [*]1:[*]:[cH]:[cH]:[nH]:1	-0.719	0 out of 4

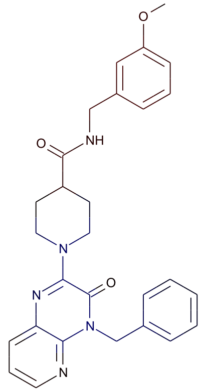

$C_{28}H_{29}N_5O_3$

Molecular Weight: 483.56156

ALogP: 2.942

Rotatable Bonds: 7

Acceptors: 6

Donors: 1

Model Prediction

Prediction: Non-Carcinogen

Probability: 0.227

Enrichment: 0.772

Bayesian Score: -3.41

Mahalanobis Distance: 14.9

Mahalanobis Distance p-value: 1.17e-007

Prediction: Positive if the Bayesian score is above the estimated best cutoff value from minimizing the false positive and false negative rate.
Probability: The esimated probability that the sample is in the positive category. This assumes that the Bayesian score follows a normal distribution and is different from the prediction using a cutoff.
Enrichment: An estimate of enrichment, that is, the increased likelihood (versus random) of this sample being in the category.
Bayesian Score: The standard Laplacian-modified Bayesian score.
Mahalanobis Distance: The Mahalanobis distance (MD) is the distance to the center of the training data. The larger the MD, the less trustworthy the prediction.
Mahalanobis Distance p-value: The p-value gives the fraction of training data with an MD greater than or equal to the one for the given sample, assuming normally distributed data. The smaller the p-value, the less trustworthy the prediciton. For highly non-normal X properties (e.g., fingerprints), the MD p-value is wildly inaccurate.

Structural Similar Compounds

Name	Moricizine	Fluticasone	Diltiazem
Structure	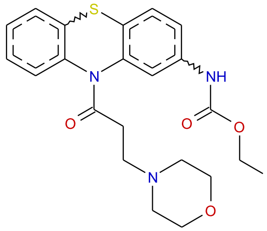	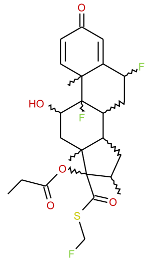	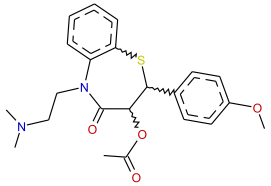
Actual Endpoint	Non-Carcinogen	Non-Carcinogen	Non-Carcinogen
Predicted Endpoint	Non-Carcinogen	Carcinogen	Non-Carcinogen
Distance	0.584	0.610	0.612
Reference	US FDA (Centre for Drug Eval.& Res./Off. Testing & Res.) Sept. 1997	US FDA (Centre for Drug Eval.& Res./Off. Testing & Res.) Sept. 1997	US FDA (Centre for Drug Eval.& Res./Off. Testing & Res.) Sept. 1997

Model Applicability

Unknown features are fingerprint features in the query molecule, but not found or appearing too infrequently in the training set.

1. OPS PC28 out of range. Value: 3.5487. Training min, max, SD, explained variance: -2.6547, 3.4541, 0.9656, 0.0098.

Feature Contribution

Top features for positive contribution

Fingerprint	Bit/Smiles	Feature Structure	Score	Carcinogen in training set
FCFP_6	-1757681964	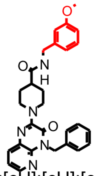 [*]C[c]1:[cH]:[cH]:[cH]:[c](OC):[cH]:1	0.676	2 out of 2

FCFP_6	566058135	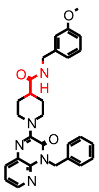 [*]NC(=O)C([*])[*]	0.447	17 out of 40
FCFP_6	-1043339860	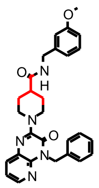 [*]CC(C[*])C(=[*])[*]	0.383	24 out of 61
**Top Features for negative contribution**				
**Fingerprint**	**Bit/Smiles**	**Feature Structure**	**Score**	**Carcinogen in training set**
FCFP_6	1743817318	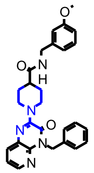 [*]\N=C(\N1CC[*]CC1)/C(=[*])[*]	-0.582	0 out of 3
FCFP_6	-1553874037	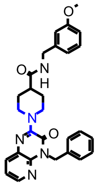 [*]CN(C[*])C(=[*])[*]	-0.45	5 out of 32
FCFP_6	-1474971978	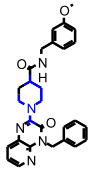 [*]C1[*]CN(CC1)C(=[*])[*]	-0.439	1 out of 8

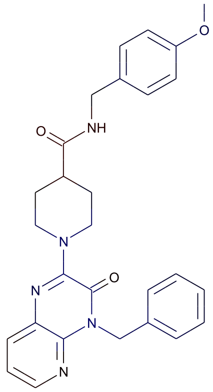

$C_{28}H_{29}N_5O_3$
Molecular Weight: 483.56156
ALogP: 2.942
Rotatable Bonds: 7
Acceptors: 6
Donors: 1

Model Prediction

Prediction: Non-Carcinogen
Probability: 0.193
Enrichment: 0.655
Bayesian Score: -5.45
Mahalanobis Distance: 14.6
Mahalanobis Distance p-value: 6.44e-007

Prediction: Positive if the Bayesian score is above the estimated best cutoff value from minimizing the false positive and false negative rate.
Probability: The esimated probability that the sample is in the positive category. This assumes that the Bayesian score follows a normal distribution and is different from the prediction using a cutoff.
Enrichment: An estimate of enrichment, that is, the increased likelihood (versus random) of this sample being in the category.
Bayesian Score: The standard Laplacian-modified Bayesian score.
Mahalanobis Distance: The Mahalanobis distance (MD) is the distance to the center of the training data. The larger the MD, the less trustworthy the prediction.
Mahalanobis Distance p-value: The p-value gives the fraction of training data with an MD greater than or equal to the one for the given sample, assuming normally distributed data. The smaller the p-value, the less trustworthy the prediciton. For highly non-normal X properties (e.g., fingerprints), the MD p-value is wildly inaccurate.

Structural Similar Compounds

Name	Moricizine	Diltiazem	Fluticasone
Structure	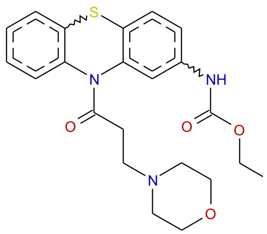	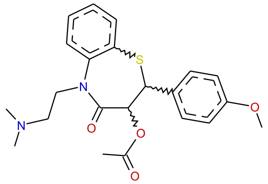	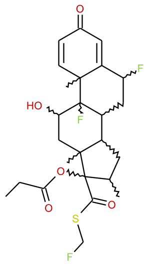
Actual Endpoint	Non-Carcinogen	Non-Carcinogen	Non-Carcinogen
Predicted Endpoint	Non-Carcinogen	Non-Carcinogen	Carcinogen
Distance	0.583	0.600	0.609
Reference	US FDA (Centre for Drug Eval.& Res./Off. Testing & Res.) Sept. 1997	US FDA (Centre for Drug Eval.& Res./Off. Testing & Res.) Sept. 1997	US FDA (Centre for Drug Eval.& Res./Off. Testing & Res.) Sept. 1997

Model Applicability

Unknown features are fingerprint features in the query molecule, but not found or appearing too infrequently in the training set.

1. All properties and OPS components are within expected ranges.

Feature Contribution

Top features for positive contribution

Fingerprint	Bit/Smiles	Feature Structure	Score	Carcinogen in training set
FCFP_6	566058135	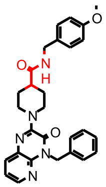 [*]NC(=O)C([*])[*]	0.447	17 out of 40

FCFP_6	-1043339860	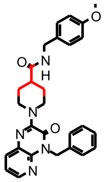 [*]CC(C[*])C(=[*])[*]	0.383	24 out of 61
FCFP_6	1390842262	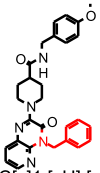 [*]N([*])C[c]1:[cH]:[cH]:[cH]:[cH]:[cH]:1	0.271	1 out of 2
**Top Features for negative contribution**				
**Fingerprint**	**Bit/Smiles**	**Feature Structure**	**Score**	**Carcinogen in training set**
FCFP_6	-9847677	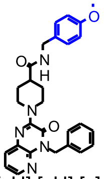 [*][c]1:[cH]:[cH]:[c] (OC):[cH]:[cH]:1	-0.719	0 out of 4
FCFP_6	1743817318	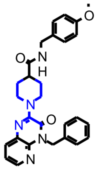 [*]\N=C(\N1CC[*]CC1)/ C(=[*])[*]	-0.582	0 out of 3
FCFP_6	-1553874037	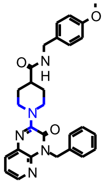 [*]CN(C[*])C(=[*])[*]	-0.45	5 out of 32

Molecule

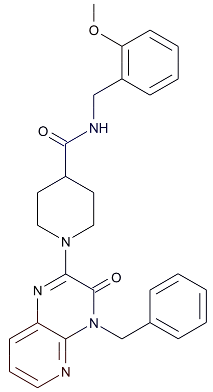

$C_{28}H_{29}N_5O_3$
Molecular Weight: 483.56156
ALogP: 2.942
Rotatable Bonds: 7
Acceptors: 6
Donors: 1

Model Prediction

Prediction: Non-Carcinogen
Probability: 0.479
Enrichment: 1.22
Bayesian Score: -2.62
Mahalanobis Distance: 11.5
Mahalanobis Distance p-value: 2.07e-005

Prediction: Positive if the Bayesian score is above the estimated best cutoff value from minimizing the false positive and false negative rate.
Probability: The esimated probability that the sample is in the positive category. This assumes that the Bayesian score follows a normal distribution and is different from the prediction using a cutoff.
Enrichment: An estimate of enrichment, that is, the increased likelihood (versus random) of this sample being in the category.
Bayesian Score: The standard Laplacian-modified Bayesian score.
Mahalanobis Distance: The Mahalanobis distance (MD) is the distance to the center of the training data. The larger the MD, the less trustworthy the prediction.
Mahalanobis Distance p-value: The p-value gives the fraction of training data with an MD greater than or equal to the one for the given sample, assuming normally distributed data. The smaller the p-value, the less trustworthy the prediciton. For highly non-normal X properties (e.g., fingerprints), the MD p-value is wildly inaccurate.

Structural Similar Compounds

Name	Curcumin	RHODAMINE 6G	ROTENONE
Structure	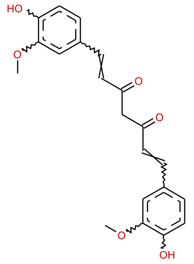	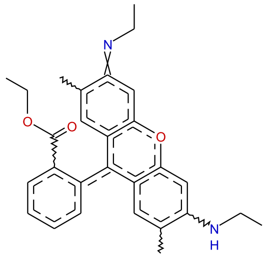	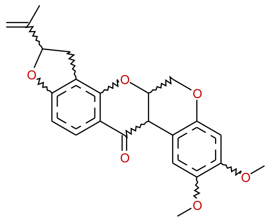
Actual Endpoint	Carcinogen	Non-Carcinogen	Non-Carcinogen
Predicted Endpoint	Carcinogen	Non-Carcinogen	Non-Carcinogen
Distance	0.690	0.732	0.773
Reference	NTP427	NTP/TR-364	NTP/TR-320

Model Applicability

Unknown features are fingerprint features in the query molecule, but not found or appearing too infrequently in the training set.

1. OPS PC11 out of range. Value: 5.7128. Training min, max, SD, explained variance: -3.6964, 3.2756, 1.387, 0.0309.

Feature Contribution

Top features for positive contribution

Fingerprint	Bit/Smiles	Feature Structure	Score	Carcinogen in training set
SCFP_12	1190896200	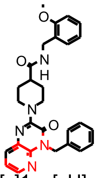 [*]N([*])[c]1:n:[cH]:[cH]:[*]:[c]:1[*]	0.377	1 out of 1

SCFP_12	-758850909	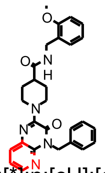 [*][c]1:[*]:n:[cH]:[cH]:[cH]:1	0.329	4 out of 7
SCFP_12	-937094999	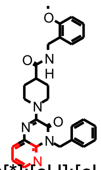 [*][c]1:[*]:[cH]:[cH]:[cH]:n:1	0.231	4 out of 8

Top Features for negative contribution

Fingerprint	Bit/Smiles	Feature Structure	Score	Carcinogen in training set
SCFP_12	-111024397	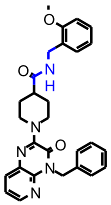 [*]CNC(=[*])[*]	-0.784	2 out of 15
SCFP_12	9	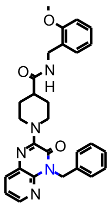 [*]N([*])[*]	-0.613	9 out of 47
SCFP_12	1257024795	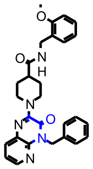 [*]N([*])C(=O)C(=[*])[*]	-0.356	1 out of 5

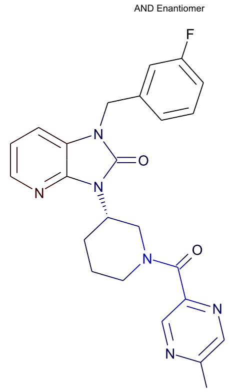

$C_{24}H_{23}FN_6O_2$
Molecular Weight: 446.47682
ALogP: 2.244
Rotatable Bonds: 4
Acceptors: 5
Donors: 0

Model Prediction

Prediction: Non-Carcinogen
Probability: 0.133
Enrichment: 0.339
Bayesian Score: -6.52
Mahalanobis Distance: 10.8
Mahalanobis Distance p-value: 0.00035

Prediction: Positive if the Bayesian score is above the estimated best cutoff value from minimizing the false positive and false negative rate.
Probability: The esimated probability that the sample is in the positive category. This assumes that the Bayesian score follows a normal distribution and is different from the prediction using a cutoff.
Enrichment: An estimate of enrichment, that is, the increased likelihood (versus random) of this sample being in the category.
Bayesian Score: The standard Laplacian-modified Bayesian score.
Mahalanobis Distance: The Mahalanobis distance (MD) is the distance to the center of the training data. The larger the MD, the less trustworthy the prediction.
Mahalanobis Distance p-value: The p-value gives the fraction of training data with an MD greater than or equal to the one for the given sample, assuming normally distributed data. The smaller the p-value, the less trustworthy the prediciton. For highly non-normal X properties (e.g., fingerprints), the MD p-value is wildly inaccurate.

Structural Similar Compounds

Name	ROTENONE	Rotenone	TETRACHLORVINPHOS
Structure	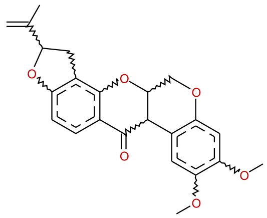	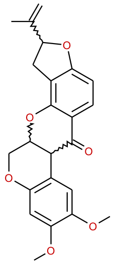	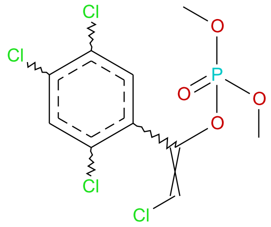
Actual Endpoint	Non-Carcinogen	Non-Carcinogen	Carcinogen
Predicted Endpoint	Non-Carcinogen	Non-Carcinogen	Carcinogen
Distance	0.675	0.675	0.686
Reference	NTP/TR-320	NTP320	NTP/TR-33

Model Applicability

Unknown features are fingerprint features in the query molecule, but not found or appearing too infrequently in the training set.

1. All properties and OPS components are within expected ranges.

Feature Contribution

Top features for positive contribution				
**Fingerprint**	**Bit/Smiles**	**Feature Structure**	**Score**	**Carcinogen in training set**
SCFP_12	1190896200	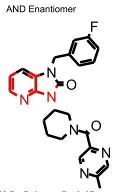 [*]N([*])[c]1:n:[cH]:[cH]:[*]:[c]:1[*]	0.377	1 out of 1

SCFP_12	-758850909	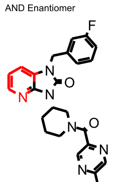 [*][c]1:[*]:n:[cH]:[cH]:[cH]:1	0.329	4 out of 7
SCFP_12	-937094999	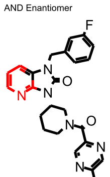 [*][c]1:[*]:[cH]:[cH]:[cH]:n:1	0.231	4 out of 8

Top Features for negative contribution

**Fingerprint**	**Bit/Smiles**	**Feature Structure**	**Score**	**Carcinogen in training set**
SCFP_12	1256786467	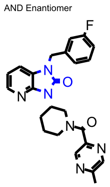 [*]N1[*]:[*]N([*])C1=O	-1.07	1 out of 13
SCFP_12	-1343150366	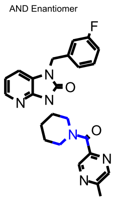 [*]CN(C[*])C(=[*])[*]	-1.05	0 out of 5
SCFP_12	-587569116	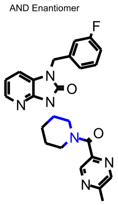 [*]C([*])CN([*])[*]	-0.91	0 out of 4

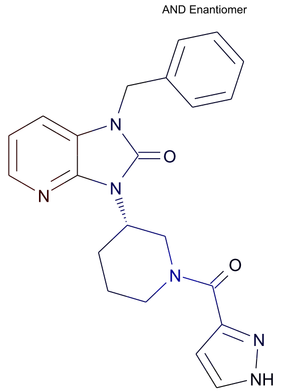

$C_{22}H_{22}N_6O_2$
Molecular Weight: 402.44907
ALogP: 2.501
Rotatable Bonds: 4
Acceptors: 4
Donors: 1

Model Prediction

Prediction: Non-Carcinogen
Probability: 0.166
Enrichment: 0.423
Bayesian Score: -6.08
Mahalanobis Distance: 10.1
Mahalanobis Distance p-value: 0.00353

Prediction: Positive if the Bayesian score is above the estimated best cutoff value from minimizing the false positive and false negative rate.
Probability: The esimated probability that the sample is in the positive category. This assumes that the Bayesian score follows a normal distribution and is different from the prediction using a cutoff.
Enrichment: An estimate of enrichment, that is, the increased likelihood (versus random) of this sample being in the category.
Bayesian Score: The standard Laplacian-modified Bayesian score.
Mahalanobis Distance: The Mahalanobis distance (MD) is the distance to the center of the training data. The larger the MD, the less trustworthy the prediction.
Mahalanobis Distance p-value: The p-value gives the fraction of training data with an MD greater than or equal to the one for the given sample, assuming normally distributed data. The smaller the p-value, the less trustworthy the prediciton. For highly non-normal X properties (e.g., fingerprints), the MD p-value is wildly inaccurate.

Structural Similar Compounds

Name	CHLORENDIC ACID	Chlorendic Acid	TOLAZAMIDE
Structure	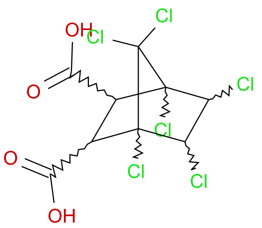	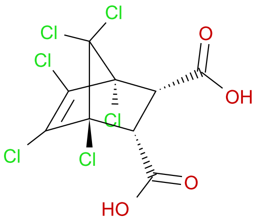	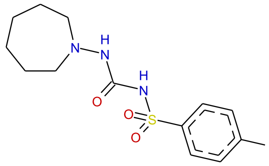
Actual Endpoint	Carcinogen	Carcinogen	Non-Carcinogen
Predicted Endpoint	Carcinogen	Carcinogen	Non-Carcinogen
Distance	0.661	0.661	0.665
Reference	NTP/TR-304	NTP304	NTP/TR-51

Model Applicability

Unknown features are fingerprint features in the query molecule, but not found or appearing too infrequently in the training set.

1. OPS PC11 out of range. Value: 3.3117. Training min, max, SD, explained variance: -3.6964, 3.2756, 1.387, 0.0309.

Feature Contribution

Top features for positive contribution

Fingerprint	Bit/Smiles	Feature Structure	Score	Carcinogen in training set
SCFP_12	1190896200	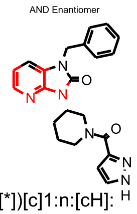 [*]N([*])[c]1:n:[cH]:[cH]:[*]:[c]:1[*]	0.377	1 out of 1

SCFP_12	-758850909	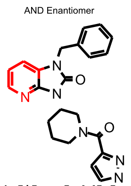 [*][c]1:[*]:n:[cH]:[cH]:[cH]:1	0.329	4 out of 7
SCFP_12	-937094999	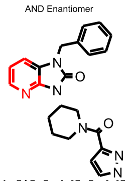 [*][c]1:[*]:[cH]:[cH]:[cH]:n:1	0.231	4 out of 8
**Top Features for negative contribution**				
**Fingerprint**	**Bit/Smiles**	**Feature Structure**	**Score**	**Carcinogen in training set**
SCFP_12	1256786467	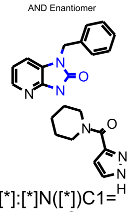 [*]N1[*]:[*]N([*])C1=O	-1.07	1 out of 13
SCFP_12	-1343150366	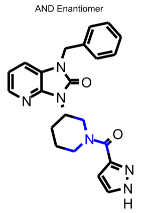 [*]CN(C[*])C(=[*])[*]	-1.05	0 out of 5
SCFP_12	-587569116	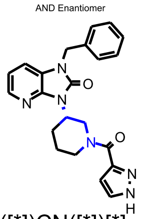 [*]C([*])CN([*])[*]	-0.91	0 out of 4

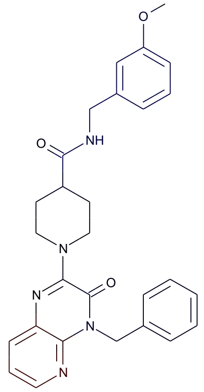

$C_{28}H_{29}N_5O_3$
Molecular Weight: 483.56156
ALogP: 2.942
Rotatable Bonds: 7
Acceptors: 6
Donors: 1

Model Prediction

Prediction: Non-Carcinogen
Probability: 0.371
Enrichment: 0.943
Bayesian Score: -3.89
Mahalanobis Distance: 11.5
Mahalanobis Distance p-value: 2.07e-005

Prediction: Positive if the Bayesian score is above the estimated best cutoff value from minimizing the false positive and false negative rate.
Probability: The esimated probability that the sample is in the positive category. This assumes that the Bayesian score follows a normal distribution and is different from the prediction using a cutoff.
Enrichment: An estimate of enrichment, that is, the increased likelihood (versus random) of this sample being in the category.
Bayesian Score: The standard Laplacian-modified Bayesian score.
Mahalanobis Distance: The Mahalanobis distance (MD) is the distance to the center of the training data. The larger the MD, the less trustworthy the prediction.
Mahalanobis Distance p-value: The p-value gives the fraction of training data with an MD greater than or equal to the one for the given sample, assuming normally distributed data. The smaller the p-value, the less trustworthy the prediciton. For highly non-normal X properties (e.g., fingerprints), the MD p-value is wildly inaccurate.

Structural Similar Compounds

Name	Curcumin	RHODAMINE 6G	Rotenone
Structure	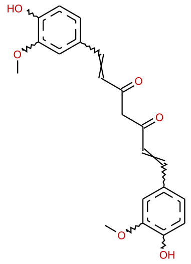	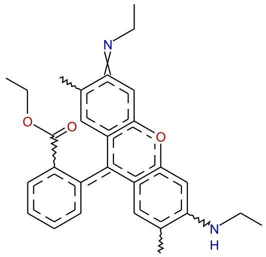	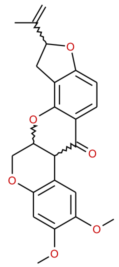
Actual Endpoint	Carcinogen	Non-Carcinogen	Non-Carcinogen
Predicted Endpoint	Carcinogen	Non-Carcinogen	Non-Carcinogen
Distance	0.691	0.732	0.774
Reference	NTP427	NTP/TR-364	NTP320

Model Applicability

Unknown features are fingerprint features in the query molecule, but not found or appearing too infrequently in the training set.

1. OPS PC11 out of range. Value: 5.7128. Training min, max, SD, explained variance: -3.6964, 3.2756, 1.387, 0.0309.

Feature Contribution

Top features for positive contribution

Fingerprint	Bit/Smiles	Feature Structure	Score	Carcinogen in training set
SCFP_12	1190896200	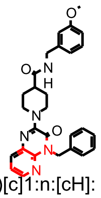 [*]N([*])[c]1:n:[cH]:[cH]:[*]:[c]:1[*]	0.377	1 out of 1

SCFP_12	-758850909	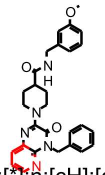 [*][c]1:[*]:n:[cH]:[cH]:[cH]:1	0.329	4 out of 7
SCFP_12	-937094999	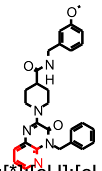 [*][c]1:[*]:[cH]:[cH]:[cH]:n:1	0.231	4 out of 8
**Top Features for negative contribution**				
**Fingerprint**	**Bit/Smiles**	**Feature Structure**	**Score**	**Carcinogen in training set**
SCFP_12	-1632615624	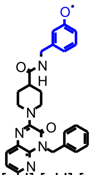 [*]C[c]1:[cH]:[cH]:[cH]:[c](O[*]):[cH]:1	-1.05	0 out of 5
SCFP_12	-111024397	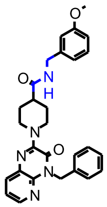 [*]CNC(=[*])[*]	-0.784	2 out of 15
SCFP_12	9	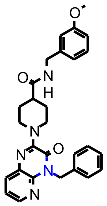 [*]N([*])[*]	-0.613	9 out of 47

#UNDEFINED

TOPKAT_Mouse_Male_NTP

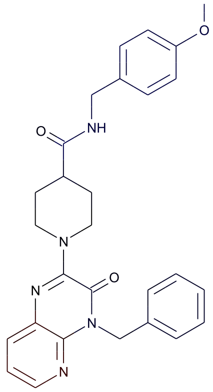

$C_{28}H_{29}N_5O_3$
Molecular Weight: 483.56156
ALogP: 2.942
Rotatable Bonds: 7
Acceptors: 6
Donors: 1

Structural Similar Compounds

Name	Curcumin	RHODAMINE 6G	TETRACHLORVINPHOS
Structure	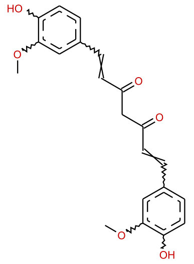	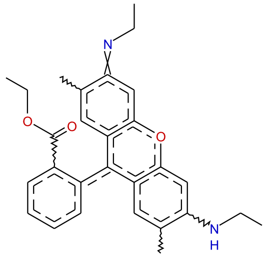	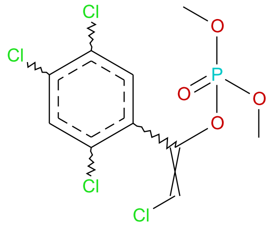
Actual Endpoint	Carcinogen	Non-Carcinogen	Carcinogen
Predicted Endpoint	Carcinogen	Non-Carcinogen	Carcinogen
Distance	0.691	0.732	0.776
Reference	NTP427	NTP/TR-364	NTP/TR-33

Model Prediction

Prediction: Non-Carcinogen
Probability: 0.359
Enrichment: 0.912
Bayesian Score: -4.02
Mahalanobis Distance: 11.5
Mahalanobis Distance p-value: 2.07e-005

Prediction: Positive if the Bayesian score is above the estimated best cutoff value from minimizing the false positive and false negative rate.
Probability: The esimated probability that the sample is in the positive category. This assumes that the Bayesian score follows a normal distribution and is different from the prediction using a cutoff.
Enrichment: An estimate of enrichment, that is, the increased likelihood (versus random) of this sample being in the category.
Bayesian Score: The standard Laplacian-modified Bayesian score.
Mahalanobis Distance: The Mahalanobis distance (MD) is the distance to the center of the training data. The larger the MD, the less trustworthy the prediction.
Mahalanobis Distance p-value: The p-value gives the fraction of training data with an MD greater than or equal to the one for the given sample, assuming normally distributed data. The smaller the p-value, the less trustworthy the prediciton. For highly non-normal X properties (e.g., fingerprints), the MD p-value is wildly inaccurate.

Model Applicability

Unknown features are fingerprint features in the query molecule, but not found or appearing too infrequently in the training set.

1. OPS PC11 out of range. Value: 5.7128. Training min, max, SD, explained variance: -3.6964, 3.2756, 1.387, 0.0309.

Feature Contribution

Top features for positive contribution

Fingerprint	Bit/Smiles	Feature Structure	Score	Carcinogen in training set
SCFP_12	1190896200	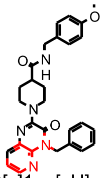 [*]N([*])[c]1:n:[cH]:[cH]:[*]:[c]:1[*]	0.377	1 out of 1

SCFP_12	-758850909	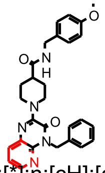<br>[*][c]1:[*]:n:[cH]:[cH]:[cH]:1	0.329	4 out of 7
SCFP_12	-937094999	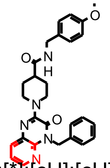<br>[*][c]1:[*]:[cH]:[cH]:[cH]:n:1	0.231	4 out of 8
**Top Features for negative contribution**				
**Fingerprint**	**Bit/Smiles**	**Feature Structure**	**Score**	**Carcinogen in training set**
SCFP_12	-111024397	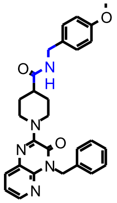<br>[*]CNC(=[*])[*]	-0.784	2 out of 15
SCFP_12	9	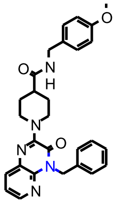<br>[*]N([*])[*]	-0.613	9 out of 47
SCFP_12	-1849095515	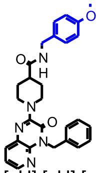<br>[*]C[c]1:[cH]:[cH]:[c](OC):[cH]:[cH]:1	-0.555	0 out of 2

Molecule

TOPKAT_Ocular_Irritancy_Mild_vs_Moderate_Severe

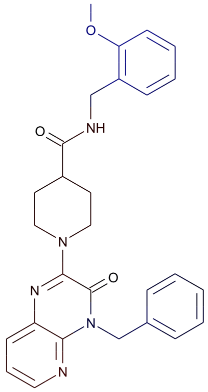

$C_{28}H_{29}N_5O_3$
Molecular Weight: 483.56156
ALogP: 2.942
Rotatable Bonds: 7
Acceptors: 6
Donors: 1

Model Prediction

Prediction: Mild
Probability: 0.778
Enrichment: 1.13
Bayesian Score: -1.75
Mahalanobis Distance: 12.8
Mahalanobis Distance p-value: 1.02e-006

Prediction: Positive if the Bayesian score is above the estimated best cutoff value from minimizing the false positive and false negative rate.
Probability: The esimated probability that the sample is in the positive category. This assumes that the Bayesian score follows a normal distribution and is different from the prediction using a cutoff.
Enrichment: An estimate of enrichment, that is, the increased likelihood (versus random) of this sample being in the category.
Bayesian Score: The standard Laplacian-modified Bayesian score.
Mahalanobis Distance: The Mahalanobis distance (MD) is the distance to the center of the training data. The larger the MD, the less trustworthy the prediction.
Mahalanobis Distance p-value: The p-value gives the fraction of training data with an MD greater than or equal to the one for the given sample, assuming normally distributed data. The smaller the p-value, the less trustworthy the prediciton. For highly non-normal X properties (e.g., fingerprints), the MD p-value is wildly inaccurate.

Structural Similar Compounds

Name	COLCHICINE	1-BENZOYLAMINO-4-METHOXY-5-CHLORANTHRAQUINONE	ANTHRAQUINONE; 1;1'-IMINODI-
Structure	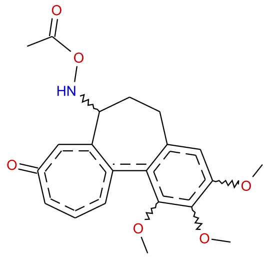	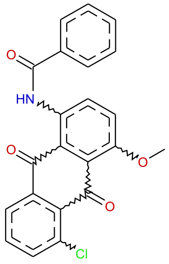	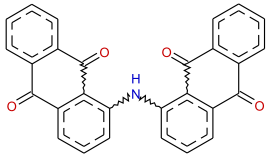
Actual Endpoint	Moderate_Severe	Mild	Mild
Predicted Endpoint	Moderate_Severe	Mild	Mild
Distance	0.673	0.750	0.780
Reference	AJOPAA 31;837;48	28ZPAK-;90;72	28ZPAK-;125;72

Model Applicability

Unknown features are fingerprint features in the query molecule, but not found or appearing too infrequently in the training set.

1. All properties and OPS components are within expected ranges.

Feature Contribution

Top features for positive contribution

Fingerprint	Bit/Smiles	Feature Structure	Score	Moderate_Severe in training set
FCFP_10	907096426	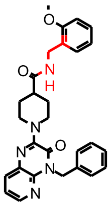 [*]NC[c](:[*]):[*]	0.332	5 out of 5

FCFP_10	-1695756380	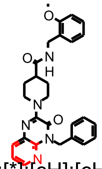 [*][c]1:[*]:[cH]:[cH]:[cH]:n:1	0.285	10 out of 11
FCFP_10	-124655670	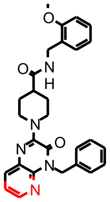 [*]:[cH]:[cH]:n:[*]	0.259	14 out of 16

Top Features for negative contribution

**Fingerprint**	**Bit/Smiles**	**Feature Structure**	**Score**	**Moderate_Severe in training set**
FCFP_10	1458856986	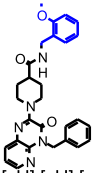 [*]C[c]1:[cH]:[cH]:[cH]:[cH]:[c]:1OC	-0.842	0 out of 2
FCFP_10	-1977641857	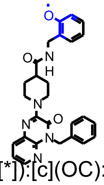 [*][c](:[*]):[c](OC):[cH]:[*]	-0.78	4 out of 15
FCFP_10	907036844	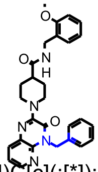 [*]N([*])C[c](:[*]):[*]	-0.6	1 out of 4

#UNDEFINED

TOPKAT_Ocular_Irritancy_Mild_vs_Moderate_Severe

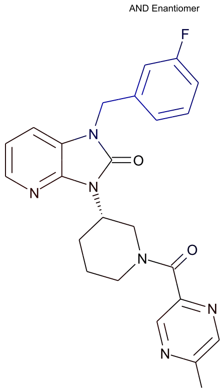

$C_{24}H_{23}FN_6O_2$
Molecular Weight: 446.47682
ALogP: 2.244
Rotatable Bonds: 4
Acceptors: 5
Donors: 0

Structural Similar Compounds

Name	1;8;9-ANTHRACENETRIOL; TRIACETATE	PROPANE;2;2-BIS(P-2;3-EPOXYPROPOXY)PHENYL)-	N(2-ETHYL BUTOXY ETHOXY-PROPYL)BICYCLO(2.2.1)HEPTENE-2;3-DICARBOXIMIDE
Structure	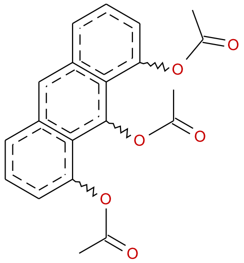	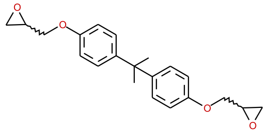	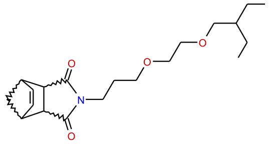
Actual Endpoint	Moderate_Severe	Moderate_Severe	Moderate_Severe
Predicted Endpoint	Moderate_Severe	Moderate_Severe	Moderate_Severe
Distance	0.687	0.752	0.760
Reference	BJOPAL 53;819;69	28ZPAK-;137;72	AIHAAP 23;95;62

Model Prediction

Prediction: Mild
Probability: 0.776
Enrichment: 1.13
Bayesian Score: -1.82
Mahalanobis Distance: 12.1
Mahalanobis Distance p-value: 3.25e-005

Prediction: Positive if the Bayesian score is above the estimated best cutoff value from minimizing the false positive and false negative rate.
Probability: The esimated probability that the sample is in the positive category. This assumes that the Bayesian score follows a normal distribution and is different from the prediction using a cutoff.
Enrichment: An estimate of enrichment, that is, the increased likelihood (versus random) of this sample being in the category.
Bayesian Score: The standard Laplacian-modified Bayesian score.
Mahalanobis Distance: The Mahalanobis distance (MD) is the distance to the center of the training data. The larger the MD, the less trustworthy the prediction.
Mahalanobis Distance p-value: The p-value gives the fraction of training data with an MD greater than or equal to the one for the given sample, assuming normally distributed data. The smaller the p-value, the less trustworthy the prediciton. For highly non-normal X properties (e.g., fingerprints), the MD p-value is wildly inaccurate.

Model Applicability

Unknown features are fingerprint features in the query molecule, but not found or appearing too infrequently in the training set.

1. All properties and OPS components are within expected ranges.

Feature Contribution

Top features for positive contribution

Fingerprint	Bit/Smiles	Feature Structure	Score	Moderate_Severe in training set
FCFP_10	-745491832	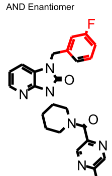 [*][c]1:[*]:[cH]:[cH]:[c](F):[cH]:1	0.304	29 out of 32

FCFP_10	-1695756380	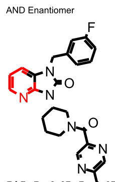 [*][c]1:[*]:[cH]:[cH]:[cH]:n:1	0.285	10 out of 11
FCFP_10	-1474971978	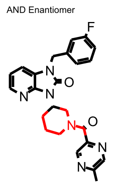 [*]C1[*]CN(CC1)C(=[*])[*]	0.259	14 out of 16
**Top Features for negative contribution**				
**Fingerprint**	**Bit/Smiles**	**Feature Structure**	**Score**	**Moderate_Severe in training set**
FCFP_10	-1700637232	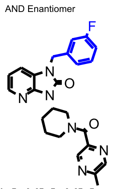 [*]C[c]1:[cH]:[cH]:[cH]:[c](F):[cH]:1	-1.34	1 out of 10
FCFP_10	-1549163031	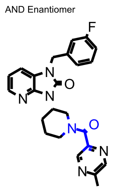 [*]N([*])C(=O)[c](:[*]):[*]	-0.657	5 out of 16
FCFP_10	907036844	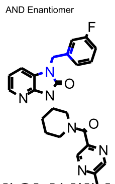 [*]N([*])C[c](:[*]):[*]	-0.6	1 out of 4

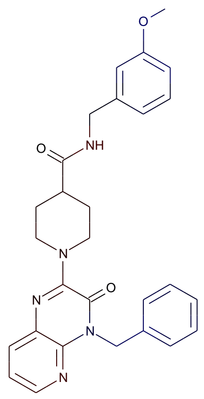

$C_{28}H_{29}N_5O_3$
Molecular Weight: 483.56156
ALogP: 2.942
Rotatable Bonds: 7
Acceptors: 6
Donors: 1

Model Prediction

Prediction: Mild
Probability: 0.804
Enrichment: 1.17
Bayesian Score: -0.851
Mahalanobis Distance: 12.8
Mahalanobis Distance p-value: 1.02e-006

Prediction: Positive if the Bayesian score is above the estimated best cutoff value from minimizing the false positive and false negative rate.
Probability: The esimated probability that the sample is in the positive category. This assumes that the Bayesian score follows a normal distribution and is different from the prediction using a cutoff.
Enrichment: An estimate of enrichment, that is, the increased likelihood (versus random) of this sample being in the category.
Bayesian Score: The standard Laplacian-modified Bayesian score.
Mahalanobis Distance: The Mahalanobis distance (MD) is the distance to the center of the training data. The larger the MD, the less trustworthy the prediction.
Mahalanobis Distance p-value: The p-value gives the fraction of training data with an MD greater than or equal to the one for the given sample, assuming normally distributed data. The smaller the p-value, the less trustworthy the prediciton. For highly non-normal X properties (e.g., fingerprints), the MD p-value is wildly inaccurate.

Structural Similar Compounds

Name	COLCHICINE	1-BENZOYLAMINO-4-METHOXY-5-CHLORANTHRAQUINONE	ANTHRAQUINONE; 1;1'-IMINODI-
Structure	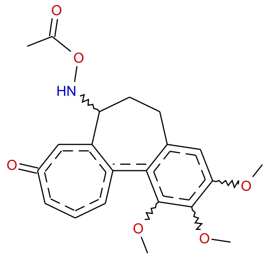	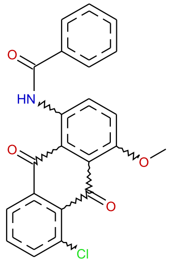	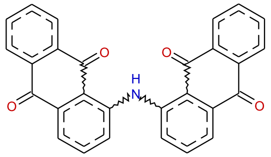
Actual Endpoint	Moderate_Severe	Mild	Mild
Predicted Endpoint	Moderate_Severe	Mild	Mild
Distance	0.671	0.754	0.781
Reference	AJOPAA 31;837;48	28ZPAK-;90;72	28ZPAK-;125;72

Model Applicability

Unknown features are fingerprint features in the query molecule, but not found or appearing too infrequently in the training set.

1. All properties and OPS components are within expected ranges.

Feature Contribution

Top features for positive contribution

Fingerprint	Bit/Smiles	Feature Structure	Score	Moderate_Severe in training set
FCFP_10	907096426	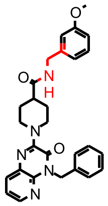 [*]NC[c](:[*]):[*]	0.332	5 out of 5

FCFP_10	427906732	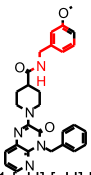 [*]NC[c]1:[cH]:[cH]:[*]:[c]([*]):[cH]:1	0.294	3 out of 3
FCFP_10	-1695756380	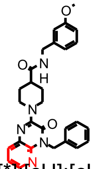 [*][c]1:[*]:[cH]:[cH]:[cH]:n:1	0.285	10 out of 11
**Top Features for negative contribution**				
**Fingerprint**	**Bit/Smiles**	**Feature Structure**	**Score**	**Moderate_Severe in training set**
FCFP_10	-1977641857	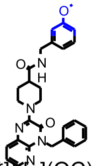 [*][c](:[*]):[c](OC):[cH]:[*]	-0.78	4 out of 15
FCFP_10	-1695347203	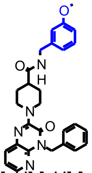 [*]C[c]1:[cH]:[cH]:[cH]:[c](O[*]):[cH]:1	-0.6	1 out of 4
FCFP_10	907036844	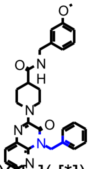 [*]N([*])C[c](:[*]):[*]	-0.6	1 out of 4

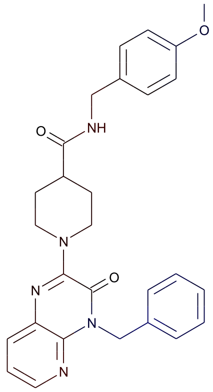

$C_{28}H_{29}N_5O_3$
Molecular Weight: 483.56156
ALogP: 2.942
Rotatable Bonds: 7
Acceptors: 6
Donors: 1

Model Prediction

Prediction: Moderate_Severe
Probability: 0.821
Enrichment: 1.19
Bayesian Score: -0.0497
Mahalanobis Distance: 12.8
Mahalanobis Distance p-value: 1.02e-006

Prediction: Positive if the Bayesian score is above the estimated best cutoff value from minimizing the false positive and false negative rate.
Probability: The esimated probability that the sample is in the positive category. This assumes that the Bayesian score follows a normal distribution and is different from the prediction using a cutoff.
Enrichment: An estimate of enrichment, that is, the increased likelihood (versus random) of this sample being in the category.
Bayesian Score: The standard Laplacian-modified Bayesian score.
Mahalanobis Distance: The Mahalanobis distance (MD) is the distance to the center of the training data. The larger the MD, the less trustworthy the prediction.
Mahalanobis Distance p-value: The p-value gives the fraction of training data with an MD greater than or equal to the one for the given sample, assuming normally distributed data. The smaller the p-value, the less trustworthy the prediciton. For highly non-normal X properties (e.g., fingerprints), the MD p-value is wildly inaccurate.

Structural Similar Compounds

Name	COLCHICINE	1-BENZOYLAMINO-4-METHOXY-5-CHLORANTHRAQUINONE	ANTHRAQUINONE; 1;1'-IMINODI-
Structure	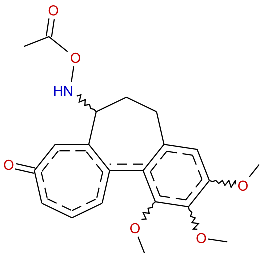	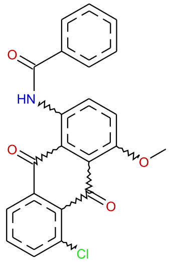	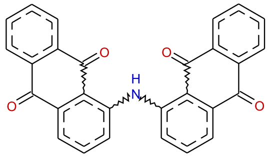
Actual Endpoint	Moderate_Severe	Mild	Mild
Predicted Endpoint	Moderate_Severe	Mild	Mild
Distance	0.674	0.754	0.780
Reference	AJOPAA 31;837;48	28ZPAK-;90;72	28ZPAK-;125;72

Model Applicability

Unknown features are fingerprint features in the query molecule, but not found or appearing too infrequently in the training set.

1. All properties and OPS components are within expected ranges.

Feature Contribution

Top features for positive contribution

Fingerprint	Bit/Smiles	Feature Structure	Score	Moderate_Severe in training set
FCFP_10	-1034142694	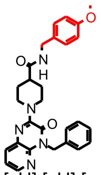 [*]C[c]1:[cH]:[cH]:[c](OC):[cH]:[cH]:1	0.338	18 out of 19

FCFP_10	907096426	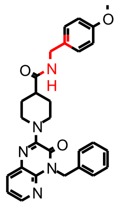 [*]NC[c](:[*]):[*]	0.332	5 out of 5
FCFP_10	427906732	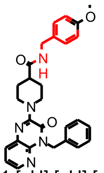 [*]NC[c]1:[cH]:[cH]:[*]:[c]([*]):[cH]:1	0.294	3 out of 3

Top Features for negative contribution

Fingerprint	Bit/Smiles	Feature Structure	Score	Moderate_Severe in training set
FCFP_10	-1977641857	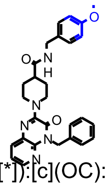 [*][c](:[*]):[c](OC): [cH]:[*]	-0.78	4 out of 15
FCFP_10	907036844	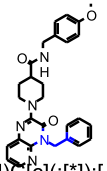 [*]N([*])C[c](:[*]):[*]	-0.6	1 out of 4
FCFP_10	1390842262	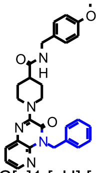 [*]N([*])C[c]1:[cH]:[cH]:[cH]:[cH]:[cH]:1	-0.4	1 out of 3

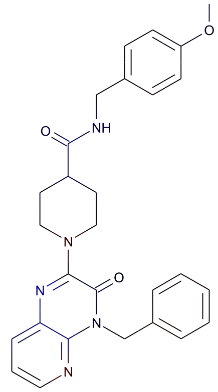

$C_{28}H_{29}N_5O_3$
Molecular Weight: 483.56156
ALogP: 2.942
Rotatable Bonds: 7
Acceptors: 6
Donors: 1

Model Prediction

Prediction: Moderate
Probability: 0.586
Enrichment: 0.945
Bayesian Score: -2.54
Mahalanobis Distance: 12.8
Mahalanobis Distance p-value: 2.12e-007

Prediction: Positive if the Bayesian score is above the estimated best cutoff value from minimizing the false positive and false negative rate.
Probability: The esimated probability that the sample is in the positive category. This assumes that the Bayesian score follows a normal distribution and is different from the prediction using a cutoff.
Enrichment: An estimate of enrichment, that is, the increased likelihood (versus random) of this sample being in the category.
Bayesian Score: The standard Laplacian-modified Bayesian score.
Mahalanobis Distance: The Mahalanobis distance (MD) is the distance to the center of the training data. The larger the MD, the less trustworthy the prediction.
Mahalanobis Distance p-value: The p-value gives the fraction of training data with an MD greater than or equal to the one for the given sample, assuming normally distributed data. The smaller the p-value, the less trustworthy the prediciton. For highly non-normal X properties (e.g., fingerprints), the MD p-value is wildly inaccurate.

Structural Similar Compounds

Name	COLCHICINE	Cinchoninamide; 2-butoxy-N-(2-(diethylamino)ethyl)-; monohydrochloride	1;8;9-ANTHRACENETRIOL; TRIACETATE
Structure	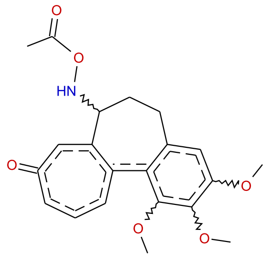	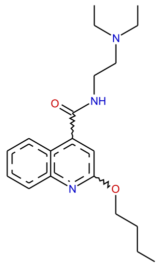	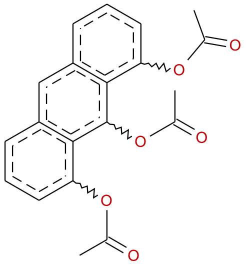
Actual Endpoint	Severe	Severe	Severe
Predicted Endpoint	Severe	Severe	Severe
Distance	0.675	0.793	0.803
Reference	AJOPAA 31;837;48	Arzneimittel-Forschung 8;181;58	BJOPAL 53;819;69

Model Applicability

Unknown features are fingerprint features in the query molecule, but not found or appearing too infrequently in the training set.

1. All properties and OPS components are within expected ranges.

Feature Contribution

Top features for positive contribution				
Fingerprint	Bit/Smiles	Feature Structure	Score	Severe in training set
SCFP_12	-937094999	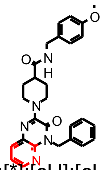 [*][c]1:[*]:[cH]:[cH]:[cH]:n:1	0.42	7 out of 7

SCFP_12	-496201075	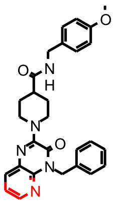 [*]:[cH]:[cH]:n:[*]	0.378	12 out of 13
SCFP_12	-758850909	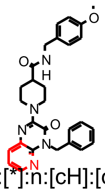 [*][c]1:[*]:n:[cH]:[cH]:[cH]:1	0.348	3 out of 3

Top Features for negative contribution

Fingerprint	Bit/Smiles	Feature Structure	Score	Severe in training set
SCFP_12	-1377141613	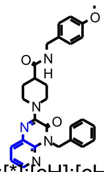 [*][c]1:[*]:[cH]:[cH]:[cH]:[c]:1N=[*]	-1.04	0 out of 3
SCFP_12	1851000357	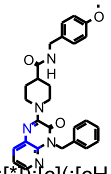 [*][c](:[*]):[c](:[cH]:[*])N=[*]	-1.04	0 out of 3
SCFP_12	1256995004	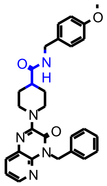 [*]NC(=O)C([*])[*]	-0.483	12 out of 33

Molecule

TOPKAT_Ocular_Irritancy_None_vs_Irritant

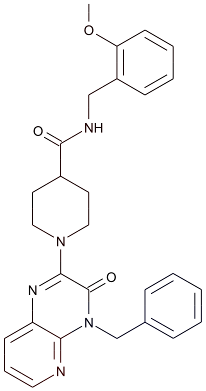

$C_{28}H_{29}N_5O_3$

Molecular Weight: 483.56156

ALogP: 2.942

Rotatable Bonds: 7

Acceptors: 6

Donors: 1

Model Prediction

Prediction: Irritant

Probability: 1

Enrichment: 1.18

Bayesian Score: 2.07

Mahalanobis Distance: 11.2

Mahalanobis Distance p-value: 0.00231

Prediction: Positive if the Bayesian score is above the estimated best cutoff value from minimizing the false positive and false negative rate.
Probability: The esimated probability that the sample is in the positive category. This assumes that the Bayesian score follows a normal distribution and is different from the prediction using a cutoff.
Enrichment: An estimate of enrichment, that is, the increased likelihood (versus random) of this sample being in the category.
Bayesian Score: The standard Laplacian-modified Bayesian score.
Mahalanobis Distance: The Mahalanobis distance (MD) is the distance to the center of the training data. The larger the MD, the less trustworthy the prediction.
Mahalanobis Distance p-value: The p-value gives the fraction of training data with an MD greater than or equal to the one for the given sample, assuming normally distributed data. The smaller the p-value, the less trustworthy the prediciton. For highly non-normal X properties (e.g., fingerprints), the MD p-value is wildly inaccurate.

Structural Similar Compounds

Name	COLCHICINE	1-BENZOYLAMINO-4-METHOXY-5-CHLORANTHRAQUINONE	ANTHRAQUINONE; 1;1'-IMINODI-
Structure	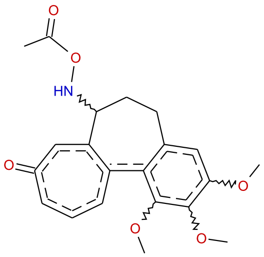	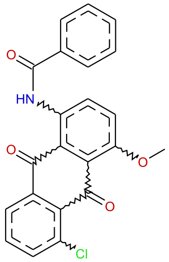	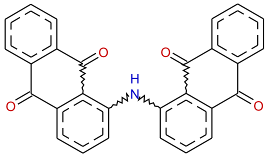
Actual Endpoint	Irritant	Irritant	Irritant
Predicted Endpoint	Irritant	Irritant	Irritant
Distance	0.665	0.734	0.750
Reference	AJOPAA 31;837;48	28ZPAK-;90;72	28ZPAK-;125;72

Model Applicability

Unknown features are fingerprint features in the query molecule, but not found or appearing too infrequently in the training set.

1. All properties and OPS components are within expected ranges.

Feature Contribution

Top features for positive contribution				
**Fingerprint**	**Bit/Smiles**	**Feature Structure**	**Score**	**Irritant in training set**
FCFP_12	1747237384	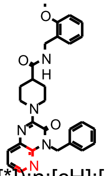 [*][c](:[*]):n:[cH]:[*]	0.208	44 out of 44

FCFP_12	-124655670	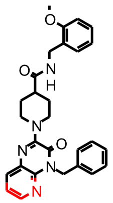 [*]:[cH]:[cH]:n:[*]	0.2	16 out of 16
FCFP_12	-1695756380	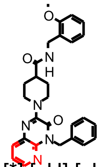 [*][c]1:[*]:[cH]:[cH]:[cH]:n:1	0.194	11 out of 11
**Top Features for negative contribution**				
**Fingerprint**	**Bit/Smiles**	**Feature Structure**	**Score**	**Irritant in training set**
FCFP_12	2106393770	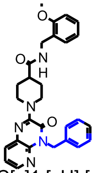 [*]N([*])C[c]1:[cH]:[cH]:[*]:[cH]:[cH]:1	-0.231	3 out of 5
FCFP_12	1390842262	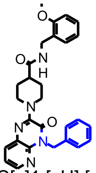 [*]N([*])C[c]1:[cH]:[cH]:[cH]:[cH]:[cH]:1	-0.231	3 out of 5
FCFP_12	907036844	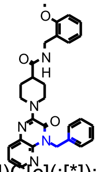 [*]N([*])C[c](:[*]):[*]	-0.156	4 out of 6

#UNDEFINED

TOPKAT_Ocular_Irritancy_None_vs_Irritant

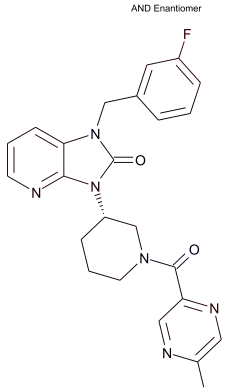

$C_{24}H_{23}FN_6O_2$

Molecular Weight: 446.47682

ALogP: 2.244

Rotatable Bonds: 4

Acceptors: 5

Donors: 0

Structural Similar Compounds

Name	1;8;9-ANTHRACENETRIOL; TRIACETATE	BIS(3;4-EPOXY CYCLOHEXYL METHYL)ADIPATE	PROPANE;2;2-BIS(P-2;3-EPOXYPROPOXY)PHENYL)-
Structure	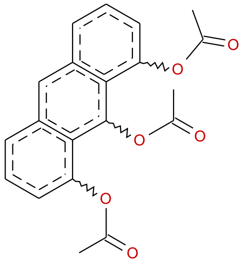	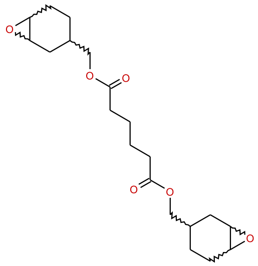	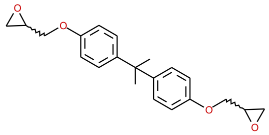
Actual Endpoint	Irritant	Non-Irritant	Irritant
Predicted Endpoint	Irritant	Non-Irritant	Irritant
Distance	0.676	0.723	0.735
Reference	BJOPAL 53;819;69	AIHAAP 30;470;69	28ZPAK-;137;72

Model Prediction

Prediction: Irritant

Probability: 1

Enrichment: 1.18

Bayesian Score: 2.38

Mahalanobis Distance: 12.4

Mahalanobis Distance p-value: 8.67e-006

Prediction: Positive if the Bayesian score is above the estimated best cutoff value from minimizing the false positive and false negative rate.
Probability: The esimated probability that the sample is in the positive category. This assumes that the Bayesian score follows a normal distribution and is different from the prediction using a cutoff.
Enrichment: An estimate of enrichment, that is, the increased likelihood (versus random) of this sample being in the category.
Bayesian Score: The standard Laplacian-modified Bayesian score.
Mahalanobis Distance: The Mahalanobis distance (MD) is the distance to the center of the training data. The larger the MD, the less trustworthy the prediction.
Mahalanobis Distance p-value: The p-value gives the fraction of training data with an MD greater than or equal to the one for the given sample, assuming normally distributed data. The smaller the p-value, the less trustworthy the prediciton. For highly non-normal X properties (e.g., fingerprints), the MD p-value is wildly inaccurate.

Model Applicability

Unknown features are fingerprint features in the query molecule, but not found or appearing too infrequently in the training set.

1. All properties and OPS components are within expected ranges.

Feature Contribution

Top features for positive contribution

Fingerprint	Bit/Smiles	Feature Structure	Score	Irritant in training set
FCFP_12	1747237384	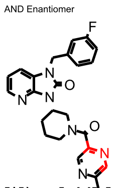 [*][c](:[*]):n:[cH]:[*]	0.208	44 out of 44

FCFP_12	-124655670	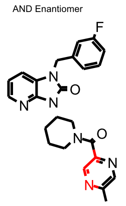 [*]:[cH]:[cH]:n:[*]	0.2	16 out of 16
FCFP_12	-1539132615	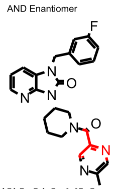 [*]C(=[*])[c](:[cH]:[*]):n:[*]	0.197	13 out of 13

Top Features for negative contribution

Fingerprint	Bit/Smiles	Feature Structure	Score	Irritant in training set
FCFP_12	-1549163031	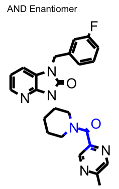 [*]N([*])C(=O)[c](:[*]):[*]	-0.623	16 out of 38
FCFP_12	2106393770	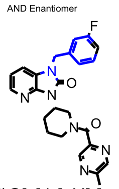 [*]N([*])C[c]1:[cH]:[cH]:[*]:[cH]:[cH]:1	-0.231	3 out of 5
FCFP_12	1390842262	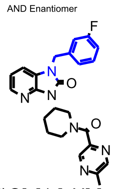 [*]N([*])C[c]1:[cH]:[cH]:[cH]:[cH]:[cH]:1	-0.231	3 out of 5

#UNDEFINED

TOPKAT_Ocular_Irritancy_None_vs_Irritant

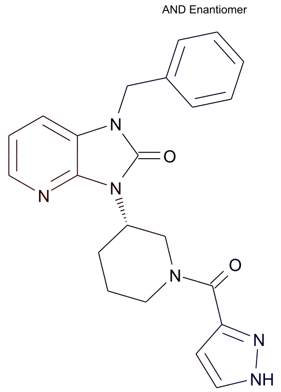

$C_{22}H_{22}N_6O_2$
Molecular Weight: 402.44907
ALogP: 2.501
Rotatable Bonds: 4
Acceptors: 4
Donors: 1

Structural Similar Compounds

Name	1-BENZOYLAMINO-4-METHOXY-5-CHLORANTHRAQUINONE	Anthraquinone; 1-amino-2;4-dibromo-	BENZAMIDE; N-(5-CHLORO-1-ANTHRAQUINONYL)-
Structure	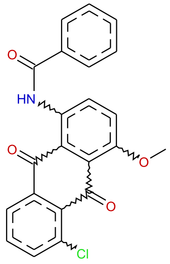	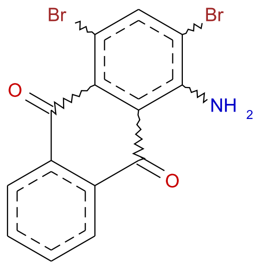	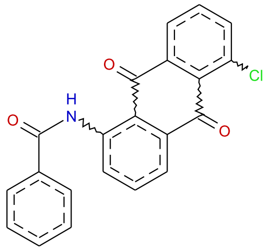
Actual Endpoint	Irritant	Irritant	Irritant
Predicted Endpoint	Irritant	Irritant	Irritant
Distance	0.624	0.638	0.670
Reference	28ZPAK-;90;72	Prehled Prumyslove Toxikologie; Organicke Latky; Marhold; J. pp 565;86	28ZPAK 89;72

Model Prediction

Prediction: Non-Irritant
Probability: 0.974
Enrichment: 1.15
Bayesian Score: -0.473
Mahalanobis Distance: 12
Mahalanobis Distance p-value: 4.75e-005

Prediction: Positive if the Bayesian score is above the estimated best cutoff value from minimizing the false positive and false negative rate.
Probability: The esimated probability that the sample is in the positive category. This assumes that the Bayesian score follows a normal distribution and is different from the prediction using a cutoff.
Enrichment: An estimate of enrichment, that is, the increased likelihood (versus random) of this sample being in the category.
Bayesian Score: The standard Laplacian-modified Bayesian score.
Mahalanobis Distance: The Mahalanobis distance (MD) is the distance to the center of the training data. The larger the MD, the less trustworthy the prediction.
Mahalanobis Distance p-value: The p-value gives the fraction of training data with an MD greater than or equal to the one for the given sample, assuming normally distributed data. The smaller the p-value, the less trustworthy the prediciton. For highly non-normal X properties (e.g., fingerprints), the MD p-value is wildly inaccurate.

Model Applicability

Unknown features are fingerprint features in the query molecule, but not found or appearing too infrequently in the training set.

1. All properties and OPS components are within expected ranges.

Feature Contribution

Top features for positive contribution

Fingerprint	Bit/Smiles	Feature Structure	Score	Irritant in training set
FCFP_12	1747237384	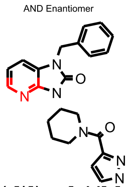 [*][c](:[*]):n:[cH]:[H *]	0.208	44 out of 44

FCFP_12	-124655670	AND Enantiomer 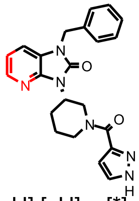 [*]:[cH]:[cH]:n:[*]	0.2	16 out of 16
FCFP_12	-1539132615	AND Enantiomer 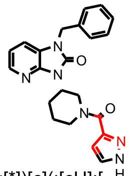 [*]C(=[*])[c](:[cH]:[*]):n:[*]	0.197	13 out of 13

Top Features for negative contribution

Fingerprint	Bit/Smiles	Feature Structure	Score	Irritant in training set
FCFP_12	-1549163031	AND Enantiomer 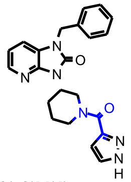 [*]N([*])C(=O)[c](:[*]):[*]	-0.623	16 out of 38
FCFP_12	1747267175	AND Enantiomer 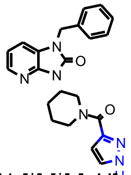 [*][c]1:[*]:[*]:[nH]: n:1	-0.592	0 out of 1
FCFP_12	262592487	AND Enantiomer 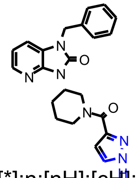 [*]1:[*]:n:[nH]:[cH]: 1	-0.592	0 out of 1

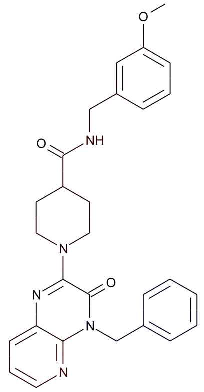

$C_{28}H_{29}N_5O_3$
Molecular Weight: 483.56156
ALogP: 2.942
Rotatable Bonds: 7
Acceptors: 6
Donors: 1

Model Prediction

Prediction: Irritant
Probability: 1
Enrichment: 1.18
Bayesian Score: 1.97
Mahalanobis Distance: 11.2
Mahalanobis Distance p-value: 0.00231

Prediction: Positive if the Bayesian score is above the estimated best cutoff value from minimizing the false positive and false negative rate.
Probability: The esimated probability that the sample is in the positive category. This assumes that the Bayesian score follows a normal distribution and is different from the prediction using a cutoff.
Enrichment: An estimate of enrichment, that is, the increased likelihood (versus random) of this sample being in the category.
Bayesian Score: The standard Laplacian-modified Bayesian score.
Mahalanobis Distance: The Mahalanobis distance (MD) is the distance to the center of the training data. The larger the MD, the less trustworthy the prediction.
Mahalanobis Distance p-value: The p-value gives the fraction of training data with an MD greater than or equal to the one for the given sample, assuming normally distributed data. The smaller the p-value, the less trustworthy the prediciton. For highly non-normal X properties (e.g., fingerprints), the MD p-value is wildly inaccurate.

Structural Similar Compounds

Name	COLCHICINE	1-BENZOYLAMINO-4-METHOXY-5-CHLORANTHRAQUINONE	ANTHRAQUINONE; 1;1'-IMINODI-
Structure	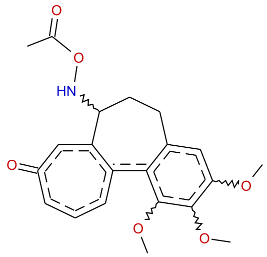	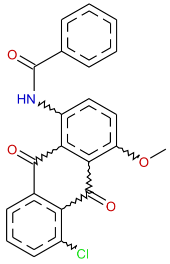	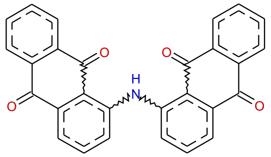
Actual Endpoint	Irritant	Irritant	Irritant
Predicted Endpoint	Irritant	Irritant	Irritant
Distance	0.663	0.738	0.751
Reference	AJOPAA 31;837;48	28ZPAK-;90;72	28ZPAK-;125;72

Model Applicability

Unknown features are fingerprint features in the query molecule, but not found or appearing too infrequently in the training set.

1. All properties and OPS components are within expected ranges.

Feature Contribution

Top features for positive contribution

Fingerprint	Bit/Smiles	Feature Structure	Score	Irritant in training set
FCFP_12	1747237384	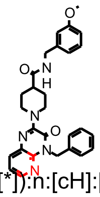 [*][c](:[*]):n:[cH]:[*]	0.208	44 out of 44

FCFP_12	-124655670	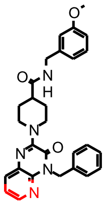 [*]:[cH]:[cH]:n:[*]	0.2	16 out of 16
FCFP_12	-1695756380	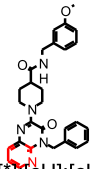 [*][c]1:[*]:[cH]:[cH]:[cH]:n:1	0.194	11 out of 11

Top Features for negative contribution

**Fingerprint**	**Bit/Smiles**	**Feature Structure**	**Score**	**Irritant in training set**
FCFP_12	-1757681964	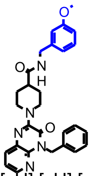 [*]C[c]1:[cH]:[cH]:[cH]:[c](OC):[cH]:1	-0.268	1 out of 2
FCFP_12	1390842262	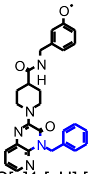 [*]N([*])C[c]1:[cH]:[cH]:[cH]:[cH]:[cH]:1	-0.231	3 out of 5
FCFP_12	2106393770	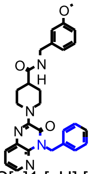 [*]N([*])C[c]1:[cH]:[cH]:[*]:[cH]:[cH]:1	-0.231	3 out of 5

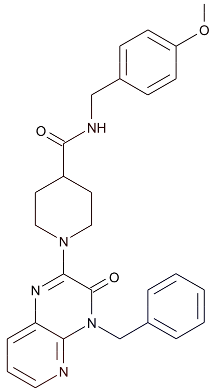

$C_{28}H_{29}N_5O_3$
Molecular Weight: 483.56156
ALogP: 2.942
Rotatable Bonds: 7
Acceptors: 6
Donors: 1

Model Prediction

Prediction: Irritant

Probability: 1

Enrichment: 1.18

Bayesian Score: 1.94

Mahalanobis Distance: 11.2

Mahalanobis Distance p-value: 0.00231

Prediction: Positive if the Bayesian score is above the estimated best cutoff value from minimizing the false positive and false negative rate.
Probability: The esimated probability that the sample is in the positive category. This assumes that the Bayesian score follows a normal distribution and is different from the prediction using a cutoff.
Enrichment: An estimate of enrichment, that is, the increased likelihood (versus random) of this sample being in the category.
Bayesian Score: The standard Laplacian-modified Bayesian score.
Mahalanobis Distance: The Mahalanobis distance (MD) is the distance to the center of the training data. The larger the MD, the less trustworthy the prediction.
Mahalanobis Distance p-value: The p-value gives the fraction of training data with an MD greater than or equal to the one for the given sample, assuming normally distributed data. The smaller the p-value, the less trustworthy the prediciton. For highly non-normal X properties (e.g., fingerprints), the MD p-value is wildly inaccurate.

Structural Similar Compounds

Name	COLCHICINE	1-BENZOYLAMINO-4-METHOXY-5-CHLORANTHRAQUINONE	ANTHRAQUINONE; 1;1'-IMINODI-
Structure	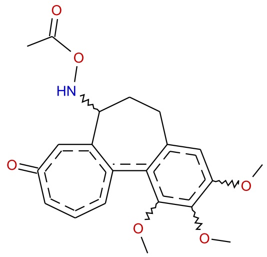	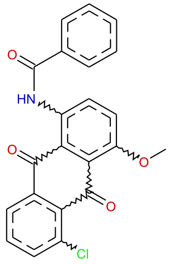	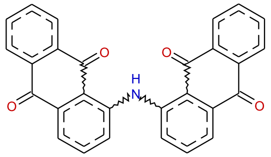
Actual Endpoint	Irritant	Irritant	Irritant
Predicted Endpoint	Irritant	Irritant	Irritant
Distance	0.666	0.738	0.751
Reference	AJOPAA 31;837;48	28ZPAK-;90;72	28ZPAK-;125;72

Model Applicability

Unknown features are fingerprint features in the query molecule, but not found or appearing too infrequently in the training set.

1. All properties and OPS components are within expected ranges.

Feature Contribution

Top features for positive contribution

Fingerprint	Bit/Smiles	Feature Structure	Score	Irritant in training set
FCFP_12	1747237384	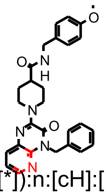 [*][c](:[*]):n:[cH]:[*]	0.208	44 out of 44

FCFP_12	-124655670	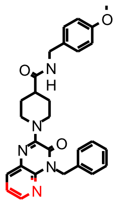 [*]:[cH]:[cH]:n:[*]	0.2	16 out of 16
FCFP_12	-1695756380	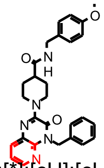 [*][c]1:[*]:[cH]:[cH]<br>:[cH]:n:1	0.194	11 out of 11

Top Features for negative contribution

Fingerprint	Bit/Smiles	Feature Structure	Score	Irritant in training set
FCFP_12	1390842262	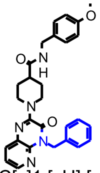 [*]N([*])C[c]1:[cH]:[<br>cH]:[cH]:[cH]:[cH]:1	-0.231	3 out of 5
FCFP_12	2106393770	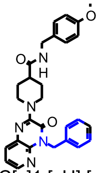 [*]N([*])C[c]1:[cH]:[<br>cH]:[*]:[cH]:[cH]:1	-0.231	3 out of 5
FCFP_12	907036844	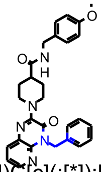 [*]N([*])C[c](:[*]):[<br>*]	-0.156	4 out of 6

Molecule

TOPKAT_Rat_Female_FDA_None_vs_Carcinogen

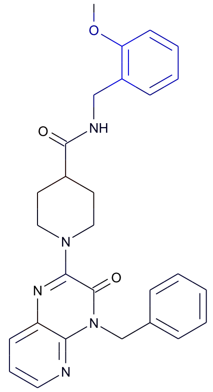

$C_{28}H_{29}N_5O_3$
Molecular Weight: 483.56156
ALogP: 2.942
Rotatable Bonds: 7
Acceptors: 6
Donors: 1

Structural Similar Compounds

Name	Moricizine	Diltiazem	Cisapride
Structure	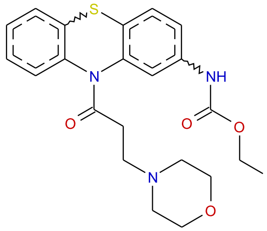	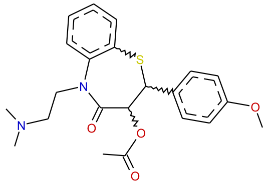	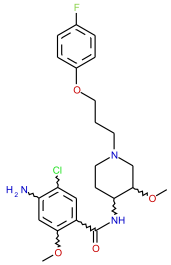
Actual Endpoint	Carcinogen	Carcinogen	Non-Carcinogen
Predicted Endpoint	Carcinogen	Carcinogen	Non-Carcinogen
Distance	0.600	0.647	0.650
Reference	US FDA (Centre for Drug Eval.& Res./Off. Testing & Res.) Sept. 1997	US FDA (Centre for Drug Eval.& Res./Off. Testing & Res.) Sept. 1997	US FDA (Centre for Drug Eval.& Res./Off. Testing & Res.) Sept. 1997

Model Prediction

Prediction: Non-Carcinogen
Probability: 0.188
Enrichment: 0.584
Bayesian Score: -8.86
Mahalanobis Distance: 13.2
Mahalanobis Distance p-value: 6.22e-005

Prediction: Positive if the Bayesian score is above the estimated best cutoff value from minimizing the false positive and false negative rate.
Probability: The esimated probability that the sample is in the positive category. This assumes that the Bayesian score follows a normal distribution and is different from the prediction using a cutoff.
Enrichment: An estimate of enrichment, that is, the increased likelihood (versus random) of this sample being in the category.
Bayesian Score: The standard Laplacian-modified Bayesian score.
Mahalanobis Distance: The Mahalanobis distance (MD) is the distance to the center of the training data. The larger the MD, the less trustworthy the prediction.
Mahalanobis Distance p-value: The p-value gives the fraction of training data with an MD greater than or equal to the one for the given sample, assuming normally distributed data. The smaller the p-value, the less trustworthy the prediciton. For highly non-normal X properties (e.g., fingerprints), the MD p-value is wildly inaccurate.

Model Applicability

Unknown features are fingerprint features in the query molecule, but not found or appearing too infrequently in the training set.

1. OPS PC27 out of range. Value: -3.4494. Training min, max, SD, explained variance: -3.1786, 3.0121, 1.053, 0.0116.

Feature Contribution

Top features for positive contribution

Fingerprint	Bit/Smiles	Feature Structure	Score	Carcinogen in training set
ECFP_12	-509950643	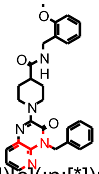 [*]N([*])[c](:n:[*]):[c]([*]):[*]	0.288	2 out of 4

ECFP_12	-1650219925	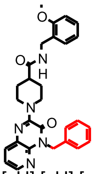 [*]C[c]1:[cH]:[cH]:[cH]:[cH]:[cH]:1	0.208	6 out of 15
ECFP_12	-1106753576	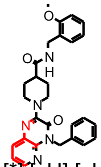 [*][c]1:[*]:[cH]:[cH]:[cH]:[c]:1N=[*]	0.208	1 out of 2

Top Features for negative contribution

Fingerprint	Bit/Smiles	Feature Structure	Score	Carcinogen in training set
ECFP_12	1634699529	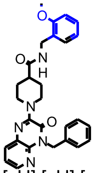 [*]O[c]1:[cH]:[cH]:[cH]:[*]:[c]:1[*]	-1.25	0 out of 8
ECFP_12	497523368	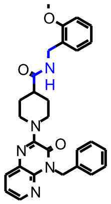 [*]CNC(=[*])[*]	-0.989	1 out of 14
ECFP_12	1584051730	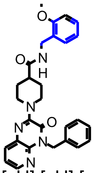 [*]C[c]1:[cH]:[cH]:[cH]:[*]:[c]:1[*]	-0.811	0 out of 4

#UNDEFINED

TOPKAT_Rat_Female_FDA_None_vs_Carcinogen

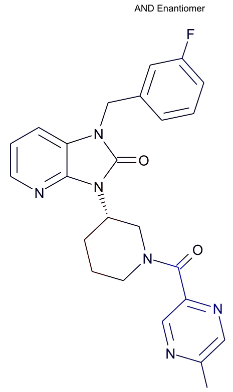

$C_{24}H_{23}FN_6O_2$
Molecular Weight: 446.47682
ALogP: 2.244
Rotatable Bonds: 4
Acceptors: 5
Donors: 0

Structural Similar Compounds

Name	Risperidone	Levocabstine	Buspirone
Structure	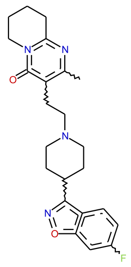	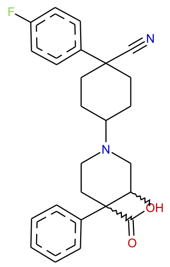	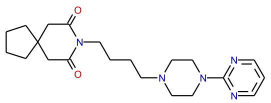
Actual Endpoint	Carcinogen	Non-Carcinogen	Non-Carcinogen
Predicted Endpoint	Carcinogen	Non-Carcinogen	Non-Carcinogen
Distance	0.610	0.636	0.650
Reference	US FDA (Centre for Drug Eval.& Res./Off. Testing & Res.) Sept. 1997	US FDA (Centre for Drug Eval.& Res./Off. Testing & Res.) Sept. 1997	US FDA (Centre for Drug Eval.& Res./Off. Testing & Res.) Sept. 1997

Model Prediction

Prediction: Non-Carcinogen
Probability: 0.217
Enrichment: 0.672
Bayesian Score: -5.43
Mahalanobis Distance: 12.9
Mahalanobis Distance p-value: 0.000197

Prediction: Positive if the Bayesian score is above the estimated best cutoff value from minimizing the false positive and false negative rate.
Probability: The esimated probability that the sample is in the positive category. This assumes that the Bayesian score follows a normal distribution and is different from the prediction using a cutoff.
Enrichment: An estimate of enrichment, that is, the increased likelihood (versus random) of this sample being in the category.
Bayesian Score: The standard Laplacian-modified Bayesian score.
Mahalanobis Distance: The Mahalanobis distance (MD) is the distance to the center of the training data. The larger the MD, the less trustworthy the prediction.
Mahalanobis Distance p-value: The p-value gives the fraction of training data with an MD greater than or equal to the one for the given sample, assuming normally distributed data. The smaller the p-value, the less trustworthy the prediciton. For highly non-normal X properties (e.g., fingerprints), the MD p-value is wildly inaccurate.

Model Applicability

Unknown features are fingerprint features in the query molecule, but not found or appearing too infrequently in the training set.

1. All properties and OPS components are within expected ranges.

Feature Contribution

Top features for positive contribution

Fingerprint	Bit/Smiles	Feature Structure	Score	Carcinogen in training set
ECFP_12	1221859335	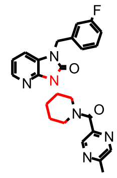 AND Enantiomer [*]N([*])[C@@H]1C[*]CCC1	0.421	1 out of 1

ECFP_12	-709633021	AND Enantiomer 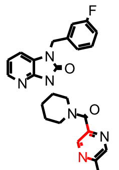 [*][c](:[*]):[cH]:n:[*]	0.341	7 out of 15
ECFP_12	-1950934120	AND Enantiomer 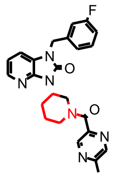 [*][C@@H]1[*]N([*])CCC1	0.33	3 out of 6

Top Features for negative contribution

Fingerprint	Bit/Smiles	Feature Structure	Score	Carcinogen in training set
ECFP_12	-1102925512	AND Enantiomer 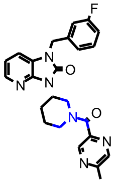 [*]CN(C[*])C(=[*])[*]	-1.06	0 out of 6
ECFP_12	1171844666	AND Enantiomer 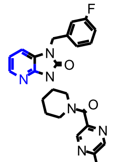 [*][c]1:[*]:[cH]:[cH]:[cH]:n:1	-0.797	1 out of 11
ECFP_12	1413420509	AND Enantiomer 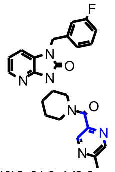 [*]C(=[*])[c](:[cH]:[*]):n:[*]	-0.661	0 out of 3

#UNDEFINED

TOPKAT_Rat_Female_FDA_None_vs_Carcinogen

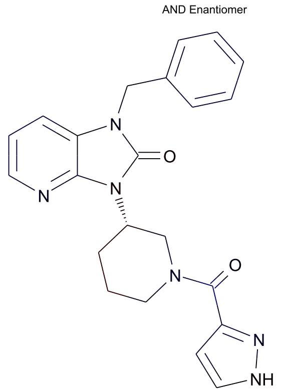

$C_{22}H_{22}N_6O_2$

Molecular Weight: 402.44907

ALogP: 2.501

Rotatable Bonds: 4

Acceptors: 4

Donors: 1

Model Prediction

Prediction: Non-Carcinogen

Probability: 0.241

Enrichment: 0.749

Bayesian Score: -3.4

Mahalanobis Distance: 11.5

Mahalanobis Distance p-value: 0.0203

Prediction: Positive if the Bayesian score is above the estimated best cutoff value from minimizing the false positive and false negative rate.
Probability: The esimated probability that the sample is in the positive category. This assumes that the Bayesian score follows a normal distribution and is different from the prediction using a cutoff.
Enrichment: An estimate of enrichment, that is, the increased likelihood (versus random) of this sample being in the category.
Bayesian Score: The standard Laplacian-modified Bayesian score.
Mahalanobis Distance: The Mahalanobis distance (MD) is the distance to the center of the training data. The larger the MD, the less trustworthy the prediction.
Mahalanobis Distance p-value: The p-value gives the fraction of training data with an MD greater than or equal to the one for the given sample, assuming normally distributed data. The smaller the p-value, the less trustworthy the prediciton. For highly non-normal X properties (e.g., fingerprints), the MD p-value is wildly inaccurate.

Structural Similar Compounds

Name	Levocabstine	Lansoprazole	Omeprazole
Structure	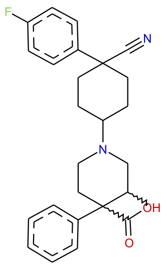	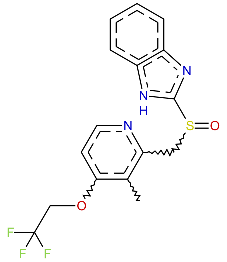	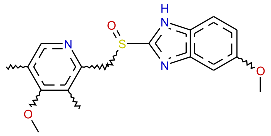
Actual Endpoint	Non-Carcinogen	Carcinogen	Carcinogen
Predicted Endpoint	Non-Carcinogen	Carcinogen	Carcinogen
Distance	0.555	0.638	0.644
Reference	US FDA (Centre for Drug Eval.& Res./Off. Testing & Res.) Sept. 1997	US FDA (Centre for Drug Eval.& Res./Off. Testing & Res.) Sept. 1997	US FDA (Centre for Drug Eval.& Res./Off. Testing & Res.) Sept. 1997

Model Applicability

Unknown features are fingerprint features in the query molecule, but not found or appearing too infrequently in the training set.

1. All properties and OPS components are within expected ranges.
2. Unknown ECFP_2 feature: 600440273: [*][c]1:[*]:[*]:[nH]:n:1
3. Unknown ECFP_2 feature: -954588747: [*]1:[*]:n:[nH]:[cH]:1

Feature Contribution

Top features for positive contribution

Fingerprint	Bit/Smiles	Feature Structure	Score	Carcinogen in training set
ECFP_12	1221859335	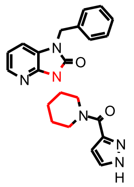 AND Enantiomer [*]N([*])[C@@H]1C[*]C CC1	0.421	1 out of 1

ECFP_12	-152683720	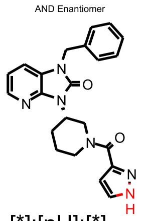<br>[*]:[nH]:[*]	0.412	9 out of 18
ECFP_12	-1950934120	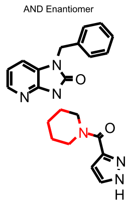<br>[*][C@@H]1[*]N([*])CC<br>C1	0.33	3 out of 6
**Top Features for negative contribution**				
**Fingerprint**	**Bit/Smiles**	**Feature Structure**	**Score**	**Carcinogen in training set**
ECFP_12	-1102925512	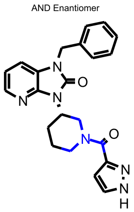<br>[*]CN(C[*])C(=[*])[*]	-1.06	0 out of 6
ECFP_12	1171844666	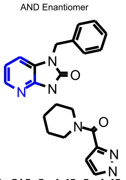<br>[*][c]1:[*]:[cH]:[cH]<br>:[cH]:n:1	-0.797	1 out of 11
ECFP_12	1413420509	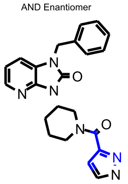<br>[*]C(=[*])[c](:[cH]:[<br>*]):n:[*]	-0.661	0 out of 3

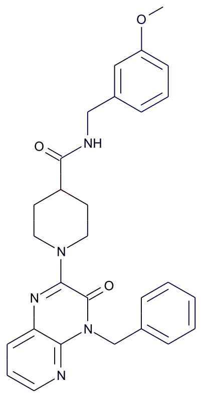

$C_{28}H_{29}N_5O_3$
Molecular Weight: 483.56156
ALogP: 2.942
Rotatable Bonds: 7
Acceptors: 6
Donors: 1

Model Prediction

Prediction: Non-Carcinogen
Probability: 0.213
Enrichment: 0.66
Bayesian Score: -5.8
Mahalanobis Distance: 15.9
Mahalanobis Distance p-value: 1.44e-010

Prediction: Positive if the Bayesian score is above the estimated best cutoff value from minimizing the false positive and false negative rate.
Probability: The esimated probability that the sample is in the positive category. This assumes that the Bayesian score follows a normal distribution and is different from the prediction using a cutoff.
Enrichment: An estimate of enrichment, that is, the increased likelihood (versus random) of this sample being in the category.
Bayesian Score: The standard Laplacian-modified Bayesian score.
Mahalanobis Distance: The Mahalanobis distance (MD) is the distance to the center of the training data. The larger the MD, the less trustworthy the prediction.
Mahalanobis Distance p-value: The p-value gives the fraction of training data with an MD greater than or equal to the one for the given sample, assuming normally distributed data. The smaller the p-value, the less trustworthy the prediciton. For highly non-normal X properties (e.g., fingerprints), the MD p-value is wildly inaccurate.

Structural Similar Compounds

Name	Moricizine	Diltiazem	Ketoconazole
Structure	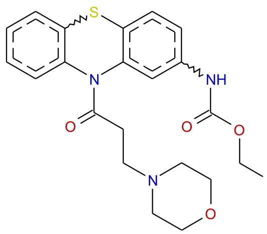	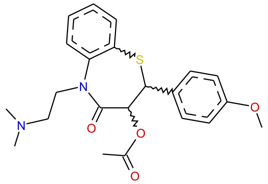	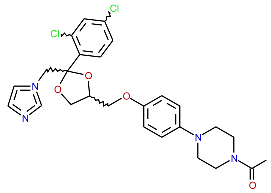
Actual Endpoint	Carcinogen	Carcinogen	Non-Carcinogen
Predicted Endpoint	Carcinogen	Carcinogen	Non-Carcinogen
Distance	0.598	0.643	0.650
Reference	US FDA (Centre for Drug Eval.& Res./Off. Testing & Res.) Sept. 1997	US FDA (Centre for Drug Eval.& Res./Off. Testing & Res.) Sept. 1997	US FDA (Centre for Drug Eval.& Res./Off. Testing & Res.) Sept. 1997

Model Applicability

Unknown features are fingerprint features in the query molecule, but not found or appearing too infrequently in the training set.

1. OPS PC30 out of range. Value: 2.9925. Training min, max, SD, explained variance: -2.9582, 2.682, 0.9684, 0.0098.

Feature Contribution

Top features for positive contribution

Fingerprint	Bit/Smiles	Feature Structure	Score	Carcinogen in training set
ECFP_12	-509950643	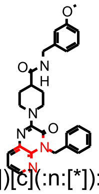 [*]N([*])[c](:n:[*]):[c]([*]):[*]	0.288	2 out of 4

ECFP_12	2055803015	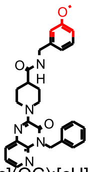<br>[*]:[cH]:[c](OC):[cH]<br>:[*]	0.264	6 out of 14
ECFP_12	-1650219925	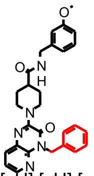<br>[*]C[c]1:[cH]:[cH]:[c<br>H]:[cH]:[cH]:1	0.208	6 out of 15
**Top Features for negative contribution**				
**Fingerprint**	**Bit/Smiles**	**Feature Structure**	**Score**	**Carcinogen in training set**
ECFP_12	497523368	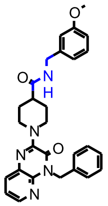<br>[*]CNC(=[*])[*]	-0.989	1 out of 14
ECFP_12	1171844666	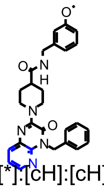<br>[*][c]1:[*]:[cH]:[cH]<br>:[cH]:n:1	-0.797	1 out of 11
ECFP_12	1571214559	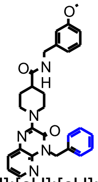<br>[*]1:[cH]:[cH]:[cH]:[<br>cH]:[cH]:1	-0.56	11 out of 64

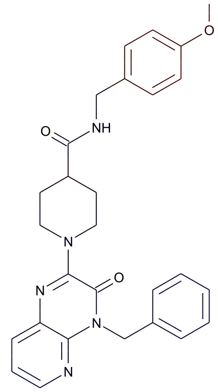

$C_{28}H_{29}N_5O_3$
Molecular Weight: 483.56156
ALogP: 2.942
Rotatable Bonds: 7
Acceptors: 6
Donors: 1

Model Prediction

Prediction: Non-Carcinogen
Probability: 0.245
Enrichment: 0.762
Bayesian Score: -3.11
Mahalanobis Distance: 11.9
Mahalanobis Distance p-value: 0.00652

Prediction: Positive if the Bayesian score is above the estimated best cutoff value from minimizing the false positive and false negative rate.
Probability: The esimated probability that the sample is in the positive category. This assumes that the Bayesian score follows a normal distribution and is different from the prediction using a cutoff.
Enrichment: An estimate of enrichment, that is, the increased likelihood (versus random) of this sample being in the category.
Bayesian Score: The standard Laplacian-modified Bayesian score.
Mahalanobis Distance: The Mahalanobis distance (MD) is the distance to the center of the training data. The larger the MD, the less trustworthy the prediction.
Mahalanobis Distance p-value: The p-value gives the fraction of training data with an MD greater than or equal to the one for the given sample, assuming normally distributed data. The smaller the p-value, the less trustworthy the prediciton. For highly non-normal X properties (e.g., fingerprints), the MD p-value is wildly inaccurate.

Structural Similar Compounds

Name	Moricizine	Diltiazem	Ketoconazole
Structure	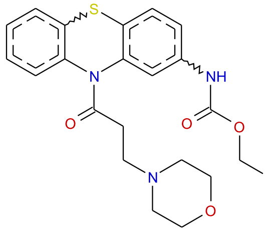	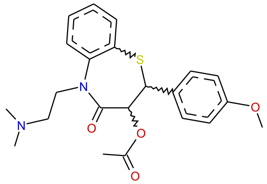	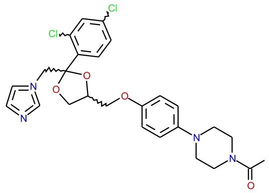
Actual Endpoint	Carcinogen	Carcinogen	Non-Carcinogen
Predicted Endpoint	Carcinogen	Carcinogen	Non-Carcinogen
Distance	0.600	0.635	0.649
Reference	US FDA (Centre for Drug Eval.& Res./Off. Testing & Res.) Sept. 1997	US FDA (Centre for Drug Eval.& Res./Off. Testing & Res.) Sept. 1997	US FDA (Centre for Drug Eval.& Res./Off. Testing & Res.) Sept. 1997

Model Applicability

Unknown features are fingerprint features in the query molecule, but not found or appearing too infrequently in the training set.

1. All properties and OPS components are within expected ranges.

Feature Contribution

Top features for positive contribution				
**Fingerprint**	**Bit/Smiles**	**Feature Structure**	**Score**	**Carcinogen in training set**
ECFP_12	110318898	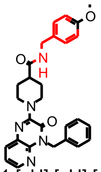 [*]NC[c]1:[cH]:[cH]:[*]:[cH]:[cH]:1	0.421	1 out of 1

ECFP_12	-1271104377	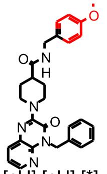 CO[c]1:[cH]:[cH]:[*]:[cH]:[cH]:1	0.33	3 out of 6
ECFP_12	693720869	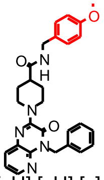 [*][c]1:[cH]:[cH]:[c](OC):[cH]:[cH]:1	0.33	3 out of 6

Top Features for negative contribution

Fingerprint	Bit/Smiles	Feature Structure	Score	Carcinogen in training set
ECFP_12	497523368	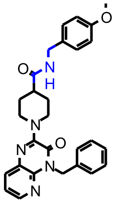 [*]CNC(=[*])[*]	-0.989	1 out of 14
ECFP_12	1171844666	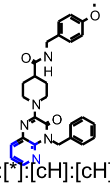 [*][c]1:[*]:[cH]:[cH]:[cH]:n:1	-0.797	1 out of 11
ECFP_12	1571214559	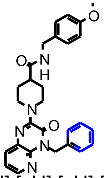 [*]1:[cH]:[cH]:[cH]:[cH]:[cH]:1	-0.56	11 out of 64

Molecule

TOPKAT_Rat_Female_NTP

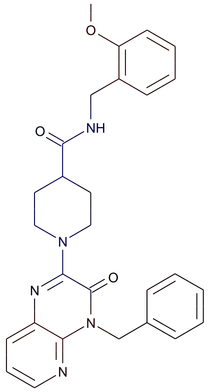

$C_{28}H_{29}N_5O_3$

Molecular Weight: 483.56156

ALogP: 2.942

Rotatable Bonds: 7

Acceptors: 6

Donors: 1

Model Prediction

Prediction: Non-Carcinogen

Probability: 0.471

Enrichment: 1.03

Bayesian Score: -1.78

Mahalanobis Distance: 12.2

Mahalanobis Distance p-value: 2.74e-007

Prediction: Positive if the Bayesian score is above the estimated best cutoff value from minimizing the false positive and false negative rate.
Probability: The esimated probability that the sample is in the positive category. This assumes that the Bayesian score follows a normal distribution and is different from the prediction using a cutoff.
Enrichment: An estimate of enrichment, that is, the increased likelihood (versus random) of this sample being in the category.
Bayesian Score: The standard Laplacian-modified Bayesian score.
Mahalanobis Distance: The Mahalanobis distance (MD) is the distance to the center of the training data. The larger the MD, the less trustworthy the prediction.
Mahalanobis Distance p-value: The p-value gives the fraction of training data with an MD greater than or equal to the one for the given sample, assuming normally distributed data. The smaller the p-value, the less trustworthy the prediciton. For highly non-normal X properties (e.g., fingerprints), the MD p-value is wildly inaccurate.

Structural Similar Compounds

Name	Curcumin	Rhodamine 6G	Rotenone
Structure	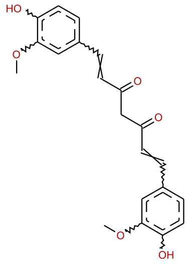	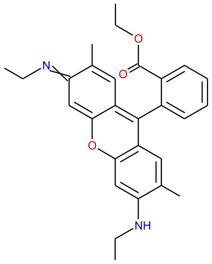	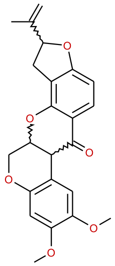
Actual Endpoint	Carcinogen	Carcinogen	Non-Carcinogen
Predicted Endpoint	Carcinogen	Carcinogen	Non-Carcinogen
Distance	0.697	0.722	0.771
Reference	NTP427	NTP364	NTP320

Model Applicability

Unknown features are fingerprint features in the query molecule, but not found or appearing too infrequently in the training set.

1. All properties and OPS components are within expected ranges.
2. Unknown FCFP_2 feature: 580453787: [*]C(=N[c](:[*]):[*])[*]

Feature Contribution

Top features for positive contribution

Fingerprint	Bit/Smiles	Feature Structure	Score	Carcinogen in training set
FCFP_12	-1410049896	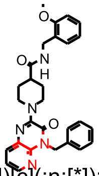 [*]N([*])[c](:n:[*]):[c]([*]):[*]	0.344	1 out of 1

FCFP_12	-2090462286	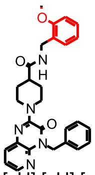 [*]O[c]1:[cH]:[cH]:[cH]:[cH]:[c]:1[*]	0.32	9 out of 15
FCFP_12	1674451008	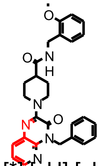 [*][c]1:[*]:[cH]:[cH]:[cH]:[c]:1N=[*]	0.291	26 out of 46

Top Features for negative contribution

Fingerprint	Bit/Smiles	Feature Structure	Score	Carcinogen in training set
FCFP_12	-1553874037	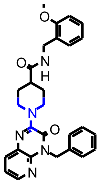 [*]CN(C[*])C(=[*])[*]	-0.774	1 out of 8
FCFP_12	565998553	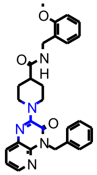 [*]\N=C(\N([*])[*])/C(=[*])[*]	-0.751	4 out of 23
FCFP_12	-1272798659	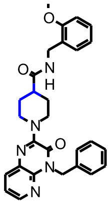 [*]CCC([*])[*]	-0.706	10 out of 51

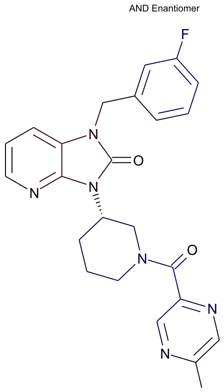

$C_{24}H_{23}FN_6O_2$
Molecular Weight: 446.47682
ALogP: 2.244
Rotatable Bonds: 4
Acceptors: 5
Donors: 0

Model Prediction

Prediction: Non-Carcinogen
Probability: 0.441
Enrichment: 0.968
Bayesian Score: -2.73
Mahalanobis Distance: 12.4
Mahalanobis Distance p-value: 9.5e-008

Prediction: Positive if the Bayesian score is above the estimated best cutoff value from minimizing the false positive and false negative rate.
Probability: The esimated probability that the sample is in the positive category. This assumes that the Bayesian score follows a normal distribution and is different from the prediction using a cutoff.
Enrichment: An estimate of enrichment, that is, the increased likelihood (versus random) of this sample being in the category.
Bayesian Score: The standard Laplacian-modified Bayesian score.
Mahalanobis Distance: The Mahalanobis distance (MD) is the distance to the center of the training data. The larger the MD, the less trustworthy the prediction.
Mahalanobis Distance p-value: The p-value gives the fraction of training data with an MD greater than or equal to the one for the given sample, assuming normally distributed data. The smaller the p-value, the less trustworthy the prediciton. For highly non-normal X properties (e.g., fingerprints), the MD p-value is wildly inaccurate.

Structural Similar Compounds

Name	ROTENONE	Rotenone	Scopolamine hydrobromide trihydrate
Structure	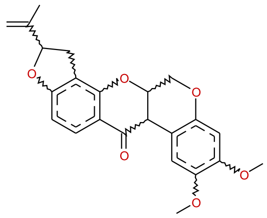	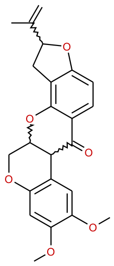	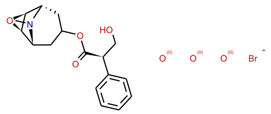
Actual Endpoint	Non-Carcinogen	Non-Carcinogen	Non-Carcinogen
Predicted Endpoint	Non-Carcinogen	Non-Carcinogen	Non-Carcinogen
Distance	0.661	0.661	0.750
Reference	TR-320	NTP320	NTP445

Model Applicability

Unknown features are fingerprint features in the query molecule, but not found or appearing too infrequently in the training set.

1. OPS PC7 out of range. Value: 5.8021. Training min, max, SD, explained variance: -3.0784, 5.5311, 1.55, 0.0420.

Feature Contribution

Top features for positive contribution				
Fingerprint	Bit/Smiles	Feature Structure	Score	Carcinogen in training set
FCFP_12	-1410049896	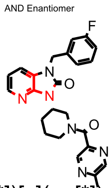 [*]N([*])[c](:n:[*]):[c]([*]):[*]	0.344	1 out of 1

FCFP_12	136120670	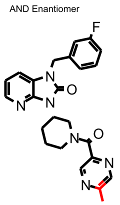 [*]:[c](:[*])C	0.3	15 out of 26
FCFP_12	1674451008	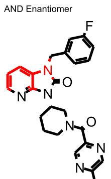 [*][c]1:[*]:[cH]:[cH]:[cH]:[c]:1N=[*]	0.291	26 out of 46

Top Features for negative contribution

Fingerprint	Bit/Smiles	Feature Structure	Score	Carcinogen in training set
FCFP_12	551850122	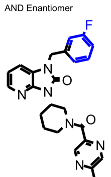 F[c]1:[cH]:[*]:[cH]:[cH]:[cH]:1	-0.824	2 out of 14
FCFP_12	-1553874037	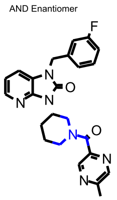 [*]CN(C[*])C(=[*])[*]	-0.774	1 out of 8
FCFP_12	-1272798659	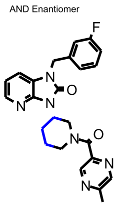 [*]CCC([*])[*]	-0.706	10 out of 51

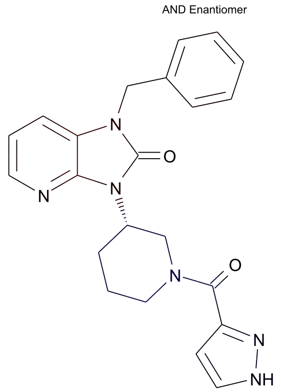

$C_{22}H_{22}N_6O_2$
Molecular Weight: 402.44907
ALogP: 2.501
Rotatable Bonds: 4
Acceptors: 4
Donors: 1

Structural Similar Compounds

Name	Chlorendic Acid	TOLAZAMIDE	Scopolamine hydrobromide trihydrate
Structure	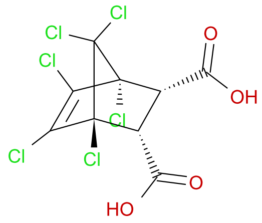	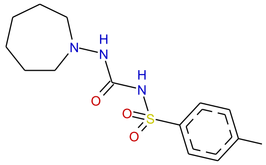	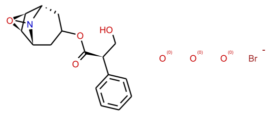
Actual Endpoint	Carcinogen	Non-Carcinogen	Non-Carcinogen
Predicted Endpoint	Carcinogen	Non-Carcinogen	Non-Carcinogen
Distance	0.670	0.677	0.682
Reference	NTP304	TR-51	NTP445

Model Applicability

Unknown features are fingerprint features in the query molecule, but not found or appearing too infrequently in the training set.

1. All properties and OPS components are within expected ranges.
2. Unknown FCFP_2 feature: 1747267175: [*][c]1:[*]:[*]:[nH]:n:1
3. Unknown FCFP_2 feature: 262592487: [*]1:[*]:n:[nH]:[cH]:1
4. Unknown FCFP_2 feature: 1618184456: [*]1:[*]:[cH]:[cH]:[nH]:1

Model Prediction

Prediction: Non-Carcinogen

Probability: 0.498

Enrichment: 1.09

Bayesian Score: -0.841

Mahalanobis Distance: 11.6

Mahalanobis Distance p-value: 4.09e-006

Prediction: Positive if the Bayesian score is above the estimated best cutoff value from minimizing the false positive and false negative rate.
Probability: The esimated probability that the sample is in the positive category. This assumes that the Bayesian score follows a normal distribution and is different from the prediction using a cutoff.
Enrichment: An estimate of enrichment, that is, the increased likelihood (versus random) of this sample being in the category.
Bayesian Score: The standard Laplacian-modified Bayesian score.
Mahalanobis Distance: The Mahalanobis distance (MD) is the distance to the center of the training data. The larger the MD, the less trustworthy the prediction.
Mahalanobis Distance p-value: The p-value gives the fraction of training data with an MD greater than or equal to the one for the given sample, assuming normally distributed data. The smaller the p-value, the less trustworthy the prediciton. For highly non-normal X properties (e.g., fingerprints), the MD p-value is wildly inaccurate.

Feature Contribution

Top features for positive contribution

Fingerprint	Bit/Smiles	Feature Structure	Score	Carcinogen in training set
FCFP_12	-1410049896	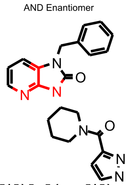 [*]N([*])[c](:n:[*]):[c]([*]):[*]	0.344	1 out of 1

FCFP_12	1674451008	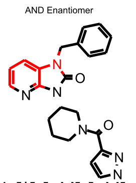 [*][c]1:[*]:[cH]:[cH]:[cH]:[c]:1N=[*]	0.291	26 out of 46
FCFP_12	-1549163031	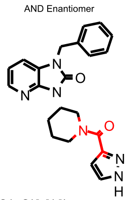 [*]N([*])C(=O)[c](:[*]):[*]	0.225	6 out of 11

Top Features for negative contribution

Fingerprint	Bit/Smiles	Feature Structure	Score	Carcinogen in training set
FCFP_12	-1553874037	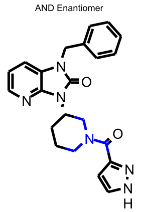 [*]CN(C[*])C(=[*])[*]	-0.774	1 out of 8
FCFP_12	-1272798659	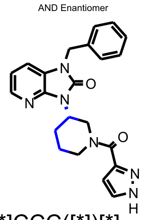 [*]CCC([*])[*]	-0.706	10 out of 51
FCFP_12	19	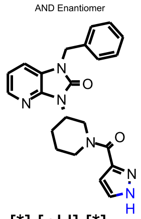 [*]:[nH]:[*]	-0.349	0 out of 1

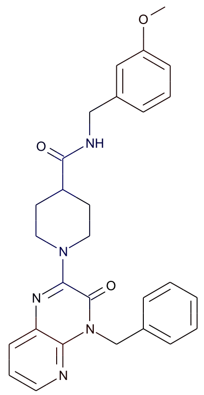

$C_{28}H_{29}N_5O_3$

Molecular Weight: 483.56156

ALogP: 2.942

Rotatable Bonds: 7

Acceptors: 6

Donors: 1

Model Prediction

Prediction: Non-Carcinogen

Probability: 0.464

Enrichment: 1.02

Bayesian Score: -2.01

Mahalanobis Distance: 12.2

Mahalanobis Distance p-value: 2.74e-007

Prediction: Positive if the Bayesian score is above the estimated best cutoff value from minimizing the false positive and false negative rate.
Probability: The esimated probability that the sample is in the positive category. This assumes that the Bayesian score follows a normal distribution and is different from the prediction using a cutoff.
Enrichment: An estimate of enrichment, that is, the increased likelihood (versus random) of this sample being in the category.
Bayesian Score: The standard Laplacian-modified Bayesian score.
Mahalanobis Distance: The Mahalanobis distance (MD) is the distance to the center of the training data. The larger the MD, the less trustworthy the prediction.
Mahalanobis Distance p-value: The p-value gives the fraction of training data with an MD greater than or equal to the one for the given sample, assuming normally distributed data. The smaller the p-value, the less trustworthy the prediciton. For highly non-normal X properties (e.g., fingerprints), the MD p-value is wildly inaccurate.

Structural Similar Compounds

Name	Curcumin	Rhodamine 6G	Rotenone
Structure	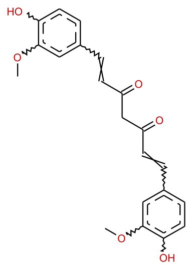	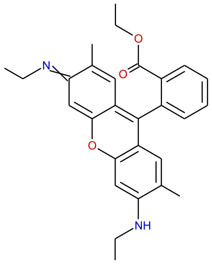	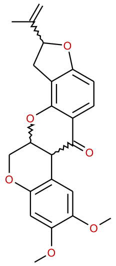
Actual Endpoint	Carcinogen	Carcinogen	Non-Carcinogen
Predicted Endpoint	Carcinogen	Carcinogen	Non-Carcinogen
Distance	0.695	0.726	0.772
Reference	NTP427	NTP364	NTP320

Model Applicability

Unknown features are fingerprint features in the query molecule, but not found or appearing too infrequently in the training set.

1. All properties and OPS components are within expected ranges.
2. Unknown FCFP_2 feature: 580453787: [*]C(=N[c](:[*]):[*])[*]

Feature Contribution

Top features for positive contribution

Fingerprint	Bit/Smiles	Feature Structure	Score	Carcinogen in training set
FCFP_12	-1410049896	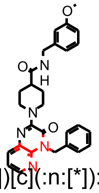 [*]N([*])[c](:n:[*]):[c]([*]):[*]	0.344	1 out of 1

FCFP_12	-1757681964	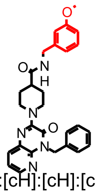<br>[*]C[c]1:[cH]:[cH]:[cH]:[c](OC):[cH]:1	0.344	1 out of 1
FCFP_12	1674451008	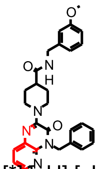<br>[*][c]1:[*]:[cH]:[cH]:[cH]:[c]:1N=[*]	0.291	26 out of 46

Top Features for negative contribution

Fingerprint	Bit/Smiles	Feature Structure	Score	Carcinogen in training set
FCFP_12	-1553874037	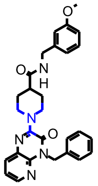<br>[*]CN(C[*])C(=[*])[*]	-0.774	1 out of 8
FCFP_12	565998553	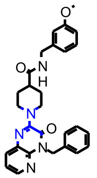<br>[*]\N=C(\N([*])[*])/C(=[*])[*]	-0.751	4 out of 23
FCFP_12	-1272798659	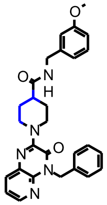<br>[*]CCC([*])[*]	-0.706	10 out of 51

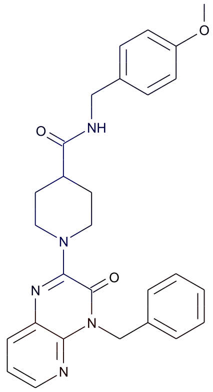

$C_{28}H_{29}N_5O_3$
Molecular Weight: 483.56156
ALogP: 2.942
Rotatable Bonds: 7
Acceptors: 6
Donors: 1

Model Prediction

Prediction: Non-Carcinogen
Probability: 0.431
Enrichment: 0.948
Bayesian Score: -3
Mahalanobis Distance: 12.2
Mahalanobis Distance p-value: 2.74e-007

Prediction: Positive if the Bayesian score is above the estimated best cutoff value from minimizing the false positive and false negative rate.
Probability: The esimated probability that the sample is in the positive category. This assumes that the Bayesian score follows a normal distribution and is different from the prediction using a cutoff.
Enrichment: An estimate of enrichment, that is, the increased likelihood (versus random) of this sample being in the category.
Bayesian Score: The standard Laplacian-modified Bayesian score.
Mahalanobis Distance: The Mahalanobis distance (MD) is the distance to the center of the training data. The larger the MD, the less trustworthy the prediction.
Mahalanobis Distance p-value: The p-value gives the fraction of training data with an MD greater than or equal to the one for the given sample, assuming normally distributed data. The smaller the p-value, the less trustworthy the prediciton. For highly non-normal X properties (e.g., fingerprints), the MD p-value is wildly inaccurate.

Structural Similar Compounds

Name	Curcumin	Rhodamine 6G	Rotenone
Structure	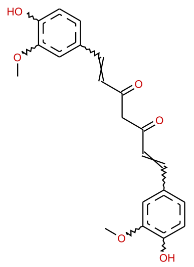	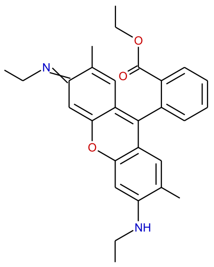	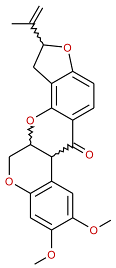
Actual Endpoint	Carcinogen	Carcinogen	Non-Carcinogen
Predicted Endpoint	Carcinogen	Carcinogen	Non-Carcinogen
Distance	0.698	0.726	0.774
Reference	NTP427	NTP364	NTP320

Model Applicability

Unknown features are fingerprint features in the query molecule, but not found or appearing too infrequently in the training set.

1. All properties and OPS components are within expected ranges.
2. Unknown FCFP_2 feature: 580453787: [*]C(=N[c](:[*]):[*])[*]

Feature Contribution

Top features for positive contribution

Fingerprint	Bit/Smiles	Feature Structure	Score	Carcinogen in training set
FCFP_12	-1410049896	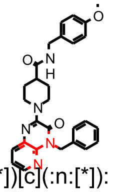 [*]N([*])[c](:n:[*]):[c]([*]):[*]	0.344	1 out of 1

FCFP_12	1674451008	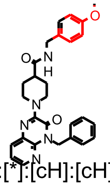 [*][c]1:[*]:[cH]:[cH]:[cH]:[c]:1N=[*]	0.291	26 out of 46
FCFP_12	-1977641857	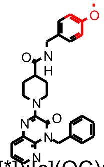 [*][c](:[*]):[c](OC):[cH]:[*]	0.25	11 out of 20

Top Features for negative contribution

Fingerprint	Bit/Smiles	Feature Structure	Score	Carcinogen in training set
FCFP_12	-1553874037	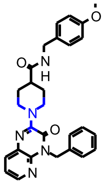 [*]CN(C[*])C(=[*])[*]	-0.774	1 out of 8
FCFP_12	565998553	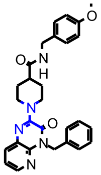 [*]\N=C(\N([*])[*])/C(=[*])[*]	-0.751	4 out of 23
FCFP_12	-1272798659	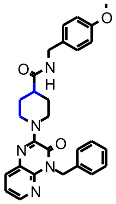 [*]CCC([*])[*]	-0.706	10 out of 51

Molecule

TOPKAT_Rat_Male_FDA_None_vs_Carcinogen

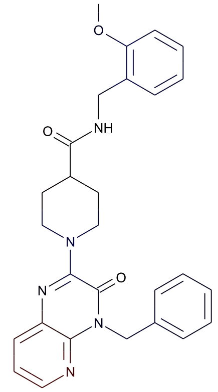

$C_{28}H_{29}N_5O_3$
Molecular Weight: 483.56156
ALogP: 2.942
Rotatable Bonds: 7
Acceptors: 6
Donors: 1

Model Prediction

Prediction: Non-Carcinogen
Probability: 0.265
Enrichment: 0.793
Bayesian Score: -3.66
Mahalanobis Distance: 16.7
Mahalanobis Distance p-value: 1.48e-010

Prediction: Positive if the Bayesian score is above the estimated best cutoff value from minimizing the false positive and false negative rate.
Probability: The esimated probability that the sample is in the positive category. This assumes that the Bayesian score follows a normal distribution and is different from the prediction using a cutoff.
Enrichment: An estimate of enrichment, that is, the increased likelihood (versus random) of this sample being in the category.
Bayesian Score: The standard Laplacian-modified Bayesian score.
Mahalanobis Distance: The Mahalanobis distance (MD) is the distance to the center of the training data. The larger the MD, the less trustworthy the prediction.
Mahalanobis Distance p-value: The p-value gives the fraction of training data with an MD greater than or equal to the one for the given sample, assuming normally distributed data. The smaller the p-value, the less trustworthy the prediciton. For highly non-normal X properties (e.g., fingerprints), the MD p-value is wildly inaccurate.

Structural Similar Compounds

Name	Moricizine	Diltiazem	Ketoconazole
Structure	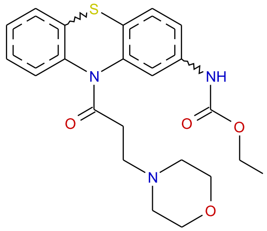	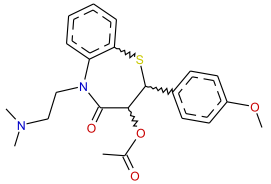	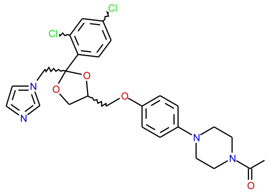
Actual Endpoint	Carcinogen	Non-Carcinogen	Non-Carcinogen
Predicted Endpoint	Carcinogen	Non-Carcinogen	Non-Carcinogen
Distance	0.589	0.609	0.626
Reference	US FDA (Centre for Drug Eval.& Res./Off. Testing & Res.) Sept. 1997	US FDA (Centre for Drug Eval.& Res./Off. Testing & Res.) Sept. 1997	US FDA (Centre for Drug Eval.& Res./Off. Testing & Res.) Sept. 1997

Model Applicability

Unknown features are fingerprint features in the query molecule, but not found or appearing too infrequently in the training set.

1. All properties and OPS components are within expected ranges.

Feature Contribution

Top features for positive contribution				
**Fingerprint**	**Bit/Smiles**	**Feature Structure**	**Score**	**Carcinogen in training set**
SCFP_6	-1377141613	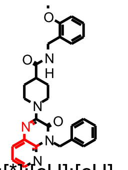 [*][c]1:[*]:[cH]:[cH]:[cH]:[c]:1N=[*]	0.429	3 out of 5

SCFP_6	38222003	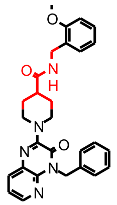<br>[*]CNC(=O)C(C[*])C[*]	0.415	1 out of 1
SCFP_6	-758850909	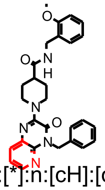<br>[*][c]1:[*]:n:[cH]:[c<br>H]:[cH]:1	0.355	5 out of 10
**Top Features for negative contribution**				
**Fingerprint**	**Bit/Smiles**	**Feature Structure**	**Score**	**Carcinogen in training set**
SCFP_6	-1325723550	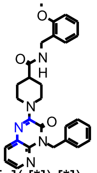<br>[*]C(=N[c](:[*]):[*])<br>[*]	-0.664	1 out of 9
SCFP_6	1653911926	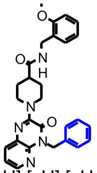<br>[*][c]1:[cH]:[cH]:[cH<br>]:[cH]:[cH]:1	-0.504	12 out of 64
SCFP_6	399719551	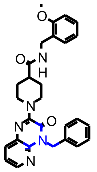<br>[*]CN(C(=[*])[*])[c](<br>:[*]):[*]	-0.48	2 out of 12

#UNDEFINED

TOPKAT_Rat_Male_FDA_None_vs_Carcinogen

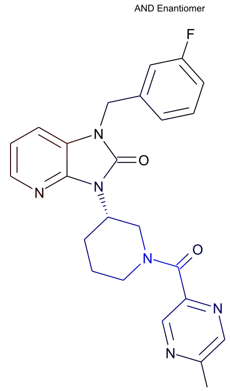

$C_{24}H_{23}FN_6O_2$
Molecular Weight: 446.47682
ALogP: 2.244
Rotatable Bonds: 4
Acceptors: 5
Donors: 0

Structural Similar Compounds

Name	Risperidone	Levocabstine	Buspirone
Structure	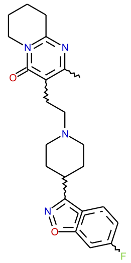	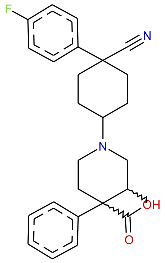	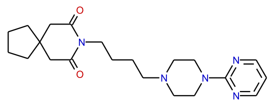
Actual Endpoint	Carcinogen	Non-Carcinogen	Non-Carcinogen
Predicted Endpoint	Carcinogen	Non-Carcinogen	Non-Carcinogen
Distance	0.595	0.619	0.632
Reference	US FDA (Centre for Drug Eval.& Res./Off. Testing & Res.) Sept. 1997	US FDA (Centre for Drug Eval.& Res./Off. Testing & Res.) Sept. 1997	US FDA (Centre for Drug Eval.& Res./Off. Testing & Res.) Sept. 1997

Model Prediction

Prediction: Non-Carcinogen
Probability: 0.236
Enrichment: 0.708
Bayesian Score: -5.11
Mahalanobis Distance: 17.5
Mahalanobis Distance p-value: 1.51e-012

Prediction: Positive if the Bayesian score is above the estimated best cutoff value from minimizing the false positive and false negative rate.
Probability: The esimated probability that the sample is in the positive category. This assumes that the Bayesian score follows a normal distribution and is different from the prediction using a cutoff.
Enrichment: An estimate of enrichment, that is, the increased likelihood (versus random) of this sample being in the category.
Bayesian Score: The standard Laplacian-modified Bayesian score.
Mahalanobis Distance: The Mahalanobis distance (MD) is the distance to the center of the training data. The larger the MD, the less trustworthy the prediction.
Mahalanobis Distance p-value: The p-value gives the fraction of training data with an MD greater than or equal to the one for the given sample, assuming normally distributed data. The smaller the p-value, the less trustworthy the prediciton. For highly non-normal X properties (e.g., fingerprints), the MD p-value is wildly inaccurate.

Model Applicability

Unknown features are fingerprint features in the query molecule, but not found or appearing too infrequently in the training set.

1. All properties and OPS components are within expected ranges.

Feature Contribution

Top features for positive contribution

Fingerprint	Bit/Smiles	Feature Structure	Score	Carcinogen in training set
SCFP_6	-758850909	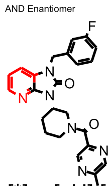 [*][c]1:[*]:n:[cH]:[cH]:[cH]:1	0.355	5 out of 10

SCFP_6	136686699	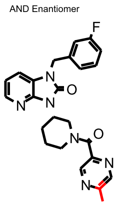 [*]:[c](:[*])C	0.287	17 out of 39
SCFP_6	-1375926917	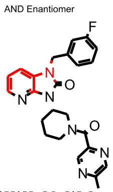 [*]N1[*][*][c]2:[*]:[cH]:[cH]:[cH]:[c]1:2	0.251	11 out of 26

Top Features for negative contribution

Fingerprint	Bit/Smiles	Feature Structure	Score	Carcinogen in training set
SCFP_6	306578635	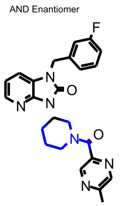 [*]C(=[*])N1C[*]CCC1	-0.825	0 out of 4
SCFP_6	-827073191	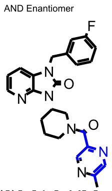 [*]C(=[*])[c]1:[cH]:[*]:[c]([*]):[cH]:n:1	-0.674	0 out of 3
SCFP_6	-666395712	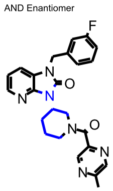 [*]N([*])[C@@H]1C[*]CCC1	-0.664	1 out of 9

#UNDEFINED

TOPKAT_Rat_Male_FDA_None_vs_Carcinogen

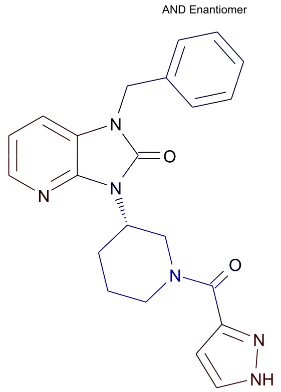

$C_{22}H_{22}N_6O_2$
Molecular Weight: 402.44907
ALogP: 2.501
Rotatable Bonds: 4
Acceptors: 4
Donors: 1

Structural Similar Compounds

Name	Levocabstine	Lansoprazole	Omeprazole
Structure	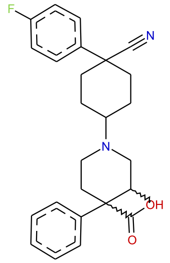	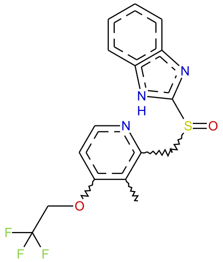	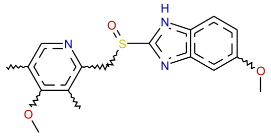
Actual Endpoint	Non-Carcinogen	Carcinogen	Carcinogen
Predicted Endpoint	Non-Carcinogen	Carcinogen	Carcinogen
Distance	0.549	0.624	0.630
Reference	US FDA (Centre for Drug Eval.& Res./Off. Testing & Res.) Sept. 1997	US FDA (Centre for Drug Eval.& Res./Off. Testing & Res.) Sept. 1997	US FDA (Centre for Drug Eval.& Res./Off. Testing & Res.) Sept. 1997

Model Prediction

Prediction: Non-Carcinogen
Probability: 0.279
Enrichment: 0.833
Bayesian Score: -3.04
Mahalanobis Distance: 16.5
Mahalanobis Distance p-value: 3.82e-010

Prediction: Positive if the Bayesian score is above the estimated best cutoff value from minimizing the false positive and false negative rate.
Probability: The esimated probability that the sample is in the positive category. This assumes that the Bayesian score follows a normal distribution and is different from the prediction using a cutoff.
Enrichment: An estimate of enrichment, that is, the increased likelihood (versus random) of this sample being in the category.
Bayesian Score: The standard Laplacian-modified Bayesian score.
Mahalanobis Distance: The Mahalanobis distance (MD) is the distance to the center of the training data. The larger the MD, the less trustworthy the prediction.
Mahalanobis Distance p-value: The p-value gives the fraction of training data with an MD greater than or equal to the one for the given sample, assuming normally distributed data. The smaller the p-value, the less trustworthy the prediciton. For highly non-normal X properties (e.g., fingerprints), the MD p-value is wildly inaccurate.

Model Applicability

Unknown features are fingerprint features in the query molecule, but not found or appearing too infrequently in the training set.

1. All properties and OPS components are within expected ranges.

Feature Contribution

Top features for positive contribution

Fingerprint	Bit/Smiles	Feature Structure	Score	Carcinogen in training set
SCFP_6	149212520	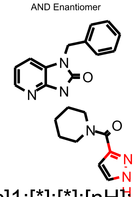 [*][c]1:[*]:[*]:[nH]:n:1	0.543	9 out of 15

SCFP_6	-378091462	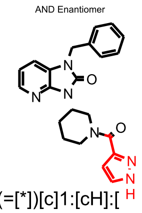 [*]C(=[*])[c]1:[cH]:[cH]:[nH]:n:1	0.415	1 out of 1
SCFP_6	-758850909	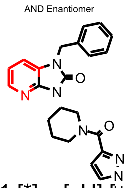 [*][c]1:[*]:n:[cH]:[c H]:[cH]:1	0.355	5 out of 10

Top Features for negative contribution

Fingerprint	Bit/Smiles	Feature Structure	Score	Carcinogen in training set
SCFP_6	306578635	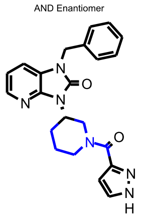 [*]C(=[*])N1C[*]CCC1	-0.825	0 out of 4
SCFP_6	-666395712	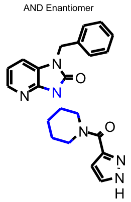 [*]N([*])[C@@H]1C[*]C CC1	-0.664	1 out of 9
SCFP_6	-1343150366	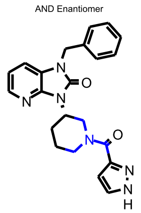 [*]CN(C[*])C(=[*])[*]	-0.659	3 out of 21

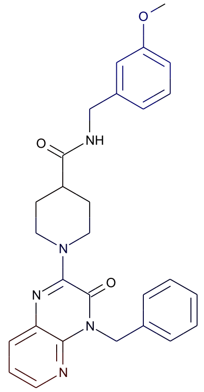

$C_{28}H_{29}N_5O_3$
Molecular Weight: 483.56156
ALogP: 2.942
Rotatable Bonds: 7
Acceptors: 6
Donors: 1

Structural Similar Compounds

Name	Moricizine	Diltiazem	Ketoconazole
Structure	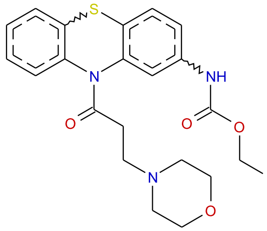	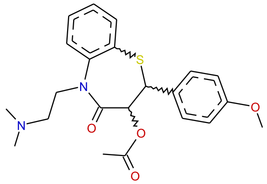	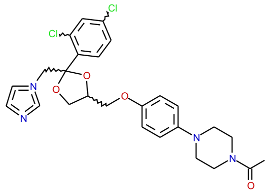
Actual Endpoint	Carcinogen	Non-Carcinogen	Non-Carcinogen
Predicted Endpoint	Carcinogen	Non-Carcinogen	Non-Carcinogen
Distance	0.589	0.604	0.623
Reference	US FDA (Centre for Drug Eval.& Res./Off. Testing & Res.) Sept. 1997	US FDA (Centre for Drug Eval.& Res./Off. Testing & Res.) Sept. 1997	US FDA (Centre for Drug Eval.& Res./Off. Testing & Res.) Sept. 1997

Model Prediction

Prediction: Non-Carcinogen
Probability: 0.237
Enrichment: 0.708
Bayesian Score: -5.1
Mahalanobis Distance: 16.8
Mahalanobis Distance p-value: 1.03e-010

Prediction: Positive if the Bayesian score is above the estimated best cutoff value from minimizing the false positive and false negative rate.
Probability: The esimated probability that the sample is in the positive category. This assumes that the Bayesian score follows a normal distribution and is different from the prediction using a cutoff.
Enrichment: An estimate of enrichment, that is, the increased likelihood (versus random) of this sample being in the category.
Bayesian Score: The standard Laplacian-modified Bayesian score.
Mahalanobis Distance: The Mahalanobis distance (MD) is the distance to the center of the training data. The larger the MD, the less trustworthy the prediction.
Mahalanobis Distance p-value: The p-value gives the fraction of training data with an MD greater than or equal to the one for the given sample, assuming normally distributed data. The smaller the p-value, the less trustworthy the prediciton. For highly non-normal X properties (e.g., fingerprints), the MD p-value is wildly inaccurate.

Model Applicability

Unknown features are fingerprint features in the query molecule, but not found or appearing too infrequently in the training set.

1. All properties and OPS components are within expected ranges.

Feature Contribution

Top features for positive contribution				
**Fingerprint**	**Bit/Smiles**	**Feature Structure**	**Score**	**Carcinogen in training set**
SCFP_6	-1377141613	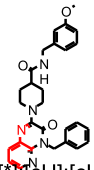 [*][c]1:[*]:[cH]:[cH]:[cH]:[c]:1N=[*]	0.429	3 out of 5

SCFP_6	38222003	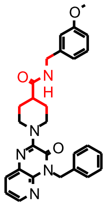 [*]CNC(=O)C(C[*])C[*]	0.415	1 out of 1
SCFP_6	-758850909	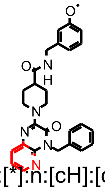 [*][c]1:[*]:n:[cH]:[cH]:[cH]:1	0.355	5 out of 10
**Top Features for negative contribution**				
**Fingerprint**	**Bit/Smiles**	**Feature Structure**	**Score**	**Carcinogen in training set**
SCFP_6	-1632615624	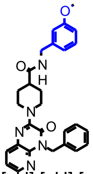 [*]C[c]1:[cH]:[cH]:[cH]:[c](O[*]):[cH]:1	-1.07	0 out of 6
SCFP_6	125999298	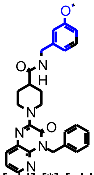 [*]C[c]1:[cH]:[*]:[cH]:[c](O[*]):[cH]:1	-0.7	3 out of 22
SCFP_6	-1325723550	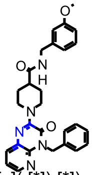 [*]C(=N[c](:[*]):[*])[*]	-0.664	1 out of 9

#UNDEFINED

TOPKAT_Rat_Male_FDA_None_vs_Carcinogen

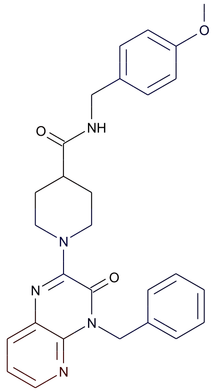

$C_{28}H_{29}N_5O_3$
Molecular Weight: 483.56156
ALogP: 2.942
Rotatable Bonds: 7
Acceptors: 6
Donors: 1

Structural Similar Compounds

Name	Moricizine	Diltiazem	Ketoconazole
Structure	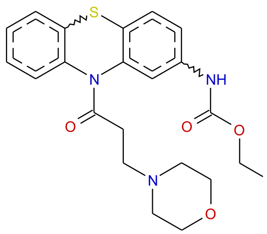	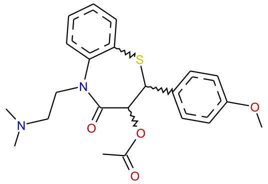	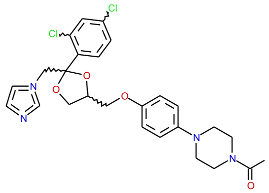
Actual Endpoint	Carcinogen	Non-Carcinogen	Non-Carcinogen
Predicted Endpoint	Carcinogen	Non-Carcinogen	Non-Carcinogen
Distance	0.588	0.593	0.622
Reference	US FDA (Centre for Drug Eval.& Res./Off. Testing & Res.) Sept. 1997	US FDA (Centre for Drug Eval.& Res./Off. Testing & Res.) Sept. 1997	US FDA (Centre for Drug Eval.& Res./Off. Testing & Res.) Sept. 1997

Model Prediction

Prediction: Non-Carcinogen
Probability: 0.26
Enrichment: 0.778
Bayesian Score: -3.9
Mahalanobis Distance: 15.5
Mahalanobis Distance p-value: 6.95e-008

Prediction: Positive if the Bayesian score is above the estimated best cutoff value from minimizing the false positive and false negative rate.
Probability: The esimated probability that the sample is in the positive category. This assumes that the Bayesian score follows a normal distribution and is different from the prediction using a cutoff.
Enrichment: An estimate of enrichment, that is, the increased likelihood (versus random) of this sample being in the category.
Bayesian Score: The standard Laplacian-modified Bayesian score.
Mahalanobis Distance: The Mahalanobis distance (MD) is the distance to the center of the training data. The larger the MD, the less trustworthy the prediction.
Mahalanobis Distance p-value: The p-value gives the fraction of training data with an MD greater than or equal to the one for the given sample, assuming normally distributed data. The smaller the p-value, the less trustworthy the prediciton. For highly non-normal X properties (e.g., fingerprints), the MD p-value is wildly inaccurate.

Model Applicability

Unknown features are fingerprint features in the query molecule, but not found or appearing too infrequently in the training set.

1. All properties and OPS components are within expected ranges.

Feature Contribution

Top features for positive contribution				
**Fingerprint**	**Bit/Smiles**	**Feature Structure**	**Score**	**Carcinogen in training set**
SCFP_6	-1377141613	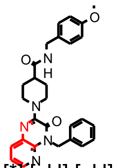 [*][c]1:[*]:[cH]:[cH]:[cH]:[c]:1N=[*]	0.429	3 out of 5

SCFP_6	38222003	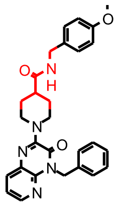 [*]CNC(=O)C(C[*])C[*]	0.415	1 out of 1
SCFP_6	-758850909	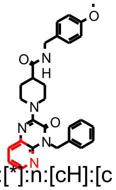 [*][c]1:[*]:n:[cH]:[cH]:[cH]:1	0.355	5 out of 10

Top Features for negative contribution

Fingerprint	Bit/Smiles	Feature Structure	Score	Carcinogen in training set
SCFP_6	-1325723550	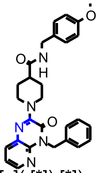 [*]C(=N[c](:[*]):[*])[*]	-0.664	1 out of 9
SCFP_6	1653911926	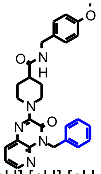 [*][c]1:[cH]:[cH]:[cH]:[cH]:[cH]:1	-0.504	12 out of 64
SCFP_6	399719551	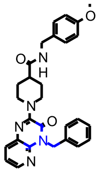 [*]CN(C(=[*])[*])[c](:[*]):[*]	-0.48	2 out of 12

Molecule

TOPKAT_Rat_Male_NTP

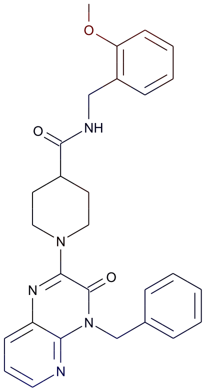

$C_{28}H_{29}N_5O_3$

Molecular Weight: 483.56156

ALogP: 2.942

Rotatable Bonds: 7

Acceptors: 6

Donors: 1

Structural Similar Compounds

Name	Curcumin	Rhodamine 6G	Rotenone
Structure	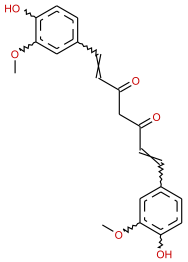	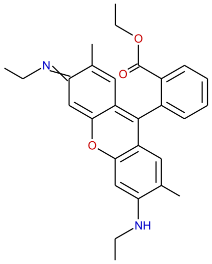	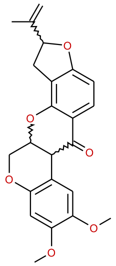
Actual Endpoint	Non-Carcinogen	Carcinogen	Carcinogen
Predicted Endpoint	Non-Carcinogen	Carcinogen	Carcinogen
Distance	0.708	0.742	0.785
Reference	NTP427	NTP364	NTP320

Model Prediction

Prediction: Non-Carcinogen

Probability: 0.492

Enrichment: 0.966

Bayesian Score: -2.86

Mahalanobis Distance: 10.6

Mahalanobis Distance p-value: 8.98e-005

Prediction: Positive if the Bayesian score is above the estimated best cutoff value from minimizing the false positive and false negative rate.
Probability: The esimated probability that the sample is in the positive category. This assumes that the Bayesian score follows a normal distribution and is different from the prediction using a cutoff.
Enrichment: An estimate of enrichment, that is, the increased likelihood (versus random) of this sample being in the category.
Bayesian Score: The standard Laplacian-modified Bayesian score.
Mahalanobis Distance: The Mahalanobis distance (MD) is the distance to the center of the training data. The larger the MD, the less trustworthy the prediction.
Mahalanobis Distance p-value: The p-value gives the fraction of training data with an MD greater than or equal to the one for the given sample, assuming normally distributed data. The smaller the p-value, the less trustworthy the prediciton. For highly non-normal X properties (e.g., fingerprints), the MD p-value is wildly inaccurate.

Model Applicability

Unknown features are fingerprint features in the query molecule, but not found or appearing too infrequently in the training set.

1. OPS PC4 out of range. Value: 7.5477. Training min, max, SD, explained variance: -4.722, 6.6535, 2.046, 0.0759.
2. Unknown ECFP_2 feature: 671679640: [*]\N=C(\N([*])[*])/C(=[*])[*]
3. Unknown ECFP_2 feature: 2085698692: [*]C(=N[c](:[*]):[*])[*]
4. Unknown ECFP_2 feature: 1951894094: [*]CN(C[*])C(=[*])[*]
5. Unknown ECFP_2 feature: -597295171: [*][c](:[*]):[c](:[cH]:[*])N=[*]
6. Unknown ECFP_2 feature: -81134287: [*]NC(=O)C([*])[*]
7. Unknown ECFP_2 feature: -44121127: [*]N([*])C[c](:[*]):[*]

Feature Contribution

Top features for positive contribution

Fingerprint	Bit/Smiles	Feature Structure	Score	Carcinogen in training set

ECFP_12	1680623188	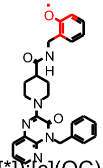 [*][c](:[*]):[c](OC):[cH]:[*]	0.405	14 out of 18
ECFP_12	1408898974	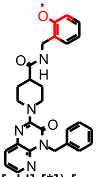 [*]O[c](:[cH]:[*]):[c]([*]):[*]	0.356	14 out of 19
ECFP_12	1307307440	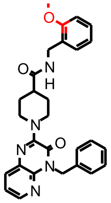 [*]:[c](:[*])OC	0.348	16 out of 22
**Top Features for negative contribution**				
**Fingerprint**	**Bit/Smiles**	**Feature Structure**	**Score**	**Carcinogen in training set**
ECFP_12	-2024509555	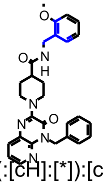 [*]C[c](:[cH]:[*]):[c]([*]):[*]	-0.916	0 out of 3
ECFP_12	-677309799	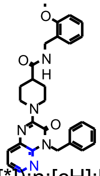 [*][c](:[*]):n:[cH]:[*]	-0.606	2 out of 9

ECFP_12	1171844666	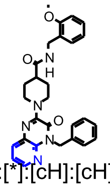 [*][c]1:[*]:[cH]:[cH]:[cH]:n:1	-0.56	1 out of 5

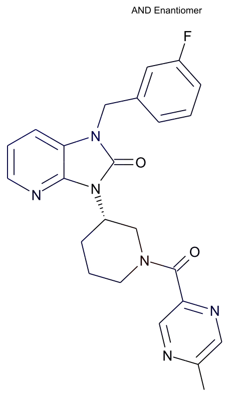

$C_{24}H_{23}FN_6O_2$
Molecular Weight: 446.47682
ALogP: 2.244
Rotatable Bonds: 4
Acceptors: 5
Donors: 0

Structural Similar Compounds

Name	Rotenone	3;3'-Dimethoxybenzidine-4-4'-diisocyanate	Scopolamine hydrobromide trihydrate
Structure	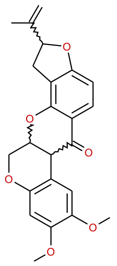	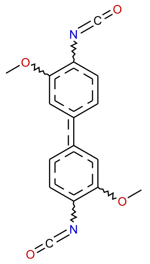	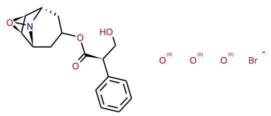
Actual Endpoint	Carcinogen	Carcinogen	Non-Carcinogen
Predicted Endpoint	Carcinogen	Carcinogen	Non-Carcinogen
Distance	0.675	0.743	0.761
Reference	NTP320	NTP/TR-128	NTP445

Model Prediction

Prediction: Non-Carcinogen
Probability: 0.3
Enrichment: 0.59
Bayesian Score: -6.33
Mahalanobis Distance: 12.1
Mahalanobis Distance p-value: 9.73e-008

Prediction: Positive if the Bayesian score is above the estimated best cutoff value from minimizing the false positive and false negative rate.
Probability: The esimated probability that the sample is in the positive category. This assumes that the Bayesian score follows a normal distribution and is different from the prediction using a cutoff.
Enrichment: An estimate of enrichment, that is, the increased likelihood (versus random) of this sample being in the category.
Bayesian Score: The standard Laplacian-modified Bayesian score.
Mahalanobis Distance: The Mahalanobis distance (MD) is the distance to the center of the training data. The larger the MD, the less trustworthy the prediction.
Mahalanobis Distance p-value: The p-value gives the fraction of training data with an MD greater than or equal to the one for the given sample, assuming normally distributed data. The smaller the p-value, the less trustworthy the prediciton. For highly non-normal X properties (e.g., fingerprints), the MD p-value is wildly inaccurate.

Model Applicability

Unknown features are fingerprint features in the query molecule, but not found or appearing too infrequently in the training set.

1. All properties and OPS components are within expected ranges.
2. Unknown ECFP_2 feature: -957084426: [*]C([*])N1C(=[*])[*][*]:[c]1:[*]
3. Unknown ECFP_2 feature: -1102925512: [*]CN(C[*])C(=[*])[*]
4. Unknown ECFP_2 feature: 2077298510: [*]N([*])C(=O)[c](:[*]):[*]
5. Unknown ECFP_2 feature: -1869628272: [*]CC(C[*])N([*])[*]
6. Unknown ECFP_2 feature: -44121127: [*]N([*])C[c](:[*]):[*]
7. Unknown ECFP_2 feature: -176686665: [*]:[cH]:[c](F):[cH]:[*]
8. Unknown ECFP_2 feature: 220735655: [*]:[c](:[*])F

Feature Contribution

Top features for positive contribution

Fingerprint	Bit/Smiles	Feature Structure	Score	Carcinogen in training set

ECFP_12	-756348342	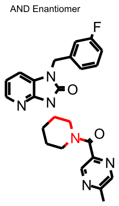 [*]C([*])CN([*])[*]	0.288	1 out of 1
ECFP_12	-509950643	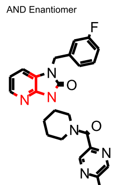 [*]N([*])[c](:n:[*]):[c]([*]):[*]	0.288	1 out of 1
ECFP_12	2106656448	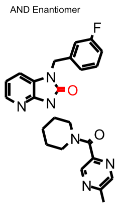 [*]C(=O)[*]	0.227	26 out of 41
**Top Features for negative contribution**				
**Fingerprint**	**Bit/Smiles**	**Feature Structure**	**Score**	**Carcinogen in training set**
ECFP_12	-1332781180	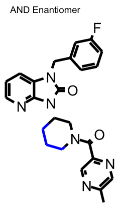 [*]CCC[*]	-1.01	1 out of 9
ECFP_12	-677309799	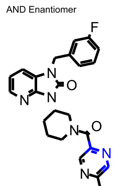 [*][c](:[*]):n:[cH]:[*]	-0.606	2 out of 9

ECFP_12	1171844666	AND Enantiomer 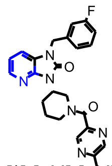 [*][c]1:[*]:[cH]:[cH]:[cH]:n:1	-0.56	1 out of 5

#UNDEFINED

TOPKAT_Rat_Male_NTP

AND Enantiomer

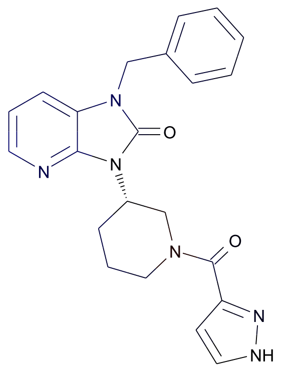

$C_{22}H_{22}N_6O_2$
Molecular Weight: 402.44907
ALogP: 2.501
Rotatable Bonds: 4
Acceptors: 4
Donors: 1

Model Prediction

Prediction: Non-Carcinogen
Probability: 0.318
Enrichment: 0.625
Bayesian Score: -6.01
Mahalanobis Distance: 9.43
Mahalanobis Distance p-value: 0.0068

Prediction: Positive if the Bayesian score is above the estimated best cutoff value from minimizing the false positive and false negative rate.
Probability: The esimated probability that the sample is in the positive category. This assumes that the Bayesian score follows a normal distribution and is different from the prediction using a cutoff.
Enrichment: An estimate of enrichment, that is, the increased likelihood (versus random) of this sample being in the category.
Bayesian Score: The standard Laplacian-modified Bayesian score.
Mahalanobis Distance: The Mahalanobis distance (MD) is the distance to the center of the training data. The larger the MD, the less trustworthy the prediction.
Mahalanobis Distance p-value: The p-value gives the fraction of training data with an MD greater than or equal to the one for the given sample, assuming normally distributed data. The smaller the p-value, the less trustworthy the prediciton. For highly non-normal X properties (e.g., fingerprints), the MD p-value is wildly inaccurate.

Structural Similar Compounds

Name	Chlorendic Acid	Chlorendic Acid	Acetohexamide
Structure	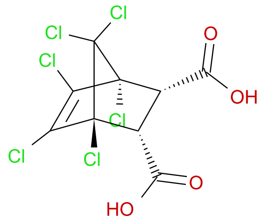	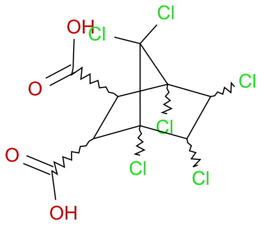	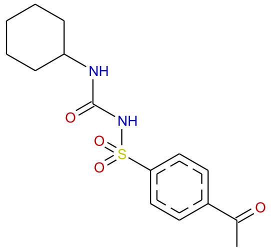
Actual Endpoint	Carcinogen	Carcinogen	Non-Carcinogen
Predicted Endpoint	Carcinogen	Carcinogen	Non-Carcinogen
Distance	0.669	0.672	0.687
Reference	NTP304	NTP/TR-304	NTP/TR-050

Model Applicability

Unknown features are fingerprint features in the query molecule, but not found or appearing too infrequently in the training set.

1. All properties and OPS components are within expected ranges.
2. Unknown ECFP_2 feature: -957084426: [*]C([*])N1C(=[*])[*][*]:[c]1:[*]
3. Unknown ECFP_2 feature: -1102925512: [*]CN(C[*])C(=[*])[*]
4. Unknown ECFP_2 feature: 2077298510: [*]N([*])C(=O)[c](:[*]):[*]
5. Unknown ECFP_2 feature: -1869628272: [*]CC(C[*])N([*])[*]
6. Unknown ECFP_2 feature: 600440273: [*][c]1:[*]:[*]:[nH]:n:1
7. Unknown ECFP_2 feature: -954588747: [*]1:[*]:n:[nH]:[cH]:1
8. Unknown ECFP_2 feature: -44121127: [*]N([*])C[c](:[*]):[*]
9. Unknown ECFP_2 feature: 1998023064: [*]1:[*]:[cH]:[cH]:[nH]:1

Feature Contribution

Top features for positive contribution				
Fingerprint	Bit/Smiles	Feature Structure	Score	Carcinogen in training set

ECFP_12	-509950643	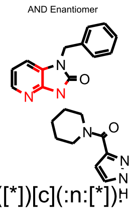 [*]N([*])[c](:n:[*]):[c]([*]):[*]	0.288	1 out of 1
ECFP_12	-756348342	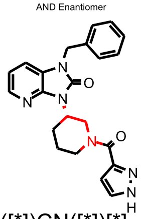 [*]C([*])CN([*])[*]	0.288	1 out of 1
ECFP_12	2106656448	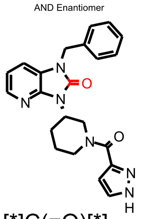 [*]C(=O)[*]	0.227	26 out of 41

Top Features for negative contribution

**Fingerprint**	**Bit/Smiles**	**Feature Structure**	**Score**	**Carcinogen in training set**
ECFP_12	-1332781180	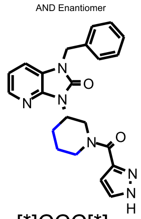 [*]CCC[*]	-1.01	1 out of 9
ECFP_12	-677309799	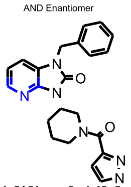 [*][c](:[*]):n:[cH]:[*]	-0.606	2 out of 9

ECFP_12	1171844666	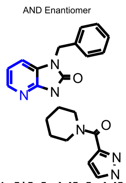 [*][c]1:[*]:[cH]:[cH]:[cH]:n:1	-0.56	1 out of 5

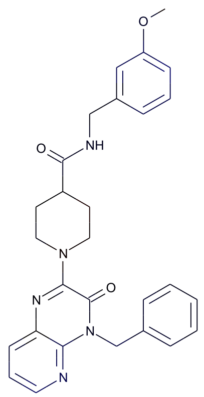

$C_{28}H_{29}N_5O_3$
Molecular Weight: 483.56156
ALogP: 2.942
Rotatable Bonds: 7
Acceptors: 6
Donors: 1

Model Prediction

Prediction: Non-Carcinogen
Probability: 0.371
Enrichment: 0.73
Bayesian Score: -5.07
Mahalanobis Distance: 14.7
Mahalanobis Distance p-value: 1.69e-014

Prediction: Positive if the Bayesian score is above the estimated best cutoff value from minimizing the false positive and false negative rate.
Probability: The esimated probability that the sample is in the positive category. This assumes that the Bayesian score follows a normal distribution and is different from the prediction using a cutoff.
Enrichment: An estimate of enrichment, that is, the increased likelihood (versus random) of this sample being in the category.
Bayesian Score: The standard Laplacian-modified Bayesian score.
Mahalanobis Distance: The Mahalanobis distance (MD) is the distance to the center of the training data. The larger the MD, the less trustworthy the prediction.
Mahalanobis Distance p-value: The p-value gives the fraction of training data with an MD greater than or equal to the one for the given sample, assuming normally distributed data. The smaller the p-value, the less trustworthy the prediciton. For highly non-normal X properties (e.g., fingerprints), the MD p-value is wildly inaccurate.

Structural Similar Compounds

Name	Curcumin	Rhodamine 6G	Rotenone
Structure	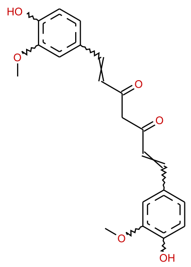	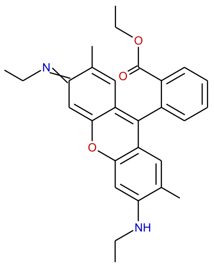	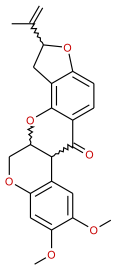
Actual Endpoint	Non-Carcinogen	Carcinogen	Carcinogen
Predicted Endpoint	Non-Carcinogen	Carcinogen	Carcinogen
Distance	0.712	0.741	0.787
Reference	NTP427	NTP364	NTP320

Model Applicability

Unknown features are fingerprint features in the query molecule, but not found or appearing too infrequently in the training set.

1. All properties and OPS components are within expected ranges.
2. Unknown ECFP_2 feature: 671679640: [*]\N=C(\N([*])[*])/C(=[*])[*]
3. Unknown ECFP_2 feature: 2085698692: [*]C(=N[c](:[*]):[*])[*]
4. Unknown ECFP_2 feature: 1951894094: [*]CN(C[*])C(=[*])[*]
5. Unknown ECFP_2 feature: -597295171: [*][c](:[*]):[c](:[cH]:[*])N=[*]
6. Unknown ECFP_2 feature: -81134287: [*]NC(=O)C([*])[*]
7. Unknown ECFP_2 feature: -44121127: [*]N([*])C[c](:[*]):[*]

Feature Contribution

Top features for positive contribution

Fingerprint	Bit/Smiles	Feature Structure	Score	Carcinogen in training set
ECFP_12	1307307440	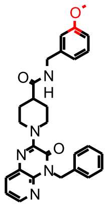 [*]:[c](:[*])OC	0.348	16 out of 22

ECFP_12	-857146788	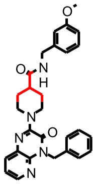 [*]CC(C[*])C(=[*])[*]	0.288	1 out of 1
ECFP_12	-509950643	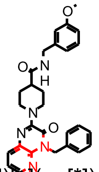 [*]N([*])[c](:n:[*]):[c]([*]):[*]	0.288	1 out of 1

Top Features for negative contribution

Fingerprint	Bit/Smiles	Feature Structure	Score	Carcinogen in training set
ECFP_12	432225086	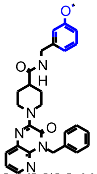 [*]O[c]1:[cH]:[*]:[cH]:[cH]:[cH]:1	-0.693	0 out of 2
ECFP_12	-677309799	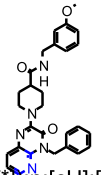 [*][c](:[*]):n:[cH]:[*]	-0.606	2 out of 9
ECFP_12	1171844666	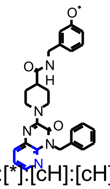 [*][c]1:[*]:[cH]:[cH]:[cH]:n:1	-0.56	1 out of 5

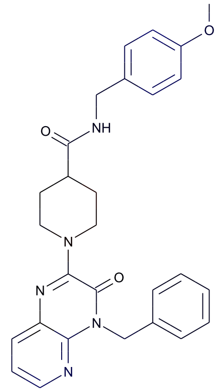

$C_{28}H_{29}N_5O_3$
Molecular Weight: 483.56156
ALogP: 2.942
Rotatable Bonds: 7
Acceptors: 6
Donors: 1

Model Prediction

Prediction: Non-Carcinogen
Probability: 0.361
Enrichment: 0.709
Bayesian Score: -5.26
Mahalanobis Distance: 14.2
Mahalanobis Distance p-value: 4.54e-013

Prediction: Positive if the Bayesian score is above the estimated best cutoff value from minimizing the false positive and false negative rate.
Probability: The esimated probability that the sample is in the positive category. This assumes that the Bayesian score follows a normal distribution and is different from the prediction using a cutoff.
Enrichment: An estimate of enrichment, that is, the increased likelihood (versus random) of this sample being in the category.
Bayesian Score: The standard Laplacian-modified Bayesian score.
Mahalanobis Distance: The Mahalanobis distance (MD) is the distance to the center of the training data. The larger the MD, the less trustworthy the prediction.
Mahalanobis Distance p-value: The p-value gives the fraction of training data with an MD greater than or equal to the one for the given sample, assuming normally distributed data. The smaller the p-value, the less trustworthy the prediciton. For highly non-normal X properties (e.g., fingerprints), the MD p-value is wildly inaccurate.

Structural Similar Compounds

Name	Curcumin	Rhodamine 6G	Rotenone
Structure	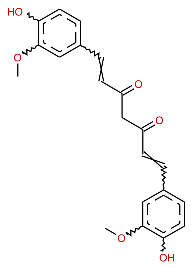	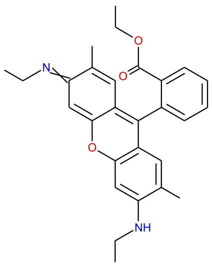	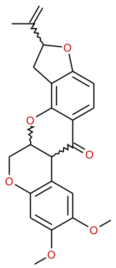
Actual Endpoint	Non-Carcinogen	Carcinogen	Carcinogen
Predicted Endpoint	Non-Carcinogen	Carcinogen	Carcinogen
Distance	0.715	0.743	0.789
Reference	NTP427	NTP364	NTP320

Model Applicability

Unknown features are fingerprint features in the query molecule, but not found or appearing too infrequently in the training set.

1. All properties and OPS components are within expected ranges.
2. Unknown ECFP_2 feature: 671679640: [*]\N=C(\N([*])[*])/C(=[*])[*]
3. Unknown ECFP_2 feature: 2085698692: [*]C(=N[c](:[*]):[*])[*]
4. Unknown ECFP_2 feature: 1951894094: [*]CN(C[*])C(=[*])[*]
5. Unknown ECFP_2 feature: -597295171: [*][c](:[*]):[c](:[cH]:[*])N=[*]
6. Unknown ECFP_2 feature: -81134287: [*]NC(=O)C([*])[*]
7. Unknown ECFP_2 feature: -44121127: [*]N([*])C[c](:[*]):[*]

Feature Contribution

Top features for positive contribution

Fingerprint	Bit/Smiles	Feature Structure	Score	Carcinogen in training set
ECFP_12	1307307440	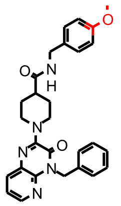 [*]:[c](:[*])OC	0.348	16 out of 22

ECFP_12	769925792	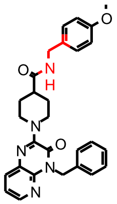 [*]NC[c](:[*]):[*]	0.288	1 out of 1
ECFP_12	-509950643	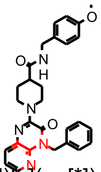 [*]N([*])[c](:n:[*]):[c]([*]):[*]	0.288	1 out of 1
**Top Features for negative contribution**				
**Fingerprint**	**Bit/Smiles**	**Feature Structure**	**Score**	**Carcinogen in training set**
ECFP_12	-677309799	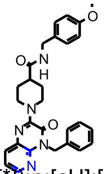 [*][c](:[*]):n:[cH]:[*]	-0.606	2 out of 9
ECFP_12	1171844666	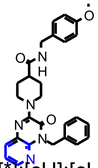 [*][c]1:[*]:[cH]:[cH]:[cH]:n:1	-0.56	1 out of 5
ECFP_12	-1897341097	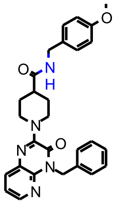 [*]N[*]	-0.429	13 out of 41

#UNDEFINED

TOPKAT_Skin_Irritancy_Mild_vs_Moderate_Severe

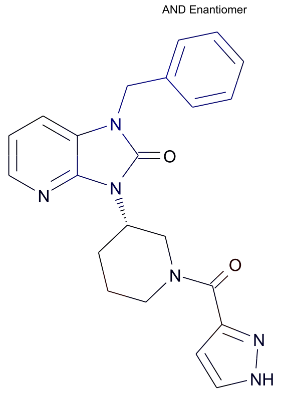

$C_{22}H_{22}N_6O_2$
Molecular Weight: 402.44907
ALogP: 2.501
Rotatable Bonds: 4
Acceptors: 4
Donors: 1

Model Prediction

Prediction: Mild

Probability: 0.121

Enrichment: 0.328

Bayesian Score: -7.57

Mahalanobis Distance: 9.46

Mahalanobis Distance p-value: 0.118

Prediction: Positive if the Bayesian score is above the estimated best cutoff value from minimizing the false positive and false negative rate.
Probability: The esimated probability that the sample is in the positive category. This assumes that the Bayesian score follows a normal distribution and is different from the prediction using a cutoff.
Enrichment: An estimate of enrichment, that is, the increased likelihood (versus random) of this sample being in the category.
Bayesian Score: The standard Laplacian-modified Bayesian score.
Mahalanobis Distance: The Mahalanobis distance (MD) is the distance to the center of the training data. The larger the MD, the less trustworthy the prediction.
Mahalanobis Distance p-value: The p-value gives the fraction of training data with an MD greater than or equal to the one for the given sample, assuming normally distributed data. The smaller the p-value, the less trustworthy the prediciton. For highly non-normal X properties (e.g., fingerprints), the MD p-value is wildly inaccurate.

Structural Similar Compounds

Name	Benzenesulfonic acid, 5-(2H-naphtho(1,2-d)triazol-2-yl)-2-(2-phenyl ethenyl)-, sodium salt	5-Norbornene-2,3-dicarboxylic acid, 1,4,5,6,7,7-hexachloro-	Anthraquinone, 1,1'-iminodi-
Structure	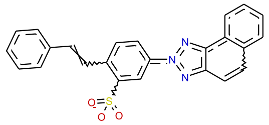	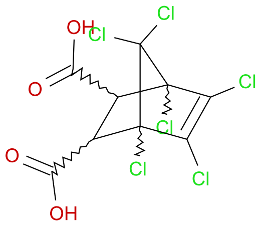	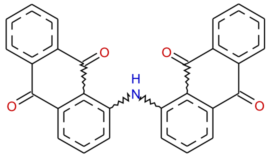
Actual Endpoint	Mild	Mild	Mild
Predicted Endpoint	Mild	Mild	Mild
Distance	0.722	0.730	0.761
Reference	MVCRB3 MVC-Report. (Stockholm, Sweden) No.1-2, 1972-73. Discontinued. Volu me(issue)/page/year: 2,193,1973	85JCAE "Prehled Prumyslove Toxikologie; Organicke Latky," Marhold, J., Prague , Czechoslovakia, Avicenum, 1986 Volume(issue)/page/year: -,581,1986	85JCAE "Prehled Prumyslove Toxikologie; Organicke Latky," Marhold, J., Prague , Czechoslovakia, Avicenum, 1986 Volume(issue)/page/year: -,735,1986

Model Applicability

Unknown features are fingerprint features in the query molecule, but not found or appearing too infrequently in the training set.

1. All properties and OPS components are within expected ranges.
2. Unknown FCFP_2 feature: 1747267175: [*][c]1:[*]:[*]:[nH]:n:1
3. Unknown FCFP_2 feature: 262592487: [*]1:[*]:n:[nH]:[cH]:1
4. Unknown FCFP_2 feature: 1618184456: [*]1:[*]:[cH]:[cH]:[nH]:1

Feature Contribution

Top features for positive contribution

Fingerprint	Bit/Smiles	Feature Structure	Score	Moderate_Severe in training set

FCFP_12	-668483809	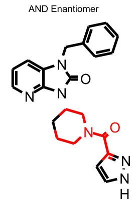 [*][C@H]1[*]CCN(C1)C(=O)[c](:[*]):[*]	0.579	4 out of 5
FCFP_12	-989213044	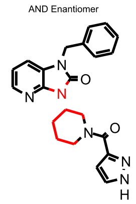 [*]N([*])[C@@H]1C[*]CCC1	0.416	18 out of 32
FCFP_12	-1549163031	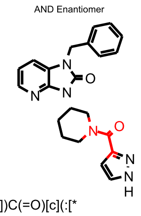 [*]N([*])C(=O)[c](:[*]):[*]	0.371	25 out of 47
**Top Features for negative contribution**				
**Fingerprint**	**Bit/Smiles**	**Feature Structure**	**Score**	**Moderate_Severe in training set**
FCFP_12	675769755	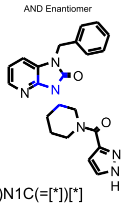 [*]C([*])N1C(=[*])[*][*]:[c]1:[*]	-1.05	1 out of 13
FCFP_12	2106393770	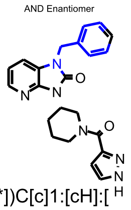 [*]N([*])C[c]1:[cH]:[cH]:[*]:[cH]:[cH]:1	-0.98	1 out of 12

FCFP_12	1390842262	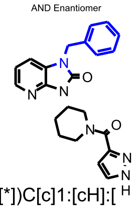 [*]N([*])C[c]1:[cH]:[cH]:[cH]:[cH]:[cH]:1	-0.753	1 out of 9

TOPKAT_Skin_Irritancy_Mild_vs_Moderate_Severe

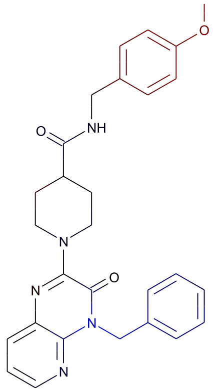

$C_{28}H_{29}N_5O_3$

Molecular Weight: 483.56156

ALogP: 2.942

Rotatable Bonds: 7

Acceptors: 6

Donors: 1

Model Prediction

Prediction: Mild

Probability: 0.206

Enrichment: 0.561

Bayesian Score: -4.99

Mahalanobis Distance: 11.1

Mahalanobis Distance p-value: 0.000577

Prediction: Positive if the Bayesian score is above the estimated best cutoff value from minimizing the false positive and false negative rate.
Probability: The esimated probability that the sample is in the positive category. This assumes that the Bayesian score follows a normal distribution and is different from the prediction using a cutoff.
Enrichment: An estimate of enrichment, that is, the increased likelihood (versus random) of this sample being in the category.
Bayesian Score: The standard Laplacian-modified Bayesian score.
Mahalanobis Distance: The Mahalanobis distance (MD) is the distance to the center of the training data. The larger the MD, the less trustworthy the prediction.
Mahalanobis Distance p-value: The p-value gives the fraction of training data with an MD greater than or equal to the one for the given sample, assuming normally distributed data. The smaller the p-value, the less trustworthy the prediciton. For highly non-normal X properties (e.g., fingerprints), the MD p-value is wildly inaccurate.

Structural Similar Compounds

Name	Pregna-1,4-diene-3,20-dione, 21-(acetyloxy)-11-hydroxy-6-methyl-17- (1-oxopropoxy)-, (6-alpha,11-beta)-	Benzenesulfonic acid, 5-(2H-naphtho(1,2-d)triazol-2-yl)-2-(2-phenyl ethenyl)-, sodium salt	Anthraquinone, 1,1'-iminodi-
Structure	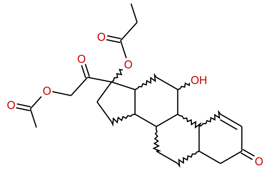	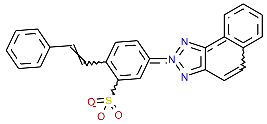	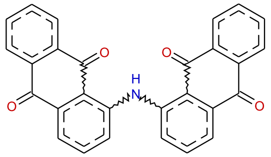
Actual Endpoint	Mild	Mild	Mild
Predicted Endpoint	Mild	Mild	Mild
Distance	0.696	0.779	0.810
Reference	YACHDS Yakuri to Chiryo. Pharmacology and Therapeutics. (Raifu Saiensu Shup pan K.K., 2-5-13, Yaesu, Chuo-ku, Tokyo 104, Japan) V.1-1972- Volume(issue) /page/year: 19,3103,1991	MVCRB3 MVC-Report. (Stockholm, Sweden) No.1-2, 1972-73. Discontinued. Volu me(issue)/page/year: 2,193,1973	85JCAE "Prehled Prumyslove Toxikologie; Organicke Latky," Marhold, J., Prague , Czechoslovakia, Avicenum, 1986 Volume(issue)/page/year: -,735,1986

Model Applicability

Unknown features are fingerprint features in the query molecule, but not found or appearing too infrequently in the training set.

1. All properties and OPS components are within expected ranges.

Feature Contribution

Top features for positive contribution

Fingerprint	Bit/Smiles	Feature Structure	Score	Moderate_Severe in training set

FCFP_12	580453787	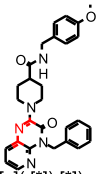 [*]C(=N[c](:[*]):[*])[*]	0.64	5 out of 6
FCFP_12	-9847677	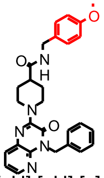 [*][c]1:[cH]:[cH]:[c](OC):[cH]:[cH]:1	0.419	6 out of 10
FCFP_12	-1977641857	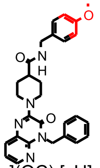 [*]:[cH]:[c](OC):[cH]:[*]	0.416	18 out of 32

Top Features for negative contribution

Fingerprint	Bit/Smiles	Feature Structure	Score	Moderate_Severe in training set
FCFP_12	-885550502	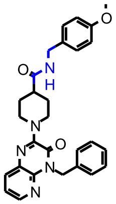 [*]CNC(=[*])[*]	-1.05	2 out of 21
FCFP_12	675769755	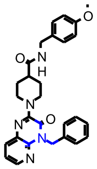 [*]C([*])N1C(=[*])[*][*]:[c]1:[*]	-1.05	1 out of 13

FCFP_12	2106393770	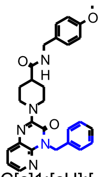<br>[*]N([*])C[c]1:[cH]:[cH]:[*]:[cH]:[cH]:1	-0.98	1 out of 12

Molecule

TOPKAT_Skin_Irritancy_None_vs_Irritant

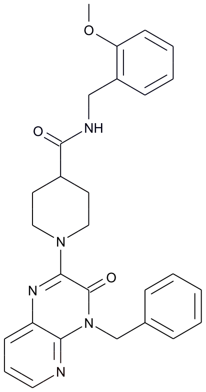

$C_{28}H_{29}N_5O_3$
Molecular Weight: 483.56156
ALogP: 2.942
Rotatable Bonds: 7
Acceptors: 6
Donors: 1

Model Prediction

Prediction: Non-Irritant
Probability: 0.972
Enrichment: 1.06
Bayesian Score: -0.874
Mahalanobis Distance: 14.7
Mahalanobis Distance p-value: 3.5e-013

Prediction: Positive if the Bayesian score is above the estimated best cutoff value from minimizing the false positive and false negative rate.
Probability: The esimated probability that the sample is in the positive category. This assumes that the Bayesian score follows a normal distribution and is different from the prediction using a cutoff.
Enrichment: An estimate of enrichment, that is, the increased likelihood (versus random) of this sample being in the category.
Bayesian Score: The standard Laplacian-modified Bayesian score.
Mahalanobis Distance: The Mahalanobis distance (MD) is the distance to the center of the training data. The larger the MD, the less trustworthy the prediction.
Mahalanobis Distance p-value: The p-value gives the fraction of training data with an MD greater than or equal to the one for the given sample, assuming normally distributed data. The smaller the p-value, the less trustworthy the prediciton. For highly non-normal X properties (e.g., fingerprints), the MD p-value is wildly inaccurate.

Structural Similar Compounds

Name	Pregna-1,4-diene-3,20-dione, 21-(acetyloxy)-11-hydroxy-6-methyl-17- (1-oxopropoxy)-, (6-alpha,11-beta)-	Benzenesulfonic acid, 5-(2H-naphtho(1,2-d)triazol-2-yl)-2-(2-phenyl ethenyl)-, sodium salt	Anthraquinone, 1,1'-iminodi-
Structure	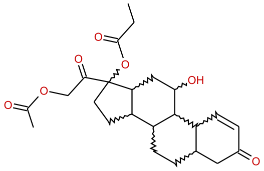	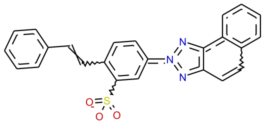	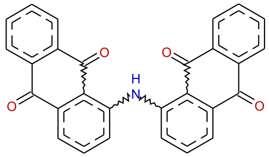
Actual Endpoint	Irritant	Irritant	Irritant
Predicted Endpoint	Irritant	Irritant	Non-Irritant
Distance	0.701	0.777	0.818
Reference	YACHDS Yakuri to Chiryo. Pharmacology and Therapeutics. (Raifu Saiensu Shup pan K.K., 2-5-13, Yaesu, Chuo-ku, Tokyo 104, Japan) V.1-1972- Volume(issue) /page/year: 19,3103,1991	MVCRB3 MVC-Report. (Stockholm, Sweden) No.1-2, 1972-73. Discontinued. Volu me(issue)/page/year: 2,193,1973	85JCAE "Prehled Prumyslove Toxikologie; Organicke Latky," Marhold, J., Prague , Czechoslovakia, Avicenum, 1986 Volume(issue)/page/year: -,735,1986

Model Applicability

Unknown features are fingerprint features in the query molecule, but not found or appearing too infrequently in the training set.

1. All properties and OPS components are within expected ranges.

Feature Contribution

Top features for positive contribution

Fingerprint	Bit/Smiles	Feature Structure	Score	Irritant in training set

FCFP_12	-58290195	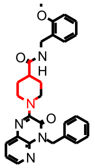 [*]N1[*]CC(CC1)C(=[*])[*]	0.0847	22 out of 22
FCFP_12	-124655670	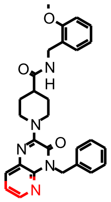 [*]:[cH]:[cH]:n:[*]	0.0821	13 out of 13
FCFP_12	580453787	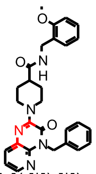 [*]C(=N[c](:[*]):[*])[*]	0.0795	9 out of 9
**Top Features for negative contribution**				
**Fingerprint**	**Bit/Smiles**	**Feature Structure**	**Score**	**Irritant in training set**
FCFP_12	566058135	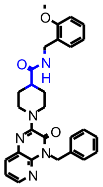 [*]NC(=O)C([*])[*]	-0.367	13 out of 21
FCFP_12	1458856986	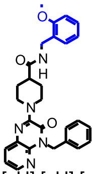 [*]C[c]1:[cH]:[cH]:[cH]:[cH]:[c]:1OC	-0.109	4 out of 5

FCFP_12	675769755	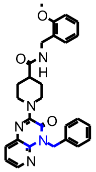<br>[*]CN(C(=[*])[*])[c](<br>:[*]):[*]	-0.088	15 out of 18

#UNDEFINED

TOPKAT_Skin_Irritancy_None_vs_Irritant

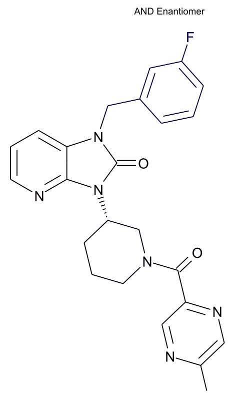

$C_{24}H_{23}FN_6O_2$
Molecular Weight: 446.47682
ALogP: 2.244
Rotatable Bonds: 4
Acceptors: 5
Donors: 0

Model Prediction

Prediction: Non-Irritant
Probability: 0.957
Enrichment: 1.04
Bayesian Score: -1.67
Mahalanobis Distance: 11.1
Mahalanobis Distance p-value: 0.00137

Prediction: Positive if the Bayesian score is above the estimated best cutoff value from minimizing the false positive and false negative rate.
Probability: The esimated probability that the sample is in the positive category. This assumes that the Bayesian score follows a normal distribution and is different from the prediction using a cutoff.
Enrichment: An estimate of enrichment, that is, the increased likelihood (versus random) of this sample being in the category.
Bayesian Score: The standard Laplacian-modified Bayesian score.
Mahalanobis Distance: The Mahalanobis distance (MD) is the distance to the center of the training data. The larger the MD, the less trustworthy the prediction.
Mahalanobis Distance p-value: The p-value gives the fraction of training data with an MD greater than or equal to the one for the given sample, assuming normally distributed data. The smaller the p-value, the less trustworthy the prediciton. For highly non-normal X properties (e.g., fingerprints), the MD p-value is wildly inaccurate.

Structural Similar Compounds

Name	Benzenesulfonic acid, 5-(2H-naphtho(1,2-d)triazol-2-yl)-2-(2-phenyl ethenyl)-, sodium salt	Benzoic acid, p-amidino-, propyl ester	Phosphoric acid, 1,2-dibromo-2,2-dichloroethyl dimethyl ester
Structure	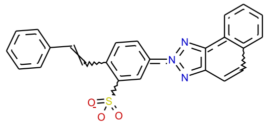	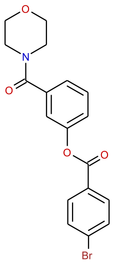	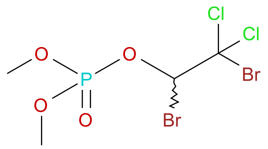
Actual Endpoint	Irritant	Irritant	Irritant
Predicted Endpoint	Irritant	Non-Irritant	Irritant
Distance	0.620	0.665	0.715
Reference	MVCRB3 MVC-Report. (Stockholm, Sweden) No.1-2, 1972-73. Discontinued. Volu me(issue)/page/year: 2,193,1973	JAPMA8 Journal of the American Pharmaceutical Association, Scientific Edition . (Washington, DC) V.29-49, 1940-60. For publisher information, see JPMSAE. V olume(issue)/page/year: 41,202,1952	TXAPA9 Toxicology and Applied Pharmacology. (Academic Press, Inc., 1 E. Firs t St., Duluth, MN 55802) V.1- 1959- Volume(issue)/page/year: 21,369,1972

Model Applicability

Unknown features are fingerprint features in the query molecule, but not found or appearing too infrequently in the training set.

1. All properties and OPS components are within expected ranges.

Feature Contribution

Top features for positive contribution				
**Fingerprint**	**Bit/Smiles**	**Feature Structure**	**Score**	**Irritant in training set**

FCFP_12	-124655670	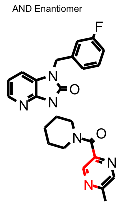 [*]:[cH]:[cH]:n:[*]	0.0821	13 out of 13
FCFP_12	-1986158408	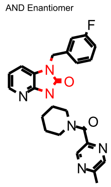 [*]N1[*]:[*]N([*])C1=O	0.0821	13 out of 13
FCFP_12	-1539132615	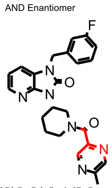 [*]C(=[*])[c](:[cH]:[*]):n:[*]	0.0795	9 out of 9

Top Features for negative contribution

Fingerprint	Bit/Smiles	Feature Structure	Score	Irritant in training set
FCFP_12	-1700637232	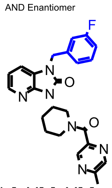 [*]C[c]1:[cH]:[cH]:[cH]:[c](F):[cH]:1	-0.846	1 out of 4
FCFP_12	-668483809	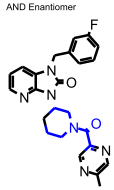 [*][C@H]1[*]CCN(C1)C(=O)[c](:[*]):[*]	-0.173	6 out of 8

FCFP_12	367998008	AND Enantiomer 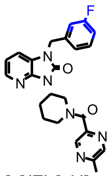 [*]:[cH]:[c](F):[cH]:[*]	-0.129	61 out of 76

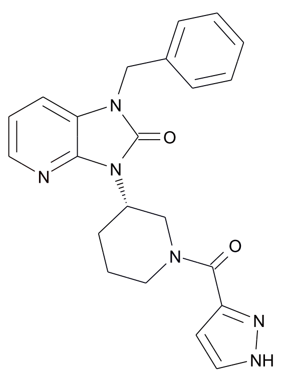

$C_{22}H_{22}N_6O_2$
Molecular Weight: 402.44907
ALogP: 2.501
Rotatable Bonds: 4
Acceptors: 4
Donors: 1

Model Prediction

Prediction: Irritant
Probability: 0.974
Enrichment: 1.06
Bayesian Score: -0.64
Mahalanobis Distance: 10.3
Mahalanobis Distance p-value: 0.0246

Prediction: Positive if the Bayesian score is above the estimated best cutoff value from minimizing the false positive and false negative rate.
Probability: The esimated probability that the sample is in the positive category. This assumes that the Bayesian score follows a normal distribution and is different from the prediction using a cutoff.
Enrichment: An estimate of enrichment, that is, the increased likelihood (versus random) of this sample being in the category.
Bayesian Score: The standard Laplacian-modified Bayesian score.
Mahalanobis Distance: The Mahalanobis distance (MD) is the distance to the center of the training data. The larger the MD, the less trustworthy the prediction.
Mahalanobis Distance p-value: The p-value gives the fraction of training data with an MD greater than or equal to the one for the given sample, assuming normally distributed data. The smaller the p-value, the less trustworthy the prediciton. For highly non-normal X properties (e.g., fingerprints), the MD p-value is wildly inaccurate.

Structural Similar Compounds

Name	Benzenesulfonic acid, 5-(2H-naphtho(1,2-d)triazol-2-yl)-2-(2-phenyl ethenyl)-, sodium salt	5-Norbornene-2,3-dicarboxylic acid, 1,4,5,6,7,7-hexachloro-	Benzoic acid, p-amidino-, propyl ester
Structure	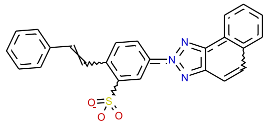	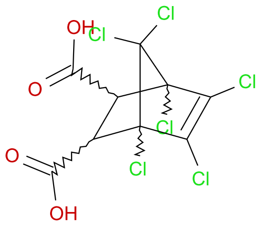	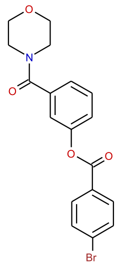
Actual Endpoint	Irritant	Irritant	Irritant
Predicted Endpoint	Irritant	Irritant	Non-Irritant
Distance	0.718	0.724	0.727
Reference	MVCRB3 MVC-Report. (Stockholm, Sweden) No.1-2, 1972-73. Discontinued. Volu me(issue)/page/year: 2,193,1973	85JCAE "Prehled Prumyslove Toxikologie; Organicke Latky," Marhold, J., Prague , Czechoslovakia, Avicenum, 1986 Volume(issue)/page/year: -,581,1986	JAPMA8 Journal of the American Pharmaceutical Association, Scientific Edition . (Washington, DC) V.29-49, 1940-60. For publisher information, see JPMSAE. V olume(issue)/page/year: 41,202,1952

Model Applicability

Unknown features are fingerprint features in the query molecule, but not found or appearing too infrequently in the training set.

1. All properties and OPS components are within expected ranges.
2. Unknown FCFP_2 feature: 1747267175: [*][c]1:[*]:[*]:[nH]:n:1
3. Unknown FCFP_2 feature: 262592487: [*]1:[*]:n:[nH]:[cH]:1
4. Unknown FCFP_2 feature: 1618184456: [*]1:[*]:[cH]:[cH]:[nH]:1

Feature Contribution

Top features for positive contribution				
Fingerprint	Bit/Smiles	Feature Structure	Score	Irritant in training set

FCFP_12	-1986158408	AND Enantiomer 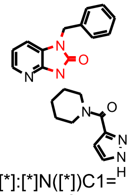 [*]N1[*]:[*]N([*])C1=O	0.0821	13 out of 13
FCFP_12	-124655670	AND Enantiomer 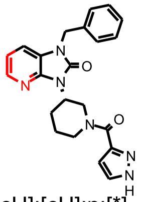 [*]:[cH]:[cH]:n:[*]	0.0821	13 out of 13
FCFP_12	-1539132615	AND Enantiomer 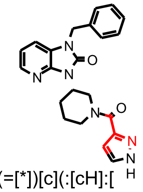 [*]C(=[*])[c](:[cH]:[*]):n:[*]	0.0795	9 out of 9

Top Features for negative contribution

Fingerprint	Bit/Smiles	Feature Structure	Score	Irritant in training set
FCFP_12	-668483809	AND Enantiomer 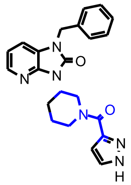 [*][C@H]1[*]CCN(C1)C(=O)[c](:[*]):[*]	-0.173	6 out of 8
FCFP_12	675769755	AND Enantiomer 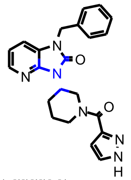 [*]CN(C(=[*])[*])[c](:[*]):[*]	-0.088	15 out of 18

FCFP_12	1674451008	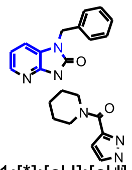 [*][c]1:[*]:[cH]:[cH]:[cH]:[c]:1N=[*]	-0.0873	93 out of 111

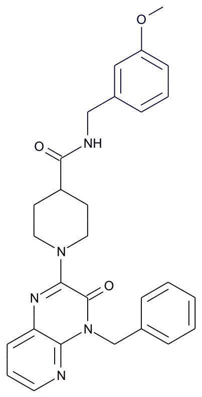

$C_{28}H_{29}N_5O_3$

Molecular Weight: 483.56156

ALogP: 2.942

Rotatable Bonds: 7

Acceptors: 6

Donors: 1

Model Prediction

Prediction: Non-Irritant

Probability: 0.96

Enrichment: 1.04

Bayesian Score: -1.54

Mahalanobis Distance: 14.7

Mahalanobis Distance p-value: 3.5e-013

Prediction: Positive if the Bayesian score is above the estimated best cutoff value from minimizing the false positive and false negative rate.
Probability: The esimated probability that the sample is in the positive category. This assumes that the Bayesian score follows a normal distribution and is different from the prediction using a cutoff.
Enrichment: An estimate of enrichment, that is, the increased likelihood (versus random) of this sample being in the category.
Bayesian Score: The standard Laplacian-modified Bayesian score.
Mahalanobis Distance: The Mahalanobis distance (MD) is the distance to the center of the training data. The larger the MD, the less trustworthy the prediction.
Mahalanobis Distance p-value: The p-value gives the fraction of training data with an MD greater than or equal to the one for the given sample, assuming normally distributed data. The smaller the p-value, the less trustworthy the prediciton. For highly non-normal X properties (e.g., fingerprints), the MD p-value is wildly inaccurate.

Structural Similar Compounds

Name	Pregna-1,4-diene-3,20-dione, 21-(acetyloxy)-11-hydroxy-6-methyl-17- (1-oxopropoxy)-, (6-alpha,11-beta)-	Benzenesulfonic acid, 5-(2H-naphtho(1,2-d)triazol-2-yl)-2-(2-phenyl ethenyl)-, sodium salt	Anthraquinone, 1,1'-iminodi-
Structure	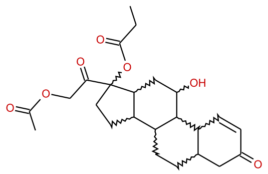	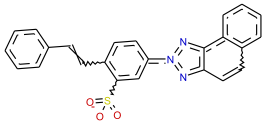	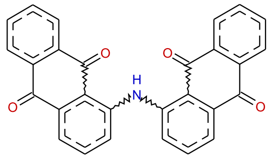
Actual Endpoint	Irritant	Irritant	Irritant
Predicted Endpoint	Irritant	Irritant	Non-Irritant
Distance	0.702	0.777	0.819
Reference	YACHDS Yakuri to Chiryo. Pharmacology and Therapeutics. (Raifu Saiensu Shup pan K.K., 2-5-13, Yaesu, Chuo-ku, Tokyo 104, Japan) V.1- 1972- Volume(issue) /page/year: 19,3103,1991	MVCRB3 MVC-Report. (Stockholm, Sweden) No.1-2, 1972-73. Discontinued. Volu me(issue)/page/year: 2,193,1973	85JCAE "Prehled Prumyslove Toxikologie; Organicke Latky," Marhold, J., Prague , Czechoslovakia, Avicenum, 1986 Volume(issue)/page/year: -,735,1986

Model Applicability

Unknown features are fingerprint features in the query molecule, but not found or appearing too infrequently in the training set.

1. All properties and OPS components are within expected ranges.

Feature Contribution

Top features for positive contribution				
Fingerprint	Bit/Smiles	Feature Structure	Score	Irritant in training set

FCFP_12	-58290195	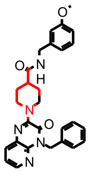 [*]N1[*]CC(CC1)C(=[*])[*]	0.0847	22 out of 22
FCFP_12	-124655670	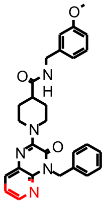 [*]:[cH]:[cH]:n:[*]	0.0821	13 out of 13
FCFP_12	580453787	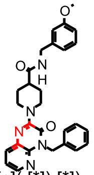 [*]C(=N[c](:[*]):[*])[*]	0.0795	9 out of 9
**Top Features for negative contribution**				
**Fingerprint**	**Bit/Smiles**	**Feature Structure**	**Score**	**Irritant in training set**
FCFP_12	-1757681964	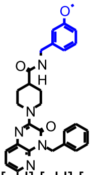 [*]C[c]1:[cH]:[cH]:[cH]:[c](OC):[cH]:1	-0.627	1 out of 3
FCFP_12	566058135	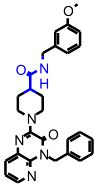 [*]NC(=O)C([*])[*]	-0.367	13 out of 21

FCFP_12	-1695347203	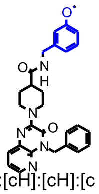 [*]C[c]1:[cH]:[cH]:[cH]:[c](O[*]):[cH]:1	-0.261	4 out of 6

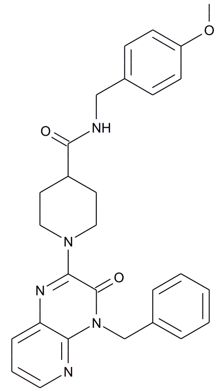

$C_{28}H_{29}N_5O_3$
Molecular Weight: 483.56156
ALogP: 2.942
Rotatable Bonds: 7
Acceptors: 6
Donors: 1

Model Prediction

Prediction: Irritant
Probability: 0.974
Enrichment: 1.06
Bayesian Score: -0.708
Mahalanobis Distance: 14.7
Mahalanobis Distance p-value: 3.5e-013

Prediction: Positive if the Bayesian score is above the estimated best cutoff value from minimizing the false positive and false negative rate.
Probability: The esimated probability that the sample is in the positive category. This assumes that the Bayesian score follows a normal distribution and is different from the prediction using a cutoff.
Enrichment: An estimate of enrichment, that is, the increased likelihood (versus random) of this sample being in the category.
Bayesian Score: The standard Laplacian-modified Bayesian score.
Mahalanobis Distance: The Mahalanobis distance (MD) is the distance to the center of the training data. The larger the MD, the less trustworthy the prediction.
Mahalanobis Distance p-value: The p-value gives the fraction of training data with an MD greater than or equal to the one for the given sample, assuming normally distributed data. The smaller the p-value, the less trustworthy the prediciton. For highly non-normal X properties (e.g., fingerprints), the MD p-value is wildly inaccurate.

Structural Similar Compounds

Name	Pregna-1,4-diene-3,20-dione, 21-(acetyloxy)-11-hydroxy-6-methyl-17- (1-oxopropoxy)-, (6-alpha,11-beta)-	Benzenesulfonic acid, 5-(2H-naphtho(1,2-d)triazol-2-yl)-2-(2-phenyl ethenyl)-, sodium salt	Anthraquinone, 1,1'-iminodi-
Structure	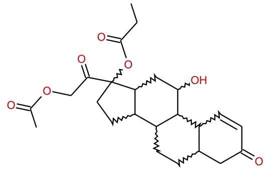	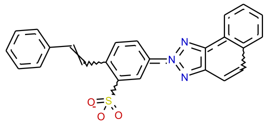	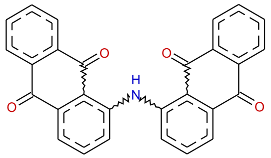
Actual Endpoint	Irritant	Irritant	Irritant
Predicted Endpoint	Irritant	Irritant	Non-Irritant
Distance	0.702	0.777	0.819
Reference	YACHDS Yakuri to Chiryo. Pharmacology and Therapeutics. (Raifu Saiensu Shup pan K.K., 2-5-13, Yaesu, Chuo-ku, Tokyo 104, Japan) V.1-1972- Volume(issue) /page/year: 19,3103,1991	MVCRB3 MVC-Report. (Stockholm, Sweden) No.1-2, 1972-73. Discontinued. Volu me(issue)/page/year: 2,193,1973	85JCAE "Prehled Prumyslove Toxikologie; Organicke Latky," Marhold, J., Prague , Czechoslovakia, Avicenum, 1986 Volume(issue)/page/year: -,735,1986

Model Applicability

Unknown features are fingerprint features in the query molecule, but not found or appearing too infrequently in the training set.

1. All properties and OPS components are within expected ranges.

Feature Contribution

Top features for positive contribution				
Fingerprint	Bit/Smiles	Feature Structure	Score	Irritant in training set

FCFP_12	-58290195	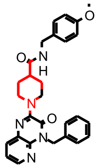 [*]N1[*]CC(CC1)C(=[*])[*]	0.0847	22 out of 22
FCFP_12	-124655670	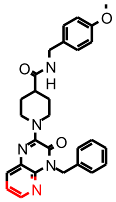 [*]:[cH]:[cH]:n:[*]	0.0821	13 out of 13
FCFP_12	580453787	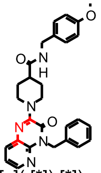 [*]C(=N[c](:[*]):[*])[*]	0.0795	9 out of 9

Top Features for negative contribution

Fingerprint	Bit/Smiles	Feature Structure	Score	Irritant in training set
FCFP_12	566058135	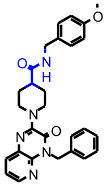 [*]NC(=O)C([*])[*]	-0.367	13 out of 21
FCFP_12	346218766	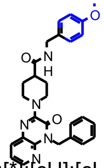 [*][c]1:[*]:[cH]:[cH]:[c](OC):[cH]:1	-0.0911	35 out of 42

FCFP_12	675769755	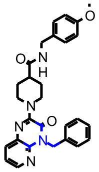<br>[*]CN(C(=[*])[*])[c](:[*]):[*]	-0.088	15 out of 18

Molecule

TOPKAT_Skin_Sensitization_None_vs_Sensitizer

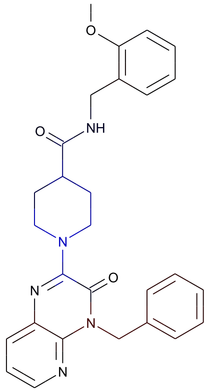

$C_{28}H_{29}N_5O_3$

Molecular Weight: 483.56156

ALogP: 2.942

Rotatable Bonds: 7

Acceptors: 6

Donors: 1

Model Prediction

Prediction: Non-Sensitizer

Probability: 0.647

Enrichment: 0.943

Bayesian Score: -2.65

Mahalanobis Distance: 10.1

Mahalanobis Distance p-value: 1.56e-005

Prediction: Positive if the Bayesian score is above the estimated best cutoff value from minimizing the false positive and false negative rate.
Probability: The esimated probability that the sample is in the positive category. This assumes that the Bayesian score follows a normal distribution and is different from the prediction using a cutoff.
Enrichment: An estimate of enrichment, that is, the increased likelihood (versus random) of this sample being in the category.
Bayesian Score: The standard Laplacian-modified Bayesian score.
Mahalanobis Distance: The Mahalanobis distance (MD) is the distance to the center of the training data. The larger the MD, the less trustworthy the prediction.
Mahalanobis Distance p-value: The p-value gives the fraction of training data with an MD greater than or equal to the one for the given sample, assuming normally distributed data. The smaller the p-value, the less trustworthy the prediciton. For highly non-normal X properties (e.g., fingerprints), the MD p-value is wildly inaccurate.

Structural Similar Compounds

Name	Mometasone furoate	Tixocortol pivalate	Budesonide
Structure	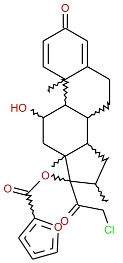	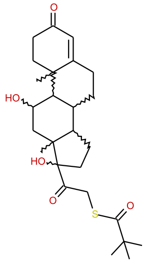	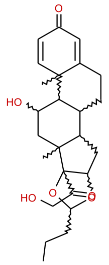
Actual Endpoint	Sensitizer	Sensitizer	Sensitizer
Predicted Endpoint	Sensitizer	Sensitizer	Sensitizer
Distance	0.638	0.695	0.727
Reference	Contact Dermatitis (1996) 34:161	Contact Dermatitis (1996) 34:161	Contact Dermatitis (1996) 34:161

Model Applicability

Unknown features are fingerprint features in the query molecule, but not found or appearing too infrequently in the training set.

1. All properties and OPS components are within expected ranges.

Feature Contribution

Top features for positive contribution

Fingerprint	Bit/Smiles	Feature Structure	Score	Sensitizer in training set
FCFP_12	907036844	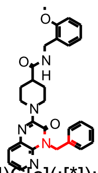 [*]N([*])C[c](:[*]):[*]	0.29	9 out of 9

FCFP_12	2106393770	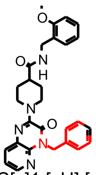 [*]N([*])C[c]1:[cH]:[cH]:[*]:[cH]:[cH]:1	0.281	7 out of 7
FCFP_12	1390842262	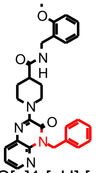 [*]N([*])C[c]1:[cH]:[cH]:[cH]:[cH]:[cH]:1	0.281	7 out of 7

Top Features for negative contribution

Fingerprint	Bit/Smiles	Feature Structure	Score	Sensitizer in training set
FCFP_12	-1474971978	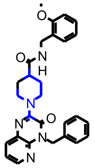 [*]C1[*]CN(CC1)C(=[*])[*]	-1.53	0 out of 5
FCFP_12	-1553874037	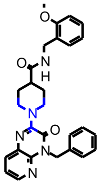 [*]CN(C[*])C(=[*])[*]	-0.802	3 out of 11
FCFP_12	1743817318	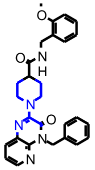 [*]\N=C(\N1CC[*]CC1)/C(=[*])[*]	-0.542	0 out of 1

#UNDEFINED

TOPKAT_Skin_Sensitization_None_vs_Sensitizer

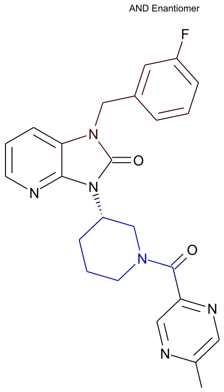

$C_{24}H_{23}FN_6O_2$
Molecular Weight: 446.47682
ALogP: 2.244
Rotatable Bonds: 4
Acceptors: 5
Donors: 0

Structural Similar Compounds

Name	Mometasone furoate	3-(benzenesulfonyloxymethyl)-5;5-dimethyldihydro-2(3H)-furanone	1;3-bis(4-Methoxyphenyl)-propane-1;3-dione
Structure	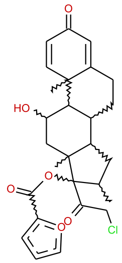	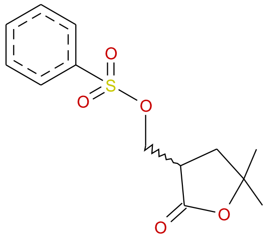	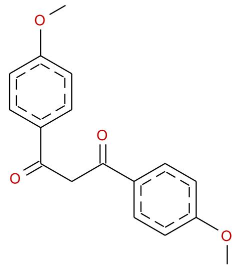
Actual Endpoint	Sensitizer	Sensitizer	Non-Sensitizer
Predicted Endpoint	Sensitizer	Sensitizer	Non-Sensitizer
Distance	0.714	0.808	0.810
Reference	Contact Dermatitis (1996) 34:161	David A Basketter (priv comm)	SAR and QSAR in Env Res (1994) 2:159

Model Prediction

Prediction: Non-Sensitizer

Probability: 0.671

Enrichment: 0.978

Bayesian Score: -2.26

Mahalanobis Distance: 11.9

Mahalanobis Distance p-value: 1.09e-009

Prediction: Positive if the Bayesian score is above the estimated best cutoff value from minimizing the false positive and false negative rate.
Probability: The esimated probability that the sample is in the positive category. This assumes that the Bayesian score follows a normal distribution and is different from the prediction using a cutoff.
Enrichment: An estimate of enrichment, that is, the increased likelihood (versus random) of this sample being in the category.
Bayesian Score: The standard Laplacian-modified Bayesian score.
Mahalanobis Distance: The Mahalanobis distance (MD) is the distance to the center of the training data. The larger the MD, the less trustworthy the prediction.
Mahalanobis Distance p-value: The p-value gives the fraction of training data with an MD greater than or equal to the one for the given sample, assuming normally distributed data. The smaller the p-value, the less trustworthy the prediciton. For highly non-normal X properties (e.g., fingerprints), the MD p-value is wildly inaccurate.

Model Applicability

Unknown features are fingerprint features in the query molecule, but not found or appearing too infrequently in the training set.

1. All properties and OPS components are within expected ranges.

Feature Contribution

Top features for positive contribution

Fingerprint	Bit/Smiles	Feature Structure	Score	Sensitizer in training set
FCFP_12	907036844	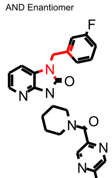 [*]N([*])C[c](:[*]):[*]	0.29	9 out of 9

FCFP_12	-1986158408	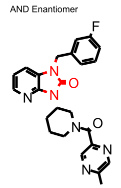<br>[*]N1[*]:[*]N([*])C1=<br>O	0.286	8 out of 8
FCFP_12	2106393770	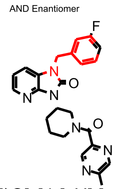<br>[*]N([*])C[c]1:[cH]:[<br>cH]:[*]:[cH]:[cH]:1	0.281	7 out of 7

Top Features for negative contribution

**Fingerprint**	**Bit/Smiles**	**Feature Structure**	**Score**	**Sensitizer in training set**
FCFP_12	-1474971978	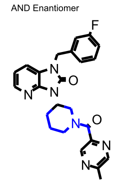<br>[*]C1[*]CN(CC1)C(=[*]<br>)[*]	-1.53	0 out of 5
FCFP_12	-1553874037	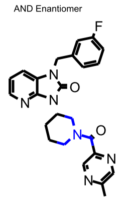<br>[*]CN(C[*])C(=[*])[*]	-0.802	3 out of 11
FCFP_12	-989213044	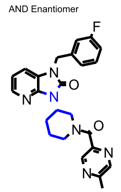<br>[*]N([*])[C@@H]1C[*]C<br>CC1	-0.663	1 out of 4

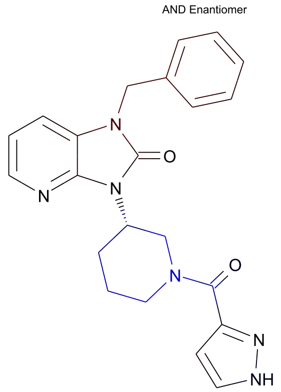

$C_{22}H_{22}N_6O_2$
Molecular Weight: 402.44907
ALogP: 2.501
Rotatable Bonds: 4
Acceptors: 4
Donors: 1

Structural Similar Compounds

Name	Mometasone furoate	Norbornanacetyloxy-4-benzene sulfonate	1-(4-Methoxy-5-benzofuranyl)-3-phenyl-1;3-propanediol
Structure	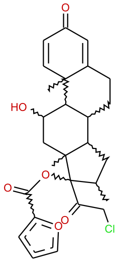	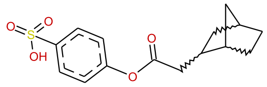	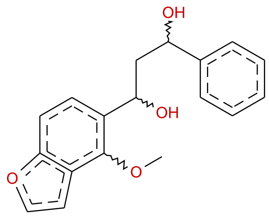
Actual Endpoint	Sensitizer	Sensitizer	Non-Sensitizer
Predicted Endpoint	Sensitizer	Sensitizer	Non-Sensitizer
Distance	0.712	0.725	0.731
Reference	Contact Dermatitis (1996) 34:161	SAR and QSAR in Env Res (1994) 2:159	SAR and QSAR in Env Res (1994) 2:159

Model Prediction

Prediction: Non-Sensitizer
Probability: 0.602
Enrichment: 0.877
Bayesian Score: -3.29
Mahalanobis Distance: 10.1
Mahalanobis Distance p-value: 1.24e-005

Prediction: Positive if the Bayesian score is above the estimated best cutoff value from minimizing the false positive and false negative rate.
Probability: The esimated probability that the sample is in the positive category. This assumes that the Bayesian score follows a normal distribution and is different from the prediction using a cutoff.
Enrichment: An estimate of enrichment, that is, the increased likelihood (versus random) of this sample being in the category.
Bayesian Score: The standard Laplacian-modified Bayesian score.
Mahalanobis Distance: The Mahalanobis distance (MD) is the distance to the center of the training data. The larger the MD, the less trustworthy the prediction.
Mahalanobis Distance p-value: The p-value gives the fraction of training data with an MD greater than or equal to the one for the given sample, assuming normally distributed data. The smaller the p-value, the less trustworthy the prediciton. For highly non-normal X properties (e.g., fingerprints), the MD p-value is wildly inaccurate.

Model Applicability

Unknown features are fingerprint features in the query molecule, but not found or appearing too infrequently in the training set.

1. All properties and OPS components are within expected ranges.
2. Unknown FCFP_2 feature: 1747267175: [*][c]1:[*]:[*]:[nH]:n:1
3. Unknown FCFP_2 feature: 262592487: [*]1:[*]:n:[nH]:[cH]:1
4. Unknown FCFP_2 feature: 1618184456: [*]1:[*]:[cH]:[cH]:[nH]:1

Feature Contribution

Top features for positive contribution

Fingerprint	Bit/Smiles	Feature Structure	Score	Sensitizer in training set
FCFP_12	907036844	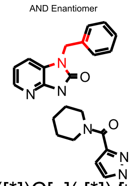 [*]N([*])C[c](:[*]):[*]	0.29	9 out of 9

FCFP_12	-1986158408	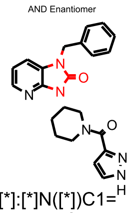 [*]N1[*]:[*]N([*])C1=O	0.286	8 out of 8
FCFP_12	2106393770	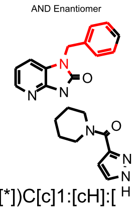 [*]N([*])C[c]1:[cH]:[cH]:[*]:[cH]:[cH]:1	0.281	7 out of 7
**Top Features for negative contribution**				
**Fingerprint**	**Bit/Smiles**	**Feature Structure**	**Score**	**Sensitizer in training set**
FCFP_12	-1474971978	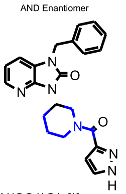 [*]C1[*]CN(CC1)C(=[*])[*]	-1.53	0 out of 5
FCFP_12	-1553874037	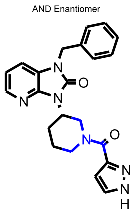 [*]CN(C[*])C(=[*])[*]	-0.802	3 out of 11
FCFP_12	-989213044	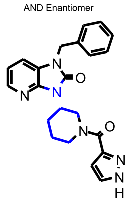 [*]N([*])[C@@H]1C[*]CC1	-0.663	1 out of 4

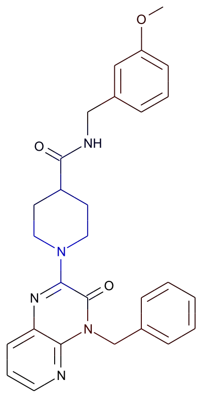

$C_{28}H_{29}N_5O_3$
Molecular Weight: 483.56156
ALogP: 2.942
Rotatable Bonds: 7
Acceptors: 6
Donors: 1

Model Prediction

Prediction: Non-Sensitizer
Probability: 0.706
Enrichment: 1.03
Bayesian Score: -1.69
Mahalanobis Distance: 10.1
Mahalanobis Distance p-value: 1.56e-005

Prediction: Positive if the Bayesian score is above the estimated best cutoff value from minimizing the false positive and false negative rate.
Probability: The esimated probability that the sample is in the positive category. This assumes that the Bayesian score follows a normal distribution and is different from the prediction using a cutoff.
Enrichment: An estimate of enrichment, that is, the increased likelihood (versus random) of this sample being in the category.
Bayesian Score: The standard Laplacian-modified Bayesian score.
Mahalanobis Distance: The Mahalanobis distance (MD) is the distance to the center of the training data. The larger the MD, the less trustworthy the prediction.
Mahalanobis Distance p-value: The p-value gives the fraction of training data with an MD greater than or equal to the one for the given sample, assuming normally distributed data. The smaller the p-value, the less trustworthy the prediciton. For highly non-normal X properties (e.g., fingerprints), the MD p-value is wildly inaccurate.

Structural Similar Compounds

Name	Mometasone furoate	Tixocortol pivalate	Budesonide
Structure	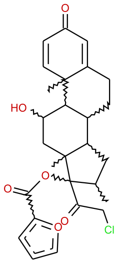	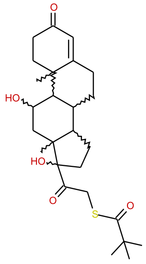	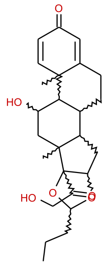
Actual Endpoint	Sensitizer	Sensitizer	Sensitizer
Predicted Endpoint	Sensitizer	Sensitizer	Sensitizer
Distance	0.639	0.696	0.727
Reference	Contact Dermatitis (1996) 34:161	Contact Dermatitis (1996) 34:161	Contact Dermatitis (1996) 34:161

Model Applicability

Unknown features are fingerprint features in the query molecule, but not found or appearing too infrequently in the training set.

1. All properties and OPS components are within expected ranges.

Feature Contribution

Top features for positive contribution

Fingerprint	Bit/Smiles	Feature Structure	Score	Sensitizer in training set
FCFP_12	907036844	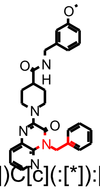 [*]N([*])C[c](:[*]):[*]	0.29	9 out of 9

FCFP_12	2106393770	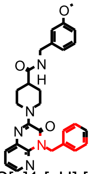 [*]N([*])C[c]1:[cH]:[cH]:[*]:[cH]:[cH]:1	0.281	7 out of 7
FCFP_12	1390842262	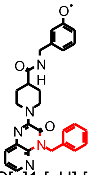 [*]N([*])C[c]1:[cH]:[cH]:[cH]:[cH]:[cH]:1	0.281	7 out of 7
**Top Features for negative contribution**				
**Fingerprint**	**Bit/Smiles**	**Feature Structure**	**Score**	**Sensitizer in training set**
FCFP_12	-1474971978	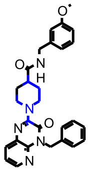 [*]C1[*]CN(CC1)C(=[*])[*]	-1.53	0 out of 5
FCFP_12	-1553874037	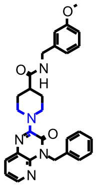 [*]CN(C[*])C(=[*])[*]	-0.802	3 out of 11
FCFP_12	1743817318	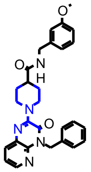 [*]\N=C(\N1CC[*]CC1)/C(=[*])[*]	-0.542	0 out of 1

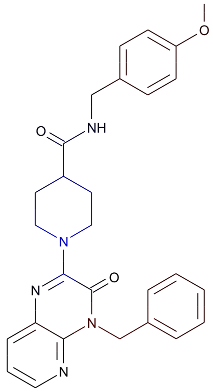

$C_{28}H_{29}N_5O_3$
Molecular Weight: 483.56156
ALogP: 2.942
Rotatable Bonds: 7
Acceptors: 6
Donors: 1

Model Prediction

Prediction: Non-Sensitizer
Probability: 0.685
Enrichment: 0.998
Bayesian Score: -2.04
Mahalanobis Distance: 10.1
Mahalanobis Distance p-value: 1.56e-005

Prediction: Positive if the Bayesian score is above the estimated best cutoff value from minimizing the false positive and false negative rate.
Probability: The esimated probability that the sample is in the positive category. This assumes that the Bayesian score follows a normal distribution and is different from the prediction using a cutoff.
Enrichment: An estimate of enrichment, that is, the increased likelihood (versus random) of this sample being in the category.
Bayesian Score: The standard Laplacian-modified Bayesian score.
Mahalanobis Distance: The Mahalanobis distance (MD) is the distance to the center of the training data. The larger the MD, the less trustworthy the prediction.
Mahalanobis Distance p-value: The p-value gives the fraction of training data with an MD greater than or equal to the one for the given sample, assuming normally distributed data. The smaller the p-value, the less trustworthy the prediciton. For highly non-normal X properties (e.g., fingerprints), the MD p-value is wildly inaccurate.

Structural Similar Compounds

Name	Mometasone furoate	Tixocortol pivalate	Budesonide
Structure	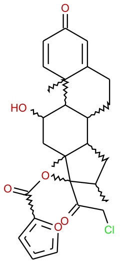	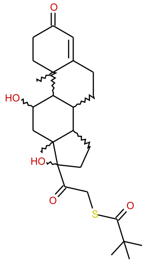	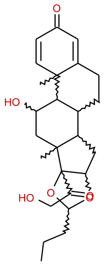
Actual Endpoint	Sensitizer	Sensitizer	Sensitizer
Predicted Endpoint	Sensitizer	Sensitizer	Sensitizer
Distance	0.639	0.696	0.727
Reference	Contact Dermatitis (1996) 34:161	Contact Dermatitis (1996) 34:161	Contact Dermatitis (1996) 34:161

Model Applicability

Unknown features are fingerprint features in the query molecule, but not found or appearing too infrequently in the training set.

1. All properties and OPS components are within expected ranges.

Feature Contribution

Top features for positive contribution

Fingerprint	Bit/Smiles	Feature Structure	Score	Sensitizer in training set
FCFP_12	907036844	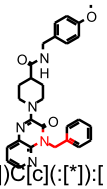 [*]N([*])C[c](:[*]):[*]	0.29	9 out of 9

FCFP_12	1390842262	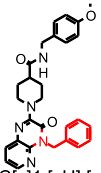 [*]N([*])C[c]1:[cH]:[cH]:[cH]:[cH]:[cH]:1	0.281	7 out of 7
FCFP_12	2106393770	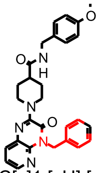 [*]N([*])C[c]1:[cH]:[cH]:[*]:[cH]:[cH]:1	0.281	7 out of 7

Top Features for negative contribution

Fingerprint	Bit/Smiles	Feature Structure	Score	Sensitizer in training set
FCFP_12	-1474971978	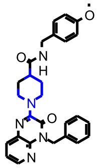 [*]C1[*]CN(CC1)C(=[*])[*]	-1.53	0 out of 5
FCFP_12	-1553874037	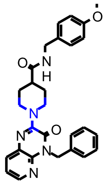 [*]CN(C[*])C(=[*])[*]	-0.802	3 out of 11
FCFP_12	1743817318	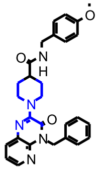 [*]\N=C(\N1CC[*]CC1)/C(=[*])[*]	-0.542	0 out of 1

Molecule

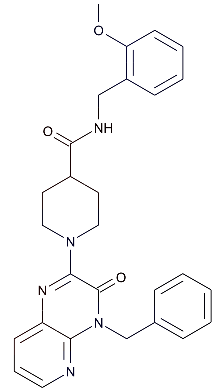

$C_{28}H_{29}N_5O_3$
Molecular Weight: 483.56156
ALogP: 2.942
Rotatable Bonds: 7
Acceptors: 6
Donors: 1

Model Prediction

Prediction: Non-Carcinogen
Probability: 0.437
Enrichment: 0.849
Bayesian Score: -2.67
Mahalanobis Distance: 11.5
Mahalanobis Distance p-value: 4.73e-008

Prediction: Positive if the Bayesian score is above the estimated best cutoff value from minimizing the false positive and false negative rate.
Probability: The esimated probability that the sample is in the positive category. This assumes that the Bayesian score follows a normal distribution and is different from the prediction using a cutoff.
Enrichment: An estimate of enrichment, that is, the increased likelihood (versus random) of this sample being in the category.
Bayesian Score: The standard Laplacian-modified Bayesian score.
Mahalanobis Distance: The Mahalanobis distance (MD) is the distance to the center of the training data. The larger the MD, the less trustworthy the prediction.
Mahalanobis Distance p-value: The p-value gives the fraction of training data with an MD greater than or equal to the one for the given sample, assuming normally distributed data. The smaller the p-value, the less trustworthy the prediciton. For highly non-normal X properties (e.g., fingerprints), the MD p-value is wildly inaccurate.

TOPKAT_Weight_of_Evidence_Rodent_Carcinogenicity

Structural Similar Compounds

Name	Moricizine	Diltiazem	Felodipine
Structure	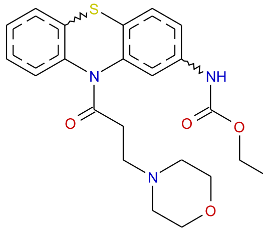	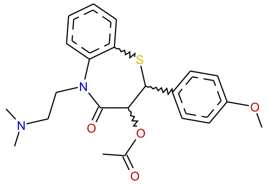	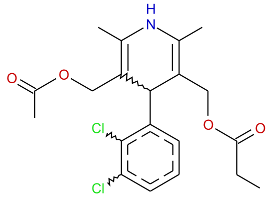
Actual Endpoint	Carcinogen	Carcinogen	Non-Carcinogen
Predicted Endpoint	Carcinogen	Carcinogen	Non-Carcinogen
Distance	0.595	0.632	0.644
Reference	US FDA (Centre for Drug Eval.& Res./Off. Testing & Res.) Sept. 1997	US FDA (Centre for Drug Eval.& Res./Off. Testing & Res.) Sept. 1997	US FDA (Centre for Drug Eval.& Res./Off. Testing & Res.) Sept. 1997

Model Applicability

Unknown features are fingerprint features in the query molecule, but not found or appearing too infrequently in the training set.

1. All properties and OPS components are within expected ranges.

Feature Contribution

Top features for positive contribution

Fingerprint	Bit/Smiles	Feature Structure	Score	Carcinogen in training set
SCFP_8	-1377141613	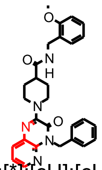 [*][c]1:[*]:[cH]:[cH]:[cH]:[c]:1N=[*]	0.444	8 out of 10

SCFP_8	1851000357	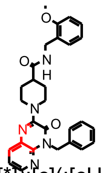 [*][c](:[*]):[c](:[cH]:[*])N=[*]	0.365	8 out of 11
SCFP_8	38222003	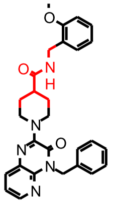 [*]CNC(=O)C(C[*])C[*]	0.303	1 out of 1

Top Features for negative contribution

Fingerprint	Bit/Smiles	Feature Structure	Score	Carcinogen in training set
SCFP_8	-587479743	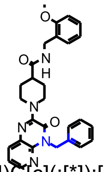 [*]N([*])C[c](:[*]):[*]	-0.39	0 out of 1
SCFP_8	-1325723550	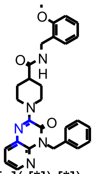 [*]C(=N[c](:[*]):[*]) [*]	-0.37	2 out of 7
SCFP_8	5	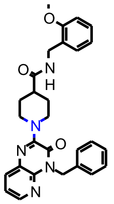 [*]N([*])[*]	-0.247	52 out of 140

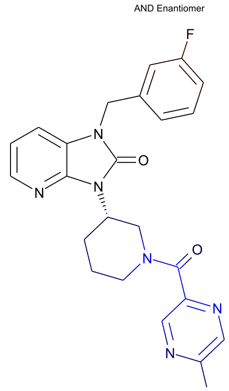

$C_{24}H_{23}FN_6O_2$
Molecular Weight: 446.47682
ALogP: 2.244
Rotatable Bonds: 4
Acceptors: 5
Donors: 0

Structural Similar Compounds

Name	Risperidone	Buspirone	Levocabstine
Structure	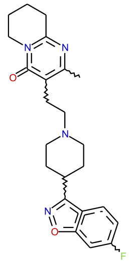	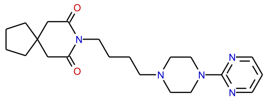	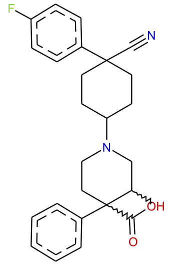
Actual Endpoint	Carcinogen	Non-Carcinogen	Carcinogen
Predicted Endpoint	Carcinogen	Non-Carcinogen	Carcinogen
Distance	0.605	0.638	0.639
Reference	US FDA (Centre for Drug Eval.& Res./Off. Testing & Res.) Sept. 1997	US FDA (Centre for Drug Eval.& Res./Off. Testing & Res.) Sept. 1997	US FDA (Centre for Drug Eval.& Res./Off. Testing & Res.) Sept. 1997

Model Prediction

Prediction: Non-Carcinogen
Probability: 0.294
Enrichment: 0.571
Bayesian Score: -7.6
Mahalanobis Distance: 6.94
Mahalanobis Distance p-value: 0.507

Prediction: Positive if the Bayesian score is above the estimated best cutoff value from minimizing the false positive and false negative rate.
Probability: The esimated probability that the sample is in the positive category. This assumes that the Bayesian score follows a normal distribution and is different from the prediction using a cutoff.
Enrichment: An estimate of enrichment, that is, the increased likelihood (versus random) of this sample being in the category.
Bayesian Score: The standard Laplacian-modified Bayesian score.
Mahalanobis Distance: The Mahalanobis distance (MD) is the distance to the center of the training data. The larger the MD, the less trustworthy the prediction.
Mahalanobis Distance p-value: The p-value gives the fraction of training data with an MD greater than or equal to the one for the given sample, assuming normally distributed data. The smaller the p-value, the less trustworthy the prediciton. For highly non-normal X properties (e.g., fingerprints), the MD p-value is wildly inaccurate.

Model Applicability

Unknown features are fingerprint features in the query molecule, but not found or appearing too infrequently in the training set.

1. All properties and OPS components are within expected ranges.

Feature Contribution

Top features for positive contribution

Fingerprint	Bit/Smiles	Feature Structure	Score	Carcinogen in training set
SCFP_8	-971326317	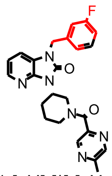 AND Enantiomer [*]C[c]1:[cH]:[*]:[cH]:[c](F):[cH]:1	0.303	1 out of 1

SCFP_8	136686699	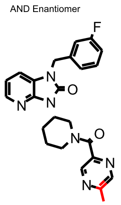 [*]:[c](:[*])C	0.173	28 out of 49
SCFP_8	1194442465	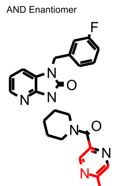 [*][c]1:[*]:[cH]:[c](C):n:[cH]:1	0.166	3 out of 5

Top Features for negative contribution

Fingerprint	Bit/Smiles	Feature Structure	Score	Carcinogen in training set
SCFP_8	-827073191	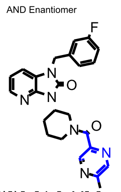 [*]C(=[*])[c]1:[cH]:[*]:[c]([*]):[cH]:n:1	-0.889	0 out of 3
SCFP_8	306578635	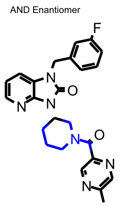 [*]C(=[*])N1C[*]CCC1	-0.889	0 out of 3
SCFP_8	240509252	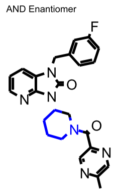 [*][C@@H]1[*]N([*])CCC1	-0.879	1 out of 8

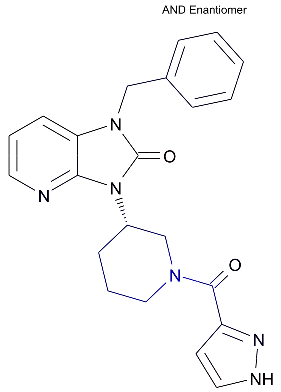

$C_{22}H_{22}N_6O_2$
Molecular Weight: 402.44907
ALogP: 2.501
Rotatable Bonds: 4
Acceptors: 4
Donors: 1

Model Prediction

Prediction: Non-Carcinogen
Probability: 0.347
Enrichment: 0.674
Bayesian Score: -5.49
Mahalanobis Distance: 6.44
Mahalanobis Distance p-value: 0.759

Prediction: Positive if the Bayesian score is above the estimated best cutoff value from minimizing the false positive and false negative rate.
Probability: The esimated probability that the sample is in the positive category. This assumes that the Bayesian score follows a normal distribution and is different from the prediction using a cutoff.
Enrichment: An estimate of enrichment, that is, the increased likelihood (versus random) of this sample being in the category.
Bayesian Score: The standard Laplacian-modified Bayesian score.
Mahalanobis Distance: The Mahalanobis distance (MD) is the distance to the center of the training data. The larger the MD, the less trustworthy the prediction.
Mahalanobis Distance p-value: The p-value gives the fraction of training data with an MD greater than or equal to the one for the given sample, assuming normally distributed data. The smaller the p-value, the less trustworthy the prediciton. For highly non-normal X properties (e.g., fingerprints), the MD p-value is wildly inaccurate.

Structural Similar Compounds

Name	Levocabstine	Lansoprazole	Omeprazole
Structure	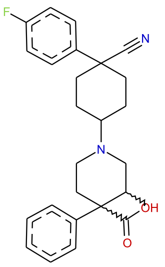	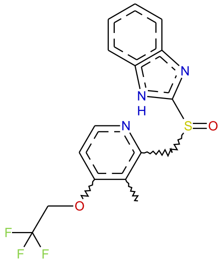	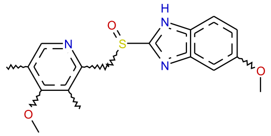
Actual Endpoint	Carcinogen	Carcinogen	Carcinogen
Predicted Endpoint	Carcinogen	Carcinogen	Carcinogen
Distance	0.554	0.627	0.634
Reference	US FDA (Centre for Drug Eval.& Res./Off. Testing & Res.) Sept. 1997	US FDA (Centre for Drug Eval.& Res./Off. Testing & Res.) Sept. 1997	US FDA (Centre for Drug Eval.& Res./Off. Testing & Res.) Sept. 1997

Model Applicability

Unknown features are fingerprint features in the query molecule, but not found or appearing too infrequently in the training set.

1. All properties and OPS components are within expected ranges.

Feature Contribution

Top features for positive contribution				
**Fingerprint**	**Bit/Smiles**	**Feature Structure**	**Score**	**Carcinogen in training set**
SCFP_8	-378091462	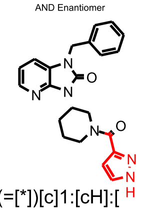 [*]C(=[*])[c]1:[cH]:[cH]:[nH]:n:1	0.303	1 out of 1

SCFP_8	-1946918893	AND Enantiomer<br>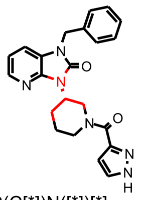<br>[*]CC(C[*])N([*])[*]	0.158	8 out of 14
SCFP_8	-1375926917	AND Enantiomer<br>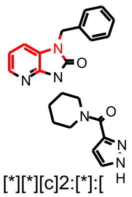<br>[*]N1[*][*][c]2:[*]:[<br>cH]:[cH]:[cH]:[c]1:2	0.136	16 out of 29

Top Features for negative contribution

**Fingerprint**	**Bit/Smiles**	**Feature Structure**	**Score**	**Carcinogen in training set**
SCFP_8	306578635	AND Enantiomer<br>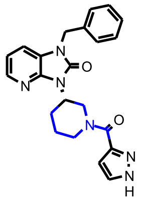<br>[*]C(=[*])N1C[*]CCC1	-0.889	0 out of 3
SCFP_8	240509252	AND Enantiomer<br>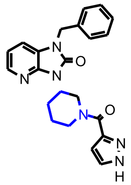<br>[*][C@@H]1[*]N([*])CC<br>C1	-0.879	1 out of 8
SCFP_8	-1343150366	AND Enantiomer<br>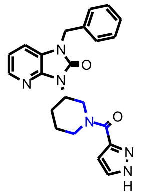<br>[*]CN(C[*])C(=[*])[*]	-0.77	3 out of 16

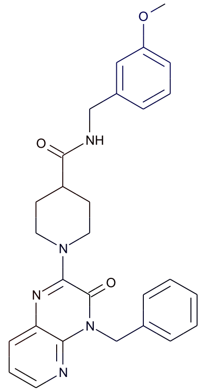

$C_{28}H_{29}N_5O_3$
Molecular Weight: 483.56156
ALogP: 2.942
Rotatable Bonds: 7
Acceptors: 6
Donors: 1

Model Prediction

Prediction: Non-Carcinogen
Probability: 0.402
Enrichment: 0.781
Bayesian Score: -3.7
Mahalanobis Distance: 11.5
Mahalanobis Distance p-value: 4.73e-008

Prediction: Positive if the Bayesian score is above the estimated best cutoff value from minimizing the false positive and false negative rate.
Probability: The esimated probability that the sample is in the positive category. This assumes that the Bayesian score follows a normal distribution and is different from the prediction using a cutoff.
Enrichment: An estimate of enrichment, that is, the increased likelihood (versus random) of this sample being in the category.
Bayesian Score: The standard Laplacian-modified Bayesian score.
Mahalanobis Distance: The Mahalanobis distance (MD) is the distance to the center of the training data. The larger the MD, the less trustworthy the prediction.
Mahalanobis Distance p-value: The p-value gives the fraction of training data with an MD greater than or equal to the one for the given sample, assuming normally distributed data. The smaller the p-value, the less trustworthy the prediciton. For highly non-normal X properties (e.g., fingerprints), the MD p-value is wildly inaccurate.

Structural Similar Compounds

Name	Moricizine	Diltiazem	Felodipine
Structure	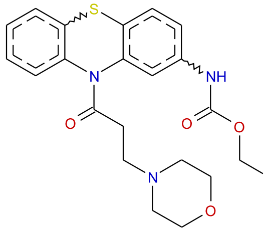	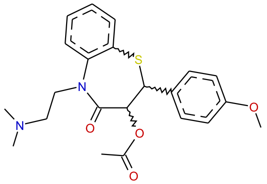	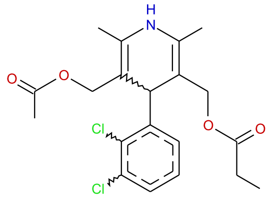
Actual Endpoint	Carcinogen	Carcinogen	Non-Carcinogen
Predicted Endpoint	Carcinogen	Carcinogen	Non-Carcinogen
Distance	0.595	0.629	0.644
Reference	US FDA (Centre for Drug Eval.& Res./Off. Testing & Res.) Sept. 1997	US FDA (Centre for Drug Eval.& Res./Off. Testing & Res.) Sept. 1997	US FDA (Centre for Drug Eval.& Res./Off. Testing & Res.) Sept. 1997

Model Applicability

Unknown features are fingerprint features in the query molecule, but not found or appearing too infrequently in the training set.

1. All properties and OPS components are within expected ranges.

Feature Contribution

Top features for positive contribution

Fingerprint	Bit/Smiles	Feature Structure	Score	Carcinogen in training set
SCFP_8	-1377141613	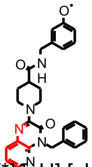 [*][c]1:[*]:[cH]:[cH]:[cH]:[c]:1N=[*]	0.444	8 out of 10

SCFP_8	1851000357	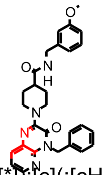 [*][c](:[*]):[c](:[cH]:[*])N=[*]	0.365	8 out of 11
SCFP_8	38222003	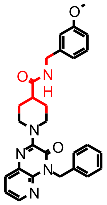 [*]CNC(=O)C(C[*])C[*]	0.303	1 out of 1

Top Features for negative contribution

Fingerprint	Bit/Smiles	Feature Structure	Score	Carcinogen in training set
SCFP_8	-1632615624	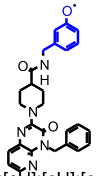 [*]C[c]1:[cH]:[cH]:[c H]:[c](O[*]):[cH]:1	-0.659	1 out of 6
SCFP_8	-587479743	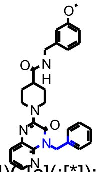 [*]N([*])C[c](:[*]):[*]	-0.39	0 out of 1
SCFP_8	1435948812	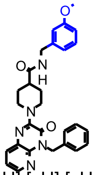 [*][c]1:[cH]:[cH]:[cH]:[c](OC):[cH]:1	-0.375	1 out of 4

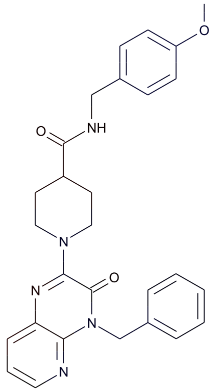

$C_{28}H_{29}N_5O_3$
Molecular Weight: 483.56156
ALogP: 2.942
Rotatable Bonds: 7
Acceptors: 6
Donors: 1

Model Prediction

Prediction: Non-Carcinogen
Probability: 0.426
Enrichment: 0.828
Bayesian Score: -2.98
Mahalanobis Distance: 11.5
Mahalanobis Distance p-value: 4.73e-008

Prediction: Positive if the Bayesian score is above the estimated best cutoff value from minimizing the false positive and false negative rate.
Probability: The esimated probability that the sample is in the positive category. This assumes that the Bayesian score follows a normal distribution and is different from the prediction using a cutoff.
Enrichment: An estimate of enrichment, that is, the increased likelihood (versus random) of this sample being in the category.
Bayesian Score: The standard Laplacian-modified Bayesian score.
Mahalanobis Distance: The Mahalanobis distance (MD) is the distance to the center of the training data. The larger the MD, the less trustworthy the prediction.
Mahalanobis Distance p-value: The p-value gives the fraction of training data with an MD greater than or equal to the one for the given sample, assuming normally distributed data. The smaller the p-value, the less trustworthy the prediciton. For highly non-normal X properties (e.g., fingerprints), the MD p-value is wildly inaccurate.

Structural Similar Compounds

Name	Moricizine	Diltiazem	Felodipine
Structure	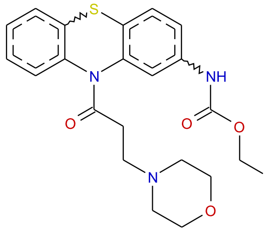	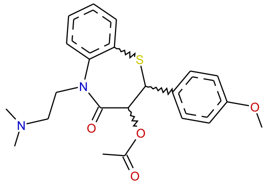	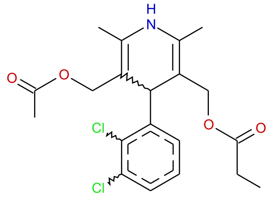
Actual Endpoint	Carcinogen	Carcinogen	Non-Carcinogen
Predicted Endpoint	Carcinogen	Carcinogen	Non-Carcinogen
Distance	0.595	0.621	0.644
Reference	US FDA (Centre for Drug Eval.& Res./Off. Testing & Res.) Sept. 1997	US FDA (Centre for Drug Eval.& Res./Off. Testing & Res.) Sept. 1997	US FDA (Centre for Drug Eval.& Res./Off. Testing & Res.) Sept. 1997

Model Applicability

Unknown features are fingerprint features in the query molecule, but not found or appearing too infrequently in the training set.

1. All properties and OPS components are within expected ranges.

Feature Contribution

Top features for positive contribution

Fingerprint	Bit/Smiles	Feature Structure	Score	Carcinogen in training set
SCFP_8	-1377141613	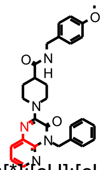 [*][c]1:[*]:[cH]:[cH]:[cH]:[c]:1N=[*]	0.444	8 out of 10

SCFP_8	1851000357	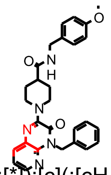 [*][c](:[*]):[c](:[cH]:[*])N=[*]	0.365	8 out of 11
SCFP_8	38222003	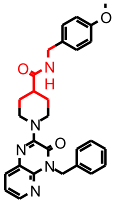 [*]CNC(=O)C(C[*])C[*]	0.303	1 out of 1
**Top Features for negative contribution**				
**Fingerprint**	**Bit/Smiles**	**Feature Structure**	**Score**	**Carcinogen in training set**
SCFP_8	-587479743	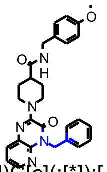 [*]N([*])C[c](:[*]):[*]	-0.39	0 out of 1
SCFP_8	-1325723550	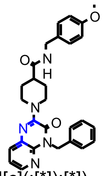 [*]C(=N[c](:[*]):[*])[*]	-0.37	2 out of 7
SCFP_8	5	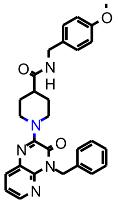 [*]N([*])[*]	-0.247	52 out of 140

Molecule

TOPKAT_Carcinogenic_Potency_TD50_Mouse

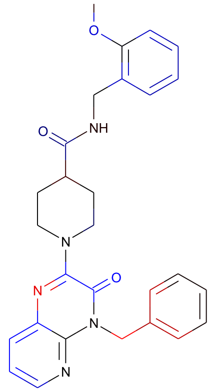

$C_{28}H_{29}N_5O_3$

Molecular Weight: 483.56156

ALogP: 2.942

Rotatable Bonds: 7

Acceptors: 6

Donors: 1

Model Prediction

Prediction: 16.3

Unit: mg/kg_body_weight/day

Mahalanobis Distance: 12.5

Mahalanobis Distance p-value: 2.22e-006

Mahalanobis Distance: The Mahalanobis distance (MD) is a generalization of the Euclidean distance that accounts for correlations among the X properties. It is calculated as the distance to the center of the training data. The larger the MD, the less trustworthy the prediction.
Mahalanobis Distance p-value: The p-value gives the fraction of training data with an MD greater than or equal to the one for the given sample, assuming normally distributed data. The smaller the p-value, the less trustworthy the prediciton. For highly non-normal X properties (e.g., fingerprints), the MD p-value is wildly inaccurate.

Structural Similar Compounds

Name	Sterigmatocystin s	223	Phenolphthalein
Structure	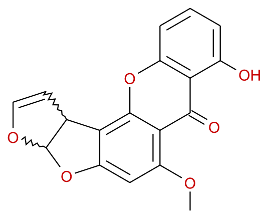	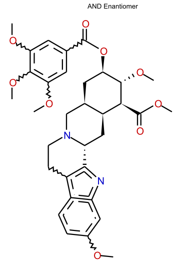	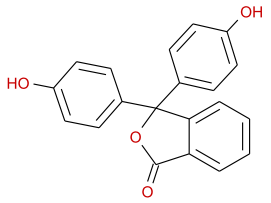
Actual Endpoint (-log C)	5.55284	5.08368	2.43468
Predicted Endpoint (-log C)	3.6442	5.08273	3.66084
Distance	0.836	0.837	0.860
Reference	CPDB	CPDB	CPDB

Model Applicability

Unknown features are fingerprint features in the query molecule, but not found or appearing too infrequently in the training set.

1. OPS PC7 out of range. Value: 5.6421. Training min, max, SD, explained variance: -5.1479, 5.5527, 1.707, 0.0363.
2. Unknown ECFP_2 feature: -661097313: [*]CN(C(=[*])[*])[c](:[*]):[*]
3. Unknown ECFP_2 feature: 671679640: [*]\N=C(\N([*])[*])/C(=[*])[*]
4. Unknown ECFP_2 feature: 1951894094: [*]CN(C[*])C(=[*])[*]
5. Unknown ECFP_2 feature: -597295171: [*][c](:[*]):[c](:[cH]:[*])N=[*]
6. Unknown ECFP_2 feature: -44121127: [*]N([*])C[c](:[*]):[*]
7. Unknown ECFP_2 feature: -857146788: [*]CC(C[*])C(=[*])[*]

Feature Contribution

Top features for positive contribution			
Fingerprint	Bit/Smiles	Feature Structure	Score

ECFP_6	655739385	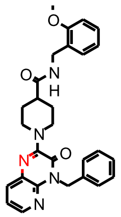 [*]N=[*]	0.229
ECFP_6	1559650422	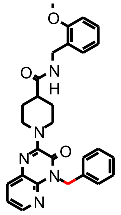 [*]C[*]	0.203
ECFP_6	-2024255407	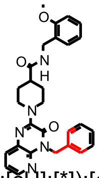 [*]C[c](:[cH]:[*]):[cH]:[*]	0.172
**Top Features for negative contribution**			
**Fingerprint**	**Bit/Smiles**	**Feature Structure**	**Score**
ECFP_6	2106656448	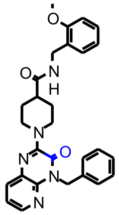 [*]C(=O)[*]	-0.275
ECFP_6	1996767644	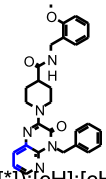 [*][c](:[*]):[cH]:[cH]:[*]	-0.251

ECFP_6	642810091	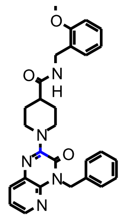 [*]C(=[*])[*]	-0.247

#UNDEFINED

TOPKAT_Carcinogenic_Potency_TD50_Mouse

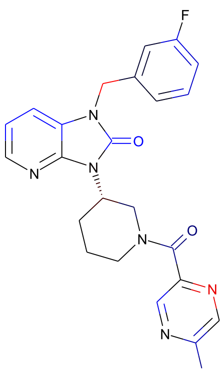

$C_{24}H_{23}FN_6O_2$

Molecular Weight: 446.47682

ALogP: 2.244

Rotatable Bonds: 4

Acceptors: 5

Donors: 0

Model Prediction

Prediction: 23.6

Unit: mg/kg_body_weight/day

Mahalanobis Distance: 14

Mahalanobis Distance p-value: 3.21e-010

Mahalanobis Distance: The Mahalanobis distance (MD) is a generalization of the Euclidean distance that accounts for correlations among the X properties. It is calculated as the distance to the center of the training data. The larger the MD, the less trustworthy the prediction.

Mahalanobis Distance p-value: The p-value gives the fraction of training data with an MD greater than or equal to the one for the given sample, assuming normally distributed data. The smaller the p-value, the less trustworthy the prediciton. For highly non-normal X properties (e.g., fingerprints), the MD p-value is wildly inaccurate.

Structural Similar Compounds

Name	Sterigmatocystin s	Phenolphthalein	C.I. pigment red 3
Structure	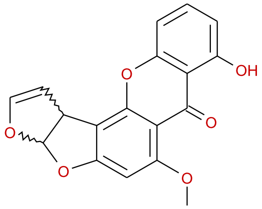	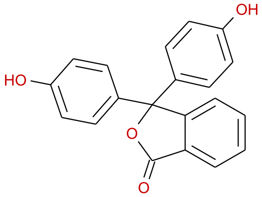	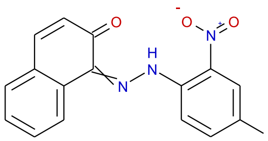
Actual Endpoint (-log C)	5.55284	2.43468	0.937339
Predicted Endpoint (-log C)	3.6442	3.66084	3.17837
Distance	0.761	0.834	0.847
Reference	CPDB	CPDB	CPDB

Model Applicability

Unknown features are fingerprint features in the query molecule, but not found or appearing too infrequently in the training set.

1. All properties and OPS components are within expected ranges.
2. Unknown ECFP_2 feature: -957084426: [*]C([*])N1C(=[*])[*][*]:[c]1:[*]
3. Unknown ECFP_2 feature: 1135573248: [*]N1[*]:[*]N([*])C1=O
4. Unknown ECFP_2 feature: -661097313: [*]CN(C(=[*])[*])[c](:[*]):[*]
5. Unknown ECFP_2 feature: -1236953626: [*]N1[*][*][c](:[*]):[c]1:[cH]:[*]
6. Unknown ECFP_2 feature: -1102925512: [*]CN(C[*])C(=[*])[*]
7. Unknown ECFP_2 feature: 2077298510: [*]N([*])C(=O)[c](:[*]):[*]
8. Unknown ECFP_2 feature: -1869628272: [*]CC(C[*])N([*])[*]
9. Unknown ECFP_2 feature: 1413420509: [*]C(=[*])[c](:[cH]:[*]):n:[*]
10. Unknown ECFP_2 feature: -44121127: [*]N([*])C[c](:[*]):[*]

Feature Contribution

Top features for positive contribution			
**Fingerprint**	**Bit/Smiles**	**Feature Structure**	**Score**

ECFP_6	655739385	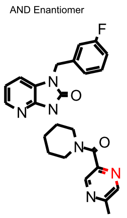 [*]N=[*]	0.229
ECFP_6	1559650422	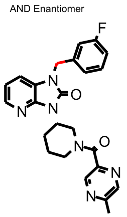 [*]C[*]	0.203
ECFP_6	-2024255407	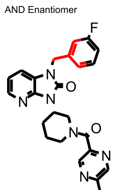 [*]C[c](:[cH]:[*]):[cH]:[*]	0.172

Top Features for negative contribution

Fingerprint	Bit/Smiles	Feature Structure	Score
ECFP_6	2106656448	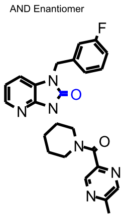 [*]C(=O)[*]	-0.275
ECFP_6	1996767644	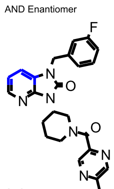 [*][c](:[*]):[cH]:[cH]:[*]	-0.251

ECFP_6	642810091	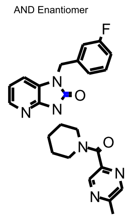 [*]C(=[*])[*]	-0.247

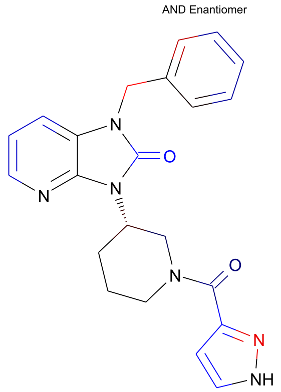

$C_{22}H_{22}N_6O_2$
Molecular Weight: 402.44907
ALogP: 2.501
Rotatable Bonds: 4
Acceptors: 4
Donors: 1

Model Prediction

Prediction: 11.7
Unit: mg/kg_body_weight/day
Mahalanobis Distance: 11.9
Mahalanobis Distance p-value: 3.08e-005

Mahalanobis Distance: The Mahalanobis distance (MD) is a generalization of the Euclidean distance that accounts for correlations among the X properties. It is calculated as the distance to the center of the training data. The larger the MD, the less trustworthy the prediction.
Mahalanobis Distance p-value: The p-value gives the fraction of training data with an MD greater than or equal to the one for the given sample, assuming normally distributed data. The smaller the p-value, the less trustworthy the prediciton. For highly non-normal X properties (e.g., fingerprints), the MD p-value is wildly inaccurate.

Structural Similar Compounds

Name	Phenolphthalein	Sterigmatocystin s	Ripazepam
Structure	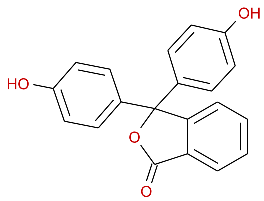	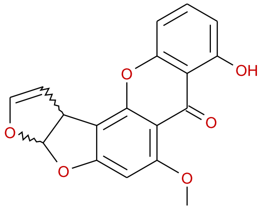	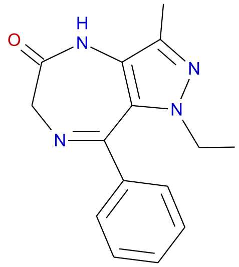
Actual Endpoint (-log C)	2.43468	5.55284	3.37174
Predicted Endpoint (-log C)	3.66084	3.6442	3.42826
Distance	0.719	0.746	0.804
Reference	CPDB	CPDB	CPDB

Model Applicability

Unknown features are fingerprint features in the query molecule, but not found or appearing too infrequently in the training set.

1. All properties and OPS components are within expected ranges.
2. Unknown ECFP_2 feature: -957084426: [*]C([*])N1C(=[*])[*][*]:[c]1:[*]
3. Unknown ECFP_2 feature: 1135573248: [*]N1[*]:[*]N([*])C1=O
4. Unknown ECFP_2 feature: -661097313: [*]CN(C(=[*])[*])[c](:[*]):[*]
5. Unknown ECFP_2 feature: -1236953626: [*]N1[*][*][c](:[*]):[c]1:[cH]:[*]
6. Unknown ECFP_2 feature: -1102925512: [*]CN(C[*])C(=[*])[*]
7. Unknown ECFP_2 feature: 2077298510: [*]N([*])C(=O)[c](:[*]):[*]
8. Unknown ECFP_2 feature: 1413420509: [*]C(=[*])[c](:[cH]:[*]):n:[*]
9. Unknown ECFP_2 feature: -1869628272: [*]CC(C[*])N([*])[*]
10. Unknown ECFP_2 feature: -44121127: [*]N([*])C[c](:[*]):[*]

Feature Contribution

Top features for positive contribution			
Fingerprint	Bit/Smiles	Feature Structure	Score

ECFP_6	655739385	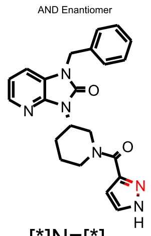 [*]N=[*]	0.229
ECFP_6	1559650422	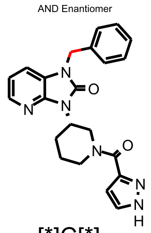 [*]C[*]	0.203
ECFP_6	-2024255407	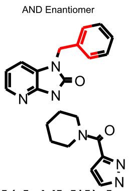 [*]C[c](:[cH]:[*]):[cH]:[*]	0.172

Top Features for negative contribution

**Fingerprint**	**Bit/Smiles**	**Feature Structure**	**Score**
ECFP_6	2106656448	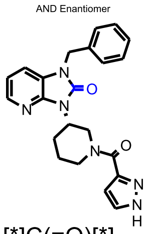 [*]C(=O)[*]	-0.275
ECFP_6	1996767644	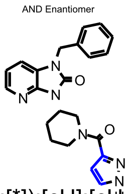 [*][c](:[*]):[cH]:[cH]:[*]	-0.251

ECFP_6	642810091	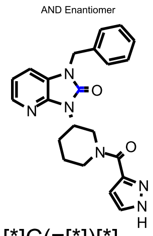 [*]C(=[*])[*]	-0.247

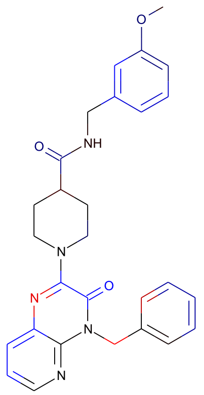

$C_{28}H_{29}N_5O_3$
Molecular Weight: 483.56156
ALogP: 2.942
Rotatable Bonds: 7
Acceptors: 6
Donors: 1

Model Prediction

Prediction: 21.1
Unit: mg/kg_body_weight/day
Mahalanobis Distance: 14
Mahalanobis Distance p-value: 3.34e-010

Mahalanobis Distance: The Mahalanobis distance (MD) is a generalization of the Euclidean distance that accounts for correlations among the X properties. It is calculated as the distance to the center of the training data. The larger the MD, the less trustworthy the prediction.
Mahalanobis Distance p-value: The p-value gives the fraction of training data with an MD greater than or equal to the one for the given sample, assuming normally distributed data. The smaller the p-value, the less trustworthy the prediciton. For highly non-normal X properties (e.g., fingerprints), the MD p-value is wildly inaccurate.

Structural Similar Compounds

Name	223	Sterigmatocystin s	Phenolphthalein
Structure	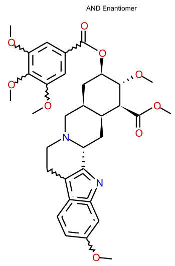	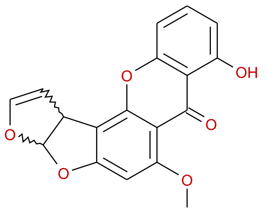	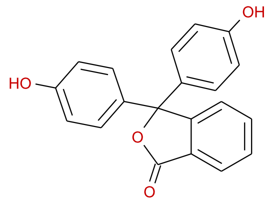
Actual Endpoint (-log C)	5.08368	5.55284	2.43468
Predicted Endpoint (-log C)	5.08273	3.6442	3.66084
Distance	0.831	0.836	0.860
Reference	CPDB	CPDB	CPDB

Model Applicability

Unknown features are fingerprint features in the query molecule, but not found or appearing too infrequently in the training set.

1. All properties and OPS components are within expected ranges.
2. Unknown ECFP_2 feature: -661097313: [*]CN(C(=[*])[*])[c](:[*]):[*]
3. Unknown ECFP_2 feature: 671679640: [*]\N=C(\N([*])[*])/C(=[*])[*]
4. Unknown ECFP_2 feature: 1951894094: [*]CN(C[*])C(=[*])[*]
5. Unknown ECFP_2 feature: -597295171: [*][c](:[*]):[c](:[cH]:[*])N=[*]
6. Unknown ECFP_2 feature: -44121127: [*]N([*])C[c](:[*]):[*]
7. Unknown ECFP_2 feature: -857146788: [*]CC(C[*])C(=[*])[*]

Feature Contribution

Top features for positive contribution

Fingerprint	Bit/Smiles	Feature Structure	Score

ECFP_6	655739385	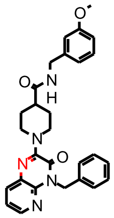 [*]N=[*]	0.229
ECFP_6	1559650422	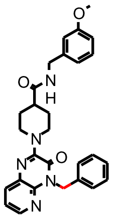 [*]C[*]	0.203
ECFP_6	-2024255407	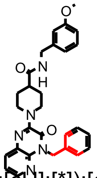 [*]C[c](:[cH]:[*]):[cH]:[*]	0.172

Top Features for negative contribution

Fingerprint	Bit/Smiles	Feature Structure	Score
ECFP_6	2106656448	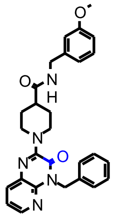 [*]C(=O)[*]	-0.275
ECFP_6	1996767644	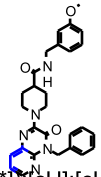 [*][c](:[*]):[cH]:[cH]:[*]	-0.251

ECFP_6	642810091	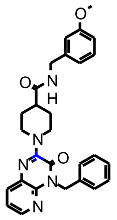 [*]C(=[*])[*]	-0.247

#UNDEFINED

TOPKAT_Carcinogenic_Potency_TD50_Mouse

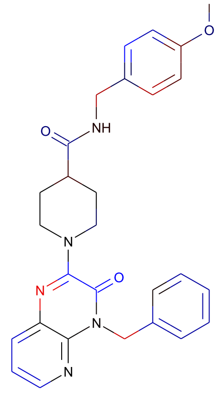

$C_{28}H_{29}N_5O_3$
Molecular Weight: 483.56156
ALogP: 2.942
Rotatable Bonds: 7
Acceptors: 6
Donors: 1

Model Prediction

Prediction: 12.8
Unit: mg/kg_body_weight/day
Mahalanobis Distance: 13.7
Mahalanobis Distance p-value: 1.8e-009

Mahalanobis Distance: The Mahalanobis distance (MD) is a generalization of the Euclidean distance that accounts for correlations among the X properties. It is calculated as the distance to the center of the training data. The larger the MD, the less trustworthy the prediction.
Mahalanobis Distance p-value: The p-value gives the fraction of training data with an MD greater than or equal to the one for the given sample, assuming normally distributed data. The smaller the p-value, the less trustworthy the prediciton. For highly non-normal X properties (e.g., fingerprints), the MD p-value is wildly inaccurate.

Structural Similar Compounds

Name	223	Sterigmatocystin s	Phenolphthalein
Structure	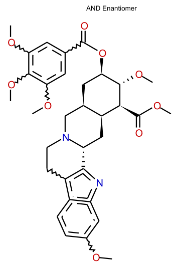	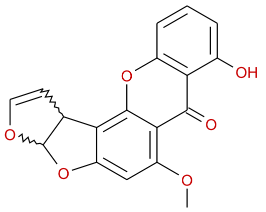	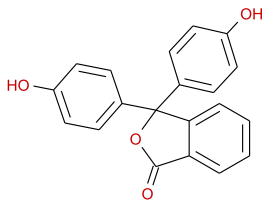
Actual Endpoint (-log C)	5.08368	5.55284	2.43468
Predicted Endpoint (-log C)	5.08273	3.6442	3.66084
Distance	0.833	0.843	0.859
Reference	CPDB	CPDB	CPDB

Model Applicability

Unknown features are fingerprint features in the query molecule, but not found or appearing too infrequently in the training set.

1. All properties and OPS components are within expected ranges.
2. Unknown ECFP_2 feature: -661097313: [*]CN(C(=[*])[*])[c](:[*]):[*]
3. Unknown ECFP_2 feature: 671679640: [*]\N=C(\N([*])[*])/C(=[*])[*]
4. Unknown ECFP_2 feature: 1951894094: [*]CN(C[*])C(=[*])[*]
5. Unknown ECFP_2 feature: -597295171: [*][c](:[*]):[c](:[cH]:[*])N=[*]
6. Unknown ECFP_2 feature: -44121127: [*]N([*])C[c](:[*]):[*]
7. Unknown ECFP_2 feature: -857146788: [*]CC(C[*])C(=[*])[*]

Feature Contribution

Top features for positive contribution			
Fingerprint	Bit/Smiles	Feature Structure	Score

ECFP_6	655739385	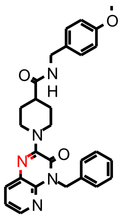 [*]N=[*]	0.229
ECFP_6	1559650422	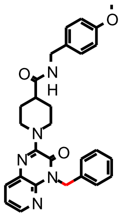 [*]C[*]	0.203
ECFP_6	-2024255407	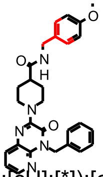 [*]C[c](:[cH]:[*]):[cH]:[*]	0.172

Top Features for negative contribution

Fingerprint	Bit/Smiles	Feature Structure	Score
ECFP_6	2106656448	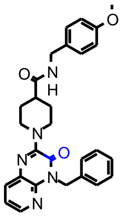 [*]C(=O)[*]	-0.275
ECFP_6	1996767644	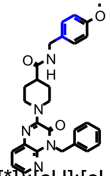 [*][c](:[*]):[cH]:[cH]:[*]	-0.251

ECFP_6	642810091	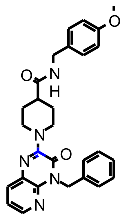 [*]C(=[*])[*]	-0.247

Molecule

TOPKAT_Carcinogenic_Potency_TD50_Rat

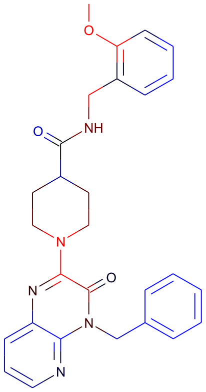

$C_{28}H_{29}N_5O_3$

Molecular Weight: 483.56156

ALogP: 2.942

Rotatable Bonds: 7

Acceptors: 6

Donors: 1

Model Prediction

Prediction: 1.26

Unit: mg/kg_body_weight/day

Mahalanobis Distance: 18

Mahalanobis Distance p-value: 3.08e-021

Mahalanobis Distance: The Mahalanobis distance (MD) is a generalization of the Euclidean distance that accounts for correlations among the X properties. It is calculated as the distance to the center of the training data. The larger the MD, the less trustworthy the prediction.
Mahalanobis Distance p-value: The p-value gives the fraction of training data with an MD greater than or equal to the one for the given sample, assuming normally distributed data. The smaller the p-value, the less trustworthy the prediciton. For highly non-normal X properties (e.g., fingerprints), the MD p-value is wildly inaccurate.

Structural Similar Compounds

Name	5,6-Dimethoxysterigmatocystin	C.I. direct brown 95	FD & C violet no. 1
Structure	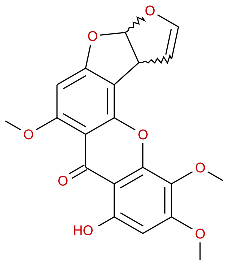	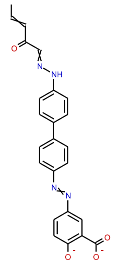	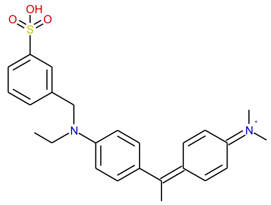
Actual Endpoint (-log C)	6.02361	5.31387	2.8543
Predicted Endpoint (-log C)	4.98771	4.30266	3.40838
Distance	0.743	0.767	0.768
Reference	CPDB	CPDB	CPDB

Model Applicability

Unknown features are fingerprint features in the query molecule, but not found or appearing too infrequently in the training set.

1. OPS PC27 out of range. Value: -3.5745. Training min, max, SD, explained variance: -2.8642, 4.2058, 1.002, 0.0096.

Feature Contribution

Top features for positive contribution			
**Fingerprint**	**Bit/Smiles**	**Feature Structure**	**Score**
FCFP_6	136627117	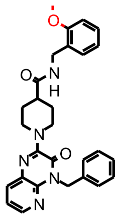 [*]OC	0.69

FCFP_6	565998553	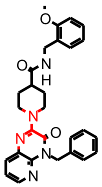 [*]\N=C(\N([*])[*])/C(=[*])[*]	0.357
FCFP_6	-2090462286	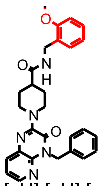 [*]O[c]1:[cH]:[cH]:[cH]:[cH]:[c]:1[*]	0.245

Top Features for negative contribution

Fingerprint	Bit/Smiles	Feature Structure	Score
FCFP_6	991735244	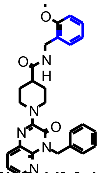 [*][c]1:[*]:[cH]:[cH]:[cH]:[cH]:1	-0.422
FCFP_6	-2093839777	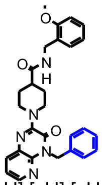 [*][c]1:[cH]:[cH]:[cH]:[cH]:[cH]:1	-0.378
FCFP_6	16	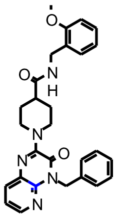 [*][c](:[*]):[*]	-0.354

#UNDEFINED

TOPKAT_Carcinogenic_Potency_TD50_Rat

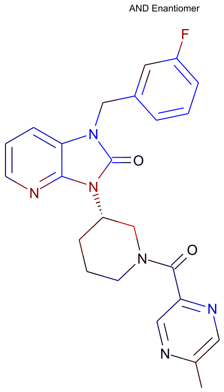

$C_{24}H_{23}FN_6O_2$
Molecular Weight: 446.47682
ALogP: 2.244
Rotatable Bonds: 4
Acceptors: 5
Donors: 0

Model Prediction

Prediction: 11.7
Unit: mg/kg_body_weight/day
Mahalanobis Distance: 18.8
Mahalanobis Distance p-value: 3.66e-024

Mahalanobis Distance: The Mahalanobis distance (MD) is a generalization of the Euclidean distance that accounts for correlations among the X properties. It is calculated as the distance to the center of the training data. The larger the MD, the less trustworthy the prediction.
Mahalanobis Distance p-value: The p-value gives the fraction of training data with an MD greater than or equal to the one for the given sample, assuming normally distributed data. The smaller the p-value, the less trustworthy the prediciton. For highly non-normal X properties (e.g., fingerprints), the MD p-value is wildly inaccurate.

Structural Similar Compounds

Name	4-Morpholino-2-(5-nitro-2-thi-enyl)quinazoline	5,6-Dimethoxysterigmatocystin	Omeprazole
Structure	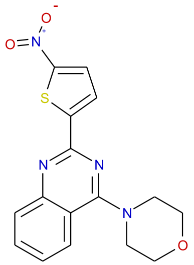	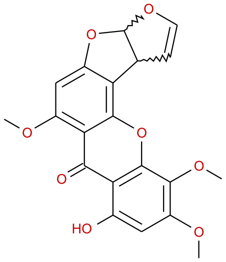	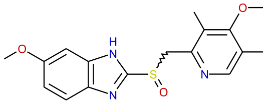
Actual Endpoint (-log C)	4.83293	6.02361	3.4628
Predicted Endpoint (-log C)	4.97658	4.98771	4.7324
Distance	0.724	0.732	0.759
Reference	CPDB	CPDB	CPDB

Model Applicability

Unknown features are fingerprint features in the query molecule, but not found or appearing too infrequently in the training set.

1. All properties and OPS components are within expected ranges.

Feature Contribution

Top features for positive contribution

Fingerprint	Bit/Smiles	Feature Structure	Score
FCFP_6	1	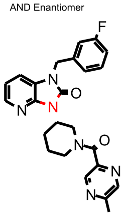 [*]N([*])[*]	0.234

FCFP_6	32	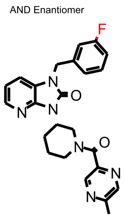 [*]F	0.154
FCFP_6	730557100	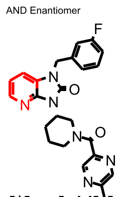 [*][c]1:[*]:n:[cH]:[cH]:[cH]:1	0.141

Top Features for negative contribution

Fingerprint	Bit/Smiles	Feature Structure	Score
FCFP_6	991735244	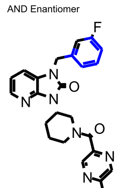 [*][c]1:[*]:[cH]:[cH]:[cH]:[cH]:1	-0.422
FCFP_6	16	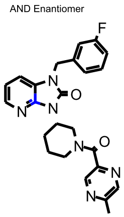 [*][c](:[*]):[*]	-0.354
FCFP_6	1674451008	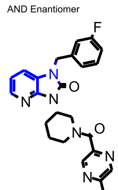 [*][c]1:[*]:[cH]:[cH]:[cH]:[c]:1N=[*]	-0.233

#UNDEFINED

TOPKAT_Carcinogenic_Potency_TD50_Rat

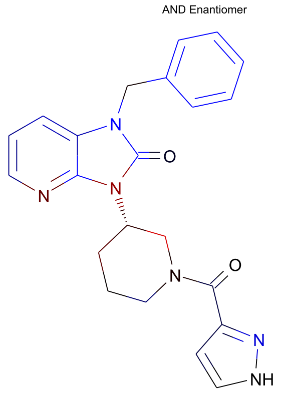

$C_{22}H_{22}N_6O_2$

Molecular Weight: 402.44907

ALogP: 2.501

Rotatable Bonds: 4

Acceptors: 4

Donors: 1

Model Prediction

Prediction: 39.9

Unit: mg/kg_body_weight/day

Mahalanobis Distance: 17.6

Mahalanobis Distance p-value: 8.04e-020

Mahalanobis Distance: The Mahalanobis distance (MD) is a generalization of the Euclidean distance that accounts for correlations among the X properties. It is calculated as the distance to the center of the training data. The larger the MD, the less trustworthy the prediction.
Mahalanobis Distance p-value: The p-value gives the fraction of training data with an MD greater than or equal to the one for the given sample, assuming normally distributed data. The smaller the p-value, the less trustworthy the prediciton. For highly non-normal X properties (e.g., fingerprints), the MD p-value is wildly inaccurate.

Structural Similar Compounds

Name	Omeprazole	4-Morpholino-2-(5-nitro-2-thi-enyl)quinazoline	5,6-Dimethoxysterigmatocystin
Structure	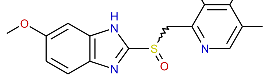	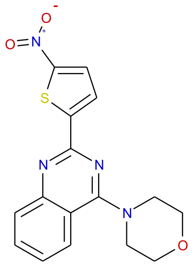	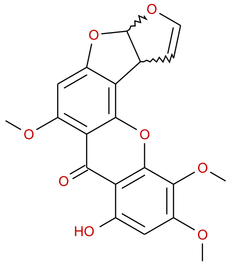
Actual Endpoint (-log C)	3.4628	4.83293	6.02361
Predicted Endpoint (-log C)	4.7324	4.97658	4.98771
Distance	0.706	0.719	0.721
Reference	CPDB	CPDB	CPDB

Model Applicability

Unknown features are fingerprint features in the query molecule, but not found or appearing too infrequently in the training set.

1. All properties and OPS components are within expected ranges.

Feature Contribution

Top features for positive contribution

Fingerprint	Bit/Smiles	Feature Structure	Score
FCFP_6	1	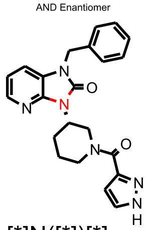 [*]N([*])[*]	0.234

FCFP_6	730557100	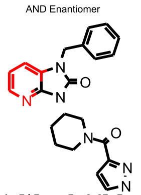 [*][c]1:[*]:n:[cH]:[cH]:[cH]:1	0.141
FCFP_6	203677720	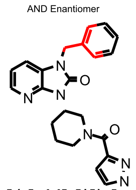 [*]C[c](:[cH]:[*]):[c]([*]):[*]	0.137
**Top Features for negative contribution**			
**Fingerprint**	**Bit/Smiles**	**Feature Structure**	**Score**
FCFP_6	991735244	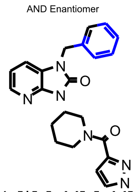 [*][c]1:[*]:[cH]:[cH]:[cH]:[cH]:1	-0.422
FCFP_6	-2093839777	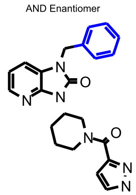 [*][c]1:[cH]:[cH]:[cH]:[cH]:[cH]:1	-0.378
FCFP_6	16	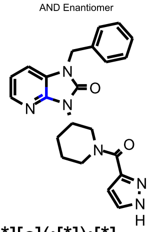 [*][c](:[*]):[*]	-0.354

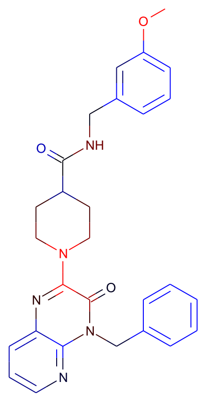

$C_{28}H_{29}N_5O_3$
Molecular Weight: 483.56156
ALogP: 2.942
Rotatable Bonds: 7
Acceptors: 6
Donors: 1

Model Prediction

Prediction: 2.95
Unit: mg/kg_body_weight/day
Mahalanobis Distance: 18
Mahalanobis Distance p-value: 3.53e-021

Mahalanobis Distance: The Mahalanobis distance (MD) is a generalization of the Euclidean distance that accounts for correlations among the X properties. It is calculated as the distance to the center of the training data. The larger the MD, the less trustworthy the prediction.
Mahalanobis Distance p-value: The p-value gives the fraction of training data with an MD greater than or equal to the one for the given sample, assuming normally distributed data. The smaller the p-value, the less trustworthy the prediciton. For highly non-normal X properties (e.g., fingerprints), the MD p-value is wildly inaccurate.

Structural Similar Compounds

Name	5,6-Dimethoxysterigmatocystin	C.I. direct brown 95	FD & C violet no. 1
Structure	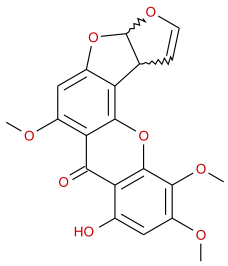	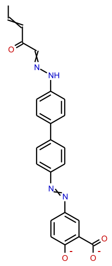	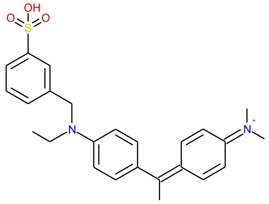
Actual Endpoint (-log C)	6.02361	5.31387	2.8543
Predicted Endpoint (-log C)	4.98771	4.30266	3.40838
Distance	0.748	0.764	0.770
Reference	CPDB	CPDB	CPDB

Model Applicability

Unknown features are fingerprint features in the query molecule, but not found or appearing too infrequently in the training set.

1. OPS PC5 out of range. Value: -7.9563. Training min, max, SD, explained variance: -7.7557, 7.4225, 2.16, 0.0448.

Feature Contribution

Top features for positive contribution			
**Fingerprint**	**Bit/Smiles**	**Feature Structure**	**Score**
FCFP_6	136627117	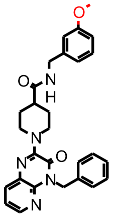 [*]OC	0.69

FCFP_6	565998553	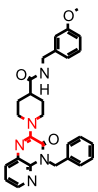 [*]\N=C(\N([*])[*])/C(=[*])[*]	0.357
FCFP_6	1	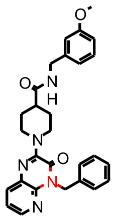 [*]N([*])[*]	0.234

Top Features for negative contribution

Fingerprint	Bit/Smiles	Feature Structure	Score
FCFP_6	991735244	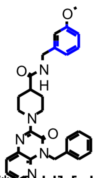 [*][c]1:[*]:[cH]:[cH]:[cH]:[cH]:1	-0.422
FCFP_6	-2093839777	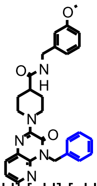 [*][c]1:[cH]:[cH]:[cH]:[cH]:[cH]:1	-0.378
FCFP_6	16	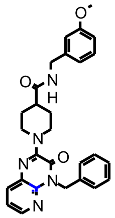 [*][c](:[*]):[*]	-0.354

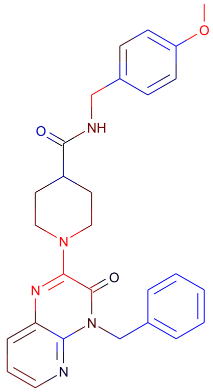

$C_{28}H_{29}N_5O_3$
Molecular Weight: 483.56156
ALogP: 2.942
Rotatable Bonds: 7
Acceptors: 6
Donors: 1

Structural Similar Compounds

Name	5,6-Dimethoxysterigmatocystin	C.I. direct brown 95	FD & C violet no. 1
Structure	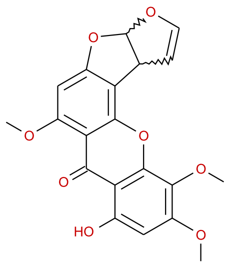	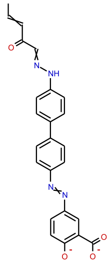	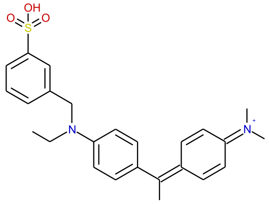
Actual Endpoint (-log C)	6.02361	5.31387	2.8543
Predicted Endpoint (-log C)	4.98771	4.30266	3.40838
Distance	0.747	0.767	0.768
Reference	CPDB	CPDB	CPDB

Model Prediction

Prediction: 2.11
Unit: mg/kg_body_weight/day
Mahalanobis Distance: 16.9
Mahalanobis Distance p-value: 2.62e-017

Mahalanobis Distance: The Mahalanobis distance (MD) is a generalization of the Euclidean distance that accounts for correlations among the X properties. It is calculated as the distance to the center of the training data. The larger the MD, the less trustworthy the prediction.
Mahalanobis Distance p-value: The p-value gives the fraction of training data with an MD greater than or equal to the one for the given sample, assuming normally distributed data. The smaller the p-value, the less trustworthy the prediciton. For highly non-normal X properties (e.g., fingerprints), the MD p-value is wildly inaccurate.

Model Applicability

Unknown features are fingerprint features in the query molecule, but not found or appearing too infrequently in the training set.

1. All properties and OPS components are within expected ranges.

Feature Contribution

Top features for positive contribution

Fingerprint	Bit/Smiles	Feature Structure	Score
FCFP_6	136627117	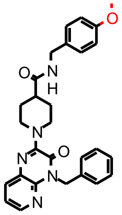 [*]OC	0.69

FCFP_6	565998553	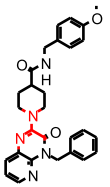 [*]\N=C(\N([*])[*])/C (=[*])[*]	0.357
FCFP_6	1	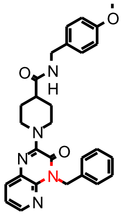 [*]N([*])[*]	0.234
**Top Features for negative contribution**			
**Fingerprint**	**Bit/Smiles**	**Feature Structure**	**Score**
FCFP_6	991735244	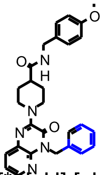 [*][c]1:[*]:[cH]:[cH] :[cH]:[cH]:1	-0.422
FCFP_6	-2093839777	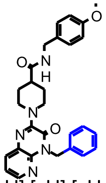 [*][c]1:[cH]:[cH]:[cH]:[cH]:[cH]:1	-0.378
FCFP_6	16	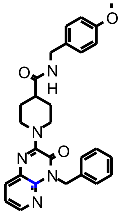 [*][c](:[*]):[*]	-0.354

Molecule

TOPKAT_Chronic_LOAEL

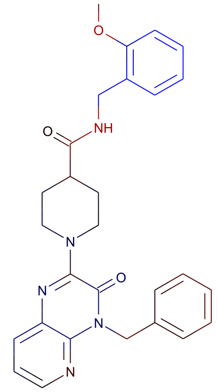

$C_{28}H_{29}N_5O_3$

Molecular Weight: 483.56156

ALogP: 2.942

Rotatable Bonds: 7

Acceptors: 6

Donors: 1

Model Prediction

Prediction: 0.0258

Unit: g/kg_body_weight

Mahalanobis Distance: 31.9

Mahalanobis Distance p-value: 3.97e-028

Mahalanobis Distance: The Mahalanobis distance (MD) is a generalization of the Euclidean distance that accounts for correlations among the X properties. It is calculated as the distance to the center of the training data. The larger the MD, the less trustworthy the prediction.

Mahalanobis Distance p-value: The p-value gives the fraction of training data with an MD greater than or equal to the one for the given sample, assuming normally distributed data. The smaller the p-value, the less trustworthy the prediciton. For highly non-normal X properties (e.g., fingerprints), the MD p-value is wildly inaccurate.

Structural Similar Compounds

Name	DILTIAZEM	RHODAMINE 6G	ASSURE
Structure	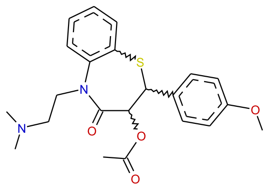	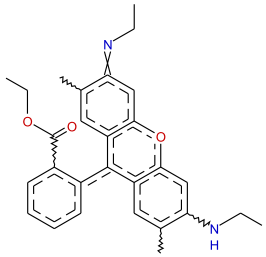	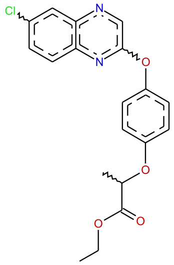
Actual Endpoint (-log C)	4.21961	4.54906	5.00328
Predicted Endpoint (-log C)	4.005	4.6787	4.27671
Distance	0.621	0.698	0.709
Reference	NDA-18602	NTP 364 39	EPA COVER SHEET 0335;891001;(1)

Model Applicability

Unknown features are fingerprint features in the query molecule, but not found or appearing too infrequently in the training set.

1. OPS PC18 out of range. Value: -4.8817. Training min, max, SD, explained variance: -4.7991, 6.1674, 1.831, 0.0147.
2. OPS PC22 out of range. Value: -4.3897. Training min, max, SD, explained variance: -4.3287, 5.3383, 1.588, 0.0110.
3. Unknown ECFP_6 feature: -661097313: [*]CN(C(=[*])[*])[c](:[*]):[*]
4. Unknown ECFP_6 feature: 671679640: [*]\N=C(\N([*])[*])/C(=[*])[*]
5. Unknown ECFP_6 feature: 1945129186: [*]N([*])C(=O)C(=[*])[*]
6. Unknown ECFP_6 feature: 2085698692: [*]C(=N[c](:[*]):[*])[*]
7. Unknown ECFP_6 feature: -509950643: [*]N([*])[c](:n:[*]):[c]([*]):[*]
8. Unknown ECFP_6 feature: 1951894094: [*]CN(C[*])C(=[*])[*]
9. Unknown ECFP_6 feature: -597295171: [*][c](:[*]):[c](:[cH]:[*])N=[*]
10. Unknown ECFP_6 feature: -81134287: [*]NC(=O)C([*])[*]
11. Unknown ECFP_6 feature: -44121127: [*]N([*])C[c](:[*]):[*]
12. Unknown ECFP_6 feature: 497523368: [*]CNC(=[*])[*]
13. Unknown ECFP_6 feature: -677309799: [*][c](:[*]):n:[cH]:[*]
14. Unknown ECFP_6 feature: -2024509555: [*]C[c](:[cH]:[*]):[c]([*]):[*]
15. Unknown ECFP_6 feature: -757679000: [*]CCN([*])[*]
16. Unknown ECFP_6 feature: -857146788: [*]CC(C[*])C(=[*])[*]
17. Unknown ECFP_6 feature: 769925792: [*]NC[c](:[*]):[*]
18. Unknown ECFP_6 feature: 1307307440: [*]:[c](:[*])OC

19. Unknown ECFP_6 feature: 1996163143: [*]:[cH]:[cH]:n:[*]
20. Unknown ECFP_6 feature: 1997021792: [*]:[cH]:[cH]:[cH]:[*]

Feature Contribution

Top features for positive contribution			
**Fingerprint**	**Bit/Smiles**	**Feature Structure**	**Score**
ECFP_6	-167460056	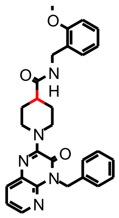 [*]C([*])[*]	0.136
ECFP_6	1559650422	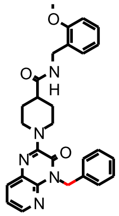 [*]C[*]	0.129
FCFP_6	3	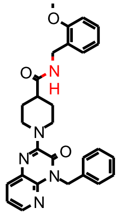 [*]N[*]	0.0924
**Top Features for negative contribution**			
**Fingerprint**	**Bit/Smiles**	**Feature Structure**	**Score**
FCFP_6	991735244	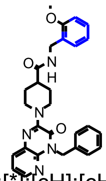 [*][c]1:[*]:[cH]:[cH]:[cH]:[cH]:1	-0.134

ECFP_6	1564392544	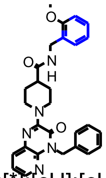 [*][c]1:[*]:[cH]:[cH]:[cH]:[cH]:1	-0.133
ECFP_6	2106656448	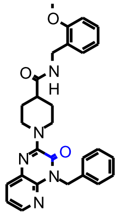 [*]C(=O)[*]	-0.11

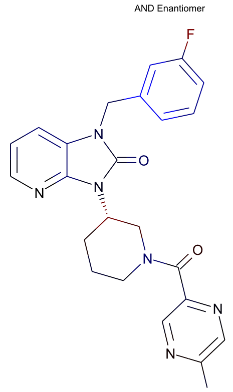

$C_{24}H_{23}FN_6O_2$
Molecular Weight: 446.47682
ALogP: 2.244
Rotatable Bonds: 4
Acceptors: 5
Donors: 0

Model Prediction

Prediction: 0.0476
Unit: g/kg_body_weight
Mahalanobis Distance: 32.2
Mahalanobis Distance p-value: 1.15e-028

Mahalanobis Distance: The Mahalanobis distance (MD) is a generalization of the Euclidean distance that accounts for correlations among the X properties. It is calculated as the distance to the center of the training data. The larger the MD, the less trustworthy the prediction.
Mahalanobis Distance p-value: The p-value gives the fraction of training data with an MD greater than or equal to the one for the given sample, assuming normally distributed data. The smaller the p-value, the less trustworthy the prediciton. For highly non-normal X properties (e.g., fingerprints), the MD p-value is wildly inaccurate.

Structural Similar Compounds

Name	DILTIAZEM	ROTENONE	ASSURE
Structure	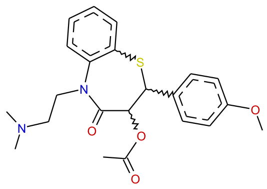	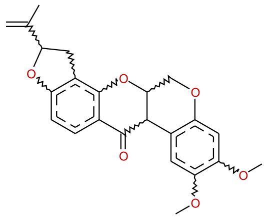	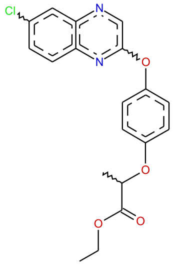
Actual Endpoint (-log C)	4.21961	5.0219	5.00328
Predicted Endpoint (-log C)	4.005	4.24871	4.27671
Distance	0.639	0.698	0.700
Reference	NDA-18602	NTP REPORT # 320	EPA COVER SHEET 0335;891001;(1)

Model Applicability

Unknown features are fingerprint features in the query molecule, but not found or appearing too infrequently in the training set.

1. All properties and OPS components are within expected ranges.
2. Unknown ECFP_6 feature: -1046436026: [*]F
3. Unknown ECFP_6 feature: -957084426: [*]C([*])N1C(=[*])[*][*]:[c]1:[*]
4. Unknown ECFP_6 feature: 1135573248: [*]N1[*]:[*]N([*])C1=O
5. Unknown ECFP_6 feature: -661097313: [*]CN(C(=[*])[*])[c](:[*]):[*]
6. Unknown ECFP_6 feature: -509950643: [*]N([*])[c](:n:[*]):[c]([*]):[*]
7. Unknown ECFP_6 feature: -1236953626: [*]N1[*][*][c](:[*]):[c]1:[cH]:[*]
8. Unknown ECFP_6 feature: -1102925512: [*]CN(C[*])C(=[*])[*]
9. Unknown ECFP_6 feature: 2077298510: [*]N([*])C(=O)[c](:[*]):[*]
10. Unknown ECFP_6 feature: -1869628272: [*]CC(C[*])N([*])[*]
11. Unknown ECFP_6 feature: 1413420509: [*]C(=[*])[c](:[cH]:[*]):n:[*]
12. Unknown ECFP_6 feature: -677309799: [*][c](:[*]):n:[cH]:[*]
13. Unknown ECFP_6 feature: -756348342: [*]C([*])CN([*])[*]
14. Unknown ECFP_6 feature: -44121127: [*]N([*])C[c](:[*]):[*]
15. Unknown ECFP_6 feature: -709633021: [*][c](:[*]):[cH]:n:[*]
16. Unknown ECFP_6 feature: 2146815437: [*]:[cH]:[c](C):n:[*]
17. Unknown ECFP_6 feature: -757679000: [*]CCN([*])[*]
18. Unknown ECFP_6 feature: -176686665: [*]:[cH]:[c](F):[cH]:[*]
19. Unknown ECFP_6 feature: 220735655: [*]:[c](:[*])F

20. Unknown ECFP_6 feature: -1332781180: [*]CCC[*]
21. Unknown ECFP_6 feature: 1997021792: [*]:[cH]:[cH]:[cH]:[*]
22. Unknown ECFP_6 feature: 1996163143: [*]:[cH]:[cH]:n:[*]

Feature Contribution

Top features for positive contribution

Fingerprint	Bit/Smiles	Feature Structure	Score
ECFP_6	-167460056	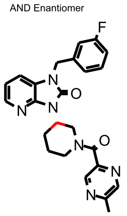 [*]C([*])[*]	0.136
ECFP_6	1559650422	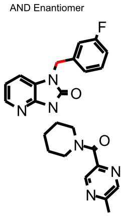 [*]C[*]	0.129
FCFP_6	32	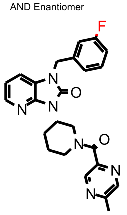 [*]F	0.101

Top Features for negative contribution

Fingerprint	Bit/Smiles	Feature Structure	Score

FCFP_6	991735244	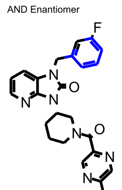 [*][c]1:[*]:[cH]:[cH]:[cH]:[cH]:1	-0.134
ECFP_6	2106656448	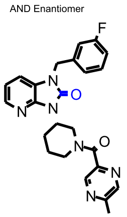 [*]C(=O)[*]	-0.11
FCFP_6	1	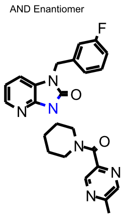 [*]N([*])[*]	-0.102

#UNDEFINED TOPKAT_Chronic_LOAEL

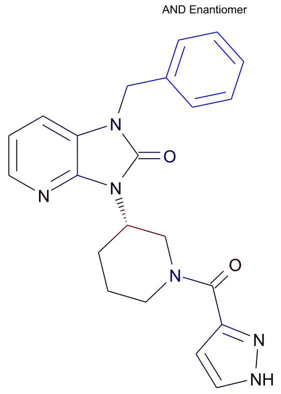

$C_{22}H_{22}N_6O_2$
Molecular Weight: 402.44907
ALogP: 2.501
Rotatable Bonds: 4
Acceptors: 4
Donors: 1

Model Prediction

Prediction: 0.157
Unit: g/kg_body_weight
Mahalanobis Distance: 35.6
Mahalanobis Distance p-value: 1.12e-034

Mahalanobis Distance: The Mahalanobis distance (MD) is a generalization of the Euclidean distance that accounts for correlations among the X properties. It is calculated as the distance to the center of the training data. The larger the MD, the less trustworthy the prediction.
Mahalanobis Distance p-value: The p-value gives the fraction of training data with an MD greater than or equal to the one for the given sample, assuming normally distributed data. The smaller the p-value, the less trustworthy the prediciton. For highly non-normal X properties (e.g., fingerprints), the MD p-value is wildly inaccurate.

Structural Similar Compounds

Name	FLUCONAZOLE	DILTIAZEM	QUININE SULFATE
Structure	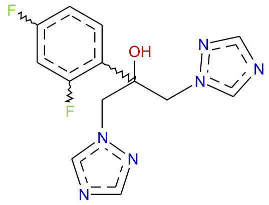	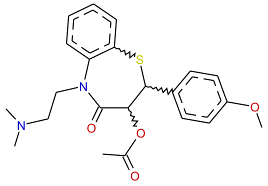	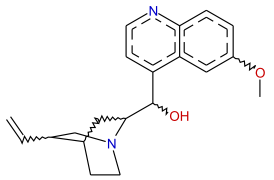
Actual Endpoint (-log C)	5.08818	4.21961	3.66601
Predicted Endpoint (-log C)	4.97396	4.005	5.04022
Distance	0.673	0.675	0.680
Reference	NDA-19949	NDA-18602	PATHOL.RES.PRAC.163.373.1978

Model Applicability

Unknown features are fingerprint features in the query molecule, but not found or appearing too infrequently in the training set.

1. All properties and OPS components are within expected ranges.
2. Unknown FCFP_2 feature: 262592487: [*]1:[*]:n:[nH]:[cH]:1
3. Unknown ECFP_6 feature: -152683720: [*]:[nH]:[*]
4. Unknown ECFP_6 feature: -957084426: [*]C([*])N1C(=[*])[*][*]:[c]1:[*]
5. Unknown ECFP_6 feature: 1135573248: [*]N1[*]:[*]N([*])C1=O
6. Unknown ECFP_6 feature: -661097313: [*]CN(C(=[*])[*])[c](:[*]):[*]
7. Unknown ECFP_6 feature: -509950643: [*]N([*])[c](:n:[*]):[c]([*]):[*]
8. Unknown ECFP_6 feature: -1236953626: [*]N1[*][*][c](:[*]):[c]1:[cH]:[*]
9. Unknown ECFP_6 feature: -1102925512: [*]CN(C[*])C(=[*])[*]
10. Unknown ECFP_6 feature: 2077298510: [*]N([*])C(=O)[c](:[*]):[*]
11. Unknown ECFP_6 feature: 1413420509: [*]C(=[*])[c](:[cH]:[*]):n:[*]
12. Unknown ECFP_6 feature: -1869628272: [*]CC(C[*])N([*])[*]
13. Unknown ECFP_6 feature: 600440273: [*][c]1:[*]:[*]:[nH]:n:1
14. Unknown ECFP_6 feature: -756348342: [*]C([*])CN([*])[*]
15. Unknown ECFP_6 feature: -677309799: [*][c](:[*]):n:[cH]:[*]
16. Unknown ECFP_6 feature: -954588747: [*]1:[*]:n:[nH]:[cH]:1
17. Unknown ECFP_6 feature: -44121127: [*]N([*])C[c](:[*]):[*]
18. Unknown ECFP_6 feature: 1998023064: [*]1:[*]:[cH]:[cH]:[nH]:1
19. Unknown ECFP_6 feature: -757679000: [*]CCN([*])[*]

20. Unknown ECFP_6 feature: -1332781180: [*]CCC[*]
21. Unknown ECFP_6 feature: 1996163143: [*]:[cH]:[cH]:n:[*]
22. Unknown ECFP_6 feature: 1997021792: [*]:[cH]:[cH]:[cH]:[*]

Feature Contribution

Top features for positive contribution			
**Fingerprint**	**Bit/Smiles**	**Feature Structure**	**Score**
ECFP_6	-167460056	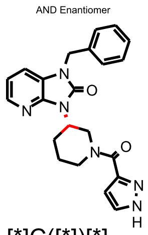 [*]C([*])[*]	0.136
ECFP_6	1559650422	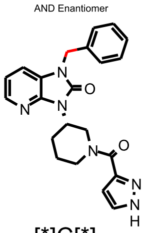 [*]C[*]	0.129
FCFP_6	-2093839777	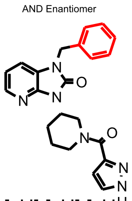 [*][c]1:[cH]:[cH]:[cH]:[cH]:[cH]:1	0.078

Top Features for negative contribution			
**Fingerprint**	**Bit/Smiles**	**Feature Structure**	**Score**

FCFP_6	991735244	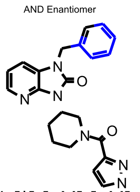 [*][c]1:[*]:[cH]:[cH]:[cH]:[cH]:1	-0.134
ECFP_6	1564392544	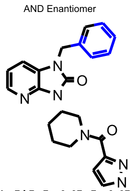 [*][c]1:[*]:[cH]:[cH]:[cH]:[cH]:1	-0.133
ECFP_6	2106656448	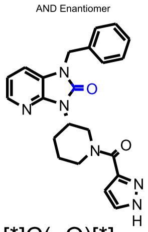 [*]C(=O)[*]	-0.11

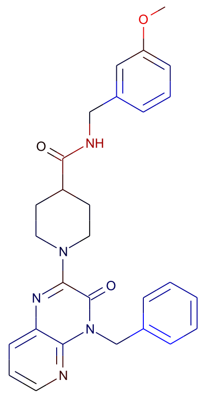

$C_{28}H_{29}N_5O_3$
Molecular Weight: 483.56156
ALogP: 2.942
Rotatable Bonds: 7
Acceptors: 6
Donors: 1

Model Prediction

Prediction: 0.0178
Unit: g/kg_body_weight
Mahalanobis Distance: 32.4
Mahalanobis Distance p-value: 6.19e-029

Mahalanobis Distance: The Mahalanobis distance (MD) is a generalization of the Euclidean distance that accounts for correlations among the X properties. It is calculated as the distance to the center of the training data. The larger the MD, the less trustworthy the prediction.
Mahalanobis Distance p-value: The p-value gives the fraction of training data with an MD greater than or equal to the one for the given sample, assuming normally distributed data. The smaller the p-value, the less trustworthy the prediciton. For highly non-normal X properties (e.g., fingerprints), the MD p-value is wildly inaccurate.

Structural Similar Compounds

Name	DILTIAZEM	RHODAMINE 6G	ASSURE
Structure	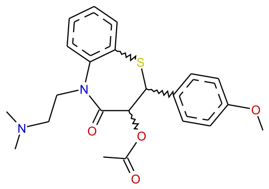	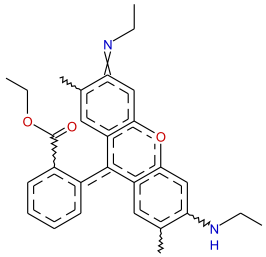	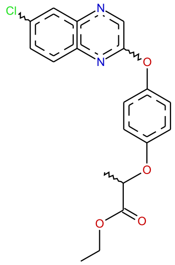
Actual Endpoint (-log C)	4.21961	4.54906	5.00328
Predicted Endpoint (-log C)	4.005	4.6787	4.27671
Distance	0.617	0.701	0.707
Reference	NDA-18602	NTP 364 39	EPA COVER SHEET 0335;891001;(1)

Model Applicability

Unknown features are fingerprint features in the query molecule, but not found or appearing too infrequently in the training set.

1. OPS PC18 out of range. Value: -4.812. Training min, max, SD, explained variance: -4.7991, 6.1674, 1.831, 0.0147.
2. OPS PC22 out of range. Value: -4.9558. Training min, max, SD, explained variance: -4.3287, 5.3383, 1.588, 0.0110.
3. Unknown ECFP_6 feature: -661097313: [*]CN(C(=[*])[*])[c](:[*]):[*]
4. Unknown ECFP_6 feature: 671679640: [*]\N=C(\N([*])[*])/C(=[*])[*]
5. Unknown ECFP_6 feature: 1945129186: [*]N([*])C(=O)C(=[*])[*]
6. Unknown ECFP_6 feature: 2085698692: [*]C(=N[c](:[*]):[*])[*]
7. Unknown ECFP_6 feature: -509950643: [*]N([*])[c](:n:[*]):[c]([*]):[*]
8. Unknown ECFP_6 feature: 1951894094: [*]CN(C[*])C(=[*])[*]
9. Unknown ECFP_6 feature: -597295171: [*][c](:[*]):[c](:[cH]:[*])N=[*]
10. Unknown ECFP_6 feature: -81134287: [*]NC(=O)C([*])[*]
11. Unknown ECFP_6 feature: -44121127: [*]N([*])C[c](:[*]):[*]
12. Unknown ECFP_6 feature: -677309799: [*][c](:[*]):n:[cH]:[*]
13. Unknown ECFP_6 feature: 497523368: [*]CNC(=[*])[*]
14. Unknown ECFP_6 feature: -757679000: [*]CCN([*])[*]
15. Unknown ECFP_6 feature: -857146788: [*]CC(C[*])C(=[*])[*]
16. Unknown ECFP_6 feature: 769925792: [*]NC[c](:[*]):[*]
17. Unknown ECFP_6 feature: 1307307440: [*]:[c](:[*])OC
18. Unknown ECFP_6 feature: 1997021792: [*]:[cH]:[cH]:[cH]:[*]

19.	Unknown ECFP_6 feature: 1996163143: [*]:[cH]:[cH]:n:[*]

Feature Contribution

Top features for positive contribution

Fingerprint	Bit/Smiles	Feature Structure	Score
ECFP_6	-167460056	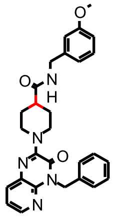 [*]C([*])[*]	0.136
ECFP_6	1559650422	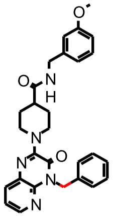 [*]C[*]	0.129
ECFP_6	-176455838	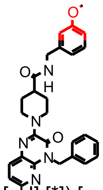 [*]O[c](:[cH]:[*]):[cH]:[*]	0.106

Top Features for negative contribution

Fingerprint	Bit/Smiles	Feature Structure	Score
FCFP_6	991735244	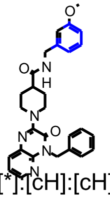 [*][c]1:[*]:[cH]:[cH]:[cH]:[cH]:1	-0.134

ECFP_6	1564392544	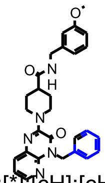 [*][c]1:[*]:[cH]:[cH]:[cH]:[cH]:1	-0.133
ECFP_6	2106656448	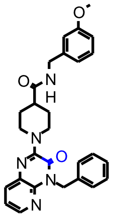 [*]C(=O)[*]	-0.11

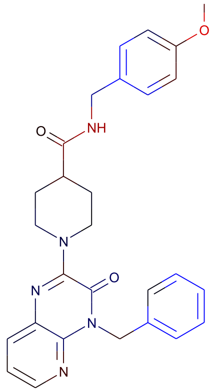

$C_{28}H_{29}N_5O_3$
Molecular Weight: 483.56156
ALogP: 2.942
Rotatable Bonds: 7
Acceptors: 6
Donors: 1

Structural Similar Compounds

Name	DILTIAZEM	RHODAMINE 6G	ASSURE
Structure	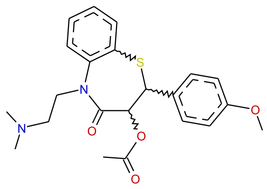	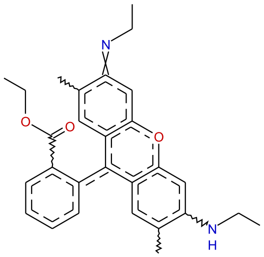	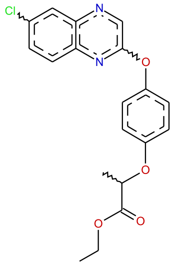
Actual Endpoint (-log C)	4.21961	4.54906	5.00328
Predicted Endpoint (-log C)	4.005	4.6787	4.27671
Distance	0.608	0.702	0.706
Reference	NDA-18602	NTP 364 39	EPA COVER SHEET 0335;891001;(1)

Model Prediction

Prediction: 0.0189
Unit: g/kg_body_weight
Mahalanobis Distance: 32.4
Mahalanobis Distance p-value: 6.19e-029

Mahalanobis Distance: The Mahalanobis distance (MD) is a generalization of the Euclidean distance that accounts for correlations among the X properties. It is calculated as the distance to the center of the training data. The larger the MD, the less trustworthy the prediction.
Mahalanobis Distance p-value: The p-value gives the fraction of training data with an MD greater than or equal to the one for the given sample, assuming normally distributed data. The smaller the p-value, the less trustworthy the prediciton. For highly non-normal X properties (e.g., fingerprints), the MD p-value is wildly inaccurate.

Model Applicability

Unknown features are fingerprint features in the query molecule, but not found or appearing too infrequently in the training set.

1. OPS PC18 out of range. Value: -4.812. Training min, max, SD, explained variance: -4.7991, 6.1674, 1.831, 0.0147.
2. OPS PC22 out of range. Value: -4.9558. Training min, max, SD, explained variance: -4.3287, 5.3383, 1.588, 0.0110.
3. Unknown ECFP_6 feature: -661097313: [*]CN(C(=[*])[*])[c](:[*]):[*]
4. Unknown ECFP_6 feature: 671679640: [*]\N=C(\N([*])[*])/C(=[*])[*]
5. Unknown ECFP_6 feature: 1945129186: [*]N([*])C(=O)C(=[*])[*]
6. Unknown ECFP_6 feature: 2085698692: [*]C(=N[c](:[*]):[*])[*]
7. Unknown ECFP_6 feature: -509950643: [*]N([*])[c](:n:[*]):[c]([*]):[*]
8. Unknown ECFP_6 feature: 1951894094: [*]CN(C[*])C(=[*])[*]
9. Unknown ECFP_6 feature: -597295171: [*][c](:[*]):[c](:[cH]:[*])N=[*]
10. Unknown ECFP_6 feature: -81134287: [*]NC(=O)C([*])[*]
11. Unknown ECFP_6 feature: -44121127: [*]N([*])C[c](:[*]):[*]
12. Unknown ECFP_6 feature: -677309799: [*][c](:[*]):n:[cH]:[*]
13. Unknown ECFP_6 feature: 497523368: [*]CNC(=[*])[*]
14. Unknown ECFP_6 feature: -757679000: [*]CCN([*])[*]
15. Unknown ECFP_6 feature: -857146788: [*]CC(C[*])C(=[*])[*]
16. Unknown ECFP_6 feature: 769925792: [*]NC[c](:[*]):[*]
17. Unknown ECFP_6 feature: 1307307440: [*]:[c](:[*])OC
18. Unknown ECFP_6 feature: 1996163143: [*]:[cH]:[cH]:n:[*]

19. Unknown ECFP_6 feature: 1997021792: [*]:[cH]:[cH]:[cH]:[*]

Feature Contribution

Top features for positive contribution

Fingerprint	Bit/Smiles	Feature Structure	Score
ECFP_6	-167460056	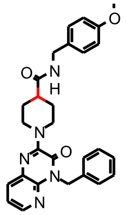 [*]C([*])[*]	0.136
ECFP_6	1559650422	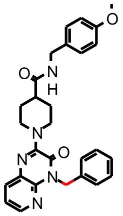 [*]C[*]	0.129
ECFP_6	-176455838	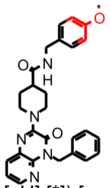 [*]O[c](:[cH]:[*]):[cH]:[*]	0.106

Top Features for negative contribution

Fingerprint	Bit/Smiles	Feature Structure	Score
FCFP_6	991735244	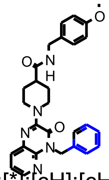 [*][c]1:[*]:[cH]:[cH]:[cH]:[cH]:1	-0.134

ECFP_6	1564392544	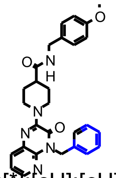 [*][c]1:[*]:[cH]:[cH]:[cH]:[cH]:1	-0.133
ECFP_6	2106656448	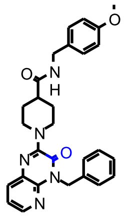 [*]C(=O)[*]	-0.11

Molecule

TOPKAT_Daphnia_EC50

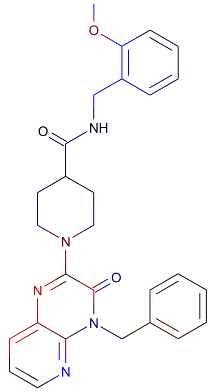

$C_{28}H_{29}N_5O_3$

Molecular Weight: 483.56156

ALogP: 2.942

Rotatable Bonds: 7

Acceptors: 6

Donors: 1

Structural Similar Compounds

Name	Naphthaleneacetic acid	3-Methoxybenzeneamine	Fenpyroximate
Structure	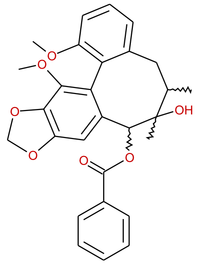	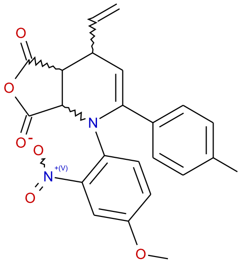	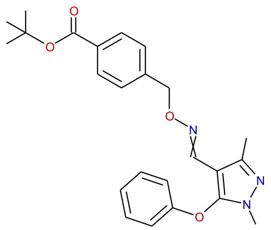
Actual Endpoint (-log C)	3.015	6.58436	8.068
Predicted Endpoint (-log C)	5.76302	5.62256	6.55124
Distance	0.592	0.696	0.719
Reference	Toropov and Benfenati, 2006, Bioorganic & Medicinal Chemistry, 14(8), 2779-2788	EPA EcoTox Database	Toropov and Benfenati, 2006, Bioorganic & Medicinal Chemistry, 14(8), 2779-2788

Model Prediction

Prediction: 0.5

Unit: mg/l

Mahalanobis Distance: 37.6

Mahalanobis Distance p-value: 8.14e-056

Mahalanobis Distance: The Mahalanobis distance (MD) is a generalization of the Euclidean distance that accounts for correlations among the X properties. It is calculated as the distance to the center of the training data. The larger the MD, the less trustworthy the prediction.
Mahalanobis Distance p-value: The p-value gives the fraction of training data with an MD greater than or equal to the one for the given sample, assuming normally distributed data. The smaller the p-value, the less trustworthy the prediciton. For highly non-normal X properties (e.g., fingerprints), the MD p-value is wildly inaccurate.

Model Applicability

Unknown features are fingerprint features in the query molecule, but not found or appearing too infrequently in the training set.

1. All properties and OPS components are within expected ranges.
2. Unknown ECFP_6 feature: -661097313: [*]CN(C(=[*])[*])[c](:[*]):[*]
3. Unknown ECFP_6 feature: 671679640: [*]\N=C(\N([*])[*])/C(=[*])[*]
4. Unknown ECFP_6 feature: 1945129186: [*]N([*])C(=O)C(=[*])[*]
5. Unknown ECFP_6 feature: 2085698692: [*]C(=N[c](:[*]):[*])[*]
6. Unknown ECFP_6 feature: -509950643: [*]N([*])[c](:n:[*]):[c]([*]):[*]
7. Unknown ECFP_6 feature: 1951894094: [*]CN(C[*])C(=[*])[*]
8. Unknown ECFP_6 feature: -597295171: [*][c](:[*]):[c](:[cH]:[*])N=[*]
9. Unknown ECFP_6 feature: -81134287: [*]NC(=O)C([*])[*]
10. Unknown ECFP_6 feature: -44121127: [*]N([*])C[c](:[*]):[*]
11. Unknown ECFP_6 feature: 497523368: [*]CNC(=[*])[*]
12. Unknown ECFP_6 feature: -677309799: [*][c](:[*]):n:[cH]:[*]
13. Unknown ECFP_6 feature: -2024509555: [*]C[c](:[cH]:[*]):[c]([*]):[*]
14. Unknown ECFP_6 feature: -757679000: [*]CCN([*])[*]
15. Unknown ECFP_6 feature: -857146788: [*]CC(C[*])C(=[*])[*]
16. Unknown ECFP_6 feature: 769925792: [*]NC[c](:[*]):[*]
17. Unknown ECFP_6 feature: -1331450522: [*]CCC([*])[*]
18. Unknown ECFP_6 feature: 1307307440: [*]:[c](:[*])OC

19. Unknown ECFP_6 feature: 1996163143: [*]:[cH]:[cH]:n:[*]

Feature Contribution

Top features for positive contribution

Fingerprint	Bit/Smiles	Feature Structure	Score
ECFP_6	-1059365320	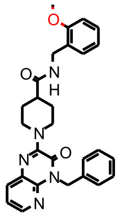 [*]O[*]	0.165
ECFP_6	642810091	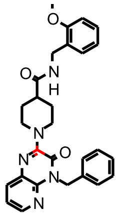 [*]C(=[*])[*]	0.148
FCFP_6	565998553	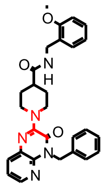 [*]\N=C(\N([*])[*])/C (=[*])[*]	0.114

Top Features for negative contribution

Fingerprint	Bit/Smiles	Feature Structure	Score
FCFP_6	0	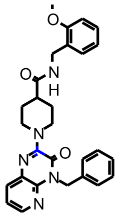 [*]C(=[*])[*]	-0.202

ECFP_6	-1100000244	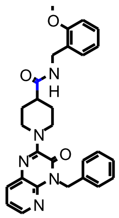 [*]C(=[*])[*]	-0.199
FCFP_6	17	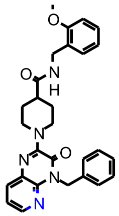 [*]:n:[*]	-0.189

#UNDEFINED

TOPKAT_Daphnia_EC50

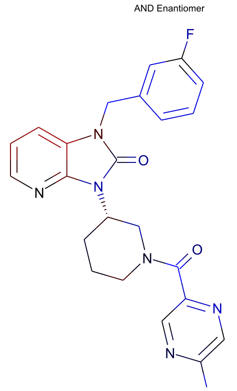

$C_{24}H_{23}FN_6O_2$

Molecular Weight: 446.47682

ALogP: 2.244

Rotatable Bonds: 4

Acceptors: 5

Donors: 0

Structural Similar Compounds

Name	Difenoconazole	Rotenone	Naphthaleneacetic acid
Structure	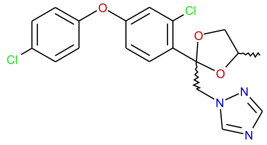	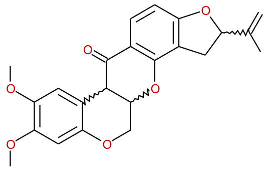	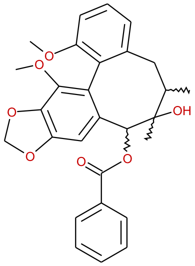
Actual Endpoint (-log C)	5.722	8.028	3.015
Predicted Endpoint (-log C)	5.71868	5.51369	5.76302
Distance	0.653	0.708	0.712
Reference	Toropov and Benfenati, 2006, Bioorganic & Medicinal Chemistry, 14(8), 2779-2788	Toropov and Benfenati, 2006, Bioorganic & Medicinal Chemistry, 14(8), 2779-2788	Toropov and Benfenati, 2006, Bioorganic & Medicinal Chemistry, 14(8), 2779-2788

Model Prediction

Prediction: 3.56

Unit: mg/l

Mahalanobis Distance: 38.5

Mahalanobis Distance p-value: 9.35e-059

Mahalanobis Distance: The Mahalanobis distance (MD) is a generalization of the Euclidean distance that accounts for correlations among the X properties. It is calculated as the distance to the center of the training data. The larger the MD, the less trustworthy the prediction.
Mahalanobis Distance p-value: The p-value gives the fraction of training data with an MD greater than or equal to the one for the given sample, assuming normally distributed data. The smaller the p-value, the less trustworthy the prediciton. For highly non-normal X properties (e.g., fingerprints), the MD p-value is wildly inaccurate.

Model Applicability

Unknown features are fingerprint features in the query molecule, but not found or appearing too infrequently in the training set.

1. All properties and OPS components are within expected ranges.
2. Unknown ECFP_6 feature: -957084426: [*]C([*])N1C(=[*])[*][*]:[c]1:[*]
3. Unknown ECFP_6 feature: 1135573248: [*]N1[*]:[*]N([*])C1=O
4. Unknown ECFP_6 feature: -661097313: [*]CN(C(=[*])[*])[c](:[*]):[*]
5. Unknown ECFP_6 feature: -509950643: [*]N([*])[c](:n:[*]):[c]([*]):[*]
6. Unknown ECFP_6 feature: -1236953626: [*]N1[*][*][c](:[*]):[c]1:[cH]:[*]
7. Unknown ECFP_6 feature: -1102925512: [*]CN(C[*])C(=[*])[*]
8. Unknown ECFP_6 feature: 2077298510: [*]N([*])C(=O)[c](:[*]):[*]
9. Unknown ECFP_6 feature: -1869628272: [*]CC(C[*])N([*])[*]
10. Unknown ECFP_6 feature: 1413420509: [*]C(=[*])[c](:[cH]:[*]):n:[*]
11. Unknown ECFP_6 feature: -677309799: [*][c](:[*]):n:[cH]:[*]
12. Unknown ECFP_6 feature: -756348342: [*]C([*])CN([*])[*]
13. Unknown ECFP_6 feature: -44121127: [*]N([*])C[c](:[*]):[*]
14. Unknown ECFP_6 feature: -709633021: [*][c](:[*]):[cH]:n:[*]
15. Unknown ECFP_6 feature: 2146815437: [*]:[cH]:[c](C):n:[*]
16. Unknown ECFP_6 feature: -757679000: [*]CCN([*])[*]
17. Unknown ECFP_6 feature: -176686665: [*]:[cH]:[c](F):[cH]:[*]
18. Unknown ECFP_6 feature: 220735655: [*]:[c](:[*])F

19. Unknown ECFP_6 feature: -1331450522: [*]CCC([*])[*]
20. Unknown ECFP_6 feature: -1332781180: [*]CCC[*]
21. Unknown ECFP_6 feature: 1996163143: [*]:[cH]:[cH]:n:[*]

Feature Contribution

Top features for positive contribution

Fingerprint	Bit/Smiles	Feature Structure	Score
ECFP_6	642810091	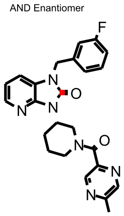 [*]C(=[*])[*]	0.148
FCFP_6	-98332825	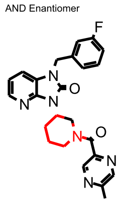 [*][C@@H]1[*]N([*])CC C1	0.0931
ECFP_6	1996767644	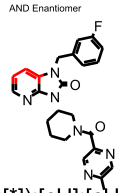 [*][c](:[*]):[cH]:[cH]:[*]	0.0755

Top Features for negative contribution

Fingerprint	Bit/Smiles	Feature Structure	Score

FCFP_6	0	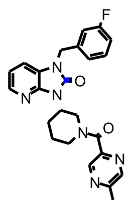 [*]C(=[*])[*]	-0.202
ECFP_6	-1100000244	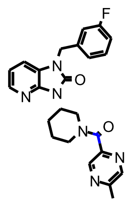 [*]C(=[*])[*]	-0.199
FCFP_6	17	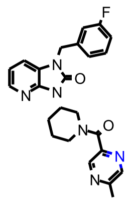 [*]:n:[*]	-0.189

#UNDEFINED

TOPKAT_Daphnia_EC50

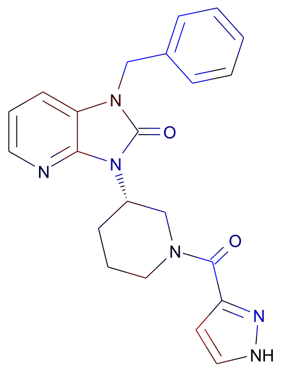

$C_{22}H_{22}N_6O_2$
Molecular Weight: 402.44907
ALogP: 2.501
Rotatable Bonds: 4
Acceptors: 4
Donors: 1

Structural Similar Compounds

Name	Difenoconazole	3-Methoxybenzeneamine	Naphthaleneacetic acid
Structure	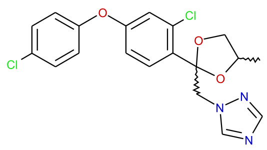	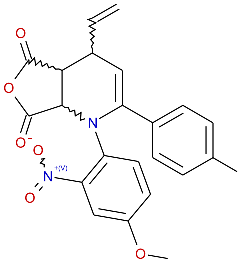	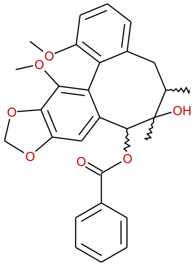
Actual Endpoint (-log C)	5.722	6.58436	3.015
Predicted Endpoint (-log C)	5.71868	5.62256	5.76302
Distance	0.679	0.735	0.737
Reference	Toropov and Benfenati, 2006, Bioorganic & Medicinal Chemistry, 14(8), 2779-2788	EPA EcoTox Database	Toropov and Benfenati, 2006, Bioorganic & Medicinal Chemistry, 14(8), 2779-2788

Model Prediction

Prediction: 4.14
Unit: mg/l
Mahalanobis Distance: 38
Mahalanobis Distance p-value: 2.82e-057

Mahalanobis Distance: The Mahalanobis distance (MD) is a generalization of the Euclidean distance that accounts for correlations among the X properties. It is calculated as the distance to the center of the training data. The larger the MD, the less trustworthy the prediction.
Mahalanobis Distance p-value: The p-value gives the fraction of training data with an MD greater than or equal to the one for the given sample, assuming normally distributed data. The smaller the p-value, the less trustworthy the prediciton. For highly non-normal X properties (e.g., fingerprints), the MD p-value is wildly inaccurate.

Model Applicability

Unknown features are fingerprint features in the query molecule, but not found or appearing too infrequently in the training set.

1. All properties and OPS components are within expected ranges.
2. Unknown FCFP_2 feature: 1747267175: [*][c]1:[*]:[*]:[nH]:n:1
3. Unknown FCFP_2 feature: 262592487: [*]1:[*]:n:[nH]:[cH]:1
4. Unknown ECFP_6 feature: -152683720: [*]:[nH]:[*]
5. Unknown ECFP_6 feature: -957084426: [*]C([*])N1C(=[*])[*][*]:[c]1:[*]
6. Unknown ECFP_6 feature: 1135573248: [*]N1[*]:[*]N([*])C1=O
7. Unknown ECFP_6 feature: -661097313: [*]CN(C(=[*])[*])[c](:[*]):[*]
8. Unknown ECFP_6 feature: -509950643: [*]N([*])[c](:n:[*]):[c]([*]):[*]
9. Unknown ECFP_6 feature: -1236953626: [*]N1[*][*][c](:[*]):[c]1:[cH]:[*]
10. Unknown ECFP_6 feature: -1102925512: [*]CN(C[*])C(=[*])[*]
11. Unknown ECFP_6 feature: 2077298510: [*]N([*])C(=O)[c](:[*]):[*]
12. Unknown ECFP_6 feature: 1413420509: [*]C(=[*])[c](:[cH]:[*]):n:[*]
13. Unknown ECFP_6 feature: -1869628272: [*]CC(C[*])N([*])[*]
14. Unknown ECFP_6 feature: 600440273: [*][c]1:[*]:[*]:[nH]:n:1
15. Unknown ECFP_6 feature: -756348342: [*]C([*])CN([*])[*]
16. Unknown ECFP_6 feature: -677309799: [*][c](:[*]):n:[cH]:[*]
17. Unknown ECFP_6 feature: -954588747: [*]1:[*]:n:[nH]:[cH]:1
18. Unknown ECFP_6 feature: -44121127: [*]N([*])C[c](:[*]):[*]

19. Unknown ECFP_6 feature: 1998023064: [*]1:[*]:[cH]:[cH]:[nH]:1
20. Unknown ECFP_6 feature: -757679000: [*]CCN([*])[*]
21. Unknown ECFP_6 feature: -1331450522: [*]CCC([*])[*]
22. Unknown ECFP_6 feature: -1332781180: [*]CCC[*]
23. Unknown ECFP_6 feature: 1996163143: [*]:[cH]:[cH]:n:[*]

Feature Contribution

Top features for positive contribution

Fingerprint	Bit/Smiles	Feature Structure	Score
ECFP_6	642810091	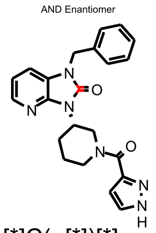 [*]C(=[*])[*]	0.148
FCFP_6	-98332825	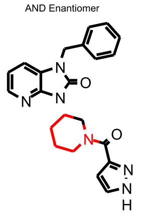 [*][C@@H]1[*]N([*])CC C1	0.0931
ECFP_6	1564392544	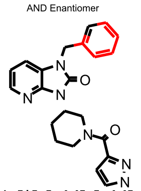 [*][c]1:[*]:[cH]:[cH] :[cH]:[cH]:1	0.082

Top Features for negative contribution

Fingerprint	Bit/Smiles	Feature Structure	Score

FCFP_6	0	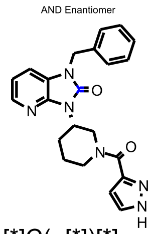 [*]C(=[*])[*]	-0.202
ECFP_6	-1100000244	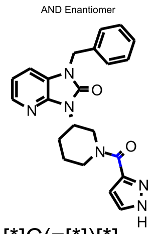 [*]C(=[*])[*]	-0.199
FCFP_6	17	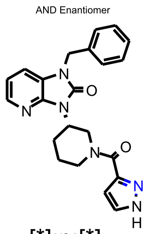 [*]:n:[*]	-0.189

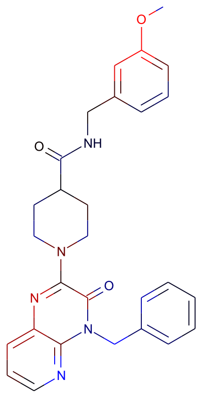

$C_{28}H_{29}N_5O_3$
Molecular Weight: 483.56156
ALogP: 2.942
Rotatable Bonds: 7
Acceptors: 6
Donors: 1

Structural Similar Compounds

Name	Naphthaleneacetic acid	3-Methoxybenzeneamine	Fenpyroximate
Structure	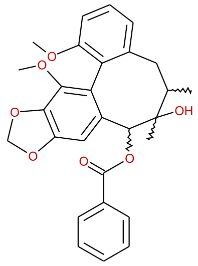	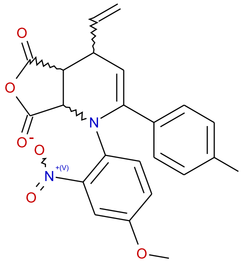	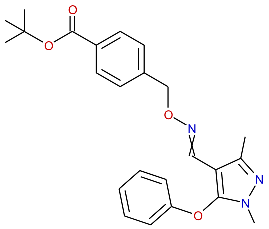
Actual Endpoint (-log C)	3.015	6.58436	8.068
Predicted Endpoint (-log C)	5.76302	5.62256	6.55124
Distance	0.590	0.689	0.718
Reference	Toropov and Benfenati, 2006, Bioorganic & Medicinal Chemistry, 14(8), 2779-2788	EPA EcoTox Database	Toropov and Benfenati, 2006, Bioorganic & Medicinal Chemistry, 14(8), 2779-2788

Model Prediction

Prediction: 0.297
Unit: mg/l
Mahalanobis Distance: 39.7
Mahalanobis Distance p-value: 1.81e-062

Mahalanobis Distance: The Mahalanobis distance (MD) is a generalization of the Euclidean distance that accounts for correlations among the X properties. It is calculated as the distance to the center of the training data. The larger the MD, the less trustworthy the prediction.
Mahalanobis Distance p-value: The p-value gives the fraction of training data with an MD greater than or equal to the one for the given sample, assuming normally distributed data. The smaller the p-value, the less trustworthy the prediciton. For highly non-normal X properties (e.g., fingerprints), the MD p-value is wildly inaccurate.

Model Applicability

Unknown features are fingerprint features in the query molecule, but not found or appearing too infrequently in the training set.

1. All properties and OPS components are within expected ranges.
2. Unknown ECFP_6 feature: -661097313: [*]CN(C(=[*])[*])[c](:[*]):[*]
3. Unknown ECFP_6 feature: 671679640: [*]\N=C(\N([*])[*])/C(=[*])[*]
4. Unknown ECFP_6 feature: 1945129186: [*]N([*])C(=O)C(=[*])[*]
5. Unknown ECFP_6 feature: 2085698692: [*]C(=N[c](:[*]):[*])[*]
6. Unknown ECFP_6 feature: -509950643: [*]N([*])[c](:n:[*]):[c]([*]):[*]
7. Unknown ECFP_6 feature: 1951894094: [*]CN(C[*])C(=[*])[*]
8. Unknown ECFP_6 feature: -597295171: [*][c](:[*]):[c](:[cH]:[*])N=[*]
9. Unknown ECFP_6 feature: -81134287: [*]NC(=O)C([*])[*]
10. Unknown ECFP_6 feature: -44121127: [*]N([*])C[c](:[*]):[*]
11. Unknown ECFP_6 feature: -677309799: [*][c](:[*]):n:[cH]:[*]
12. Unknown ECFP_6 feature: 497523368: [*]CNC(=[*])[*]
13. Unknown ECFP_6 feature: -757679000: [*]CCN([*])[*]
14. Unknown ECFP_6 feature: -857146788: [*]CC(C[*])C(=[*])[*]
15. Unknown ECFP_6 feature: -1331450522: [*]CCC([*])[*]
16. Unknown ECFP_6 feature: 769925792: [*]NC[c](:[*]):[*]
17. Unknown ECFP_6 feature: 1307307440: [*]:[c](:[*])OC
18. Unknown ECFP_6 feature: 1996163143: [*]:[cH]:[cH]:n:[*]

Feature Contribution

Top features for positive contribution			
**Fingerprint**	**Bit/Smiles**	**Feature Structure**	**Score**
ECFP_6	-1059365320	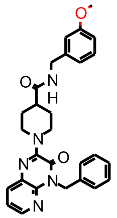<br>[*]O[*]	0.165
ECFP_6	642810091	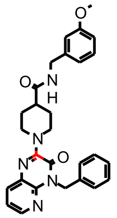<br>[*]C(=[*])[*]	0.148
FCFP_6	565998553	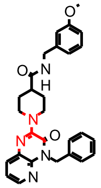<br>[*]\N=C(\N([*])[*])/C (=[*])[*]	0.114

Top Features for negative contribution			
**Fingerprint**	**Bit/Smiles**	**Feature Structure**	**Score**
FCFP_6	0	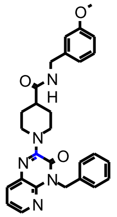<br>[*]C(=[*])[*]	-0.202

ECFP_6	-1100000244	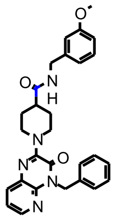 [*]C(=[*])[*]	-0.199
FCFP_6	17	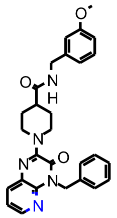 [*]:n:[*]	-0.189

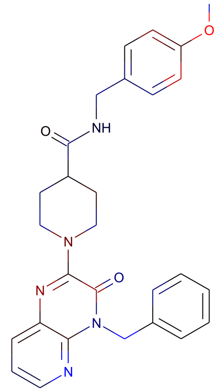

$C_{28}H_{29}N_5O_3$

Molecular Weight: 483.56156

ALogP: 2.942

Rotatable Bonds: 7

Acceptors: 6

Donors: 1

Structural Similar Compounds

Name	Naphthaleneacetic acid	3-Methoxybenzeneamine	Fenpyroximate
Structure	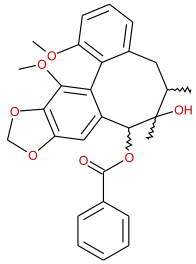	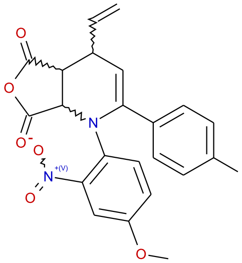	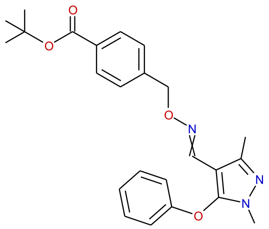
Actual Endpoint (-log C)	3.015	6.58436	8.068
Predicted Endpoint (-log C)	5.76302	5.62256	6.55124
Distance	0.594	0.690	0.717
Reference	Toropov and Benfenati, 2006, Bioorganic & Medicinal Chemistry, 14(8), 2779-2788	EPA EcoTox Database	Toropov and Benfenati, 2006, Bioorganic & Medicinal Chemistry, 14(8), 2779-2788

Model Prediction

Prediction: 0.41

Unit: mg/l

Mahalanobis Distance: 38.1

Mahalanobis Distance p-value: 2.16e-057

Mahalanobis Distance: The Mahalanobis distance (MD) is a generalization of the Euclidean distance that accounts for correlations among the X properties. It is calculated as the distance to the center of the training data. The larger the MD, the less trustworthy the prediction.
Mahalanobis Distance p-value: The p-value gives the fraction of training data with an MD greater than or equal to the one for the given sample, assuming normally distributed data. The smaller the p-value, the less trustworthy the prediciton. For highly non-normal X properties (e.g., fingerprints), the MD p-value is wildly inaccurate.

Model Applicability

Unknown features are fingerprint features in the query molecule, but not found or appearing too infrequently in the training set.

1. All properties and OPS components are within expected ranges.
2. Unknown ECFP_6 feature: -661097313: [*]CN(C(=[*])[*])[c](:[*]):[*]
3. Unknown ECFP_6 feature: 671679640: [*]\N=C(\N([*])[*])/C(=[*])[*]
4. Unknown ECFP_6 feature: 1945129186: [*]N([*])C(=O)C(=[*])[*]
5. Unknown ECFP_6 feature: 2085698692: [*]C(=N[c](:[*]):[*])[*]
6. Unknown ECFP_6 feature: -509950643: [*]N([*])[c](:n:[*]):[c]([*]):[*]
7. Unknown ECFP_6 feature: 1951894094: [*]CN(C[*])C(=[*])[*]
8. Unknown ECFP_6 feature: -597295171: [*][c](:[*]):[c](:[cH]:[*])N=[*]
9. Unknown ECFP_6 feature: -81134287: [*]NC(=O)C([*])[*]
10. Unknown ECFP_6 feature: -44121127: [*]N([*])C[c](:[*]):[*]
11. Unknown ECFP_6 feature: -677309799: [*][c](:[*]):n:[cH]:[*]
12. Unknown ECFP_6 feature: 497523368: [*]CNC(=[*])[*]
13. Unknown ECFP_6 feature: -757679000: [*]CCN([*])[*]
14. Unknown ECFP_6 feature: -857146788: [*]CC(C[*])C(=[*])[*]
15. Unknown ECFP_6 feature: -1331450522: [*]CCC([*])[*]
16. Unknown ECFP_6 feature: 769925792: [*]NC[c](:[*]):[*]
17. Unknown ECFP_6 feature: 1307307440: [*]:[c](:[*])OC
18. Unknown ECFP_6 feature: 1996163143: [*]:[cH]:[cH]:n:[*]

Feature Contribution

Top features for positive contribution			
**Fingerprint**	**Bit/Smiles**	**Feature Structure**	**Score**
ECFP_6	-1059365320	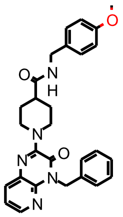 [*]O[*]	0.165
ECFP_6	642810091	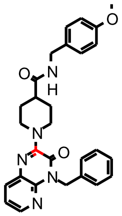 [*]C(=[*])[*]	0.148
FCFP_6	565998553	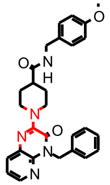 [*]\N=C(\N([*])[*])/C (=[*])[*]	0.114

Top Features for negative contribution			
**Fingerprint**	**Bit/Smiles**	**Feature Structure**	**Score**
FCFP_6	0	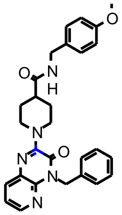 [*]C(=[*])[*]	-0.202

ECFP_6	-1100000244	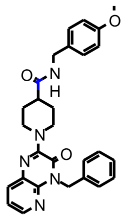 [*]C(=[*])[*]	-0.199
FCFP_6	17	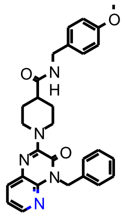 [*]:n:[*]	-0.189

Molecule

TOPKAT_Fathead_Minnow_LC50

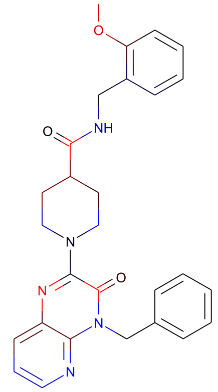

$C_{28}H_{29}N_5O_3$

Molecular Weight: 483.56156

ALogP: 2.942

Rotatable Bonds: 7

Acceptors: 6

Donors: 1

Model Prediction

Prediction: 0.00096

Unit: g/l

Mahalanobis Distance: 14.3

Mahalanobis Distance p-value: 4.05e-017

Mahalanobis Distance: The Mahalanobis distance (MD) is a generalization of the Euclidean distance that accounts for correlations among the X properties. It is calculated as the distance to the center of the training data. The larger the MD, the less trustworthy the prediction.
Mahalanobis Distance p-value: The p-value gives the fraction of training data with an MD greater than or equal to the one for the given sample, assuming normally distributed data. The smaller the p-value, the less trustworthy the prediciton. For highly non-normal X properties (e.g., fingerprints), the MD p-value is wildly inaccurate.

Structural Similar Compounds

Name	Rotenone	Rotenone	Flucythrinate
Structure	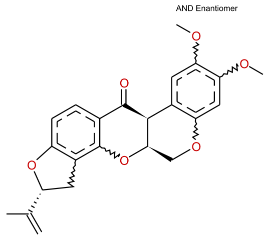	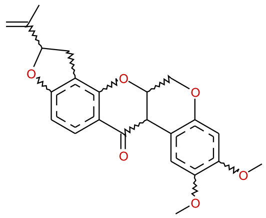	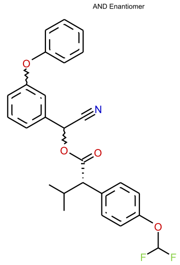
Actual Endpoint (-log C)	7.87943	7.942	9.37572
Predicted Endpoint (-log C)	6.1742	6.1742	8.49266
Distance	0.890	0.890	0.950
Reference	DSSTox/EPAFHM	ATOCFM Volume 5	DSSTox/EPAFHM

Model Applicability

Unknown features are fingerprint features in the query molecule, but not found or appearing too infrequently in the training set.

1. OPS PC9 out of range. Value: -5.4786. Training min, max, SD, explained variance: -4.4045, 4.4278, 1.418, 0.0346.
2. Unknown FCFP_2 feature: 580453787: [*]C(=N[c](:[*]):[*])[*]

Feature Contribution

Top features for positive contribution

Fingerprint	Bit/Smiles	Feature Structure	Score
FCFP_2	-1043339860	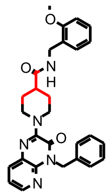 [*]CC(C[*])C(=[*])[*]	0.305

Fingerprint	Bit/Smiles	Feature Structure	Score
FCFP_2	565998553	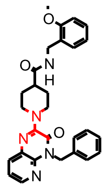 [*]\N=C(\N([*])[*])/C (=[*])[*]	0.224
FCFP_2	1036089772	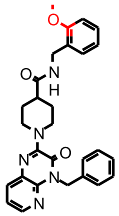 [*]:[c](:[*])OC	0.119
**Top Features for negative contribution**			
**Fingerprint**	**Bit/Smiles**	**Feature Structure**	**Score**
FCFP_2	1	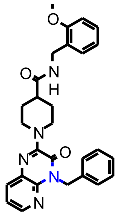 [*]N([*])[*]	-0.306
FCFP_2	0	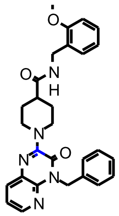 [*]C(=[*])[*]	-0.275
FCFP_2	-1272768868	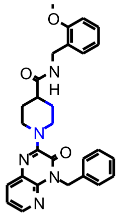 [*]CCN([*])[*]	-0.247

#UNDEFINED

TOPKAT_Fathead_Minnow_LC50

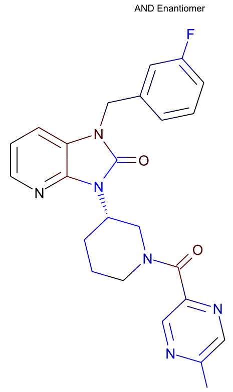

$C_{24}H_{23}FN_6O_2$
Molecular Weight: 446.47682
ALogP: 2.244
Rotatable Bonds: 4
Acceptors: 5
Donors: 0

Model Prediction

Prediction: 0.0124
Unit: g/l
Mahalanobis Distance: 15
Mahalanobis Distance p-value: 6.7e-020

Mahalanobis Distance: The Mahalanobis distance (MD) is a generalization of the Euclidean distance that accounts for correlations among the X properties. It is calculated as the distance to the center of the training data. The larger the MD, the less trustworthy the prediction.
Mahalanobis Distance p-value: The p-value gives the fraction of training data with an MD greater than or equal to the one for the given sample, assuming normally distributed data. The smaller the p-value, the less trustworthy the prediciton. For highly non-normal X properties (e.g., fingerprints), the MD p-value is wildly inaccurate.

Structural Similar Compounds

Name	Rotenone	Rotenone	Diphenylphthalate
Structure	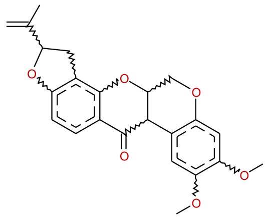	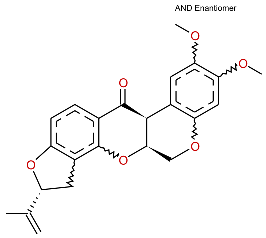	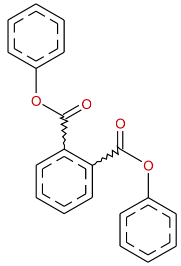
Actual Endpoint (-log C)	7.942	7.87943	6.6
Predicted Endpoint (-log C)	6.1742	6.1742	6.4642
Distance	0.766	0.766	0.831
Reference	ATOCFM Volume 5	DSSTox/EPAFHM	ATOCFM Volume 2

Model Applicability

Unknown features are fingerprint features in the query molecule, but not found or appearing too infrequently in the training set.

1. All properties and OPS components are within expected ranges.

Feature Contribution

Top features for positive contribution			
**Fingerprint**	**Bit/Smiles**	**Feature Structure**	**Score**
FCFP_2	-1549163031	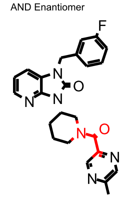 [*]N([*])C(=O)[c](:[*]):[*]	0.078

FCFP_2	332760439	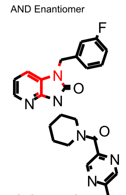 [*][c](:[*]):[c](:[cH]:[*])N=[*]	0.0709
FCFP_2	1872154524	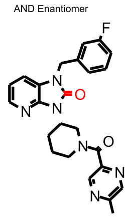 [*]C(=O)[*]	0.0496

Top Features for negative contribution

Fingerprint	Bit/Smiles	Feature Structure	Score
FCFP_2	1	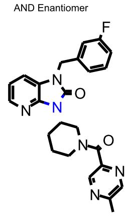 [*]N([*])[*]	-0.306
FCFP_2	0	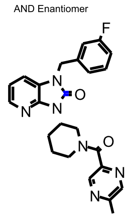 [*]C(=[*])[*]	-0.275
FCFP_2	-1272768868	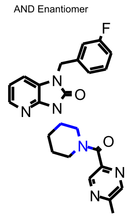 [*]CCN([*])[*]	-0.247

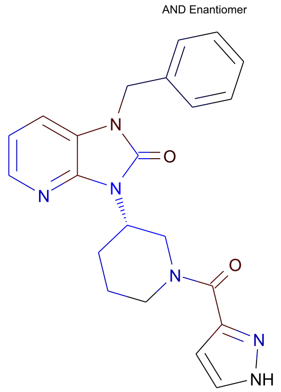

$C_{22}H_{22}N_6O_2$
Molecular Weight: 402.44907
ALogP: 2.501
Rotatable Bonds: 4
Acceptors: 4
Donors: 1

Model Prediction

Prediction: 0.00759
Unit: g/l
Mahalanobis Distance: 13.1
Mahalanobis Distance p-value: 3.86e-013

Mahalanobis Distance: The Mahalanobis distance (MD) is a generalization of the Euclidean distance that accounts for correlations among the X properties. It is calculated as the distance to the center of the training data. The larger the MD, the less trustworthy the prediction.
Mahalanobis Distance p-value: The p-value gives the fraction of training data with an MD greater than or equal to the one for the given sample, assuming normally distributed data. The smaller the p-value, the less trustworthy the prediciton. For highly non-normal X properties (e.g., fingerprints), the MD p-value is wildly inaccurate.

Structural Similar Compounds

Name	Diphenylphthalate	Rotenone	Rotenone
Structure	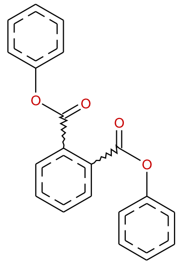	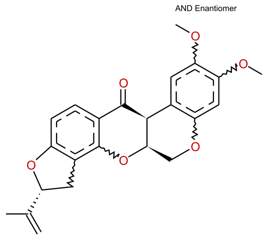	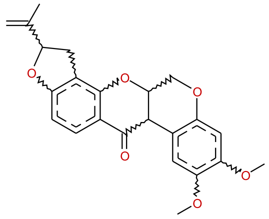
Actual Endpoint (-log C)	6.6	7.87943	7.942
Predicted Endpoint (-log C)	6.4642	6.1742	6.1742
Distance	0.828	0.871	0.871
Reference	ATOCFM Volume 2	DSSTox/EPAFHM	ATOCFM Volume 5

Model Applicability

Unknown features are fingerprint features in the query molecule, but not found or appearing too infrequently in the training set.

1. All properties and OPS components are within expected ranges.
2. Unknown FCFP_2 feature: 1747267175: [*][c]1:[*]:[*]:[nH]:n:1
3. Unknown FCFP_2 feature: 262592487: [*]1:[*]:n:[nH]:[cH]:1

Feature Contribution

Top features for positive contribution			
**Fingerprint**	**Bit/Smiles**	**Feature Structure**	**Score**
FCFP_2	-1549163031	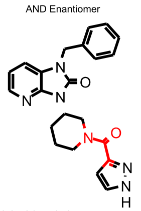 [*]N([*])C(=O)[c](:[*]):[*]	0.078

FCFP_2	332760439	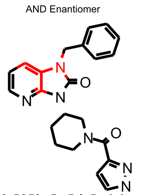 [*][c](:[*]):[c](:[cH]:[*])N=[*]	0.0709
FCFP_2	1872154524	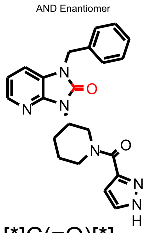 [*]C(=O)[*]	0.0496

Top Features for negative contribution

**Fingerprint**	**Bit/Smiles**	**Feature Structure**	**Score**
FCFP_2	1	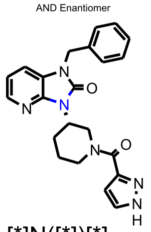 [*]N([*])[*]	-0.306
FCFP_2	0	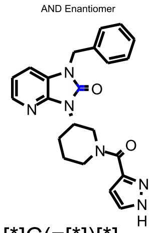 [*]C(=[*])[*]	-0.275
FCFP_2	-1272768868	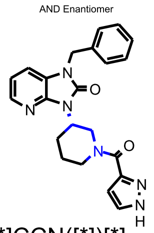 [*]CCN([*])[*]	-0.247

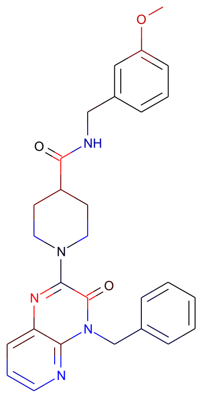

$C_{28}H_{29}N_5O_3$

Molecular Weight: 483.56156

ALogP: 2.942

Rotatable Bonds: 7

Acceptors: 6

Donors: 1

Model Prediction

Prediction: 0.00096

Unit: g/l

Mahalanobis Distance: 14.3

Mahalanobis Distance p-value: 4.05e-017

Mahalanobis Distance: The Mahalanobis distance (MD) is a generalization of the Euclidean distance that accounts for correlations among the X properties. It is calculated as the distance to the center of the training data. The larger the MD, the less trustworthy the prediction.
Mahalanobis Distance p-value: The p-value gives the fraction of training data with an MD greater than or equal to the one for the given sample, assuming normally distributed data. The smaller the p-value, the less trustworthy the prediciton. For highly non-normal X properties (e.g., fingerprints), the MD p-value is wildly inaccurate.

Structural Similar Compounds

Name	Rotenone	Rotenone	Flucythrinate
Structure	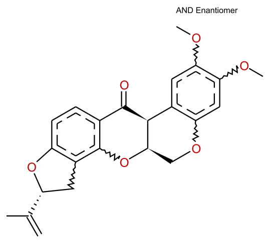	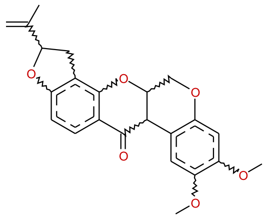	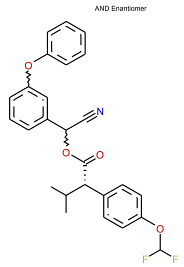
Actual Endpoint (-log C)	7.87943	7.942	9.37572
Predicted Endpoint (-log C)	6.1742	6.1742	8.49266
Distance	0.890	0.890	0.950
Reference	DSSTox/EPAFHM	ATOCFM Volume 5	DSSTox/EPAFHM

Model Applicability

Unknown features are fingerprint features in the query molecule, but not found or appearing too infrequently in the training set.

1. OPS PC9 out of range. Value: -5.4786. Training min, max, SD, explained variance: -4.4045, 4.4278, 1.418, 0.0346.
2. Unknown FCFP_2 feature: 580453787: [*]C(=N[c](:[*]):[*])[*]

Feature Contribution

Top features for positive contribution

Fingerprint	Bit/Smiles	Feature Structure	Score
FCFP_2	-1043339860	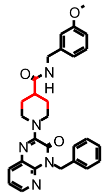 [*]CC(C[*])C(=[*])[*]	0.305

FCFP_2	565998553	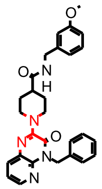 [*]\N=C(\N([*])[*])/C (=[*])[*]	0.224
FCFP_2	1036089772	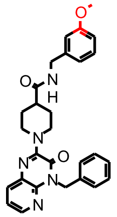 [*]:[c](:[*])OC	0.119

Top Features for negative contribution

Fingerprint	Bit/Smiles	Feature Structure	Score
FCFP_2	1	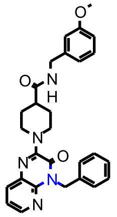 [*]N([*])[*]	-0.306
FCFP_2	0	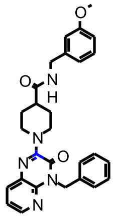 [*]C(=[*])[*]	-0.275
FCFP_2	-1272768868	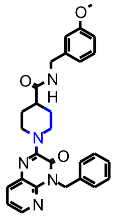 [*]CCN([*])[*]	-0.247

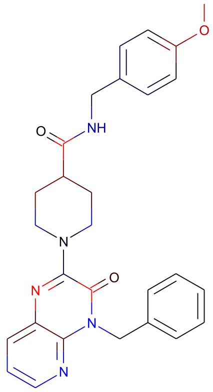

$C_{28}H_{29}N_5O_3$

Molecular Weight: 483.56156

ALogP: 2.942

Rotatable Bonds: 7

Acceptors: 6

Donors: 1

Model Prediction

Prediction: 0.00096

Unit: g/l

Mahalanobis Distance: 14.3

Mahalanobis Distance p-value: 4.05e-017

Mahalanobis Distance: The Mahalanobis distance (MD) is a generalization of the Euclidean distance that accounts for correlations among the X properties. It is calculated as the distance to the center of the training data. The larger the MD, the less trustworthy the prediction.

Mahalanobis Distance p-value: The p-value gives the fraction of training data with an MD greater than or equal to the one for the given sample, assuming normally distributed data. The smaller the p-value, the less trustworthy the prediciton. For highly non-normal X properties (e.g., fingerprints), the MD p-value is wildly inaccurate.

Structural Similar Compounds

Name	Rotenone	Rotenone	Flucythrinate
Structure	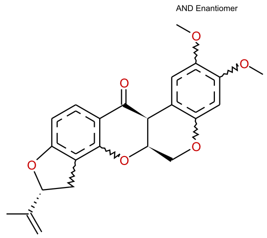	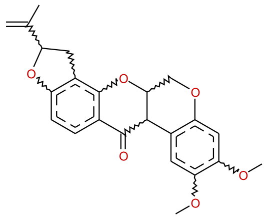	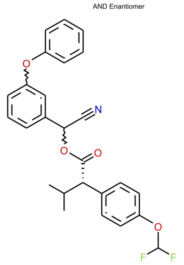
Actual Endpoint (-log C)	7.87943	7.942	9.37572
Predicted Endpoint (-log C)	6.1742	6.1742	8.49266
Distance	0.890	0.890	0.950
Reference	DSSTox/EPAFHM	ATOCFM Volume 5	DSSTox/EPAFHM

Model Applicability

Unknown features are fingerprint features in the query molecule, but not found or appearing too infrequently in the training set.

1. OPS PC9 out of range. Value: -5.4786. Training min, max, SD, explained variance: -4.4045, 4.4278, 1.418, 0.0346.
2. Unknown FCFP_2 feature: 580453787: [*]C(=N[c](:[*]):[*])[*]

Feature Contribution

Top features for positive contribution

Fingerprint	Bit/Smiles	Feature Structure	Score
FCFP_2	-1043339860	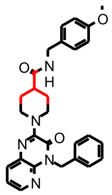 [*]CC(C[*])C(=[*])[*]	0.305

FCFP_2	565998553	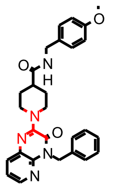 [*]\N=C(\N([*])[*])/C(=[*])[*]	0.224
FCFP_2	1036089772	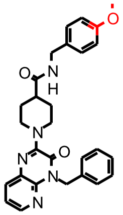 [*]:[c](:[*])OC	0.119
**Top Features for negative contribution**			
**Fingerprint**	**Bit/Smiles**	**Feature Structure**	**Score**
FCFP_2	1	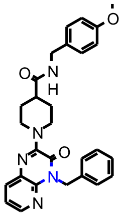 [*]N([*])[*]	-0.306
FCFP_2	0	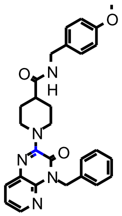 [*]C(=[*])[*]	-0.275
FCFP_2	-1272768868	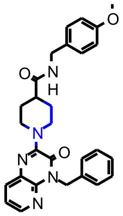 [*]CCN([*])[*]	-0.247

Molecule

TOPKAT_Rat_Inhalational_LC50

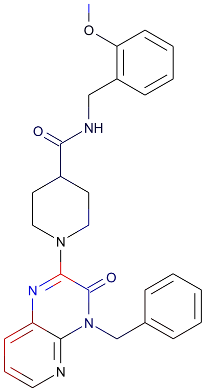

$C_{28}H_{29}N_5O_3$

Molecular Weight: 483.56156

ALogP: 2.942

Rotatable Bonds: 7

Acceptors: 6

Donors: 1

Model Prediction

Prediction: 4.29e+003

Unit: mg/m3/h

Mahalanobis Distance: 12.5

Mahalanobis Distance p-value: 1.27e-007

Mahalanobis Distance: The Mahalanobis distance (MD) is a generalization of the Euclidean distance that accounts for correlations among the X properties. It is calculated as the distance to the center of the training data. The larger the MD, the less trustworthy the prediction.

Mahalanobis Distance p-value: The p-value gives the fraction of training data with an MD greater than or equal to the one for the given sample, assuming normally distributed data. The smaller the p-value, the less trustworthy the prediciton. For highly non-normal X properties (e.g., fingerprints), the MD p-value is wildly inaccurate.

Structural Similar Compounds

Name	Benzoic acid; 4-(((((1;3-dimethyl-5-phenoxy-1H-pyrazol-4-yl)methylene)amino)oxy)methyl)-; 1;1-dimethylethyl ester; (E)-	1H-1;2;4-Triazole-1-ethanamidothioic acid; N-(2;4-dichlorophenyl)-;	Propanoic acid; 2-(4-((6-chloro-2-quinoxalinyl)oxy)phenoxy)-; 2-(((1-methylethylidene)amino)oxy)ethyl ester; (R)-
Structure	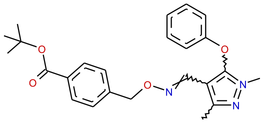	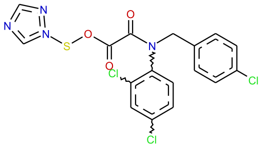	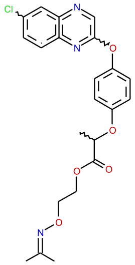
Actual Endpoint (-log C)	2.5042	2.0499	1.6473
Predicted Endpoint (-log C)	2.56205	2.8772	1.81634
Distance	0.729	0.733	0.767
Reference	NNGADV Nippon Noyaku Gakkaishi. Journal of the Pesticide Science Society of Japan. (Nippon Noyaku Gakkai; 1-43-11; Komagome; Toshima-ku; Tokyo 170; Japan) V.1- 1976- Volume(issue)/page/year: 17;S261;199	NNGADV Nippon Noyaku Gakkaishi. Journal of the Pesticide Science Society of Japan. (Nippon Noyaku Gakkai; 1-43-11; Komagome; Toshima-ku; Tokyo 170; Japan) V.1- 1976- Volume(issue)/page/year: 20;373;1995	PEMNDP Pesticide Manual. (The British Crop Protection Council; 20 Bridport R d.; Thornton Heath CR4 7QG; UK) V.1- 1968- Volume(issue)/page/year: 9;718;1 991

Model Applicability

Unknown features are fingerprint features in the query molecule, but not found or appearing too infrequently in the training set.

1. All properties and OPS components are within expected ranges.
2. Unknown ECFP_2 feature: 671679640: [*]\N=C(\N([*])[*])/C(=[*])[*]
3. Unknown ECFP_2 feature: -509950643: [*]N([*])[c](:n:[*]):[c]([*]):[*]
4. Unknown ECFP_2 feature: 1951894094: [*]CN(C[*])C(=[*])[*]
5. Unknown ECFP_2 feature: -597295171: [*][c](:[*]):[c](:[cH]:[*])N=[*]
6. Unknown ECFP_2 feature: -81134287: [*]NC(=O)C([*])[*]
7. Unknown ECFP_2 feature: -857146788: [*]CC(C[*])C(=[*])[*]
8. Unknown ECFP_2 feature: 769925792: [*]NC[c](:[*]):[*]

Feature Contribution

Top features for positive contribution

Fingerprint	Bit/Smiles	Feature Structure	Score
ECFP_2	642810091	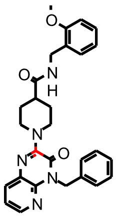 [*]C(=[*])[*]	0.214
ECFP_2	1996767644	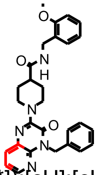 [*][c](:[*]):[cH]:[cH]:[*]	0.127
ECFP_2	-1074141656	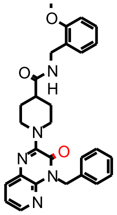 [*]=O	0.0468

Top Features for negative contribution

Fingerprint	Bit/Smiles	Feature Structure	Score
ECFP_2	734603939	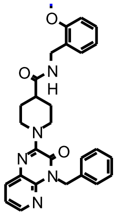 [*]C	-0.302

ECFP_2	655739385	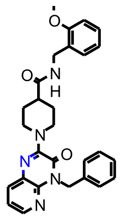 [*]N=[*]	-0.217
ECFP_2	2106656448	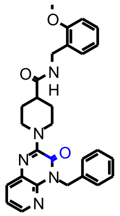 [*]C(=O)[*]	-0.151

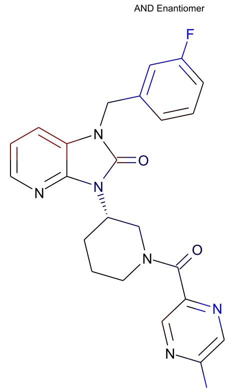

$C_{24}H_{23}FN_6O_2$

Molecular Weight: 446.47682

ALogP: 2.244

Rotatable Bonds: 4

Acceptors: 5

Donors: 0

Model Prediction

Prediction: 2.17e+004

Unit: mg/m3/h

Mahalanobis Distance: 13.7

Mahalanobis Distance p-value: 9.39e-011

Mahalanobis Distance: The Mahalanobis distance (MD) is a generalization of the Euclidean distance that accounts for correlations among the X properties. It is calculated as the distance to the center of the training data. The larger the MD, the less trustworthy the prediction.
Mahalanobis Distance p-value: The p-value gives the fraction of training data with an MD greater than or equal to the one for the given sample, assuming normally distributed data. The smaller the p-value, the less trustworthy the prediciton. For highly non-normal X properties (e.g., fingerprints), the MD p-value is wildly inaccurate.

Structural Similar Compounds

Name	Maleimide; N;N'-(methylenedi-p-phenylene)di-	1H-1;2;4-Triazole; 1-((2-(2-chloro-4-(4-chlorophenoxy)phenyl)-4-met hyl-1;3-dioxolan-2-yl)methyl)-	1;3-Indandione; 2-diphenylacetyl-
Structure	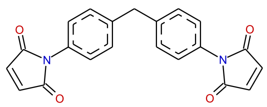	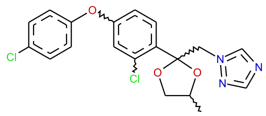	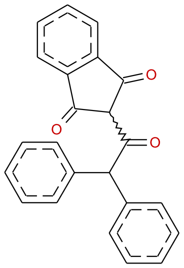
Actual Endpoint (-log C)	2.4082	3.3535	1.6289
Predicted Endpoint (-log C)	2.40256	1.62735	1.79293
Distance	0.709	0.709	0.768
Reference	EPASR* United States Environmental Protection Agency; Office of Pesticides an d Toxic Substances. (U.S. Environmental Protection Agency; 401 M St.; SW; Washin gton; DC 20460) History unknown. Volume(issue)/pag	PEMNDP Pesticide Manual. (The British Crop Protection Council; 20 Bridport R d.; Thornton Heath CR4 7QG; UK) V.1- 1968- Volume(issue)/page/year: 9;277;1 991	PEMNDP Pesticide Manual. (The British Crop Protection Council; 20 Bridport R d.; Thornton Heath CR4 7QG; UK) V.1- 1968- Volume(issue)/page/year: 9;310;1 991

Model Applicability

Unknown features are fingerprint features in the query molecule, but not found or appearing too infrequently in the training set.

1. All properties and OPS components are within expected ranges.
2. Unknown ECFP_2 feature: -509950643: [*]N([*])[c](:n:[*]):[c]([*]):[*]
3. Unknown ECFP_2 feature: 2077298510: [*]N([*])C(=O)[c](:[*]):[*]
4. Unknown ECFP_2 feature: 1413420509: [*]C(=[*])[c](:[cH]:[*]):n:[*]

Feature Contribution

Top features for positive contribution

Fingerprint	Bit/Smiles	Feature Structure	Score

ECFP_2	642810091	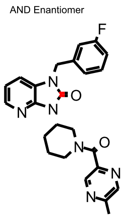 [*]C(=[*])[*]	0.214
ECFP_2	1996767644	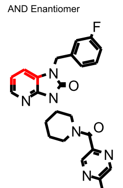 [*][c](:[*]):[cH]:[cH]:[*]	0.127
ECFP_2	-1074141656	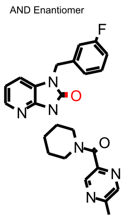 [*]=O	0.0468
**Top Features for negative contribution**			
**Fingerprint**	**Bit/Smiles**	**Feature Structure**	**Score**
ECFP_2	734603939	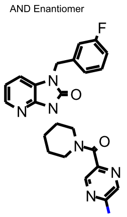 [*]C	-0.302
ECFP_2	-1046436026	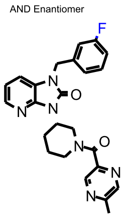 [*]F	-0.26

ECFP_2	655739385	AND Enantiomer 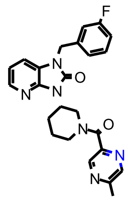 [*]N=[*]	-0.217

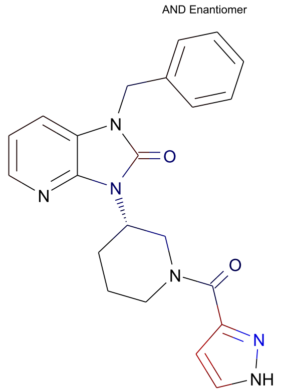

$C_{22}H_{22}N_6O_2$
Molecular Weight: 402.44907
ALogP: 2.501
Rotatable Bonds: 4
Acceptors: 4
Donors: 1

Model Prediction

Prediction: 3.78e+003
Unit: mg/m3/h
Mahalanobis Distance: 10.9
Mahalanobis Distance p-value: 0.000556

Mahalanobis Distance: The Mahalanobis distance (MD) is a generalization of the Euclidean distance that accounts for correlations among the X properties. It is calculated as the distance to the center of the training data. The larger the MD, the less trustworthy the prediction.
Mahalanobis Distance p-value: The p-value gives the fraction of training data with an MD greater than or equal to the one for the given sample, assuming normally distributed data. The smaller the p-value, the less trustworthy the prediciton. For highly non-normal X properties (e.g., fingerprints), the MD p-value is wildly inaccurate.

Structural Similar Compounds

Name	4-Pyridinecarboxamide; N-(4-chloro-2-(hydroxyphenylmethyl)phenyl)-	Maleimide; N;N'-(methylenedi-p-phenylene)di-	1;3-Indandione; 2-diphenylacetyl-
Structure	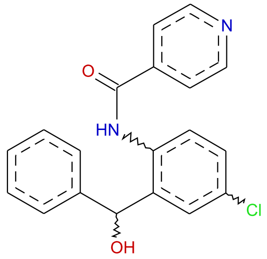	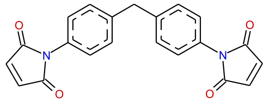	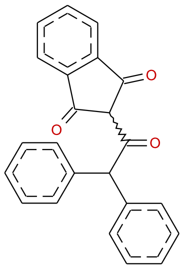
Actual Endpoint (-log C)	2.268	2.4082	1.6289
Predicted Endpoint (-log C)	1.54125	2.40256	1.79293
Distance	0.695	0.720	0.745
Reference	NNGADV Nippon Noyaku Gakkaishi. Journal of the Pesticide Science Society of Japan. (Nippon Noyaku Gakkai; 1-43-11; Komagome; Toshima-ku; Tokyo 170; Japan) V.1- 1976- Volume(issue)/page/year: 13;391;1988	EPASR* United States Environmental Protection Agency; Office of Pesticides an d Toxic Substances. (U.S. Environmental Protection Agency; 401 M St.; SW; Washin gton; DC 20460) History unknown. Volume(issue)/pag	PEMNDP Pesticide Manual. (The British Crop Protection Council; 20 Bridport R d.; Thornton Heath CR4 7QG; UK) V.1- 1968- Volume(issue)/page/year: 9;310;1 991

Model Applicability

Unknown features are fingerprint features in the query molecule, but not found or appearing too infrequently in the training set.

1. All properties and OPS components are within expected ranges.
2. Unknown ECFP_2 feature: -509950643: [*]N([*])[c](:n:[*]):[c]([*]):[*]
3. Unknown ECFP_2 feature: 2077298510: [*]N([*])C(=O)[c](:[*]):[*]
4. Unknown ECFP_2 feature: 1413420509: [*]C(=[*])[c](:[cH]:[*]):n:[*]
5. Unknown ECFP_2 feature: -954588747: [*]1:[*]:n:[nH]:[cH]:1
6. Unknown ECFP_2 feature: 1998023064: [*]1:[*]:[cH]:[cH]:[nH]:1

Feature Contribution

Top features for positive contribution			
Fingerprint	Bit/Smiles	Feature Structure	Score

ECFP_2	642810091	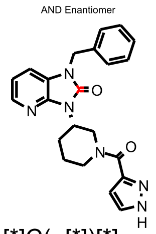 [*]C(=[*])[*]	0.214
ECFP_2	1996767644	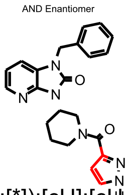 [*][c](:[*]):[cH]:[cH]:[*]	0.127
ECFP_2	-1074141656	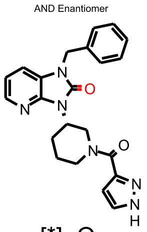 [*]=O	0.0468

Top Features for negative contribution

Fingerprint	Bit/Smiles	Feature Structure	Score
ECFP_2	655739385	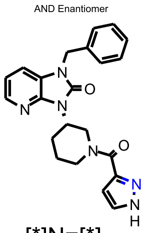 [*]N=[*]	-0.217
ECFP_2	2106656448	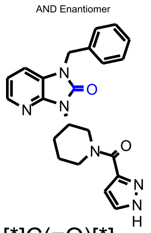 [*]C(=O)[*]	-0.151

ECFP_2	-992506539	AND Enantiomer 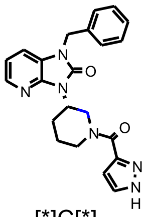 [*]C[*]	-0.119

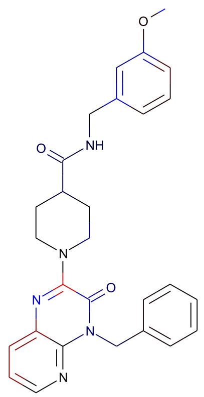

$C_{28}H_{29}N_5O_3$

Molecular Weight: 483.56156

ALogP: 2.942

Rotatable Bonds: 7

Acceptors: 6

Donors: 1

Model Prediction

Prediction: 6.18e+003

Unit: mg/m3/h

Mahalanobis Distance: 13.4

Mahalanobis Distance p-value: 7.29e-010

Mahalanobis Distance: The Mahalanobis distance (MD) is a generalization of the Euclidean distance that accounts for correlations among the X properties. It is calculated as the distance to the center of the training data. The larger the MD, the less trustworthy the prediction.
Mahalanobis Distance p-value: The p-value gives the fraction of training data with an MD greater than or equal to the one for the given sample, assuming normally distributed data. The smaller the p-value, the less trustworthy the prediciton. For highly non-normal X properties (e.g., fingerprints), the MD p-value is wildly inaccurate.

Structural Similar Compounds

Name	Benzoic acid; 4-(((((1;3-dimethyl-5-phenoxy-1H-pyrazol-4-yl)methylene)amino)oxy)methyl)-; 1;1-dimethylethyl ester; (E)-	1H-1;2;4-Triazole-1-ethanamidothioic acid; N-(2;4-dichlorophenyl)-;	Propanoic acid; 2-(4-((6-chloro-2-quinoxalinyl)oxy)phenoxy)-; 2-(((1-methylethylidene)amino) oxy)ethyl ester; (R)-
Structure	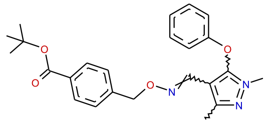	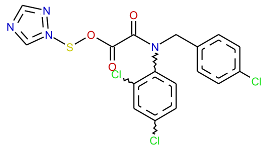	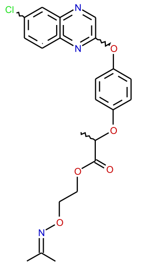
Actual Endpoint (-log C)	2.5042	2.0499	1.6473
Predicted Endpoint (-log C)	2.56205	2.8772	1.81634
Distance	0.718	0.722	0.746
Reference	NNGADV Nippon Noyaku Gakkaishi. Journal of the Pesticide Science Society of Japan. (Nippon Noyaku Gakkai; 1-43-11; Komagome; Toshima-ku; Tokyo 170; Japan) V.1- 1976- Volume(issue)/page/year: 17;S261;199	NNGADV Nippon Noyaku Gakkaishi. Journal of the Pesticide Science Society of Japan. (Nippon Noyaku Gakkai; 1-43-11; Komagome; Toshima-ku; Tokyo 170; Japan) V.1- 1976- Volume(issue)/page/year: 20;373;1995	PEMNDP Pesticide Manual. (The British Crop Protection Council; 20 Bridport R d.; Thornton Heath CR4 7QG; UK) V.1- 1968- Volume(issue)/page/year: 9;718;1 991

Model Applicability

Unknown features are fingerprint features in the query molecule, but not found or appearing too infrequently in the training set.

1. All properties and OPS components are within expected ranges.
2. Unknown ECFP_2 feature: 671679640: [*]\N=C(\N([*])[*])/C(=[*])[*]
3. Unknown ECFP_2 feature: -509950643: [*]N([*])[c](:n:[*]):[c]([*]):[*]
4. Unknown ECFP_2 feature: 1951894094: [*]CN(C[*])C(=[*])[*]
5. Unknown ECFP_2 feature: -597295171: [*][c](:[*]):[c](:[cH]:[*])N=[*]
6. Unknown ECFP_2 feature: -81134287: [*]NC(=O)C([*])[*]
7. Unknown ECFP_2 feature: -857146788: [*]CC(C[*])C(=[*])[*]
8. Unknown ECFP_2 feature: 769925792: [*]NC[c](:[*]):[*]

Feature Contribution

Top features for positive contribution

Fingerprint	Bit/Smiles	Feature Structure	Score
ECFP_2	642810091	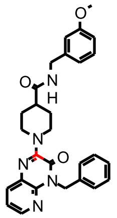 [*]C(=[*])[*]	0.214
ECFP_2	1996767644	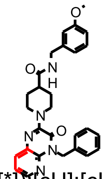 [*][c](:[*]):[cH]:[cH]:[*]	0.127
ECFP_2	-176455838	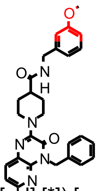 [*]O[c](:[cH]:[*]):[c H]:[*]	0.047

Top Features for negative contribution

Fingerprint	Bit/Smiles	Feature Structure	Score
ECFP_2	734603939	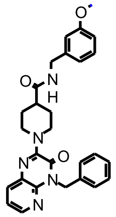 [*]C	-0.302

ECFP_2	655739385	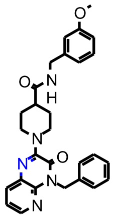 [*]N=[*]	-0.217
ECFP_2	-786013480	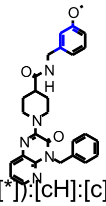 [*][c](:[*]):[cH]:[c]([*]):[*]	-0.206

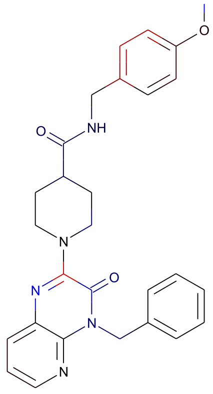

$C_{28}H_{29}N_5O_3$

Molecular Weight: 483.56156

ALogP: 2.942

Rotatable Bonds: 7

Acceptors: 6

Donors: 1

Model Prediction

Prediction: 3.85e+003

Unit: mg/m3/h

Mahalanobis Distance: 13

Mahalanobis Distance p-value: 9.26e-009

Mahalanobis Distance: The Mahalanobis distance (MD) is a generalization of the Euclidean distance that accounts for correlations among the X properties. It is calculated as the distance to the center of the training data. The larger the MD, the less trustworthy the prediction.
Mahalanobis Distance p-value: The p-value gives the fraction of training data with an MD greater than or equal to the one for the given sample, assuming normally distributed data. The smaller the p-value, the less trustworthy the prediciton. For highly non-normal X properties (e.g., fingerprints), the MD p-value is wildly inaccurate.

Structural Similar Compounds

Name	Benzoic acid; 4-(((((1;3-dimethyl-5-phenoxy-1H-pyrazol-4-yl)methyle ne)amino)oxy)methyl)-; 1;1-dimethylethyl ester; (E)-	1H-1;2;4-Triazole-1-ethanamidothioic acid; N-(2;4-dichlorophenyl)-;	Propanoic acid; 2-(4-((6-chloro-2-quinoxalinyl)oxy)phenoxy)-; 2-(((1-methylethylidene)amino) oxy)ethyl ester; (R)-
Structure	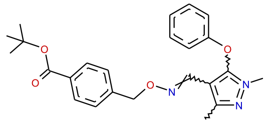	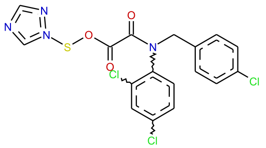	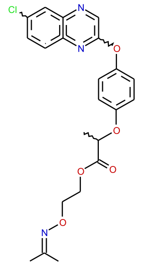
Actual Endpoint (-log C)	2.5042	2.0499	1.6473
Predicted Endpoint (-log C)	2.56205	2.8772	1.81634
Distance	0.716	0.731	0.754
Reference	NNGADV Nippon Noyaku Gakkaishi. Journal of the Pesticide Science Society of Japan. (Nippon Noyaku Gakkai; 1-43-11; Komagome; Toshima-ku; Tokyo 170; Japan) V.1- 1976- Volume(issue)/page/year: 17;S261;199	NNGADV Nippon Noyaku Gakkaishi. Journal of the Pesticide Science Society of Japan. (Nippon Noyaku Gakkai; 1-43-11; Komagome; Toshima-ku; Tokyo 170; Japan) V.1- 1976- Volume(issue)/page/year: 20;373;1995	PEMNDP Pesticide Manual. (The British Crop Protection Council; 20 Bridport R d.; Thornton Heath CR4 7QG; UK) V.1- 1968- Volume(issue)/page/year: 9;718;1 991

Model Applicability

Unknown features are fingerprint features in the query molecule, but not found or appearing too infrequently in the training set.

1. All properties and OPS components are within expected ranges.
2. Unknown ECFP_2 feature: 671679640: [*]\N=C(\N([*])[*])/C(=[*])[*]
3. Unknown ECFP_2 feature: -509950643: [*]N([*])[c](:n:[*]):[c]([*]):[*]
4. Unknown ECFP_2 feature: 1951894094: [*]CN(C[*])C(=[*])[*]
5. Unknown ECFP_2 feature: -597295171: [*][c](:[*]):[c](:[cH]:[*])N=[*]
6. Unknown ECFP_2 feature: -81134287: [*]NC(=O)C([*])[*]
7. Unknown ECFP_2 feature: -857146788: [*]CC(C[*])C(=[*])[*]
8. Unknown ECFP_2 feature: 769925792: [*]NC[c](:[*]):[*]

Feature Contribution

Top features for positive contribution

Fingerprint	Bit/Smiles	Feature Structure	Score
ECFP_2	642810091	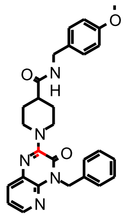 [*]C(=[*])[*]	0.214
ECFP_2	1996767644	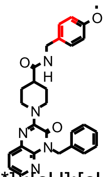 [*][c](:[*]):[cH]:[cH]:[*]	0.127
ECFP_2	-176455838	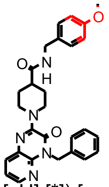 [*]O[c](:[cH]:[*]):[cH]:[*]	0.047

Top Features for negative contribution

Fingerprint	Bit/Smiles	Feature Structure	Score
ECFP_2	734603939	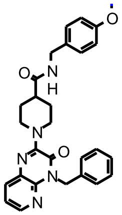 [*]C	-0.302

ECFP_2	655739385	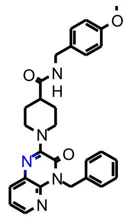<br>[*]N=[*]	-0.217
ECFP_2	2106656448	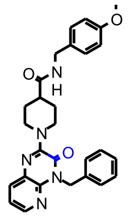<br>[*]C(=O)[*]	-0.151

Molecule

TOPKAT_Rat_Maximum_Tolerated_Dose_Feed

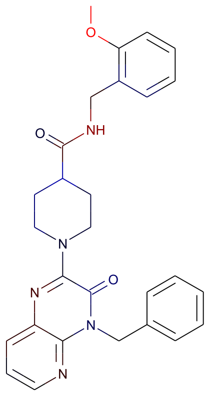

$C_{28}H_{29}N_5O_3$

Molecular Weight: 483.56156

ALogP: 2.942

Rotatable Bonds: 7

Acceptors: 6

Donors: 1

Structural Similar Compounds

Name	ROTENONE	3,3'-DIMETHOXYBENZIDINE-4,4'-DIISOCYANATE	PYRILAMINE
Structure	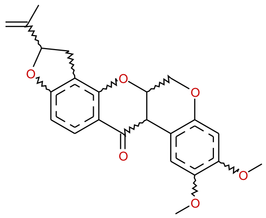	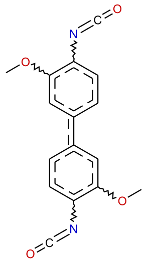	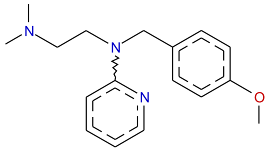
Actual Endpoint (-log C)	5.06769	2.17504	3.32511
Predicted Endpoint (-log C)	4.11907	3.78717	3.65163
Distance	0.746	0.754	0.798
Reference	NCI/NTP TR-320	NCI/NTP TR-128	NCI/NTP Report 10, Nov. 1987

Model Prediction

Prediction: 0.0247

Unit: g/kg_body_weight

Mahalanobis Distance: 11.2

Mahalanobis Distance p-value: 3.5e-007

Mahalanobis Distance: The Mahalanobis distance (MD) is a generalization of the Euclidean distance that accounts for correlations among the X properties. It is calculated as the distance to the center of the training data. The larger the MD, the less trustworthy the prediction.
Mahalanobis Distance p-value: The p-value gives the fraction of training data with an MD greater than or equal to the one for the given sample, assuming normally distributed data. The smaller the p-value, the less trustworthy the prediciton. For highly non-normal X properties (e.g., fingerprints), the MD p-value is wildly inaccurate.

Model Applicability

Unknown features are fingerprint features in the query molecule, but not found or appearing too infrequently in the training set.

1. OPS PC9 out of range. Value: 5.3652. Training min, max, SD, explained variance: -2.8548, 3.3954, 1.263, 0.0360.

Feature Contribution

Top features for positive contribution

Fingerprint	Bit/Smiles	Feature Structure	Score
FCFP_2	136627117	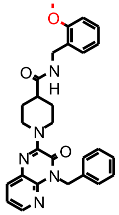 [*]OC	0.173

FCFP_2	-885550502	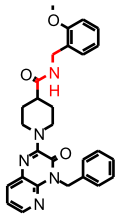 [*]CNC(=[*])[*]	0.115
FCFP_2	1036089772	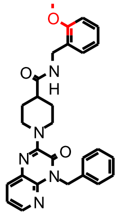 [*]:[c](:[*])OC	0.0749

Top Features for negative contribution

Fingerprint	Bit/Smiles	Feature Structure	Score
FCFP_2	-1272798659	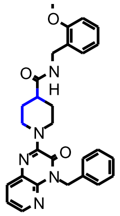 [*]CCC([*])[*]	-0.111
FCFP_2	1872154524	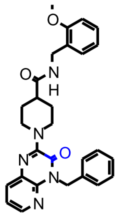 [*]C(=O)[*]	-0.105
FCFP_2	203677720	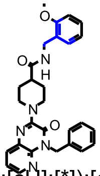 [*]C[c](:[cH]:[*]):[c]([*]):[*]	-0.0829

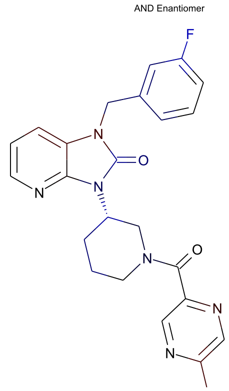

$C_{24}H_{23}FN_6O_2$
Molecular Weight: 446.47682
ALogP: 2.244
Rotatable Bonds: 4
Acceptors: 5
Donors: 0

Structural Similar Compounds

Name	ROTENONE	3,3'-DIMETHOXYBENZIDINE-4,4'-DIISOCYANATE	D&C YELLOW NO. 11
Structure	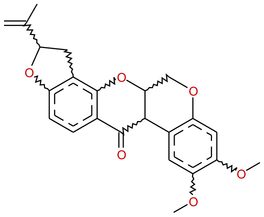	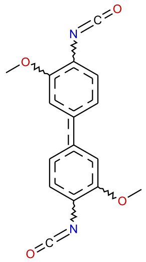	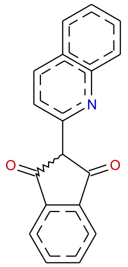
Actual Endpoint (-log C)	5.06769	2.17504	4.03869
Predicted Endpoint (-log C)	4.11907	3.78717	3.54593
Distance	0.668	0.745	0.756
Reference	NCI/NTP TR-320	NCI/NTP TR-128	NCI/NTP TR-463

Model Prediction

Prediction: 0.0338
Unit: g/kg_body_weight
Mahalanobis Distance: 11.5
Mahalanobis Distance p-value: 1.13e-007

Mahalanobis Distance: The Mahalanobis distance (MD) is a generalization of the Euclidean distance that accounts for correlations among the X properties. It is calculated as the distance to the center of the training data. The larger the MD, the less trustworthy the prediction.
Mahalanobis Distance p-value: The p-value gives the fraction of training data with an MD greater than or equal to the one for the given sample, assuming normally distributed data. The smaller the p-value, the less trustworthy the prediciton. For highly non-normal X properties (e.g., fingerprints), the MD p-value is wildly inaccurate.

Model Applicability

Unknown features are fingerprint features in the query molecule, but not found or appearing too infrequently in the training set.

1. All properties and OPS components are within expected ranges.

Feature Contribution

Top features for positive contribution

Fingerprint	Bit/Smiles	Feature Structure	Score
FCFP_2	136120670	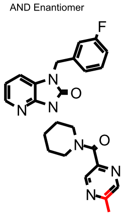 [*]:[c](:[*])C	0.064

FCFP_2	332760439	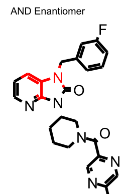 [*][c](:[*]):[c](:[cH]:[*])N=[*]	0.0611
FCFP_2	17	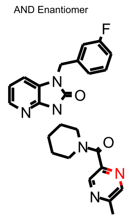 [*]:n:[*]	0.0441

Top Features for negative contribution

Fingerprint	Bit/Smiles	Feature Structure	Score
FCFP_2	71476542	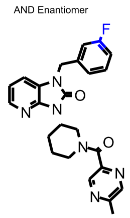 [*]:[c](:[*])F	-0.134
FCFP_2	-1272798659	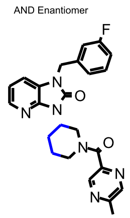 [*]CCC([*])[*]	-0.111
FCFP_2	1872154524	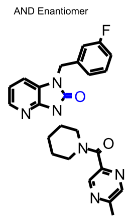 [*]C(=O)[*]	-0.105

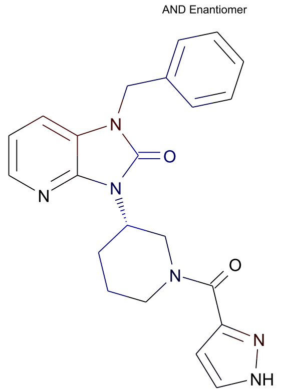

$C_{22}H_{22}N_6O_2$

Molecular Weight: 402.44907

ALogP: 2.501

Rotatable Bonds: 4

Acceptors: 4

Donors: 1

Model Prediction

Prediction: 0.0568

Unit: g/kg_body_weight

Mahalanobis Distance: 9.94

Mahalanobis Distance p-value: 5.57e-005

Mahalanobis Distance: The Mahalanobis distance (MD) is a generalization of the Euclidean distance that accounts for correlations among the X properties. It is calculated as the distance to the center of the training data. The larger the MD, the less trustworthy the prediction.
Mahalanobis Distance p-value: The p-value gives the fraction of training data with an MD greater than or equal to the one for the given sample, assuming normally distributed data. The smaller the p-value, the less trustworthy the prediciton. For highly non-normal X properties (e.g., fingerprints), the MD p-value is wildly inaccurate.

Structural Similar Compounds

Name	PHENOLPHTHALEIN	C.I.PIGMENT RED 3	D&C YELLOW NO. 11
Structure	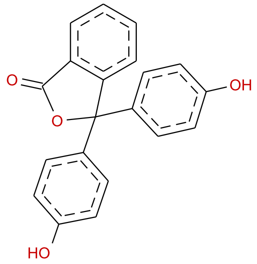	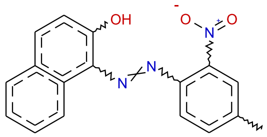	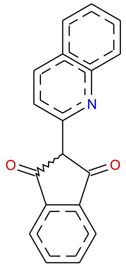
Actual Endpoint (-log C)	2.20184	2.65635	4.03869
Predicted Endpoint (-log C)	2.8857	2.97957	3.54593
Distance	0.661	0.713	0.713
Reference	NCI/NTP TR-465	NCI/NTP TR-407	NCI/NTP TR-463

Model Applicability

Unknown features are fingerprint features in the query molecule, but not found or appearing too infrequently in the training set.

1. All properties and OPS components are within expected ranges.
2. Unknown FCFP_2 feature: 1747267175: [*][c]1:[*]:[*]:[nH]:n:1

Feature Contribution

Top features for positive contribution			
**Fingerprint**	**Bit/Smiles**	**Feature Structure**	**Score**
FCFP_2	332760439	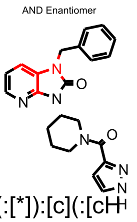 [*][c](:[*]):[c](:[cH]:[*])N=[*]	0.0611

FCFP_2	17	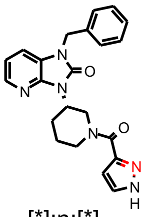 [*]:n:[*]	0.0441
**Top Features for negative contribution**			
**Fingerprint**	**Bit/Smiles**	**Feature Structure**	**Score**
FCFP_2	-1272798659	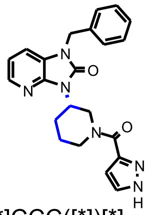 [*]CCC([*])[*]	-0.111
FCFP_2	1872154524	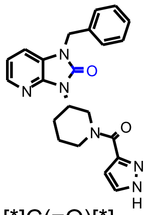 [*]C(=O)[*]	-0.105
FCFP_2	203677720	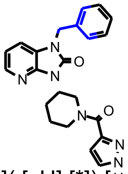 [*]C[c](:[cH]:[*]):[c]([*]):[*]	-0.0829

#UNDEFINED

TOPKAT_Rat_Maximum_Tolerated_Dose_Feed

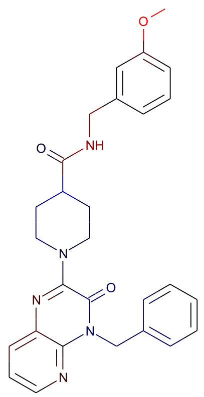

$C_{28}H_{29}N_5O_3$
Molecular Weight: 483.56156
ALogP: 2.942
Rotatable Bonds: 7
Acceptors: 6
Donors: 1

Structural Similar Compounds

Name	ROTENONE	3,3'-DIMETHOXYBENZIDINE-4,4'-DIISOCYANATE	PYRILAMINE
Structure	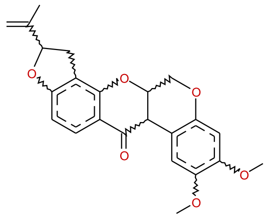	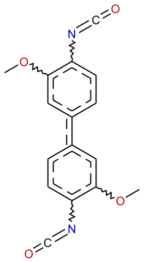	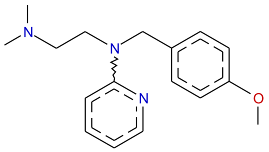
Actual Endpoint (-log C)	5.06769	2.17504	3.32511
Predicted Endpoint (-log C)	4.11907	3.78717	3.65163
Distance	0.746	0.754	0.798
Reference	NCI/NTP TR-320	NCI/NTP TR-128	NCI/NTP Report 10, Nov. 1987

Model Prediction

Prediction: 0.0247
Unit: g/kg_body_weight
Mahalanobis Distance: 11.2
Mahalanobis Distance p-value: 3.5e-007

Mahalanobis Distance: The Mahalanobis distance (MD) is a generalization of the Euclidean distance that accounts for correlations among the X properties. It is calculated as the distance to the center of the training data. The larger the MD, the less trustworthy the prediction.
Mahalanobis Distance p-value: The p-value gives the fraction of training data with an MD greater than or equal to the one for the given sample, assuming normally distributed data. The smaller the p-value, the less trustworthy the prediciton. For highly non-normal X properties (e.g., fingerprints), the MD p-value is wildly inaccurate.

Model Applicability

Unknown features are fingerprint features in the query molecule, but not found or appearing too infrequently in the training set.

1. OPS PC9 out of range. Value: 5.3652. Training min, max, SD, explained variance: -2.8548, 3.3954, 1.263, 0.0360.

Feature Contribution

Top features for positive contribution

Fingerprint	Bit/Smiles	Feature Structure	Score
FCFP_2	136627117	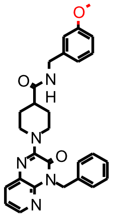 [*]OC	0.173

FCFP_2	-885550502	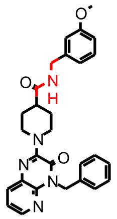 [*]CNC(=[*])[*]	0.115
FCFP_2	1036089772	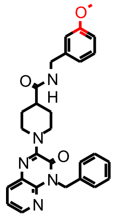 [*]:[c](:[*])OC	0.0749

Top Features for negative contribution

Fingerprint	Bit/Smiles	Feature Structure	Score
FCFP_2	-1272798659	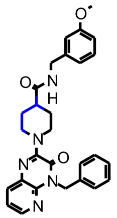 [*]CCC([*])[*]	-0.111
FCFP_2	1872154524	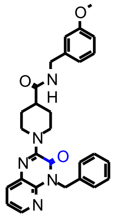 [*]C(=O)[*]	-0.105
FCFP_2	203677720	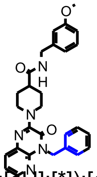 [*]C[c](:[cH]:[*]):[c]([*]):[*]	-0.0829

#UNDEFINED

TOPKAT_Rat_Maximum_Tolerated_Dose_Feed

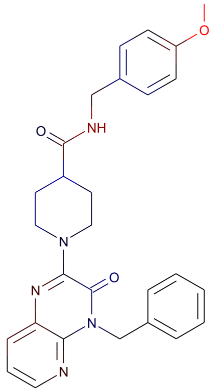

$C_{28}H_{29}N_5O_3$

Molecular Weight: 483.56156

ALogP: 2.942

Rotatable Bonds: 7

Acceptors: 6

Donors: 1

Structural Similar Compounds

Name	ROTENONE	3,3'-DIMETHOXYBENZIDINE-4,4'-DIISOCYANATE	PYRILAMINE
Structure	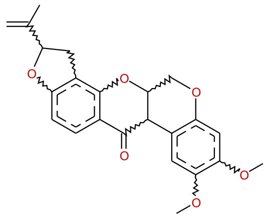	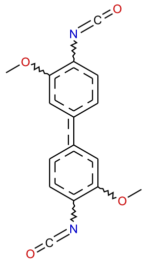	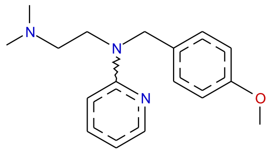
Actual Endpoint (-log C)	5.06769	2.17504	3.32511
Predicted Endpoint (-log C)	4.11907	3.78717	3.65163
Distance	0.746	0.754	0.798
Reference	NCI/NTP TR-320	NCI/NTP TR-128	NCI/NTP Report 10, Nov. 1987

Model Prediction

Prediction: 0.0247

Unit: g/kg_body_weight

Mahalanobis Distance: 11.2

Mahalanobis Distance p-value: 3.5e-007

Mahalanobis Distance: The Mahalanobis distance (MD) is a generalization of the Euclidean distance that accounts for correlations among the X properties. It is calculated as the distance to the center of the training data. The larger the MD, the less trustworthy the prediction.
Mahalanobis Distance p-value: The p-value gives the fraction of training data with an MD greater than or equal to the one for the given sample, assuming normally distributed data. The smaller the p-value, the less trustworthy the prediciton. For highly non-normal X properties (e.g., fingerprints), the MD p-value is wildly inaccurate.

Model Applicability

Unknown features are fingerprint features in the query molecule, but not found or appearing too infrequently in the training set.

1. OPS PC9 out of range. Value: 5.3652. Training min, max, SD, explained variance: -2.8548, 3.3954, 1.263, 0.0360.

Feature Contribution

Top features for positive contribution

Fingerprint	Bit/Smiles	Feature Structure	Score
FCFP_2	136627117	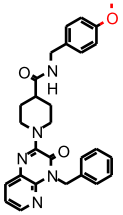 [*]OC	0.173

FCFP_2	-885550502	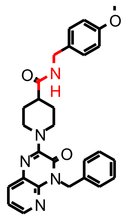 [*]CNC(=[*])[*]	0.115
FCFP_2	1036089772	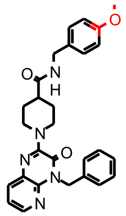 [*]:[c](:[*])OC	0.0749

Top Features for negative contribution

Fingerprint	Bit/Smiles	Feature Structure	Score
FCFP_2	-1272798659	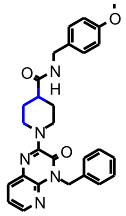 [*]CCC([*])[*]	-0.111
FCFP_2	1872154524	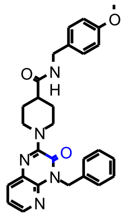 [*]C(=O)[*]	-0.105
FCFP_2	203677720	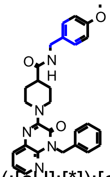 [*]C[c](:[cH]:[*]):[c<br>]([*]):[*]	-0.0829

Molecule

TOPKAT_Rat_Maximum_Tolerated_Dose_Gavage

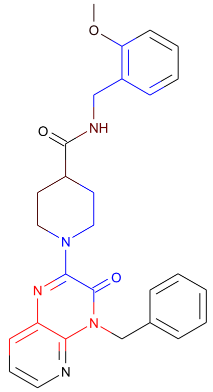

$C_{28}H_{29}N_5O_3$

Molecular Weight: 483.56156

ALogP: 2.942

Rotatable Bonds: 7

Acceptors: 6

Donors: 1

Model Prediction

Prediction: 0.00519

Unit: g/kg_body_weight

Mahalanobis Distance: 13.6

Mahalanobis Distance p-value: 4.88e-011

Mahalanobis Distance: The Mahalanobis distance (MD) is a generalization of the Euclidean distance that accounts for correlations among the X properties. It is calculated as the distance to the center of the training data. The larger the MD, the less trustworthy the prediction.
Mahalanobis Distance p-value: The p-value gives the fraction of training data with an MD greater than or equal to the one for the given sample, assuming normally distributed data. The smaller the p-value, the less trustworthy the prediciton. For highly non-normal X properties (e.g., fingerprints), the MD p-value is wildly inaccurate.

Structural Similar Compounds

Name	OCHRATOXIN	PENICILLIN VK	PHENYLBUTAZONE
Structure	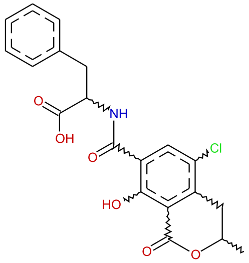	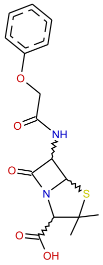	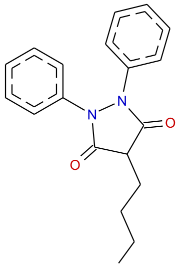
Actual Endpoint (-log C)	6.28396	2.54455	3.48909
Predicted Endpoint (-log C)	5.12358	3.9702	3.17333
Distance	1.023	1.044	1.073
Reference	NCI/NTP TR-358	NCI/NTP TR-336	NCI/NTP TR-367

Model Applicability

Unknown features are fingerprint features in the query molecule, but not found or appearing too infrequently in the training set.

1. Molecular_Weight out of range. Value: 483.56. Training min, max, mean, SD: 68.074, 434.63, 171.13, 85.06.
2. Num_AromaticRings out of range. Value: 3. Training min, max, mean, SD: 0, 2, 0.5625, 0.693.
3. OPS PC6 out of range. Value: -3.1191. Training min, max, SD, explained variance: -2.4321, 2.9885, 1.256, 0.0488.
4. Unknown FCFP_2 feature: 580453787: [*]C(=N[c](:[*]):[*])[*]
5. Unknown FCFP_2 feature: -1410049896: [*]N([*])[c](:n:[*]):[c]([*]):[*]
6. Unknown FCFP_2 feature: 907036844: [*]N([*])C[c](:[*]):[*]

Feature Contribution

Top features for positive contribution

Fingerprint	Bit/Smiles	Feature Structure	Score

FCFP_2	332760439	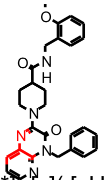 [*][c](:[*]):[c](:[cH]:[*])N=[*]	0.672
FCFP_2	1	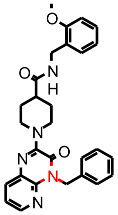 [*]N([*])[*]	0.511
FCFP_2	3	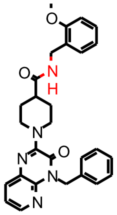 [*]N[*]	0.104
**Top Features for negative contribution**			
**Fingerprint**	**Bit/Smiles**	**Feature Structure**	**Score**
FCFP_2	203677720	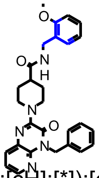 [*]C[c](:[cH]:[*]):[c]([*]):[*]	-0.406
FCFP_2	565998553	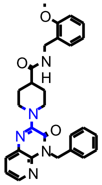 [*]\N=C(\N([*])[*])/C (=[*])[*]	-0.348

FCFP_2	1872154524	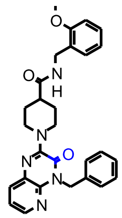 [*]C(=O)[*]	-0.307

#UNDEFINED

TOPKAT_Rat_Maximum_Tolerated_Dose_Gavage

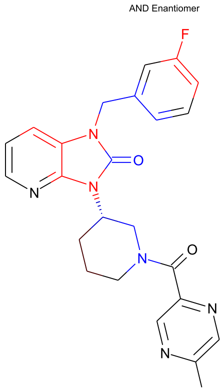

$C_{24}H_{23}FN_6O_2$

Molecular Weight: 446.47682

ALogP: 2.244

Rotatable Bonds: 4

Acceptors: 5

Donors: 0

Model Prediction

Prediction: 0.000787

Unit: g/kg_body_weight

Mahalanobis Distance: 11.5

Mahalanobis Distance p-value: 4.58e-008

Mahalanobis Distance: The Mahalanobis distance (MD) is a generalization of the Euclidean distance that accounts for correlations among the X properties. It is calculated as the distance to the center of the training data. The larger the MD, the less trustworthy the prediction.
Mahalanobis Distance p-value: The p-value gives the fraction of training data with an MD greater than or equal to the one for the given sample, assuming normally distributed data. The smaller the p-value, the less trustworthy the prediciton. For highly non-normal X properties (e.g., fingerprints), the MD p-value is wildly inaccurate.

Structural Similar Compounds

Name	PHENYLBUTAZONE	8-METHOXYPSORALEN	CHLORPHENIRAMINE MALEATE
Structure	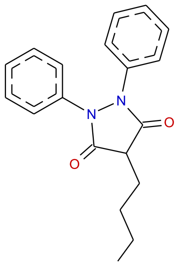	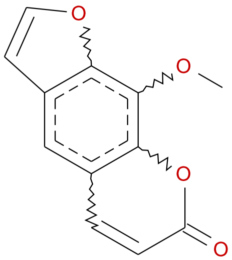	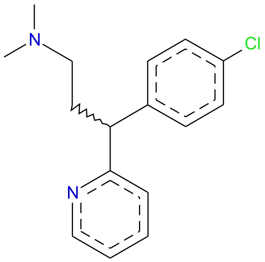
Actual Endpoint (-log C)	3.48909	3.45978	3.96188
Predicted Endpoint (-log C)	3.17333	4.14745	3.83117
Distance	0.976	0.989	1.013
Reference	NCI/NTP TR-367	NCI/NTP TR-359	NCI/NTP TR-317

Model Applicability

Unknown features are fingerprint features in the query molecule, but not found or appearing too infrequently in the training set.

1. Molecular_Weight out of range. Value: 446.48. Training min, max, mean, SD: 68.074, 434.63, 171.13, 85.06.
2. Num_AromaticRings out of range. Value: 3. Training min, max, mean, SD: 0, 2, 0.5625, 0.693.
3. Unknown FCFP_2 feature: -1986158408: [*]N1[*]:[*]N([*])C1=O
4. Unknown FCFP_2 feature: -1410049896: [*]N([*])[c](:n:[*]):[c]([*]):[*]
5. Unknown FCFP_2 feature: 907036844: [*]N([*])C[c](:[*]):[*]

Feature Contribution

Top features for positive contribution

Fingerprint	Bit/Smiles	Feature Structure	Score
FCFP_2	332760439	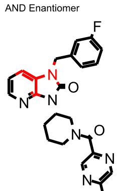 [*][c](:[*]):[c](:[cH]:[*])N=[*]	0.672

FCFP_2	32	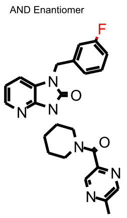 [*]F	0.526
FCFP_2	1	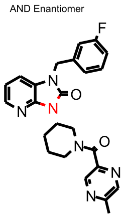 [*]N([*])[*]	0.511

Top Features for negative contribution

Fingerprint	Bit/Smiles	Feature Structure	Score
FCFP_2	203677720	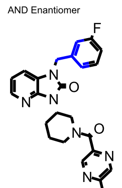 [*]C[c](:[cH]:[*]):[c]([*]):[*]	-0.406
FCFP_2	1872154524	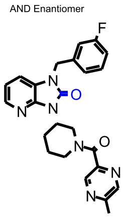 [*]C(=O)[*]	-0.307
FCFP_2	0	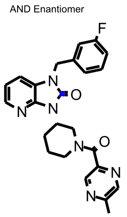 [*]C(=[*])[*]	-0.29

#UNDEFINED

TOPKAT_Rat_Maximum_Tolerated_Dose_Gavage

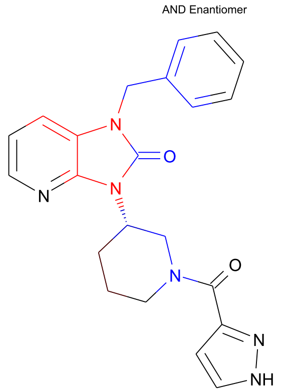

$C_{22}H_{22}N_6O_2$

Molecular Weight: 402.44907

ALogP: 2.501

Rotatable Bonds: 4

Acceptors: 4

Donors: 1

Model Prediction

Prediction: 0.0221

Unit: g/kg_body_weight

Mahalanobis Distance: 12.1

Mahalanobis Distance p-value: 5.92e-009

Mahalanobis Distance: The Mahalanobis distance (MD) is a generalization of the Euclidean distance that accounts for correlations among the X properties. It is calculated as the distance to the center of the training data. The larger the MD, the less trustworthy the prediction.

Mahalanobis Distance p-value: The p-value gives the fraction of training data with an MD greater than or equal to the one for the given sample, assuming normally distributed data. The smaller the p-value, the less trustworthy the prediciton. For highly non-normal X properties (e.g., fingerprints), the MD p-value is wildly inaccurate.

Structural Similar Compounds

Name	SULFISOOXAZOLE	PHENYLBUTAZONE	8-METHOXYPSORALEN
Structure	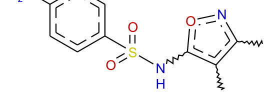	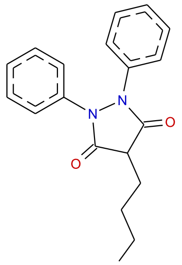	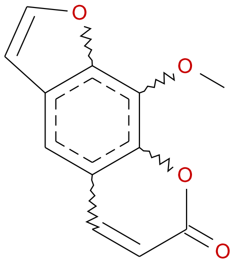
Actual Endpoint (-log C)	2.82494	3.48909	3.45978
Predicted Endpoint (-log C)	3.0705	3.17333	4.14745
Distance	0.921	0.930	0.943
Reference	NCI/NTP TR-138	NCI/NTP TR-367	NCI/NTP TR-359

Model Applicability

Unknown features are fingerprint features in the query molecule, but not found or appearing too infrequently in the training set.

1. Num_AromaticRings out of range. Value: 3. Training min, max, mean, SD: 0, 2, 0.5625, 0.693.
2. OPS PC6 out of range. Value: -2.9302. Training min, max, SD, explained variance: -2.4321, 2.9885, 1.256, 0.0488.
3. Unknown FCFP_2 feature: 19: [*]:[nH]:[*]
4. Unknown FCFP_2 feature: -1986158408: [*]N1[*]:[*]N([*])C1=O
5. Unknown FCFP_2 feature: -1410049896: [*]N([*])[c](:n:[*]):[c]([*]):[*]
6. Unknown FCFP_2 feature: 1747267175: [*][c]1:[*]:[*]:[nH]:n:1
7. Unknown FCFP_2 feature: 262592487: [*]1:[*]:n:[nH]:[cH]:1
8. Unknown FCFP_2 feature: 907036844: [*]N([*])C[c](:[*]):[*]
9. Unknown FCFP_2 feature: 1618184456: [*]1:[*]:[cH]:[cH]:[nH]:1

Feature Contribution

Top features for positive contribution

Fingerprint	Bit/Smiles	Feature Structure	Score

FCFP_2	332760439	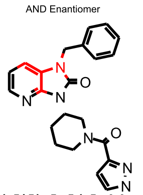 [*][c](:[*]):[c](:[cH]:[*])N=[*]	0.672
FCFP_2	1	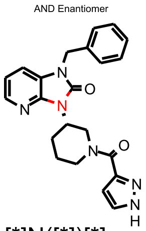 [*]N([*])[*]	0.511
FCFP_2	-1272798659	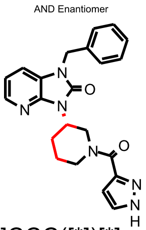 [*]CCC([*])[*]	0.0703

Top Features for negative contribution

Fingerprint	Bit/Smiles	Feature Structure	Score
FCFP_2	203677720	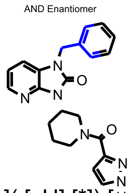 [*]C[c](:[cH]:[*]):[c]([*]):[*]	-0.406
FCFP_2	1872154524	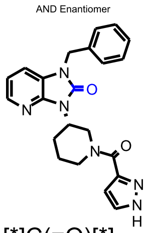 [*]C(=O)[*]	-0.307

FCFP_2	0	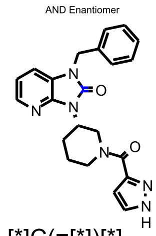 [*]C(=[*])[*]	-0.29

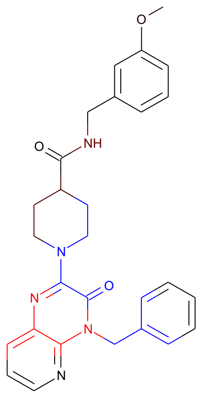

$C_{28}H_{29}N_5O_3$

Molecular Weight: 483.56156

ALogP: 2.942

Rotatable Bonds: 7

Acceptors: 6

Donors: 1

Model Prediction

Prediction: 0.00519

Unit: g/kg_body_weight

Mahalanobis Distance: 13.6

Mahalanobis Distance p-value: 4.88e-011

Mahalanobis Distance: The Mahalanobis distance (MD) is a generalization of the Euclidean distance that accounts for correlations among the X properties. It is calculated as the distance to the center of the training data. The larger the MD, the less trustworthy the prediction.
Mahalanobis Distance p-value: The p-value gives the fraction of training data with an MD greater than or equal to the one for the given sample, assuming normally distributed data. The smaller the p-value, the less trustworthy the prediciton. For highly non-normal X properties (e.g., fingerprints), the MD p-value is wildly inaccurate.

Structural Similar Compounds

Name	OCHRATOXIN	PENICILLIN VK	PHENYLBUTAZONE
Structure	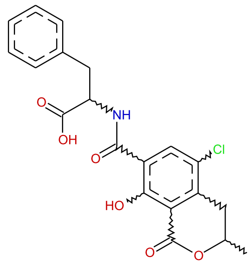	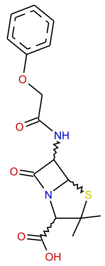	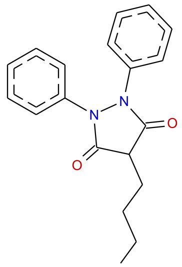
Actual Endpoint (-log C)	6.28396	2.54455	3.48909
Predicted Endpoint (-log C)	5.12358	3.9702	3.17333
Distance	1.023	1.044	1.073
Reference	NCI/NTP TR-358	NCI/NTP TR-336	NCI/NTP TR-367

Model Applicability

Unknown features are fingerprint features in the query molecule, but not found or appearing too infrequently in the training set.

1. Molecular_Weight out of range. Value: 483.56. Training min, max, mean, SD: 68.074, 434.63, 171.13, 85.06.
2. Num_AromaticRings out of range. Value: 3. Training min, max, mean, SD: 0, 2, 0.5625, 0.693.
3. OPS PC6 out of range. Value: -3.1191. Training min, max, SD, explained variance: -2.4321, 2.9885, 1.256, 0.0488.
4. Unknown FCFP_2 feature: 580453787: [*]C(=N[c](:[*]):[*])[*]
5. Unknown FCFP_2 feature: -1410049896: [*]N([*])[c](:n:[*]):[c]([*]):[*]
6. Unknown FCFP_2 feature: 907036844: [*]N([*])C[c](:[*]):[*]

Feature Contribution

Top features for positive contribution

Fingerprint	Bit/Smiles	Feature Structure	Score

FCFP_2	332760439	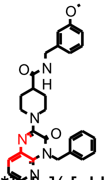 [*][c](:[*]):[c](:[cH]:[*])N=[*]	0.672
FCFP_2	1	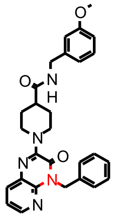 [*]N([*])[*]	0.511
FCFP_2	3	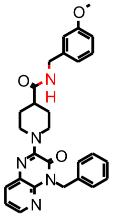 [*]N[*]	0.104

Top Features for negative contribution

Fingerprint	Bit/Smiles	Feature Structure	Score
FCFP_2	203677720	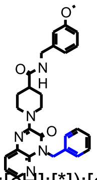 [*]C[c](:[cH]:[*]):[c]([*]):[*]	-0.406
FCFP_2	565998553	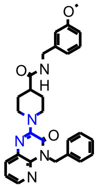 [*]\N=C(\N([*])[*])/C (=[*])[*]	-0.348

FCFP_2	1872154524	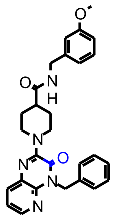 [*]C(=O)[*]	-0.307

#UNDEFINED

TOPKAT_Rat_Maximum_Tolerated_Dose_Gavage

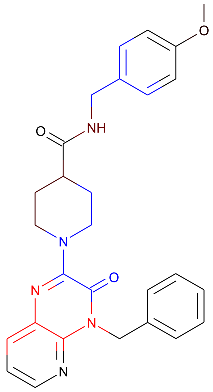

$C_{28}H_{29}N_5O_3$

Molecular Weight: 483.56156

ALogP: 2.942

Rotatable Bonds: 7

Acceptors: 6

Donors: 1

Model Prediction

Prediction: 0.00519

Unit: g/kg_body_weight

Mahalanobis Distance: 13.6

Mahalanobis Distance p-value: 4.88e-011

Mahalanobis Distance: The Mahalanobis distance (MD) is a generalization of the Euclidean distance that accounts for correlations among the X properties. It is calculated as the distance to the center of the training data. The larger the MD, the less trustworthy the prediction.
Mahalanobis Distance p-value: The p-value gives the fraction of training data with an MD greater than or equal to the one for the given sample, assuming normally distributed data. The smaller the p-value, the less trustworthy the prediciton. For highly non-normal X properties (e.g., fingerprints), the MD p-value is wildly inaccurate.

Structural Similar Compounds

Name	OCHRATOXIN	PENICILLIN VK	PHENYLBUTAZONE
Structure	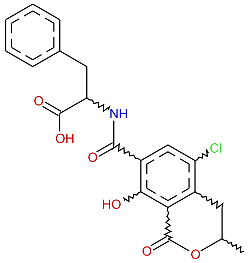	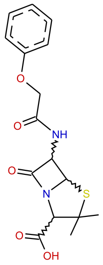	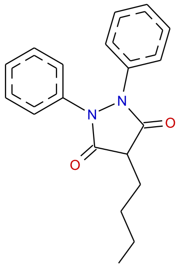
Actual Endpoint (-log C)	6.28396	2.54455	3.48909
Predicted Endpoint (-log C)	5.12358	3.9702	3.17333
Distance	1.023	1.044	1.073
Reference	NCI/NTP TR-358	NCI/NTP TR-336	NCI/NTP TR-367

Model Applicability

Unknown features are fingerprint features in the query molecule, but not found or appearing too infrequently in the training set.

1. Molecular_Weight out of range. Value: 483.56. Training min, max, mean, SD: 68.074, 434.63, 171.13, 85.06.
2. Num_AromaticRings out of range. Value: 3. Training min, max, mean, SD: 0, 2, 0.5625, 0.693.
3. OPS PC6 out of range. Value: -3.1191. Training min, max, SD, explained variance: -2.4321, 2.9885, 1.256, 0.0488.
4. Unknown FCFP_2 feature: 580453787: [*]C(=N[c](:[*]):[*])[*]
5. Unknown FCFP_2 feature: -1410049896: [*]N([*])[c](:n:[*]):[c]([*]):[*]
6. Unknown FCFP_2 feature: 907036844: [*]N([*])C[c](:[*]):[*]

Feature Contribution

Top features for positive contribution

Fingerprint	Bit/Smiles	Feature Structure	Score

FCFP_2	332760439	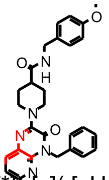 [*][c](:[*]):[c](:[cH]:[*])N=[*]	0.672
FCFP_2	1	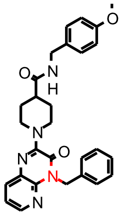 [*]N([*])[*]	0.511
FCFP_2	3	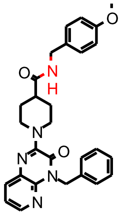 [*]N[*]	0.104

Top Features for negative contribution

Fingerprint	Bit/Smiles	Feature Structure	Score
FCFP_2	203677720	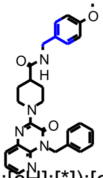 [*]C[c](:[cH]:[*]):[c]([*]):[*]	-0.406
FCFP_2	565998553	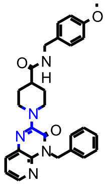 [*]\N=C(\N([*])[*])/C (=[*])[*]	-0.348

FCFP_2	1872154524	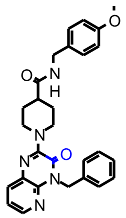 [*]C(=O)[*]	-0.307

Molecule

TOPKAT_Rat_Oral_LD50

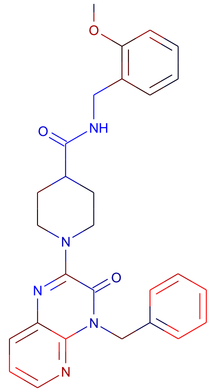

$C_{28}H_{29}N_5O_3$

Molecular Weight: 483.56156

ALogP: 2.942

Rotatable Bonds: 7

Acceptors: 6

Donors: 1

Structural Similar Compounds

Name	bis-OXATIN ACETATE	KETOCONAZOLE	1-ACETYL-3;3-bis-[4-(ACETYLOXY)PHENYL]-1;3-DIHYDRO-2H-INDOL-2-ONE
Structure	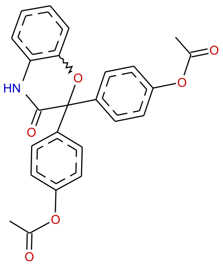	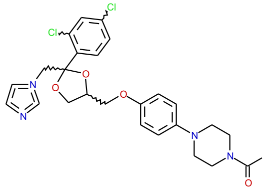	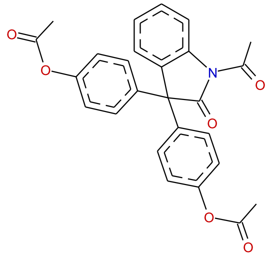
Actual Endpoint (-log C)	1.717	3.505	2.948
Predicted Endpoint (-log C)	2.40947	2.65464	2.6866
Distance	0.540	0.587	0.592
Reference	NIIRDN 6;609;82	MDACAP 17;373;81	JAPMA8 42;468;53

Model Prediction

Prediction: 1.43

Unit: g/kg_body_weight

Mahalanobis Distance: 23.3

Mahalanobis Distance p-value: 4.14e-022

Mahalanobis Distance: The Mahalanobis distance (MD) is a generalization of the Euclidean distance that accounts for correlations among the X properties. It is calculated as the distance to the center of the training data. The larger the MD, the less trustworthy the prediction.
Mahalanobis Distance p-value: The p-value gives the fraction of training data with an MD greater than or equal to the one for the given sample, assuming normally distributed data. The smaller the p-value, the less trustworthy the prediciton. For highly non-normal X properties (e.g., fingerprints), the MD p-value is wildly inaccurate.

Model Applicability

Unknown features are fingerprint features in the query molecule, but not found or appearing too infrequently in the training set.

1. All properties and OPS components are within expected ranges.
2. Unknown FCFP_6 feature: 16: [*][c](:[*]):[*]
3. Unknown FCFP_6 feature: 580453787: [*]C(=N[c](:[*]):[*])[*]
4. Unknown FCFP_6 feature: -1410049896: [*]N([*])[c](:n:[*]):[c]([*]):[*]
5. Unknown FCFP_6 feature: 1747237384: [*][c](:[*]):n:[cH]:[*]
6. Unknown FCFP_6 feature: 907096426: [*]NC[c](:[*]):[*]
7. Unknown FCFP_6 feature: 1618154665: [*][c](:[*]):[cH]:[cH]:[*]

Feature Contribution

Top features for positive contribution			
Fingerprint	Bit/Smiles	Feature Structure	Score

ECFP_6	642810091	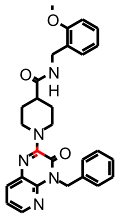 [*]C(=[*])[*]	0.281
ECFP_6	-1897341097	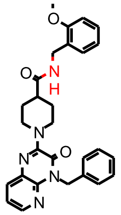 [*]N[*]	0.216
ECFP_6	1571214559	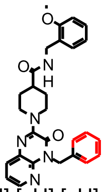 [*]1:[cH]:[cH]:[cH]:[cH]:[cH]:1	0.19
**Top Features for negative contribution**			
**Fingerprint**	**Bit/Smiles**	**Feature Structure**	**Score**
ECFP_6	2106656448	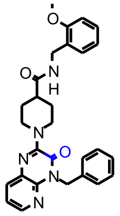 [*]C(=O)[*]	-0.352
ECFP_6	497523368	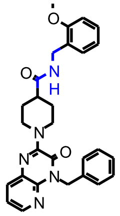 [*]CNC(=[*])[*]	-0.301

ECFP_6	655739385	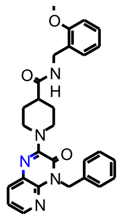 [*]N=[*]	-0.239

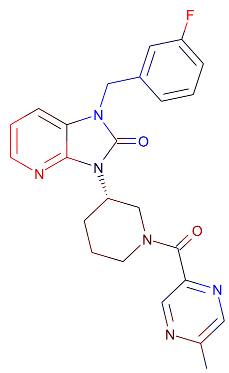

$C_{24}H_{23}FN_6O_2$
Molecular Weight: 446.47682
ALogP: 2.244
Rotatable Bonds: 4
Acceptors: 5
Donors: 0

Structural Similar Compounds

Name	1-ACETYL-3;3-bis-[4-(ACETYLOXY)PHENYL]-1;3-DIHYDRO-2H-INDOL-2-ONE	KETOCONAZOLE	ZOPICIONE
Structure	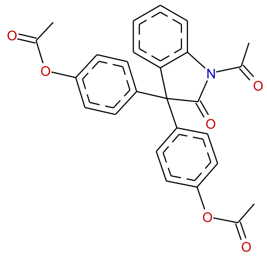	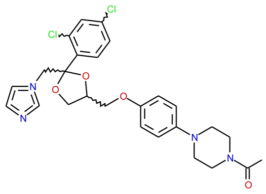	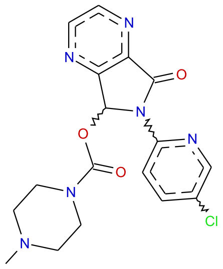
Actual Endpoint (-log C)	2.948	3.505	2.672
Predicted Endpoint (-log C)	2.6866	2.65464	3.16557
Distance	0.591	0.627	0.634
Reference	JAPMA8 42;468;53	MDACAP 17;373;81	OYYAA2 26;935;83

Model Prediction

Prediction: 0.931
Unit: g/kg_body_weight
Mahalanobis Distance: 22
Mahalanobis Distance p-value: 4.08e-016

Mahalanobis Distance: The Mahalanobis distance (MD) is a generalization of the Euclidean distance that accounts for correlations among the X properties. It is calculated as the distance to the center of the training data. The larger the MD, the less trustworthy the prediction.
Mahalanobis Distance p-value: The p-value gives the fraction of training data with an MD greater than or equal to the one for the given sample, assuming normally distributed data. The smaller the p-value, the less trustworthy the prediciton. For highly non-normal X properties (e.g., fingerprints), the MD p-value is wildly inaccurate.

Model Applicability

Unknown features are fingerprint features in the query molecule, but not found or appearing too infrequently in the training set.

1. All properties and OPS components are within expected ranges.
2. Unknown FCFP_6 feature: 16: [*][c](:[*]):[*]
3. Unknown FCFP_6 feature: -1410049896: [*]N([*])[c](:n:[*]):[c]([*]):[*]
4. Unknown FCFP_6 feature: 1747237384: [*][c](:[*]):n:[cH]:[*]
5. Unknown FCFP_6 feature: 1618154665: [*][c](:[*]):[cH]:[cH]:[*]
6. Unknown FCFP_6 feature: 71476542: [*]:[c](:[*])F

Feature Contribution

Top features for positive contribution

Fingerprint	Bit/Smiles	Feature Structure	Score

ECFP_6	-1046436026	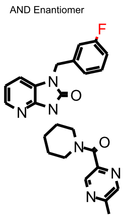 [*]F	0.349
ECFP_6	642810091	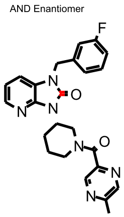 [*]C(=[*])[*]	0.281
FCFP_6	-1549163031	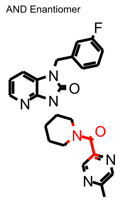 [*]N([*])C(=O)[c](:[*]):[*]	0.171

Top Features for negative contribution

Fingerprint	Bit/Smiles	Feature Structure	Score
ECFP_6	2106656448	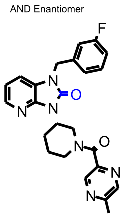 [*]C(=O)[*]	-0.352
ECFP_6	655739385	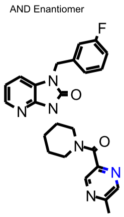 [*]N=[*]	-0.239

FCFP_6	907036844	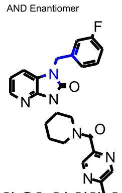 [*]N([*])C[c](:[*]):[*]	-0.222

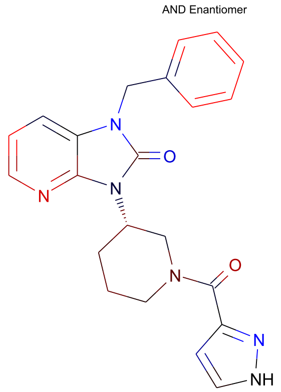

$C_{22}H_{22}N_6O_2$
Molecular Weight: 402.44907
ALogP: 2.501
Rotatable Bonds: 4
Acceptors: 4
Donors: 1

Structural Similar Compounds

Name	1-BENZENESULFONYL-5;5-DIPHENYLHYDANTOIN	9H-PURINE; 6-BENZYLAMINO-9-TETRAHYDROPYRAN-2-YL-	ACRIDINE; 9-[3-(DIMETHYLAMINO)PROPYLAMINO]-1-NITRO-
Structure	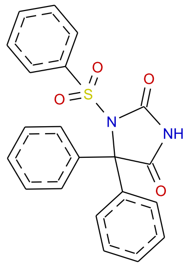	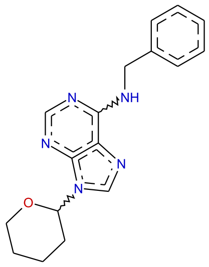	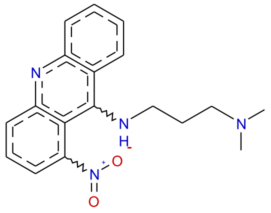
Actual Endpoint (-log C)	2.363	2.276	4.101
Predicted Endpoint (-log C)	2.34793	2.38854	3.3633
Distance	0.546	0.559	0.612
Reference	ARZNAD 20;1579;70	85ARAE 3;48;76/77	MMDPA6 8;252;76

Model Prediction

Prediction: 0.514
Unit: g/kg_body_weight
Mahalanobis Distance: 23
Mahalanobis Distance p-value: 1.38e-020

Mahalanobis Distance: The Mahalanobis distance (MD) is a generalization of the Euclidean distance that accounts for correlations among the X properties. It is calculated as the distance to the center of the training data. The larger the MD, the less trustworthy the prediction.
Mahalanobis Distance p-value: The p-value gives the fraction of training data with an MD greater than or equal to the one for the given sample, assuming normally distributed data. The smaller the p-value, the less trustworthy the prediciton. For highly non-normal X properties (e.g., fingerprints), the MD p-value is wildly inaccurate.

Model Applicability

Unknown features are fingerprint features in the query molecule, but not found or appearing too infrequently in the training set.

1. All properties and OPS components are within expected ranges.
2. Unknown FCFP_6 feature: 16: [*][c](:[*]):[*]
3. Unknown FCFP_6 feature: 19: [*]:[nH]:[*]
4. Unknown FCFP_6 feature: -1410049896: [*]N([*])[c](:n:[*]):[c]([*]):[*]
5. Unknown FCFP_6 feature: 1747267175: [*][c]1:[*]:[*]:[nH]:n:1
6. Unknown FCFP_6 feature: 1747237384: [*][c](:[*]):n:[cH]:[*]
7. Unknown FCFP_6 feature: 262592487: [*]1:[*]:n:[nH]:[cH]:1
8. Unknown FCFP_6 feature: 1618154665: [*][c](:[*]):[cH]:[cH]:[*]
9. Unknown FCFP_6 feature: 1618184456: [*]1:[*]:[cH]:[cH]:[nH]:1

Feature Contribution

Top features for positive contribution

Fingerprint	Bit/Smiles	Feature Structure	Score

ECFP_6	642810091	AND Enantiomer 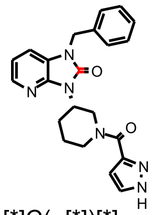 [*]C(=[*])[*]	0.281
ECFP_6	1571214559	AND Enantiomer 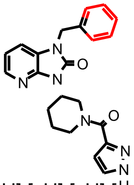 [*]1:[cH]:[cH]:[cH]:[cH]:[cH]:1	0.19
FCFP_6	-1549163031	AND Enantiomer 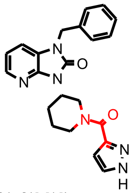 [*]N([*])C(=O)[c](:[*]):[*]	0.171

Top Features for negative contribution

Fingerprint	Bit/Smiles	Feature Structure	Score
ECFP_6	2106656448	AND Enantiomer 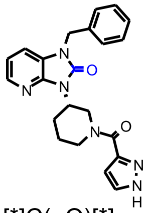 [*]C(=O)[*]	-0.352
ECFP_6	655739385	AND Enantiomer 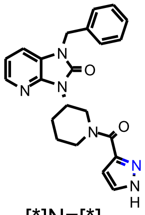 [*]N=[*]	-0.239

FCFP_6	907036844	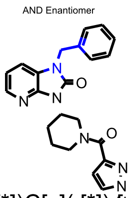 [*]N([*])C[c](:[*]):[*]	-0.222

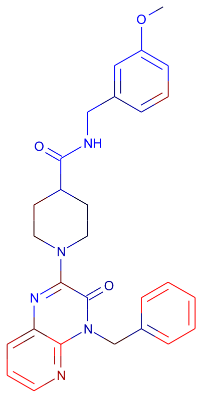

$C_{28}H_{29}N_5O_3$
Molecular Weight: 483.56156
ALogP: 2.942
Rotatable Bonds: 7
Acceptors: 6
Donors: 1

Structural Similar Compounds

Name	bis-OXATIN ACETATE	KETOCONAZOLE	1-ACETYL-3;3-bis-[4-(ACETYLOXY)PHENYL]-1;3-DIHYDRO-2H-INDOL-2-ONE
Structure	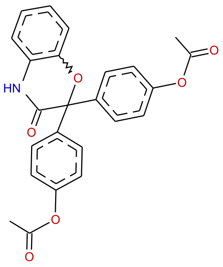	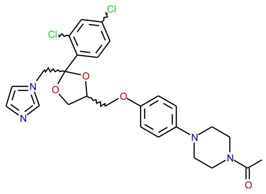	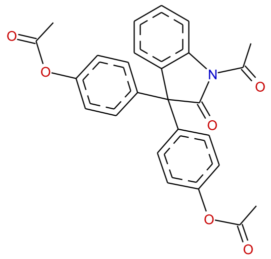
Actual Endpoint (-log C)	1.717	3.505	2.948
Predicted Endpoint (-log C)	2.40947	2.65464	2.6866
Distance	0.538	0.586	0.590
Reference	NIIRDN 6;609;82	MDACAP 17;373;81	JAPMA8 42;468;53

Model Prediction

Prediction: 3.59
Unit: g/kg_body_weight
Mahalanobis Distance: 23.1
Mahalanobis Distance p-value: 2.97e-021

Mahalanobis Distance: The Mahalanobis distance (MD) is a generalization of the Euclidean distance that accounts for correlations among the X properties. It is calculated as the distance to the center of the training data. The larger the MD, the less trustworthy the prediction.
Mahalanobis Distance p-value: The p-value gives the fraction of training data with an MD greater than or equal to the one for the given sample, assuming normally distributed data. The smaller the p-value, the less trustworthy the prediciton. For highly non-normal X properties (e.g., fingerprints), the MD p-value is wildly inaccurate.

Model Applicability

Unknown features are fingerprint features in the query molecule, but not found or appearing too infrequently in the training set.

1. All properties and OPS components are within expected ranges.
2. Unknown FCFP_6 feature: 16: [*][c](:[*]):[*]
3. Unknown FCFP_6 feature: 580453787: [*]C(=N[c](:[*]):[*])[*]
4. Unknown FCFP_6 feature: -1410049896: [*]N([*])[c](:n:[*]):[c]([*]):[*]
5. Unknown FCFP_6 feature: 1747237384: [*][c](:[*]):n:[cH]:[*]
6. Unknown FCFP_6 feature: 907096426: [*]NC[c](:[*]):[*]
7. Unknown FCFP_6 feature: 1618154665: [*][c](:[*]):[cH]:[cH]:[*]

Feature Contribution

Top features for positive contribution			
Fingerprint	Bit/Smiles	Feature Structure	Score

ECFP_6	642810091	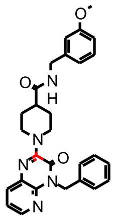 [*]C(=[*])[*]	0.281
ECFP_6	-1897341097	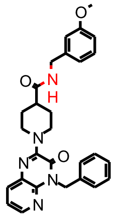 [*]N[*]	0.216
ECFP_6	1571214559	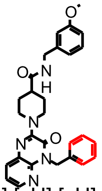 [*]1:[cH]:[cH]:[cH]:[cH]:[cH]:1	0.19

Top Features for negative contribution

Fingerprint	Bit/Smiles	Feature Structure	Score
ECFP_6	2106656448	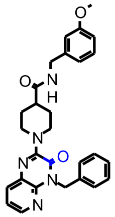 [*]C(=O)[*]	-0.352
ECFP_6	497523368	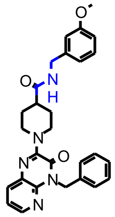 [*]CNC(=[*])[*]	-0.301

ECFP_6	-176455838	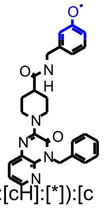 [*]O[c](:[cH]:[*]):[cH]:[*]	-0.257

#UNDEFINED

TOPKAT_Rat_Oral_LD50

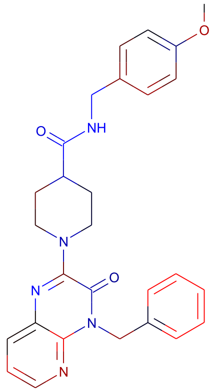

$C_{28}H_{29}N_5O_3$

Molecular Weight: 483.56156

ALogP: 2.942

Rotatable Bonds: 7

Acceptors: 6

Donors: 1

Structural Similar Compounds

Name	bis-OXATIN ACETATE	KETOCONAZOLE	1-ACETYL-3;3-bis-[4-(ACETYLOXY)PHENYL]-1;3-DIHYDRO-2H-INDOL-2-ONE
Structure	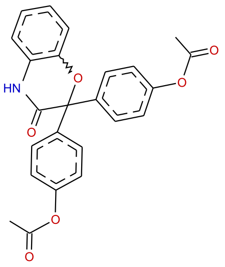	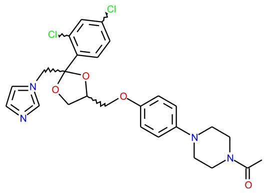	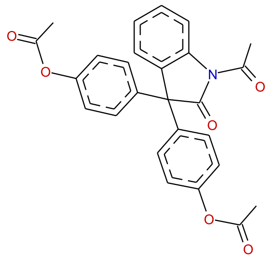
Actual Endpoint (-log C)	1.717	3.505	2.948
Predicted Endpoint (-log C)	2.40947	2.65464	2.6866
Distance	0.534	0.585	0.585
Reference	NIIRDN 6;609;82	MDACAP 17;373;81	JAPMA8 42;468;53

Model Prediction

Prediction: 1.76

Unit: g/kg_body_weight

Mahalanobis Distance: 23.1

Mahalanobis Distance p-value: 2.97e-021

Mahalanobis Distance: The Mahalanobis distance (MD) is a generalization of the Euclidean distance that accounts for correlations among the X properties. It is calculated as the distance to the center of the training data. The larger the MD, the less trustworthy the prediction.
Mahalanobis Distance p-value: The p-value gives the fraction of training data with an MD greater than or equal to the one for the given sample, assuming normally distributed data. The smaller the p-value, the less trustworthy the prediciton. For highly non-normal X properties (e.g., fingerprints), the MD p-value is wildly inaccurate.

Model Applicability

Unknown features are fingerprint features in the query molecule, but not found or appearing too infrequently in the training set.

1. All properties and OPS components are within expected ranges.
2. Unknown FCFP_6 feature: 16: [*][c](:[*]):[*]
3. Unknown FCFP_6 feature: 580453787: [*]C(=N[c](:[*]):[*])[*]
4. Unknown FCFP_6 feature: -1410049896: [*]N([*])[c](:n:[*]):[c]([*]):[*]
5. Unknown FCFP_6 feature: 1747237384: [*][c](:[*]):n:[cH]:[*]
6. Unknown FCFP_6 feature: 907096426: [*]NC[c](:[*]):[*]
7. Unknown FCFP_6 feature: 1618154665: [*][c](:[*]):[cH]:[cH]:[*]

Feature Contribution

Top features for positive contribution

Fingerprint	Bit/Smiles	Feature Structure	Score

ECFP_6	642810091	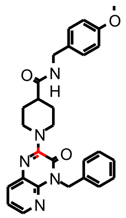 [*]C(=[*])[*]	0.281
ECFP_6	-1897341097	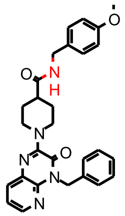 [*]N[*]	0.216
ECFP_6	1571214559	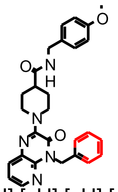 [*]1:[cH]:[cH]:[cH]:[cH]:[cH]:1	0.19

Top Features for negative contribution

Fingerprint	Bit/Smiles	Feature Structure	Score
ECFP_6	2106656448	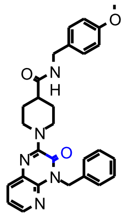 [*]C(=O)[*]	-0.352
ECFP_6	497523368	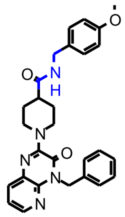 [*]CNC(=[*])[*]	-0.301

ECFP_6	-176455838	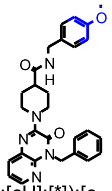 [*]O[c](:[cH]:[*]):[cH]:[*]	-0.257
