# Supplementary material for: Identification of Potential Candidates with Antimicrobial Activity Against Antibiotic-Resistant Staphylococcus aureus Strains: A Hierarchical Bioinformatics Approach
Source: Int J Mol Sci. 2026 Mar 17;27(6):2736. doi: 10.3390/ijms27062736 (PMC13026990; doi:10.3390/ijms27062736)
Supplement: Supplementary file 1 [file ijms-27-02736-s001.zip › Top5_QNZ_ToxicityReports.pdf]

Molecule

TOPKAT_Aerobic_Biodegradability

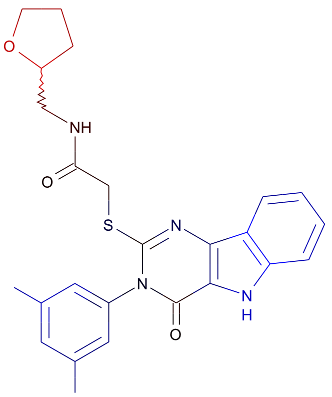

$C_{25}H_{26}N_4O_3S$

Molecular Weight: 462.56393

ALogP: 4.789

Rotatable Bonds: 6

Acceptors: 5

Donors: 2

Structural Similar Compounds

Name	9,10-Anthracenedione,_1-amino-2-(4-bromophenoxy)-4-hydroxy-	Benzenesulfonamide,_3-nitro-N-phenyl-4-(phenylamino)-	Bicyclo_2.2.1_hept-5-ene-2,3-dicarboxylic_acid,_1,4,5,6,7,7-hexachloro-
Structure	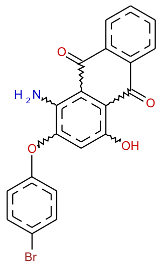	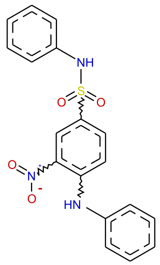	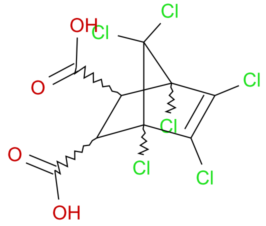
Actual Endpoint	Non-Degradable	Non-Degradable	Non-Degradable
Predicted Endpoint	Non-Degradable	Non-Degradable	Non-Degradable
Distance	0.646	0.687	0.750
Reference	Environmental Toxicology & Chemistry 18(9), 1763-1768, 1999.	Environmental Toxicology & Chemistry 18(9), 1763-1768, 1999.	Environmental Toxicology & Chemistry 18(9), 1763-1768, 1999.

Model Prediction

Prediction: Non-Degradable

Probability: 0.219

Enrichment: 0.502

Bayesian Score: -6.54

Mahalanobis Distance: 18.9

Mahalanobis Distance p-value: 4.79e-027

Prediction: Positive if the Bayesian score is above the estimated best cutoff value from minimizing the false positive and false negative rate.
Probability: The esimated probability that the sample is in the positive category. This assumes that the Bayesian score follows a normal distribution and is different from the prediction using a cutoff.
Enrichment: An estimate of enrichment, that is, the increased likelihood (versus random) of this sample being in the category.
Bayesian Score: The standard Laplacian-modified Bayesian score.
Mahalanobis Distance: The Mahalanobis distance (MD) is the distance to the center of the training data. The larger the MD, the less trustworthy the prediction.
Mahalanobis Distance p-value: The p-value gives the fraction of training data with an MD greater than or equal to the one for the given sample, assuming normally distributed data. The smaller the p-value, the less trustworthy the prediciton. For highly non-normal X properties (e.g., fingerprints), the MD p-value is wildly inaccurate.

Model Applicability

Unknown features are fingerprint features in the query molecule, but not found or appearing too infrequently in the training set.

1. OPS PC13 out of range. Value: -4.905. Training min, max, SD, explained variance: -3.5916, 5.7035, 1.413, 0.0229.

Feature Contribution

Top features for positive contribution

Fingerprint	Bit/Smiles	Feature Structure	Score	Degradable in training set
SCFP_12	-2012880343	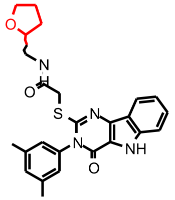 [*]C1CCCO1	0.752	45 out of 51

SCFP_12	-1905025356	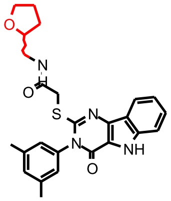 [*]CC1CCCO1	0.59	3 out of 3
SCFP_12	-413535019	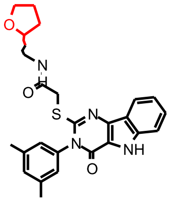 [*]C1CCCO1	0.561	10 out of 13

Top Features for negative contribution

Fingerprint	Bit/Smiles	Feature Structure	Score	Degradable in training set
SCFP_12	-350503170	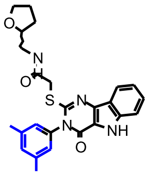 C[c]1:[cH]:[*]:[cH]:[c](C):[cH]:1	-1.47	1 out of 19
SCFP_12	112554633	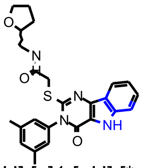 [*]:[cH]:[c]1:[nH]:[*]:[*]:[c]:1:[*]	-1.08	1 out of 12
SCFP_12	-1381862798	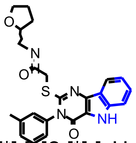 [*]1:[*]:[c]2:[*]:[cH]:[cH]:[cH]:[c]:2:[nH]:1	-1.08	1 out of 12

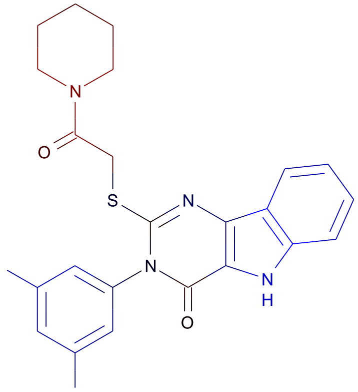

$C_{25}H_{26}N_4O_2S$
Molecular Weight: 446.56453
ALogP: 5.553
Rotatable Bonds: 4
Acceptors: 4
Donors: 1

Structural Similar Compounds

Name	Rhodamine_B	Benzeneacetic_acid,_4-chloro-.alpha.-(4-chlorophenyl)-.alpha.-hydroxy-,_ethyl_ester	Peroxide,_bis(2,4-dichlorobenzoyl)
Structure	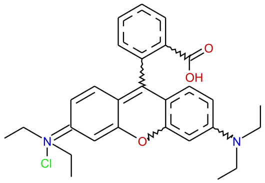	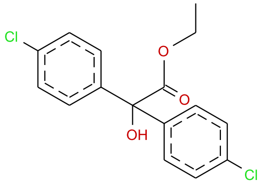	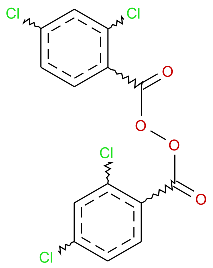
Actual Endpoint	Non-Degradable	Non-Degradable	Non-Degradable
Predicted Endpoint	Non-Degradable	Non-Degradable	Non-Degradable
Distance	0.659	0.744	0.757
Reference	Environmental Toxicology & Chemistry 18(9), 1763-1768, 1999.	Environmental Toxicology & Chemistry 18(9), 1763-1768, 1999.	Environmental Toxicology & Chemistry 18(9), 1763-1768, 1999.

Model Prediction

Prediction: Non-Degradable
Probability: 0.181
Enrichment: 0.415
Bayesian Score: -7.83
Mahalanobis Distance: 17.5
Mahalanobis Distance p-value: 7.56e-021

Prediction: Positive if the Bayesian score is above the estimated best cutoff value from minimizing the false positive and false negative rate.
Probability: The esimated probability that the sample is in the positive category. This assumes that the Bayesian score follows a normal distribution and is different from the prediction using a cutoff.
Enrichment: An estimate of enrichment, that is, the increased likelihood (versus random) of this sample being in the category.
Bayesian Score: The standard Laplacian-modified Bayesian score.
Mahalanobis Distance: The Mahalanobis distance (MD) is the distance to the center of the training data. The larger the MD, the less trustworthy the prediction.
Mahalanobis Distance p-value: The p-value gives the fraction of training data with an MD greater than or equal to the one for the given sample, assuming normally distributed data. The smaller the p-value, the less trustworthy the prediciton. For highly non-normal X properties (e.g., fingerprints), the MD p-value is wildly inaccurate.

Model Applicability

Unknown features are fingerprint features in the query molecule, but not found or appearing too infrequently in the training set.

1. All properties and OPS components are within expected ranges.

Feature Contribution

Top features for positive contribution

Fingerprint	Bit/Smiles	Feature Structure	Score	Degradable in training set
SCFP_12	-2103400817	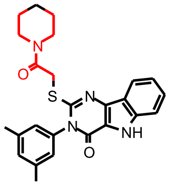 [*]CC(=O)N1CC[*]CC1	0.734	7 out of 7

SCFP_12	1175638033	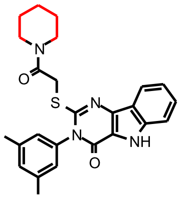 [*]1CCCCC1	0.58	108 out of 148
SCFP_12	-1343150366	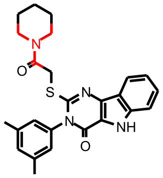 [*]CN(C[*])C(=[*])[*]	0.561	10 out of 13
**Top Features for negative contribution**				
**Fingerprint**	**Bit/Smiles**	**Feature Structure**	**Score**	**Degradable in training set**
SCFP_12	-350503170	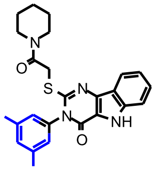 C[c]1:[cH]:[*]:[cH]:[c](C):[cH]:1	-1.47	1 out of 19
SCFP_12	112554633	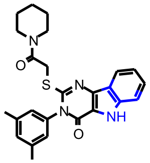 [*]:[cH]:[c]1:[nH]:[*]:[*]:[c]:1:[*]	-1.08	1 out of 12
SCFP_12	-1381862798	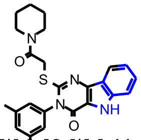 [*]1:[*]:[c]2:[*]:[cH]:[cH]:[cH]:[c]:2:[n H]:1	-1.08	1 out of 12

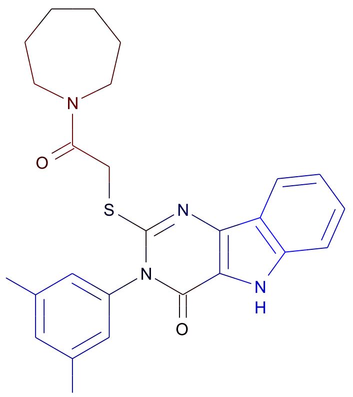

$C_{26}H_{28}N_4O_2S$
Molecular Weight: 460.59111
ALogP: 6.009
Rotatable Bonds: 4
Acceptors: 4
Donors: 1

Structural Similar Compounds

Name	Rhodamine_B	Peroxide,_bis(2,4-dichlorobenzoyl)	Benzeneacetic_acid,_4-chloro-.alpha.-(4-chlorophenyl)-.alpha.-hydroxy-,_ethyl_ester
Structure	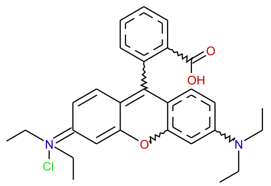	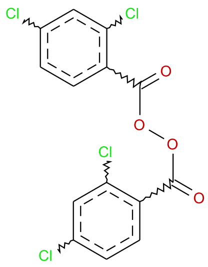	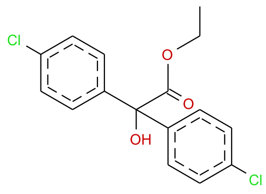
Actual Endpoint	Non-Degradable	Non-Degradable	Non-Degradable
Predicted Endpoint	Non-Degradable	Non-Degradable	Non-Degradable
Distance	0.646	0.769	0.773
Reference	Environmental Toxicology & Chemistry 18(9), 1763-1768, 1999.	Environmental Toxicology & Chemistry 18(9), 1763-1768, 1999.	Environmental Toxicology & Chemistry 18(9), 1763-1768, 1999.

Model Prediction

Prediction: Non-Degradable
Probability: 0.16
Enrichment: 0.367
Bayesian Score: -8.62
Mahalanobis Distance: 17.5
Mahalanobis Distance p-value: 5.43e-021

Prediction: Positive if the Bayesian score is above the estimated best cutoff value from minimizing the false positive and false negative rate.
Probability: The esimated probability that the sample is in the positive category. This assumes that the Bayesian score follows a normal distribution and is different from the prediction using a cutoff.
Enrichment: An estimate of enrichment, that is, the increased likelihood (versus random) of this sample being in the category.
Bayesian Score: The standard Laplacian-modified Bayesian score.
Mahalanobis Distance: The Mahalanobis distance (MD) is the distance to the center of the training data. The larger the MD, the less trustworthy the prediction.
Mahalanobis Distance p-value: The p-value gives the fraction of training data with an MD greater than or equal to the one for the given sample, assuming normally distributed data. The smaller the p-value, the less trustworthy the prediciton. For highly non-normal X properties (e.g., fingerprints), the MD p-value is wildly inaccurate.

Model Applicability

Unknown features are fingerprint features in the query molecule, but not found or appearing too infrequently in the training set.

1. All properties and OPS components are within expected ranges.

Feature Contribution

Top features for positive contribution

Fingerprint	Bit/Smiles	Feature Structure	Score	Degradable in training set
SCFP_12	-2103400817	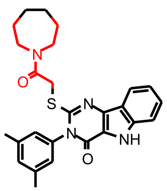 [*]CC(=O)N1CC[*]CC1	0.734	7 out of 7

SCFP_12	1175638033	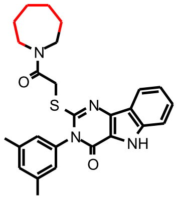 [*]1CCCCC1	0.58	108 out of 148
SCFP_12	-1343150366	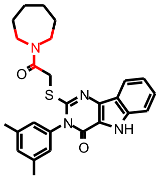 [*]CN(C[*])C(=[*])[*]	0.561	10 out of 13

Top Features for negative contribution

Fingerprint	Bit/Smiles	Feature Structure	Score	Degradable in training set
SCFP_12	-350503170	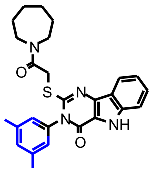 C[c]1:[cH]:[*]:[cH]:[c](C):[cH]:1	-1.47	1 out of 19
SCFP_12	-1381862798	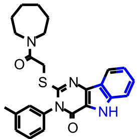 [*]1:[*]:[c]2:[*]:[cH]:[cH]:[cH]:[c]:2:[n H]:1	-1.08	1 out of 12
SCFP_12	112554633	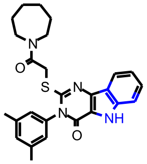 [*]:[cH]:[c]1:[nH]:[*]:[*]:[c]:1:[*]	-1.08	1 out of 12

#UNDEFINED

TOPKAT_Aerobic_Biodegradability

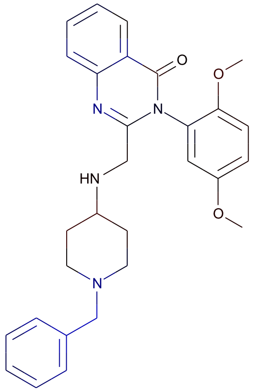

$C_{29}H_{32}N_{4}O_{3}$
Molecular Weight: 484.58938
ALogP: 3.743
Rotatable Bonds: 8
Acceptors: 6
Donors: 1

Structural Similar Compounds

Name	Rhodamine_B	2,5-Cyclohexadiene-1,4-dione,_bis(O-benzoyloxime)	Propanenitrile,_3,3'-__4-_(2,6-dichloro-4-nitrophenyl)azo_phenyl_imino_bis-
Structure	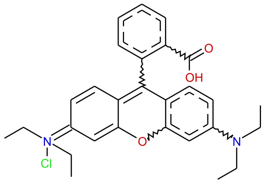	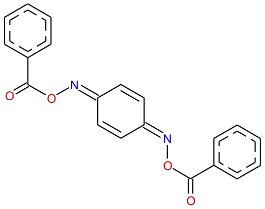	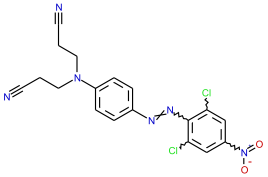
Actual Endpoint	Non-Degradable	Non-Degradable	Non-Degradable
Predicted Endpoint	Non-Degradable	Non-Degradable	Non-Degradable
Distance	0.747	0.826	0.855
Reference	Environmental Toxicology & Chemistry 18(9), 1763-1768, 1999.	Environmental Toxicology & Chemistry 18(9), 1763-1768, 1999.	Environmental Toxicology & Chemistry 18(9), 1763-1768, 1999.

Model Prediction

Prediction: Non-Degradable
Probability: 0.292
Enrichment: 0.669
Bayesian Score: -4.47
Mahalanobis Distance: 14.8
Mahalanobis Distance p-value: 1.9e-011

Prediction: Positive if the Bayesian score is above the estimated best cutoff value from minimizing the false positive and false negative rate.
Probability: The esimated probability that the sample is in the positive category. This assumes that the Bayesian score follows a normal distribution and is different from the prediction using a cutoff.
Enrichment: An estimate of enrichment, that is, the increased likelihood (versus random) of this sample being in the category.
Bayesian Score: The standard Laplacian-modified Bayesian score.
Mahalanobis Distance: The Mahalanobis distance (MD) is the distance to the center of the training data. The larger the MD, the less trustworthy the prediction.
Mahalanobis Distance p-value: The p-value gives the fraction of training data with an MD greater than or equal to the one for the given sample, assuming normally distributed data. The smaller the p-value, the less trustworthy the prediciton. For highly non-normal X properties (e.g., fingerprints), the MD p-value is wildly inaccurate.

Model Applicability

Unknown features are fingerprint features in the query molecule, but not found or appearing too infrequently in the training set.

1. OPS PC16 out of range. Value: 5.3985. Training min, max, SD, explained variance: -3.0094, 4.2513, 1.289, 0.0191.

Feature Contribution

Top features for positive contribution

Fingerprint	Bit/Smiles	Feature Structure	Score	Degradable in training set
SCFP_12	-1272798659	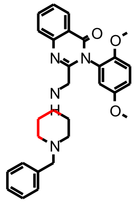 [*]C1[*][*]CC1	0.518	160 out of 234

SCFP_12	1742928053	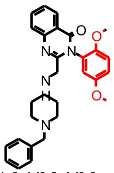 [*]O[c]1:[cH]:[cH]:[c](OC):[cH]:[c]:1[*]	0.504	2 out of 2
SCFP_12	1311071855	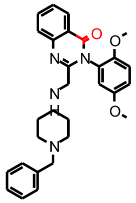 [*]C(=O)[*]	0.461	173 out of 268

Top Features for negative contribution

**Fingerprint**	**Bit/Smiles**	**Feature Structure**	**Score**	**Degradable in training set**
SCFP_12	6	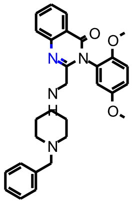 [*]N=[*]	-1.02	2 out of 18
SCFP_12	1851000357	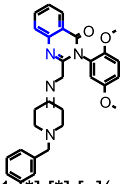 [*][c]1:[*]:[*]:[c](:[*]):[c]:1N=[*]	-0.964	0 out of 4
SCFP_12	-1377141613	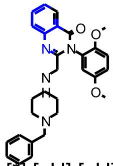 [*][c]1:[*]:[cH]:[cH]:[cH]:[c]:1N=[*]	-0.964	0 out of 4

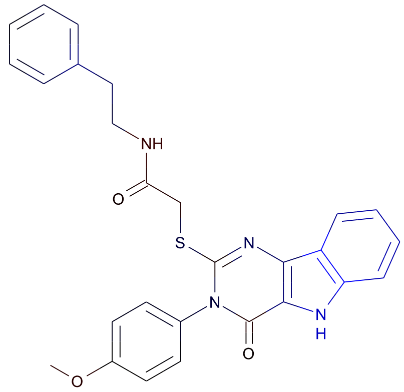

$C_{27}H_{24}N_4O_3S$

Molecular Weight: 484.56946

ALogP: 5.346

Rotatable Bonds: 8

Acceptors: 5

Donors: 2

Structural Similar Compounds

Name	9,10-Anthracenedione,_1-amino-2-(4-bromophenoxy)-4-hydroxy-	Benzenesulfonamide,_3-nitro-N-phenyl-4-(phenylamino)-	Mitin_FF
Structure	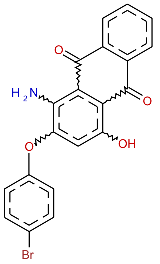	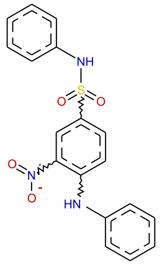	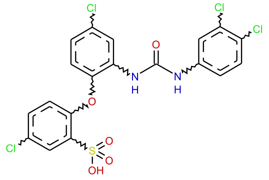
Actual Endpoint	Non-Degradable	Non-Degradable	Non-Degradable
Predicted Endpoint	Non-Degradable	Non-Degradable	Non-Degradable
Distance	0.724	0.741	0.746
Reference	Environmental Toxicology & Chemistry 18(9), 1763-1768, 1999.	Environmental Toxicology & Chemistry 18(9), 1763-1768, 1999.	Environmental Toxicology & Chemistry 18(9), 1763-1768, 1999.

Model Prediction

Prediction: Non-Degradable

Probability: 0.148

Enrichment: 0.339

Bayesian Score: -9.14

Mahalanobis Distance: 18.3

Mahalanobis Distance p-value: 2.01e-024

Prediction: Positive if the Bayesian score is above the estimated best cutoff value from minimizing the false positive and false negative rate.
Probability: The esimated probability that the sample is in the positive category. This assumes that the Bayesian score follows a normal distribution and is different from the prediction using a cutoff.
Enrichment: An estimate of enrichment, that is, the increased likelihood (versus random) of this sample being in the category.
Bayesian Score: The standard Laplacian-modified Bayesian score.
Mahalanobis Distance: The Mahalanobis distance (MD) is the distance to the center of the training data. The larger the MD, the less trustworthy the prediction.
Mahalanobis Distance p-value: The p-value gives the fraction of training data with an MD greater than or equal to the one for the given sample, assuming normally distributed data. The smaller the p-value, the less trustworthy the prediciton. For highly non-normal X properties (e.g., fingerprints), the MD p-value is wildly inaccurate.

Model Applicability

Unknown features are fingerprint features in the query molecule, but not found or appearing too infrequently in the training set.

1. OPS PC15 out of range. Value: 4.9851. Training min, max, SD, explained variance: -4.2438, 4.6197, 1.315, 0.0199.

Feature Contribution

Top features for positive contribution

Fingerprint	Bit/Smiles	Feature Structure	Score	Degradable in training set
SCFP_12	1256995004	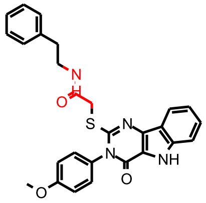 [*]CC(=O)N[*]	0.54	23 out of 32

SCFP_12	1311071855	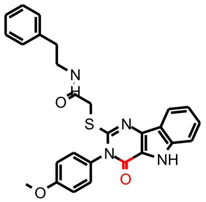 [*]C(=O)[*]	0.461	173 out of 268
SCFP_12	1287669168	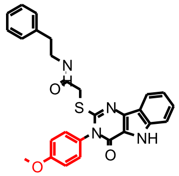 [*][c]1:[cH]:[cH]:[c](OC):[cH]:[cH]:1	0.446	5 out of 7
**Top Features for negative contribution**				
**Fingerprint**	**Bit/Smiles**	**Feature Structure**	**Score**	**Degradable in training set**
SCFP_12	-1381862798	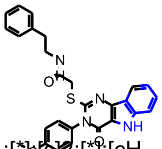 [*]1:[*]:[c]2:[*]:[cH]:[cH]:[cH]:[c]:2:[nH]:1	-1.08	1 out of 12
SCFP_12	112554633	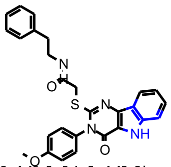 [*]:[cH]:[c]1:[nH]:[*]:[*]:[c]:1:[*]	-1.08	1 out of 12
SCFP_12	6	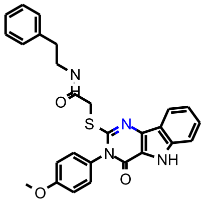 [*]N=[*]	-1.02	2 out of 18

Molecule

TOPKAT_Ames_Mutagenicity

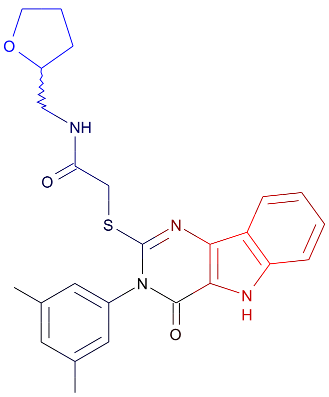

$C_{25}H_{26}N_4O_3S$

Molecular Weight: 462.56393

ALogP: 4.789

Rotatable Bonds: 6

Acceptors: 5

Donors: 2

Model Prediction

Prediction: Non-Mutagen

Probability: 0.63

Enrichment: 1.13

Bayesian Score: -4.17

Mahalanobis Distance: 17.3

Mahalanobis Distance p-value: 1.4e-022

Prediction: Positive if the Bayesian score is above the estimated best cutoff value from minimizing the false positive and false negative rate.
Probability: The esimated probability that the sample is in the positive category. This assumes that the Bayesian score follows a normal distribution and is different from the prediction using a cutoff.
Enrichment: An estimate of enrichment, that is, the increased likelihood (versus random) of this sample being in the category.
Bayesian Score: The standard Laplacian-modified Bayesian score.
Mahalanobis Distance: The Mahalanobis distance (MD) is the distance to the center of the training data. The larger the MD, the less trustworthy the prediction.
Mahalanobis Distance p-value: The p-value gives the fraction of training data with an MD greater than or equal to the one for the given sample, assuming normally distributed data. The smaller the p-value, the less trustworthy the prediciton. For highly non-normal X properties (e.g., fingerprints), the MD p-value is wildly inaccurate.

Structural Similar Compounds

Name	N/A	633-03-4	110021-94-8
Structure	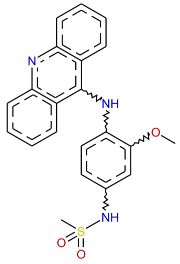	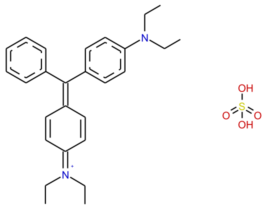	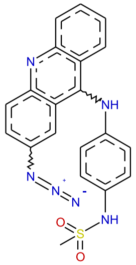
Actual Endpoint	Mutagen	Mutagen	Mutagen
Predicted Endpoint	Mutagen	Mutagen	Mutagen
Distance	0.591	0.605	0.610
Reference	Mut. Res. 280:233-244; 1992	Kazius et. al., J. Med. Chem. (2005) 48, 312-320	Kazius et. al., J. Med. Chem. (2005) 48, 312-320

Model Applicability

Unknown features are fingerprint features in the query molecule, but not found or appearing too infrequently in the training set.

1. OPS PC40 out of range. Value: 5.3918. Training min, max, SD, explained variance: -4.7, 5.0225, 0.8759, 0.0082.

Feature Contribution

Top features for positive contribution

Fingerprint	Bit/Smiles	Feature Structure	Score	Mutagen in training set
SCFP_12	128155983	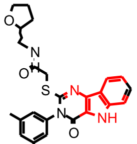 [*][c]1:[nH]:[c]2:[cH]:[*]:[cH]:[cH]:[c]:2:[c]:1N=[*]	0.425	18 out of 20

SCFP_12	2097651564	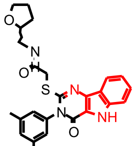<br>[*][c]1:[nH]:[c]2:[cH<br>]:[cH]:[cH]:[cH]:[c]<br>:2:[c]:1N=[*]	0.418	17 out of 19
SCFP_12	-1034807037	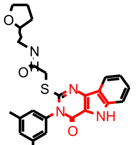<br>[*]N1[*]=N[c]2:[c](:[<br>nH]:[c](:[*]):[c]:2:<br>[*])C1=O	0.418	17 out of 19

Top Features for negative contribution

Fingerprint	Bit/Smiles	Feature Structure	Score	Mutagen in training set
SCFP_12	-37241568	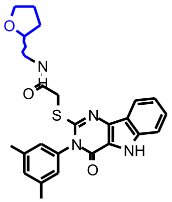<br>[*]CC1CCCO1	-1.12	1 out of 9
SCFP_12	-1905025356	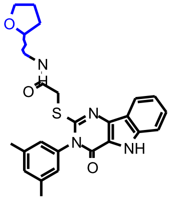<br>[*]CC1CCCO1	-1.05	19 out of 98
SCFP_12	-2012880343	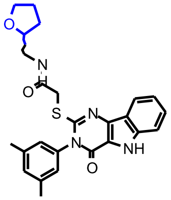<br>[*]C1CCCO1	-0.901	24 out of 106

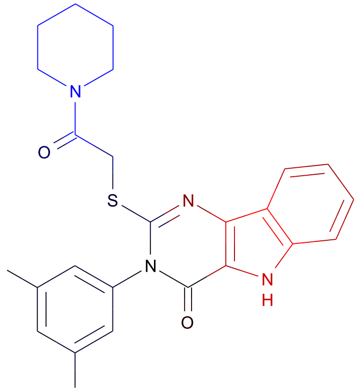

$C_{25}H_{26}N_4O_2S$
Molecular Weight: 446.56453
ALogP: 5.553
Rotatable Bonds: 4
Acceptors: 4
Donors: 1

Model Prediction

Prediction: Non-Mutagen
Probability: 0.633
Enrichment: 1.13
Bayesian Score: -4.07
Mahalanobis Distance: 14
Mahalanobis Distance p-value: 5.26e-009

Prediction: Positive if the Bayesian score is above the estimated best cutoff value from minimizing the false positive and false negative rate.
Probability: The esimated probability that the sample is in the positive category. This assumes that the Bayesian score follows a normal distribution and is different from the prediction using a cutoff.
Enrichment: An estimate of enrichment, that is, the increased likelihood (versus random) of this sample being in the category.
Bayesian Score: The standard Laplacian-modified Bayesian score.
Mahalanobis Distance: The Mahalanobis distance (MD) is the distance to the center of the training data. The larger the MD, the less trustworthy the prediction.
Mahalanobis Distance p-value: The p-value gives the fraction of training data with an MD greater than or equal to the one for the given sample, assuming normally distributed data. The smaller the p-value, the less trustworthy the prediciton. For highly non-normal X properties (e.g., fingerprints), the MD p-value is wildly inaccurate.

Structural Similar Compounds

Name	3-(4'-Phenoxybenzylidenamino)-5H-1;2;3-triazin-[5;4b]indol-4-one	3-(4'-Phenylbenzylidenamino)-5H-1;2;3-triazin-[5;4b]indol-4-one	Ziprasidone [anhydrous;]
Structure	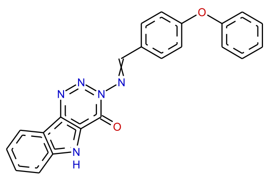	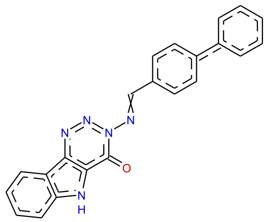	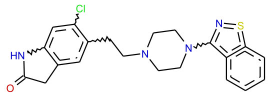
Actual Endpoint	Non-Mutagen	Non-Mutagen	Mutagen
Predicted Endpoint	Mutagen	Mutagen	Mutagen
Distance	0.540	0.545	0.585
Reference	Mutagenesis 7(1):37-39; 1992	Mutagenesis 7(1):37-39; 1992	Contrera, J.F., Matthews, E.J., Kruhlak, N.L., and Benz, R.D., Regulatory Toxicology and Pharmacology 2005, 313-323.

Model Applicability

Unknown features are fingerprint features in the query molecule, but not found or appearing too infrequently in the training set.

1. All properties and OPS components are within expected ranges.

Feature Contribution

Top features for positive contribution				
**Fingerprint**	**Bit/Smiles**	**Feature Structure**	**Score**	**Mutagen in training set**
SCFP_12	128155983	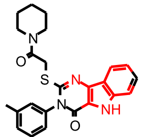 [*][c]1:[nH]:[c]2:[cH]:[*]:[cH]:[cH]:[c]:2:[c]:1N=[*]	0.425	18 out of 20

SCFP_12	-1034807037	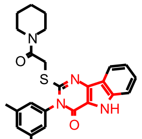 [*]N1[*]=N[c]2:[c](:[nH]:[c](:[*]):[c]:2:[*])C1=O	0.418	17 out of 19
SCFP_12	2097651564	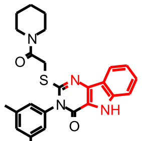 [*][c]1:[nH]:[c]2:[cH]:[cH]:[cH]:[cH]:[c]:2:[c]:1N=[*]	0.418	17 out of 19

Top Features for negative contribution

Fingerprint	Bit/Smiles	Feature Structure	Score	Mutagen in training set
SCFP_12	-1869136583	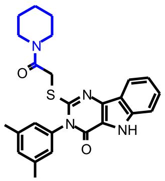 [*]C(=[*])N1CCCCC1	-1.19	0 out of 4
SCFP_12	306578635	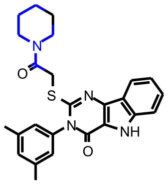 [*]C(=[*])N1C[*]CCC1	-1.09	3 out of 19
SCFP_12	1175638033	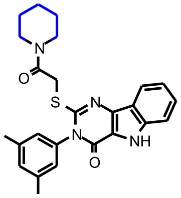 [*]1CCCCC1	-1.05	67 out of 337

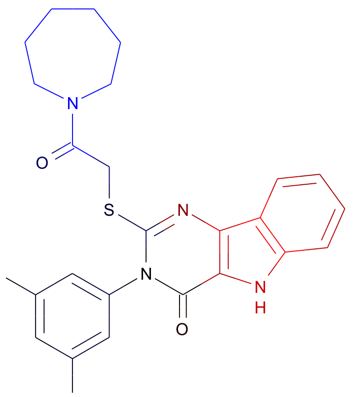

$C_{26}H_{28}N_4O_2S$
Molecular Weight: 460.59111
ALogP: 6.009
Rotatable Bonds: 4
Acceptors: 4
Donors: 1

Structural Similar Compounds

Name	3-(4'-Phenoxybenzylidenamino)-5H-1;2;3-triazin-[5;4b]indol-4-one	3-(4'-Phenylbenzylidenamino)-5H-1;2;3-triazin-[5;4b]indol-4-one	Pigment red 2
Structure	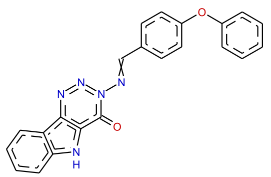	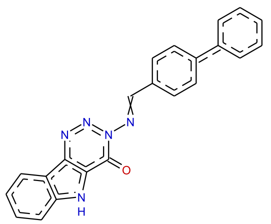	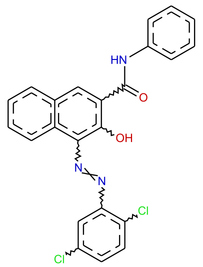
Actual Endpoint	Non-Mutagen	Non-Mutagen	Mutagen
Predicted Endpoint	Mutagen	Mutagen	Mutagen
Distance	0.564	0.571	0.609
Reference	Mutagenesis 7(1):37-39; 1992	Mutagenesis 7(1):37-39; 1992	Environ. Mol. Mut. 19(21):1992

Model Prediction

Prediction: Non-Mutagen
Probability: 0.604
Enrichment: 1.08
Bayesian Score: -4.93
Mahalanobis Distance: 14.1
Mahalanobis Distance p-value: 3.78e-009

Prediction: Positive if the Bayesian score is above the estimated best cutoff value from minimizing the false positive and false negative rate.
Probability: The esimated probability that the sample is in the positive category. This assumes that the Bayesian score follows a normal distribution and is different from the prediction using a cutoff.
Enrichment: An estimate of enrichment, that is, the increased likelihood (versus random) of this sample being in the category.
Bayesian Score: The standard Laplacian-modified Bayesian score.
Mahalanobis Distance: The Mahalanobis distance (MD) is the distance to the center of the training data. The larger the MD, the less trustworthy the prediction.
Mahalanobis Distance p-value: The p-value gives the fraction of training data with an MD greater than or equal to the one for the given sample, assuming normally distributed data. The smaller the p-value, the less trustworthy the prediciton. For highly non-normal X properties (e.g., fingerprints), the MD p-value is wildly inaccurate.

Model Applicability

Unknown features are fingerprint features in the query molecule, but not found or appearing too infrequently in the training set.

1. All properties and OPS components are within expected ranges.

Feature Contribution

Top features for positive contribution

Fingerprint	Bit/Smiles	Feature Structure	Score	Mutagen in training set
SCFP_12	128155983	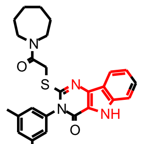 [*][c]1:[nH]:[c]2:[cH]:[*]:[cH]:[cH]:[c]:2:[c]:1N=[*]	0.425	18 out of 20

SCFP_12	-1034807037	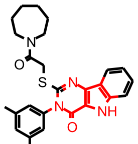 [*]N1[*]=N[c]2:[c](:[nH]:[c](:[*]):[c]:2:[*])C1=O	0.418	17 out of 19
SCFP_12	2097651564	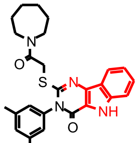 [*][c]1:[nH]:[c]2:[cH]:[cH]:[cH]:[cH]:[c]:2:[c]:1N=[*]	0.418	17 out of 19

Top Features for negative contribution

Fingerprint	Bit/Smiles	Feature Structure	Score	Mutagen in training set
SCFP_12	-1869136583	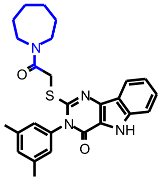 [*]C(=[*])N1CCCCCC1	-1.19	0 out of 4
SCFP_12	306578635	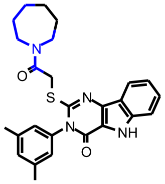 [*]C(=[*])N1C[*]CCC1	-1.09	3 out of 19
SCFP_12	1175638033	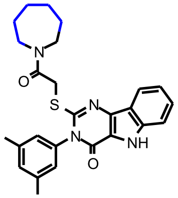 [*]1CCCCC1	-1.05	67 out of 337

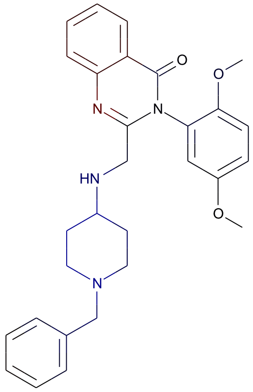

$C_{29}H_{32}N_4O_3$
Molecular Weight: 484.58938
ALogP: 3.743
Rotatable Bonds: 8
Acceptors: 6
Donors: 1

Structural Similar Compounds

Name	83621-06-1	4914-30-1	316-42-7
Structure	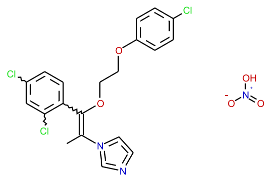	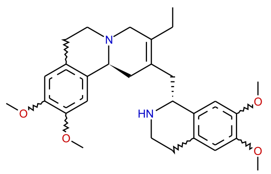	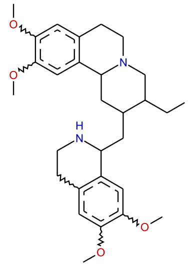
Actual Endpoint	Non-Mutagen	Non-Mutagen	Non-Mutagen
Predicted Endpoint	Non-Mutagen	Non-Mutagen	Non-Mutagen
Distance	0.578	0.590	0.592
Reference	Kazius et. al., J. Med. Chem. (2005) 48, 312-320	Kazius et. al., J. Med. Chem. (2005) 48, 312-320	Kazius et. al., J. Med. Chem. (2005) 48, 312-320

Model Prediction

Prediction: Non-Mutagen
Probability: 0.575
Enrichment: 1.03
Bayesian Score: -5.7
Mahalanobis Distance: 11.3
Mahalanobis Distance p-value: 0.0132

Prediction: Positive if the Bayesian score is above the estimated best cutoff value from minimizing the false positive and false negative rate.
Probability: The esimated probability that the sample is in the positive category. This assumes that the Bayesian score follows a normal distribution and is different from the prediction using a cutoff.
Enrichment: An estimate of enrichment, that is, the increased likelihood (versus random) of this sample being in the category.
Bayesian Score: The standard Laplacian-modified Bayesian score.
Mahalanobis Distance: The Mahalanobis distance (MD) is the distance to the center of the training data. The larger the MD, the less trustworthy the prediction.
Mahalanobis Distance p-value: The p-value gives the fraction of training data with an MD greater than or equal to the one for the given sample, assuming normally distributed data. The smaller the p-value, the less trustworthy the prediciton. For highly non-normal X properties (e.g., fingerprints), the MD p-value is wildly inaccurate.

Model Applicability

Unknown features are fingerprint features in the query molecule, but not found or appearing too infrequently in the training set.

1. All properties and OPS components are within expected ranges.

Feature Contribution

Top features for positive contribution

Fingerprint	Bit/Smiles	Feature Structure	Score	Mutagen in training set
SCFP_12	353415971	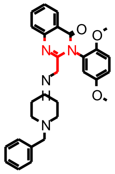 [*]C\C(=N\[*])\N([*])[*]	0.337	2 out of 2

SCFP_12	1648492661	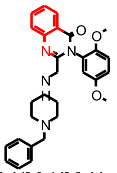 [*][c]1:[cH]:[cH]:[cH]:[cH]:[c]:1N=[*]	0.329	48 out of 60
SCFP_12	-204887640	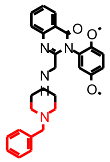 [*]CN(C[*])C[c]1:[cH]:[cH]:[cH]:[cH]:[cH]:1	0.26	4 out of 5

Top Features for negative contribution

**Fingerprint**	**Bit/Smiles**	**Feature Structure**	**Score**	**Mutagen in training set**
SCFP_12	1891546071	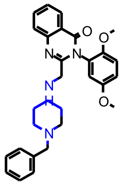 [*]NC1C[*]N([*])CC1	-0.762	0 out of 2
SCFP_12	528802270	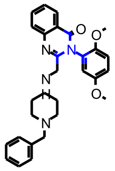 [*]C(=[*])N(C(=[*])[*])[c](:[*]):[*]	-0.496	1 out of 4
SCFP_12	-1272798659	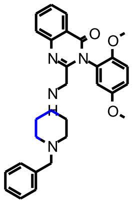 [*]C1[*][*]CC1	-0.466	439 out of 1225

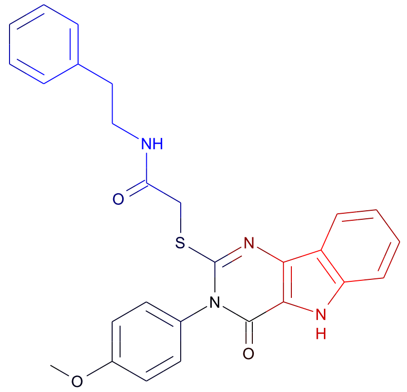

$C_{27}H_{24}N_4O_3S$

Molecular Weight: 484.56946

ALogP: 5.346

Rotatable Bonds: 8

Acceptors: 5

Donors: 2

Structural Similar Compounds

Name	633-03-4	Repaglinide	GLYBURIDE
Structure	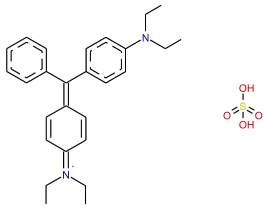	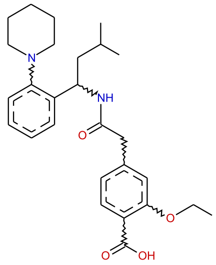	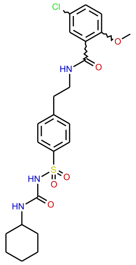
Actual Endpoint	Mutagen	Non-Mutagen	Non-Mutagen
Predicted Endpoint	Mutagen	Non-Mutagen	Non-Mutagen
Distance	0.571	0.602	0.602
Reference	Kazius et. al., J. Med. Chem. (2005) 48, 312-320	Contrera, J.F., Matthews, E.J., Kruhlak, N.L., and Benz, R.D., Regulatory Toxicology and Pharmacology 2005, 313-323.	PDR 1994

Model Prediction

Prediction: Non-Mutagen

Probability: 0.597

Enrichment: 1.07

Bayesian Score: -5.1

Mahalanobis Distance: 11.3

Mahalanobis Distance p-value: 0.0129

Prediction: Positive if the Bayesian score is above the estimated best cutoff value from minimizing the false positive and false negative rate.
Probability: The esimated probability that the sample is in the positive category. This assumes that the Bayesian score follows a normal distribution and is different from the prediction using a cutoff.
Enrichment: An estimate of enrichment, that is, the increased likelihood (versus random) of this sample being in the category.
Bayesian Score: The standard Laplacian-modified Bayesian score.
Mahalanobis Distance: The Mahalanobis distance (MD) is the distance to the center of the training data. The larger the MD, the less trustworthy the prediction.
Mahalanobis Distance p-value: The p-value gives the fraction of training data with an MD greater than or equal to the one for the given sample, assuming normally distributed data. The smaller the p-value, the less trustworthy the prediciton. For highly non-normal X properties (e.g., fingerprints), the MD p-value is wildly inaccurate.

Model Applicability

Unknown features are fingerprint features in the query molecule, but not found or appearing too infrequently in the training set.

1. All properties and OPS components are within expected ranges.

Feature Contribution

Top features for positive contribution				
**Fingerprint**	**Bit/Smiles**	**Feature Structure**	**Score**	**Mutagen in training set**
SCFP_12	128155983	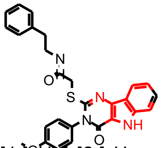 [*][c]1:[nH]:[c]2:[cH]:[*]:[cH]:[cH]:[c]:2:[c]:1N=[*]	0.425	18 out of 20

SCFP_12	-1034807037	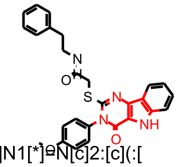<br>[*]N1[*]=N[c]2:[c](:[nH]:[c](:[*]):[c]:2:[*])C1=O	0.418	17 out of 19
SCFP_12	2097651564	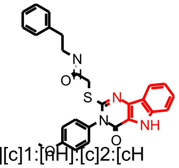<br>[*][c]1:[nH]:[c]2:[cH]:[cH]:[cH]:[cH]:[c]:2:[c]:1N=[*]	0.418	17 out of 19
**Top Features for negative contribution**				
**Fingerprint**	**Bit/Smiles**	**Feature Structure**	**Score**	**Mutagen in training set**
SCFP_12	-1849236245	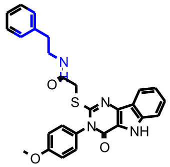<br>[*]NCC[c](:[cH]:[*]):[cH]:[*]	-1.61	0 out of 7
SCFP_12	-2136758352	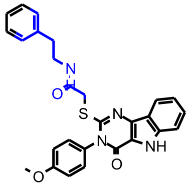<br>[*]CC(=O)NCC[c](:[cH]:[*]):[cH]:[*]	-0.998	0 out of 3
SCFP_12	921720703	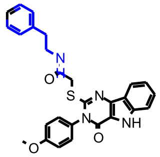<br>[*]C(=[*])NCC[c]1:[cH]:[cH]:[*]:[cH]:[cH]:1	-0.998	0 out of 3

Molecule

TOPKAT_Developmental_Toxicity_Potential

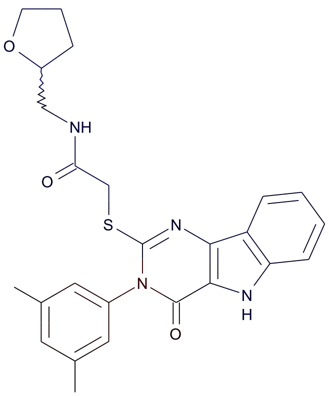

$C_{25}H_{26}N_4O_3S$

Molecular Weight: 462.56393

ALogP: 4.789

Rotatable Bonds: 6

Acceptors: 5

Donors: 2

Structural Similar Compounds

Name	Amsacrine	Estramustine Phosphate Disodium (Free acid form)	Acemetacin
Structure	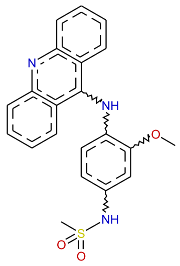	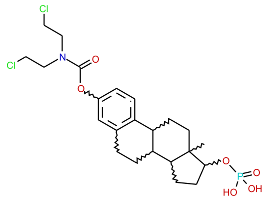	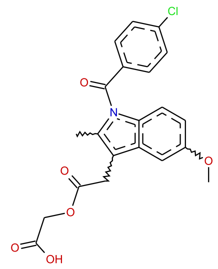
Actual Endpoint	Toxic	Non-Toxic	Non-Toxic
Predicted Endpoint	Toxic	Non-Toxic	Non-Toxic
Distance	0.566	0.620	0.637
Reference	Fundam Appl Toxicol 7(2):214-20; 1986	Oyo Yakuri 20(6):1219-1236; 1980	Oyo Yakuri 22(6):777-786; 1981

Model Prediction

Prediction: Non-Toxic

Probability: 0.42

Enrichment: 0.799

Bayesian Score: -3.72

Mahalanobis Distance: 13.1

Mahalanobis Distance p-value: 2.37e-007

Prediction: Positive if the Bayesian score is above the estimated best cutoff value from minimizing the false positive and false negative rate.
Probability: The esimated probability that the sample is in the positive category. This assumes that the Bayesian score follows a normal distribution and is different from the prediction using a cutoff.
Enrichment: An estimate of enrichment, that is, the increased likelihood (versus random) of this sample being in the category.
Bayesian Score: The standard Laplacian-modified Bayesian score.
Mahalanobis Distance: The Mahalanobis distance (MD) is the distance to the center of the training data. The larger the MD, the less trustworthy the prediction.
Mahalanobis Distance p-value: The p-value gives the fraction of training data with an MD greater than or equal to the one for the given sample, assuming normally distributed data. The smaller the p-value, the less trustworthy the prediciton. For highly non-normal X properties (e.g., fingerprints), the MD p-value is wildly inaccurate.

Model Applicability

Unknown features are fingerprint features in the query molecule, but not found or appearing too infrequently in the training set.

1. OPS PC14 out of range. Value: 4.7181. Training min, max, SD, explained variance: -3.5766, 3.955, 1.214, 0.0216.
2. OPS PC22 out of range. Value: -2.5646. Training min, max, SD, explained variance: -2.4788, 2.7503, 0.9196, 0.0124.

Feature Contribution

Top features for positive contribution

Fingerprint	Bit/Smiles	Feature Structure	Score	Toxic in training set
SCFP_6	-347281112	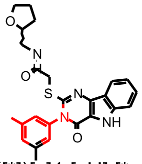 [*]N([*])[c]1:[cH]:[*]:[cH]:[c](C):[cH]:1	0.381	2 out of 2

SCFP_6	1257084377	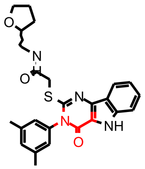 [*]N([*])C(=O)[c](:[*]):[*]	0.362	14 out of 18
SCFP_6	-1905025356	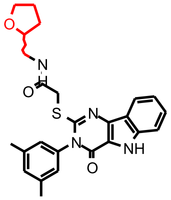 [*]CC1CCCO1	0.25	5 out of 7
**Top Features for negative contribution**				
**Fingerprint**	**Bit/Smiles**	**Feature Structure**	**Score**	**Toxic in training set**
SCFP_6	-413535019	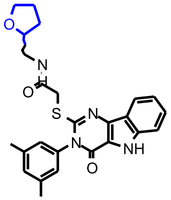 [*]C1CCCO1	-1.29	0 out of 5
SCFP_6	2005026407	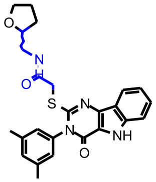 [*]CC(=O)NCC([*])[*]	-0.718	0 out of 2
SCFP_6	-1645120117	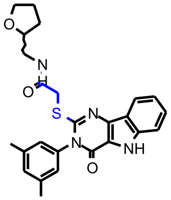 [*]SCC(=[*])[*]	-0.358	3 out of 9

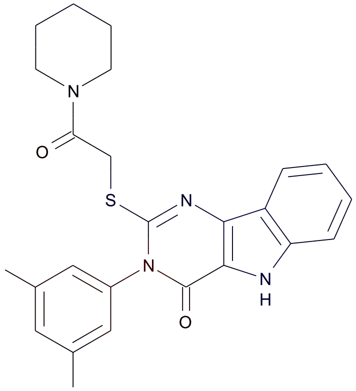

$C_{25}H_{26}N_4O_2S$
Molecular Weight: 446.56453
ALogP: 5.553
Rotatable Bonds: 4
Acceptors: 4
Donors: 1

Model Prediction

Prediction: Non-Toxic
Probability: 0.475
Enrichment: 0.902
Bayesian Score: -2.02
Mahalanobis Distance: 12.2
Mahalanobis Distance p-value: 1.11e-005

Prediction: Positive if the Bayesian score is above the estimated best cutoff value from minimizing the false positive and false negative rate.
Probability: The esimated probability that the sample is in the positive category. This assumes that the Bayesian score follows a normal distribution and is different from the prediction using a cutoff.
Enrichment: An estimate of enrichment, that is, the increased likelihood (versus random) of this sample being in the category.
Bayesian Score: The standard Laplacian-modified Bayesian score.
Mahalanobis Distance: The Mahalanobis distance (MD) is the distance to the center of the training data. The larger the MD, the less trustworthy the prediction.
Mahalanobis Distance p-value: The p-value gives the fraction of training data with an MD greater than or equal to the one for the given sample, assuming normally distributed data. The smaller the p-value, the less trustworthy the prediciton. For highly non-normal X properties (e.g., fingerprints), the MD p-value is wildly inaccurate.

Structural Similar Compounds

Name	Triclabendazole	Benzbromarone	Amsacrine
Structure	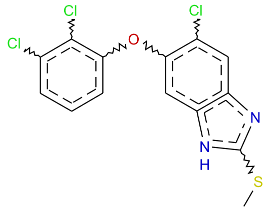	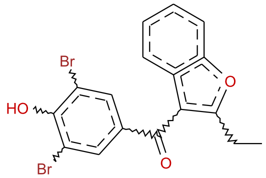	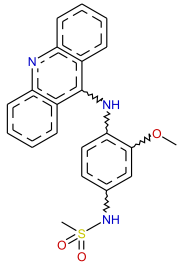
Actual Endpoint	Toxic	Toxic	Toxic
Predicted Endpoint	Toxic	Toxic	Toxic
Distance	0.614	0.618	0.662
Reference	Toxicology 43(3):283-287; 1987	Shinryo to Shinaku 16:1521-1545; 1979	Fundam Appl Toxicol 7(2):214-20; 1986

Model Applicability

Unknown features are fingerprint features in the query molecule, but not found or appearing too infrequently in the training set.

1. OPS PC14 out of range. Value: 4.273. Training min, max, SD, explained variance: -3.5766, 3.955, 1.214, 0.0216.

Feature Contribution

Top features for positive contribution				
Fingerprint	Bit/Smiles	Feature Structure	Score	Toxic in training set
SCFP_6	-2103400817	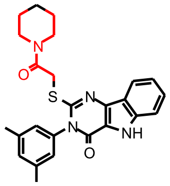 [*]CC(=O)N1CC[*]CC1	0.441	3 out of 3

SCFP_6	-347281112	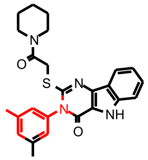 [*]N([*])[c]1:[cH]:[*]:[cH]:[c](C):[cH]:1	0.381	2 out of 2
SCFP_6	1257084377	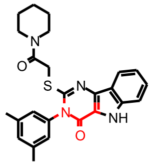 [*]N([*])C(=O)[c](:[*]):[*]	0.362	14 out of 18

Top Features for negative contribution

Fingerprint	Bit/Smiles	Feature Structure	Score	Toxic in training set
SCFP_6	240509252	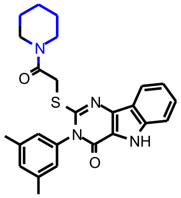 [*]N1[*]CCCC1	-0.438	1 out of 4
SCFP_6	-1645120117	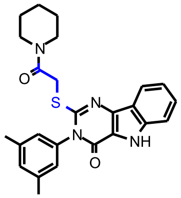 [*]SCC(=[*])[*]	-0.358	3 out of 9
SCFP_6	1851000357	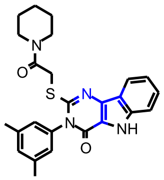 [*][c]1:[*]:[*]:[c](:[*]):[c]:1N=[*]	-0.324	2 out of 6

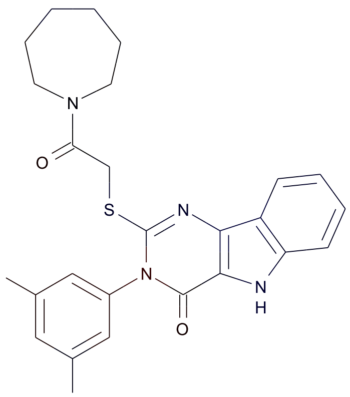

$C_{26}H_{28}N_4O_2S$
Molecular Weight: 460.59111
ALogP: 6.009
Rotatable Bonds: 4
Acceptors: 4
Donors: 1

Structural Similar Compounds

Name	Triclabendazole	Benzbromarone	tri-ortho-Cresyl Phosphate
Structure	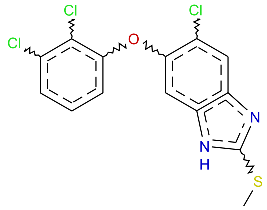	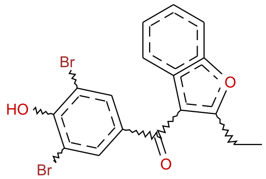	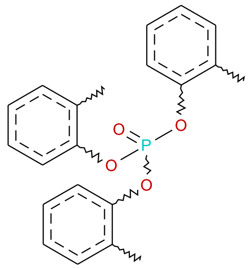
Actual Endpoint	Toxic	Toxic	Non-Toxic
Predicted Endpoint	Toxic	Toxic	Non-Toxic
Distance	0.623	0.623	0.688
Reference	Toxicology 43(3):283-287; 1987	Shinryo to Shinaku 16:1521-1545; 1979	Fundam Appl Toxicol 8:291-297; 1987

Model Prediction

Prediction: Non-Toxic
Probability: 0.475
Enrichment: 0.902
Bayesian Score: -2.02
Mahalanobis Distance: 12.3
Mahalanobis Distance p-value: 8.04e-006

Prediction: Positive if the Bayesian score is above the estimated best cutoff value from minimizing the false positive and false negative rate.
Probability: The esimated probability that the sample is in the positive category. This assumes that the Bayesian score follows a normal distribution and is different from the prediction using a cutoff.
Enrichment: An estimate of enrichment, that is, the increased likelihood (versus random) of this sample being in the category.
Bayesian Score: The standard Laplacian-modified Bayesian score.
Mahalanobis Distance: The Mahalanobis distance (MD) is the distance to the center of the training data. The larger the MD, the less trustworthy the prediction.
Mahalanobis Distance p-value: The p-value gives the fraction of training data with an MD greater than or equal to the one for the given sample, assuming normally distributed data. The smaller the p-value, the less trustworthy the prediciton. For highly non-normal X properties (e.g., fingerprints), the MD p-value is wildly inaccurate.

Model Applicability

Unknown features are fingerprint features in the query molecule, but not found or appearing too infrequently in the training set.

1. OPS PC14 out of range. Value: 4.2989. Training min, max, SD, explained variance: -3.5766, 3.955, 1.214, 0.0216.

Feature Contribution

Top features for positive contribution

Fingerprint	Bit/Smiles	Feature Structure	Score	Toxic in training set
SCFP_6	-2103400817	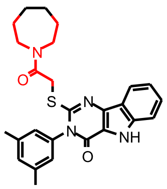 [*]CC(=O)N1CC[*]CC1	0.441	3 out of 3

SCFP_6	-347281112	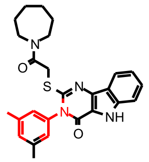 [*]N([*])[c]1:[cH]:[*]:[cH]:[c](C):[cH]:1	0.381	2 out of 2
SCFP_6	1257084377	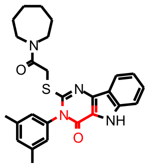 [*]N([*])C(=O)[c](:[*]):[*]	0.362	14 out of 18

Top Features for negative contribution

Fingerprint	Bit/Smiles	Feature Structure	Score	Toxic in training set
SCFP_6	240509252	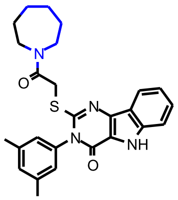 [*]N1[*]CCCC1	-0.438	1 out of 4
SCFP_6	-1645120117	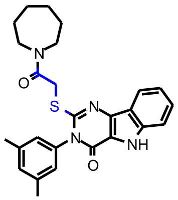 [*]SCC(=[*])[*]	-0.358	3 out of 9
SCFP_6	1851000357	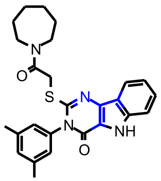 [*][c]1:[*]:[*]:[c](:[*]):[c]:1N=[*]	-0.324	2 out of 6

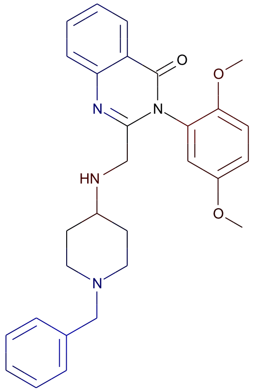

$C_{29}H_{32}N_4O_3$
Molecular Weight: 484.58938
ALogP: 3.743
Rotatable Bonds: 8
Acceptors: 6
Donors: 1

Structural Similar Compounds

Name	Nicardipine	Beclomethasone Dipropionate	Ketoconazole
Structure	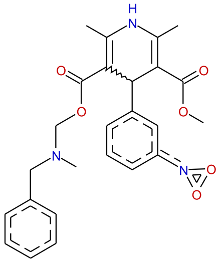	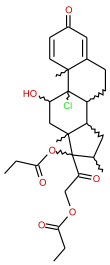	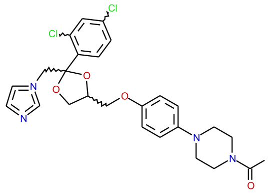
Actual Endpoint	Non-Toxic	Toxic	Toxic
Predicted Endpoint	Non-Toxic	Toxic	Toxic
Distance	0.575	0.605	0.607
Reference	Kiso to Rinsho 13:1149-1159; 1979	Oyo Yakuri 18(6):1021-1038; 1979	Kiso to Rinsho 18:1433-1448; 1984

Model Prediction

Prediction: Non-Toxic
Probability: 0.505
Enrichment: 0.96
Bayesian Score: -1.15
Mahalanobis Distance: 12.4
Mahalanobis Distance p-value: 5.95e-006

Prediction: Positive if the Bayesian score is above the estimated best cutoff value from minimizing the false positive and false negative rate.
Probability: The esimated probability that the sample is in the positive category. This assumes that the Bayesian score follows a normal distribution and is different from the prediction using a cutoff.
Enrichment: An estimate of enrichment, that is, the increased likelihood (versus random) of this sample being in the category.
Bayesian Score: The standard Laplacian-modified Bayesian score.
Mahalanobis Distance: The Mahalanobis distance (MD) is the distance to the center of the training data. The larger the MD, the less trustworthy the prediction.
Mahalanobis Distance p-value: The p-value gives the fraction of training data with an MD greater than or equal to the one for the given sample, assuming normally distributed data. The smaller the p-value, the less trustworthy the prediciton. For highly non-normal X properties (e.g., fingerprints), the MD p-value is wildly inaccurate.

Model Applicability

Unknown features are fingerprint features in the query molecule, but not found or appearing too infrequently in the training set.

1. OPS PC17 out of range. Value: 4.0897. Training min, max, SD, explained variance: -2.7025, 2.8536, 1.067, 0.0167.

Feature Contribution

Top features for positive contribution				
**Fingerprint**	**Bit/Smiles**	**Feature Structure**	**Score**	**Toxic in training set**
SCFP_6	1237755852	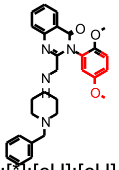 [*][c]1:[*]:[cH]:[cH]:[c](OC):[cH]:1	0.453	8 out of 9

SCFP_6	-1430588017	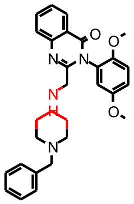 [*]CC(C[*])N[*]	0.415	18 out of 22
SCFP_6	-627385064	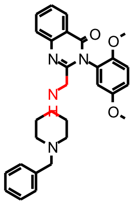 [*]CNC([*])[*]	0.414	14 out of 17
**Top Features for negative contribution**				
**Fingerprint**	**Bit/Smiles**	**Feature Structure**	**Score**	**Toxic in training set**
SCFP_6	-204887640	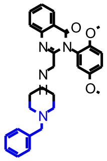 [*]CN(C[*])C[c]1:[cH]:[cH]:[cH]:[cH]:[cH]:1	-0.718	0 out of 2
SCFP_6	1274421524	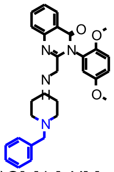 [*]N([*])C[c]1:[cH]:[cH]:[cH]:[cH]:[cH]:1	-0.718	0 out of 2
SCFP_6	1648492661	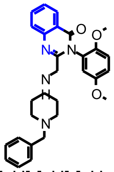 [*][c]1:[cH]:[cH]:[cH]:[cH]:[c]:1N=[*]	-0.718	0 out of 2

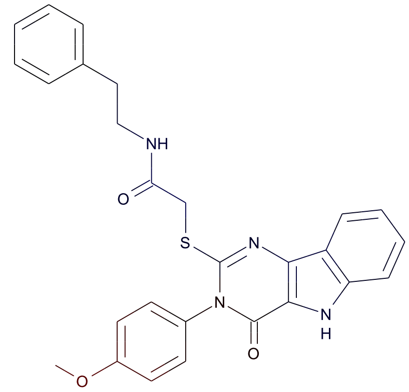

$C_{27}H_{24}N_4O_3S$
Molecular Weight: 484.56946
ALogP: 5.346
Rotatable Bonds: 8
Acceptors: 5
Donors: 2

Model Prediction

Prediction: Non-Toxic
Probability: 0.492
Enrichment: 0.936
Bayesian Score: -1.52
Mahalanobis Distance: 12.7
Mahalanobis Distance p-value: 1.74e-006

Prediction: Positive if the Bayesian score is above the estimated best cutoff value from minimizing the false positive and false negative rate.
Probability: The esimated probability that the sample is in the positive category. This assumes that the Bayesian score follows a normal distribution and is different from the prediction using a cutoff.
Enrichment: An estimate of enrichment, that is, the increased likelihood (versus random) of this sample being in the category.
Bayesian Score: The standard Laplacian-modified Bayesian score.
Mahalanobis Distance: The Mahalanobis distance (MD) is the distance to the center of the training data. The larger the MD, the less trustworthy the prediction.
Mahalanobis Distance p-value: The p-value gives the fraction of training data with an MD greater than or equal to the one for the given sample, assuming normally distributed data. The smaller the p-value, the less trustworthy the prediciton. For highly non-normal X properties (e.g., fingerprints), the MD p-value is wildly inaccurate.

Structural Similar Compounds

Name	Estramustine Phosphate Disodium (Free acid form)	Acemetacin	Amsacrine
Structure	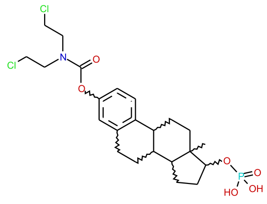	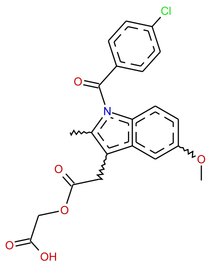	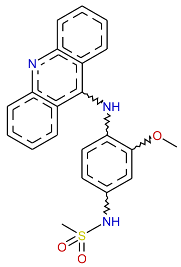
Actual Endpoint	Non-Toxic	Non-Toxic	Toxic
Predicted Endpoint	Non-Toxic	Non-Toxic	Toxic
Distance	0.536	0.652	0.654
Reference	Oyo Yakuri 20(6):1219-1236; 1980	Oyo Yakuri 22(6):777-786; 1981	Fundam Appl Toxicol 7(2):214-20; 1986

Model Applicability

Unknown features are fingerprint features in the query molecule, but not found or appearing too infrequently in the training set.

1. All properties and OPS components are within expected ranges.

Feature Contribution

Top features for positive contribution

Fingerprint	Bit/Smiles	Feature Structure	Score	Toxic in training set
SCFP_6	1237755852	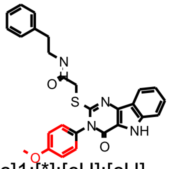 [*][c]1:[*]:[cH]:[cH]:[c](OC):[cH]:1	0.453	8 out of 9

SCFP_6	591469355	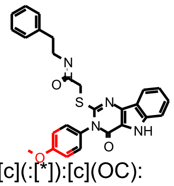 [*][c](:[*]):[c](OC):[cH]:[*]	0.411	10 out of 12
SCFP_6	1257084377	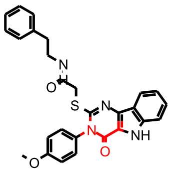 [*]N([*])C(=O)[c](:[*]):[*]	0.362	14 out of 18

Top Features for negative contribution

Fingerprint	Bit/Smiles	Feature Structure	Score	Toxic in training set
SCFP_6	2005026407	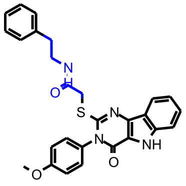 [*]CC(=O)NCC([*])[*]	-0.718	0 out of 2
SCFP_6	-1645120117	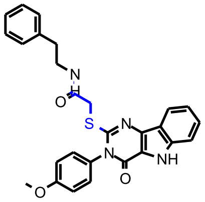 [*]SCC(=[*])[*]	-0.358	3 out of 9
SCFP_6	1851000357	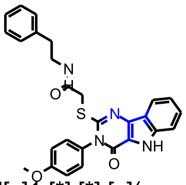 [*][c]1:[*]:[*]:[c](:[*]):[c]:1N=[*]	-0.324	2 out of 6

Molecule

TOPKAT_Mouse_Female_FDA_None_vs_Carcinogen

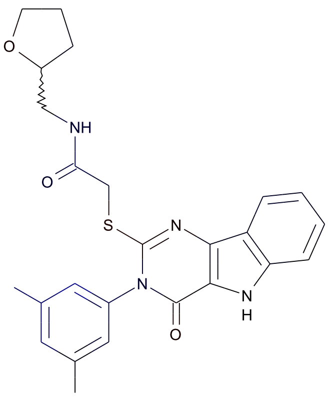

$C_{25}H_{26}N_4O_3S$

Molecular Weight: 462.56393

ALogP: 4.789

Rotatable Bonds: 6

Acceptors: 5

Donors: 2

Model Prediction

Prediction: Non-Carcinogen

Probability: 0.223

Enrichment: 0.695

Bayesian Score: -2.7

Mahalanobis Distance: 11.2

Mahalanobis Distance p-value: 0.0689

Prediction: Positive if the Bayesian score is above the estimated best cutoff value from minimizing the false positive and false negative rate.
Probability: The esimated probability that the sample is in the positive category. This assumes that the Bayesian score follows a normal distribution and is different from the prediction using a cutoff.
Enrichment: An estimate of enrichment, that is, the increased likelihood (versus random) of this sample being in the category.
Bayesian Score: The standard Laplacian-modified Bayesian score.
Mahalanobis Distance: The Mahalanobis distance (MD) is the distance to the center of the training data. The larger the MD, the less trustworthy the prediction.
Mahalanobis Distance p-value: The p-value gives the fraction of training data with an MD greater than or equal to the one for the given sample, assuming normally distributed data. The smaller the p-value, the less trustworthy the prediciton. For highly non-normal X properties (e.g., fingerprints), the MD p-value is wildly inaccurate.

Structural Similar Compounds

Name	Glimepride	Bicalutamide	Glyburide
Structure	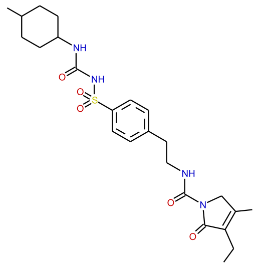	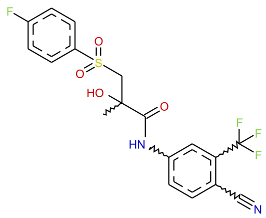	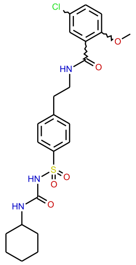
Actual Endpoint	Carcinogen	Non-Carcinogen	Non-Carcinogen
Predicted Endpoint	Carcinogen	Non-Carcinogen	Non-Carcinogen
Distance	0.632	0.653	0.655
Reference	US FDA (Centre for Drug Eval.& Res./Off. Testing & Res.) Sept. 1997	US FDA (Centre for Drug Eval.& Res./Off. Testing & Res.) Sept. 1997	US FDA (Centre for Drug Eval.& Res./Off. Testing & Res.) Sept. 1997

Model Applicability

Unknown features are fingerprint features in the query molecule, but not found or appearing too infrequently in the training set.

1. All properties and OPS components are within expected ranges.
2. Unknown ECFP_2 feature: -962771238: [*]C(=[*])N(C(=[*])[*])[c](:[*]):[*]
3. Unknown ECFP_2 feature: -962137479: [*][c]1:[*]:[*]:[c](:[*]):[c]:1N=[*]
4. Unknown ECFP_2 feature: 676970202: [*]S\C(=N\[*])\N([*])[*]
5. Unknown ECFP_2 feature: 1427820655: [*]CSC(=[*])[*]
6. Unknown ECFP_2 feature: -955816473: [*]SCC(=[*])[*]

Feature Contribution

Top features for positive contribution				
Fingerprint	Bit/Smiles	Feature Structure	Score	Carcinogen in training set

ECFP_6	-830332112	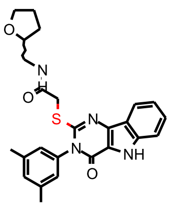 [*]S[*]	0.546	5 out of 8
ECFP_6	-554480104	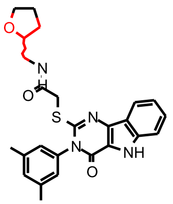 [*]CC1C[*][*]O1	0.364	4 out of 8
ECFP_6	-91640731	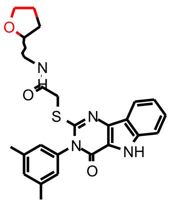 [*]1[*]OCC1	0.337	3 out of 6

Top Features for negative contribution

Fingerprint	Bit/Smiles	Feature Structure	Score	Carcinogen in training set
ECFP_6	-179515162	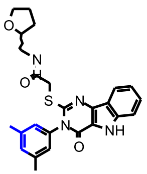 [*]:[cH]:[c](C):[cH]:[*]	-1.41	0 out of 10
ECFP_6	-175021654	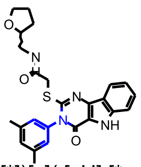 [*]N([*])[c](:[cH]:[*]):[cH]:[*]	-0.805	0 out of 4

ECFP_6	1731843802	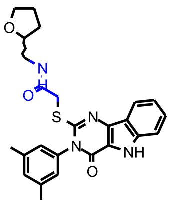 [*]CC(=O)N[*]	-0.657	0 out of 3

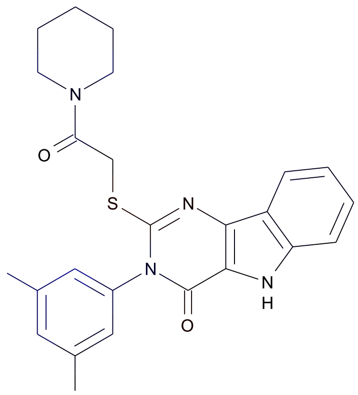

$C_{25}H_{26}N_4O_2S$

Molecular Weight: 446.56453

ALogP: 5.553

Rotatable Bonds: 4

Acceptors: 4

Donors: 1

Model Prediction

Prediction: Non-Carcinogen

Probability: 0.208

Enrichment: 0.65

Bayesian Score: -4.67

Mahalanobis Distance: 11.5

Mahalanobis Distance p-value: 0.0395

Prediction: Positive if the Bayesian score is above the estimated best cutoff value from minimizing the false positive and false negative rate.
Probability: The esimated probability that the sample is in the positive category. This assumes that the Bayesian score follows a normal distribution and is different from the prediction using a cutoff.
Enrichment: An estimate of enrichment, that is, the increased likelihood (versus random) of this sample being in the category.
Bayesian Score: The standard Laplacian-modified Bayesian score.
Mahalanobis Distance: The Mahalanobis distance (MD) is the distance to the center of the training data. The larger the MD, the less trustworthy the prediction.
Mahalanobis Distance p-value: The p-value gives the fraction of training data with an MD greater than or equal to the one for the given sample, assuming normally distributed data. The smaller the p-value, the less trustworthy the prediciton. For highly non-normal X properties (e.g., fingerprints), the MD p-value is wildly inaccurate.

Structural Similar Compounds

Name	Indomethacin	Ethynodiol	Simvastatin
Structure	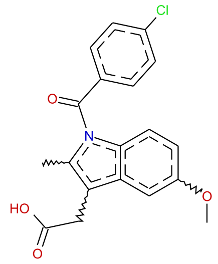	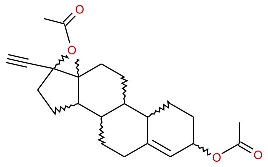	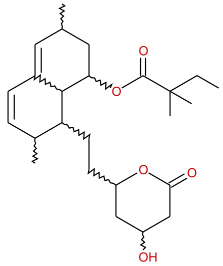
Actual Endpoint	Non-Carcinogen	Carcinogen	Carcinogen
Predicted Endpoint	Non-Carcinogen	Carcinogen	Carcinogen
Distance	0.654	0.685	0.703
Reference	US FDA (Centre for Drug Eval.& Res./Off. Testing & Res.) Sept. 1997	US FDA (Centre for Drug Eval.& Res./Off. Testing & Res.) Sept. 1997	US FDA (Centre for Drug Eval.& Res./Off. Testing & Res.) Sept. 1997

Model Applicability

Unknown features are fingerprint features in the query molecule, but not found or appearing too infrequently in the training set.

1. All properties and OPS components are within expected ranges.
2. Unknown ECFP_2 feature: -962771238: [*]C(=[*])N(C(=[*])[*])[c](:[*]):[*]
3. Unknown ECFP_2 feature: -962137479: [*][c]1:[*]:[*]:[c](:[*]):[c]:1N=[*]
4. Unknown ECFP_2 feature: 676970202: [*]S\C(=N\[*])\N([*])[*]
5. Unknown ECFP_2 feature: 1427820655: [*]CSC(=[*])[*]
6. Unknown ECFP_2 feature: -955816473: [*]SCC(=[*])[*]

Feature Contribution

Top features for positive contribution				
**Fingerprint**	**Bit/Smiles**	**Feature Structure**	**Score**	**Carcinogen in training set**

ECFP_6	-830332112	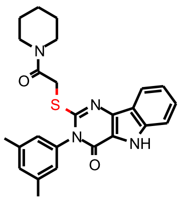 [*]S[*]	0.546	5 out of 8
ECFP_6	2085698692	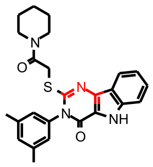 [*]C(=N[c](:[*]):[*]) [*]	0.337	3 out of 6
ECFP_6	2106656448	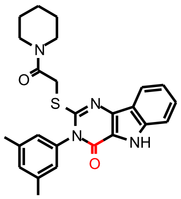 [*]C(=O)[*]	0.254	31 out of 77
**Top Features for negative contribution**				
**Fingerprint**	**Bit/Smiles**	**Feature Structure**	**Score**	**Carcinogen in training set**
ECFP_6	-179515162	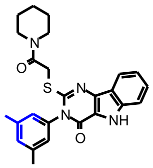 [*]:[cH]:[c](C):[cH]: [*]	-1.41	0 out of 10
ECFP_6	-175021654	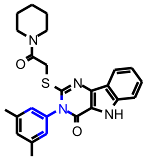 [*]N([*])[c](:[cH]:[*]):[cH]:[*]	-0.805	0 out of 4

ECFP_6	-1102925512	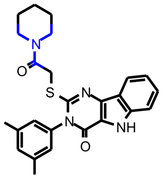 [*]CN(C[*])C(=[*])[*]	-0.805	0 out of 4

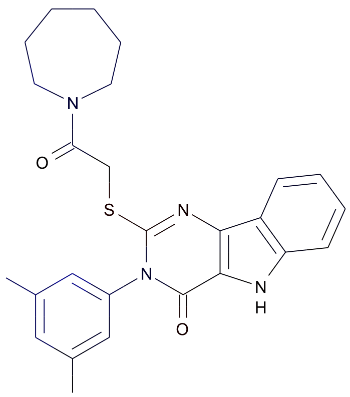

$C_{26}H_{28}N_4O_2S$
Molecular Weight: 460.59111
ALogP: 6.009
Rotatable Bonds: 4
Acceptors: 4
Donors: 1

Structural Similar Compounds

Name	Indomethacin	Ethynodiol	Simvastatin
Structure	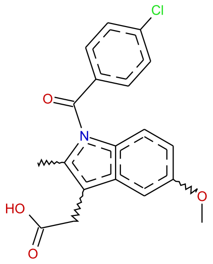	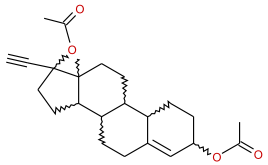	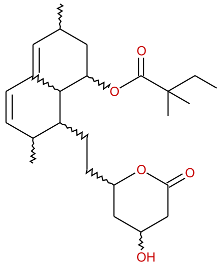
Actual Endpoint	Non-Carcinogen	Carcinogen	Carcinogen
Predicted Endpoint	Non-Carcinogen	Carcinogen	Carcinogen
Distance	0.699	0.708	0.724
Reference	US FDA (Centre for Drug Eval.& Res./Off. Testing & Res.) Sept. 1997	US FDA (Centre for Drug Eval.& Res./Off. Testing & Res.) Sept. 1997	US FDA (Centre for Drug Eval.& Res./Off. Testing & Res.) Sept. 1997

Model Prediction

Prediction: Non-Carcinogen
Probability: 0.207
Enrichment: 0.647
Bayesian Score: -4.89
Mahalanobis Distance: 11.6
Mahalanobis Distance p-value: 0.0299

Prediction: Positive if the Bayesian score is above the estimated best cutoff value from minimizing the false positive and false negative rate.
Probability: The esimated probability that the sample is in the positive category. This assumes that the Bayesian score follows a normal distribution and is different from the prediction using a cutoff.
Enrichment: An estimate of enrichment, that is, the increased likelihood (versus random) of this sample being in the category.
Bayesian Score: The standard Laplacian-modified Bayesian score.
Mahalanobis Distance: The Mahalanobis distance (MD) is the distance to the center of the training data. The larger the MD, the less trustworthy the prediction.
Mahalanobis Distance p-value: The p-value gives the fraction of training data with an MD greater than or equal to the one for the given sample, assuming normally distributed data. The smaller the p-value, the less trustworthy the prediciton. For highly non-normal X properties (e.g., fingerprints), the MD p-value is wildly inaccurate.

Model Applicability

Unknown features are fingerprint features in the query molecule, but not found or appearing too infrequently in the training set.

1. All properties and OPS components are within expected ranges.
2. Unknown ECFP_2 feature: -962771238: [*]C(=[*])N(C(=[*])[*])[c](:[*]):[*]
3. Unknown ECFP_2 feature: -962137479: [*][c]1:[*]:[*]:[c](:[*]):[c]:1N=[*]
4. Unknown ECFP_2 feature: 676970202: [*]S\C(=N\[*])\N([*])[*]
5. Unknown ECFP_2 feature: 1427820655: [*]CSC(=[*])[*]
6. Unknown ECFP_2 feature: -955816473: [*]SCC(=[*])[*]

Feature Contribution

Top features for positive contribution

Fingerprint	Bit/Smiles	Feature Structure	Score	Carcinogen in training set

ECFP_6	-830332112	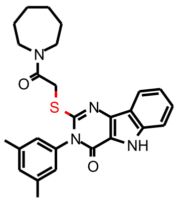 [*]S[*]	0.546	5 out of 8
ECFP_6	2085698692	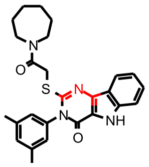 [*]C(=N[c](:[*]):[*])[*]	0.337	3 out of 6
ECFP_6	2106656448	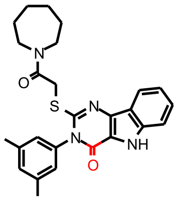 [*]C(=O)[*]	0.254	31 out of 77
**Top Features for negative contribution**				
**Fingerprint**	**Bit/Smiles**	**Feature Structure**	**Score**	**Carcinogen in training set**
ECFP_6	-179515162	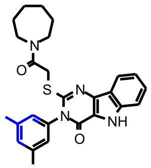 [*]:[cH]:[c](C):[cH]:[*]	-1.41	0 out of 10
ECFP_6	-1102925512	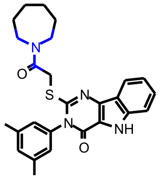 [*]CN(C[*])C(=[*])[*]	-0.805	0 out of 4

ECFP_6	-175021654	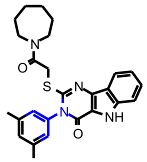 [*]N([*])[c](:[cH]:[*]):[cH]:[*]	-0.805	0 out of 4

#UNDEFINED

TOPKAT_Mouse_Female_FDA_None_vs_Carcinogen

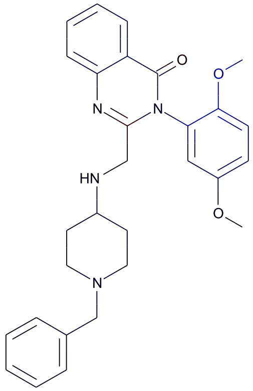

$C_{29}H_{32}N_4O_3$

Molecular Weight: 484.58938

ALogP: 3.743

Rotatable Bonds: 8

Acceptors: 6

Donors: 1

Model Prediction

Prediction: Non-Carcinogen

Probability: 0.205

Enrichment: 0.639

Bayesian Score: -6.71

Mahalanobis Distance: 15.8

Mahalanobis Distance p-value: 7.88e-009

Prediction: Positive if the Bayesian score is above the estimated best cutoff value from minimizing the false positive and false negative rate.
Probability: The esimated probability that the sample is in the positive category. This assumes that the Bayesian score follows a normal distribution and is different from the prediction using a cutoff.
Enrichment: An estimate of enrichment, that is, the increased likelihood (versus random) of this sample being in the category.
Bayesian Score: The standard Laplacian-modified Bayesian score.
Mahalanobis Distance: The Mahalanobis distance (MD) is the distance to the center of the training data. The larger the MD, the less trustworthy the prediction.
Mahalanobis Distance p-value: The p-value gives the fraction of training data with an MD greater than or equal to the one for the given sample, assuming normally distributed data. The smaller the p-value, the less trustworthy the prediciton. For highly non-normal X properties (e.g., fingerprints), the MD p-value is wildly inaccurate.

Structural Similar Compounds

Name	Emetine	Cisapride	Felodipine
Structure	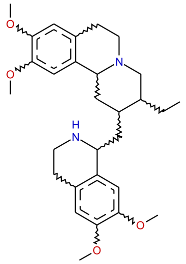	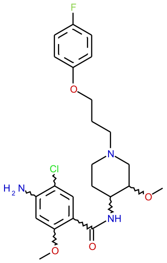	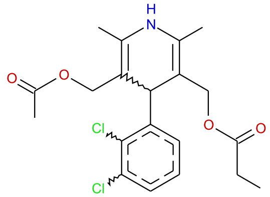
Actual Endpoint	Non-Carcinogen	Non-Carcinogen	Non-Carcinogen
Predicted Endpoint	Non-Carcinogen	Non-Carcinogen	Non-Carcinogen
Distance	0.584	0.618	0.620
Reference	US FDA (Centre for Drug Eval.& Res./Off. Testing & Res.) Sept. 1997	US FDA (Centre for Drug Eval.& Res./Off. Testing & Res.) Sept. 1997	US FDA (Centre for Drug Eval.& Res./Off. Testing & Res.) Sept. 1997

Model Applicability

Unknown features are fingerprint features in the query molecule, but not found or appearing too infrequently in the training set.

1. OPS PC19 out of range. Value: -2.9986. Training min, max, SD, explained variance: -2.8753, 4.0333, 1.285, 0.0168.
2. Unknown ECFP_2 feature: -962771238: [*]C(=[*])N(C(=[*])[*])[c](:[*]):[*]

Feature Contribution

Top features for positive contribution

Fingerprint	Bit/Smiles	Feature Structure	Score	Carcinogen in training set
ECFP_6	769925792	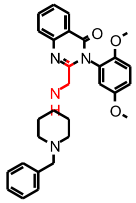 [*]NCC(=[*])[*]	0.617	2 out of 2

ECFP_6	2085698692	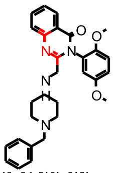 [*]C(=N[c](:[*]):[*])[*]	0.337	3 out of 6
ECFP_6	2106656448	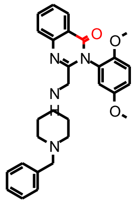 [*]C(=O)[*]	0.254	31 out of 77

Top Features for negative contribution

Fingerprint	Bit/Smiles	Feature Structure	Score	Carcinogen in training set
ECFP_6	2077607946	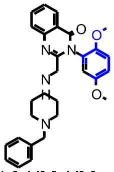 [*]O[c]1:[cH]:[cH]:[c]([*]):[*]:[c]:1[*]	-1.15	0 out of 7
ECFP_6	1307307440	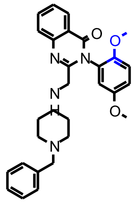 [*]:[c](:[*])OC	-0.558	4 out of 25
ECFP_6	-427397688	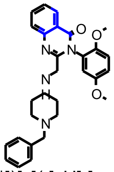 [*]C(=[*])[c](:[cH]:[*]):[c]([*]):[*]	-0.476	5 out of 28

#UNDEFINED

TOPKAT_Mouse_Female_FDA_None_vs_Carcinogen

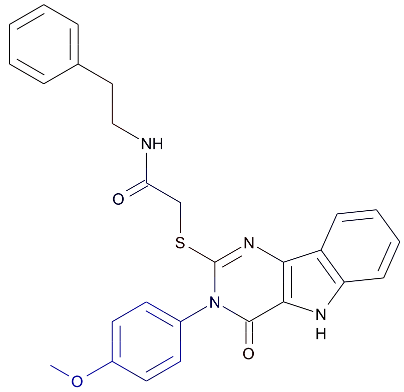

$C_{27}H_{24}N_4O_3S$

Molecular Weight: 484.56946

ALogP: 5.346

Rotatable Bonds: 8

Acceptors: 5

Donors: 2

Model Prediction

Prediction: Non-Carcinogen

Probability: 0.208

Enrichment: 0.648

Bayesian Score: -4.86

Mahalanobis Distance: 13.2

Mahalanobis Distance p-value: 0.000316

Prediction: Positive if the Bayesian score is above the estimated best cutoff value from minimizing the false positive and false negative rate.
Probability: The esimated probability that the sample is in the positive category. This assumes that the Bayesian score follows a normal distribution and is different from the prediction using a cutoff.
Enrichment: An estimate of enrichment, that is, the increased likelihood (versus random) of this sample being in the category.
Bayesian Score: The standard Laplacian-modified Bayesian score.
Mahalanobis Distance: The Mahalanobis distance (MD) is the distance to the center of the training data. The larger the MD, the less trustworthy the prediction.
Mahalanobis Distance p-value: The p-value gives the fraction of training data with an MD greater than or equal to the one for the given sample, assuming normally distributed data. The smaller the p-value, the less trustworthy the prediciton. For highly non-normal X properties (e.g., fingerprints), the MD p-value is wildly inaccurate.

Structural Similar Compounds

Name	Glyburide	Bitolterol	Glimepride
Structure	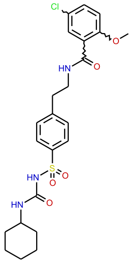	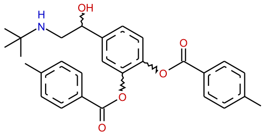	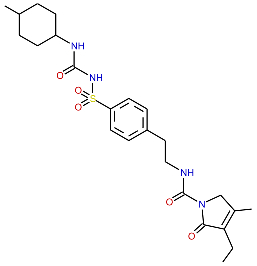
Actual Endpoint	Non-Carcinogen	Non-Carcinogen	Carcinogen
Predicted Endpoint	Non-Carcinogen	Non-Carcinogen	Carcinogen
Distance	0.615	0.641	0.647
Reference	US FDA (Centre for Drug Eval.& Res./Off. Testing & Res.) Sept. 1997	US FDA (Centre for Drug Eval.& Res./Off. Testing & Res.) Sept. 1997	US FDA (Centre for Drug Eval.& Res./Off. Testing & Res.) Sept. 1997

Model Applicability

Unknown features are fingerprint features in the query molecule, but not found or appearing too infrequently in the training set.

1. All properties and OPS components are within expected ranges.
2. Unknown ECFP_2 feature: -962771238: [*]C(=[*])N(C(=[*])[*])[c](:[*]):[*]
3. Unknown ECFP_2 feature: -962137479: [*][c]1:[*]:[*]:[c](:[*]):[c]:1N=[*]
4. Unknown ECFP_2 feature: 676970202: [*]S\C(=N\[*])\N([*])[*]
5. Unknown ECFP_2 feature: 1427820655: [*]CSC(=[*])[*]
6. Unknown ECFP_2 feature: -955816473: [*]SCC(=[*])[*]

Feature Contribution

Top features for positive contribution				
Fingerprint	Bit/Smiles	Feature Structure	Score	Carcinogen in training set

ECFP_6	-830332112	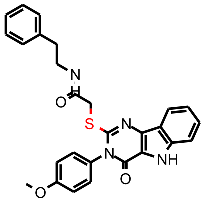 [*]S[*]	0.546	5 out of 8
ECFP_6	2085698692	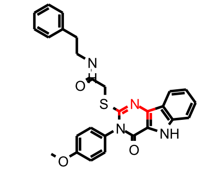 [*]C(=N[c](:[*]):[*]) [*]	0.337	3 out of 6
ECFP_6	-1791034651	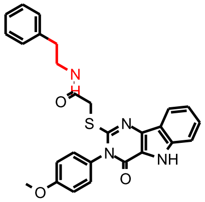 [*]CCN[*]	0.296	7 out of 16
**Top Features for negative contribution**				
**Fingerprint**	**Bit/Smiles**	**Feature Structure**	**Score**	**Carcinogen in training set**
ECFP_6	-175021654	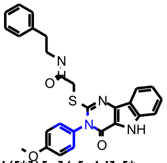 [*]N([*])[c](:[cH]:[*]):[cH]:[*]	-0.805	0 out of 4
ECFP_6	693720869	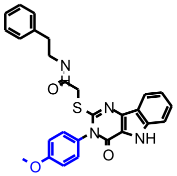 [*][c]1:[cH]:[cH]:[c] (OC):[cH]:[cH]:1	-0.805	0 out of 4

ECFP_6	-1271104377	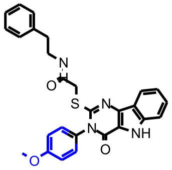 CO[c]1:[cH]:[cH]:[*]:[cH]:[cH]:1	-0.805	0 out of 4

Molecule

TOPKAT_Mouse_Female_NTP

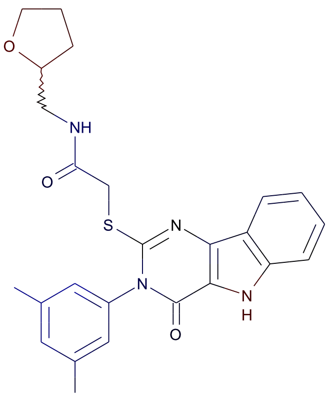

$C_{25}H_{26}N_4O_3S$
Molecular Weight: 462.56393
ALogP: 4.789
Rotatable Bonds: 6
Acceptors: 5
Donors: 2

Structural Similar Compounds

Name	Rhodamine 6G	Curcumin	Lithocholic Acid
Structure	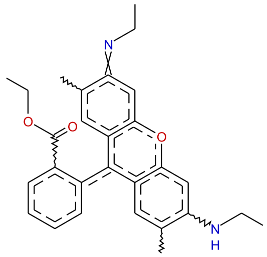	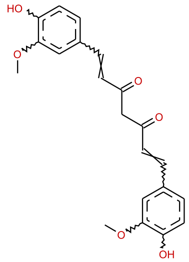	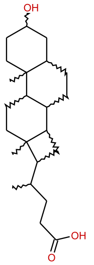
Actual Endpoint	Non-Carcinogen	Carcinogen	Non-Carcinogen
Predicted Endpoint	Non-Carcinogen	Carcinogen	Non-Carcinogen
Distance	0.678	0.698	0.741
Reference	NTP/TR-364	NTP427	NTP/TR-175

Model Prediction

Prediction: Non-Carcinogen
Probability: 0.322
Enrichment: 0.818
Bayesian Score: -5.26
Mahalanobis Distance: 10.8
Mahalanobis Distance p-value: 0.000125

Prediction: Positive if the Bayesian score is above the estimated best cutoff value from minimizing the false positive and false negative rate.
Probability: The esimated probability that the sample is in the positive category. This assumes that the Bayesian score follows a normal distribution and is different from the prediction using a cutoff.
Enrichment: An estimate of enrichment, that is, the increased likelihood (versus random) of this sample being in the category.
Bayesian Score: The standard Laplacian-modified Bayesian score.
Mahalanobis Distance: The Mahalanobis distance (MD) is the distance to the center of the training data. The larger the MD, the less trustworthy the prediction.
Mahalanobis Distance p-value: The p-value gives the fraction of training data with an MD greater than or equal to the one for the given sample, assuming normally distributed data. The smaller the p-value, the less trustworthy the prediciton. For highly non-normal X properties (e.g., fingerprints), the MD p-value is wildly inaccurate.

Model Applicability

Unknown features are fingerprint features in the query molecule, but not found or appearing too infrequently in the training set.

1. OPS PC18 out of range. Value: -3.068. Training min, max, SD, explained variance: -2.8668, 4.1288, 1.043, 0.0181.
2. Unknown ECFP_2 feature: -782828288: [*]C(=[*])[c]1:[nH]:[*]:[*]:[c]:1[*]
3. Unknown ECFP_2 feature: -962771238: [*]C(=[*])N(C(=[*])[*])[c](:[*]):[*]
4. Unknown ECFP_2 feature: -962137479: [*][c]1:[*]:[*]:[c](:[*]):[c]:1N=[*]
5. Unknown ECFP_2 feature: 676970202: [*]S\C(=N\[*])\N([*])[*]
6. Unknown ECFP_2 feature: 2085698692: [*]C(=N[c](:[*]):[*])[*]
7. Unknown ECFP_2 feature: 1427820655: [*]CSC(=[*])[*]
8. Unknown ECFP_2 feature: -84772164: [*]NCC([*])[*]

Feature Contribution

Top features for positive contribution				
**Fingerprint**	**Bit/Smiles**	**Feature Structure**	**Score**	**Carcinogen in training set**

ECFP_8	1099224616	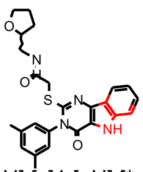 [*]:[cH]:[c]1:[nH]:[*]:[*]:[c]:1:[*]	0.544	2 out of 2
ECFP_8	-1939757055	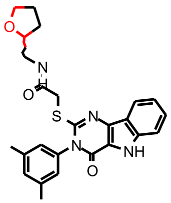 [*]C1[*][*]CO1	0.512	5 out of 7
ECFP_8	-1331450522	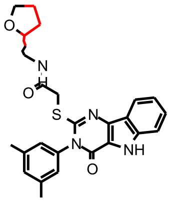 [*]C1[*][*]CC1	0.48	9 out of 14

Top Features for negative contribution

Fingerprint	Bit/Smiles	Feature Structure	Score	Carcinogen in training set
ECFP_8	1731843802	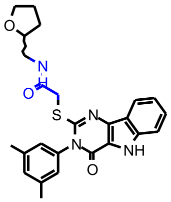 [*]CC(=O)N[*]	-0.909	0 out of 4
ECFP_8	-830332112	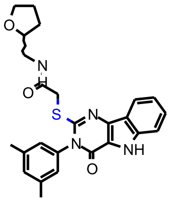 [*]S[*]	-0.856	1 out of 10

ECFP_8	1127062828	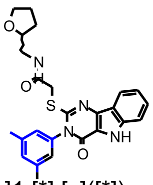 [*][c]1:[*]:[c]([*]):[cH]:[c](C):[cH]:1	-0.748	0 out of 3

#UNDEFINED

TOPKAT_Mouse_Female_NTP

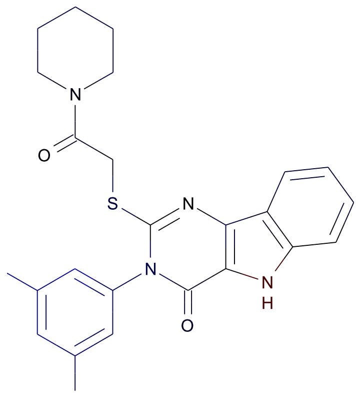

$C_{25}H_{26}N_4O_2S$
Molecular Weight: 446.56453
ALogP: 5.553
Rotatable Bonds: 4
Acceptors: 4
Donors: 1

Model Prediction

Prediction: Non-Carcinogen

Probability: 0.234

Enrichment: 0.595

Bayesian Score: -6.62

Mahalanobis Distance: 11

Mahalanobis Distance p-value: 5.57e-005

Prediction: Positive if the Bayesian score is above the estimated best cutoff value from minimizing the false positive and false negative rate.
Probability: The esimated probability that the sample is in the positive category. This assumes that the Bayesian score follows a normal distribution and is different from the prediction using a cutoff.
Enrichment: An estimate of enrichment, that is, the increased likelihood (versus random) of this sample being in the category.
Bayesian Score: The standard Laplacian-modified Bayesian score.
Mahalanobis Distance: The Mahalanobis distance (MD) is the distance to the center of the training data. The larger the MD, the less trustworthy the prediction.
Mahalanobis Distance p-value: The p-value gives the fraction of training data with an MD greater than or equal to the one for the given sample, assuming normally distributed data. The smaller the p-value, the less trustworthy the prediciton. For highly non-normal X properties (e.g., fingerprints), the MD p-value is wildly inaccurate.

Structural Similar Compounds

Name	Rhodamine 6G	Tricresyl Phosphate	Lithocholic Acid
Structure	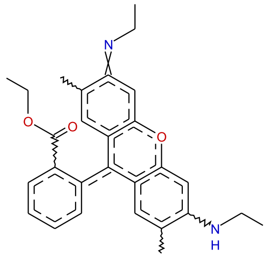	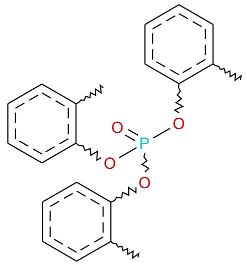	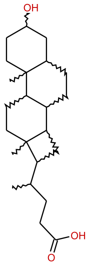
Actual Endpoint	Non-Carcinogen	Non-Carcinogen	Non-Carcinogen
Predicted Endpoint	Non-Carcinogen	Non-Carcinogen	Non-Carcinogen
Distance	0.677	0.707	0.709
Reference	NTP/TR-364	NTP/TR-433	NTP/TR-175

Model Applicability

Unknown features are fingerprint features in the query molecule, but not found or appearing too infrequently in the training set.

1. All properties and OPS components are within expected ranges.
2. Unknown ECFP_2 feature: -782828288: [*]C(=[*])[c]1:[nH]:[*]:[*]:[c]:1[*]
3. Unknown ECFP_2 feature: -962771238: [*]C(=[*])N(C(=[*])[*])[c](:[*]):[*]
4. Unknown ECFP_2 feature: -962137479: [*][c]1:[*]:[*]:[c](:[*]):[c]:1N=[*]
5. Unknown ECFP_2 feature: 676970202: [*]S\C(=N\[*])\N([*])[*]
6. Unknown ECFP_2 feature: 2085698692: [*]C(=N[c](:[*]):[*])[*]
7. Unknown ECFP_2 feature: 1427820655: [*]CSC(=[*])[*]
8. Unknown ECFP_2 feature: 1341750291: [*]CC(=O)N([*])[*]
9. Unknown ECFP_2 feature: -1102925512: [*]CN(C[*])C(=[*])[*]

Feature Contribution

Top features for positive contribution				
Fingerprint	Bit/Smiles	Feature Structure	Score	Carcinogen in training set

ECFP_8	1099224616	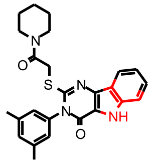 [*]:[cH]:[c]1:[nH]:[*]:[*]:[c]:1:[*]	0.544	2 out of 2
ECFP_8	558201926	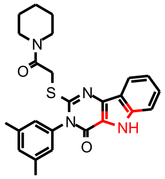 [*][c]1:[*]:[*]:[c](: [*]):[nH]:1	0.378	1 out of 1
ECFP_8	-152683720	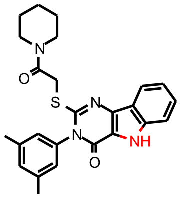 [*]:[nH]:[*]	0.351	2 out of 3
**Top Features for negative contribution**				
**Fingerprint**	**Bit/Smiles**	**Feature Structure**	**Score**	**Carcinogen in training set**
ECFP_8	662850656	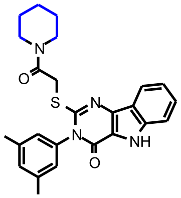 [*]1CCCCC1	-0.909	0 out of 4
ECFP_8	-830332112	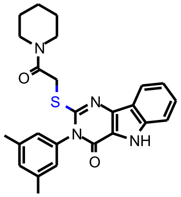 [*]S[*]	-0.856	1 out of 10

ECFP_8	1127062828	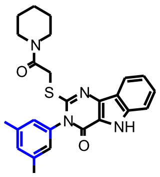<br>[*][c]1:[*]:[c]([*]):<br>[cH]:[c](C):[cH]:1	-0.748	0 out of 3

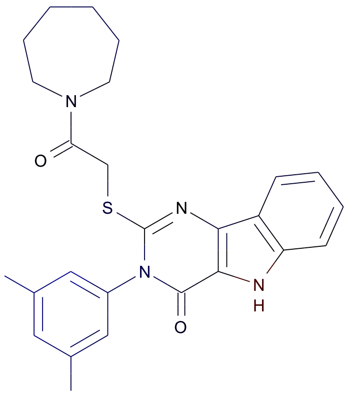

$C_{26}H_{28}N_4O_2S$
Molecular Weight: 460.59111
ALogP: 6.009
Rotatable Bonds: 4
Acceptors: 4
Donors: 1

Model Prediction

Prediction: Non-Carcinogen
Probability: 0.215
Enrichment: 0.546
Bayesian Score: -6.93
Mahalanobis Distance: 11.1
Mahalanobis Distance p-value: 3.97e-005

Prediction: Positive if the Bayesian score is above the estimated best cutoff value from minimizing the false positive and false negative rate.
Probability: The esimated probability that the sample is in the positive category. This assumes that the Bayesian score follows a normal distribution and is different from the prediction using a cutoff.
Enrichment: An estimate of enrichment, that is, the increased likelihood (versus random) of this sample being in the category.
Bayesian Score: The standard Laplacian-modified Bayesian score.
Mahalanobis Distance: The Mahalanobis distance (MD) is the distance to the center of the training data. The larger the MD, the less trustworthy the prediction.
Mahalanobis Distance p-value: The p-value gives the fraction of training data with an MD greater than or equal to the one for the given sample, assuming normally distributed data. The smaller the p-value, the less trustworthy the prediciton. For highly non-normal X properties (e.g., fingerprints), the MD p-value is wildly inaccurate.

Structural Similar Compounds

Name	Rhodamine 6G	Tricresyl Phosphate	4;4'-Thiobis-(6-tert-butyl-m-cresol)
Structure	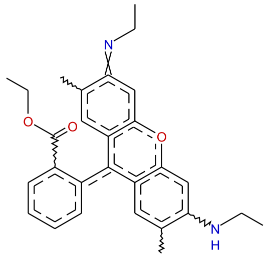	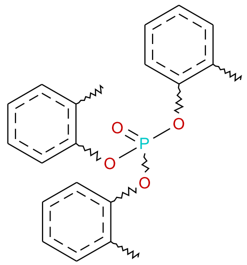	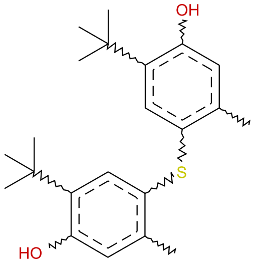
Actual Endpoint	Non-Carcinogen	Non-Carcinogen	Non-Carcinogen
Predicted Endpoint	Non-Carcinogen	Non-Carcinogen	Non-Carcinogen
Distance	0.679	0.707	0.731
Reference	NTP/TR-364	NTP/TR-433	NTP/TR-435

Model Applicability

Unknown features are fingerprint features in the query molecule, but not found or appearing too infrequently in the training set.

1. All properties and OPS components are within expected ranges.
2. Unknown ECFP_2 feature: -782828288: [*]C(=[*])[c]1:[nH]:[*]:[*]:[c]:1[*]
3. Unknown ECFP_2 feature: -962771238: [*]C(=[*])N(C(=[*])[*])[c](:[*]):[*]
4. Unknown ECFP_2 feature: -962137479: [*][c]1:[*]:[*]:[c](:[*]):[c]:1N=[*]
5. Unknown ECFP_2 feature: 676970202: [*]S\C(=N\[*])\N([*])[*]
6. Unknown ECFP_2 feature: 2085698692: [*]C(=N[c](:[*]):[*])[*]
7. Unknown ECFP_2 feature: 1427820655: [*]CSC(=[*])[*]
8. Unknown ECFP_2 feature: 1341750291: [*]CC(=O)N([*])[*]
9. Unknown ECFP_2 feature: -1102925512: [*]CN(C[*])C(=[*])[*]

Feature Contribution

Top features for positive contribution				
Fingerprint	Bit/Smiles	Feature Structure	Score	Carcinogen in training set

ECFP_8	1099224616	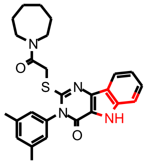 [*]:[cH]:[c]1:[nH]:[*]:[*]:[c]:1:[*]	0.544	2 out of 2
ECFP_8	558201926	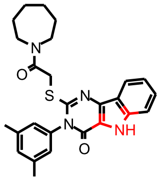 [*][c]1:[*]:[*]:[c](:[*]):[nH]:1	0.378	1 out of 1
ECFP_8	-152683720	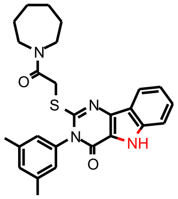 [*]:[nH]:[*]	0.351	2 out of 3
**Top Features for negative contribution**				
**Fingerprint**	**Bit/Smiles**	**Feature Structure**	**Score**	**Carcinogen in training set**
ECFP_8	662850656	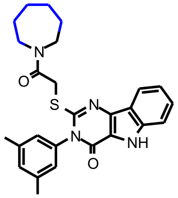 [*]1CCCCC1	-0.909	0 out of 4
ECFP_8	-830332112	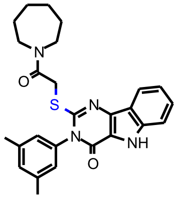 [*]S[*]	-0.856	1 out of 10

ECFP_8	1127062828	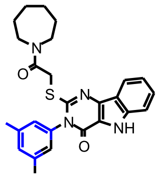 [*][c]1:[*]:[c]([*]):[cH]:[c](C):[cH]:1	-0.748	0 out of 3

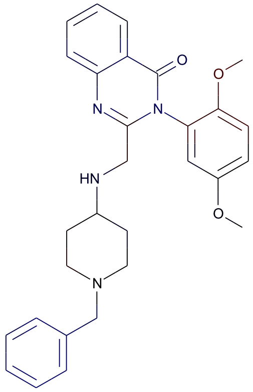

$C_{29}H_{32}N_4O_3$
Molecular Weight: 484.58938
ALogP: 3.743
Rotatable Bonds: 8
Acceptors: 6
Donors: 1

Structural Similar Compounds

Name	Rhodamine 6G	Curcumin	Butylbenzyl phthalate
Structure	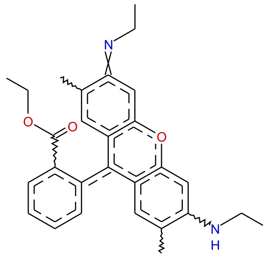	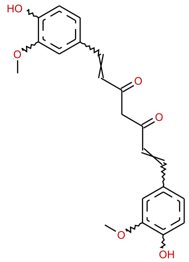	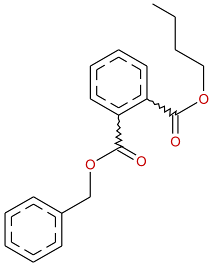
Actual Endpoint	Non-Carcinogen	Carcinogen	Non-Carcinogen
Predicted Endpoint	Non-Carcinogen	Carcinogen	Non-Carcinogen
Distance	0.665	0.714	0.798
Reference	NTP/TR-364	NTP427	NTP/TR-213

Model Prediction

Prediction: Non-Carcinogen
Probability: 0.408
Enrichment: 1.04
Bayesian Score: -3.89
Mahalanobis Distance: 11.7
Mahalanobis Distance p-value: 2.77e-006

Prediction: Positive if the Bayesian score is above the estimated best cutoff value from minimizing the false positive and false negative rate.
Probability: The esimated probability that the sample is in the positive category. This assumes that the Bayesian score follows a normal distribution and is different from the prediction using a cutoff.
Enrichment: An estimate of enrichment, that is, the increased likelihood (versus random) of this sample being in the category.
Bayesian Score: The standard Laplacian-modified Bayesian score.
Mahalanobis Distance: The Mahalanobis distance (MD) is the distance to the center of the training data. The larger the MD, the less trustworthy the prediction.
Mahalanobis Distance p-value: The p-value gives the fraction of training data with an MD greater than or equal to the one for the given sample, assuming normally distributed data. The smaller the p-value, the less trustworthy the prediciton. For highly non-normal X properties (e.g., fingerprints), the MD p-value is wildly inaccurate.

Model Applicability

Unknown features are fingerprint features in the query molecule, but not found or appearing too infrequently in the training set.

1. All properties and OPS components are within expected ranges.
2. Unknown ECFP_2 feature: -962771238: [*]C(=[*])N(C(=[*])[*])[c](:[*]):[*]
3. Unknown ECFP_2 feature: -1073177635: [*]C\C(=N\[*])\N([*])[*]
4. Unknown ECFP_2 feature: 2085698692: [*]C(=N[c](:[*]):[*])[*]
5. Unknown ECFP_2 feature: -2041399277: [*]CN(C[*])C[*]
6. Unknown ECFP_2 feature: 496787418: [*]CNC([*])[*]
7. Unknown ECFP_2 feature: -44121127: [*]N([*])C[c](:[*]):[*]

Feature Contribution

Top features for positive contribution

Fingerprint	Bit/Smiles	Feature Structure	Score	Carcinogen in training set
ECFP_8	-1331450522	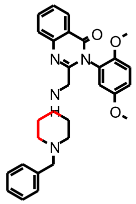 [*]C1[*][*]CC1	0.48	9 out of 14

ECFP_8	1680623188	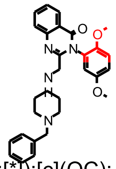 [*][c](:[*]):[c](OC):[cH]:[*]	0.422	9 out of 15
ECFP_8	769925792	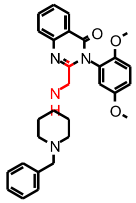 [*]NCC(=[*])[*]	0.378	1 out of 1
**Top Features for negative contribution**				
**Fingerprint**	**Bit/Smiles**	**Feature Structure**	**Score**	**Carcinogen in training set**
ECFP_8	-1650219925	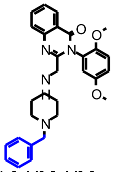 [*]C[c]1:[cH]:[cH]:[cH]:[cH]:[cH]:1	-0.856	1 out of 10
ECFP_8	-1897341097	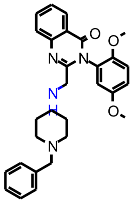 [*]N[*]	-0.555	10 out of 49
ECFP_8	-859078569	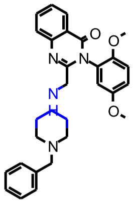 [*]CC(C[*])N[*]	-0.555	0 out of 2

#UNDEFINED

TOPKAT_Mouse_Female_NTP

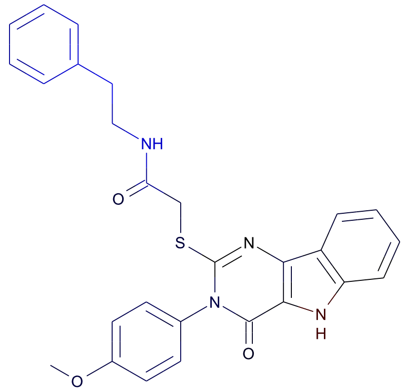

$C_{27}H_{24}N_4O_3S$

Molecular Weight: 484.56946

ALogP: 5.346

Rotatable Bonds: 8

Acceptors: 5

Donors: 2

Model Prediction

Prediction: Non-Carcinogen

Probability: 0.0448

Enrichment: 0.114

Bayesian Score: -11

Mahalanobis Distance: 12.9

Mahalanobis Distance p-value: 3.92e-009

Prediction: Positive if the Bayesian score is above the estimated best cutoff value from minimizing the false positive and false negative rate.
Probability: The esimated probability that the sample is in the positive category. This assumes that the Bayesian score follows a normal distribution and is different from the prediction using a cutoff.
Enrichment: An estimate of enrichment, that is, the increased likelihood (versus random) of this sample being in the category.
Bayesian Score: The standard Laplacian-modified Bayesian score.
Mahalanobis Distance: The Mahalanobis distance (MD) is the distance to the center of the training data. The larger the MD, the less trustworthy the prediction.
Mahalanobis Distance p-value: The p-value gives the fraction of training data with an MD greater than or equal to the one for the given sample, assuming normally distributed data. The smaller the p-value, the less trustworthy the prediciton. For highly non-normal X properties (e.g., fingerprints), the MD p-value is wildly inaccurate.

Structural Similar Compounds

Name	Rhodamine 6G	Curcumin	C.I. pigment red 23
Structure	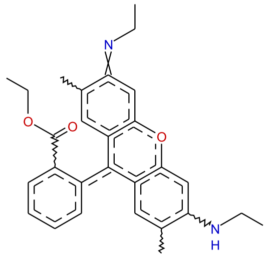	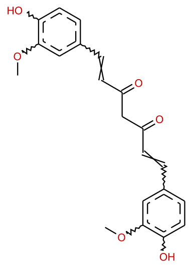	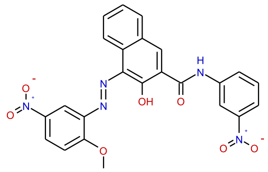
Actual Endpoint	Non-Carcinogen	Carcinogen	Non-Carcinogen
Predicted Endpoint	Non-Carcinogen	Carcinogen	Non-Carcinogen
Distance	0.674	0.714	0.795
Reference	NTP/TR-364	NTP427	NTP411

Model Applicability

Unknown features are fingerprint features in the query molecule, but not found or appearing too infrequently in the training set.

1. All properties and OPS components are within expected ranges.
2. Unknown ECFP_2 feature: -782828288: [*]C(=[*])[c]1:[nH]:[*]:[*]:[c]:1[*]
3. Unknown ECFP_2 feature: -962771238: [*]C(=[*])N(C(=[*])[*])[c](:[*]):[*]
4. Unknown ECFP_2 feature: -962137479: [*][c]1:[*]:[*]:[c](:[*]):[c]:1N=[*]
5. Unknown ECFP_2 feature: 676970202: [*]S\C(=N\[*])\N([*])[*]
6. Unknown ECFP_2 feature: 2085698692: [*]C(=N[c](:[*]):[*])[*]
7. Unknown ECFP_2 feature: 1427820655: [*]CSC(=[*])[*]

Feature Contribution

Top features for positive contribution

Fingerprint	Bit/Smiles	Feature Structure	Score	Carcinogen in training set
ECFP_8	1099224616	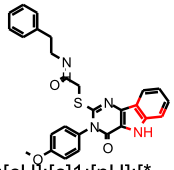 [*]:[cH]:[c]1:[nH]:[*]:[*]:[c]:1:[*]	0.544	2 out of 2

ECFP_8	558201926	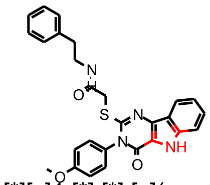 [*][c]1:[*]:[*]:[c](:[*]):[nH]:1	0.378	1 out of 1
ECFP_8	-152683720	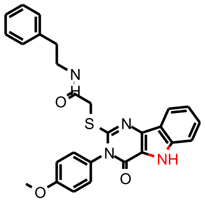 [*]:[nH]:[*]	0.351	2 out of 3

Top Features for negative contribution

Fingerprint	Bit/Smiles	Feature Structure	Score	Carcinogen in training set
ECFP_8	1731843802	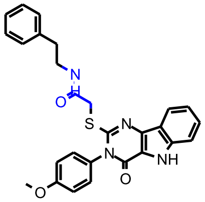 [*]CC(=O)N[*]	-0.909	0 out of 4
ECFP_8	-1650219925	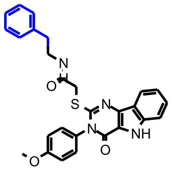 [*]C[c]1:[cH]:[cH]:[cH]:[cH]:[cH]:1	-0.856	1 out of 10
ECFP_8	-830332112	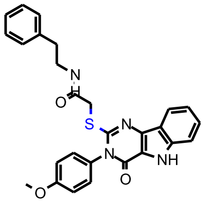 [*]S[*]	-0.856	1 out of 10

Molecule

TOPKAT_Mouse_Male_FDA_None_vs_Carcinogen

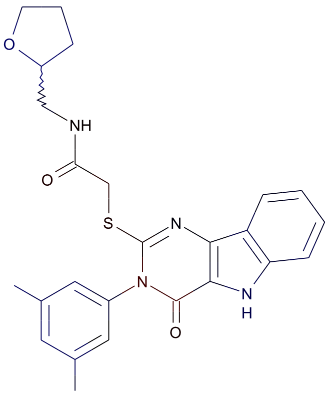

$C_{25}H_{26}N_4O_3S$

Molecular Weight: 462.56393

ALogP: 4.789

Rotatable Bonds: 6

Acceptors: 5

Donors: 2

Structural Similar Compounds

Name	Glimepride	Bicalutamide	Glyburide
Structure	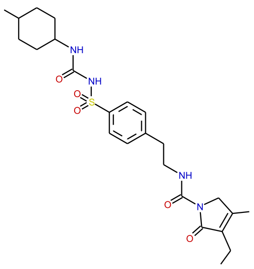	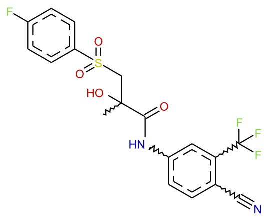	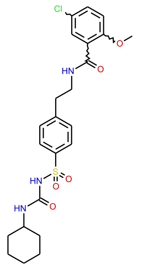
Actual Endpoint	Carcinogen	Carcinogen	Non-Carcinogen
Predicted Endpoint	Carcinogen	Carcinogen	Non-Carcinogen
Distance	0.645	0.651	0.661
Reference	US FDA (Centre for Drug Eval.& Res./Off. Testing & Res.) Sept. 1997	US FDA (Centre for Drug Eval.& Res./Off. Testing & Res.) Sept. 1997	US FDA (Centre for Drug Eval.& Res./Off. Testing & Res.) Sept. 1997

Model Prediction

Prediction: Non-Carcinogen

Probability: 0.2

Enrichment: 0.681

Bayesian Score: -4.95

Mahalanobis Distance: 13.9

Mahalanobis Distance p-value: 8.87e-006

Prediction: Positive if the Bayesian score is above the estimated best cutoff value from minimizing the false positive and false negative rate.
Probability: The esimated probability that the sample is in the positive category. This assumes that the Bayesian score follows a normal distribution and is different from the prediction using a cutoff.
Enrichment: An estimate of enrichment, that is, the increased likelihood (versus random) of this sample being in the category.
Bayesian Score: The standard Laplacian-modified Bayesian score.
Mahalanobis Distance: The Mahalanobis distance (MD) is the distance to the center of the training data. The larger the MD, the less trustworthy the prediction.
Mahalanobis Distance p-value: The p-value gives the fraction of training data with an MD greater than or equal to the one for the given sample, assuming normally distributed data. The smaller the p-value, the less trustworthy the prediciton. For highly non-normal X properties (e.g., fingerprints), the MD p-value is wildly inaccurate.

Model Applicability

Unknown features are fingerprint features in the query molecule, but not found or appearing too infrequently in the training set.

1. All properties and OPS components are within expected ranges.

Feature Contribution

Top features for positive contribution

Fingerprint	Bit/Smiles	Feature Structure	Score	Carcinogen in training set
FCFP_6	-387072142	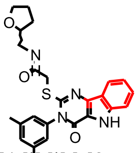 [*][c]1:[*]:[*]:[c]2:[cH]:[cH]:[cH]:[cH]:[c]:1:2	0.477	4 out of 8

FCFP_6	566058135	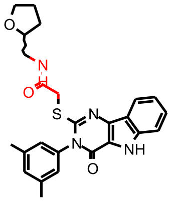 [*]CC(=O)N[*]	0.447	17 out of 40
FCFP_6	-776001689	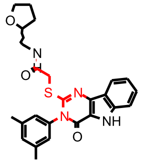 [*]\N=C(\SCC(=[*])[*])/N([*])[*]	0.38	2 out of 4

Top Features for negative contribution

Fingerprint	Bit/Smiles	Feature Structure	Score	Carcinogen in training set
FCFP_6	-1773728142	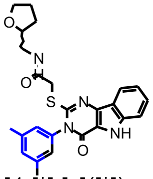 [*][c]1:[*]:[c]([*]):[cH]:[c](C):[cH]:1	-1.29	0 out of 10
FCFP_6	-98332825	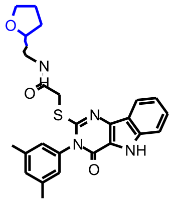 [*]C1CCCO1	-0.793	1 out of 13
FCFP_6	-451251206	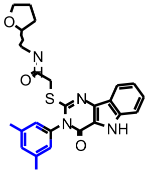 C[c]1:[cH]:[*]:[cH]:[c](C):[cH]:1	-0.731	1 out of 12

#UNDEFINED

TOPKAT_Mouse_Male_FDA_None_vs_Carcinogen

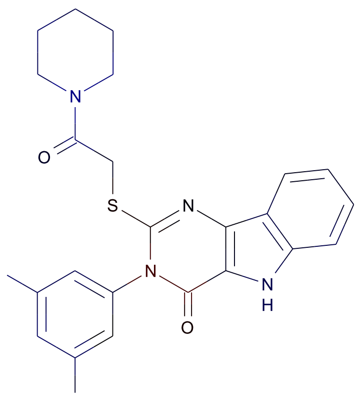

$C_{25}H_{26}N_4O_2S$

Molecular Weight: 446.56453

ALogP: 5.553

Rotatable Bonds: 4

Acceptors: 4

Donors: 1

Model Prediction

Prediction: Non-Carcinogen

Probability: 0.198

Enrichment: 0.673

Bayesian Score: -5.09

Mahalanobis Distance: 14.5

Mahalanobis Distance p-value: 7.12e-007

Prediction: Positive if the Bayesian score is above the estimated best cutoff value from minimizing the false positive and false negative rate.
Probability: The esimated probability that the sample is in the positive category. This assumes that the Bayesian score follows a normal distribution and is different from the prediction using a cutoff.
Enrichment: An estimate of enrichment, that is, the increased likelihood (versus random) of this sample being in the category.
Bayesian Score: The standard Laplacian-modified Bayesian score.
Mahalanobis Distance: The Mahalanobis distance (MD) is the distance to the center of the training data. The larger the MD, the less trustworthy the prediction.
Mahalanobis Distance p-value: The p-value gives the fraction of training data with an MD greater than or equal to the one for the given sample, assuming normally distributed data. The smaller the p-value, the less trustworthy the prediciton. For highly non-normal X properties (e.g., fingerprints), the MD p-value is wildly inaccurate.

Structural Similar Compounds

Name	Indomethacin	Ethynodiol	Lansoprazole
Structure	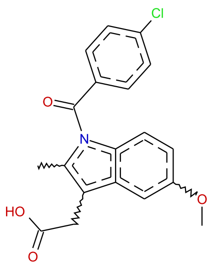	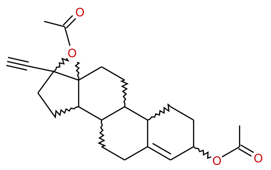	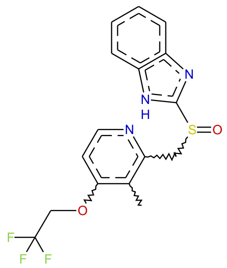
Actual Endpoint	Non-Carcinogen	Carcinogen	Carcinogen
Predicted Endpoint	Non-Carcinogen	Carcinogen	Carcinogen
Distance	0.657	0.683	0.705
Reference	US FDA (Centre for Drug Eval.& Res./Off. Testing & Res.) Sept. 1997	US FDA (Centre for Drug Eval.& Res./Off. Testing & Res.) Sept. 1997	US FDA (Centre for Drug Eval.& Res./Off. Testing & Res.) Sept. 1997

Model Applicability

Unknown features are fingerprint features in the query molecule, but not found or appearing too infrequently in the training set.

1. All properties and OPS components are within expected ranges.

Feature Contribution

Top features for positive contribution				
**Fingerprint**	**Bit/Smiles**	**Feature Structure**	**Score**	**Carcinogen in training set**
FCFP_6	-387072142	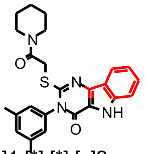 [*][c]1:[*]:[*]:[c]2:[cH]:[cH]:[cH]:[cH]:[c]:1:2	0.477	4 out of 8

FCFP_6	-776001689	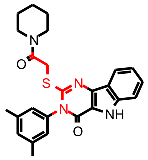 [*]\N=C(\SCC(=[*])[*])/N([*])[*]	0.38	2 out of 4
FCFP_6	203707511	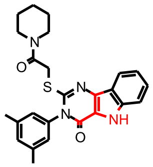 [*]C(=[*])[c]1:[nH]:[*]:[*]:[c]:1[*]	0.38	2 out of 4

Top Features for negative contribution

Fingerprint	Bit/Smiles	Feature Structure	Score	Carcinogen in training set
FCFP_6	-1773728142	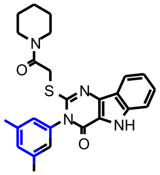 [*][c]1:[*]:[c]([*]): [cH]:[c](C):[cH]:1	-1.29	0 out of 10
FCFP_6	-98332825	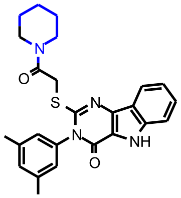 [*]C1CCCO1	-0.793	1 out of 13
FCFP_6	-451251206	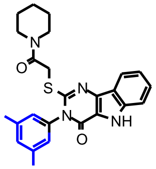 C[c]1:[cH]:[*]:[cH]:[c](C):[cH]:1	-0.731	1 out of 12

#UNDEFINED

TOPKAT_Mouse_Male_FDA_None_vs_Carcinogen

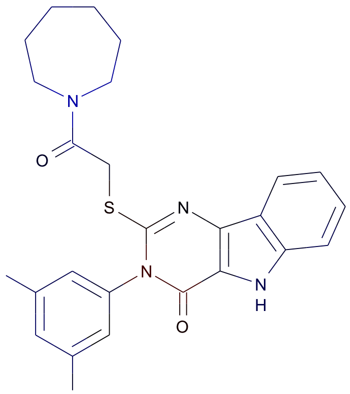

$C_{26}H_{28}N_4O_2S$
Molecular Weight: 460.59111
ALogP: 6.009
Rotatable Bonds: 4
Acceptors: 4
Donors: 1

Structural Similar Compounds

Name	Indomethacin	Ethynodiol	Quazepam
Structure	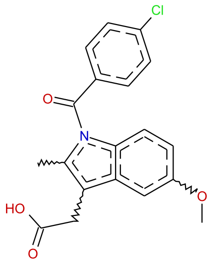	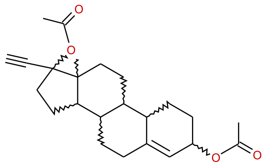	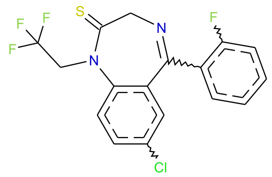
Actual Endpoint	Non-Carcinogen	Carcinogen	Non-Carcinogen
Predicted Endpoint	Non-Carcinogen	Carcinogen	Non-Carcinogen
Distance	0.702	0.706	0.724
Reference	US FDA (Centre for Drug Eval.& Res./Off. Testing & Res.) Sept. 1997	US FDA (Centre for Drug Eval.& Res./Off. Testing & Res.) Sept. 1997	US FDA (Centre for Drug Eval.& Res./Off. Testing & Res.) Sept. 1997

Model Prediction

Prediction: Non-Carcinogen
Probability: 0.184
Enrichment: 0.624
Bayesian Score: -6.09
Mahalanobis Distance: 14.6
Mahalanobis Distance p-value: 4.33e-007

Prediction: Positive if the Bayesian score is above the estimated best cutoff value from minimizing the false positive and false negative rate.
Probability: The esimated probability that the sample is in the positive category. This assumes that the Bayesian score follows a normal distribution and is different from the prediction using a cutoff.
Enrichment: An estimate of enrichment, that is, the increased likelihood (versus random) of this sample being in the category.
Bayesian Score: The standard Laplacian-modified Bayesian score.
Mahalanobis Distance: The Mahalanobis distance (MD) is the distance to the center of the training data. The larger the MD, the less trustworthy the prediction.
Mahalanobis Distance p-value: The p-value gives the fraction of training data with an MD greater than or equal to the one for the given sample, assuming normally distributed data. The smaller the p-value, the less trustworthy the prediciton. For highly non-normal X properties (e.g., fingerprints), the MD p-value is wildly inaccurate.

Model Applicability

Unknown features are fingerprint features in the query molecule, but not found or appearing too infrequently in the training set.

1. All properties and OPS components are within expected ranges.

Feature Contribution

Top features for positive contribution				
**Fingerprint**	**Bit/Smiles**	**Feature Structure**	**Score**	**Carcinogen in training set**
FCFP_6	-387072142	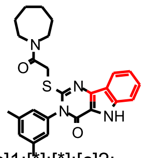 [*][c]1:[*]:[*]:[c]2:[cH]:[cH]:[cH]:[cH]:[c]:1:2	0.477	4 out of 8

FCFP_6	203707511	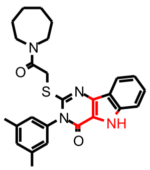 [*]C(=[*])[c]1:[nH]:[*]:[*]:[c]:1[*]	0.38	2 out of 4
FCFP_6	-776001689	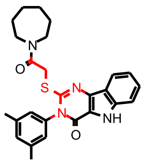 [*]\N=C(\SCC(=[*])[*])/N([*])[*]	0.38	2 out of 4

Top Features for negative contribution

Fingerprint	Bit/Smiles	Feature Structure	Score	Carcinogen in training set
FCFP_6	-1773728142	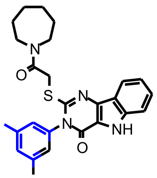 [*][c]1:[*]:[c]([*]): [cH]:[c](C):[cH]:1	-1.29	0 out of 10
FCFP_6	-98332825	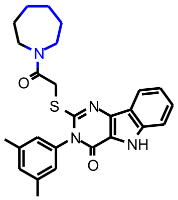 [*]C1CCCO1	-0.793	1 out of 13
FCFP_6	-451251206	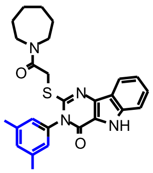 C[c]1:[cH]:[*]:[cH]:[c](C):[cH]:1	-0.731	1 out of 12

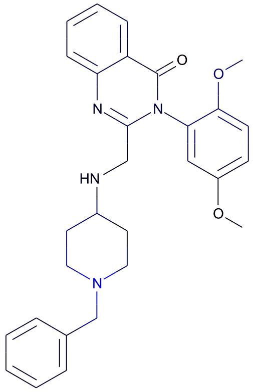

$C_{29}H_{32}N_4O_3$
Molecular Weight: 484.58938
ALogP: 3.743
Rotatable Bonds: 8
Acceptors: 6
Donors: 1

Model Prediction

Prediction: Non-Carcinogen
Probability: 0.152
Enrichment: 0.515
Bayesian Score: -8.81
Mahalanobis Distance: 14.3
Mahalanobis Distance p-value: 2.02e-006

Prediction: Positive if the Bayesian score is above the estimated best cutoff value from minimizing the false positive and false negative rate.
Probability: The esimated probability that the sample is in the positive category. This assumes that the Bayesian score follows a normal distribution and is different from the prediction using a cutoff.
Enrichment: An estimate of enrichment, that is, the increased likelihood (versus random) of this sample being in the category.
Bayesian Score: The standard Laplacian-modified Bayesian score.
Mahalanobis Distance: The Mahalanobis distance (MD) is the distance to the center of the training data. The larger the MD, the less trustworthy the prediction.
Mahalanobis Distance p-value: The p-value gives the fraction of training data with an MD greater than or equal to the one for the given sample, assuming normally distributed data. The smaller the p-value, the less trustworthy the prediciton. For highly non-normal X properties (e.g., fingerprints), the MD p-value is wildly inaccurate.

Structural Similar Compounds

Name	Emetine	Felodipine	Cisapride
Structure	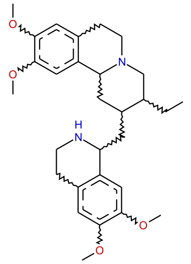	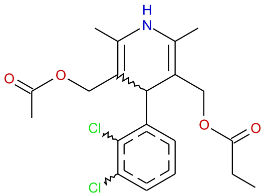	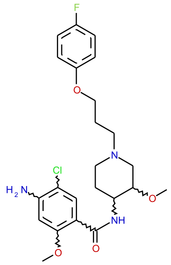
Actual Endpoint	Non-Carcinogen	Non-Carcinogen	Non-Carcinogen
Predicted Endpoint	Non-Carcinogen	Non-Carcinogen	Non-Carcinogen
Distance	0.558	0.596	0.602
Reference	US FDA (Centre for Drug Eval.& Res./Off. Testing & Res.) Sept. 1997	US FDA (Centre for Drug Eval.& Res./Off. Testing & Res.) Sept. 1997	US FDA (Centre for Drug Eval.& Res./Off. Testing & Res.) Sept. 1997

Model Applicability

Unknown features are fingerprint features in the query molecule, but not found or appearing too infrequently in the training set.

1. OPS PC22 out of range. Value: -3.3253. Training min, max, SD, explained variance: -2.9663, 3.4535, 1.118, 0.0131.

Feature Contribution

Top features for positive contribution

Fingerprint	Bit/Smiles	Feature Structure	Score	Carcinogen in training set
FCFP_6	1679744180	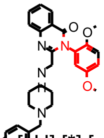 [*]O[c]1:[cH]:[*]:[c]([*]):[c](:[cH]:1)N([*])[*]	0.271	1 out of 2

FCFP_6	-796673622	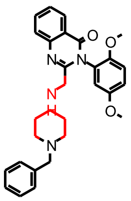 [*]CNC1CC[*]CC1	0.259	2 out of 5
FCFP_6	1872154524	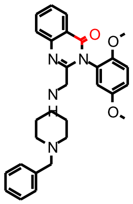 [*]C(=O)[*]	0.205	69 out of 213

Top Features for negative contribution

**Fingerprint**	**Bit/Smiles**	**Feature Structure**	**Score**	**Carcinogen in training set**
FCFP_6	309602933	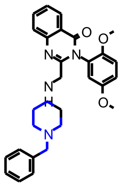 [*]CN1C[*]C([*])CC1	-1.4	1 out of 27
FCFP_6	1028934530	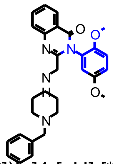 [*]N([*])[c]1:[cH]:[*]:[cH]:[cH]:[c]:1OC	-0.596	1 out of 10
FCFP_6	356782498	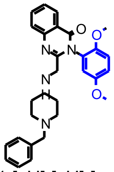 [*]O[c]1:[cH]:[cH]:[c](OC):[cH]:[c]:1[*]	-0.582	0 out of 3

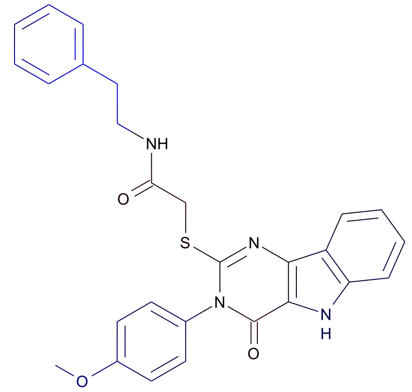

$C_{27}H_{24}N_4O_3S$
Molecular Weight: 484.56946
ALogP: 5.346
Rotatable Bonds: 8
Acceptors: 5
Donors: 2

Model Prediction

Prediction: Non-Carcinogen
Probability: 0.165
Enrichment: 0.56
Bayesian Score: -7.56
Mahalanobis Distance: 13.3
Mahalanobis Distance p-value: 8.8e-005

Prediction: Positive if the Bayesian score is above the estimated best cutoff value from minimizing the false positive and false negative rate.
Probability: The esimated probability that the sample is in the positive category. This assumes that the Bayesian score follows a normal distribution and is different from the prediction using a cutoff.
Enrichment: An estimate of enrichment, that is, the increased likelihood (versus random) of this sample being in the category.
Bayesian Score: The standard Laplacian-modified Bayesian score.
Mahalanobis Distance: The Mahalanobis distance (MD) is the distance to the center of the training data. The larger the MD, the less trustworthy the prediction.
Mahalanobis Distance p-value: The p-value gives the fraction of training data with an MD greater than or equal to the one for the given sample, assuming normally distributed data. The smaller the p-value, the less trustworthy the prediciton. For highly non-normal X properties (e.g., fingerprints), the MD p-value is wildly inaccurate.

Structural Similar Compounds

Name	Glyburide	Bitolterol	Glimepride
Structure	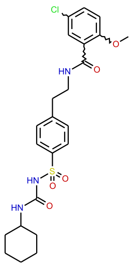	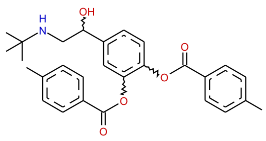	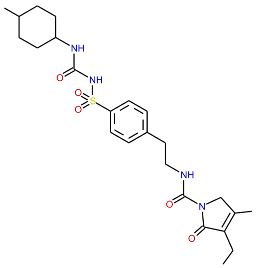
Actual Endpoint	Non-Carcinogen	Non-Carcinogen	Carcinogen
Predicted Endpoint	Non-Carcinogen	Non-Carcinogen	Carcinogen
Distance	0.594	0.612	0.645
Reference	US FDA (Centre for Drug Eval.& Res./Off. Testing & Res.) Sept. 1997	US FDA (Centre for Drug Eval.& Res./Off. Testing & Res.) Sept. 1997	US FDA (Centre for Drug Eval.& Res./Off. Testing & Res.) Sept. 1997

Model Applicability

Unknown features are fingerprint features in the query molecule, but not found or appearing too infrequently in the training set.

1. All properties and OPS components are within expected ranges.

Feature Contribution

Top features for positive contribution

Fingerprint	Bit/Smiles	Feature Structure	Score	Carcinogen in training set
FCFP_6	-387072142	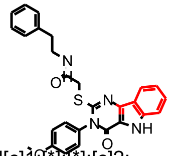 [*][c]1c[*]:[*]:[c]2:[cH]:[cH]:[cH]:[cH]:[c]:1:2	0.477	4 out of 8

FCFP_6	566058135	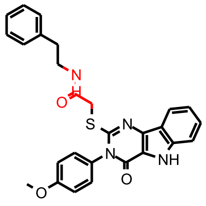 [*]CC(=O)N[*]	0.447	17 out of 40
FCFP_6	-776001689	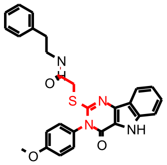 [*]\N=C(\SCC(=[*])[*])/N([*])[*]	0.38	2 out of 4

Top Features for negative contribution

Fingerprint	Bit/Smiles	Feature Structure	Score	Carcinogen in training set
FCFP_6	1981711554	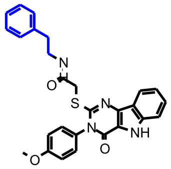 [*]CC[c]1:[cH]:[cH]:[cH]:[cH]:[cH]:1	-1.42	0 out of 12
FCFP_6	-497728148	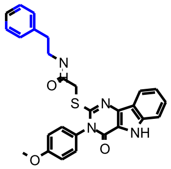 [*]CC[c]1:[cH]:[cH]:[*]:[cH]:[cH]:1	-0.96	2 out of 26
FCFP_6	-9847677	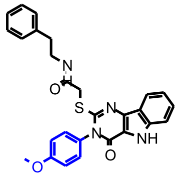 [*][c]1:[cH]:[cH]:[c] (OC):[cH]:[cH]:1	-0.719	0 out of 4

Molecule

TOPKAT_Mouse_Male_NTP

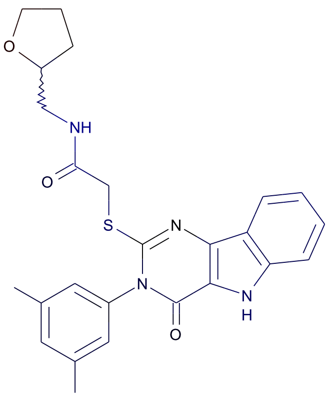

$C_{25}H_{26}N_4O_3S$
Molecular Weight: 462.56393
ALogP: 4.789
Rotatable Bonds: 6
Acceptors: 5
Donors: 2

Structural Similar Compounds

Name	Curcumin	RHODAMINE 6G	C.I.PIGMENT RED 3
Structure	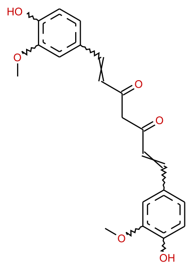	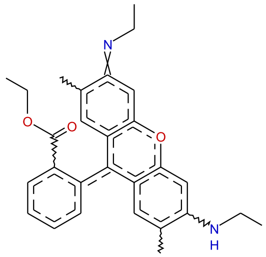	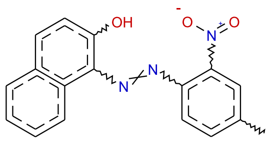
Actual Endpoint	Carcinogen	Non-Carcinogen	Carcinogen
Predicted Endpoint	Carcinogen	Non-Carcinogen	Carcinogen
Distance	0.700	0.738	0.775
Reference	NTP427	NTP/TR-364	NTP/TR-407

Model Prediction

Prediction: Non-Carcinogen
Probability: 0.0274
Enrichment: 0.0696
Bayesian Score: -8.89
Mahalanobis Distance: 13
Mahalanobis Distance p-value: 1.15e-008

Prediction: Positive if the Bayesian score is above the estimated best cutoff value from minimizing the false positive and false negative rate.
Probability: The esimated probability that the sample is in the positive category. This assumes that the Bayesian score follows a normal distribution and is different from the prediction using a cutoff.
Enrichment: An estimate of enrichment, that is, the increased likelihood (versus random) of this sample being in the category.
Bayesian Score: The standard Laplacian-modified Bayesian score.
Mahalanobis Distance: The Mahalanobis distance (MD) is the distance to the center of the training data. The larger the MD, the less trustworthy the prediction.
Mahalanobis Distance p-value: The p-value gives the fraction of training data with an MD greater than or equal to the one for the given sample, assuming normally distributed data. The smaller the p-value, the less trustworthy the prediciton. For highly non-normal X properties (e.g., fingerprints), the MD p-value is wildly inaccurate.

Model Applicability

Unknown features are fingerprint features in the query molecule, but not found or appearing too infrequently in the training set.

1. OPS PC11 out of range. Value: 3.4549. Training min, max, SD, explained variance: -3.6964, 3.2756, 1.387, 0.0309.
2. OPS PC16 out of range. Value: -3.4058. Training min, max, SD, explained variance: -2.7666, 3.8549, 1.117, 0.0200.

Feature Contribution

Top features for positive contribution

Fingerprint	Bit/Smiles	Feature Structure	Score	Carcinogen in training set
SCFP_12	-1905025356	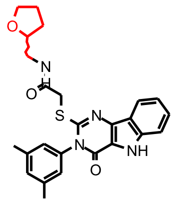 [*]CC1CCCO1	0.337	3 out of 5

SCFP_12	-711686199	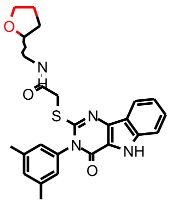 [*]1[*]OCC1	0.243	23 out of 48
SCFP_12	276193969	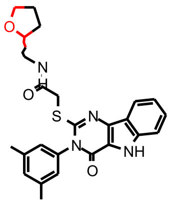 [*]C1[*][*]CO1	0.182	10 out of 22
**Top Features for negative contribution**				
**Fingerprint**	**Bit/Smiles**	**Feature Structure**	**Score**	**Carcinogen in training set**
SCFP_12	17	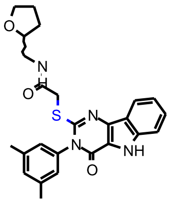 [*]S[*]	-1.24	1 out of 16
SCFP_12	-587569116	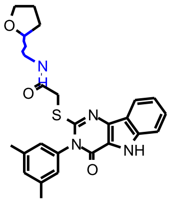 [*]NCC([*])[*]	-0.91	0 out of 4
SCFP_12	-111024397	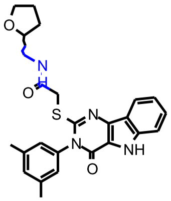 [*]CNC(=[*])[*]	-0.784	2 out of 15

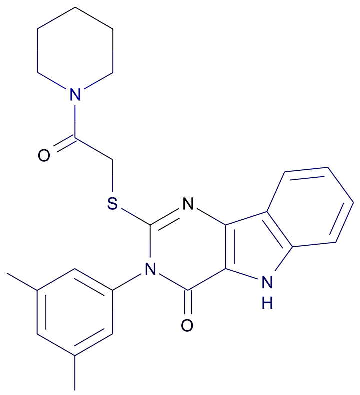

$C_{25}H_{26}N_4O_2S$
Molecular Weight: 446.56453
ALogP: 5.553
Rotatable Bonds: 4
Acceptors: 4
Donors: 1

Model Prediction

Prediction: Non-Carcinogen
Probability: 0.0193
Enrichment: 0.0491
Bayesian Score: -9.3
Mahalanobis Distance: 11.2
Mahalanobis Distance p-value: 7.82e-005

Prediction: Positive if the Bayesian score is above the estimated best cutoff value from minimizing the false positive and false negative rate.
Probability: The esimated probability that the sample is in the positive category. This assumes that the Bayesian score follows a normal distribution and is different from the prediction using a cutoff.
Enrichment: An estimate of enrichment, that is, the increased likelihood (versus random) of this sample being in the category.
Bayesian Score: The standard Laplacian-modified Bayesian score.
Mahalanobis Distance: The Mahalanobis distance (MD) is the distance to the center of the training data. The larger the MD, the less trustworthy the prediction.
Mahalanobis Distance p-value: The p-value gives the fraction of training data with an MD greater than or equal to the one for the given sample, assuming normally distributed data. The smaller the p-value, the less trustworthy the prediciton. For highly non-normal X properties (e.g., fingerprints), the MD p-value is wildly inaccurate.

Structural Similar Compounds

Name	C.I.PIGMENT RED 3	C.I. Pigment Red 3	RHODAMINE 6G
Structure	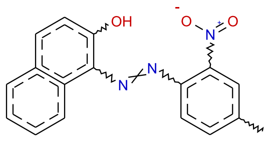	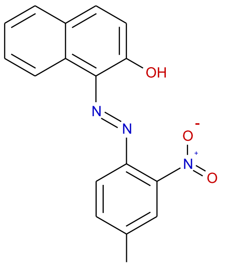	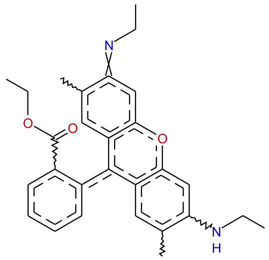
Actual Endpoint	Carcinogen	Carcinogen	Non-Carcinogen
Predicted Endpoint	Carcinogen	Carcinogen	Non-Carcinogen
Distance	0.690	0.690	0.706
Reference	NTP/TR-407	NTP407	NTP/TR-364

Model Applicability

Unknown features are fingerprint features in the query molecule, but not found or appearing too infrequently in the training set.

1. OPS PC11 out of range. Value: 4.2037. Training min, max, SD, explained variance: -3.6964, 3.2756, 1.387, 0.0309.

Feature Contribution

Top features for positive contribution				
**Fingerprint**	**Bit/Smiles**	**Feature Structure**	**Score**	**Carcinogen in training set**
SCFP_12	1175638033	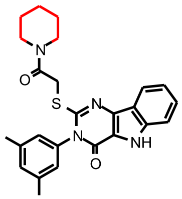 [*]1CCCCC1	0.241	5 out of 10

SCFP_12	622342378	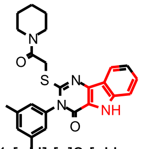<br>[*][c]1:[nH]:[c]2:[cH]:[cH]:[*]:[cH]:[c]:2:[c]:1[*]	0.138	1 out of 2
SCFP_12	112554633	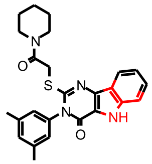<br>[*]:[cH]:[c]1:[nH]:[*]:[*]:[c]:1:[*]	0.106	3 out of 7

Top Features for negative contribution

Fingerprint	Bit/Smiles	Feature Structure	Score	Carcinogen in training set
SCFP_12	17	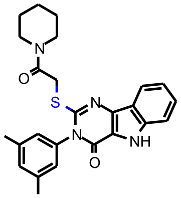<br>[*]S[*]	-1.24	1 out of 16
SCFP_12	-1343150366	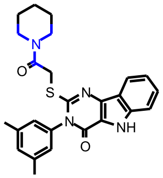<br>[*]CN(C[*])C(=[*])[*]	-1.05	0 out of 5
SCFP_12	-587569116	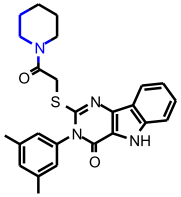<br>[*]NCC([*])[*]	-0.91	0 out of 4

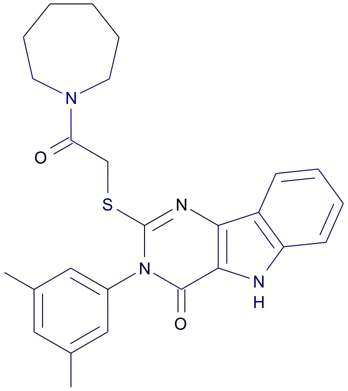

$C_{26}H_{28}N_4O_2S$
Molecular Weight: 460.59111
ALogP: 6.009
Rotatable Bonds: 4
Acceptors: 4
Donors: 1

Model Prediction

Prediction: Non-Carcinogen
Probability: 0.0193
Enrichment: 0.0491
Bayesian Score: -9.3
Mahalanobis Distance: 11.2
Mahalanobis Distance p-value: 5.91e-005

Prediction: Positive if the Bayesian score is above the estimated best cutoff value from minimizing the false positive and false negative rate.
Probability: The esimated probability that the sample is in the positive category. This assumes that the Bayesian score follows a normal distribution and is different from the prediction using a cutoff.
Enrichment: An estimate of enrichment, that is, the increased likelihood (versus random) of this sample being in the category.
Bayesian Score: The standard Laplacian-modified Bayesian score.
Mahalanobis Distance: The Mahalanobis distance (MD) is the distance to the center of the training data. The larger the MD, the less trustworthy the prediction.
Mahalanobis Distance p-value: The p-value gives the fraction of training data with an MD greater than or equal to the one for the given sample, assuming normally distributed data. The smaller the p-value, the less trustworthy the prediciton. For highly non-normal X properties (e.g., fingerprints), the MD p-value is wildly inaccurate.

Structural Similar Compounds

Name	RHODAMINE 6G	C.I.PIGMENT RED 3	C.I. Pigment Red 3
Structure	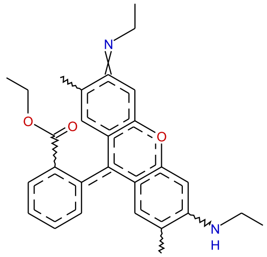	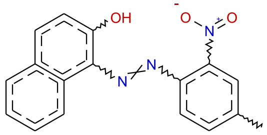	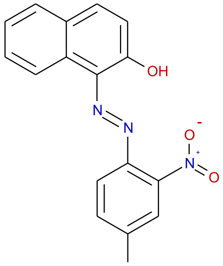
Actual Endpoint	Non-Carcinogen	Carcinogen	Carcinogen
Predicted Endpoint	Non-Carcinogen	Carcinogen	Carcinogen
Distance	0.709	0.717	0.717
Reference	NTP/TR-364	NTP/TR-407	NTP407

Model Applicability

Unknown features are fingerprint features in the query molecule, but not found or appearing too infrequently in the training set.

1. OPS PC11 out of range. Value: 4.208. Training min, max, SD, explained variance: -3.6964, 3.2756, 1.387, 0.0309.

Feature Contribution

Top features for positive contribution

Fingerprint	Bit/Smiles	Feature Structure	Score	Carcinogen in training set
SCFP_12	1175638033	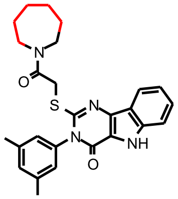 [*]1CCCCC1	0.241	5 out of 10

SCFP_12	622342378	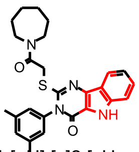 [*][c]1:[nH]:[c]2:[cH]:[cH]:[*]:[cH]:[c]:2:[c]:1[*]	0.138	1 out of 2
SCFP_12	112554633	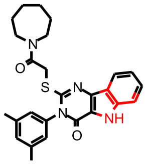 [*]:[cH]:[c]1:[nH]:[*]:[*]:[c]:1:[*]	0.106	3 out of 7

Top Features for negative contribution

Fingerprint	Bit/Smiles	Feature Structure	Score	Carcinogen in training set
SCFP_12	17	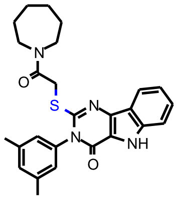 [*]S[*]	-1.24	1 out of 16
SCFP_12	-1343150366	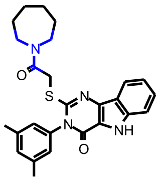 [*]CN(C[*])C(=[*])[*]	-1.05	0 out of 5
SCFP_12	-587569116	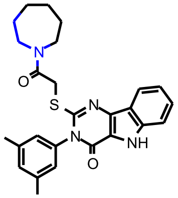 [*]NCC([*])[*]	-0.91	0 out of 4

#UNDEFINED

TOPKAT_Mouse_Male_NTP

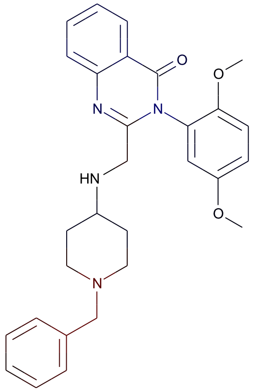

$C_{29}H_{32}N_4O_3$
Molecular Weight: 484.58938
ALogP: 3.743
Rotatable Bonds: 8
Acceptors: 6
Donors: 1

Model Prediction

Prediction: Non-Carcinogen
Probability: 0.531
Enrichment: 1.35
Bayesian Score: -1.85
Mahalanobis Distance: 12.1
Mahalanobis Distance p-value: 1.09e-006

Prediction: Positive if the Bayesian score is above the estimated best cutoff value from minimizing the false positive and false negative rate.
Probability: The esimated probability that the sample is in the positive category. This assumes that the Bayesian score follows a normal distribution and is different from the prediction using a cutoff.
Enrichment: An estimate of enrichment, that is, the increased likelihood (versus random) of this sample being in the category.
Bayesian Score: The standard Laplacian-modified Bayesian score.
Mahalanobis Distance: The Mahalanobis distance (MD) is the distance to the center of the training data. The larger the MD, the less trustworthy the prediction.
Mahalanobis Distance p-value: The p-value gives the fraction of training data with an MD greater than or equal to the one for the given sample, assuming normally distributed data. The smaller the p-value, the less trustworthy the prediciton. For highly non-normal X properties (e.g., fingerprints), the MD p-value is wildly inaccurate.

Structural Similar Compounds

Name	RHODAMINE 6G	Curcumin	BUTYL BENZYL PHTHALATE
Structure	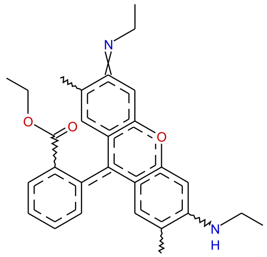	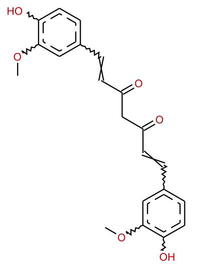	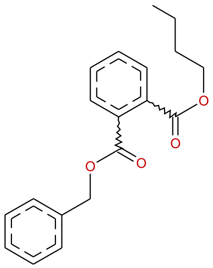
Actual Endpoint	Non-Carcinogen	Carcinogen	Non-Carcinogen
Predicted Endpoint	Non-Carcinogen	Carcinogen	Non-Carcinogen
Distance	0.653	0.704	0.770
Reference	NTP/TR-364	NTP427	NTP/TR-213

Model Applicability

Unknown features are fingerprint features in the query molecule, but not found or appearing too infrequently in the training set.

1. All properties and OPS components are within expected ranges.

Feature Contribution

Top features for positive contribution				
Fingerprint	Bit/Smiles	Feature Structure	Score	Carcinogen in training set
SCFP_12	2088794301	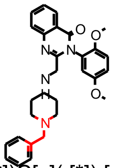 [*]N([*])C[c](:[*]):[*]	0.543	2 out of 2

SCFP_12	-205766035	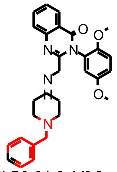 [*]N([*])C[c]1:[cH]:[cH]:[*]:[cH]:[cH]:1	0.543	2 out of 2
SCFP_12	1453622480	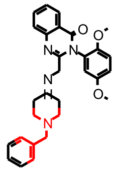 [*]CN(C[*])C[c](:[cH]:[*]):[cH]:[*]	0.377	1 out of 1
**Top Features for negative contribution**				
**Fingerprint**	**Bit/Smiles**	**Feature Structure**	**Score**	**Carcinogen in training set**
SCFP_12	1648492661	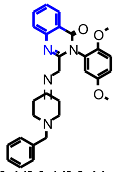 [*][c]1:[cH]:[cH]:[cH]:[cH]:[c]:1N=[*]	-0.91	0 out of 4
SCFP_12	9	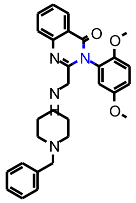 [*]N([*])[*]	-0.613	9 out of 47
SCFP_12	1257084377	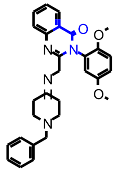 [*]N([*])C(=O)[c](:[*]):[*]	-0.479	1 out of 6

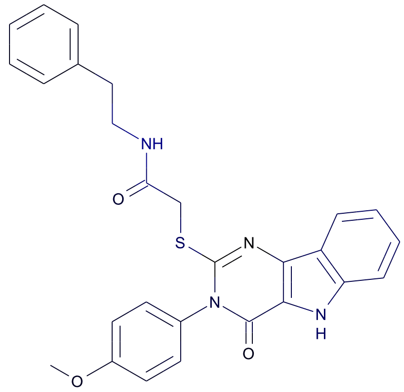

$C_{27}H_{24}N_4O_3S$
Molecular Weight: 484.56946
ALogP: 5.346
Rotatable Bonds: 8
Acceptors: 5
Donors: 2

Model Prediction

Prediction: Non-Carcinogen
Probability: 0.0148
Enrichment: 0.0377
Bayesian Score: -9.62
Mahalanobis Distance: 12.5
Mahalanobis Distance p-value: 1.88e-007

Prediction: Positive if the Bayesian score is above the estimated best cutoff value from minimizing the false positive and false negative rate.
Probability: The esimated probability that the sample is in the positive category. This assumes that the Bayesian score follows a normal distribution and is different from the prediction using a cutoff.
Enrichment: An estimate of enrichment, that is, the increased likelihood (versus random) of this sample being in the category.
Bayesian Score: The standard Laplacian-modified Bayesian score.
Mahalanobis Distance: The Mahalanobis distance (MD) is the distance to the center of the training data. The larger the MD, the less trustworthy the prediction.
Mahalanobis Distance p-value: The p-value gives the fraction of training data with an MD greater than or equal to the one for the given sample, assuming normally distributed data. The smaller the p-value, the less trustworthy the prediciton. For highly non-normal X properties (e.g., fingerprints), the MD p-value is wildly inaccurate.

Structural Similar Compounds

Name	Curcumin	RHODAMINE 6G	C.I. pigment red 23
Structure	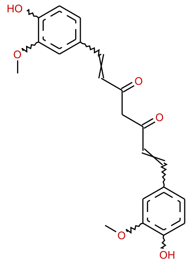	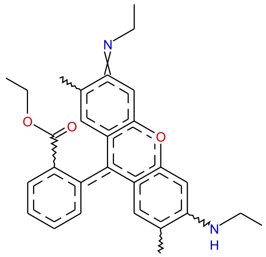	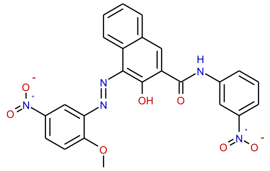
Actual Endpoint	Carcinogen	Non-Carcinogen	Non-Carcinogen
Predicted Endpoint	Carcinogen	Non-Carcinogen	Non-Carcinogen
Distance	0.703	0.735	0.826
Reference	NTP427	NTP/TR-364	NTP411

Model Applicability

Unknown features are fingerprint features in the query molecule, but not found or appearing too infrequently in the training set.

1. OPS PC11 out of range. Value: 6.1029. Training min, max, SD, explained variance: -3.6964, 3.2756, 1.387, 0.0309.

Feature Contribution

Top features for positive contribution

Fingerprint	Bit/Smiles	Feature Structure	Score	Carcinogen in training set
SCFP_12	-1211866396	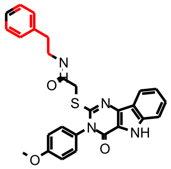 [*]CC[c]1:[cH]:[cH]:[*]:[cH]:[cH]:1	0.231	4 out of 8

SCFP_12	591469355	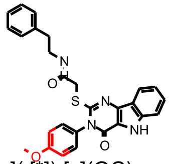 [*][c](:[*]):[c](OC):[cH]:[*]	0.142	7 out of 16
SCFP_12	622342378	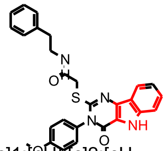 [*][c]1:[nH]:[c]2:[cH]:[cH]:[*]:[cH]:[c]:2:[c]:1[*]	0.138	1 out of 2
**Top Features for negative contribution**				
**Fingerprint**	**Bit/Smiles**	**Feature Structure**	**Score**	**Carcinogen in training set**
SCFP_12	17	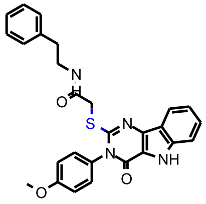 [*]S[*]	-1.24	1 out of 16
SCFP_12	-587569116	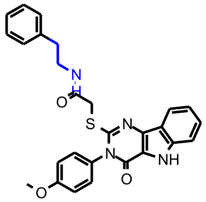 [*]NCC([*])[*]	-0.91	0 out of 4
SCFP_12	-111024397	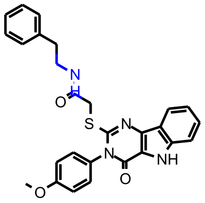 [*]CNC(=[*])[*]	-0.784	2 out of 15

Molecule

TOPKAT_Ocular_Irritancy_Mild_vs_Moderate_Severe

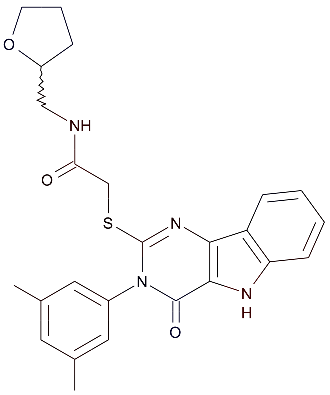

$C_{25}H_{26}N_4O_3S$

Molecular Weight: 462.56393

ALogP: 4.789

Rotatable Bonds: 6

Acceptors: 5

Donors: 2

Model Prediction

Prediction: Moderate_Severe

Probability: 0.833

Enrichment: 1.21

Bayesian Score: 0.777

Mahalanobis Distance: 11.4

Mahalanobis Distance p-value: 0.000864

Prediction: Positive if the Bayesian score is above the estimated best cutoff value from minimizing the false positive and false negative rate.
Probability: The esimated probability that the sample is in the positive category. This assumes that the Bayesian score follows a normal distribution and is different from the prediction using a cutoff.
Enrichment: An estimate of enrichment, that is, the increased likelihood (versus random) of this sample being in the category.
Bayesian Score: The standard Laplacian-modified Bayesian score.
Mahalanobis Distance: The Mahalanobis distance (MD) is the distance to the center of the training data. The larger the MD, the less trustworthy the prediction.
Mahalanobis Distance p-value: The p-value gives the fraction of training data with an MD greater than or equal to the one for the given sample, assuming normally distributed data. The smaller the p-value, the less trustworthy the prediciton. For highly non-normal X properties (e.g., fingerprints), the MD p-value is wildly inaccurate.

Structural Similar Compounds

Name	ANTHRAQUINONE; 1;1'-IMINODI-	ANTHRAQUINONE; 1;4-BIS(p-TOLYLAMINO)-	DINAPHTHO(1;2;3-CD:3';2';1'-IM)PERYLENE-5;10-DIONE;16;17-DIHYDROXY
Structure	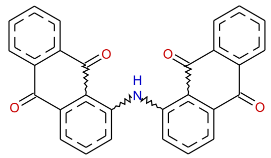	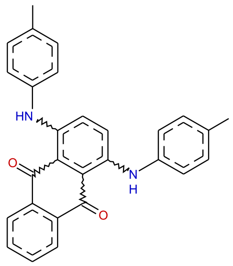	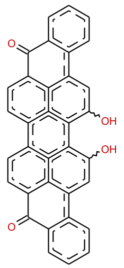
Actual Endpoint	Mild	Moderate_Severe	Mild
Predicted Endpoint	Mild	Mild	Mild
Distance	0.744	0.754	0.757
Reference	28ZPAK-;125;72	28ZPAK -;124;72	28ZPAK-;104;72

Model Applicability

Unknown features are fingerprint features in the query molecule, but not found or appearing too infrequently in the training set.

1. All properties and OPS components are within expected ranges.
2. Unknown FCFP_2 feature: 203707511: [*]C(=[*])[c]1:[nH]:[*]:[*]:[c]:1[*]

Feature Contribution

Top features for positive contribution				
**Fingerprint**	**Bit/Smiles**	**Feature Structure**	**Score**	**Moderate_Severe in training set**
FCFP_10	-547731249	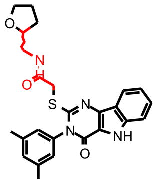 [*]CC(=O)NCC([*])[*]	0.294	3 out of 3

FCFP_10	-1272709286	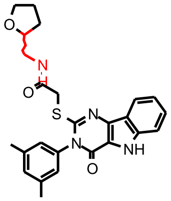 [*]NCC([*])[*]	0.285	234 out of 266
FCFP_10	155061250	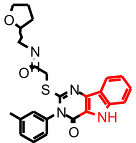 [*][c]1:[nH]:[c]2:[cH]:[cH]:[cH]:[cH]:[c]:2:[c]:1[*]	0.256	2 out of 2
**Top Features for negative contribution**				
**Fingerprint**	**Bit/Smiles**	**Feature Structure**	**Score**	**Moderate_Severe in training set**
FCFP_10	-1549163031	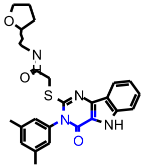 [*]N([*])C(=O)[c](:[*]):[*]	-0.657	5 out of 16
FCFP_10	418759064	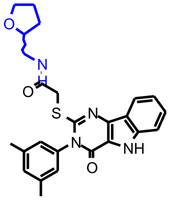 [*]NCC1CCCO1	-0.507	0 out of 1
FCFP_10	-1320007763	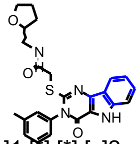 [*][c]1:[*]:[*]:[c]2:[*]:[cH]:[cH]:[cH]:[c]:1:2	-0.316	19 out of 40

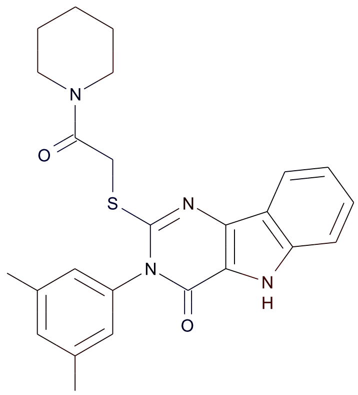

$C_{25}H_{26}N_4O_2S$
Molecular Weight: 446.56453
ALogP: 5.553
Rotatable Bonds: 4
Acceptors: 4
Donors: 1

Model Prediction

Prediction: Moderate_Severe
Probability: 0.824
Enrichment: 1.2
Bayesian Score: 0.124
Mahalanobis Distance: 12.3
Mahalanobis Distance p-value: 1.54e-005

Prediction: Positive if the Bayesian score is above the estimated best cutoff value from minimizing the false positive and false negative rate.
Probability: The esimated probability that the sample is in the positive category. This assumes that the Bayesian score follows a normal distribution and is different from the prediction using a cutoff.
Enrichment: An estimate of enrichment, that is, the increased likelihood (versus random) of this sample being in the category.
Bayesian Score: The standard Laplacian-modified Bayesian score.
Mahalanobis Distance: The Mahalanobis distance (MD) is the distance to the center of the training data. The larger the MD, the less trustworthy the prediction.
Mahalanobis Distance p-value: The p-value gives the fraction of training data with an MD greater than or equal to the one for the given sample, assuming normally distributed data. The smaller the p-value, the less trustworthy the prediciton. For highly non-normal X properties (e.g., fingerprints), the MD p-value is wildly inaccurate.

Structural Similar Compounds

Name	ANTHRAQUINONE; 1;1'-IMINODI-	2-(1'-ANTHRAQUINONYL)-AMINOBENZANTHRONE	1-BENZOYLAMINO-4-METHOXY-5-CHLORANTHRAQUINONE
Structure	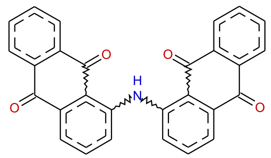	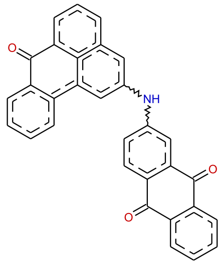	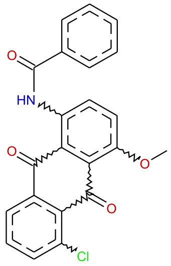
Actual Endpoint	Mild	Mild	Mild
Predicted Endpoint	Mild	Mild	Mild
Distance	0.588	0.598	0.642
Reference	28ZPAK-;125;72	28ZPAK-;126;72	28ZPAK-;90;72

Model Applicability

Unknown features are fingerprint features in the query molecule, but not found or appearing too infrequently in the training set.

1. All properties and OPS components are within expected ranges.
2. Unknown FCFP_2 feature: 203707511: [*]C(=[*])[c]1:[nH]:[*]:[*]:[c]:1[*]

Feature Contribution

Top features for positive contribution

Fingerprint	Bit/Smiles	Feature Structure	Score	Moderate_Severe in training set
FCFP_10	-1474971978	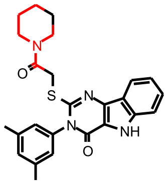 [*]C(=[*])N1C[*]CCC1	0.259	14 out of 16

FCFP_10	155061250	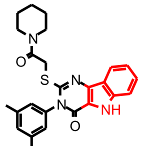 [*][c]1:[nH]:[c]2:[cH]:[cH]:[cH]:[cH]:[c]:2:[c]:1[*]	0.256	2 out of 2
FCFP_10	1673997923	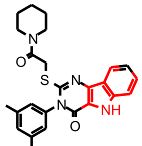 [*][c]1:[nH]:[c]2:[cH]:[cH]:[*]:[cH]:[c]:2:[c]:1[*]	0.256	2 out of 2

Top Features for negative contribution

Fingerprint	Bit/Smiles	Feature Structure	Score	Moderate_Severe in training set
FCFP_10	1908898748	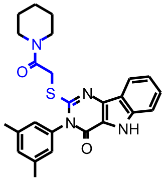 [*]N([*])C(=O)CSC(=[*])[*]	-1.09	0 out of 3
FCFP_10	-1549163031	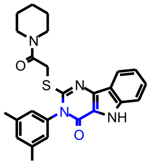 [*]N([*])C(=O)[c](:[*]):[*]	-0.657	5 out of 16
FCFP_10	-1320007763	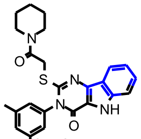 [*][c]1:[*]:[*]:[c]2:[*]:[cH]:[cH]:[cH]:[c]:1:2	-0.316	19 out of 40

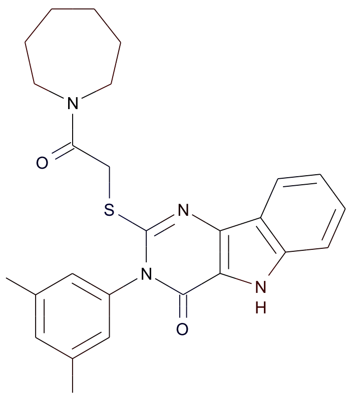

$C_{26}H_{28}N_4O_2S$
Molecular Weight: 460.59111
ALogP: 6.009
Rotatable Bonds: 4
Acceptors: 4
Donors: 1

Model Prediction

Prediction: Moderate_Severe
Probability: 0.831
Enrichment: 1.21
Bayesian Score: 0.623
Mahalanobis Distance: 21.7
Mahalanobis Distance p-value: 9e-045

Prediction: Positive if the Bayesian score is above the estimated best cutoff value from minimizing the false positive and false negative rate.
Probability: The esimated probability that the sample is in the positive category. This assumes that the Bayesian score follows a normal distribution and is different from the prediction using a cutoff.
Enrichment: An estimate of enrichment, that is, the increased likelihood (versus random) of this sample being in the category.
Bayesian Score: The standard Laplacian-modified Bayesian score.
Mahalanobis Distance: The Mahalanobis distance (MD) is the distance to the center of the training data. The larger the MD, the less trustworthy the prediction.
Mahalanobis Distance p-value: The p-value gives the fraction of training data with an MD greater than or equal to the one for the given sample, assuming normally distributed data. The smaller the p-value, the less trustworthy the prediciton. For highly non-normal X properties (e.g., fingerprints), the MD p-value is wildly inaccurate.

Structural Similar Compounds

Name	2-(1'-ANTHRAQUINONYL)-AMINOBENZANTHRONE	ANTHRAQUINONE; 1;1'-IMINODI-	1-BENZOYLAMINO-4-METHOXY-5-CHLORANTHRAQUINONE
Structure	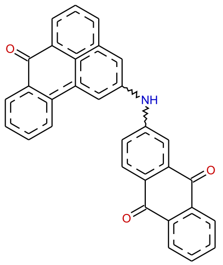	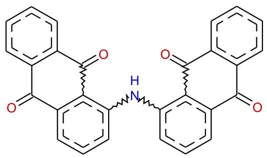	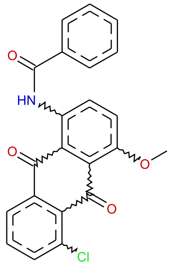
Actual Endpoint	Mild	Mild	Mild
Predicted Endpoint	Mild	Mild	Mild
Distance	0.567	0.614	0.694
Reference	28ZPAK-;126;72	28ZPAK-;125;72	28ZPAK-;90;72

Model Applicability

Unknown features are fingerprint features in the query molecule, but not found or appearing too infrequently in the training set.

1. All properties and OPS components are within expected ranges.
2. Unknown FCFP_2 feature: 203707511: [*]C(=[*])[c]1:[nH]:[*]:[*]:[c]:1[*]

Feature Contribution

Top features for positive contribution

Fingerprint	Bit/Smiles	Feature Structure	Score	Moderate_Severe in training set
FCFP_10	-1474971978	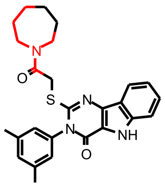 [*]C(=[*])N1C[*]CCC1	0.259	14 out of 16

FCFP_10	241966039	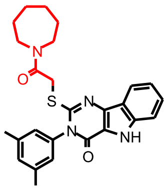 [*]CC(=O)N1CCCCCC1	0.256	2 out of 2
FCFP_10	155061250	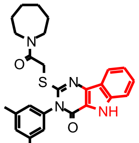 [*][c]1:[nH]:[c]2:[cH]:[cH]:[cH]:[cH]:[c]:2:[c]:1[*]	0.256	2 out of 2
**Top Features for negative contribution**				
**Fingerprint**	**Bit/Smiles**	**Feature Structure**	**Score**	**Moderate_Severe in training set**
FCFP_10	1908898748	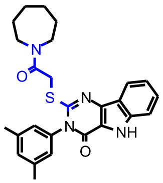 [*]N([*])C(=O)CSC(=[*])[*]	-1.09	0 out of 3
FCFP_10	-1549163031	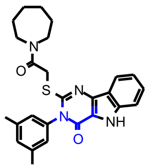 [*]N([*])C(=O)[c](:[*]):[*]	-0.657	5 out of 16
FCFP_10	-1320007763	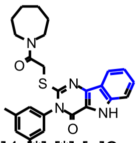 [*][c]1:[*]:[*]:[c]2:[*]:[cH]:[cH]:[cH]:[c]:1:2	-0.316	19 out of 40

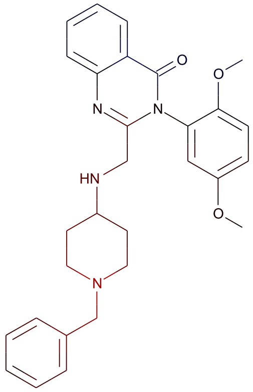

$C_{29}H_{32}N_4O_3$
Molecular Weight: 484.58938
ALogP: 3.743
Rotatable Bonds: 8
Acceptors: 6
Donors: 1

Model Prediction

Prediction: Moderate_Severe
Probability: 0.907
Enrichment: 1.32
Bayesian Score: 3.78
Mahalanobis Distance: 13.3
Mahalanobis Distance p-value: 4.01e-008

Prediction: Positive if the Bayesian score is above the estimated best cutoff value from minimizing the false positive and false negative rate.
Probability: The esimated probability that the sample is in the positive category. This assumes that the Bayesian score follows a normal distribution and is different from the prediction using a cutoff.
Enrichment: An estimate of enrichment, that is, the increased likelihood (versus random) of this sample being in the category.
Bayesian Score: The standard Laplacian-modified Bayesian score.
Mahalanobis Distance: The Mahalanobis distance (MD) is the distance to the center of the training data. The larger the MD, the less trustworthy the prediction.
Mahalanobis Distance p-value: The p-value gives the fraction of training data with an MD greater than or equal to the one for the given sample, assuming normally distributed data. The smaller the p-value, the less trustworthy the prediciton. For highly non-normal X properties (e.g., fingerprints), the MD p-value is wildly inaccurate.

Structural Similar Compounds

Name	COLCHICINE	Benzoic acid; p-(N-butyl-2-(butylamino)acetamido)-; butyl ester;	1-BENZOYLAMINO-4-METHOXY-5-CHLORANTHRAQUINONE
Structure	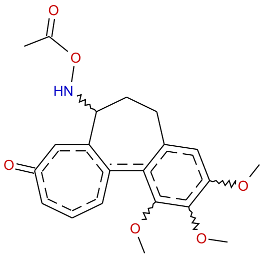	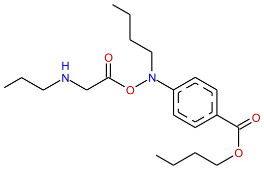	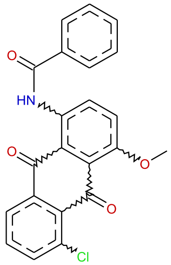
Actual Endpoint	Moderate_Severe	Moderate_Severe	Mild
Predicted Endpoint	Moderate_Severe	Moderate_Severe	Mild
Distance	0.727	0.751	0.753
Reference	AJOPAA 31;837;48	Arzneimittel-Forschung 8;609;58	28ZPAK-;90;72

Model Applicability

Unknown features are fingerprint features in the query molecule, but not found or appearing too infrequently in the training set.

1. All properties and OPS components are within expected ranges.

Feature Contribution

Top features for positive contribution				
**Fingerprint**	**Bit/Smiles**	**Feature Structure**	**Score**	**Moderate_Severe in training set**
FCFP_10	34686627	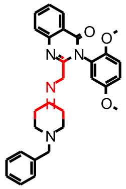 [*]CC(C[*])NCC(=[*])[*]	0.344	6 out of 6

FCFP_10	395218401	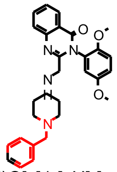 [*]N([*])C[c]1:[cH]:[cH]:[*]:[cH]:[cH]:1	0.344	6 out of 6
FCFP_10	906798516	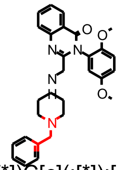 [*]N([*])C[c](:[*]):[*]	0.344	6 out of 6

Top Features for negative contribution

Fingerprint	Bit/Smiles	Feature Structure	Score	Moderate_Severe in training set
FCFP_10	-1977641857	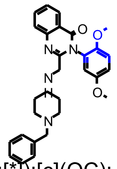 [*][c](:[*]):[c](OC):[cH]:[*]	-0.78	4 out of 15
FCFP_10	-1549163031	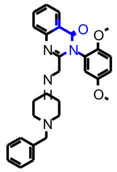 [*]N([*])C(=O)[c](:[*]):[*]	-0.657	5 out of 16
FCFP_10	136627117	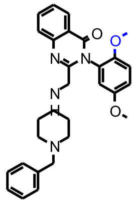 [*]OC	-0.316	46 out of 96

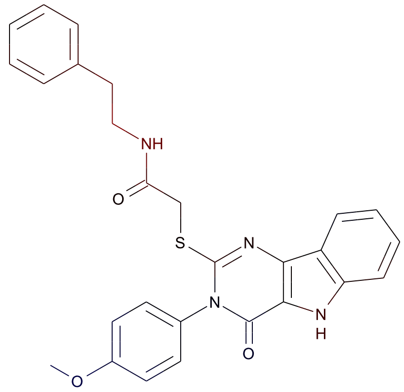

$C_{27}H_{24}N_4O_3S$
Molecular Weight: 484.56946
ALogP: 5.346
Rotatable Bonds: 8
Acceptors: 5
Donors: 2

Model Prediction

Prediction: Moderate_Severe
Probability: 0.833
Enrichment: 1.21
Bayesian Score: 0.763
Mahalanobis Distance: 9.89
Mahalanobis Distance p-value: 0.123

Prediction: Positive if the Bayesian score is above the estimated best cutoff value from minimizing the false positive and false negative rate.
Probability: The esimated probability that the sample is in the positive category. This assumes that the Bayesian score follows a normal distribution and is different from the prediction using a cutoff.
Enrichment: An estimate of enrichment, that is, the increased likelihood (versus random) of this sample being in the category.
Bayesian Score: The standard Laplacian-modified Bayesian score.
Mahalanobis Distance: The Mahalanobis distance (MD) is the distance to the center of the training data. The larger the MD, the less trustworthy the prediction.
Mahalanobis Distance p-value: The p-value gives the fraction of training data with an MD greater than or equal to the one for the given sample, assuming normally distributed data. The smaller the p-value, the less trustworthy the prediciton. For highly non-normal X properties (e.g., fingerprints), the MD p-value is wildly inaccurate.

Structural Similar Compounds

Name	ANTHRAQUINONE; 1;4-BIS(p-TOLYLAMINO)-	DINAPHTHO(1;2;3-CD:3';2';1'-IM)PERYLENE-5;10-DIONE;16;17-DIHYDROXY	ANTHRAQUINONE; 1;1'-IMINODI-
Structure	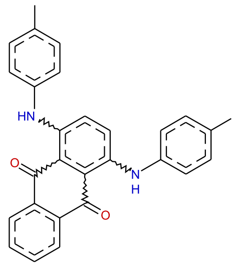	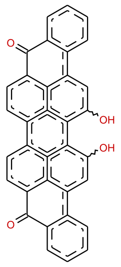	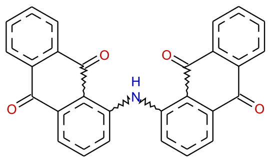
Actual Endpoint	Moderate_Severe	Mild	Mild
Predicted Endpoint	Mild	Mild	Mild
Distance	0.741	0.754	0.769
Reference	28ZPAK -;124;72	28ZPAK-;104;72	28ZPAK-;125;72

Model Applicability

Unknown features are fingerprint features in the query molecule, but not found or appearing too infrequently in the training set.

1. All properties and OPS components are within expected ranges.
2. Unknown FCFP_2 feature: 203707511: [*]C(=[*])[c]1:[nH]:[*]:[*]:[c]:1[*]

Feature Contribution

Top features for positive contribution

Fingerprint	Bit/Smiles	Feature Structure	Score	Moderate_Severe in training set
FCFP_10	-497728148	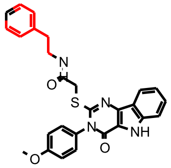 [*]CC[c]1:[cH]:[cH]:[*]:[cH]:[cH]:1	0.356	24 out of 25

FCFP_10	-547731249	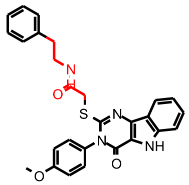 [*]CC(=O)NCC([*])[*]	0.294	3 out of 3
FCFP_10	-1272709286	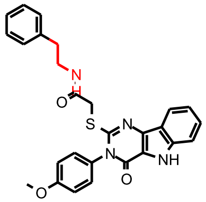 [*]NCC([*])[*]	0.285	234 out of 266
**Top Features for negative contribution**				
**Fingerprint**	**Bit/Smiles**	**Feature Structure**	**Score**	**Moderate_Severe in training set**
FCFP_10	-1977641857	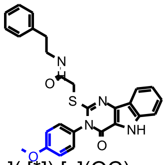 [*][c](:[*]):[c](OC):[cH]:[*]	-0.78	4 out of 15
FCFP_10	-1549163031	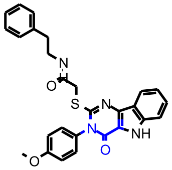 [*]N([*])C(=O)[c](:[*]):[*]	-0.657	5 out of 16
FCFP_10	-9847677	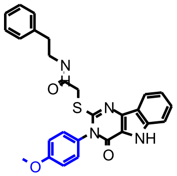 [*][c]1:[cH]:[cH]:[c](OC):[cH]:[cH]:1	-0.4	1 out of 3

Molecule

TOPKAT_Ocular_Irritancy_Moderate_vs_Severe

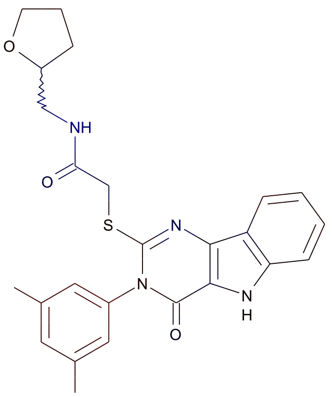

$C_{25}H_{26}N_4O_3S$
Molecular Weight: 462.56393
ALogP: 4.789
Rotatable Bonds: 6
Acceptors: 5
Donors: 2

Structural Similar Compounds

Name	ANTHRAQUINONE; 1;4-BIS(p-TOLYLAMINO)-	5-NORBORNENE-2;3-DICARBOXYLIC ACID; 1;4;5;6;7;7-HEXACHLORO-	Cinchoninamide; 2-butoxy-N-(2-(diethylamino)ethyl)-; monohydrochloride
Structure	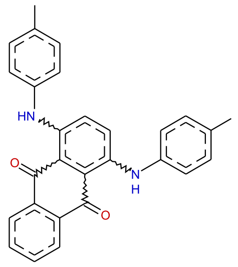	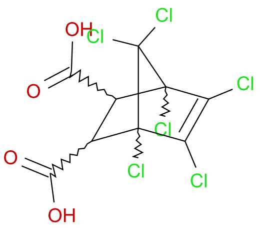	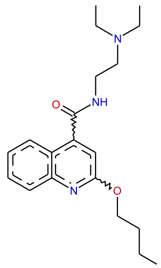
Actual Endpoint	Moderate	Severe	Severe
Predicted Endpoint	Moderate	Severe	Severe
Distance	0.768	0.794	0.819
Reference	28ZPAK -;124;72	28ZPAK-;92;72	Arzneimittel-Forschung 8;181;58

Model Prediction

Prediction: Moderate
Probability: 0.618
Enrichment: 0.996
Bayesian Score: -1.88
Mahalanobis Distance: 17.3
Mahalanobis Distance p-value: 9.11e-023

Prediction: Positive if the Bayesian score is above the estimated best cutoff value from minimizing the false positive and false negative rate.
Probability: The esimated probability that the sample is in the positive category. This assumes that the Bayesian score follows a normal distribution and is different from the prediction using a cutoff.
Enrichment: An estimate of enrichment, that is, the increased likelihood (versus random) of this sample being in the category.
Bayesian Score: The standard Laplacian-modified Bayesian score.
Mahalanobis Distance: The Mahalanobis distance (MD) is the distance to the center of the training data. The larger the MD, the less trustworthy the prediction.
Mahalanobis Distance p-value: The p-value gives the fraction of training data with an MD greater than or equal to the one for the given sample, assuming normally distributed data. The smaller the p-value, the less trustworthy the prediciton. For highly non-normal X properties (e.g., fingerprints), the MD p-value is wildly inaccurate.

Model Applicability

Unknown features are fingerprint features in the query molecule, but not found or appearing too infrequently in the training set.

1. OPS PC17 out of range. Value: -3.8047. Training min, max, SD, explained variance: -3.5374, 3.4597, 1.013, 0.0135.

Feature Contribution

Top features for positive contribution

Fingerprint	Bit/Smiles	Feature Structure	Score	Severe in training set
SCFP_12	-347281112	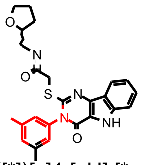 [*]N([*])[c]1:[cH]:[*]:[cH]:[c](C):[cH]:1	0.376	4 out of 4

SCFP_12	-978550879	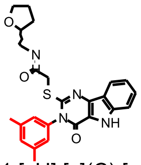 [*][c]1:[cH]:[c](C):[cH]:[c](C):[cH]:1	0.348	3 out of 3
SCFP_12	-1059974201	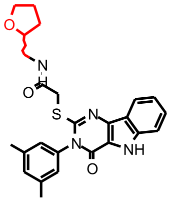 [*]CC1CCCO1	0.303	2 out of 2
**Top Features for negative contribution**				
**Fingerprint**	**Bit/Smiles**	**Feature Structure**	**Score**	**Severe in training set**
SCFP_12	1851000357	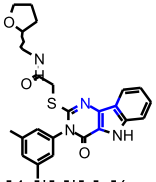 [*][c]1:[*]:[*]:[c](: [*]):[c]:1N=[*]	-1.04	0 out of 3
SCFP_12	2005026407	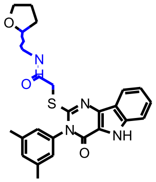 [*]CC(=O)NCC([*])[*]	-0.796	0 out of 2
SCFP_12	-587569116	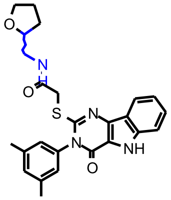 [*]NCC([*])[*]	-0.619	11 out of 35

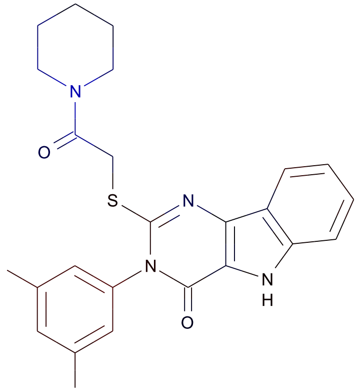

$C_{25}H_{26}N_4O_2S$
Molecular Weight: 446.56453
ALogP: 5.553
Rotatable Bonds: 4
Acceptors: 4
Donors: 1

Structural Similar Compounds

Name	o-Acetotoluidide; 6'-chloro-2-(p-chlorobenzyl(2-(pyrrolidinyl)ethyl)amino)-;	ANTHRAQUINONE;1-(2;4;6-TRIMETHYLPHENYLAMINO)-	2';6'-Acetoxylidide; 2-(p-chlorobenzyl(2-(pyrrolidinyl)ethyl)amino)-;
Structure	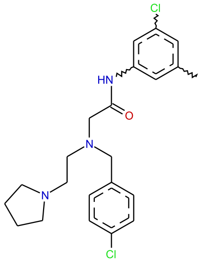	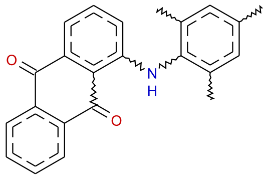	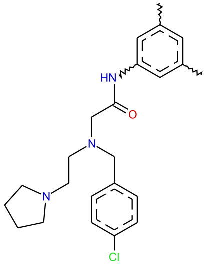
Actual Endpoint	Severe	Moderate	Severe
Predicted Endpoint	Severe	Moderate	Severe
Distance	0.701	0.703	0.706
Reference	Arzneimittel-Forschung 9;167;59	28ZPAK-;242;72	Arzneimittel-Forschung 9;167;59

Model Prediction

Prediction: Moderate
Probability: 0.555
Enrichment: 0.896
Bayesian Score: -3.13
Mahalanobis Distance: 10.9
Mahalanobis Distance p-value: 0.00199

Prediction: Positive if the Bayesian score is above the estimated best cutoff value from minimizing the false positive and false negative rate.
Probability: The esimated probability that the sample is in the positive category. This assumes that the Bayesian score follows a normal distribution and is different from the prediction using a cutoff.
Enrichment: An estimate of enrichment, that is, the increased likelihood (versus random) of this sample being in the category.
Bayesian Score: The standard Laplacian-modified Bayesian score.
Mahalanobis Distance: The Mahalanobis distance (MD) is the distance to the center of the training data. The larger the MD, the less trustworthy the prediction.
Mahalanobis Distance p-value: The p-value gives the fraction of training data with an MD greater than or equal to the one for the given sample, assuming normally distributed data. The smaller the p-value, the less trustworthy the prediciton. For highly non-normal X properties (e.g., fingerprints), the MD p-value is wildly inaccurate.

Model Applicability

Unknown features are fingerprint features in the query molecule, but not found or appearing too infrequently in the training set.

1. All properties and OPS components are within expected ranges.

Feature Contribution

Top features for positive contribution

Fingerprint	Bit/Smiles	Feature Structure	Score	Severe in training set
SCFP_12	-347281112	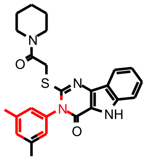 [*]N([*])[c]1:[cH]:[*]:[cH]:[c](C):[cH]:1	0.376	4 out of 4

SCFP_12	-978550879	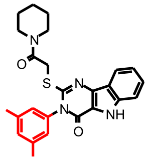<br>[*][c]1:[cH]:[c](C):[<br>cH]:[c](C):[cH]:1	0.348	3 out of 3
SCFP_12	528802270	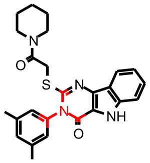<br>[*]C(=[*])N(C(=[*])[*<br>])[c](:[*]):[*]	0.303	2 out of 2

Top Features for negative contribution

Fingerprint	Bit/Smiles	Feature Structure	Score	Severe in training set
SCFP_12	1851000357	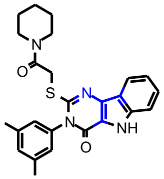<br>[*][c]1:[*]:[*]:[c](:<br>[*]):[c]:1N=[*]	-1.04	0 out of 3
SCFP_12	-2103400817	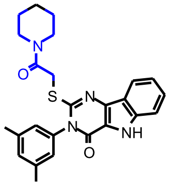<br>[*]CC(=O)N1CC[*]CC1	-0.941	2 out of 11
SCFP_12	-1343150366	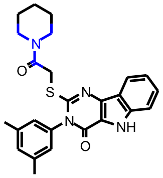<br>[*]CN(C[*])C(=[*])[*]	-0.667	7 out of 24

#UNDEFINED

TOPKAT_Ocular_Irritancy_Moderate_vs_Severe

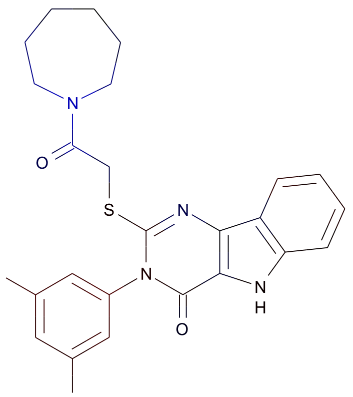

$C_{26}H_{28}N_4O_2S$
Molecular Weight: 460.59111
ALogP: 6.009
Rotatable Bonds: 4
Acceptors: 4
Donors: 1

Structural Similar Compounds

Name	ANTHRAQUINONE;1-(2;4;6-TRIMETHYLPHENYLAMINO)-	o-Acetotoluidide; 6'-chloro-2-(p-chlorobenzyl(2-(pyrrolidinyl)ethyl)amino)-;	2';6'-Acetoxylidide; 2-(p-chlorobenzyl(2-(pyrrolidinyl)ethyl)amino)-;
Structure	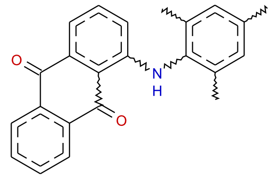	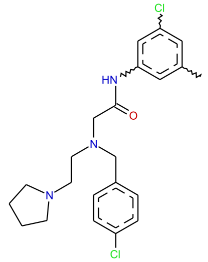	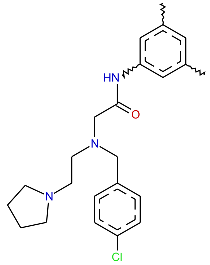
Actual Endpoint	Moderate	Severe	Severe
Predicted Endpoint	Moderate	Severe	Severe
Distance	0.717	0.717	0.728
Reference	28ZPAK-;242;72	Arzneimittel-Forschung 9;167;59	Arzneimittel-Forschung 9;167;59

Model Prediction

Prediction: Moderate
Probability: 0.546
Enrichment: 0.881
Bayesian Score: -3.3
Mahalanobis Distance: 11.1
Mahalanobis Distance p-value: 0.0012

Prediction: Positive if the Bayesian score is above the estimated best cutoff value from minimizing the false positive and false negative rate.
Probability: The esimated probability that the sample is in the positive category. This assumes that the Bayesian score follows a normal distribution and is different from the prediction using a cutoff.
Enrichment: An estimate of enrichment, that is, the increased likelihood (versus random) of this sample being in the category.
Bayesian Score: The standard Laplacian-modified Bayesian score.
Mahalanobis Distance: The Mahalanobis distance (MD) is the distance to the center of the training data. The larger the MD, the less trustworthy the prediction.
Mahalanobis Distance p-value: The p-value gives the fraction of training data with an MD greater than or equal to the one for the given sample, assuming normally distributed data. The smaller the p-value, the less trustworthy the prediciton. For highly non-normal X properties (e.g., fingerprints), the MD p-value is wildly inaccurate.

Model Applicability

Unknown features are fingerprint features in the query molecule, but not found or appearing too infrequently in the training set.

1. All properties and OPS components are within expected ranges.

Feature Contribution

Top features for positive contribution				
**Fingerprint**	**Bit/Smiles**	**Feature Structure**	**Score**	**Severe in training set**
SCFP_12	-347281112	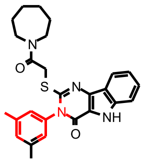 [*]N([*])[c]1:[cH]:[*]:[cH]:[c](C):[cH]:1	0.376	4 out of 4

SCFP_12	-978550879	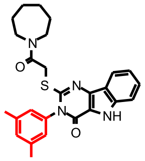 [*][c]1:[cH]:[c](C):[cH]:[c](C):[cH]:1	0.348	3 out of 3
SCFP_12	528802270	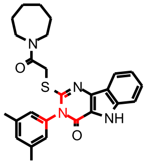 [*]C(=[*])N(C(=[*])[*])[c](:[*]):[*]	0.303	2 out of 2
**Top Features for negative contribution**				
**Fingerprint**	**Bit/Smiles**	**Feature Structure**	**Score**	**Severe in training set**
SCFP_12	1851000357	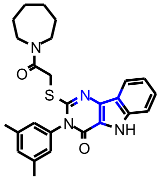 [*][c]1:[*]:[*]:[c](: [*]):[c]:1N=[*]	-1.04	0 out of 3
SCFP_12	-2103400817	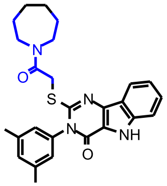 [*]CC(=O)N1CC[*]CC1	-0.941	2 out of 11
SCFP_12	-1343150366	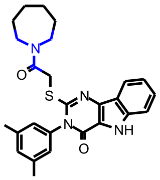 [*]CN(C[*])C(=[*])[*]	-0.667	7 out of 24

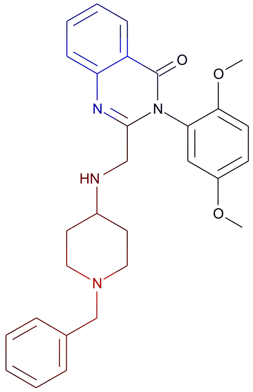

$C_{29}H_{32}N_4O_3$
Molecular Weight: 484.58938
ALogP: 3.743
Rotatable Bonds: 8
Acceptors: 6
Donors: 1

Structural Similar Compounds

Name	COLCHICINE	Benzoic acid; p-(N-butyl-2-(butylamino)acetamido)-; butyl ester;	Cinchoninamide; 2-butoxy-N-(2-(diethylamino)ethyl)-; monohydrochloride
Structure	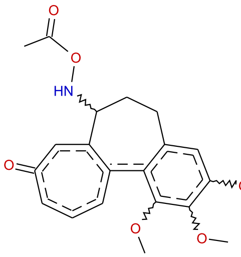	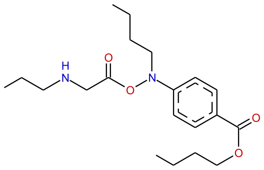	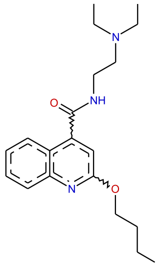
Actual Endpoint	Severe	Severe	Severe
Predicted Endpoint	Severe	Severe	Severe
Distance	0.736	0.756	0.766
Reference	AJOPAA 31;837;48	Arzneimittel-Forschung 8;609;58	Arzneimittel-Forschung 8;181;58

Model Prediction

Prediction: Severe
Probability: 0.717
Enrichment: 1.16
Bayesian Score: 0.826
Mahalanobis Distance: 11
Mahalanobis Distance p-value: 0.00147

Prediction: Positive if the Bayesian score is above the estimated best cutoff value from minimizing the false positive and false negative rate.
Probability: The esimated probability that the sample is in the positive category. This assumes that the Bayesian score follows a normal distribution and is different from the prediction using a cutoff.
Enrichment: An estimate of enrichment, that is, the increased likelihood (versus random) of this sample being in the category.
Bayesian Score: The standard Laplacian-modified Bayesian score.
Mahalanobis Distance: The Mahalanobis distance (MD) is the distance to the center of the training data. The larger the MD, the less trustworthy the prediction.
Mahalanobis Distance p-value: The p-value gives the fraction of training data with an MD greater than or equal to the one for the given sample, assuming normally distributed data. The smaller the p-value, the less trustworthy the prediciton. For highly non-normal X properties (e.g., fingerprints), the MD p-value is wildly inaccurate.

Model Applicability

Unknown features are fingerprint features in the query molecule, but not found or appearing too infrequently in the training set.

1. All properties and OPS components are within expected ranges.

Feature Contribution

Top features for positive contribution				
**Fingerprint**	**Bit/Smiles**	**Feature Structure**	**Score**	**Severe in training set**
SCFP_12	-1430588017	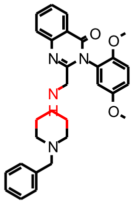 [*]CC(C[*])N[*]	0.469	21 out of 21

SCFP_12	2088734719	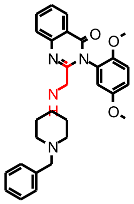 [*]NCC(=[*])[*]	0.441	10 out of 10
SCFP_12	2088794301	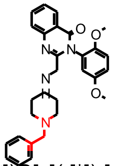 [*]N([*])C[c](:[*]):[*]	0.42	7 out of 7
**Top Features for negative contribution**				
**Fingerprint**	**Bit/Smiles**	**Feature Structure**	**Score**	**Severe in training set**
SCFP_12	-1377141613	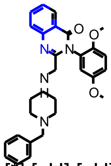 [*][c]1:[*]:[cH]:[cH]:[cH]:[c]:1N=[*]	-1.04	0 out of 3
SCFP_12	1851000357	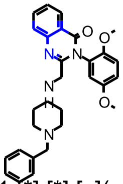 [*][c]1:[*]:[*]:[c](:[*]):[c]:1N=[*]	-1.04	0 out of 3
SCFP_12	1648492661	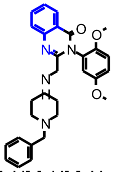 [*][c]1:[cH]:[cH]:[cH]:[cH]:[c]:1N=[*]	-0.796	0 out of 2

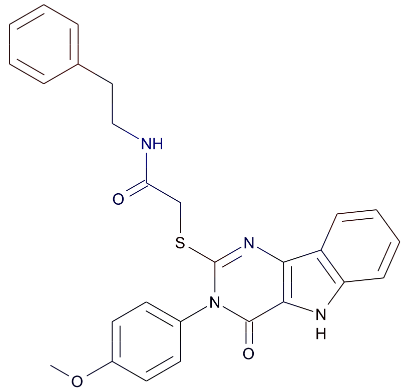

$C_{27}H_{24}N_4O_3S$
Molecular Weight: 484.56946
ALogP: 5.346
Rotatable Bonds: 8
Acceptors: 5
Donors: 2

Structural Similar Compounds

Name	ANTHRAQUINONE; 1;4-BIS(p-TOLYLAMINO)-	Cinchoninamide; 2-butoxy-N-(2-(diethylamino)ethyl)-; monohydrochloride	o-Acetotoluidide; 6'-chloro-2-(p-chlorobenzyl(2-(pyrrolidinyl)ethyl)amino)-;
Structure	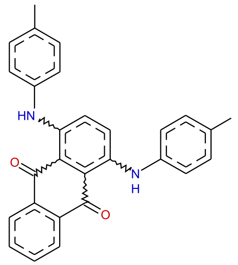	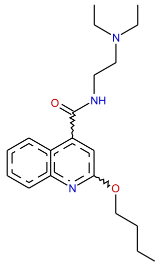	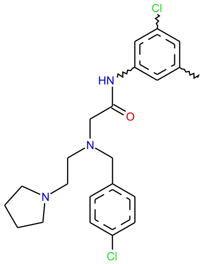
Actual Endpoint	Moderate	Severe	Severe
Predicted Endpoint	Moderate	Severe	Severe
Distance	0.752	0.851	0.859
Reference	28ZPAK -;124;72	Arzneimittel-Forschung 8;181;58	Arzneimittel-Forschung 9;167;59

Model Prediction

Prediction: Moderate
Probability: 0.544
Enrichment: 0.877
Bayesian Score: -3.34
Mahalanobis Distance: 11.9
Mahalanobis Distance p-value: 3.54e-005

Prediction: Positive if the Bayesian score is above the estimated best cutoff value from minimizing the false positive and false negative rate.
Probability: The esimated probability that the sample is in the positive category. This assumes that the Bayesian score follows a normal distribution and is different from the prediction using a cutoff.
Enrichment: An estimate of enrichment, that is, the increased likelihood (versus random) of this sample being in the category.
Bayesian Score: The standard Laplacian-modified Bayesian score.
Mahalanobis Distance: The Mahalanobis distance (MD) is the distance to the center of the training data. The larger the MD, the less trustworthy the prediction.
Mahalanobis Distance p-value: The p-value gives the fraction of training data with an MD greater than or equal to the one for the given sample, assuming normally distributed data. The smaller the p-value, the less trustworthy the prediciton. For highly non-normal X properties (e.g., fingerprints), the MD p-value is wildly inaccurate.

Model Applicability

Unknown features are fingerprint features in the query molecule, but not found or appearing too infrequently in the training set.

1. OPS PC11 out of range. Value: -4.0661. Training min, max, SD, explained variance: -3.5735, 4.44, 1.244, 0.0204.
2. OPS PC17 out of range. Value: -3.7693. Training min, max, SD, explained variance: -3.5374, 3.4597, 1.013, 0.0135.

Feature Contribution

Top features for positive contribution

Fingerprint	Bit/Smiles	Feature Structure	Score	Severe in training set

SCFP_12	-1640858361	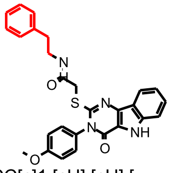 [*]CC[c]1:[cH]:[cH]:[cH]:[cH]:[cH]:1	0.376	4 out of 4
SCFP_12	528802270	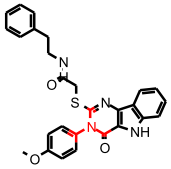 [*]C(=[*])N(C(=[*])[*])[c](:[*]):[*]	0.303	2 out of 2
SCFP_12	1508830534	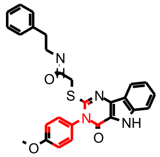 [*]C(=[*])N(C(=[*])[*])[c]1:[cH]:[c]([*]) :[*]:[c]([*]):[cH]:1	0.303	2 out of 2
**Top Features for negative contribution**				
**Fingerprint**	**Bit/Smiles**	**Feature Structure**	**Score**	**Severe in training set**
SCFP_12	1851000357	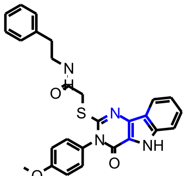 [*][c]1:[*]:[*]:[c](: [*]):[c]:1N=[*]	-1.04	0 out of 3
SCFP_12	2005026407	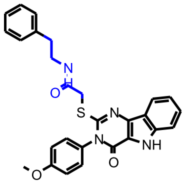 [*]CC(=O)NCC([*])[*]	-0.796	0 out of 2

SCFP_12	-587569116	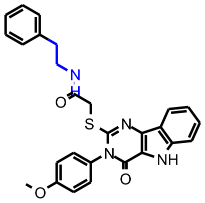 [*]NCC([*])[*]	-0.619	11 out of 35

Molecule

TOPKAT_Ocular_Irritancy_None_vs_Irritant

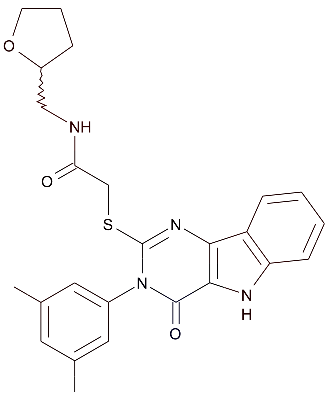

$C_{25}H_{26}N_4O_3S$
Molecular Weight: 462.56393
ALogP: 4.789
Rotatable Bonds: 6
Acceptors: 5
Donors: 2

Structural Similar Compounds

Name	BENZANILIDE;2';2'''-DITHIOBIS-	DINAPHTHO(1;2;3-CD:3';2';1'-IM)PERYLENE-5;10-DIONE;16;17-DIHYDROXY	ANTHRAQUINONE; 1;4-BIS(p-TOLYLAMINO)-
Structure	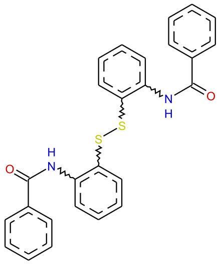	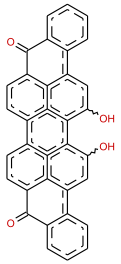	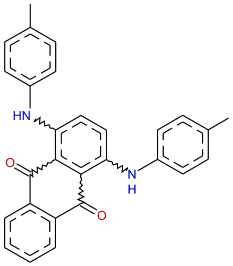
Actual Endpoint	Non-Irritant	Irritant	Irritant
Predicted Endpoint	Non-Irritant	Irritant	Non-Irritant
Distance	0.622	0.733	0.735
Reference	28ZPAK-;173;72	28ZPAK-;104;72	28ZPAK -;124;72

Model Prediction

Prediction: Irritant
Probability: 1
Enrichment: 1.18
Bayesian Score: 1.65
Mahalanobis Distance: 13.4
Mahalanobis Distance p-value: 1.42e-008

Prediction: Positive if the Bayesian score is above the estimated best cutoff value from minimizing the false positive and false negative rate.
Probability: The esimated probability that the sample is in the positive category. This assumes that the Bayesian score follows a normal distribution and is different from the prediction using a cutoff.
Enrichment: An estimate of enrichment, that is, the increased likelihood (versus random) of this sample being in the category.
Bayesian Score: The standard Laplacian-modified Bayesian score.
Mahalanobis Distance: The Mahalanobis distance (MD) is the distance to the center of the training data. The larger the MD, the less trustworthy the prediction.
Mahalanobis Distance p-value: The p-value gives the fraction of training data with an MD greater than or equal to the one for the given sample, assuming normally distributed data. The smaller the p-value, the less trustworthy the prediciton. For highly non-normal X properties (e.g., fingerprints), the MD p-value is wildly inaccurate.

Model Applicability

Unknown features are fingerprint features in the query molecule, but not found or appearing too infrequently in the training set.

1. All properties and OPS components are within expected ranges.
2. Unknown FCFP_2 feature: 203707511: [*]C(=[*])[c]1:[nH]:[*]:[*]:[c]:1[*]

Feature Contribution

Top features for positive contribution				
**Fingerprint**	**Bit/Smiles**	**Feature Structure**	**Score**	**Irritant in training set**
FCFP_12	-885550502	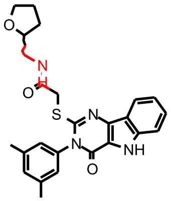 [*]CNC(=[*])[*]	0.18	64 out of 66

FCFP_12	-675291618	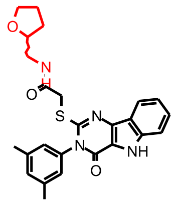 [*]NCC1CCCO1	0.175	5 out of 5
FCFP_12	2005402822	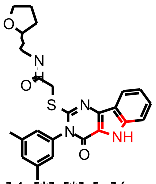 [*][c]1:[*]:[*]:[c](:[*]):[nH]:1	0.175	5 out of 5

Top Features for negative contribution

Fingerprint	Bit/Smiles	Feature Structure	Score	Irritant in training set
FCFP_12	-1549163031	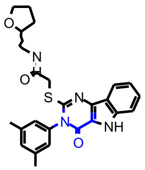 [*]N([*])C(=O)[c](:[*]):[*]	-0.623	16 out of 38
FCFP_12	580453787	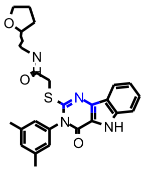 [*]C(=N[c](:[*]):[*])[*]	-0.132	2 out of 3
FCFP_12	-1475780652	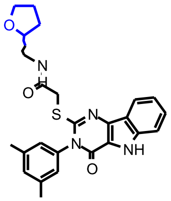 [*]C1CCCO1	-0.0837	121 out of 163

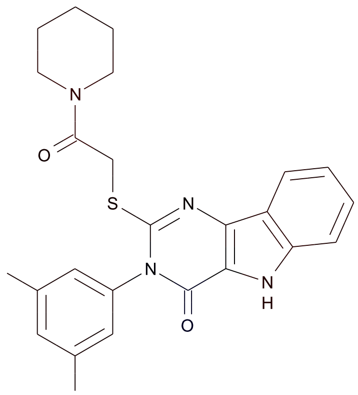

$C_{25}H_{26}N_4O_2S$
Molecular Weight: 446.56453
ALogP: 5.553
Rotatable Bonds: 4
Acceptors: 4
Donors: 1

Structural Similar Compounds

Name	ANTHRAQUINONE; 1;1'-IMINODI-	2-(1'-ANTHRAQUINONYL)-AMINOBENZANTHRONE	ANTHRA(2;1;9-mna)NAPHTH(2;3-h)ACRIDINE-5;10;15-TRIONE
Structure	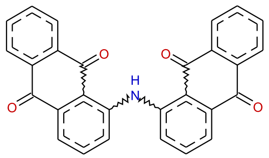	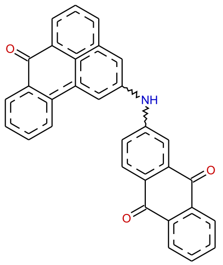	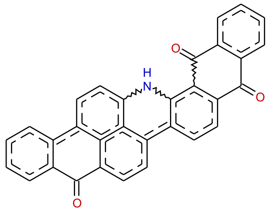
Actual Endpoint	Irritant	Irritant	Non-Irritant
Predicted Endpoint	Irritant	Irritant	Non-Irritant
Distance	0.584	0.592	0.609
Reference	28ZPAK-;125;72	28ZPAK-;126;72	28ZPAK -;248;72

Model Prediction

Prediction: Irritant
Probability: 1
Enrichment: 1.18
Bayesian Score: 1.1
Mahalanobis Distance: 12.9
Mahalanobis Distance p-value: 3.64e-007

Prediction: Positive if the Bayesian score is above the estimated best cutoff value from minimizing the false positive and false negative rate.
Probability: The esimated probability that the sample is in the positive category. This assumes that the Bayesian score follows a normal distribution and is different from the prediction using a cutoff.
Enrichment: An estimate of enrichment, that is, the increased likelihood (versus random) of this sample being in the category.
Bayesian Score: The standard Laplacian-modified Bayesian score.
Mahalanobis Distance: The Mahalanobis distance (MD) is the distance to the center of the training data. The larger the MD, the less trustworthy the prediction.
Mahalanobis Distance p-value: The p-value gives the fraction of training data with an MD greater than or equal to the one for the given sample, assuming normally distributed data. The smaller the p-value, the less trustworthy the prediciton. For highly non-normal X properties (e.g., fingerprints), the MD p-value is wildly inaccurate.

Model Applicability

Unknown features are fingerprint features in the query molecule, but not found or appearing too infrequently in the training set.

1. All properties and OPS components are within expected ranges.
2. Unknown FCFP_2 feature: 203707511: [*]C(=[*])[c]1:[nH]:[*]:[*]:[c]:1[*]

Feature Contribution

Top features for positive contribution

Fingerprint	Bit/Smiles	Feature Structure	Score	Irritant in training set
FCFP_12	-446103674	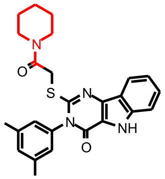 [*]C(=[*])N1CCCCC1	0.18	6 out of 6

FCFP_12	2005402822	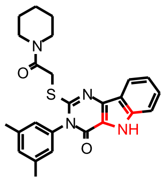 [*][c]1:[*]:[*]:[c](:[*]):[nH]:1	0.175	5 out of 5
FCFP_12	-264534230	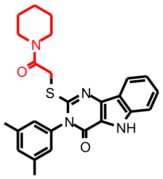 [*]CC(=O)N1CCCCC1	0.167	4 out of 4
**Top Features for negative contribution**				
**Fingerprint**	**Bit/Smiles**	**Feature Structure**	**Score**	**Irritant in training set**
FCFP_12	-1549163031	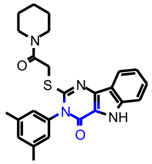 [*]N([*])C(=O)[c](:[*]):[*]	-0.623	16 out of 38
FCFP_12	1175638033	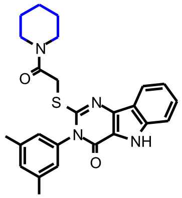 [*]1CCCCC1	-0.133	207 out of 293
FCFP_12	580453787	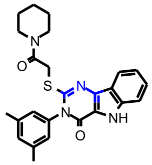 [*]C(=N[c](:[*]):[*])[*]	-0.132	2 out of 3

#UNDEFINED

TOPKAT_Ocular_Irritancy_None_vs_Irritant

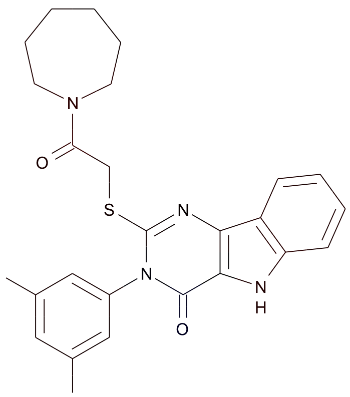

$C_{26}H_{28}N_4O_2S$
Molecular Weight: 460.59111
ALogP: 6.009
Rotatable Bonds: 4
Acceptors: 4
Donors: 1

Structural Similar Compounds

Name	2-(1'-ANTHRAQUINONYL)-AMINOBENZANTHRONE	ANTHRA(2;1;9-mna)NAPHTH(2;3-h)ACRIDINE-5;10;15-TRIONE	ANTHRAQUINONE; 1;1'-IMINODI-
Structure	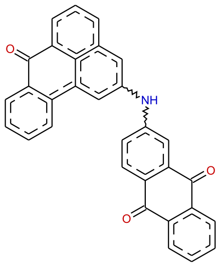	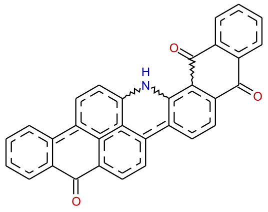	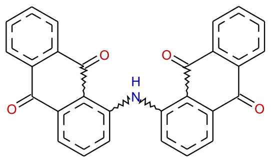
Actual Endpoint	Irritant	Non-Irritant	Irritant
Predicted Endpoint	Irritant	Non-Irritant	Irritant
Distance	0.565	0.597	0.607
Reference	28ZPAK-;126;72	28ZPAK -;248;72	28ZPAK-;125;72

Model Prediction

Prediction: Irritant
Probability: 1
Enrichment: 1.18
Bayesian Score: 0.994
Mahalanobis Distance: 21.4
Mahalanobis Distance p-value: 2.29e-044

Prediction: Positive if the Bayesian score is above the estimated best cutoff value from minimizing the false positive and false negative rate.
Probability: The esimated probability that the sample is in the positive category. This assumes that the Bayesian score follows a normal distribution and is different from the prediction using a cutoff.
Enrichment: An estimate of enrichment, that is, the increased likelihood (versus random) of this sample being in the category.
Bayesian Score: The standard Laplacian-modified Bayesian score.
Mahalanobis Distance: The Mahalanobis distance (MD) is the distance to the center of the training data. The larger the MD, the less trustworthy the prediction.
Mahalanobis Distance p-value: The p-value gives the fraction of training data with an MD greater than or equal to the one for the given sample, assuming normally distributed data. The smaller the p-value, the less trustworthy the prediciton. For highly non-normal X properties (e.g., fingerprints), the MD p-value is wildly inaccurate.

Model Applicability

Unknown features are fingerprint features in the query molecule, but not found or appearing too infrequently in the training set.

1. All properties and OPS components are within expected ranges.
2. Unknown FCFP_2 feature: 203707511: [*]C(=[*])[c]1:[nH]:[*]:[*]:[c]:1[*]

Feature Contribution

Top features for positive contribution

Fingerprint	Bit/Smiles	Feature Structure	Score	Irritant in training set
FCFP_12	-446103674	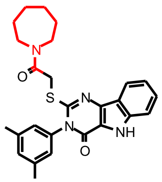 [*]C(=[*])N1CCCCCC1	0.18	6 out of 6

FCFP_12	2005402822	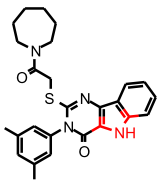 [*][c]1:[*]:[*]:[c](:[*]):[nH]:1	0.175	5 out of 5
FCFP_12	-264534230	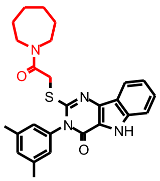 [*]CC(=O)N1CCCCCC1	0.167	4 out of 4

Top Features for negative contribution

Fingerprint	Bit/Smiles	Feature Structure	Score	Irritant in training set
FCFP_12	-1549163031	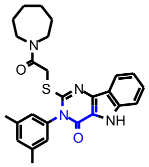 [*]N([*])C(=O)[c](:[*]):[*]	-0.623	16 out of 38
FCFP_12	1175638033	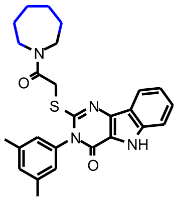 [*]1CCCCCC1	-0.133	207 out of 293
FCFP_12	580453787	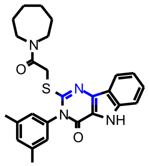 [*]C(=N[c](:[*]):[*])[*]	-0.132	2 out of 3

#UNDEFINED

TOPKAT_Ocular_Irritancy_None_vs_Irritant

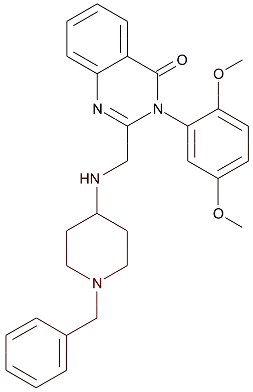

$C_{29}H_{32}N_4O_3$
Molecular Weight: 484.58938
ALogP: 3.743
Rotatable Bonds: 8
Acceptors: 6
Donors: 1

Structural Similar Compounds

Name	COLCHICINE	Benzoic acid; p-(N-butyl-2-(butylamino)acetamido)-; butyl ester;	ANTHRAQUINONE; 1;1'-IMINODI-
Structure	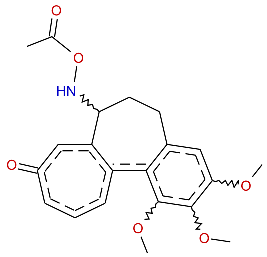	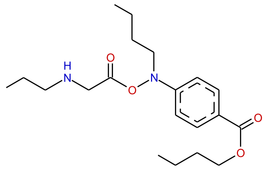	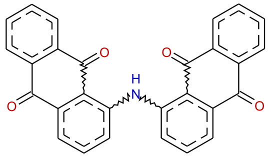
Actual Endpoint	Irritant	Irritant	Irritant
Predicted Endpoint	Irritant	Non-Irritant	Irritant
Distance	0.713	0.733	0.734
Reference	AJOPAA 31;837;48	Arzneimittel-Forschung 8;609;58	28ZPAK-;125;72

Model Prediction

Prediction: Irritant
Probability: 1
Enrichment: 1.18
Bayesian Score: 2.26
Mahalanobis Distance: 10.9
Mahalanobis Distance p-value: 0.00727

Prediction: Positive if the Bayesian score is above the estimated best cutoff value from minimizing the false positive and false negative rate.
Probability: The esimated probability that the sample is in the positive category. This assumes that the Bayesian score follows a normal distribution and is different from the prediction using a cutoff.
Enrichment: An estimate of enrichment, that is, the increased likelihood (versus random) of this sample being in the category.
Bayesian Score: The standard Laplacian-modified Bayesian score.
Mahalanobis Distance: The Mahalanobis distance (MD) is the distance to the center of the training data. The larger the MD, the less trustworthy the prediction.
Mahalanobis Distance p-value: The p-value gives the fraction of training data with an MD greater than or equal to the one for the given sample, assuming normally distributed data. The smaller the p-value, the less trustworthy the prediciton. For highly non-normal X properties (e.g., fingerprints), the MD p-value is wildly inaccurate.

Model Applicability

Unknown features are fingerprint features in the query molecule, but not found or appearing too infrequently in the training set.

1. All properties and OPS components are within expected ranges.

Feature Contribution

Top features for positive contribution

Fingerprint	Bit/Smiles	Feature Structure	Score	Irritant in training set
FCFP_12	906798516	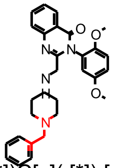 [*]N([*])C[c](:[*]):[*]	0.18	6 out of 6

FCFP_12	34686627	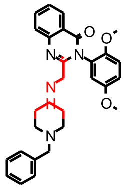 [*]CC(C[*])NCC(=[*])[*]	0.18	6 out of 6
FCFP_12	395218401	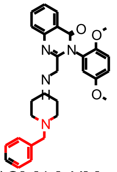 [*]N([*])C[c]1:[cH]:[cH]:[*]:[cH]:[cH]:1	0.18	6 out of 6
**Top Features for negative contribution**				
**Fingerprint**	**Bit/Smiles**	**Feature Structure**	**Score**	**Irritant in training set**
FCFP_12	-1549163031	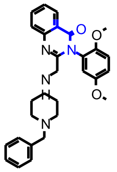 [*]N([*])C(=O)[c](:[*]):[*]	-0.623	16 out of 38
FCFP_12	580453787	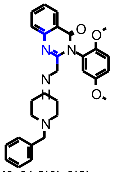 [*]C(=N[c](:[*]):[*]) [*]	-0.132	2 out of 3
FCFP_12	-1698724694	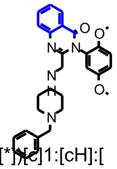 [*]C(=[*])[c]1:[cH]:[cH]:[cH]:[cH]:[c]:1[*]	-0.0964	107 out of 146

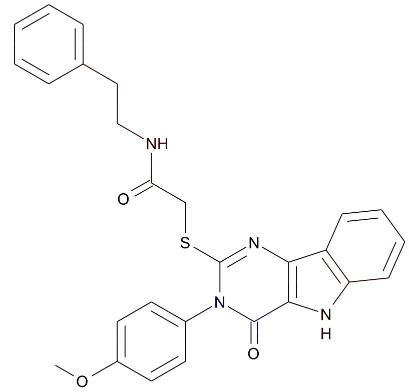

$C_{27}H_{24}N_4O_3S$
Molecular Weight: 484.56946
ALogP: 5.346
Rotatable Bonds: 8
Acceptors: 5
Donors: 2

Model Prediction

Prediction: Irritant
Probability: 0.976
Enrichment: 1.15
Bayesian Score: 0.279
Mahalanobis Distance: 9.04
Mahalanobis Distance p-value: 0.491

Prediction: Positive if the Bayesian score is above the estimated best cutoff value from minimizing the false positive and false negative rate.
Probability: The esimated probability that the sample is in the positive category. This assumes that the Bayesian score follows a normal distribution and is different from the prediction using a cutoff.
Enrichment: An estimate of enrichment, that is, the increased likelihood (versus random) of this sample being in the category.
Bayesian Score: The standard Laplacian-modified Bayesian score.
Mahalanobis Distance: The Mahalanobis distance (MD) is the distance to the center of the training data. The larger the MD, the less trustworthy the prediction.
Mahalanobis Distance p-value: The p-value gives the fraction of training data with an MD greater than or equal to the one for the given sample, assuming normally distributed data. The smaller the p-value, the less trustworthy the prediciton. For highly non-normal X properties (e.g., fingerprints), the MD p-value is wildly inaccurate.

Structural Similar Compounds

Name	BENZANILIDE;2';2'''-DITHIOBIS-	ANTHRAQUINONE; 1;4-BIS(p-TOLYLAMINO)-	DINAPHTHO(1;2;3-CD:3';2';1'-IM)PERYLENE-5;10-DIONE;16;17-DIHYDROXY
Structure	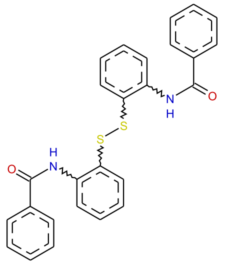	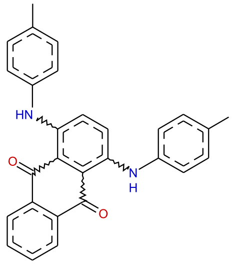	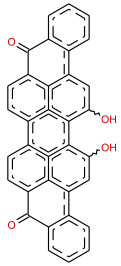
Actual Endpoint	Non-Irritant	Irritant	Irritant
Predicted Endpoint	Non-Irritant	Non-Irritant	Irritant
Distance	0.585	0.726	0.732
Reference	28ZPAK-;173;72	28ZPAK -;124;72	28ZPAK-;104;72

Model Applicability

Unknown features are fingerprint features in the query molecule, but not found or appearing too infrequently in the training set.

1. All properties and OPS components are within expected ranges.
2. Unknown FCFP_2 feature: 203707511: [*]C(=[*])[c]1:[nH]:[*]:[*]:[c]:1[*]

Feature Contribution

Top features for positive contribution				
**Fingerprint**	**Bit/Smiles**	**Feature Structure**	**Score**	**Irritant in training set**
FCFP_12	-885550502	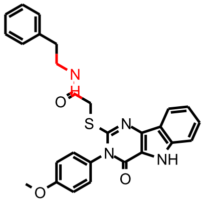 [*]CNC(=[*])[*]	0.18	64 out of 66

FCFP_12	2005402822	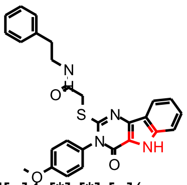 [*][c]1:[*]:[*]:[c](:[*]):[nH]:1	0.175	5 out of 5
FCFP_12	566058135	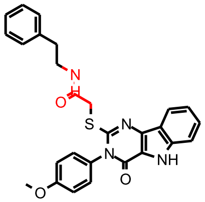 [*]CC(=O)N[*]	0.163	23 out of 24
**Top Features for negative contribution**				
**Fingerprint**	**Bit/Smiles**	**Feature Structure**	**Score**	**Irritant in training set**
FCFP_12	-1549163031	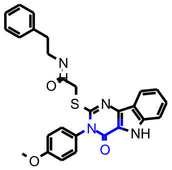 [*]N([*])C(=O)[c](:[*]):[*]	-0.623	16 out of 38
FCFP_12	-390563851	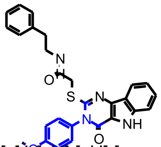 [*]O[c]1:[cH]:[cH]:[c](:[cH]:[cH]:1)N(C(=[*])[*])C(=[*])[*]	-0.592	0 out of 1
FCFP_12	580453787	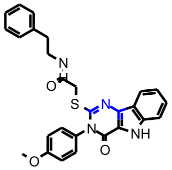 [*]C(=N[c](:[*]):[*])[*]	-0.132	2 out of 3

Molecule

TOPKAT_Rat_Female_FDA_None_vs_Carcinogen

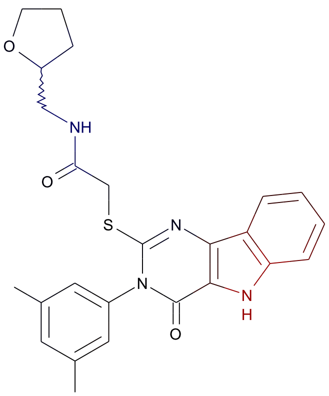

$C_{25}H_{26}N_4O_3S$
Molecular Weight: 462.56393
ALogP: 4.789
Rotatable Bonds: 6
Acceptors: 5
Donors: 2

Model Prediction

Prediction: Carcinogen
Probability: 0.284
Enrichment: 0.881
Bayesian Score: -0.756
Mahalanobis Distance: 10.6
Mahalanobis Distance p-value: 0.145

Prediction: Positive if the Bayesian score is above the estimated best cutoff value from minimizing the false positive and false negative rate.
Probability: The esimated probability that the sample is in the positive category. This assumes that the Bayesian score follows a normal distribution and is different from the prediction using a cutoff.
Enrichment: An estimate of enrichment, that is, the increased likelihood (versus random) of this sample being in the category.
Bayesian Score: The standard Laplacian-modified Bayesian score.
Mahalanobis Distance: The Mahalanobis distance (MD) is the distance to the center of the training data. The larger the MD, the less trustworthy the prediction.
Mahalanobis Distance p-value: The p-value gives the fraction of training data with an MD greater than or equal to the one for the given sample, assuming normally distributed data. The smaller the p-value, the less trustworthy the prediciton. For highly non-normal X properties (e.g., fingerprints), the MD p-value is wildly inaccurate.

Structural Similar Compounds

Name	Glimepride	Bicalutamide	Glyburide
Structure	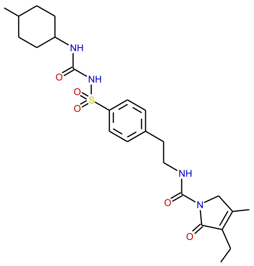	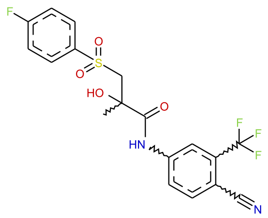	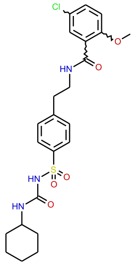
Actual Endpoint	Non-Carcinogen	Carcinogen	Non-Carcinogen
Predicted Endpoint	Non-Carcinogen	Carcinogen	Non-Carcinogen
Distance	0.654	0.668	0.673
Reference	US FDA (Centre for Drug Eval.& Res./Off. Testing & Res.) Sept. 1997	US FDA (Centre for Drug Eval.& Res./Off. Testing & Res.) Sept. 1997	US FDA (Centre for Drug Eval.& Res./Off. Testing & Res.) Sept. 1997

Model Applicability

Unknown features are fingerprint features in the query molecule, but not found or appearing too infrequently in the training set.

1. All properties and OPS components are within expected ranges.
2. Unknown ECFP_2 feature: -782828288: [*]C(=[*])[c]1:[nH]:[*]:[*]:[c]:1[*]
3. Unknown ECFP_2 feature: -962771238: [*]C(=[*])N(C(=[*])[*])[c](:[*]):[*]
4. Unknown ECFP_2 feature: 676970202: [*]S\C(=N\[*])\N([*])[*]
5. Unknown ECFP_2 feature: 1427820655: [*]CSC(=[*])[*]
6. Unknown ECFP_2 feature: -955816473: [*]SCC(=[*])[*]

Feature Contribution

Top features for positive contribution				
Fingerprint	Bit/Smiles	Feature Structure	Score	Carcinogen in training set

ECFP_12	558201926	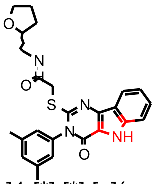 [*][c]1:[*]:[*]:[c](:[*]):[nH]:1	0.539	5 out of 8
ECFP_12	-91640731	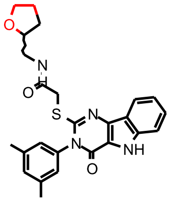 [*]1[*]OCC1	0.539	5 out of 8
ECFP_12	1099224616	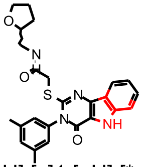 [*]:[cH]:[c]1:[nH]:[*]:[*]:[c]:1:[*]	0.456	6 out of 11
**Top Features for negative contribution**				
**Fingerprint**	**Bit/Smiles**	**Feature Structure**	**Score**	**Carcinogen in training set**
ECFP_12	497523368	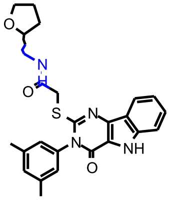 [*]CNC(=[*])[*]	-0.989	1 out of 14
ECFP_12	-84772164	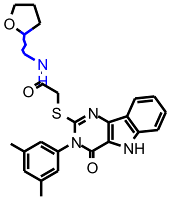 [*]NCC([*])[*]	-0.485	0 out of 2

ECFP_12	1997021792	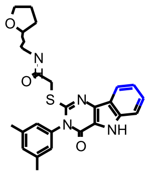 [*]:[cH]:[cH]:[cH]:[*]	-0.296	36 out of 156

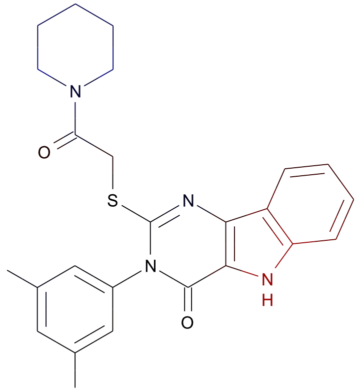

$C_{25}H_{26}N_4O_2S$
Molecular Weight: 446.56453
ALogP: 5.553
Rotatable Bonds: 4
Acceptors: 4
Donors: 1

Model Prediction

Prediction: Carcinogen
Probability: 0.314
Enrichment: 0.976
Bayesian Score: 0.741
Mahalanobis Distance: 10.8
Mahalanobis Distance p-value: 0.102

Prediction: Positive if the Bayesian score is above the estimated best cutoff value from minimizing the false positive and false negative rate.
Probability: The esimated probability that the sample is in the positive category. This assumes that the Bayesian score follows a normal distribution and is different from the prediction using a cutoff.
Enrichment: An estimate of enrichment, that is, the increased likelihood (versus random) of this sample being in the category.
Bayesian Score: The standard Laplacian-modified Bayesian score.
Mahalanobis Distance: The Mahalanobis distance (MD) is the distance to the center of the training data. The larger the MD, the less trustworthy the prediction.
Mahalanobis Distance p-value: The p-value gives the fraction of training data with an MD greater than or equal to the one for the given sample, assuming normally distributed data. The smaller the p-value, the less trustworthy the prediciton. For highly non-normal X properties (e.g., fingerprints), the MD p-value is wildly inaccurate.

Structural Similar Compounds

Name	Indomethacin	Ethynodiol	Simvastatin
Structure	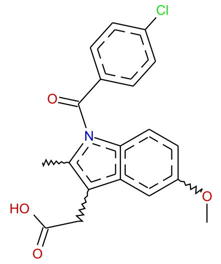	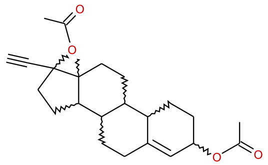	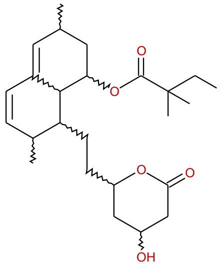
Actual Endpoint	Non-Carcinogen	Carcinogen	Carcinogen
Predicted Endpoint	Non-Carcinogen	Carcinogen	Carcinogen
Distance	0.671	0.694	0.713
Reference	US FDA (Centre for Drug Eval.& Res./Off. Testing & Res.) Sept. 1997	US FDA (Centre for Drug Eval.& Res./Off. Testing & Res.) Sept. 1997	US FDA (Centre for Drug Eval.& Res./Off. Testing & Res.) Sept. 1997

Model Applicability

Unknown features are fingerprint features in the query molecule, but not found or appearing too infrequently in the training set.

1. All properties and OPS components are within expected ranges.
2. Unknown ECFP_2 feature: -782828288: [*]C(=[*])[c]1:[nH]:[*]:[*]:[c]:1[*]
3. Unknown ECFP_2 feature: -962771238: [*]C(=[*])N(C(=[*])[*])[c](:[*]):[*]
4. Unknown ECFP_2 feature: 676970202: [*]S\C(=N\[*])\N([*])[*]
5. Unknown ECFP_2 feature: 1427820655: [*]CSC(=[*])[*]
6. Unknown ECFP_2 feature: -955816473: [*]SCC(=[*])[*]

Feature Contribution

Top features for positive contribution				
Fingerprint	Bit/Smiles	Feature Structure	Score	Carcinogen in training set

ECFP_12	1341750291	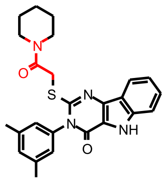 [*]CC(=O)N([*])[*]	0.613	2 out of 2
ECFP_12	558201926	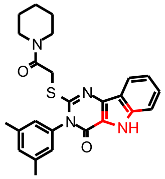 [*][c]1:[*]:[*]:[c](:[*]):[nH]:1	0.539	5 out of 8
ECFP_12	1099224616	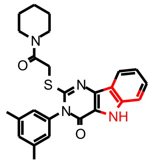 [*]:[cH]:[c]1:[nH]:[*]:[*]:[c]:1:[*]	0.456	6 out of 11
**Top Features for negative contribution**				
**Fingerprint**	**Bit/Smiles**	**Feature Structure**	**Score**	**Carcinogen in training set**
ECFP_12	-1102925512	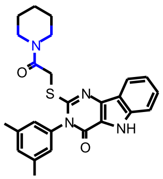 [*]CN(C[*])C(=[*])[*]	-1.06	0 out of 6
ECFP_12	662850656	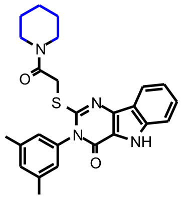 [*]1CCCCC1	-0.929	1 out of 13

ECFP_12	-1208612866	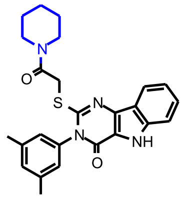 [*]N1CCCCC1	-0.485	0 out of 2

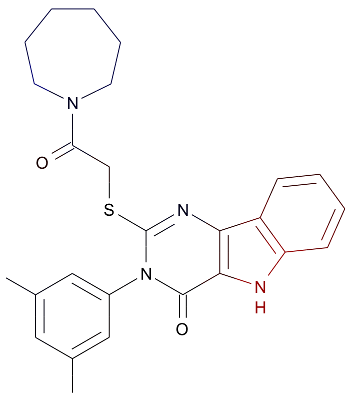

$C_{26}H_{28}N_4O_2S$
Molecular Weight: 460.59111
ALogP: 6.009
Rotatable Bonds: 4
Acceptors: 4
Donors: 1

Model Prediction

Prediction: Carcinogen
Probability: 0.315
Enrichment: 0.979
Bayesian Score: 0.786
Mahalanobis Distance: 10.9
Mahalanobis Distance p-value: 0.0855

Prediction: Positive if the Bayesian score is above the estimated best cutoff value from minimizing the false positive and false negative rate.
Probability: The esimated probability that the sample is in the positive category. This assumes that the Bayesian score follows a normal distribution and is different from the prediction using a cutoff.
Enrichment: An estimate of enrichment, that is, the increased likelihood (versus random) of this sample being in the category.
Bayesian Score: The standard Laplacian-modified Bayesian score.
Mahalanobis Distance: The Mahalanobis distance (MD) is the distance to the center of the training data. The larger the MD, the less trustworthy the prediction.
Mahalanobis Distance p-value: The p-value gives the fraction of training data with an MD greater than or equal to the one for the given sample, assuming normally distributed data. The smaller the p-value, the less trustworthy the prediciton. For highly non-normal X properties (e.g., fingerprints), the MD p-value is wildly inaccurate.

Structural Similar Compounds

Name	Indomethacin	Ethynodiol	Pimozide
Structure	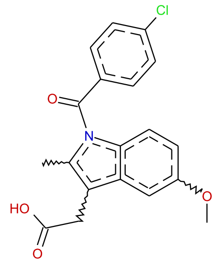	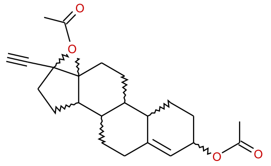	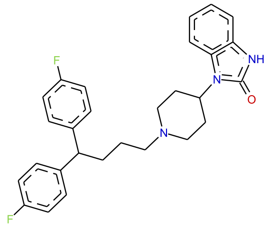
Actual Endpoint	Non-Carcinogen	Carcinogen	Non-Carcinogen
Predicted Endpoint	Non-Carcinogen	Carcinogen	Non-Carcinogen
Distance	0.718	0.718	0.728
Reference	US FDA (Centre for Drug Eval.& Res./Off. Testing & Res.) Sept. 1997	US FDA (Centre for Drug Eval.& Res./Off. Testing & Res.) Sept. 1997	US FDA (Centre for Drug Eval.& Res./Off. Testing & Res.) Sept. 1997

Model Applicability

Unknown features are fingerprint features in the query molecule, but not found or appearing too infrequently in the training set.

1. All properties and OPS components are within expected ranges.
2. Unknown ECFP_2 feature: -782828288: [*]C(=[*])[c]1:[nH]:[*]:[*]:[c]:1[*]
3. Unknown ECFP_2 feature: -962771238: [*]C(=[*])N(C(=[*])[*])[c](:[*]):[*]
4. Unknown ECFP_2 feature: 676970202: [*]S\C(=N\[*])\N([*])[*]
5. Unknown ECFP_2 feature: 1427820655: [*]CSC(=[*])[*]
6. Unknown ECFP_2 feature: -955816473: [*]SCC(=[*])[*]

Feature Contribution

Top features for positive contribution				
Fingerprint	Bit/Smiles	Feature Structure	Score	Carcinogen in training set

ECFP_12	1341750291	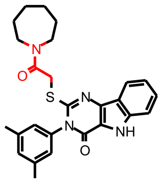 [*]CC(=O)N([*])[*]	0.613	2 out of 2
ECFP_12	558201926	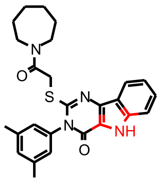 [*][c]1:[*]:[*]:[c](:[*]):[nH]:1	0.539	5 out of 8
ECFP_12	1099224616	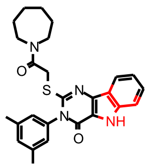 [*]:[cH]:[c]1:[nH]:[*]:[*]:[c]:1:[*]	0.456	6 out of 11
**Top Features for negative contribution**				
**Fingerprint**	**Bit/Smiles**	**Feature Structure**	**Score**	**Carcinogen in training set**
ECFP_12	-1102925512	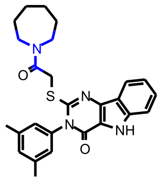 [*]CN(C[*])C(=[*])[*]	-1.06	0 out of 6
ECFP_12	662850656	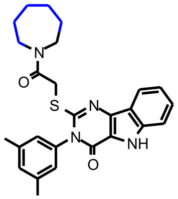 [*]1CCCCC1	-0.929	1 out of 13

ECFP_12	1997021792	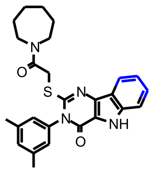 [*]:[cH]:[cH]:[cH]:[*]	-0.296	36 out of 156

#UNDEFINED

TOPKAT_Rat_Female_FDA_None_vs_Carcinogen

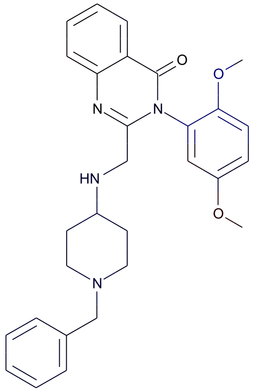

$C_{29}H_{32}N_4O_3$
Molecular Weight: 484.58938
ALogP: 3.743
Rotatable Bonds: 8
Acceptors: 6
Donors: 1

Model Prediction

Prediction: Non-Carcinogen
Probability: 0.215
Enrichment: 0.669
Bayesian Score: -5.54
Mahalanobis Distance: 13.8
Mahalanobis Distance p-value: 5.79e-006

Prediction: Positive if the Bayesian score is above the estimated best cutoff value from minimizing the false positive and false negative rate.
Probability: The esimated probability that the sample is in the positive category. This assumes that the Bayesian score follows a normal distribution and is different from the prediction using a cutoff.
Enrichment: An estimate of enrichment, that is, the increased likelihood (versus random) of this sample being in the category.
Bayesian Score: The standard Laplacian-modified Bayesian score.
Mahalanobis Distance: The Mahalanobis distance (MD) is the distance to the center of the training data. The larger the MD, the less trustworthy the prediction.
Mahalanobis Distance p-value: The p-value gives the fraction of training data with an MD greater than or equal to the one for the given sample, assuming normally distributed data. The smaller the p-value, the less trustworthy the prediciton. For highly non-normal X properties (e.g., fingerprints), the MD p-value is wildly inaccurate.

Structural Similar Compounds

Name	Emetine	Felodipine	Cisapride
Structure	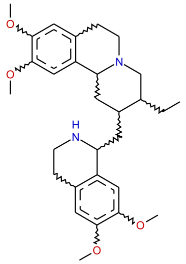	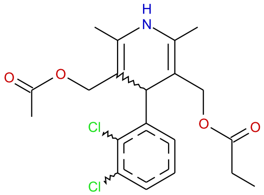	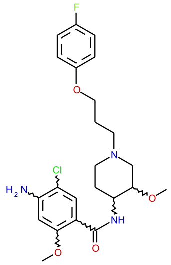
Actual Endpoint	Non-Carcinogen	Non-Carcinogen	Non-Carcinogen
Predicted Endpoint	Non-Carcinogen	Non-Carcinogen	Non-Carcinogen
Distance	0.604	0.634	0.644
Reference	US FDA (Centre for Drug Eval.& Res./Off. Testing & Res.) Sept. 1997	US FDA (Centre for Drug Eval.& Res./Off. Testing & Res.) Sept. 1997	US FDA (Centre for Drug Eval.& Res./Off. Testing & Res.) Sept. 1997

Model Applicability

Unknown features are fingerprint features in the query molecule, but not found or appearing too infrequently in the training set.

1. All properties and OPS components are within expected ranges.
2. Unknown ECFP_2 feature: -962771238: [*]C(=[*])N(C(=[*])[*])[c](:[*]):[*]

Feature Contribution

Top features for positive contribution

Fingerprint	Bit/Smiles	Feature Structure	Score	Carcinogen in training set
ECFP_12	2055803015	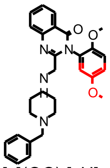 [*]:[cH]:[c](OC):[cH]:[*]	0.264	6 out of 14

ECFP_12	-1650219925	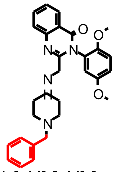 [*]C[c]1:[cH]:[cH]:[cH]:[cH]:[cH]:1	0.208	6 out of 15
ECFP_12	359396774	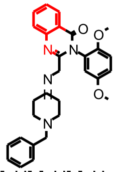 [*][c]1:[cH]:[cH]:[cH]:[cH]:[c]:1N=[*]	0.208	1 out of 2

Top Features for negative contribution

**Fingerprint**	**Bit/Smiles**	**Feature Structure**	**Score**	**Carcinogen in training set**
ECFP_12	2077607946	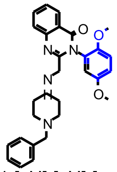 [*]O[c]1:[cH]:[cH]:[c]([*]):[*]:[c]:1[*]	-1.25	0 out of 8
ECFP_12	-281505363	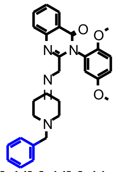 [*][c]1:[cH]:[cH]:[cH]:[cH]:[cH]:1	-0.56	11 out of 64
ECFP_12	1571214559	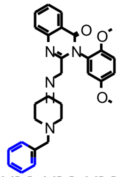 [*]1:[cH]:[cH]:[cH]:[cH]:[cH]:1	-0.56	11 out of 64

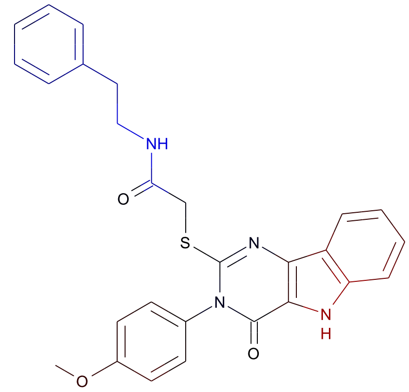

$C_{27}H_{24}N_4O_3S$

Molecular Weight: 484.56946

ALogP: 5.346

Rotatable Bonds: 8

Acceptors: 5

Donors: 2

Model Prediction

Prediction: Non-Carcinogen

Probability: 0.251

Enrichment: 0.78

Bayesian Score: -2.71

Mahalanobis Distance: 13.8

Mahalanobis Distance p-value: 5.56e-006

Prediction: Positive if the Bayesian score is above the estimated best cutoff value from minimizing the false positive and false negative rate.
Probability: The esimated probability that the sample is in the positive category. This assumes that the Bayesian score follows a normal distribution and is different from the prediction using a cutoff.
Enrichment: An estimate of enrichment, that is, the increased likelihood (versus random) of this sample being in the category.
Bayesian Score: The standard Laplacian-modified Bayesian score.
Mahalanobis Distance: The Mahalanobis distance (MD) is the distance to the center of the training data. The larger the MD, the less trustworthy the prediction.
Mahalanobis Distance p-value: The p-value gives the fraction of training data with an MD greater than or equal to the one for the given sample, assuming normally distributed data. The smaller the p-value, the less trustworthy the prediciton. For highly non-normal X properties (e.g., fingerprints), the MD p-value is wildly inaccurate.

Structural Similar Compounds

Name	Glyburide	Bitolterol	Glimepride
Structure	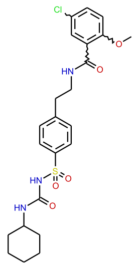	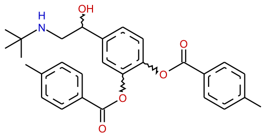	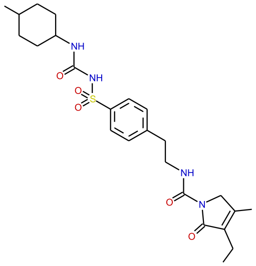
Actual Endpoint	Non-Carcinogen	Non-Carcinogen	Non-Carcinogen
Predicted Endpoint	Non-Carcinogen	Non-Carcinogen	Non-Carcinogen
Distance	0.642	0.657	0.675
Reference	US FDA (Centre for Drug Eval.& Res./Off. Testing & Res.) Sept. 1997	US FDA (Centre for Drug Eval.& Res./Off. Testing & Res.) Sept. 1997	US FDA (Centre for Drug Eval.& Res./Off. Testing & Res.) Sept. 1997

Model Applicability

Unknown features are fingerprint features in the query molecule, but not found or appearing too infrequently in the training set.

1. All properties and OPS components are within expected ranges.
2. Unknown ECFP_2 feature: -782828288: [*]C(=[*])[c]1:[nH]:[*]:[*]:[c]:1[*]
3. Unknown ECFP_2 feature: -962771238: [*]C(=[*])N(C(=[*])[*])[c](:[*]):[*]
4. Unknown ECFP_2 feature: 676970202: [*]S\C(=N\[*])\N([*])[*]
5. Unknown ECFP_2 feature: 1427820655: [*]CSC(=[*])[*]
6. Unknown ECFP_2 feature: -955816473: [*]SCC(=[*])[*]

Feature Contribution

Top features for positive contribution				
Fingerprint	Bit/Smiles	Feature Structure	Score	Carcinogen in training set

ECFP_12	558201926	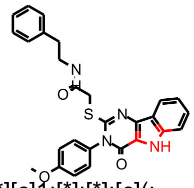 [*][c]1:[*]:[*]:[c](:[*]):[nH]:1	0.539	5 out of 8
ECFP_12	1099224616	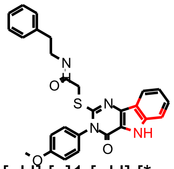 [*]:[cH]:[c]1:[nH]:[*]:[*]:[c]:1:[*]	0.456	6 out of 11
ECFP_12	1639827160	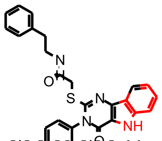 [*]1:[*]:[c]2:[*]:[cH]:[cH]:[cH]:[c]:2:[nH]:1	0.45	4 out of 7

Top Features for negative contribution

Fingerprint	Bit/Smiles	Feature Structure	Score	Carcinogen in training set
ECFP_12	497523368	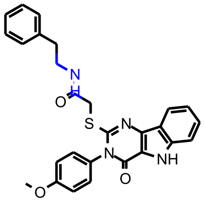 [*]CNC(=[*])[*]	-0.989	1 out of 14
ECFP_12	-2058216030	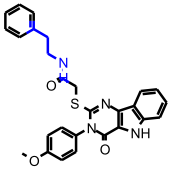 [*]C(=[*])NCC[c](:[*]):[*]	-0.811	0 out of 4

ECFP_12	-104952638	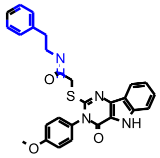<br>[*]C(=[*])NCC[c]1:[cH<br>]:[cH]:[*]:[cH]:[cH]<br>:1	-0.811	0 out of 4

Molecule

TOPKAT_Rat_Female_FDA_Single_vs_Multiple

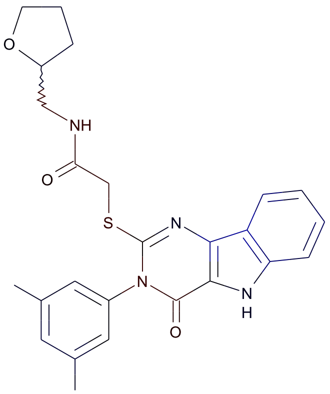

$C_{25}H_{26}N_4O_3S$
Molecular Weight: 462.56393
ALogP: 4.789
Rotatable Bonds: 6
Acceptors: 5
Donors: 2

Structural Similar Compounds

Name	Bicalutamide	Simvastatin	Moricizine
Structure	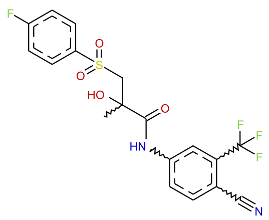	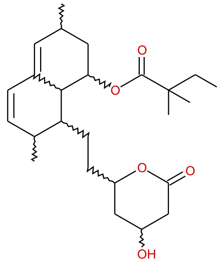	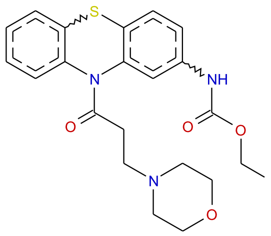
Actual Endpoint	Multiple-Carcinogen	Multiple-Carcinogen	Single-Carcinogen
Predicted Endpoint	Multiple-Carcinogen	Multiple-Carcinogen	Single-Carcinogen
Distance	0.619	0.683	0.686
Reference	US FDA (Centre for Drug Eval.& Res./Off. Testing & Res.) Sept. 1997	US FDA (Centre for Drug Eval.& Res./Off. Testing & Res.) Sept. 1997	US FDA (Centre for Drug Eval.& Res./Off. Testing & Res.) Sept. 1997

Model Prediction

Prediction: Single-Carcinogen
Probability: 0.49
Enrichment: 1.31
Bayesian Score: -0.764
Mahalanobis Distance: 14.2
Mahalanobis Distance p-value: 1.83e-005

Prediction: Positive if the Bayesian score is above the estimated best cutoff value from minimizing the false positive and false negative rate.
Probability: The esimated probability that the sample is in the positive category. This assumes that the Bayesian score follows a normal distribution and is different from the prediction using a cutoff.
Enrichment: An estimate of enrichment, that is, the increased likelihood (versus random) of this sample being in the category.
Bayesian Score: The standard Laplacian-modified Bayesian score.
Mahalanobis Distance: The Mahalanobis distance (MD) is the distance to the center of the training data. The larger the MD, the less trustworthy the prediction.
Mahalanobis Distance p-value: The p-value gives the fraction of training data with an MD greater than or equal to the one for the given sample, assuming normally distributed data. The smaller the p-value, the less trustworthy the prediciton. For highly non-normal X properties (e.g., fingerprints), the MD p-value is wildly inaccurate.

Model Applicability

Unknown features are fingerprint features in the query molecule, but not found or appearing too infrequently in the training set.

1. OPS PC12 out of range. Value: 3.2192. Training min, max, SD, explained variance: -2.8991, 3.0113, 1.313, 0.0255.

Feature Contribution

Top features for positive contribution

Fingerprint	Bit/Smiles	Feature Structure	Score	Multiple-Carcinogen in training set
SCFP_4	17	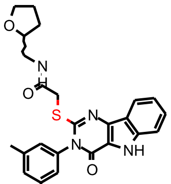 [*]S[*]	0.548	10 out of 17

SCFP_4	1205586762	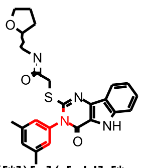 [*]N([*])[c](:[cH]:[*]):[cH]:[*]	0.451	7 out of 13
SCFP_4	1257084377	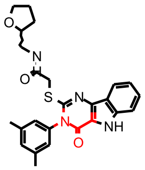 [*]N([*])C(=O)[c](:[*]):[*]	0.44	3 out of 5
**Top Features for negative contribution**				
**Fingerprint**	**Bit/Smiles**	**Feature Structure**	**Score**	**Multiple-Carcinogen in training set**
SCFP_4	622342378	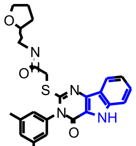 [*][c]1:[nH]:[c]2:[cH]:[cH]:[*]:[cH]:[c]: 2:[c]:1[*]	-0.816	0 out of 4
SCFP_4	112346096	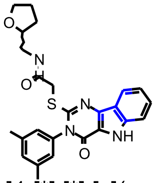 [*][c]1:[*]:[*]:[c](: [*]):[c]:1:[cH]:[*]	-0.73	1 out of 10
SCFP_4	1851000357	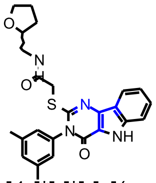 [*][c]1:[*]:[*]:[c](: [*]):[c]:1N=[*]	-0.489	0 out of 2

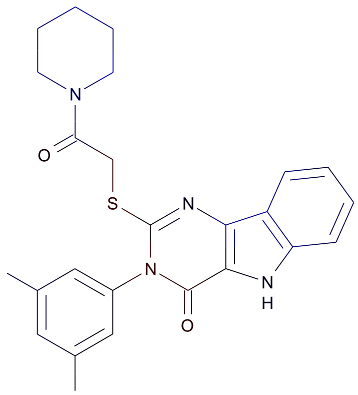

$C_{25}H_{26}N_4O_2S$

Molecular Weight: 446.56453

ALogP: 5.553

Rotatable Bonds: 4

Acceptors: 4

Donors: 1

Model Prediction

Prediction: Single-Carcinogen

Probability: 0.374

Enrichment: 1

Bayesian Score: -3.69

Mahalanobis Distance: 12.9

Mahalanobis Distance p-value: 0.000297

Prediction: Positive if the Bayesian score is above the estimated best cutoff value from minimizing the false positive and false negative rate.
Probability: The esimated probability that the sample is in the positive category. This assumes that the Bayesian score follows a normal distribution and is different from the prediction using a cutoff.
Enrichment: An estimate of enrichment, that is, the increased likelihood (versus random) of this sample being in the category.
Bayesian Score: The standard Laplacian-modified Bayesian score.
Mahalanobis Distance: The Mahalanobis distance (MD) is the distance to the center of the training data. The larger the MD, the less trustworthy the prediction.
Mahalanobis Distance p-value: The p-value gives the fraction of training data with an MD greater than or equal to the one for the given sample, assuming normally distributed data. The smaller the p-value, the less trustworthy the prediciton. For highly non-normal X properties (e.g., fingerprints), the MD p-value is wildly inaccurate.

Structural Similar Compounds

Name	Lansoprazole	Simvastatin	Ethynodiol
Structure	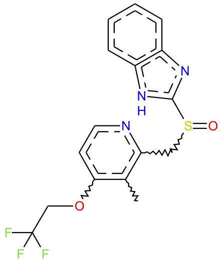	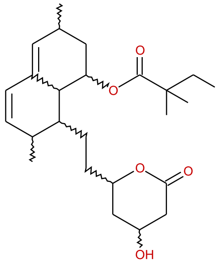	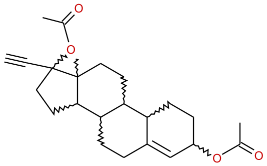
Actual Endpoint	Single-Carcinogen	Multiple-Carcinogen	Single-Carcinogen
Predicted Endpoint	Single-Carcinogen	Multiple-Carcinogen	Single-Carcinogen
Distance	0.696	0.704	0.706
Reference	US FDA (Centre for Drug Eval.& Res./Off. Testing & Res.) Sept. 1997	US FDA (Centre for Drug Eval.& Res./Off. Testing & Res.) Sept. 1997	US FDA (Centre for Drug Eval.& Res./Off. Testing & Res.) Sept. 1997

Model Applicability

Unknown features are fingerprint features in the query molecule, but not found or appearing too infrequently in the training set.

1. All properties and OPS components are within expected ranges.

Feature Contribution

Top features for positive contribution

Fingerprint	Bit/Smiles	Feature Structure	Score	Multiple-Carcinogen in training set
SCFP_4	17	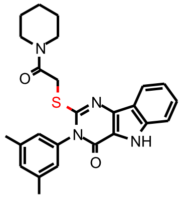 [*]S[*]	0.548	10 out of 17

SCFP_4	1205586762	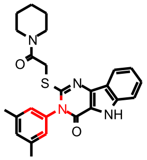 [*]N([*])[c](:[cH]:[*]):[cH]:[*]	0.451	7 out of 13
SCFP_4	1257084377	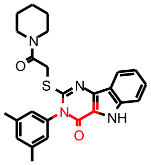 [*]N([*])C(=O)[c](:[*]):[*]	0.44	3 out of 5

Top Features for negative contribution

Fingerprint	Bit/Smiles	Feature Structure	Score	Multiple-Carcinogen in training set
SCFP_4	1175638033	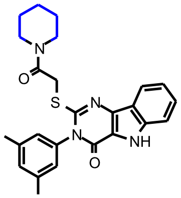 [*]1CCCCC1	-1.17	0 out of 7
SCFP_4	-1343150366	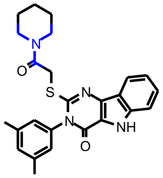 [*]CN(C[*])C(=[*])[*]	-0.946	0 out of 5
SCFP_4	622342378	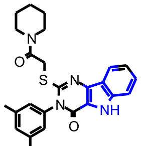 [*][c]1:[nH]:[c]2:[cH]:[cH]:[*]:[cH]:[c]: 2:[c]:1[*]	-0.816	0 out of 4

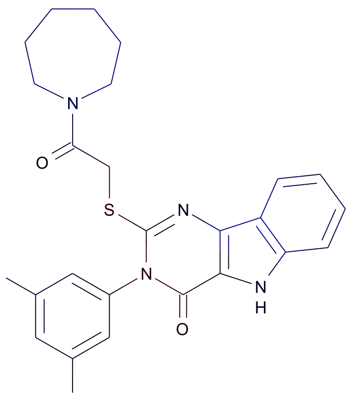

$C_{26}H_{28}N_4O_2S$
Molecular Weight: 460.59111
ALogP: 6.009
Rotatable Bonds: 4
Acceptors: 4
Donors: 1

Model Prediction

Prediction: Single-Carcinogen
Probability: 0.337
Enrichment: 0.902
Bayesian Score: -4.41
Mahalanobis Distance: 12.9
Mahalanobis Distance p-value: 0.000296

Prediction: Positive if the Bayesian score is above the estimated best cutoff value from minimizing the false positive and false negative rate.
Probability: The esimated probability that the sample is in the positive category. This assumes that the Bayesian score follows a normal distribution and is different from the prediction using a cutoff.
Enrichment: An estimate of enrichment, that is, the increased likelihood (versus random) of this sample being in the category.
Bayesian Score: The standard Laplacian-modified Bayesian score.
Mahalanobis Distance: The Mahalanobis distance (MD) is the distance to the center of the training data. The larger the MD, the less trustworthy the prediction.
Mahalanobis Distance p-value: The p-value gives the fraction of training data with an MD greater than or equal to the one for the given sample, assuming normally distributed data. The smaller the p-value, the less trustworthy the prediciton. For highly non-normal X properties (e.g., fingerprints), the MD p-value is wildly inaccurate.

Structural Similar Compounds

Name	Simvastatin	Ethynodiol	Lansoprazole
Structure	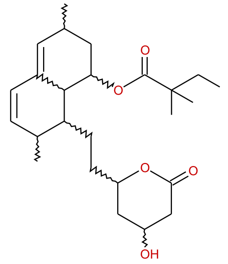	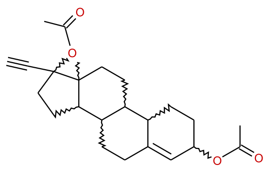	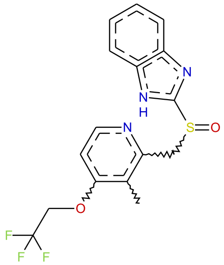
Actual Endpoint	Multiple-Carcinogen	Single-Carcinogen	Single-Carcinogen
Predicted Endpoint	Multiple-Carcinogen	Single-Carcinogen	Single-Carcinogen
Distance	0.725	0.726	0.740
Reference	US FDA (Centre for Drug Eval.& Res./Off. Testing & Res.) Sept. 1997	US FDA (Centre for Drug Eval.& Res./Off. Testing & Res.) Sept. 1997	US FDA (Centre for Drug Eval.& Res./Off. Testing & Res.) Sept. 1997

Model Applicability

Unknown features are fingerprint features in the query molecule, but not found or appearing too infrequently in the training set.

1. All properties and OPS components are within expected ranges.

Feature Contribution

Top features for positive contribution

Fingerprint	Bit/Smiles	Feature Structure	Score	Multiple-Carcinogen in training set
SCFP_4	17	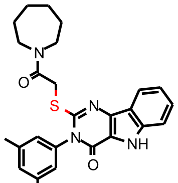 [*]S[*]	0.548	10 out of 17

SCFP_4	1205586762	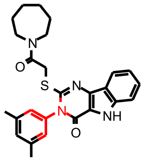 [*]N([*])[c](:[cH]:[*]):[cH]:[*]	0.451	7 out of 13
SCFP_4	1257084377	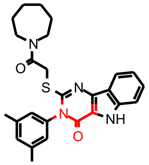 [*]N([*])C(=O)[c](:[*]):[*]	0.44	3 out of 5

Top Features for negative contribution

Fingerprint	Bit/Smiles	Feature Structure	Score	Multiple-Carcinogen in training set
SCFP_4	1175638033	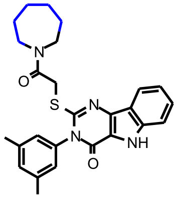 [*]1CCCCC1	-1.17	0 out of 7
SCFP_4	-1343150366	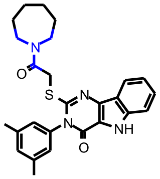 [*]CN(C[*])C(=[*])[*]	-0.946	0 out of 5
SCFP_4	622342378	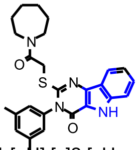 [*][c]1:[nH]:[c]2:[cH]:[cH]:[*]:[cH]:[c]: 2:[c]:1[*]	-0.816	0 out of 4

Molecule

TOPKAT_Rat_Female_NTP

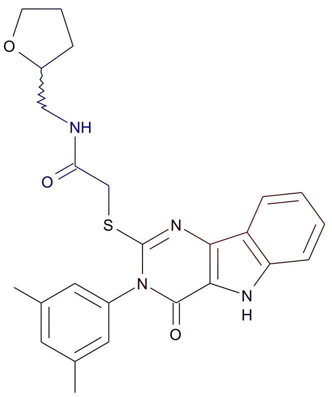

$C_{25}H_{26}N_4O_3S$
Molecular Weight: 462.56393
ALogP: 4.789
Rotatable Bonds: 6
Acceptors: 5
Donors: 2

Structural Similar Compounds

Name	Rhodamine 6G	Curcumin	Ochratoxin A
Structure	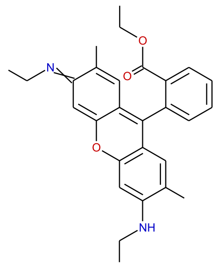	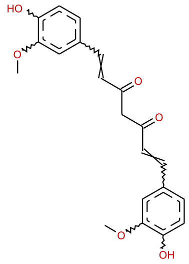	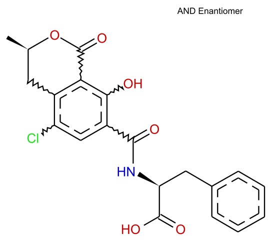
Actual Endpoint	Carcinogen	Carcinogen	Carcinogen
Predicted Endpoint	Carcinogen	Carcinogen	Carcinogen
Distance	0.672	0.689	0.699
Reference	NTP364	NTP427	NTP358 & CPDB

Model Prediction

Prediction: Non-Carcinogen
Probability: 0.477
Enrichment: 1.05
Bayesian Score: -1.58
Mahalanobis Distance: 13.8
Mahalanobis Distance p-value: 6.92e-011

Prediction: Positive if the Bayesian score is above the estimated best cutoff value from minimizing the false positive and false negative rate.
Probability: The esimated probability that the sample is in the positive category. This assumes that the Bayesian score follows a normal distribution and is different from the prediction using a cutoff.
Enrichment: An estimate of enrichment, that is, the increased likelihood (versus random) of this sample being in the category.
Bayesian Score: The standard Laplacian-modified Bayesian score.
Mahalanobis Distance: The Mahalanobis distance (MD) is the distance to the center of the training data. The larger the MD, the less trustworthy the prediction.
Mahalanobis Distance p-value: The p-value gives the fraction of training data with an MD greater than or equal to the one for the given sample, assuming normally distributed data. The smaller the p-value, the less trustworthy the prediciton. For highly non-normal X properties (e.g., fingerprints), the MD p-value is wildly inaccurate.

Model Applicability

Unknown features are fingerprint features in the query molecule, but not found or appearing too infrequently in the training set.

1. All properties and OPS components are within expected ranges.
2. Unknown FCFP_2 feature: 203707511: [*]C(=[*])[c]1:[nH]:[*]:[*]:[c]:1[*]
3. Unknown FCFP_2 feature: 580453787: [*]C(=N[c](:[*]):[*])[*]
4. Unknown FCFP_2 feature: 307448885: [*]:[cH]:[c]1:[nH]:[*]:[*]:[c]:1:[*]

Feature Contribution

Top features for positive contribution

Fingerprint	Bit/Smiles	Feature Structure	Score	Carcinogen in training set
FCFP_12	-1320007763	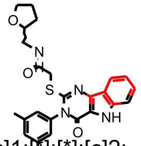 [*][c]1:[*]:[*]:[c]2:[*]:[cH]:[cH]:[cH]:[c]:1:2	0.475	10 out of 14

FCFP_12	-387072142	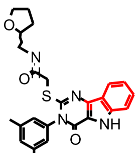 [*][c]1:[*]:[*]:[c]2:[cH]:[cH]:[cH]:[cH]:[c]:1:2	0.436	7 out of 10
FCFP_12	307419094	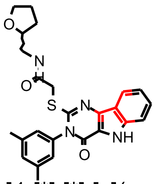 [*][c]1:[*]:[*]:[c](:[*]):[c]:1:[cH]:[*]	0.394	11 out of 17
**Top Features for negative contribution**				
**Fingerprint**	**Bit/Smiles**	**Feature Structure**	**Score**	**Carcinogen in training set**
FCFP_12	-925504834	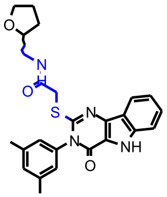 [*]CNC(=O)CS[*]	-0.982	0 out of 4
FCFP_12	-1272798659	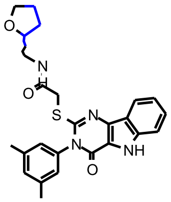 [*]C1[*][*]CC1	-0.706	10 out of 51
FCFP_12	566058135	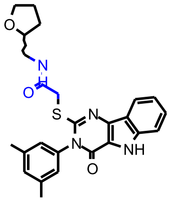 [*]CC(=O)N[*]	-0.705	3 out of 17

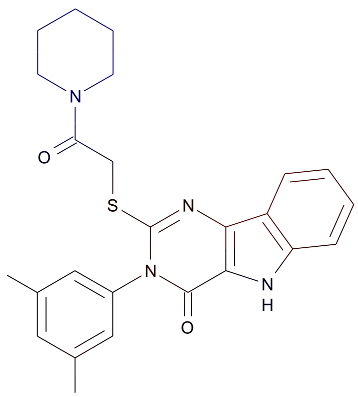

$C_{25}H_{26}N_4O_2S$
Molecular Weight: 446.56453
ALogP: 5.553
Rotatable Bonds: 4
Acceptors: 4
Donors: 1

Model Prediction

Prediction: Non-Carcinogen
Probability: 0.474
Enrichment: 1.04
Bayesian Score: -1.66
Mahalanobis Distance: 11.6
Mahalanobis Distance p-value: 4.28e-006

Prediction: Positive if the Bayesian score is above the estimated best cutoff value from minimizing the false positive and false negative rate.
Probability: The esimated probability that the sample is in the positive category. This assumes that the Bayesian score follows a normal distribution and is different from the prediction using a cutoff.
Enrichment: An estimate of enrichment, that is, the increased likelihood (versus random) of this sample being in the category.
Bayesian Score: The standard Laplacian-modified Bayesian score.
Mahalanobis Distance: The Mahalanobis distance (MD) is the distance to the center of the training data. The larger the MD, the less trustworthy the prediction.
Mahalanobis Distance p-value: The p-value gives the fraction of training data with an MD greater than or equal to the one for the given sample, assuming normally distributed data. The smaller the p-value, the less trustworthy the prediciton. For highly non-normal X properties (e.g., fingerprints), the MD p-value is wildly inaccurate.

Structural Similar Compounds

Name	Rhodamine 6G	C.I.PIGMENT RED 3	Tricresyl Phosphate
Structure	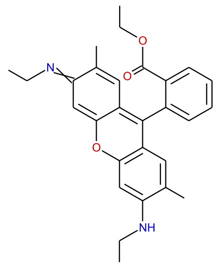	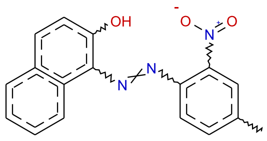	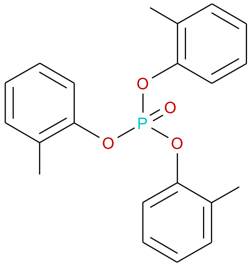
Actual Endpoint	Carcinogen	Carcinogen	Non-Carcinogen
Predicted Endpoint	Carcinogen	Carcinogen	Non-Carcinogen
Distance	0.670	0.690	0.695
Reference	NTP364	TR-407	NTP433

Model Applicability

Unknown features are fingerprint features in the query molecule, but not found or appearing too infrequently in the training set.

1. All properties and OPS components are within expected ranges.
2. Unknown FCFP_2 feature: 203707511: [*]C(=[*])[c]1:[nH]:[*]:[*]:[c]:1[*]
3. Unknown FCFP_2 feature: 580453787: [*]C(=N[c](:[*]):[*])[*]
4. Unknown FCFP_2 feature: 307448885: [*]:[cH]:[c]1:[nH]:[*]:[*]:[c]:1:[*]

Feature Contribution

Top features for positive contribution

Fingerprint	Bit/Smiles	Feature Structure	Score	Carcinogen in training set
FCFP_12	-1320007763	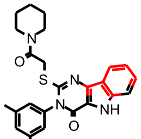 [*][c]1:[*]:[*]:[c]2:[*]:[cH]:[cH]:[cH]:[c]:1:2	0.475	10 out of 14

FCFP_12	-387072142	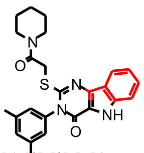 [*][c]1:[*]:[*]:[c]2:[cH]:[cH]:[cH]:[cH]:[c]:1:2	0.436	7 out of 10
FCFP_12	307419094	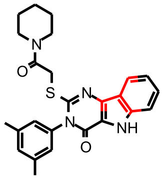 [*][c]1:[*]:[*]:[c](:[*]):[c]:1:[cH]:[*]	0.394	11 out of 17
**Top Features for negative contribution**				
**Fingerprint**	**Bit/Smiles**	**Feature Structure**	**Score**	**Carcinogen in training set**
FCFP_12	1175638033	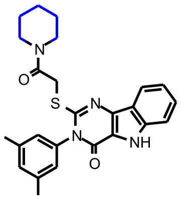 [*]1CCCCC1	-1.23	1 out of 14
FCFP_12	-1553874037	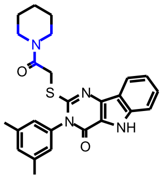 [*]CN(C[*])C(=[*])[*]	-0.774	1 out of 8
FCFP_12	565998553	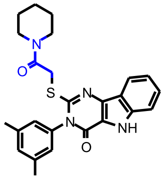 [*]CC(=O)N([*])[*]	-0.751	4 out of 23

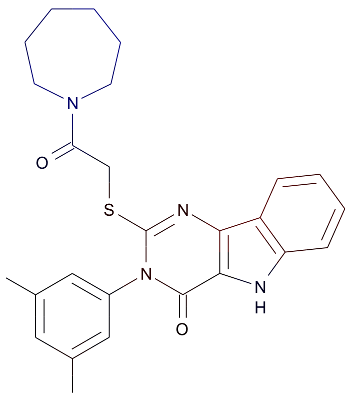

$C_{26}H_{28}N_4O_2S$
Molecular Weight: 460.59111
ALogP: 6.009
Rotatable Bonds: 4
Acceptors: 4
Donors: 1

Structural Similar Compounds

Name	Rhodamine 6G	Tricresyl Phosphate	4;4'-THIOBIS(6-t-BUTYL-m-CRESOL)
Structure	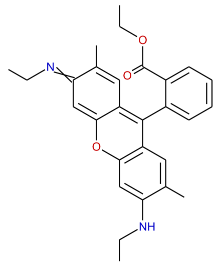	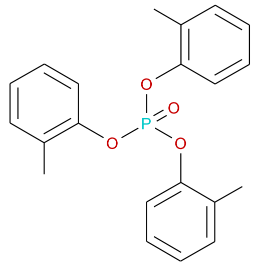	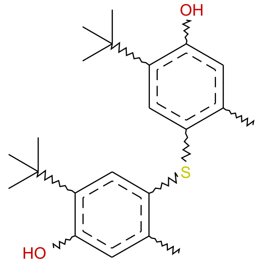
Actual Endpoint	Carcinogen	Non-Carcinogen	Non-Carcinogen
Predicted Endpoint	Carcinogen	Non-Carcinogen	Non-Carcinogen
Distance	0.673	0.695	0.711
Reference	NTP364	NTP433	TR-435

Model Prediction

Prediction: Non-Carcinogen
Probability: 0.464
Enrichment: 1.02
Bayesian Score: -2.01
Mahalanobis Distance: 11.6
Mahalanobis Distance p-value: 4.09e-006

Prediction: Positive if the Bayesian score is above the estimated best cutoff value from minimizing the false positive and false negative rate.
Probability: The esimated probability that the sample is in the positive category. This assumes that the Bayesian score follows a normal distribution and is different from the prediction using a cutoff.
Enrichment: An estimate of enrichment, that is, the increased likelihood (versus random) of this sample being in the category.
Bayesian Score: The standard Laplacian-modified Bayesian score.
Mahalanobis Distance: The Mahalanobis distance (MD) is the distance to the center of the training data. The larger the MD, the less trustworthy the prediction.
Mahalanobis Distance p-value: The p-value gives the fraction of training data with an MD greater than or equal to the one for the given sample, assuming normally distributed data. The smaller the p-value, the less trustworthy the prediciton. For highly non-normal X properties (e.g., fingerprints), the MD p-value is wildly inaccurate.

Model Applicability

Unknown features are fingerprint features in the query molecule, but not found or appearing too infrequently in the training set.

1. All properties and OPS components are within expected ranges.
2. Unknown FCFP_2 feature: 203707511: [*]C(=[*])[c]1:[nH]:[*]:[*]:[c]:1[*]
3. Unknown FCFP_2 feature: 580453787: [*]C(=N[c](:[*]):[*])[*]
4. Unknown FCFP_2 feature: 307448885: [*]:[cH]:[c]1:[nH]:[*]:[*]:[c]:1:[*]

Feature Contribution

Top features for positive contribution				
**Fingerprint**	**Bit/Smiles**	**Feature Structure**	**Score**	**Carcinogen in training set**
FCFP_12	-1320007763	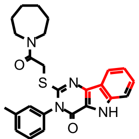 [*][c]1:[*]:[*]:[c]2:[*]:[cH]:[cH]:[cH]:[c]:1:2	0.475	10 out of 14

FCFP_12	-387072142	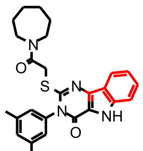<br>[*][c]1:[*]:[*]:[c]2:[cH]:[cH]:[cH]:[cH]:[c]:1:2	0.436	7 out of 10
FCFP_12	307419094	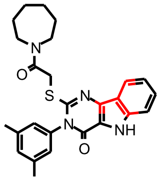<br>[*][c]1:[*]:[*]:[c](:[*]):[c]:1:[cH]:[*]	0.394	11 out of 17
**Top Features for negative contribution**				
**Fingerprint**	**Bit/Smiles**	**Feature Structure**	**Score**	**Carcinogen in training set**
FCFP_12	1175638033	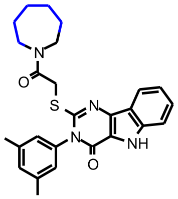<br>[*]1CCCCCC1	-1.23	1 out of 14
FCFP_12	-1553874037	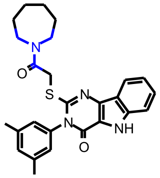<br>[*]CN(C[*])C(=[*])[*]	-0.774	1 out of 8
FCFP_12	565998553	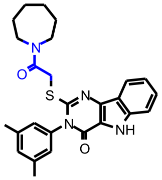<br>[*]CC(=O)N([*])[*]	-0.751	4 out of 23

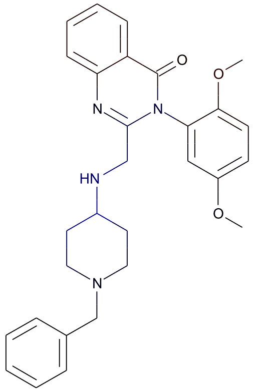

$C_{29}H_{32}N_4O_3$
Molecular Weight: 484.58938
ALogP: 3.743
Rotatable Bonds: 8
Acceptors: 6
Donors: 1

Structural Similar Compounds

Name	Rhodamine 6G	Curcumin	Butyl Benzyl Phthalate
Structure	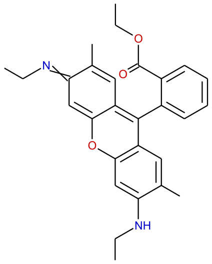	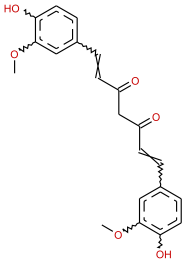	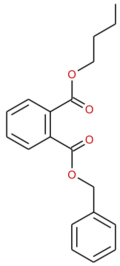
Actual Endpoint	Carcinogen	Carcinogen	Carcinogen
Predicted Endpoint	Carcinogen	Carcinogen	Carcinogen
Distance	0.660	0.702	0.782
Reference	NTP364	NTP427	NTP458 & CPDB

Model Prediction

Prediction: Non-Carcinogen
Probability: 0.424
Enrichment: 0.932
Bayesian Score: -3.22
Mahalanobis Distance: 11.1
Mahalanobis Distance p-value: 3.75e-005

Prediction: Positive if the Bayesian score is above the estimated best cutoff value from minimizing the false positive and false negative rate.
Probability: The esimated probability that the sample is in the positive category. This assumes that the Bayesian score follows a normal distribution and is different from the prediction using a cutoff.
Enrichment: An estimate of enrichment, that is, the increased likelihood (versus random) of this sample being in the category.
Bayesian Score: The standard Laplacian-modified Bayesian score.
Mahalanobis Distance: The Mahalanobis distance (MD) is the distance to the center of the training data. The larger the MD, the less trustworthy the prediction.
Mahalanobis Distance p-value: The p-value gives the fraction of training data with an MD greater than or equal to the one for the given sample, assuming normally distributed data. The smaller the p-value, the less trustworthy the prediciton. For highly non-normal X properties (e.g., fingerprints), the MD p-value is wildly inaccurate.

Model Applicability

Unknown features are fingerprint features in the query molecule, but not found or appearing too infrequently in the training set.

1. All properties and OPS components are within expected ranges.
2. Unknown FCFP_2 feature: 580453787: [*]C(=N[c](:[*]):[*])[*]
3. Unknown FCFP_2 feature: 906798516: [*]N([*])C[c](:[*]):[*]

Feature Contribution

Top features for positive contribution				
**Fingerprint**	**Bit/Smiles**	**Feature Structure**	**Score**	**Carcinogen in training set**
FCFP_12	356782498	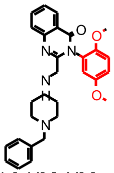 [*]O[c]1:[cH]:[cH]:[c](OC):[cH]:[c]:1[*]	0.492	2 out of 2

FCFP_12	-2090462286	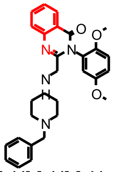<br>[*][c]1:[cH]:[cH]:[cH<br>]:[cH]:[c]:1N=[*]	0.32	9 out of 15
FCFP_12	1674451008	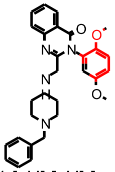<br>[*]O[c]1:[cH]:[cH]:[c<br>]([*]):[*]:[c]:1[*]	0.291	26 out of 46

Top Features for negative contribution

Fingerprint	Bit/Smiles	Feature Structure	Score	Carcinogen in training set
FCFP_12	565998553	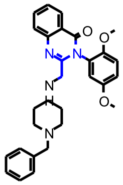<br>[*]CC(=O)N([*])[*]	-0.751	4 out of 23
FCFP_12	-1043250487	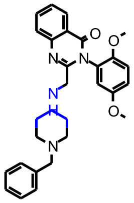<br>[*]CC(C[*])N[*]	-0.711	4 out of 22
FCFP_12	-1272798659	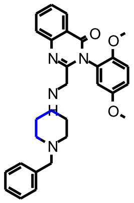<br>[*]C1[*][*]CC1	-0.706	10 out of 51

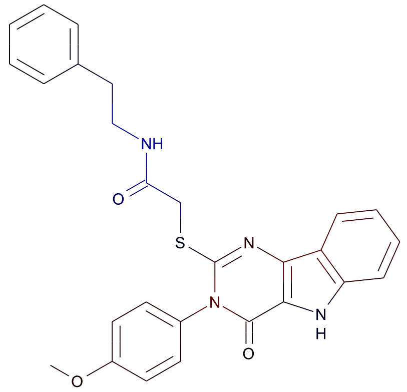

$C_{27}H_{24}N_4O_3S$
Molecular Weight: 484.56946
ALogP: 5.346
Rotatable Bonds: 8
Acceptors: 5
Donors: 2

Model Prediction

Prediction: Non-Carcinogen
Probability: 0.504
Enrichment: 1.11
Bayesian Score: -0.643
Mahalanobis Distance: 12.3
Mahalanobis Distance p-value: 1.39e-007

Prediction: Positive if the Bayesian score is above the estimated best cutoff value from minimizing the false positive and false negative rate.
Probability: The esimated probability that the sample is in the positive category. This assumes that the Bayesian score follows a normal distribution and is different from the prediction using a cutoff.
Enrichment: An estimate of enrichment, that is, the increased likelihood (versus random) of this sample being in the category.
Bayesian Score: The standard Laplacian-modified Bayesian score.
Mahalanobis Distance: The Mahalanobis distance (MD) is the distance to the center of the training data. The larger the MD, the less trustworthy the prediction.
Mahalanobis Distance p-value: The p-value gives the fraction of training data with an MD greater than or equal to the one for the given sample, assuming normally distributed data. The smaller the p-value, the less trustworthy the prediciton. For highly non-normal X properties (e.g., fingerprints), the MD p-value is wildly inaccurate.

Structural Similar Compounds

Name	Rhodamine 6G	Curcumin	C.I. pigment red 23
Structure	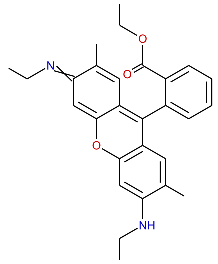	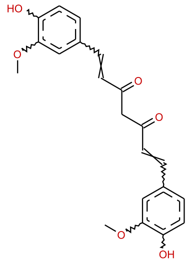	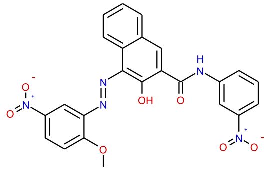
Actual Endpoint	Carcinogen	Carcinogen	Non-Carcinogen
Predicted Endpoint	Carcinogen	Carcinogen	Non-Carcinogen
Distance	0.660	0.705	0.767
Reference	NTP364	NTP427	NTP411

Model Applicability

Unknown features are fingerprint features in the query molecule, but not found or appearing too infrequently in the training set.

1. All properties and OPS components are within expected ranges.
2. Unknown FCFP_2 feature: 203707511: [*]C(=[*])[c]1:[nH]:[*]:[*]:[c]:1[*]
3. Unknown FCFP_2 feature: 580453787: [*]C(=N[c](:[*]):[*])[*]
4. Unknown FCFP_2 feature: 307448885: [*]:[cH]:[c]1:[nH]:[*]:[*]:[c]:1:[*]

Feature Contribution

Top features for positive contribution

Fingerprint	Bit/Smiles	Feature Structure	Score	Carcinogen in training set
FCFP_12	356782498	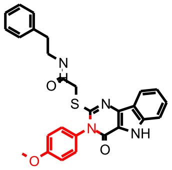 [*]O[c]1:[cH]:[cH]:[c](OC):[cH]:[c]:1[*]	0.492	2 out of 2

FCFP_12	-1320007763	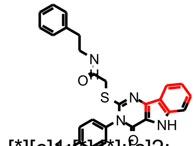 [*][c]1:[*]:[*]:[c]2: [*]:[cH]:[cH]:[cH]:[c]:1:2	0.475	10 out of 14
FCFP_12	-387072142	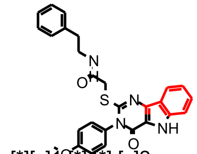 [*][c]1:[*]:[*]:[c]2: [cH]:[cH]:[cH]:[cH]: [c]:1:2	0.436	7 out of 10
**Top Features for negative contribution**				
**Fingerprint**	**Bit/Smiles**	**Feature Structure**	**Score**	**Carcinogen in training set**
FCFP_12	-925504834	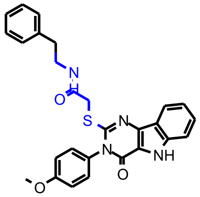 [*]CNC(=O)CS[*]	-0.982	0 out of 4
FCFP_12	566058135	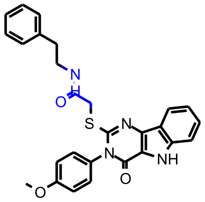 [*]CC(=O)N[*]	-0.705	3 out of 17
FCFP_12	-885550502	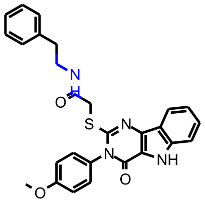 [*]CNC(=[*])[*]	-0.68	5 out of 26

Molecule

TOPKAT_Rat_Male_FDA_None_vs_Carcinogen

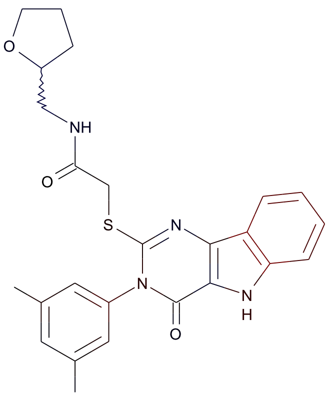

$C_{25}H_{26}N_4O_3S$
Molecular Weight: 462.56393
ALogP: 4.789
Rotatable Bonds: 6
Acceptors: 5
Donors: 2

Model Prediction

Prediction: Carcinogen
Probability: 0.391
Enrichment: 1.17
Bayesian Score: 1.16
Mahalanobis Distance: 20.4
Mahalanobis Distance p-value: 8.55e-020

Prediction: Positive if the Bayesian score is above the estimated best cutoff value from minimizing the false positive and false negative rate.
Probability: The esimated probability that the sample is in the positive category. This assumes that the Bayesian score follows a normal distribution and is different from the prediction using a cutoff.
Enrichment: An estimate of enrichment, that is, the increased likelihood (versus random) of this sample being in the category.
Bayesian Score: The standard Laplacian-modified Bayesian score.
Mahalanobis Distance: The Mahalanobis distance (MD) is the distance to the center of the training data. The larger the MD, the less trustworthy the prediction.
Mahalanobis Distance p-value: The p-value gives the fraction of training data with an MD greater than or equal to the one for the given sample, assuming normally distributed data. The smaller the p-value, the less trustworthy the prediciton. For highly non-normal X properties (e.g., fingerprints), the MD p-value is wildly inaccurate.

Structural Similar Compounds

Name	Bicalutamide	Glimepride	Glyburide
Structure	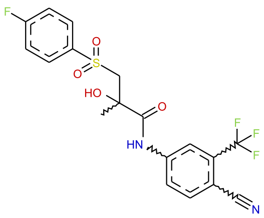	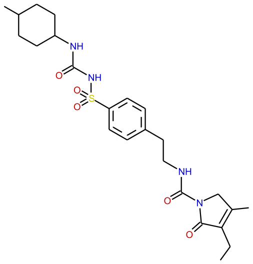	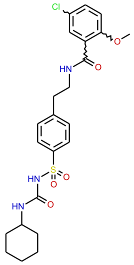
Actual Endpoint	Carcinogen	Non-Carcinogen	Non-Carcinogen
Predicted Endpoint	Carcinogen	Non-Carcinogen	Non-Carcinogen
Distance	0.645	0.649	0.657
Reference	US FDA (Centre for Drug Eval.& Res./Off. Testing & Res.) Sept. 1997	US FDA (Centre for Drug Eval.& Res./Off. Testing & Res.) Sept. 1997	US FDA (Centre for Drug Eval.& Res./Off. Testing & Res.) Sept. 1997

Model Applicability

Unknown features are fingerprint features in the query molecule, but not found or appearing too infrequently in the training set.

1. OPS PC17 out of range. Value: -4.5274. Training min, max, SD, explained variance: -3.8354, 4.9495, 1.344, 0.0168.
2. OPS PC24 out of range. Value: -4.0066. Training min, max, SD, explained variance: -3.2733, 3.1662, 1.103, 0.0113.

Feature Contribution

Top features for positive contribution

Fingerprint	Bit/Smiles	Feature Structure	Score	Carcinogen in training set
SCFP_6	1651620003	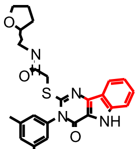 [*][c]1:[*]:[*]:[c]2:[cH]:[cH]:[cH]:[cH]:[c]:1:2	0.643	7 out of 10

SCFP_6	-1379673609	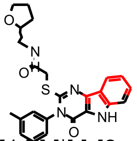<br>[*][c]1:[*]:[*]:[c]2:[*]:[cH]:[cH]:[cH]:[c]:1:2	0.526	11 out of 19
SCFP_6	1655199790	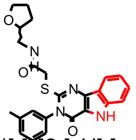<br>[*]1:[*]:[c]2:[cH]:[cH]:[cH]:[cH]:[c]:2:[nH]:1	0.52	5 out of 8

Top Features for negative contribution

Fingerprint	Bit/Smiles	Feature Structure	Score	Carcinogen in training set
SCFP_6	-37241568	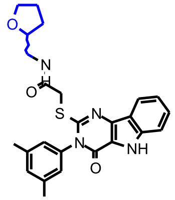<br>[*]CC1CCCO1	-0.674	0 out of 3
SCFP_6	-1325723550	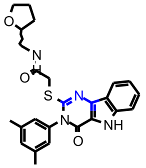<br>[*]C(=N[c](:[*]):[*])[*]	-0.664	1 out of 9
SCFP_6	-587569116	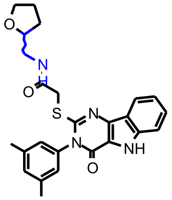<br>[*]NCC([*])[*]	-0.476	6 out of 32

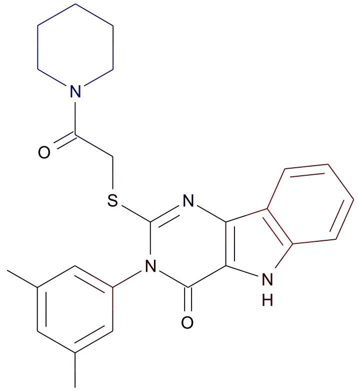

$C_{25}H_{26}N_4O_2S$
Molecular Weight: 446.56453
ALogP: 5.553
Rotatable Bonds: 4
Acceptors: 4
Donors: 1

Structural Similar Compounds

Name	Indomethacin	Ethynodiol	Mefloquine
Structure	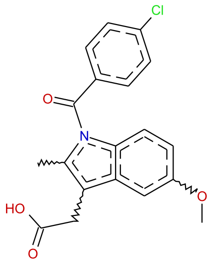	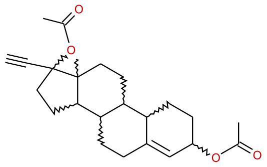	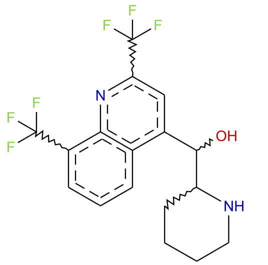
Actual Endpoint	Non-Carcinogen	Carcinogen	Non-Carcinogen
Predicted Endpoint	Non-Carcinogen	Carcinogen	Non-Carcinogen
Distance	0.651	0.691	0.704
Reference	US FDA (Centre for Drug Eval.& Res./Off. Testing & Res.) Sept. 1997	US FDA (Centre for Drug Eval.& Res./Off. Testing & Res.) Sept. 1997	US FDA (Centre for Drug Eval.& Res./Off. Testing & Res.) Sept. 1997

Model Prediction

Prediction: Carcinogen
Probability: 0.362
Enrichment: 1.08
Bayesian Score: 0.187
Mahalanobis Distance: 19.9
Mahalanobis Distance p-value: 1.76e-018

Prediction: Positive if the Bayesian score is above the estimated best cutoff value from minimizing the false positive and false negative rate.
Probability: The esimated probability that the sample is in the positive category. This assumes that the Bayesian score follows a normal distribution and is different from the prediction using a cutoff.
Enrichment: An estimate of enrichment, that is, the increased likelihood (versus random) of this sample being in the category.
Bayesian Score: The standard Laplacian-modified Bayesian score.
Mahalanobis Distance: The Mahalanobis distance (MD) is the distance to the center of the training data. The larger the MD, the less trustworthy the prediction.
Mahalanobis Distance p-value: The p-value gives the fraction of training data with an MD greater than or equal to the one for the given sample, assuming normally distributed data. The smaller the p-value, the less trustworthy the prediciton. For highly non-normal X properties (e.g., fingerprints), the MD p-value is wildly inaccurate.

Model Applicability

Unknown features are fingerprint features in the query molecule, but not found or appearing too infrequently in the training set.

1. OPS PC17 out of range. Value: -3.892. Training min, max, SD, explained variance: -3.8354, 4.9495, 1.344, 0.0168.

Feature Contribution

Top features for positive contribution				
**Fingerprint**	**Bit/Smiles**	**Feature Structure**	**Score**	**Carcinogen in training set**
SCFP_6	1651620003	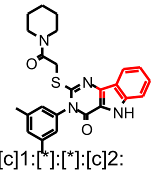 [*][c]1:[*]:[*]:[c]2:[cH]:[cH]:[cH]:[cH]:[c]:1:2	0.643	7 out of 10

SCFP_6	-1379673609	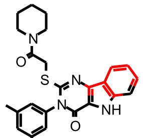 [*][c]1:[*]:[*]:[c]2:[*]:[cH]:[cH]:[cH]:[c]:1:2	0.526	11 out of 19
SCFP_6	1655199790	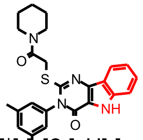 [*]1:[*]:[c]2:[cH]:[cH]:[cH]:[cH]:[c]:2:[nH]:1	0.52	5 out of 8

Top Features for negative contribution

Fingerprint	Bit/Smiles	Feature Structure	Score	Carcinogen in training set
SCFP_6	306578635	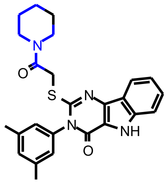 [*]C(=[*])N1C[*]CCC1	-0.825	0 out of 4
SCFP_6	1175638033	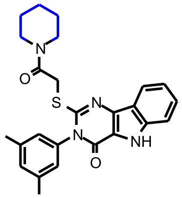 [*]1CCCCC1	-0.812	4 out of 32
SCFP_6	-1325723550	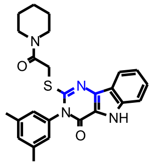 [*]C(=N[c](:[*]):[*])[*]	-0.664	1 out of 9

#UNDEFINED

TOPKAT_Rat_Male_FDA_None_vs_Carcinogen

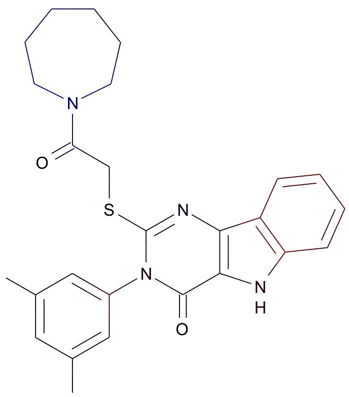

$C_{26}H_{28}N_4O_2S$

Molecular Weight: 460.59111

ALogP: 6.009

Rotatable Bonds: 4

Acceptors: 4

Donors: 1

Structural Similar Compounds

Name	Indomethacin	Ethynodiol	Loperamide
Structure	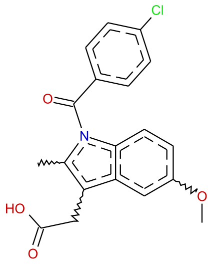	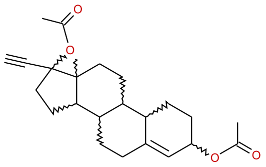	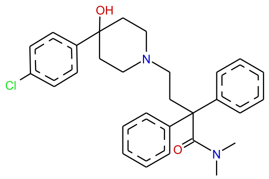
Actual Endpoint	Non-Carcinogen	Carcinogen	Non-Carcinogen
Predicted Endpoint	Non-Carcinogen	Carcinogen	Non-Carcinogen
Distance	0.697	0.715	0.730
Reference	US FDA (Centre for Drug Eval.& Res./Off. Testing & Res.) Sept. 1997	US FDA (Centre for Drug Eval.& Res./Off. Testing & Res.) Sept. 1997	US FDA (Centre for Drug Eval.& Res./Off. Testing & Res.) Sept. 1997

Model Prediction

Prediction: Non-Carcinogen

Probability: 0.348

Enrichment: 1.04

Bayesian Score: -0.295

Mahalanobis Distance: 19.9

Mahalanobis Distance p-value: 1.68e-018

Prediction: Positive if the Bayesian score is above the estimated best cutoff value from minimizing the false positive and false negative rate.
Probability: The esimated probability that the sample is in the positive category. This assumes that the Bayesian score follows a normal distribution and is different from the prediction using a cutoff.
Enrichment: An estimate of enrichment, that is, the increased likelihood (versus random) of this sample being in the category.
Bayesian Score: The standard Laplacian-modified Bayesian score.
Mahalanobis Distance: The Mahalanobis distance (MD) is the distance to the center of the training data. The larger the MD, the less trustworthy the prediction.
Mahalanobis Distance p-value: The p-value gives the fraction of training data with an MD greater than or equal to the one for the given sample, assuming normally distributed data. The smaller the p-value, the less trustworthy the prediciton. For highly non-normal X properties (e.g., fingerprints), the MD p-value is wildly inaccurate.

Model Applicability

Unknown features are fingerprint features in the query molecule, but not found or appearing too infrequently in the training set.

1. OPS PC17 out of range. Value: -3.9411. Training min, max, SD, explained variance: -3.8354, 4.9495, 1.344, 0.0168.

Feature Contribution

Top features for positive contribution

Fingerprint	Bit/Smiles	Feature Structure	Score	Carcinogen in training set
SCFP_6	1651620003	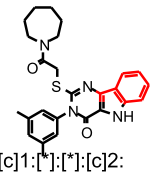 [*][c]1:[*]:[*]:[c]2:[cH]:[cH]:[cH]:[cH]:[c]:1:2	0.643	7 out of 10

SCFP_6	-1379673609	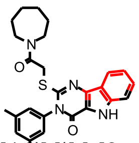 [*][c]1:[*]:[*]:[c]2:[*]:[cH]:[cH]:[cH]:[c]:1:2	0.526	11 out of 19
SCFP_6	1655199790	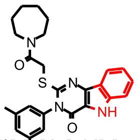 [*]1:[*]:[c]2:[cH]:[cH]:[cH]:[cH]:[c]:2:[nH]:1	0.52	5 out of 8

Top Features for negative contribution

Fingerprint	Bit/Smiles	Feature Structure	Score	Carcinogen in training set
SCFP_6	306578635	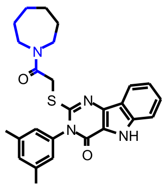 [*]C(=[*])N1C[*]CCC1	-0.825	0 out of 4
SCFP_6	1175638033	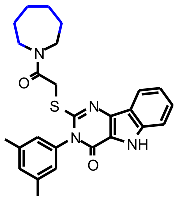 [*]1CCCCC1	-0.812	4 out of 32
SCFP_6	-1325723550	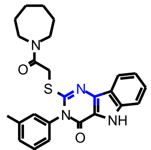 [*]C(=N[c](:[*]):[*])[*]	-0.664	1 out of 9

#UNDEFINED

TOPKAT_Rat_Male_FDA_None_vs_Carcinogen

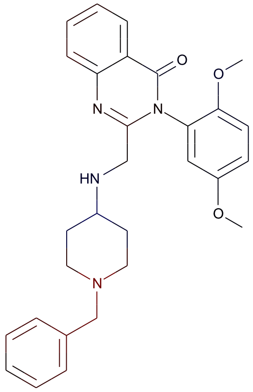

$C_{29}H_{32}N_4O_3$

Molecular Weight: 484.58938

ALogP: 3.743

Rotatable Bonds: 8

Acceptors: 6

Donors: 1

Model Prediction

Prediction: Non-Carcinogen

Probability: 0.295

Enrichment: 0.883

Bayesian Score: -2.32

Mahalanobis Distance: 18.5

Mahalanobis Distance p-value: 8e-015

Prediction: Positive if the Bayesian score is above the estimated best cutoff value from minimizing the false positive and false negative rate.
Probability: The esimated probability that the sample is in the positive category. This assumes that the Bayesian score follows a normal distribution and is different from the prediction using a cutoff.
Enrichment: An estimate of enrichment, that is, the increased likelihood (versus random) of this sample being in the category.
Bayesian Score: The standard Laplacian-modified Bayesian score.
Mahalanobis Distance: The Mahalanobis distance (MD) is the distance to the center of the training data. The larger the MD, the less trustworthy the prediction.
Mahalanobis Distance p-value: The p-value gives the fraction of training data with an MD greater than or equal to the one for the given sample, assuming normally distributed data. The smaller the p-value, the less trustworthy the prediciton. For highly non-normal X properties (e.g., fingerprints), the MD p-value is wildly inaccurate.

Structural Similar Compounds

Name	Emetine	Felodipine	Cisapride
Structure	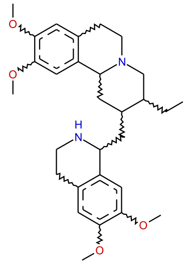	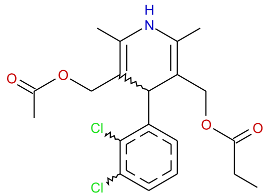	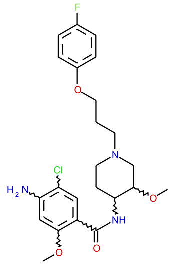
Actual Endpoint	Non-Carcinogen	Carcinogen	Non-Carcinogen
Predicted Endpoint	Non-Carcinogen	Carcinogen	Non-Carcinogen
Distance	0.574	0.611	0.611
Reference	US FDA (Centre for Drug Eval.& Res./Off. Testing & Res.) Sept. 1997	US FDA (Centre for Drug Eval.& Res./Off. Testing & Res.) Sept. 1997	US FDA (Centre for Drug Eval.& Res./Off. Testing & Res.) Sept. 1997

Model Applicability

Unknown features are fingerprint features in the query molecule, but not found or appearing too infrequently in the training set.

1. All properties and OPS components are within expected ranges.

Feature Contribution

Top features for positive contribution				
**Fingerprint**	**Bit/Smiles**	**Feature Structure**	**Score**	**Carcinogen in training set**
SCFP_6	2088734719	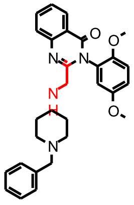 [*]NCC(=[*])[*]	0.722	7 out of 9

SCFP_6	-205766035	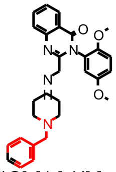 [*]N([*])C[c]1:[cH]:[cH]:[*]:[cH]:[cH]:1	0.712	3 out of 3
SCFP_6	1274421524	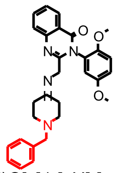 [*]N([*])C[c]1:[cH]:[cH]:[cH]:[cH]:[cH]:1	0.603	2 out of 2

Top Features for negative contribution

Fingerprint	Bit/Smiles	Feature Structure	Score	Carcinogen in training set
SCFP_6	-1430588017	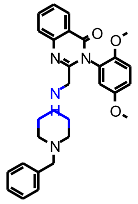 [*]CC(C[*])N[*]	-0.787	5 out of 38
SCFP_6	-627385064	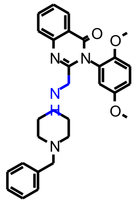 [*]CNC([*])[*]	-0.717	7 out of 48
SCFP_6	-1325723550	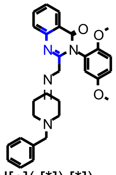 [*]C(=N[c](:[*]):[*])[*]	-0.664	1 out of 9

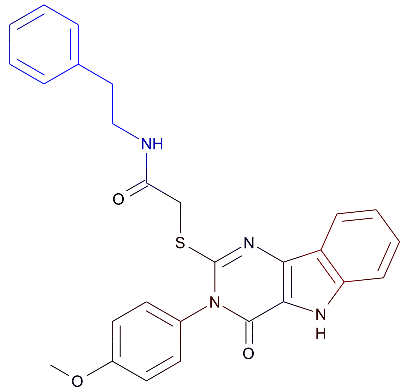

$C_{27}H_{24}N_4O_3S$
Molecular Weight: 484.56946
ALogP: 5.346
Rotatable Bonds: 8
Acceptors: 5
Donors: 2

Structural Similar Compounds

Name	Glyburide	Bitolterol	Glimepride
Structure	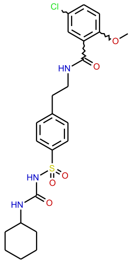	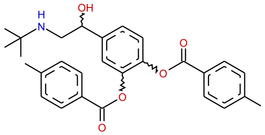	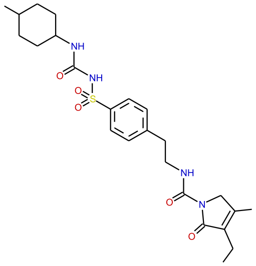
Actual Endpoint	Non-Carcinogen	Non-Carcinogen	Non-Carcinogen
Predicted Endpoint	Non-Carcinogen	Non-Carcinogen	Non-Carcinogen
Distance	0.590	0.633	0.653
Reference	US FDA (Centre for Drug Eval.& Res./Off. Testing & Res.) Sept. 1997	US FDA (Centre for Drug Eval.& Res./Off. Testing & Res.) Sept. 1997	US FDA (Centre for Drug Eval.& Res./Off. Testing & Res.) Sept. 1997

Model Prediction

Prediction: Non-Carcinogen
Probability: 0.239
Enrichment: 0.715
Bayesian Score: -4.97
Mahalanobis Distance: 16.2
Mahalanobis Distance p-value: 1.93e-009

Prediction: Positive if the Bayesian score is above the estimated best cutoff value from minimizing the false positive and false negative rate.
Probability: The esimated probability that the sample is in the positive category. This assumes that the Bayesian score follows a normal distribution and is different from the prediction using a cutoff.
Enrichment: An estimate of enrichment, that is, the increased likelihood (versus random) of this sample being in the category.
Bayesian Score: The standard Laplacian-modified Bayesian score.
Mahalanobis Distance: The Mahalanobis distance (MD) is the distance to the center of the training data. The larger the MD, the less trustworthy the prediction.
Mahalanobis Distance p-value: The p-value gives the fraction of training data with an MD greater than or equal to the one for the given sample, assuming normally distributed data. The smaller the p-value, the less trustworthy the prediciton. For highly non-normal X properties (e.g., fingerprints), the MD p-value is wildly inaccurate.

Model Applicability

Unknown features are fingerprint features in the query molecule, but not found or appearing too infrequently in the training set.

1. OPS PC29 out of range. Value: -3.5189. Training min, max, SD, explained variance: -3.1746, 3.7825, 1.007, 0.0095.

Feature Contribution

Top features for positive contribution				
Fingerprint	Bit/Smiles	Feature Structure	Score	Carcinogen in training set
SCFP_6	1651620003	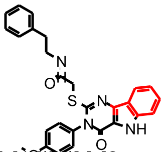 [*][c]1c[*]:[*]:[c]2:[cH]:[cH]:[cH]:[cH]:[c]:1:2	0.643	7 out of 10

SCFP_6	-1379673609	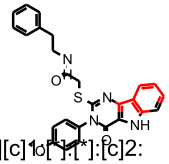 [*][c]1:[*]:[*]:[c]2:[*]:[cH]:[cH]:[cH]:[c]:1:2	0.526	11 out of 19
SCFP_6	1655199790	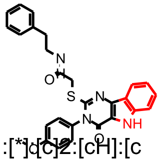 [*]1:[*]:[c]2:[cH]:[cH]:[cH]:[cH]:[c]:2:[nH]:1	0.52	5 out of 8
**Top Features for negative contribution**				
**Fingerprint**	**Bit/Smiles**	**Feature Structure**	**Score**	**Carcinogen in training set**
SCFP_6	-1211866396	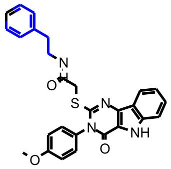 [*]CC[c]1:[cH]:[cH]:[*]:[cH]:[cH]:1	-1.1	2 out of 25
SCFP_6	-1849236245	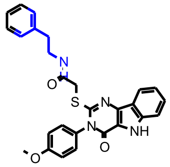 [*]NCC[c](:[cH]:[*]):[cH]:[*]	-0.825	0 out of 4
SCFP_6	-1640858361	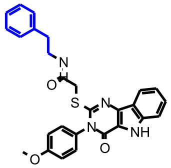 [*]CC[c]1:[cH]:[cH]:[cH]:[cH]:[cH]:1	-0.817	1 out of 11

Molecule

TOPKAT_Rat_Male_FDA_Single_vs_Multiple

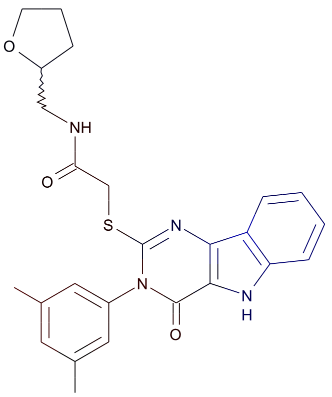

$C_{25}H_{26}N_4O_3S$
Molecular Weight: 462.56393
ALogP: 4.789
Rotatable Bonds: 6
Acceptors: 5
Donors: 2

Structural Similar Compounds

Name	Bicalutamide	Simvastatin	Fluvastatin
Structure	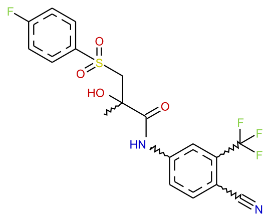	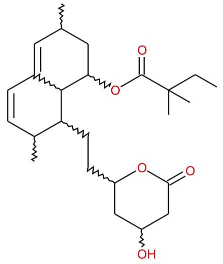	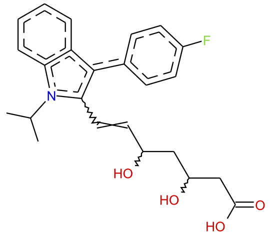
Actual Endpoint	Multiple-Carcinogen	Multiple-Carcinogen	Single-Carcinogen
Predicted Endpoint	Multiple-Carcinogen	Multiple-Carcinogen	Single-Carcinogen
Distance	0.667	0.710	0.718
Reference	US FDA (Centre for Drug Eval.& Res./Off. Testing & Res.) Sept. 1997	US FDA (Centre for Drug Eval.& Res./Off. Testing & Res.) Sept. 1997	US FDA (Centre for Drug Eval.& Res./Off. Testing & Res.) Sept. 1997

Model Prediction

Prediction: Single-Carcinogen
Probability: 0.559
Enrichment: 1.35
Bayesian Score: -0.497
Mahalanobis Distance: 22.6
Mahalanobis Distance p-value: 1.63e-012

Prediction: Positive if the Bayesian score is above the estimated best cutoff value from minimizing the false positive and false negative rate.
Probability: The esimated probability that the sample is in the positive category. This assumes that the Bayesian score follows a normal distribution and is different from the prediction using a cutoff.
Enrichment: An estimate of enrichment, that is, the increased likelihood (versus random) of this sample being in the category.
Bayesian Score: The standard Laplacian-modified Bayesian score.
Mahalanobis Distance: The Mahalanobis distance (MD) is the distance to the center of the training data. The larger the MD, the less trustworthy the prediction.
Mahalanobis Distance p-value: The p-value gives the fraction of training data with an MD greater than or equal to the one for the given sample, assuming normally distributed data. The smaller the p-value, the less trustworthy the prediciton. For highly non-normal X properties (e.g., fingerprints), the MD p-value is wildly inaccurate.

Model Applicability

Unknown features are fingerprint features in the query molecule, but not found or appearing too infrequently in the training set.

1. OPS PC9 out of range. Value: -4.7894. Training min, max, SD, explained variance: -2.9055, 6.042, 1.612, 0.0353.

Feature Contribution

Top features for positive contribution

Fingerprint	Bit/Smiles	Feature Structure	Score	Multiple-Carcinogen in training set
SCFP_8	-347281112	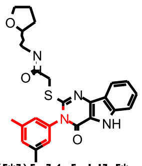 [*]N([*])[c]1:[cH]:[*]:[cH]:[c](C):[cH]:1	0.553	2 out of 2

SCFP_8	1257084377	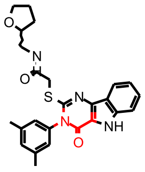 [*]N([*])C(=O)[c](:[*]):[*]	0.489	3 out of 4
SCFP_8	921637355	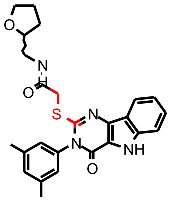 [*]CSC(=[*])[*]	0.383	1 out of 1

Top Features for negative contribution

Fingerprint	Bit/Smiles	Feature Structure	Score	Multiple-Carcinogen in training set
SCFP_8	1851000357	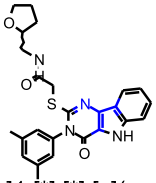 [*][c]1:[*]:[*]:[c](:[*]):[c]:1N=[*]	-0.737	0 out of 3
SCFP_8	-1381862798	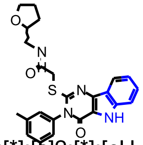 [*]1:[*]:[c]2:[*]:[cH]:[cH]:[cH]:[c]:2:[n H]:1	-0.572	1 out of 7
SCFP_8	1655199790	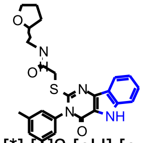 [*]1:[*]:[c]2:[cH]:[c H]:[cH]:[cH]:[c]:2:[nH]:1	-0.342	1 out of 5

#UNDEFINED

TOPKAT_Rat_Male_FDA_Single_vs_Multiple

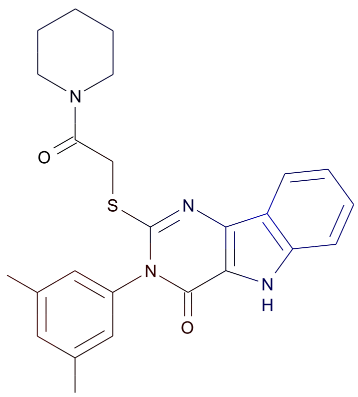

$C_{25}H_{26}N_4O_2S$

Molecular Weight: 446.56453

ALogP: 5.553

Rotatable Bonds: 4

Acceptors: 4

Donors: 1

Model Prediction

Prediction: Single-Carcinogen

Probability: 0.55

Enrichment: 1.33

Bayesian Score: -1.18

Mahalanobis Distance: 19.9

Mahalanobis Distance p-value: 3.67e-010

Prediction: Positive if the Bayesian score is above the estimated best cutoff value from minimizing the false positive and false negative rate.
Probability: The esimated probability that the sample is in the positive category. This assumes that the Bayesian score follows a normal distribution and is different from the prediction using a cutoff.
Enrichment: An estimate of enrichment, that is, the increased likelihood (versus random) of this sample being in the category.
Bayesian Score: The standard Laplacian-modified Bayesian score.
Mahalanobis Distance: The Mahalanobis distance (MD) is the distance to the center of the training data. The larger the MD, the less trustworthy the prediction.
Mahalanobis Distance p-value: The p-value gives the fraction of training data with an MD greater than or equal to the one for the given sample, assuming normally distributed data. The smaller the p-value, the less trustworthy the prediciton. For highly non-normal X properties (e.g., fingerprints), the MD p-value is wildly inaccurate.

Structural Similar Compounds

Name	Ethynodiol	Simvastatin	Lansoprazole
Structure	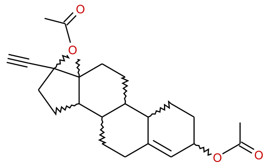	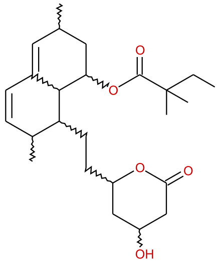	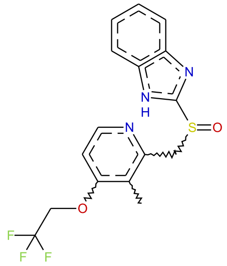
Actual Endpoint	Single-Carcinogen	Multiple-Carcinogen	Multiple-Carcinogen
Predicted Endpoint	Single-Carcinogen	Multiple-Carcinogen	Multiple-Carcinogen
Distance	0.727	0.734	0.750
Reference	US FDA (Centre for Drug Eval.& Res./Off. Testing & Res.) Sept. 1997	US FDA (Centre for Drug Eval.& Res./Off. Testing & Res.) Sept. 1997	US FDA (Centre for Drug Eval.& Res./Off. Testing & Res.) Sept. 1997

Model Applicability

Unknown features are fingerprint features in the query molecule, but not found or appearing too infrequently in the training set.

1. OPS PC8 out of range. Value: 5.4334. Training min, max, SD, explained variance: -4.9055, 5.0367, 1.697, 0.0391.
2. OPS PC9 out of range. Value: -3.6772. Training min, max, SD, explained variance: -2.9055, 6.042, 1.612, 0.0353.

Feature Contribution

Top features for positive contribution

Fingerprint	Bit/Smiles	Feature Structure	Score	Multiple-Carcinogen in training set
SCFP_8	-347281112	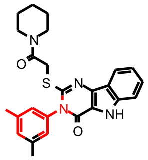 [*]N([*])[c]1:[cH]:[*]:[cH]:[c](C):[cH]:1	0.553	2 out of 2

SCFP_8	1257084377	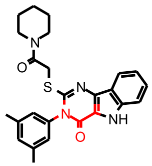 [*]N([*])C(=O)[c](:[*]):[*]	0.489	3 out of 4
SCFP_8	921637355	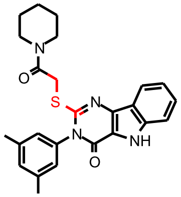 [*]CSC(=[*])[*]	0.383	1 out of 1
**Top Features for negative contribution**				
**Fingerprint**	**Bit/Smiles**	**Feature Structure**	**Score**	**Multiple-Carcinogen in training set**
SCFP_8	1851000357	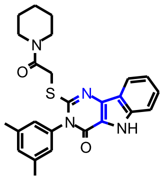 [*][c]1:[*]:[*]:[c](:[*]):[c]:1N=[*]	-0.737	0 out of 3
SCFP_8	-1381862798	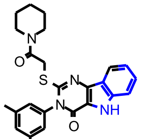 [*]1:[*]:[c]2:[*]:[cH]:[cH]:[cH]:[c]:2:[nH]:1	-0.572	1 out of 7
SCFP_8	1655199790	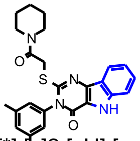 [*]1:[*]:[c]2:[cH]:[cH]:[cH]:[cH]:[c]:2:[nH]:1	-0.342	1 out of 5

Molecule

TOPKAT_Rat_Male_NTP

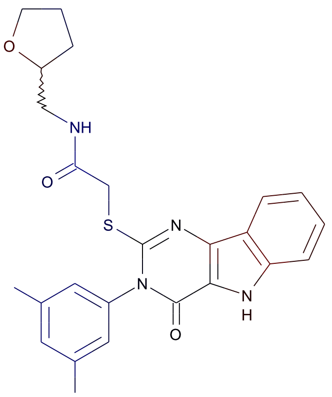

$C_{25}H_{26}N_4O_3S$
Molecular Weight: 462.56393
ALogP: 4.789
Rotatable Bonds: 6
Acceptors: 5
Donors: 2

Structural Similar Compounds

Name	Rhodamine 6G	Curcumin	Ochratoxin A
Structure	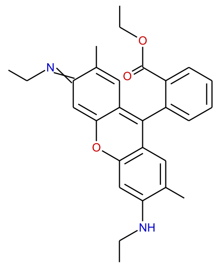	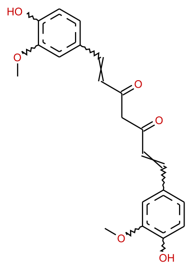	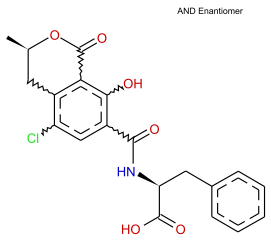
Actual Endpoint	Carcinogen	Non-Carcinogen	Carcinogen
Predicted Endpoint	Carcinogen	Non-Carcinogen	Carcinogen
Distance	0.687	0.701	0.703
Reference	NTP364	NTP427	NTP358 & CPDB

Model Prediction

Prediction: Non-Carcinogen
Probability: 0.412
Enrichment: 0.81
Bayesian Score: -4.34
Mahalanobis Distance: 9.93
Mahalanobis Distance p-value: 0.00126

Prediction: Positive if the Bayesian score is above the estimated best cutoff value from minimizing the false positive and false negative rate.
Probability: The esimated probability that the sample is in the positive category. This assumes that the Bayesian score follows a normal distribution and is different from the prediction using a cutoff.
Enrichment: An estimate of enrichment, that is, the increased likelihood (versus random) of this sample being in the category.
Bayesian Score: The standard Laplacian-modified Bayesian score.
Mahalanobis Distance: The Mahalanobis distance (MD) is the distance to the center of the training data. The larger the MD, the less trustworthy the prediction.
Mahalanobis Distance p-value: The p-value gives the fraction of training data with an MD greater than or equal to the one for the given sample, assuming normally distributed data. The smaller the p-value, the less trustworthy the prediciton. For highly non-normal X properties (e.g., fingerprints), the MD p-value is wildly inaccurate.

Model Applicability

Unknown features are fingerprint features in the query molecule, but not found or appearing too infrequently in the training set.

1. All properties and OPS components are within expected ranges.
2. Unknown ECFP_2 feature: -782828288: [*]C(=[*])[c]1:[nH]:[*]:[*]:[c]:1[*]
3. Unknown ECFP_2 feature: -962771238: [*]C(=[*])N(C(=[*])[*])[c](:[*]):[*]
4. Unknown ECFP_2 feature: -962137479: [*][c]1:[*]:[*]:[c](:[*]):[c]:1N=[*]
5. Unknown ECFP_2 feature: 676970202: [*]S\C(=N\[*])\N([*])[*]
6. Unknown ECFP_2 feature: 2085698692: [*]C(=N[c](:[*]):[*])[*]
7. Unknown ECFP_2 feature: 1427820655: [*]CSC(=[*])[*]
8. Unknown ECFP_2 feature: -84772164: [*]NCC([*])[*]

Feature Contribution

Top features for positive contribution				
Fingerprint	Bit/Smiles	Feature Structure	Score	Carcinogen in training set

ECFP_12	-554480104	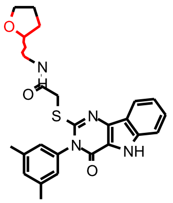 [*]CC1C[*][*]O1	0.511	4 out of 4
ECFP_12	1639858918	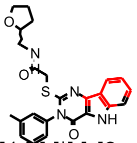 [*][c]1:[*]:[*]:[c]2:[*]:[cH]:[cH]:[cH]:[c]:1:2	0.47	7 out of 8
ECFP_12	-1939757055	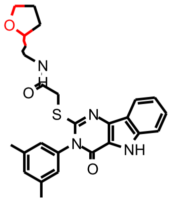 [*]C1[*][*]CO1	0.442	6 out of 7

Top Features for negative contribution

Fingerprint	Bit/Smiles	Feature Structure	Score	Carcinogen in training set
ECFP_12	1731843802	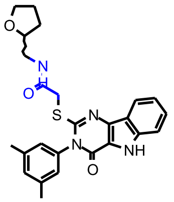 [*]CC(=O)N[*]	-1.1	0 out of 4
ECFP_12	-830332112	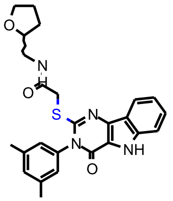 [*]S[*]	-1.01	1 out of 9

ECFP_12	-1332781180	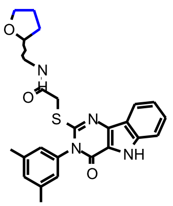 [*]1[*]CCC1	-1.01	1 out of 9

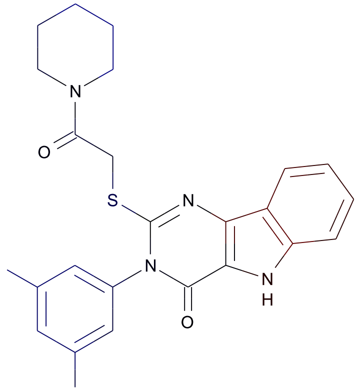

$C_{25}H_{26}N_4O_2S$
Molecular Weight: 446.56453
ALogP: 5.553
Rotatable Bonds: 4
Acceptors: 4
Donors: 1

Structural Similar Compounds

Name	Rhodamine 6G	Lithocholic Acid	C.I. Pigment Red 3
Structure	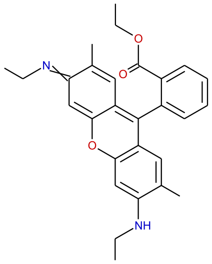	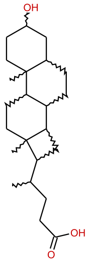	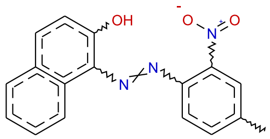
Actual Endpoint	Carcinogen	Non-Carcinogen	Carcinogen
Predicted Endpoint	Carcinogen	Non-Carcinogen	Carcinogen
Distance	0.682	0.708	0.710
Reference	NTP364	NTP/TR-175	NTP/TR-407

Model Prediction

Prediction: Non-Carcinogen
Probability: 0.374
Enrichment: 0.735
Bayesian Score: -5.02
Mahalanobis Distance: 10.5
Mahalanobis Distance p-value: 0.000123

Prediction: Positive if the Bayesian score is above the estimated best cutoff value from minimizing the false positive and false negative rate.
Probability: The esimated probability that the sample is in the positive category. This assumes that the Bayesian score follows a normal distribution and is different from the prediction using a cutoff.
Enrichment: An estimate of enrichment, that is, the increased likelihood (versus random) of this sample being in the category.
Bayesian Score: The standard Laplacian-modified Bayesian score.
Mahalanobis Distance: The Mahalanobis distance (MD) is the distance to the center of the training data. The larger the MD, the less trustworthy the prediction.
Mahalanobis Distance p-value: The p-value gives the fraction of training data with an MD greater than or equal to the one for the given sample, assuming normally distributed data. The smaller the p-value, the less trustworthy the prediciton. For highly non-normal X properties (e.g., fingerprints), the MD p-value is wildly inaccurate.

Model Applicability

Unknown features are fingerprint features in the query molecule, but not found or appearing too infrequently in the training set.

1. All properties and OPS components are within expected ranges.
2. Unknown ECFP_2 feature: -782828288: [*]C(=[*])[c]1:[nH]:[*]:[*]:[c]:1[*]
3. Unknown ECFP_2 feature: -962771238: [*]C(=[*])N(C(=[*])[*])[c](:[*]):[*]
4. Unknown ECFP_2 feature: -962137479: [*][c]1:[*]:[*]:[c](:[*]):[c]:1N=[*]
5. Unknown ECFP_2 feature: 676970202: [*]S\C(=N\[*])\N([*])[*]
6. Unknown ECFP_2 feature: 2085698692: [*]C(=N[c](:[*]):[*])[*]
7. Unknown ECFP_2 feature: 1427820655: [*]CSC(=[*])[*]
8. Unknown ECFP_2 feature: 1341750291: [*]CC(=O)N([*])[*]
9. Unknown ECFP_2 feature: -1102925512: [*]CN(C[*])C(=[*])[*]

Feature Contribution

Top features for positive contribution				
Fingerprint	Bit/Smiles	Feature Structure	Score	Carcinogen in training set

ECFP_12	1639858918	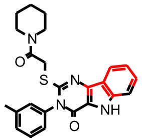 [*][c]1:[*]:[*]:[c]2:[*]:[cH]:[cH]:[cH]:[c]:1:2	0.47	7 out of 8
ECFP_12	1306977740	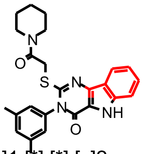 [*][c]1:[*]:[*]:[c]2:[cH]:[cH]:[cH]:[cH]:[c]:1:2	0.442	6 out of 7
ECFP_12	1333660716	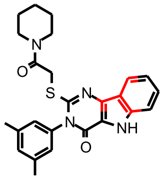 [*][c]1:[*]:[*]:[c](:[*]):[c]:1:[cH]:[*]	0.405	8 out of 10

Top Features for negative contribution

Fingerprint	Bit/Smiles	Feature Structure	Score	Carcinogen in training set
ECFP_12	662850656	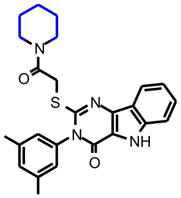 [*]1CCCCC1	-1.1	0 out of 4
ECFP_12	-830332112	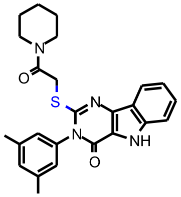 [*]S[*]	-1.01	1 out of 9

ECFP_12	-1332781180	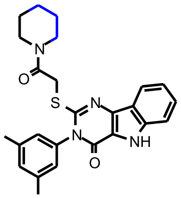 [*]1[*]CCC1	-1.01	1 out of 9

#UNDEFINED

TOPKAT_Rat_Male_NTP

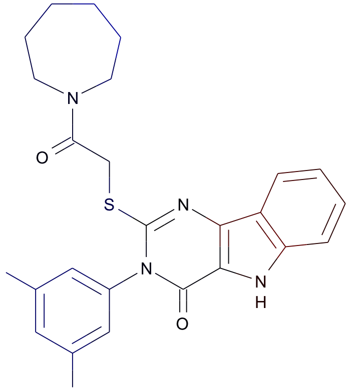

$C_{26}H_{28}N_4O_2S$

Molecular Weight: 460.59111

ALogP: 6.009

Rotatable Bonds: 4

Acceptors: 4

Donors: 1

Model Prediction

Prediction: Non-Carcinogen

Probability: 0.351

Enrichment: 0.689

Bayesian Score: -5.43

Mahalanobis Distance: 10.6

Mahalanobis Distance p-value: 8.73e-005

Prediction: Positive if the Bayesian score is above the estimated best cutoff value from minimizing the false positive and false negative rate.
Probability: The esimated probability that the sample is in the positive category. This assumes that the Bayesian score follows a normal distribution and is different from the prediction using a cutoff.
Enrichment: An estimate of enrichment, that is, the increased likelihood (versus random) of this sample being in the category.
Bayesian Score: The standard Laplacian-modified Bayesian score.
Mahalanobis Distance: The Mahalanobis distance (MD) is the distance to the center of the training data. The larger the MD, the less trustworthy the prediction.
Mahalanobis Distance p-value: The p-value gives the fraction of training data with an MD greater than or equal to the one for the given sample, assuming normally distributed data. The smaller the p-value, the less trustworthy the prediciton. For highly non-normal X properties (e.g., fingerprints), the MD p-value is wildly inaccurate.

Structural Similar Compounds

Name	Rhodamine 6G	Tricresyl Phosphate	4;4'-Thiobis-(6-tert-butyl-m-cresol)
Structure	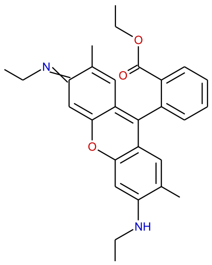	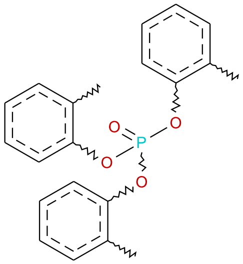	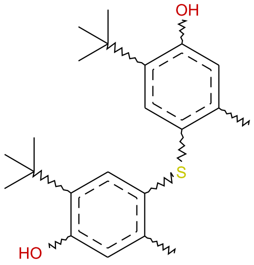
Actual Endpoint	Carcinogen	Non-Carcinogen	Non-Carcinogen
Predicted Endpoint	Carcinogen	Non-Carcinogen	Non-Carcinogen
Distance	0.685	0.710	0.731
Reference	NTP364	NTP/TR-433	NTP/TR-435

Model Applicability

Unknown features are fingerprint features in the query molecule, but not found or appearing too infrequently in the training set.

1. All properties and OPS components are within expected ranges.
2. Unknown ECFP_2 feature: -782828288: [*]C(=[*])[c]1:[nH]:[*]:[*]:[c]:1[*]
3. Unknown ECFP_2 feature: -962771238: [*]C(=[*])N(C(=[*])[*])[c](:[*]):[*]
4. Unknown ECFP_2 feature: -962137479: [*][c]1:[*]:[*]:[c](:[*]):[c]:1N=[*]
5. Unknown ECFP_2 feature: 676970202: [*]S\C(=N\[*])\N([*])[*]
6. Unknown ECFP_2 feature: 2085698692: [*]C(=N[c](:[*]):[*])[*]
7. Unknown ECFP_2 feature: 1427820655: [*]CSC(=[*])[*]
8. Unknown ECFP_2 feature: 1341750291: [*]CC(=O)N([*])[*]
9. Unknown ECFP_2 feature: -1102925512: [*]CN(C[*])C(=[*])[*]

Feature Contribution

Top features for positive contribution

Fingerprint	Bit/Smiles	Feature Structure	Score	Carcinogen in training set

ECFP_12	1639858918	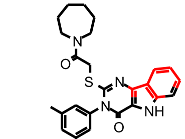 [*][c]1:[*]:[*]:[c]2:[*]:[cH]:[cH]:[cH]:[c]:1:2	0.47	7 out of 8
ECFP_12	1306977740	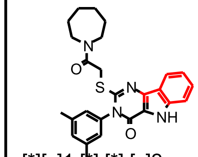 [*][c]1:[*]:[*]:[c]2:[cH]:[cH]:[cH]:[cH]:[c]:1:2	0.442	6 out of 7
ECFP_12	1333660716	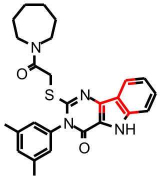 [*][c]1:[*]:[*]:[c](:[*]):[c]:1:[cH]:[*]	0.405	8 out of 10

Top Features for negative contribution

**Fingerprint**	**Bit/Smiles**	**Feature Structure**	**Score**	**Carcinogen in training set**
ECFP_12	662850656	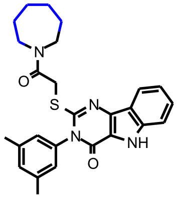 [*]1CCCCC1	-1.1	0 out of 4
ECFP_12	-1332781180	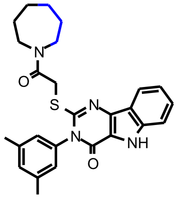 [*]1[*]CCC1	-1.01	1 out of 9

ECFP_12	-830332112	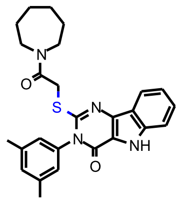<br>[*]S[*]	-1.01	1 out of 9

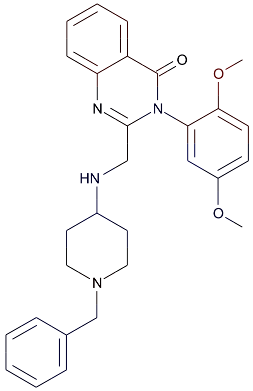

$C_{29}H_{32}N_4O_3$
Molecular Weight: 484.58938
ALogP: 3.743
Rotatable Bonds: 8
Acceptors: 6
Donors: 1

Structural Similar Compounds

Name	Rhodamine 6G	Curcumin	Butyl Benzyl Phthalate
Structure	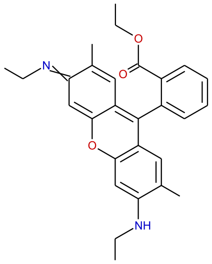	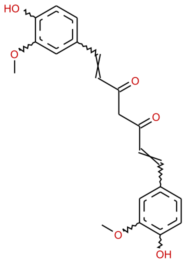	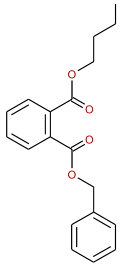
Actual Endpoint	Carcinogen	Non-Carcinogen	Carcinogen
Predicted Endpoint	Carcinogen	Non-Carcinogen	Carcinogen
Distance	0.673	0.718	0.795
Reference	NTP364	NTP427	NTP458 & CPDB

Model Applicability

Unknown features are fingerprint features in the query molecule, but not found or appearing too infrequently in the training set.

1. OPS PC4 out of range. Value: 7.7916. Training min, max, SD, explained variance: -4.722, 6.6535, 2.046, 0.0759.
2. Unknown ECFP_2 feature: -962771238: [*]C(=[*])N(C(=[*])[*])[c](:[*]):[*]
3. Unknown ECFP_2 feature: -1073177635: [*]C\C(=N\[*])\N([*])[*]
4. Unknown ECFP_2 feature: 2085698692: [*]C(=N[c](:[*]):[*])[*]
5. Unknown ECFP_2 feature: -597295171: [*][c](:[*]):[c](:[cH]:[*])N=[*]
6. Unknown ECFP_2 feature: -2041399277: [*]CN(C[*])C[*]
7. Unknown ECFP_2 feature: 496787418: [*]CNC([*])[*]
8. Unknown ECFP_2 feature: -44121127: [*]N([*])C[c](:[*]):[*]

Model Prediction

Prediction: Non-Carcinogen
Probability: 0.578
Enrichment: 1.14
Bayesian Score: -0.987
Mahalanobis Distance: 10.5
Mahalanobis Distance p-value: 0.00016

Prediction: Positive if the Bayesian score is above the estimated best cutoff value from minimizing the false positive and false negative rate.
Probability: The esimated probability that the sample is in the positive category. This assumes that the Bayesian score follows a normal distribution and is different from the prediction using a cutoff.
Enrichment: An estimate of enrichment, that is, the increased likelihood (versus random) of this sample being in the category.
Bayesian Score: The standard Laplacian-modified Bayesian score.
Mahalanobis Distance: The Mahalanobis distance (MD) is the distance to the center of the training data. The larger the MD, the less trustworthy the prediction.
Mahalanobis Distance p-value: The p-value gives the fraction of training data with an MD greater than or equal to the one for the given sample, assuming normally distributed data. The smaller the p-value, the less trustworthy the prediciton. For highly non-normal X properties (e.g., fingerprints), the MD p-value is wildly inaccurate.

Feature Contribution

Top features for positive contribution				
Fingerprint	Bit/Smiles	Feature Structure	Score	Carcinogen in training set

ECFP_12	2077607946	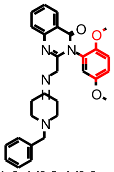 [*]O[c]1:[cH]:[cH]:[c]([*]):[*]:[c]:1[*]	0.405	5 out of 6
ECFP_12	1680623188	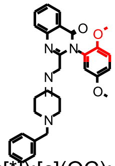 [*][c](:[*]):[c](OC):[cH]:[*]	0.405	14 out of 18
ECFP_12	-427397688	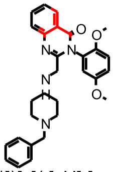 [*]C(=[*])[c](:[cH]:[*]):[c]([*]):[*]	0.367	12 out of 16
**Top Features for negative contribution**				
**Fingerprint**	**Bit/Smiles**	**Feature Structure**	**Score**	**Carcinogen in training set**
ECFP_12	-859078569	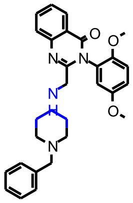 [*]CC(C[*])N[*]	-0.693	0 out of 2
ECFP_12	-1897341097	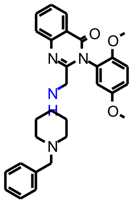 [*]N[*]	-0.429	13 out of 41

ECFP_12	-176455838	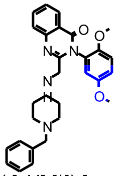 [*]O[c](:[cH]:[*]):[cH]:[*]	-0.406	3 out of 10

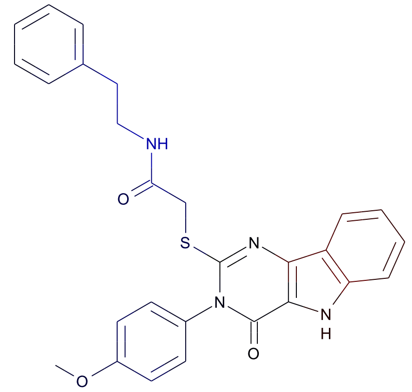

$C_{27}H_{24}N_4O_3S$

Molecular Weight: 484.56946

ALogP: 5.346

Rotatable Bonds: 8

Acceptors: 5

Donors: 2

Model Prediction

Prediction: Non-Carcinogen

Probability: 0.272

Enrichment: 0.535

Bayesian Score: -6.84

Mahalanobis Distance: 13.6

Mahalanobis Distance p-value: 1.78e-011

Prediction: Positive if the Bayesian score is above the estimated best cutoff value from minimizing the false positive and false negative rate.
Probability: The esimated probability that the sample is in the positive category. This assumes that the Bayesian score follows a normal distribution and is different from the prediction using a cutoff.
Enrichment: An estimate of enrichment, that is, the increased likelihood (versus random) of this sample being in the category.
Bayesian Score: The standard Laplacian-modified Bayesian score.
Mahalanobis Distance: The Mahalanobis distance (MD) is the distance to the center of the training data. The larger the MD, the less trustworthy the prediction.
Mahalanobis Distance p-value: The p-value gives the fraction of training data with an MD greater than or equal to the one for the given sample, assuming normally distributed data. The smaller the p-value, the less trustworthy the prediciton. For highly non-normal X properties (e.g., fingerprints), the MD p-value is wildly inaccurate.

Structural Similar Compounds

Name	Rhodamine 6G	Curcumin	C.I. pigment red 23
Structure	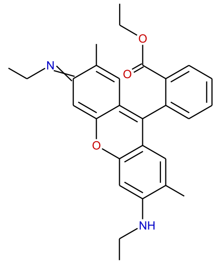	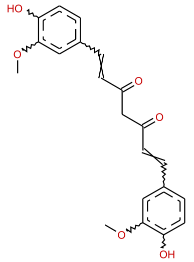	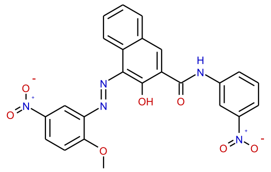
Actual Endpoint	Carcinogen	Non-Carcinogen	Carcinogen
Predicted Endpoint	Carcinogen	Non-Carcinogen	Carcinogen
Distance	0.680	0.719	0.785
Reference	NTP364	NTP427	NTP411

Model Applicability

Unknown features are fingerprint features in the query molecule, but not found or appearing too infrequently in the training set.

1. All properties and OPS components are within expected ranges.
2. Unknown ECFP_2 feature: -782828288: [*]C(=[*])[c]1:[nH]:[*]:[*]:[c]:1[*]
3. Unknown ECFP_2 feature: -962771238: [*]C(=[*])N(C(=[*])[*])[c](:[*]):[*]
4. Unknown ECFP_2 feature: -962137479: [*][c]1:[*]:[*]:[c](:[*]):[c]:1N=[*]
5. Unknown ECFP_2 feature: 676970202: [*]S\C(=N\[*])\N([*])[*]
6. Unknown ECFP_2 feature: 2085698692: [*]C(=N[c](:[*]):[*])[*]
7. Unknown ECFP_2 feature: 1427820655: [*]CSC(=[*])[*]

Feature Contribution

Top features for positive contribution

Fingerprint	Bit/Smiles	Feature Structure	Score	Carcinogen in training set
ECFP_12	1639858918	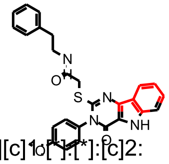 [*][c]1o[*]:[*]:[c]2:[*]:[cH]:[cH]:[cH]:[c]:1:2	0.47	7 out of 8

ECFP_12	1306977740	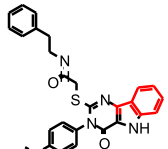 [*][c]1:[*]:[*]:[c]2:[cH]:[cH]:[cH]:[cH]:[c]:1:2	0.442	6 out of 7
ECFP_12	1333660716	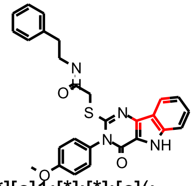 [*][c]1:[*]:[*]:[c](:[*]):[c]:1:[cH]:[*]	0.405	8 out of 10
**Top Features for negative contribution**				
**Fingerprint**	**Bit/Smiles**	**Feature Structure**	**Score**	**Carcinogen in training set**
ECFP_12	-1791034651	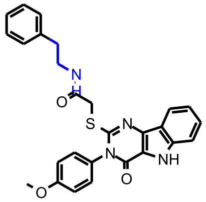 [*]CCN[*]	-1.39	0 out of 6
ECFP_12	-1795525632	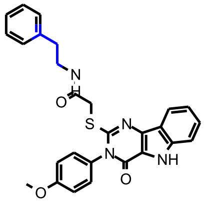 [*]CC[c](:[*]):[*]	-1.1	0 out of 4
ECFP_12	1731843802	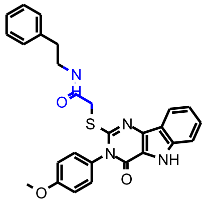 [*]CC(=O)N[*]	-1.1	0 out of 4

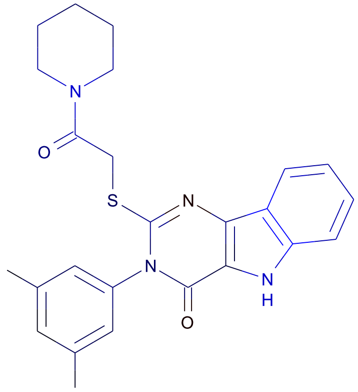

$C_{25}H_{26}N_4O_2S$
Molecular Weight: 446.56453
ALogP: 5.553
Rotatable Bonds: 4
Acceptors: 4
Donors: 1

Model Prediction

Prediction: Mild
Probability: 0.0166
Enrichment: 0.0451
Bayesian Score: -14.4
Mahalanobis Distance: 10.5
Mahalanobis Distance p-value: 0.00526

Prediction: Positive if the Bayesian score is above the estimated best cutoff value from minimizing the false positive and false negative rate.
Probability: The esimated probability that the sample is in the positive category. This assumes that the Bayesian score follows a normal distribution and is different from the prediction using a cutoff.
Enrichment: An estimate of enrichment, that is, the increased likelihood (versus random) of this sample being in the category.
Bayesian Score: The standard Laplacian-modified Bayesian score.
Mahalanobis Distance: The Mahalanobis distance (MD) is the distance to the center of the training data. The larger the MD, the less trustworthy the prediction.
Mahalanobis Distance p-value: The p-value gives the fraction of training data with an MD greater than or equal to the one for the given sample, assuming normally distributed data. The smaller the p-value, the less trustworthy the prediciton. For highly non-normal X properties (e.g., fingerprints), the MD p-value is wildly inaccurate.

Structural Similar Compounds

Name	Anthraquinone, 1,1'-iminodi-	(3,3'-Bianthra(1,9-cd)pyrazole)-6,6'(1H,1'H)-dione, 1,1'-diethyl-	Phosphorothioic acid, O-ethyl S-propyl O-(2,4,6-trichlorophenyl) es ter
Structure	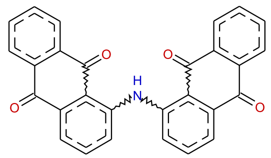	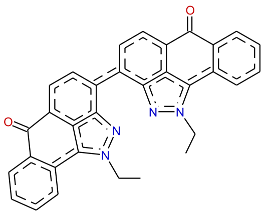	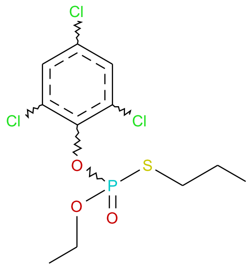
Actual Endpoint	Mild	Mild	Moderate_Severe
Predicted Endpoint	Mild	Mild	Mild
Distance	0.625	0.775	0.789
Reference	85JCAE "Prehled Prumyslove Toxikologie; Organicke Latky," Marhold, J., Prague , Czechoslovakia, Avicenum, 1986 Volume(issue)/page/year: -,735,1986	85JCAE "Prehled Prumyslove Toxikologie; Organicke Latky," Marhold, J., Prague , Czechoslovakia, Avicenum, 1986 Volume(issue)/page/year: -,1325,1986	NTIS** National Technical Information Service. (Springfield, VA 22161) Forme rly U.S. Clearinghouse for Scientific & Technical Information. Volume(issue)/pag e/year: OTS0535844

Model Applicability

Unknown features are fingerprint features in the query molecule, but not found or appearing too infrequently in the training set.

1. All properties and OPS components are within expected ranges.
2. Unknown FCFP_2 feature: 203707511: [*]C(=[*])[c]1:[nH]:[*]:[*]:[c]:1[*]

Feature Contribution

Top features for positive contribution				
**Fingerprint**	**Bit/Smiles**	**Feature Structure**	**Score**	**Moderate_Severe in training set**

FCFP_12	580453787	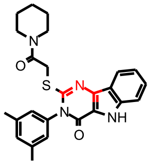 [*]C(=N[c](:[*]):[*])[*]	0.64	5 out of 6
FCFP_12	-1549163031	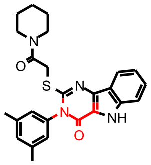 [*]N([*])C(=O)[c](:[*]):[*]	0.371	25 out of 47
FCFP_12	-1272798659	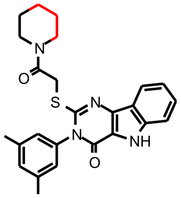 [*]CCC[*]	0.204	227 out of 513
**Top Features for negative contribution**				
**Fingerprint**	**Bit/Smiles**	**Feature Structure**	**Score**	**Moderate_Severe in training set**
FCFP_12	1604677718	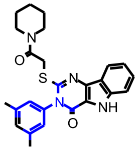 [*]C(=[*])N(C(=[*])[*])[c]1:[cH]:[c]([*]):[*]:[c]([*]):[cH]:1	-1.15	0 out of 6
FCFP_12	675769755	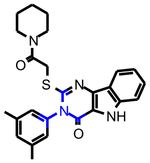 [*]C(=[*])N(C(=[*])[*])[c](:[*]):[*]	-1.05	1 out of 13

FCFP_12	307419094	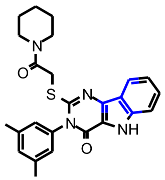 [*][c]1:[*]:[*]:[c](:[*]):[c]:1:[cH]:[*]	-0.915	2 out of 18

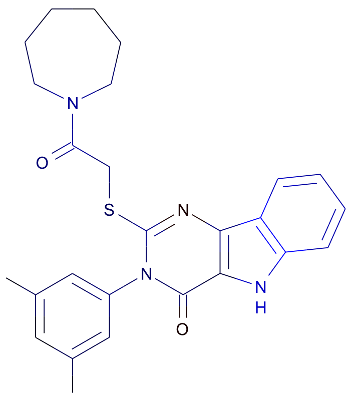

$C_{26}H_{28}N_4O_2S$

Molecular Weight: 460.59111

ALogP: 6.009

Rotatable Bonds: 4

Acceptors: 4

Donors: 1

Structural Similar Compounds

Name	Anthraquinone, 1,1'-iminodi-	(3,3'-Bianthra(1,9-cd)pyrazole)-6,6'(1H,1'H)-dione, 1,1'-diethyl-	Phosphorothioic acid, O-ethyl S-propyl O-(2,4,6-trichlorophenyl) es ter
Structure	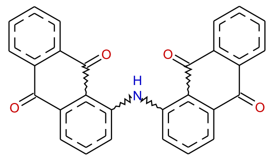	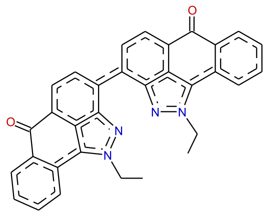	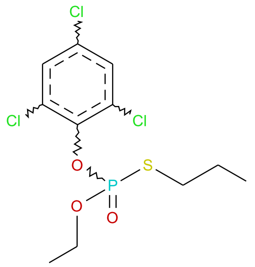
Actual Endpoint	Mild	Mild	Moderate_Severe
Predicted Endpoint	Mild	Mild	Mild
Distance	0.645	0.752	0.810
Reference	85JCAE "Prehled Prumyslove Toxikologie; Organicke Latky," Marhold, J., Prague , Czechoslovakia, Avicenum, 1986 Volume(issue)/page/year: -,735,1986	85JCAE "Prehled Prumyslove Toxikologie; Organicke Latky," Marhold, J., Prague , Czechoslovakia, Avicenum, 1986 Volume(issue)/page/year: -,1325,1986	NTIS** National Technical Information Service. (Springfield, VA 22161) Forme rly U.S. Clearinghouse for Scientific & Technical Information. Volume(issue)/pag e/year: OTS0535844

Model Prediction

Prediction: Mild

Probability: 0.0258

Enrichment: 0.0702

Bayesian Score: -13.1

Mahalanobis Distance: 15.7

Mahalanobis Distance p-value: 3.5e-017

Prediction: Positive if the Bayesian score is above the estimated best cutoff value from minimizing the false positive and false negative rate.
Probability: The esimated probability that the sample is in the positive category. This assumes that the Bayesian score follows a normal distribution and is different from the prediction using a cutoff.
Enrichment: An estimate of enrichment, that is, the increased likelihood (versus random) of this sample being in the category.
Bayesian Score: The standard Laplacian-modified Bayesian score.
Mahalanobis Distance: The Mahalanobis distance (MD) is the distance to the center of the training data. The larger the MD, the less trustworthy the prediction.
Mahalanobis Distance p-value: The p-value gives the fraction of training data with an MD greater than or equal to the one for the given sample, assuming normally distributed data. The smaller the p-value, the less trustworthy the prediciton. For highly non-normal X properties (e.g., fingerprints), the MD p-value is wildly inaccurate.

Model Applicability

Unknown features are fingerprint features in the query molecule, but not found or appearing too infrequently in the training set.

1. All properties and OPS components are within expected ranges.
2. Unknown FCFP_2 feature: 203707511: [*]C(=[*])[c]1:[nH]:[*]:[*]:[c]:1[*]

Feature Contribution

Top features for positive contribution

Fingerprint	Bit/Smiles	Feature Structure	Score	Moderate_Severe in training set

FCFP_12	580453787	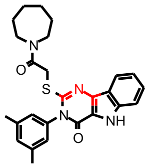 [*]C(=N[c](:[*]):[*])[*]	0.64	5 out of 6
FCFP_12	-1549163031	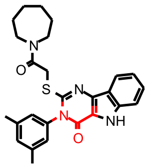 [*]N([*])C(=O)[c](:[*]):[*]	0.371	25 out of 47
FCFP_12	-1272798659	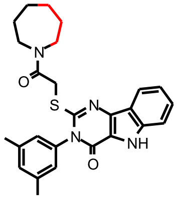 [*]CCC[*]	0.204	227 out of 513
**Top Features for negative contribution**				
**Fingerprint**	**Bit/Smiles**	**Feature Structure**	**Score**	**Moderate_Severe in training set**
FCFP_12	1604677718	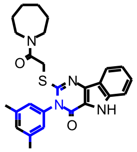 [*]C(=[*])N(C(=[*])[*])[c]1:[cH]:[c]([*]):[*]:[c]([*]):[cH]:1	-1.15	0 out of 6
FCFP_12	675769755	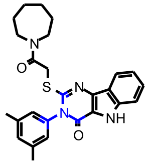 [*]C(=[*])N(C(=[*])[*])[c](:[*]):[*]	-1.05	1 out of 13

FCFP_12	307419094	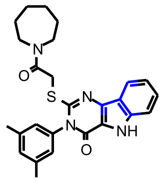 [*][c]1:[*]:[*]:[c](:[*]):[c]:1:[cH]:[*]	-0.915	2 out of 18

#UNDEFINED

TOPKAT_Skin_Irritancy_Mild_vs_Moderate_Severe

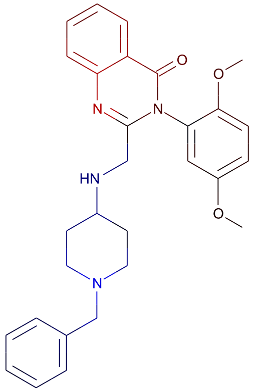

$C_{29}H_{32}N_4O_3$

Molecular Weight: 484.58938

ALogP: 3.743

Rotatable Bonds: 8

Acceptors: 6

Donors: 1

Model Prediction

Prediction: Mild

Probability: 0.228

Enrichment: 0.618

Bayesian Score: -4.44

Mahalanobis Distance: 13.8

Mahalanobis Distance p-value: 1.55e-010

Prediction: Positive if the Bayesian score is above the estimated best cutoff value from minimizing the false positive and false negative rate.
Probability: The esimated probability that the sample is in the positive category. This assumes that the Bayesian score follows a normal distribution and is different from the prediction using a cutoff.
Enrichment: An estimate of enrichment, that is, the increased likelihood (versus random) of this sample being in the category.
Bayesian Score: The standard Laplacian-modified Bayesian score.
Mahalanobis Distance: The Mahalanobis distance (MD) is the distance to the center of the training data. The larger the MD, the less trustworthy the prediction.
Mahalanobis Distance p-value: The p-value gives the fraction of training data with an MD greater than or equal to the one for the given sample, assuming normally distributed data. The smaller the p-value, the less trustworthy the prediciton. For highly non-normal X properties (e.g., fingerprints), the MD p-value is wildly inaccurate.

Structural Similar Compounds

Name	Ethanone, 2-((4-(2,4-dichloro-3-methylbenzoyl)-1,3-dimethyl-1H-pyra zol-5-yl)oxy)- 1-(4-methylphenyl)-	Carbamic acid, ((dibutylamino)thio)methyl-, 2,2-dimethyl-2,3-dihydro-7-benzofuranyl ester	Propanoic acid, 2-(4-((5-(trifluoromethyl)-2-pyridinyl)oxy)phenoxy) -, butyl ester
Structure	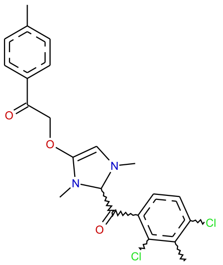	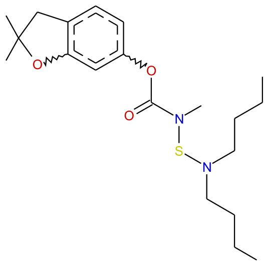	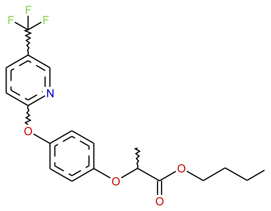
Actual Endpoint	Mild	Mild	Mild
Predicted Endpoint	Mild	Mild	Mild
Distance	0.805	0.810	0.811
Reference	NNGADV Nippon Noyaku Gakkaishi. Journal of the Pesticide Science Society of Japan. (Nippon Noyaku Gakkai, 1-43-11, Komagome, Toshima-ku, Tokyo 170, Japan) V.1-1976- Volume(issue)/page/year: 15,125,1990	NTIS** National Technical Information Service. (Springfield, VA 22161) Forme rly U.S. Clearinghouse for Scientific & Technical Information. Volume(issue)/pag e/year: OTS0539690	NNGADV Nippon Noyaku Gakkaishi. Journal of the Pesticide Science Society of Japan. (Nippon Noyaku Gakkai, 1-43-11, Komagome, Toshima-ku, Tokyo 170, Japan) V.1-1976- Volume(issue)/page/year: 15,305,1990

Model Applicability

Unknown features are fingerprint features in the query molecule, but not found or appearing too infrequently in the training set.

1. All properties and OPS components are within expected ranges.

Feature Contribution

Top features for positive contribution				
Fingerprint	Bit/Smiles	Feature Structure	Score	Moderate_Severe in training set

FCFP_12	1390383185	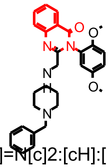 [*]N1[*]=N[c]2:[cH]:[cH]:[cH]:[cH]:[c]:2C1=O	0.761	5 out of 5
FCFP_12	562091192	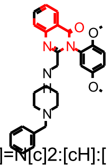 [*]N1[*]=N[c]2:[cH]:[*]:[cH]:[cH]:[c]:2C1=O	0.761	5 out of 5
FCFP_12	1150094517	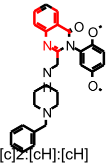 [*]C1=N[c]2:[cH]:[cH]:[*]:[cH]:[c]:2C(=[*])[*]1	0.761	5 out of 5

Top Features for negative contribution

Fingerprint	Bit/Smiles	Feature Structure	Score	Moderate_Severe in training set
FCFP_12	-885550502	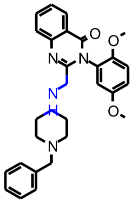 [*]CNC([*])[*]	-1.05	2 out of 21
FCFP_12	675769755	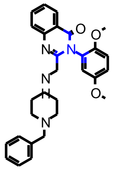 [*]C(=[*])N(C(=[*])[*])[c](:[*]):[*]	-1.05	1 out of 13

FCFP_12	309602933	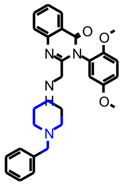 [*]CN1C[*]C([*])CC1	-1.03	0 out of 5

Molecule

TOPKAT_Skin_Irritancy_None_vs_Irritant

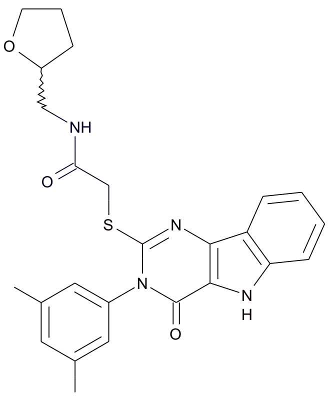

$C_{25}H_{26}N_4O_3S$

Molecular Weight: 462.56393

ALogP: 4.789

Rotatable Bonds: 6

Acceptors: 5

Donors: 2

Model Prediction

Prediction: Non-Irritant

Probability: 0.972

Enrichment: 1.05

Bayesian Score: -0.896

Mahalanobis Distance: 12.4

Mahalanobis Distance p-value: 3.47e-006

Prediction: Positive if the Bayesian score is above the estimated best cutoff value from minimizing the false positive and false negative rate.
Probability: The esimated probability that the sample is in the positive category. This assumes that the Bayesian score follows a normal distribution and is different from the prediction using a cutoff.
Enrichment: An estimate of enrichment, that is, the increased likelihood (versus random) of this sample being in the category.
Bayesian Score: The standard Laplacian-modified Bayesian score.
Mahalanobis Distance: The Mahalanobis distance (MD) is the distance to the center of the training data. The larger the MD, the less trustworthy the prediction.
Mahalanobis Distance p-value: The p-value gives the fraction of training data with an MD greater than or equal to the one for the given sample, assuming normally distributed data. The smaller the p-value, the less trustworthy the prediciton. For highly non-normal X properties (e.g., fingerprints), the MD p-value is wildly inaccurate.

Structural Similar Compounds

Name	Benzenesulfonic acid, 2,2'-(4,4'-biphenylylenedivinylene)di-, disod ium salt	Anthraquinone, 1,1'-iminodi-	2-Anthracenesulfonic acid, 1-amino-9,10-dihydro-9,10-dioxo-4-(2,4,6 -trimethylanilino)-, monosodium salt
Structure	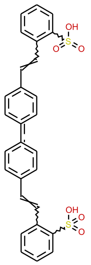	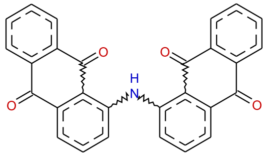	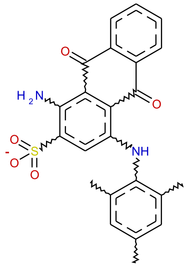
Actual Endpoint	Irritant	Irritant	Irritant
Predicted Endpoint	Non-Irritant	Non-Irritant	Non-Irritant
Distance	0.682	0.807	0.855
Reference	MVCRB3 MVC-Report. (Stockholm, Sweden) No.1-2, 1972-73. Discontinued. Volu me(issue)/page/year: 2,193,1973	85JCAE "Prehled Prumyslove Toxikologie; Organicke Latky," Marhold, J., Prague , Czechoslovakia, Avicenum, 1986 Volume(issue)/page/year: -,735,1986	85JCAE "Prehled Prumyslove Toxikologie; Organicke Latky," Marhold, J., Prague , Czechoslovakia, Avicenum, 1986 Volume(issue)/page/year: -,1327,1986

Model Applicability

Unknown features are fingerprint features in the query molecule, but not found or appearing too infrequently in the training set.

1. OPS PC22 out of range. Value: 3.8534. Training min, max, SD, explained variance: -2.9568, 3.7845, 1.016, 0.0131.
2. Unknown FCFP_2 feature: 203707511: [*]C(=[*])[c]1:[nH]:[*]:[*]:[c]:1[*]

Feature Contribution

Top features for positive contribution				
Fingerprint	Bit/Smiles	Feature Structure	Score	Irritant in training set

FCFP_12	-1986158408	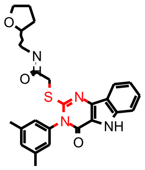 [*]S\C(=N\[*])\N([*])[*]	0.0821	13 out of 13
FCFP_12	-1475780652	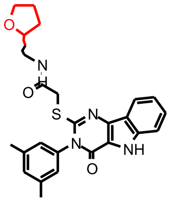 [*]C1CCCO1	0.0805	132 out of 133
FCFP_12	580453787	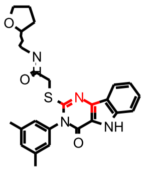 [*]C(=N[c](:[*]):[*])[*]	0.0795	9 out of 9
**Top Features for negative contribution**				
**Fingerprint**	**Bit/Smiles**	**Feature Structure**	**Score**	**Irritant in training set**
FCFP_12	566058135	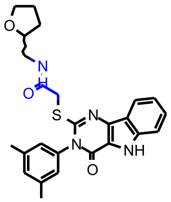 [*]CC(=O)N[*]	-0.367	13 out of 21
FCFP_12	-547731249	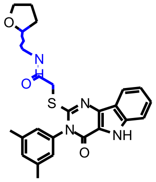 [*]CC(=O)NCC([*])[*]	-0.222	2 out of 3

FCFP_12	-451251206	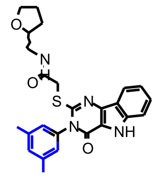 C[c]1:[cH]:[*]:[cH]:[c](C):[cH]:1	-0.132	44 out of 55

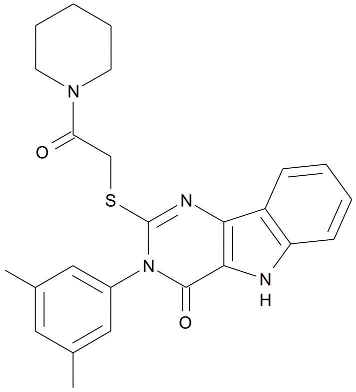

$C_{25}H_{26}N_4O_2S$
Molecular Weight: 446.56453
ALogP: 5.553
Rotatable Bonds: 4
Acceptors: 4
Donors: 1

Model Prediction

Prediction: Irritant
Probability: 0.976
Enrichment: 1.06
Bayesian Score: -0.392
Mahalanobis Distance: 12.3
Mahalanobis Distance p-value: 5.33e-006

Prediction: Positive if the Bayesian score is above the estimated best cutoff value from minimizing the false positive and false negative rate.
Probability: The esimated probability that the sample is in the positive category. This assumes that the Bayesian score follows a normal distribution and is different from the prediction using a cutoff.
Enrichment: An estimate of enrichment, that is, the increased likelihood (versus random) of this sample being in the category.
Bayesian Score: The standard Laplacian-modified Bayesian score.
Mahalanobis Distance: The Mahalanobis distance (MD) is the distance to the center of the training data. The larger the MD, the less trustworthy the prediction.
Mahalanobis Distance p-value: The p-value gives the fraction of training data with an MD greater than or equal to the one for the given sample, assuming normally distributed data. The smaller the p-value, the less trustworthy the prediciton. For highly non-normal X properties (e.g., fingerprints), the MD p-value is wildly inaccurate.

Structural Similar Compounds

Name	Anthraquinone, 1,1'-iminodi-	(3,3'-Bianthra(1,9-cd)pyrazole)-6,6'(1H,1'H)-dione, 1,1'-diethyl-	Phosphorothioic acid, O-ethyl S-propyl O-(2,4,6-trichlorophenyl) es ter
Structure	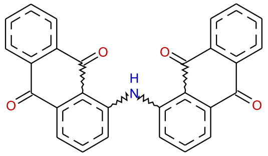	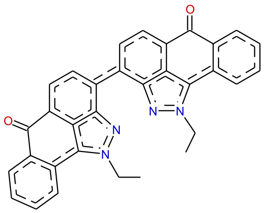	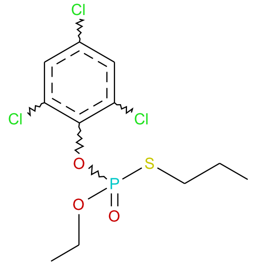
Actual Endpoint	Irritant	Irritant	Irritant
Predicted Endpoint	Non-Irritant	Non-Irritant	Irritant
Distance	0.632	0.773	0.790
Reference	85JCAE "Prehled Prumyslove Toxikologie; Organicke Latky," Marhold, J., Prague , Czechoslovakia, Avicenum, 1986 Volume(issue)/page/year: -,735,1986	85JCAE "Prehled Prumyslove Toxikologie; Organicke Latky," Marhold, J., Prague , Czechoslovakia, Avicenum, 1986 Volume(issue)/page/year: -,1325,1986	NTIS** National Technical Information Service. (Springfield, VA 22161) Forme rly U.S. Clearinghouse for Scientific & Technical Information. Volume(issue)/pag e/year: OTS0535844

Model Applicability

Unknown features are fingerprint features in the query molecule, but not found or appearing too infrequently in the training set.

1. All properties and OPS components are within expected ranges.
2. Unknown FCFP_2 feature: 203707511: [*]C(=[*])[c]1:[nH]:[*]:[*]:[c]:1[*]

Feature Contribution

Top features for positive contribution

Fingerprint	Bit/Smiles	Feature Structure	Score	Irritant in training set

FCFP_12	-1986158408	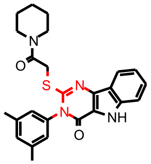 [*]S\C(=N\[*])\N([*])[*]	0.0821	13 out of 13
FCFP_12	580453787	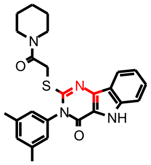 [*]C(=N[c](:[*]):[*])[*]	0.0795	9 out of 9
FCFP_12	1604677718	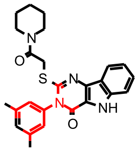 [*]C(=[*])N(C(=[*])[*])[c]1:[cH]:[c]([*]):[*]:[c]([*]):[cH]:1	0.0785	8 out of 8
**Top Features for negative contribution**				
**Fingerprint**	**Bit/Smiles**	**Feature Structure**	**Score**	**Irritant in training set**
FCFP_12	-451251206	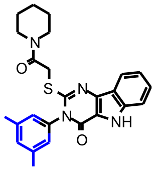 C[c]1:[cH]:[*]:[cH]:[c](C):[cH]:1	-0.132	44 out of 55
FCFP_12	-1320007763	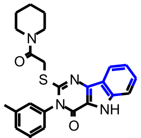 [*][c]1:[*]:[*]:[c]2:[*]:[cH]:[cH]:[cH]:[c]:1:2	-0.0893	20 out of 24

FCFP_12	675769755	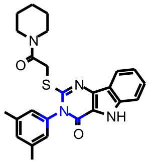 [*]C(=[*])N(C(=[*])[*])[c](:[*]):[*]	-0.088	15 out of 18

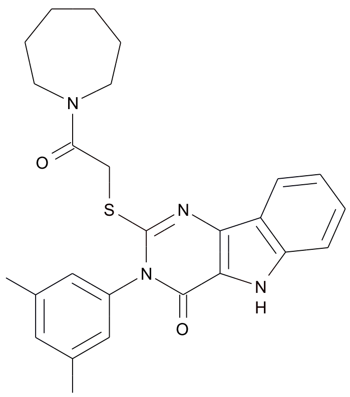

$C_{26}H_{28}N_4O_2S$
Molecular Weight: 460.59111
ALogP: 6.009
Rotatable Bonds: 4
Acceptors: 4
Donors: 1

Model Prediction

Prediction: Irritant
Probability: 0.977
Enrichment: 1.06
Bayesian Score: -0.252
Mahalanobis Distance: 15.2
Mahalanobis Distance p-value: 5.78e-015

Prediction: Positive if the Bayesian score is above the estimated best cutoff value from minimizing the false positive and false negative rate.
Probability: The esimated probability that the sample is in the positive category. This assumes that the Bayesian score follows a normal distribution and is different from the prediction using a cutoff.
Enrichment: An estimate of enrichment, that is, the increased likelihood (versus random) of this sample being in the category.
Bayesian Score: The standard Laplacian-modified Bayesian score.
Mahalanobis Distance: The Mahalanobis distance (MD) is the distance to the center of the training data. The larger the MD, the less trustworthy the prediction.
Mahalanobis Distance p-value: The p-value gives the fraction of training data with an MD greater than or equal to the one for the given sample, assuming normally distributed data. The smaller the p-value, the less trustworthy the prediciton. For highly non-normal X properties (e.g., fingerprints), the MD p-value is wildly inaccurate.

Structural Similar Compounds

Name	Anthraquinone, 1,1'-iminodi-	(3,3'-Bianthra(1,9-cd)pyrazole)-6,6'(1H,1'H)-dione, 1,1'-diethyl-	Phosphorothioic acid, O-ethyl S-propyl O-(2,4,6-trichlorophenyl) es ter
Structure	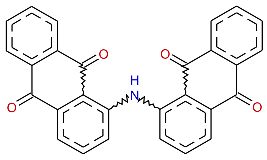	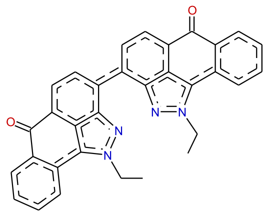	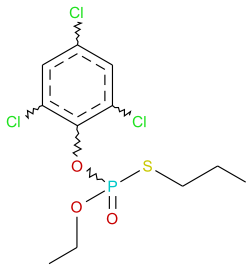
Actual Endpoint	Irritant	Irritant	Irritant
Predicted Endpoint	Non-Irritant	Non-Irritant	Irritant
Distance	0.652	0.749	0.813
Reference	85JCAE "Prehled Prumyslove Toxikologie; Organicke Latky," Marhold, J., Prague , Czechoslovakia, Avicenum, 1986 Volume(issue)/page/year: -,735,1986	85JCAE "Prehled Prumyslove Toxikologie; Organicke Latky," Marhold, J., Prague , Czechoslovakia, Avicenum, 1986 Volume(issue)/page/year: -,1325,1986	NTIS** National Technical Information Service. (Springfield, VA 22161) Forme rly U.S. Clearinghouse for Scientific & Technical Information. Volume(issue)/pag e/year: OTS0535844

Model Applicability

Unknown features are fingerprint features in the query molecule, but not found or appearing too infrequently in the training set.

1. All properties and OPS components are within expected ranges.
2. Unknown FCFP_2 feature: 203707511: [*]C(=[*])[c]1:[nH]:[*]:[*]:[c]:1[*]

Feature Contribution

Top features for positive contribution

Fingerprint	Bit/Smiles	Feature Structure	Score	Irritant in training set

FCFP_12	-1986158408	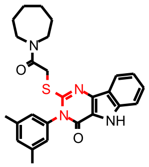 [*]S\C(=N\[*])\N([*]) [*]	0.0821	13 out of 13
FCFP_12	580453787	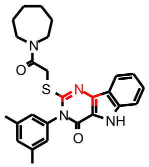 [*]C(=N[c](:[*]):[*]) [*]	0.0795	9 out of 9
FCFP_12	1604677718	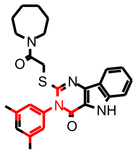 [*]C(=[*])N(C(=[*])[*])[c]1:[cH]:[c]([*]) :[*]:[c]([*]):[cH]:1	0.0785	8 out of 8

Top Features for negative contribution

Fingerprint	Bit/Smiles	Feature Structure	Score	Irritant in training set
FCFP_12	-451251206	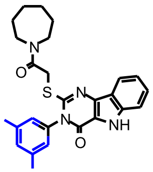 C[c]1:[cH]:[*]:[cH]:[c](C):[cH]:1	-0.132	44 out of 55
FCFP_12	-1320007763	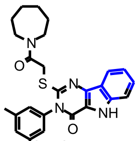 [*][c]1:[*]:[*]:[c]2: [*]:[cH]:[cH]:[cH]:[c]:1:2	-0.0893	20 out of 24

FCFP_12	675769755	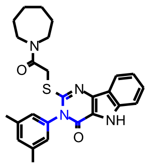<br>[*]C(=[*])N(C(=[*])[*])[c](:[*]):[*]	-0.088	15 out of 18

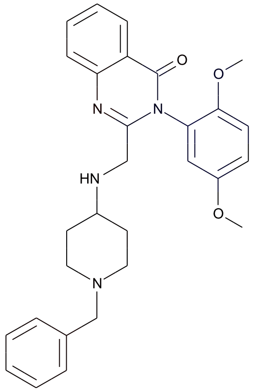

$C_{29}H_{32}N_4O_3$
Molecular Weight: 484.58938
ALogP: 3.743
Rotatable Bonds: 8
Acceptors: 6
Donors: 1

Model Prediction

Prediction: Irritant
Probability: 0.974
Enrichment: 1.06
Bayesian Score: -0.666
Mahalanobis Distance: 12.8
Mahalanobis Distance p-value: 2.78e-007

Prediction: Positive if the Bayesian score is above the estimated best cutoff value from minimizing the false positive and false negative rate.
Probability: The esimated probability that the sample is in the positive category. This assumes that the Bayesian score follows a normal distribution and is different from the prediction using a cutoff.
Enrichment: An estimate of enrichment, that is, the increased likelihood (versus random) of this sample being in the category.
Bayesian Score: The standard Laplacian-modified Bayesian score.
Mahalanobis Distance: The Mahalanobis distance (MD) is the distance to the center of the training data. The larger the MD, the less trustworthy the prediction.
Mahalanobis Distance p-value: The p-value gives the fraction of training data with an MD greater than or equal to the one for the given sample, assuming normally distributed data. The smaller the p-value, the less trustworthy the prediciton. For highly non-normal X properties (e.g., fingerprints), the MD p-value is wildly inaccurate.

Structural Similar Compounds

Name	1-Piperazineacetic acid, 4-(2-hydroxyethyl)-alpha-phenyl-, 2,6-xyly l ester, monohydrochloride	Ethanone, 2-((4-(2,4-dichloro-3-methylbenzoyl)-1,3-dimethyl-1H-pyra zol-5-yl)oxy)- 1-(4-methylphenyl)-	Carbamic acid, ((dibutylamino)thio)methyl -, 2,2-dimethyl-2,3-dihydr o-7-benzofuranyl ester
Structure	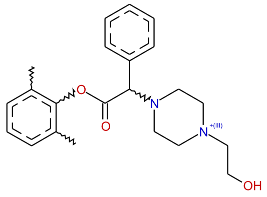	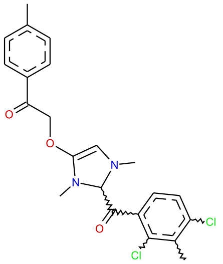	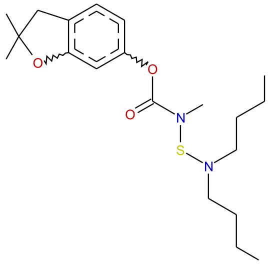
Actual Endpoint	Irritant	Irritant	Irritant
Predicted Endpoint	Irritant	Non-Irritant	Irritant
Distance	0.763	0.806	0.812
Reference	BCFAAI Bollettino Chimico Farmaceutico. (Societa Editoriale Farmaceutica, Vi a Ausonio 12, 20123 Milan, Italy) V.33- 1894- Volume(issue)/page/year: 107,3 10,1968	NNGADV Nippon Noyaku Gakkaishi. Journal of the Pesticide Science Society of Japan. (Nippon Noyaku Gakkai, 1-43-11, Komagome, Toshima-ku, Tokyo 170, Japan) V.1- 1976- Volume(issue)/page/year: 15,125,1990	NTIS** National Technical Information Service. (Springfield, VA 22161) Forme rly U.S. Clearinghouse for Scientific & Technical Information. Volume(issue)/pag e/year: OTS0539690

Model Applicability

Unknown features are fingerprint features in the query molecule, but not found or appearing too infrequently in the training set.

1. OPS PC22 out of range. Value: 3.909. Training min, max, SD, explained variance: -2.9568, 3.7845, 1.016, 0.0131.

Feature Contribution

Top features for positive contribution				
Fingerprint	Bit/Smiles	Feature Structure	Score	Irritant in training set

FCFP_12	580453787	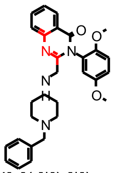 [*]C(=N[c](:[*]):[*])[*]	0.0795	9 out of 9
FCFP_12	1390383185	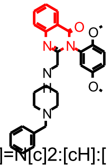 [*]N1[*]=N[c]2:[cH]:[cH]:[cH]:[cH]:[c]:2C1=O	0.0756	6 out of 6
FCFP_12	1150094517	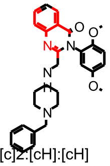 [*]C1=N[c]2:[cH]:[cH]:[*]:[cH]:[c]:2C(=[*])[*]1	0.0756	6 out of 6

Top Features for negative contribution

**Fingerprint**	**Bit/Smiles**	**Feature Structure**	**Score**	**Irritant in training set**
FCFP_12	-2007573814	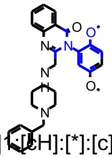 [*]O[c]1:[cH]:[*]:[c]([*]):[cH]:[c]:1N(C(=[*])[*])C(=[*])[*]	-0.65	0 out of 1
FCFP_12	309602933	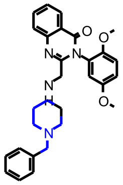 [*]CN1C[*]C([*])CC1	-0.205	11 out of 15

FCFP_12	346218766	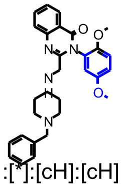 [*][c]1:[*]:[cH]:[cH]:[c](OC):[cH]:1	-0.0911	35 out of 42

#UNDEFINED

TOPKAT_Skin_Irritancy_None_vs_Irritant

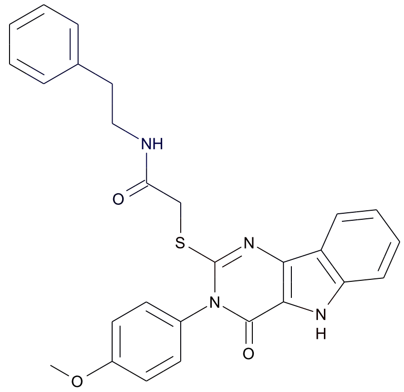

$C_{27}H_{24}N_4O_3S$

Molecular Weight: 484.56946

ALogP: 5.346

Rotatable Bonds: 8

Acceptors: 5

Donors: 2

Model Prediction

Prediction: Non-Irritant

Probability: 0.95

Enrichment: 1.03

Bayesian Score: -1.89

Mahalanobis Distance: 10.3

Mahalanobis Distance p-value: 0.0304

Prediction: Positive if the Bayesian score is above the estimated best cutoff value from minimizing the false positive and false negative rate.
Probability: The esimated probability that the sample is in the positive category. This assumes that the Bayesian score follows a normal distribution and is different from the prediction using a cutoff.
Enrichment: An estimate of enrichment, that is, the increased likelihood (versus random) of this sample being in the category.
Bayesian Score: The standard Laplacian-modified Bayesian score.
Mahalanobis Distance: The Mahalanobis distance (MD) is the distance to the center of the training data. The larger the MD, the less trustworthy the prediction.
Mahalanobis Distance p-value: The p-value gives the fraction of training data with an MD greater than or equal to the one for the given sample, assuming normally distributed data. The smaller the p-value, the less trustworthy the prediciton. For highly non-normal X properties (e.g., fingerprints), the MD p-value is wildly inaccurate.

Structural Similar Compounds

Name	Benzenesulfonic acid, 2,2'-(4,4'-biphenylylenedivinylene)di-, disod ium salt	Anthraquinone, 1,1'-iminodi-	Sulfide, bis(4-t-butyl-m-cresyl)-
Structure	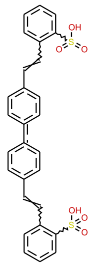	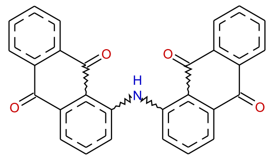	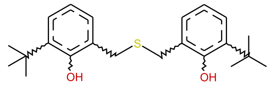
Actual Endpoint	Irritant	Irritant	Irritant
Predicted Endpoint	Non-Irritant	Non-Irritant	Irritant
Distance	0.632	0.862	0.914
Reference	MVCRB3 MVC-Report. (Stockholm, Sweden) No.1-2, 1972-73. Discontinued. Volu me(issue)/page/year: 2,193,1973	85JCAE "Prehled Prumyslove Toxikologie; Organicke Latky," Marhold, J., Prague , Czechoslovakia, Avicenum, 1986 Volume(issue)/page/year: -,735,1986	AMIHBC AMA Archives of Industrial Hygiene and Occupational Medicine. (Chicago , IL) V.2-10, 1950-54. For publisher information, see AEHLAU. Volume(issue)/pag e/year: 5,311,1952

Model Applicability

Unknown features are fingerprint features in the query molecule, but not found or appearing too infrequently in the training set.

1. OPS PC22 out of range. Value: 4.0777. Training min, max, SD, explained variance: -2.9568, 3.7845, 1.016, 0.0131.
2. Unknown FCFP_2 feature: 203707511: [*]C(=[*])[c]1:[nH]:[*]:[*]:[c]:1[*]

Feature Contribution

Top features for positive contribution				
Fingerprint	Bit/Smiles	Feature Structure	Score	Irritant in training set

FCFP_12	-1986158408	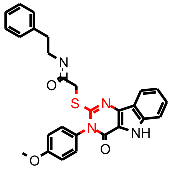 [*]S\C(=N\[*])\N([*]) [*]	0.0821	13 out of 13
FCFP_12	580453787	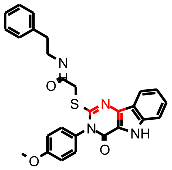 [*]C(=N[c](:[*]):[*]) [*]	0.0795	9 out of 9
FCFP_12	1604677718	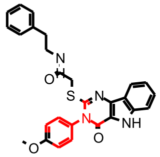 [*]C(=[*])N(C(=[*])[*])[c]1:[cH]:[c]([*]) :[*]:[c]([*]):[cH]:1	0.0785	8 out of 8

Top Features for negative contribution

**Fingerprint**	**Bit/Smiles**	**Feature Structure**	**Score**	**Irritant in training set**
FCFP_12	-2002900105	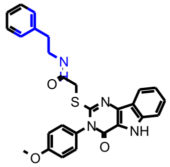 [*]NCC[c](:[cH]:[*]): [cH]:[*]	-0.65	0 out of 1
FCFP_12	566058135	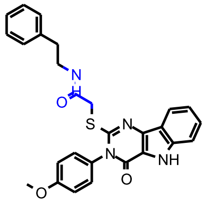 [*]CC(=O)N[*]	-0.367	13 out of 21

FCFP_12	-547731249	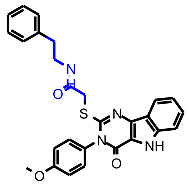 [*]CC(=O)NCC([*])[*]	-0.222	2 out of 3

Molecule

TOPKAT_Skin_Sensitization_None_vs_Sensitizer

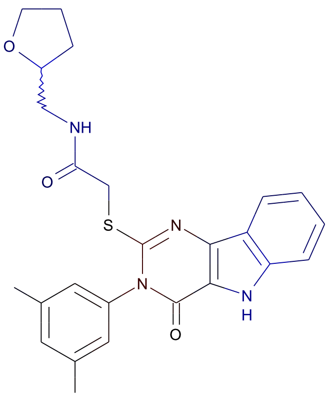

$C_{25}H_{26}N_4O_3S$

Molecular Weight: 462.56393

ALogP: 4.789

Rotatable Bonds: 6

Acceptors: 5

Donors: 2

Structural Similar Compounds

Name	Tixocortol pivalate	Mometasone furoate	Budesonide
Structure	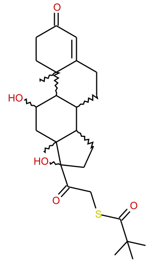	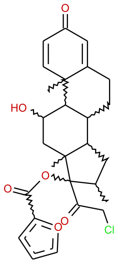	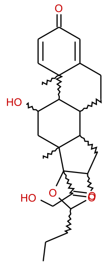
Actual Endpoint	Sensitizer	Sensitizer	Sensitizer
Predicted Endpoint	Sensitizer	Sensitizer	Sensitizer
Distance	0.619	0.671	0.745
Reference	Contact Dermatitis (1996) 34:161	Contact Dermatitis (1996) 34:161	Contact Dermatitis (1996) 34:161

Model Prediction

Prediction: Non-Sensitizer

Probability: 0.259

Enrichment: 0.377

Bayesian Score: -7.72

Mahalanobis Distance: 10.4

Mahalanobis Distance p-value: 2.9e-006

Prediction: Positive if the Bayesian score is above the estimated best cutoff value from minimizing the false positive and false negative rate.
Probability: The esimated probability that the sample is in the positive category. This assumes that the Bayesian score follows a normal distribution and is different from the prediction using a cutoff.
Enrichment: An estimate of enrichment, that is, the increased likelihood (versus random) of this sample being in the category.
Bayesian Score: The standard Laplacian-modified Bayesian score.
Mahalanobis Distance: The Mahalanobis distance (MD) is the distance to the center of the training data. The larger the MD, the less trustworthy the prediction.
Mahalanobis Distance p-value: The p-value gives the fraction of training data with an MD greater than or equal to the one for the given sample, assuming normally distributed data. The smaller the p-value, the less trustworthy the prediciton. For highly non-normal X properties (e.g., fingerprints), the MD p-value is wildly inaccurate.

Model Applicability

Unknown features are fingerprint features in the query molecule, but not found or appearing too infrequently in the training set.

1. All properties and OPS components are within expected ranges.
2. Unknown FCFP_2 feature: 203707511: [*]C(=[*])[c]1:[nH]:[*]:[*]:[c]:1[*]

Feature Contribution

Top features for positive contribution				
**Fingerprint**	**Bit/Smiles**	**Feature Structure**	**Score**	**Sensitizer in training set**
FCFP_12	-1986158408	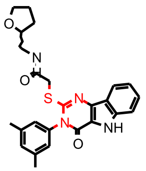 [*]S\C(=N\[*])\N([*])[*]	0.286	8 out of 8

FCFP_12	675769755	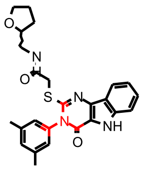 [*]C(=[*])N(C(=[*])[*])[c](:[*]):[*]	0.253	4 out of 4
FCFP_12	580453787	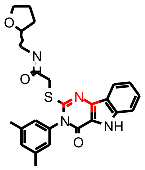 [*]C(=N[c](:[*]):[*]) [*]	0.236	3 out of 3

Top Features for negative contribution

**Fingerprint**	**Bit/Smiles**	**Feature Structure**	**Score**	**Sensitizer in training set**
FCFP_12	-547731249	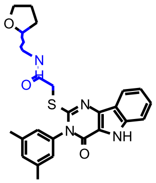 [*]CC(=O)NCC([*])[*]	-1.36	0 out of 4
FCFP_12	-989213044	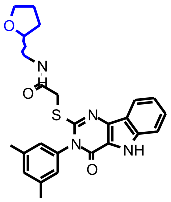 [*]CC1CCCO1	-0.663	1 out of 4
FCFP_12	19	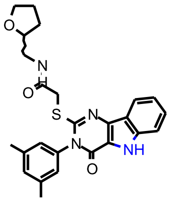 [*]:[nH]:[*]	-0.542	0 out of 1

#UNDEFINED

TOPKAT_Skin_Sensitization_None_vs_Sensitizer

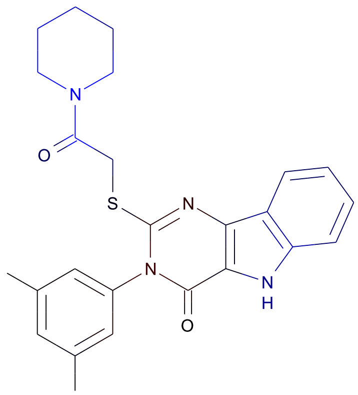

$C_{25}H_{26}N_4O_2S$

Molecular Weight: 446.56453

ALogP: 5.553

Rotatable Bonds: 4

Acceptors: 4

Donors: 1

Structural Similar Compounds

Name	Mometasone furoate	Sudan III	4;4'-Isopropylidene diphenol
Structure	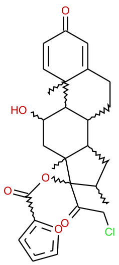	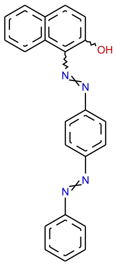	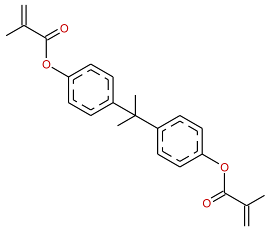
Actual Endpoint	Sensitizer	Non-Sensitizer	Sensitizer
Predicted Endpoint	Sensitizer	Non-Sensitizer	Sensitizer
Distance	0.654	0.726	0.774
Reference	Contact Dermatitis (1996) 34:161	Contact Dermatitis (1991) 25:313	Howard I Maibach (priv comm)

Model Prediction

Prediction: Non-Sensitizer

Probability: 0.156

Enrichment: 0.228

Bayesian Score: -9.4

Mahalanobis Distance: 10.5

Mahalanobis Distance p-value: 2.12e-006

Prediction: Positive if the Bayesian score is above the estimated best cutoff value from minimizing the false positive and false negative rate.
Probability: The esimated probability that the sample is in the positive category. This assumes that the Bayesian score follows a normal distribution and is different from the prediction using a cutoff.
Enrichment: An estimate of enrichment, that is, the increased likelihood (versus random) of this sample being in the category.
Bayesian Score: The standard Laplacian-modified Bayesian score.
Mahalanobis Distance: The Mahalanobis distance (MD) is the distance to the center of the training data. The larger the MD, the less trustworthy the prediction.
Mahalanobis Distance p-value: The p-value gives the fraction of training data with an MD greater than or equal to the one for the given sample, assuming normally distributed data. The smaller the p-value, the less trustworthy the prediciton. For highly non-normal X properties (e.g., fingerprints), the MD p-value is wildly inaccurate.

Model Applicability

Unknown features are fingerprint features in the query molecule, but not found or appearing too infrequently in the training set.

1. All properties and OPS components are within expected ranges.
2. Unknown FCFP_2 feature: 203707511: [*]C(=[*])[c]1:[nH]:[*]:[*]:[c]:1[*]

Feature Contribution

Top features for positive contribution

Fingerprint	Bit/Smiles	Feature Structure	Score	Sensitizer in training set
FCFP_12	-1986158408	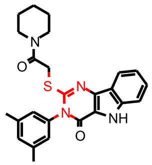 [*]S\C(=N\[*])\N([*])[*]	0.286	8 out of 8

FCFP_12	675769755	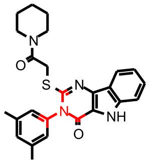 [*]C(=[*])N(C(=[*])[*<br>])[c](:[*]):[*]	0.253	4 out of 4
FCFP_12	580453787	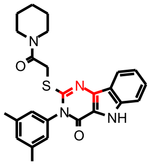 [*]C(=N[c](:[*]):[*])<br>[*]	0.236	3 out of 3
**Top Features for negative contribution**				
**Fingerprint**	**Bit/Smiles**	**Feature Structure**	**Score**	**Sensitizer in training set**
FCFP_12	-1474971978	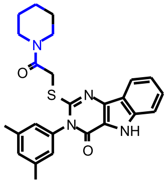 [*]C(=[*])N1C[*]CCC1	-1.53	0 out of 5
FCFP_12	-446103674	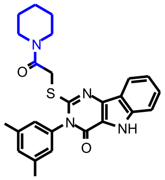 [*]C(=[*])N1CCCCC1	-0.892	0 out of 2
FCFP_12	-1553874037	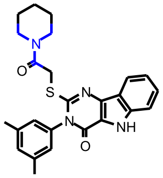 [*]CN(C[*])C(=[*])[*]	-0.802	3 out of 11

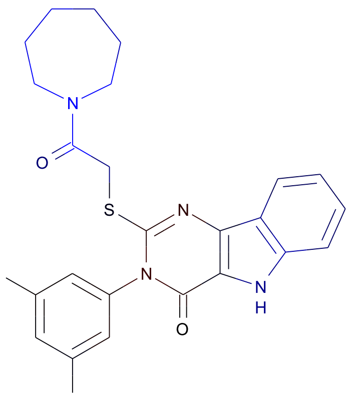

$C_{26}H_{28}N_4O_2S$
Molecular Weight: 460.59111
ALogP: 6.009
Rotatable Bonds: 4
Acceptors: 4
Donors: 1

Model Prediction

Prediction: Non-Sensitizer
Probability: 0.114
Enrichment: 0.166
Bayesian Score: -10.3
Mahalanobis Distance: 10.6
Mahalanobis Distance p-value: 9.92e-007

Prediction: Positive if the Bayesian score is above the estimated best cutoff value from minimizing the false positive and false negative rate.
Probability: The esimated probability that the sample is in the positive category. This assumes that the Bayesian score follows a normal distribution and is different from the prediction using a cutoff.
Enrichment: An estimate of enrichment, that is, the increased likelihood (versus random) of this sample being in the category.
Bayesian Score: The standard Laplacian-modified Bayesian score.
Mahalanobis Distance: The Mahalanobis distance (MD) is the distance to the center of the training data. The larger the MD, the less trustworthy the prediction.
Mahalanobis Distance p-value: The p-value gives the fraction of training data with an MD greater than or equal to the one for the given sample, assuming normally distributed data. The smaller the p-value, the less trustworthy the prediciton. For highly non-normal X properties (e.g., fingerprints), the MD p-value is wildly inaccurate.

Structural Similar Compounds

Name	Mometasone furoate	Sudan III	4;4'-Isopropylidene diphenol
Structure	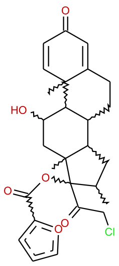	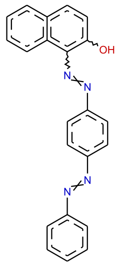	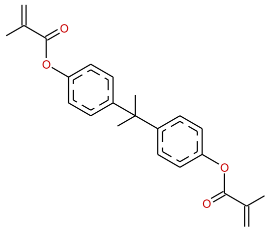
Actual Endpoint	Sensitizer	Non-Sensitizer	Sensitizer
Predicted Endpoint	Sensitizer	Non-Sensitizer	Sensitizer
Distance	0.673	0.728	0.783
Reference	Contact Dermatitis (1996) 34:161	Contact Dermatitis (1991) 25:313	Howard I Maibach (priv comm)

Model Applicability

Unknown features are fingerprint features in the query molecule, but not found or appearing too infrequently in the training set.

1. All properties and OPS components are within expected ranges.
2. Unknown FCFP_2 feature: 203707511: [*]C(=[*])[c]1:[nH]:[*]:[*]:[c]:1[*]

Feature Contribution

Top features for positive contribution

Fingerprint	Bit/Smiles	Feature Structure	Score	Sensitizer in training set
FCFP_12	-1986158408	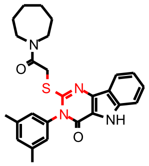 [*]S\C(=N\[*])\N([*])[*]	0.286	8 out of 8

FCFP_12	675769755	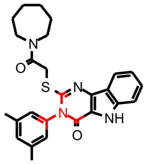 [*]C(=[*])N(C(=[*])[*])[c](:[*]):[*]	0.253	4 out of 4
FCFP_12	-776001689	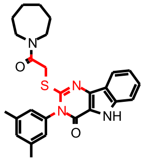 [*]\N=C(\SCC(=[*])[*])/N([*])[*]	0.236	3 out of 3

Top Features for negative contribution

**Fingerprint**	**Bit/Smiles**	**Feature Structure**	**Score**	**Sensitizer in training set**
FCFP_12	-1474971978	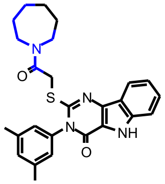 [*]C(=[*])N1C[*]CCC1	-1.53	0 out of 5
FCFP_12	-446103674	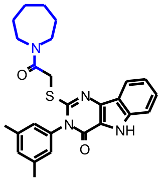 [*]C(=[*])N1CCCCC1	-0.892	0 out of 2
FCFP_12	-1553874037	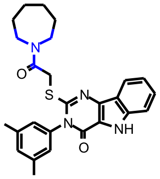 [*]CN(C[*])C(=[*])[*]	-0.802	3 out of 11

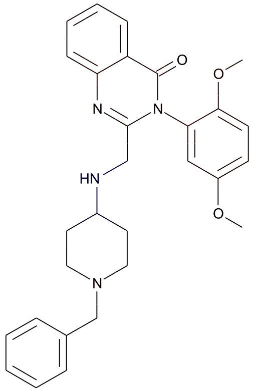

$C_{29}H_{32}N_4O_3$
Molecular Weight: 484.58938
ALogP: 3.743
Rotatable Bonds: 8
Acceptors: 6
Donors: 1

Model Prediction

Prediction: Sensitizer
Probability: 0.826
Enrichment: 1.2
Bayesian Score: 0.812
Mahalanobis Distance: 10.3
Mahalanobis Distance p-value: 6.6e-006

Prediction: Positive if the Bayesian score is above the estimated best cutoff value from minimizing the false positive and false negative rate.
Probability: The esimated probability that the sample is in the positive category. This assumes that the Bayesian score follows a normal distribution and is different from the prediction using a cutoff.
Enrichment: An estimate of enrichment, that is, the increased likelihood (versus random) of this sample being in the category.
Bayesian Score: The standard Laplacian-modified Bayesian score.
Mahalanobis Distance: The Mahalanobis distance (MD) is the distance to the center of the training data. The larger the MD, the less trustworthy the prediction.
Mahalanobis Distance p-value: The p-value gives the fraction of training data with an MD greater than or equal to the one for the given sample, assuming normally distributed data. The smaller the p-value, the less trustworthy the prediciton. For highly non-normal X properties (e.g., fingerprints), the MD p-value is wildly inaccurate.

Structural Similar Compounds

Name	Mometasone furoate	Tixocortol pivalate	Budesonide
Structure	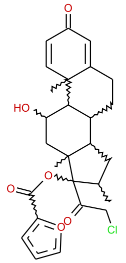	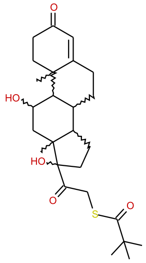	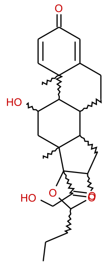
Actual Endpoint	Sensitizer	Sensitizer	Sensitizer
Predicted Endpoint	Sensitizer	Sensitizer	Sensitizer
Distance	0.663	0.735	0.789
Reference	Contact Dermatitis (1996) 34:161	Contact Dermatitis (1996) 34:161	Contact Dermatitis (1996) 34:161

Model Applicability

Unknown features are fingerprint features in the query molecule, but not found or appearing too infrequently in the training set.

1. All properties and OPS components are within expected ranges.
2. Unknown FCFP_2 feature: 906798516: [*]N([*])C[c](:[*]):[*]

Feature Contribution

Top features for positive contribution				
Fingerprint	Bit/Smiles	Feature Structure	Score	Sensitizer in training set
FCFP_12	675769755	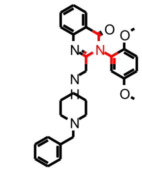 [*]C(=[*])N(C(=[*])[*])[c](:[*]):[*]	0.253	4 out of 4

FCFP_12	580453787	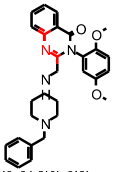 [*]C(=N[c](:[*]):[*])[*]	0.236	3 out of 3
FCFP_12	346218766	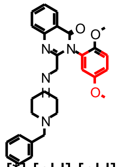 [*][c]1:[*]:[cH]:[cH]:[c](OC):[cH]:1	0.192	36 out of 41

Top Features for negative contribution

Fingerprint	Bit/Smiles	Feature Structure	Score	Sensitizer in training set
FCFP_12	-885550502	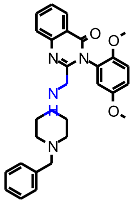 [*]CNC(=[*])[*]	-0.386	8 out of 17
FCFP_12	-1043250487	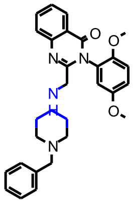 [*]CC(C[*])N[*]	-0.274	14 out of 26
FCFP_12	-1272709286	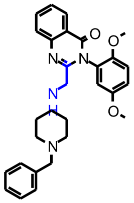 [*]NCC([*])[*]	-0.244	28 out of 50

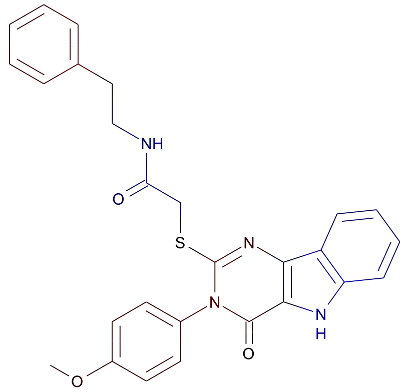

$C_{27}H_{24}N_4O_3S$
Molecular Weight: 484.56946
ALogP: 5.346
Rotatable Bonds: 8
Acceptors: 5
Donors: 2

Model Prediction

Prediction: Non-Sensitizer
Probability: 0.627
Enrichment: 0.914
Bayesian Score: -2.94
Mahalanobis Distance: 8.56
Mahalanobis Distance p-value: 0.007

Prediction: Positive if the Bayesian score is above the estimated best cutoff value from minimizing the false positive and false negative rate.
Probability: The esimated probability that the sample is in the positive category. This assumes that the Bayesian score follows a normal distribution and is different from the prediction using a cutoff.
Enrichment: An estimate of enrichment, that is, the increased likelihood (versus random) of this sample being in the category.
Bayesian Score: The standard Laplacian-modified Bayesian score.
Mahalanobis Distance: The Mahalanobis distance (MD) is the distance to the center of the training data. The larger the MD, the less trustworthy the prediction.
Mahalanobis Distance p-value: The p-value gives the fraction of training data with an MD greater than or equal to the one for the given sample, assuming normally distributed data. The smaller the p-value, the less trustworthy the prediciton. For highly non-normal X properties (e.g., fingerprints), the MD p-value is wildly inaccurate.

Structural Similar Compounds

Name	Tixocortol pivalate	Mometasone furoate	Budesonide
Structure	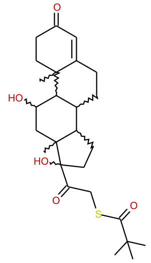	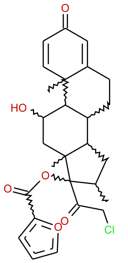	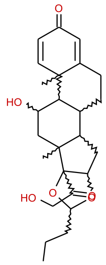
Actual Endpoint	Sensitizer	Sensitizer	Sensitizer
Predicted Endpoint	Sensitizer	Sensitizer	Sensitizer
Distance	0.694	0.711	0.830
Reference	Contact Dermatitis (1996) 34:161	Contact Dermatitis (1996) 34:161	Contact Dermatitis (1996) 34:161

Model Applicability

Unknown features are fingerprint features in the query molecule, but not found or appearing too infrequently in the training set.

1. All properties and OPS components are within expected ranges.
2. Unknown FCFP_2 feature: 203707511: [*]C(=[*])[c]1:[nH]:[*]:[*]:[c]:1[*]

Feature Contribution

Top features for positive contribution				
**Fingerprint**	**Bit/Smiles**	**Feature Structure**	**Score**	**Sensitizer in training set**
FCFP_12	-497728148	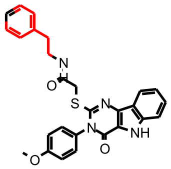 [*]CC[c]1:[cH]:[cH]:[*]:[cH]:[cH]:1	0.304	15 out of 15

FCFP_12	-1986158408	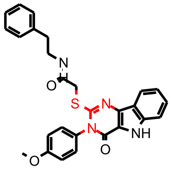<br>[*]S\C(=N\[*])\N([*])<br>[*]	0.286	8 out of 8
FCFP_12	1981711554	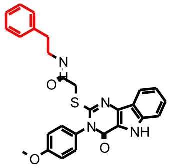<br>[*]CC[c]1:[cH]:[cH]:[<br>cH]:[cH]:[cH]:1	0.274	6 out of 6
**Top Features for negative contribution**				
**Fingerprint**	**Bit/Smiles**	**Feature Structure**	**Score**	**Sensitizer in training set**
FCFP_12	-547731249	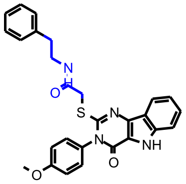<br>[*]CC(=O)NCC([*])[*]	-1.36	0 out of 4
FCFP_12	-1512836998	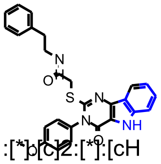<br>[*]1:[*]:[c]2:[*]:[cH<br>]:[cH]:[cH]:[c]:2:[n<br>H]:1	-0.542	0 out of 1
FCFP_12	-1192617147	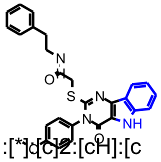<br>[*]1:[*]:[c]2:[cH]:[c<br>H]:[cH]:[cH]:[c]:2:[<br>nH]:1	-0.542	0 out of 1

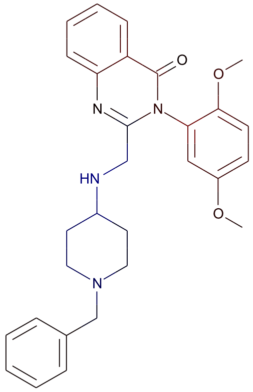

$C_{29}H_{32}N_4O_3$
Molecular Weight: 484.58938
ALogP: 3.743
Rotatable Bonds: 8
Acceptors: 6
Donors: 1

Structural Similar Compounds

Name	Mometasone furoate	Tixocortol pivalate	1;4-bis(2;2;2-Trichloro-1-formamidoethyl)piperazine
Structure	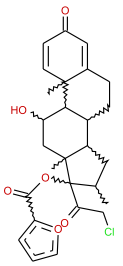	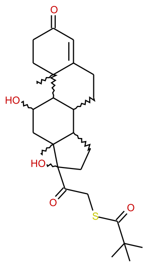	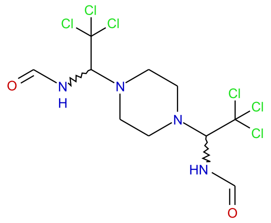
Actual Endpoint	Strong-Sensitizer	Strong-Sensitizer	Weak-Sensitizer
Predicted Endpoint	Weak-Sensitizer	Weak-Sensitizer	Weak-Sensitizer
Distance	0.704	0.761	0.804
Reference	Contact Dermatitis (1996) 34:161	Contact Dermatitis (1996) 34:161	Contact Dermatitis (1994) 31:140

Model Prediction

Prediction: Strong-Sensitizer
Probability: 0.887
Enrichment: 1.14
Bayesian Score: -0.461
Mahalanobis Distance: 9.68
Mahalanobis Distance p-value: 1.09e-005

Prediction: Positive if the Bayesian score is above the estimated best cutoff value from minimizing the false positive and false negative rate.
Probability: The esimated probability that the sample is in the positive category. This assumes that the Bayesian score follows a normal distribution and is different from the prediction using a cutoff.
Enrichment: An estimate of enrichment, that is, the increased likelihood (versus random) of this sample being in the category.
Bayesian Score: The standard Laplacian-modified Bayesian score.
Mahalanobis Distance: The Mahalanobis distance (MD) is the distance to the center of the training data. The larger the MD, the less trustworthy the prediction.
Mahalanobis Distance p-value: The p-value gives the fraction of training data with an MD greater than or equal to the one for the given sample, assuming normally distributed data. The smaller the p-value, the less trustworthy the prediciton. For highly non-normal X properties (e.g., fingerprints), the MD p-value is wildly inaccurate.

Model Applicability

Unknown features are fingerprint features in the query molecule, but not found or appearing too infrequently in the training set.

1. All properties and OPS components are within expected ranges.
2. Unknown FCFP_2 feature: 906798516: [*]N([*])C[c](:[*]):[*]

Feature Contribution

Top features for positive contribution

Fingerprint	Bit/Smiles	Feature Structure	Score	Strong-Sensitizer in training set
FCFP_12	16	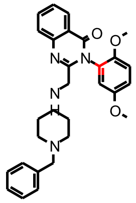 [*][c](:[*]):[*]	0.232	165 out of 165

FCFP_12	1618154665	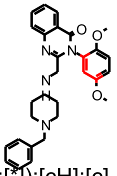 [*][c](:[*]):[cH]:[c]([*]):[*]	0.232	164 out of 164
FCFP_12	203677720	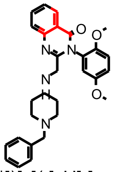 [*]C(=[*])[c](:[cH]:[*]):[c]([*]):[*]	0.232	139 out of 139
**Top Features for negative contribution**				
**Fingerprint**	**Bit/Smiles**	**Feature Structure**	**Score**	**Strong-Sensitizer in training set**
FCFP_12	-885550502	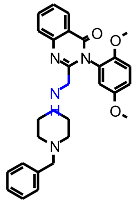 [*]CNC([*])[*]	-0.997	2 out of 9
FCFP_12	-587569116	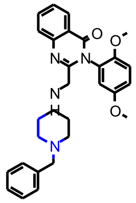 [*]CCN([*])[*]	-0.734	1 out of 4
FCFP_12	-1272709286	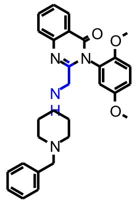 [*]NCC(=[*])[*]	-0.674	10 out of 26

Molecule

TOPKAT_Weight_of_Evidence_Rodent_Carcinogenicity

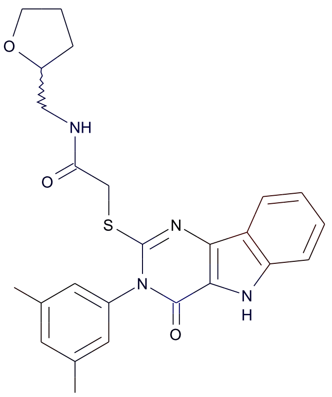

$C_{25}H_{26}N_4O_3S$
Molecular Weight: 462.56393
ALogP: 4.789
Rotatable Bonds: 6
Acceptors: 5
Donors: 2

Model Prediction

Prediction: Non-Carcinogen
Probability: 0.432
Enrichment: 0.84
Bayesian Score: -2.8
Mahalanobis Distance: 9.71
Mahalanobis Distance p-value: 0.000352

Prediction: Positive if the Bayesian score is above the estimated best cutoff value from minimizing the false positive and false negative rate.
Probability: The esimated probability that the sample is in the positive category. This assumes that the Bayesian score follows a normal distribution and is different from the prediction using a cutoff.
Enrichment: An estimate of enrichment, that is, the increased likelihood (versus random) of this sample being in the category.
Bayesian Score: The standard Laplacian-modified Bayesian score.
Mahalanobis Distance: The Mahalanobis distance (MD) is the distance to the center of the training data. The larger the MD, the less trustworthy the prediction.
Mahalanobis Distance p-value: The p-value gives the fraction of training data with an MD greater than or equal to the one for the given sample, assuming normally distributed data. The smaller the p-value, the less trustworthy the prediciton. For highly non-normal X properties (e.g., fingerprints), the MD p-value is wildly inaccurate.

Structural Similar Compounds

Name	Bicalutamide	Glimepride	Glyburide
Structure	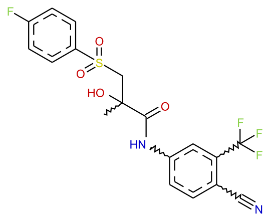	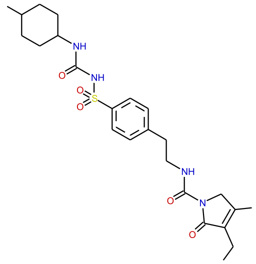	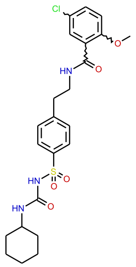
Actual Endpoint	Carcinogen	Carcinogen	Non-Carcinogen
Predicted Endpoint	Carcinogen	Non-Carcinogen	Non-Carcinogen
Distance	0.653	0.666	0.673
Reference	US FDA (Centre for Drug Eval.& Res./Off. Testing & Res.) Sept. 1997	US FDA (Centre for Drug Eval.& Res./Off. Testing & Res.) Sept. 1997	US FDA (Centre for Drug Eval.& Res./Off. Testing & Res.) Sept. 1997

Model Applicability

Unknown features are fingerprint features in the query molecule, but not found or appearing too infrequently in the training set.

1. All properties and OPS components are within expected ranges.

Feature Contribution

Top features for positive contribution				
**Fingerprint**	**Bit/Smiles**	**Feature Structure**	**Score**	**Carcinogen in training set**
SCFP_8	-347281112	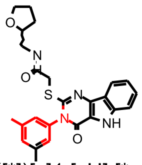 [*]N([*])[c]1:[cH]:[*]:[cH]:[c](C):[cH]:1	0.428	2 out of 2

SCFP_8	1651620003	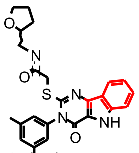 [*][c]1:[*]:[*]:[c]2:[cH]:[cH]:[cH]:[cH]:[c]:1:2	0.386	11 out of 15
SCFP_8	1851000357	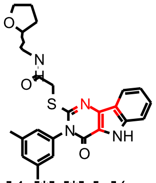 [*][c]1:[*]:[*]:[c](:[*]):[c]:1N=[*]	0.365	8 out of 11
**Top Features for negative contribution**				
**Fingerprint**	**Bit/Smiles**	**Feature Structure**	**Score**	**Carcinogen in training set**
SCFP_8	-2056510245	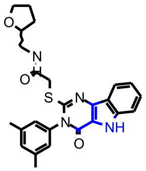 [*]C(=[*])[c]1:[nH]:[*]:[*]:[c]:1[*]	-0.651	4 out of 18
SCFP_8	1257084377	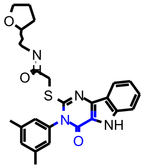 [*]N([*])C(=O)[c](:[*]):[*]	-0.443	7 out of 24
SCFP_8	528802270	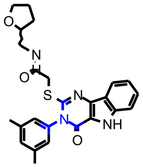 [*]C(=[*])N(C(=[*])[*])[c](:[*]):[*]	-0.39	0 out of 1

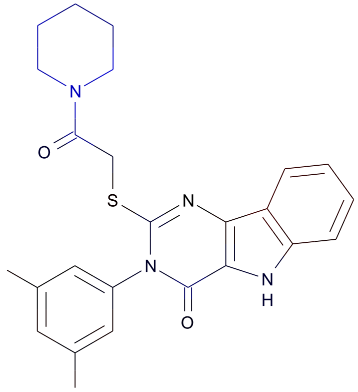

$C_{25}H_{26}N_4O_2S$

Molecular Weight: 446.56453

ALogP: 5.553

Rotatable Bonds: 4

Acceptors: 4

Donors: 1

Structural Similar Compounds

Name	Indomethacin	Ethynodiol	Simvastatin
Structure	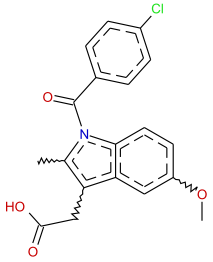	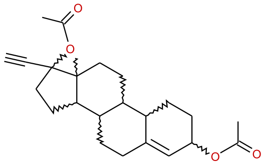	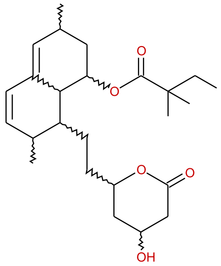
Actual Endpoint	Non-Carcinogen	Carcinogen	Carcinogen
Predicted Endpoint	Non-Carcinogen	Carcinogen	Carcinogen
Distance	0.659	0.702	0.713
Reference	US FDA (Centre for Drug Eval.& Res./Off. Testing & Res.) Sept. 1997	US FDA (Centre for Drug Eval.& Res./Off. Testing & Res.) Sept. 1997	US FDA (Centre for Drug Eval.& Res./Off. Testing & Res.) Sept. 1997

Model Prediction

Prediction: Non-Carcinogen

Probability: 0.356

Enrichment: 0.692

Bayesian Score: -5.16

Mahalanobis Distance: 8.78

Mahalanobis Distance p-value: 0.0105

Prediction: Positive if the Bayesian score is above the estimated best cutoff value from minimizing the false positive and false negative rate.
Probability: The esimated probability that the sample is in the positive category. This assumes that the Bayesian score follows a normal distribution and is different from the prediction using a cutoff.
Enrichment: An estimate of enrichment, that is, the increased likelihood (versus random) of this sample being in the category.
Bayesian Score: The standard Laplacian-modified Bayesian score.
Mahalanobis Distance: The Mahalanobis distance (MD) is the distance to the center of the training data. The larger the MD, the less trustworthy the prediction.
Mahalanobis Distance p-value: The p-value gives the fraction of training data with an MD greater than or equal to the one for the given sample, assuming normally distributed data. The smaller the p-value, the less trustworthy the prediciton. For highly non-normal X properties (e.g., fingerprints), the MD p-value is wildly inaccurate.

Model Applicability

Unknown features are fingerprint features in the query molecule, but not found or appearing too infrequently in the training set.

1. All properties and OPS components are within expected ranges.

Feature Contribution

Top features for positive contribution

Fingerprint	Bit/Smiles	Feature Structure	Score	Carcinogen in training set
SCFP_8	-347281112	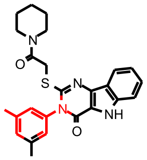 [*]N([*])[c]1:[cH]:[*]:[cH]:[c](C):[cH]:1	0.428	2 out of 2

SCFP_8	1651620003	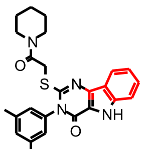 [*][c]1:[*]:[*]:[c]2:[cH]:[cH]:[cH]:[cH]:[c]:1:2	0.386	11 out of 15
SCFP_8	1851000357	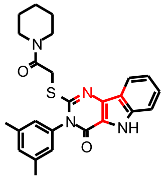 [*][c]1:[*]:[*]:[c](:[*]):[c]:1N=[*]	0.365	8 out of 11

Top Features for negative contribution

Fingerprint	Bit/Smiles	Feature Structure	Score	Carcinogen in training set
SCFP_8	-2103400817	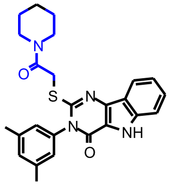 [*]CC(=O)N1CC[*]CC1	-0.889	0 out of 3
SCFP_8	306578635	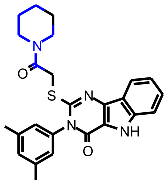 [*]C(=[*])N1C[*]CCC1	-0.889	0 out of 3
SCFP_8	240509252	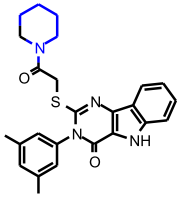 [*]N1[*]CCCC1	-0.879	1 out of 8

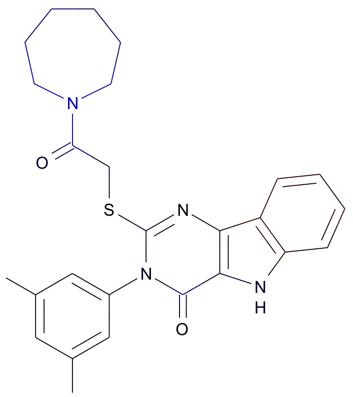

$C_{26}H_{28}N_4O_2S$
Molecular Weight: 460.59111
ALogP: 6.009
Rotatable Bonds: 4
Acceptors: 4
Donors: 1

Model Prediction

Prediction: Non-Carcinogen
Probability: 0.356
Enrichment: 0.692
Bayesian Score: -5.16
Mahalanobis Distance: 8.88
Mahalanobis Distance p-value: 0.0076

Prediction: Positive if the Bayesian score is above the estimated best cutoff value from minimizing the false positive and false negative rate.
Probability: The esimated probability that the sample is in the positive category. This assumes that the Bayesian score follows a normal distribution and is different from the prediction using a cutoff.
Enrichment: An estimate of enrichment, that is, the increased likelihood (versus random) of this sample being in the category.
Bayesian Score: The standard Laplacian-modified Bayesian score.
Mahalanobis Distance: The Mahalanobis distance (MD) is the distance to the center of the training data. The larger the MD, the less trustworthy the prediction.
Mahalanobis Distance p-value: The p-value gives the fraction of training data with an MD greater than or equal to the one for the given sample, assuming normally distributed data. The smaller the p-value, the less trustworthy the prediciton. For highly non-normal X properties (e.g., fingerprints), the MD p-value is wildly inaccurate.

Structural Similar Compounds

Name	Indomethacin	Ethynodiol	Simvastatin
Structure	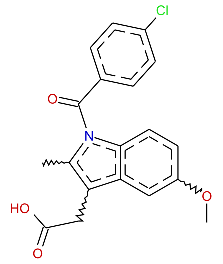	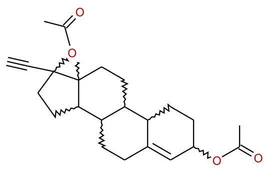	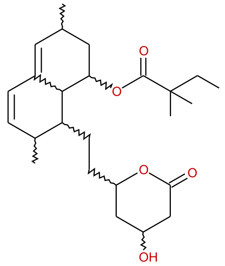
Actual Endpoint	Non-Carcinogen	Carcinogen	Carcinogen
Predicted Endpoint	Non-Carcinogen	Carcinogen	Carcinogen
Distance	0.706	0.726	0.736
Reference	US FDA (Centre for Drug Eval.& Res./Off. Testing & Res.) Sept. 1997	US FDA (Centre for Drug Eval.& Res./Off. Testing & Res.) Sept. 1997	US FDA (Centre for Drug Eval.& Res./Off. Testing & Res.) Sept. 1997

Model Applicability

Unknown features are fingerprint features in the query molecule, but not found or appearing too infrequently in the training set.

1. All properties and OPS components are within expected ranges.

Feature Contribution

Top features for positive contribution

Fingerprint	Bit/Smiles	Feature Structure	Score	Carcinogen in training set
SCFP_8	-347281112	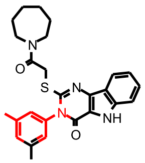 [*]N([*])[c]1:[cH]:[*]:[cH]:[c](C):[cH]:1	0.428	2 out of 2

SCFP_8	1651620003	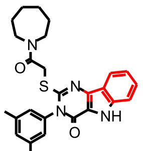 [*][c]1:[*]:[*]:[c]2:[cH]:[cH]:[cH]:[cH]:[c]:1:2	0.386	11 out of 15
SCFP_8	1851000357	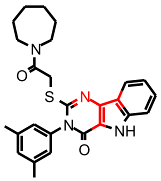 [*][c]1:[*]:[*]:[c](:[*]):[c]:1N=[*]	0.365	8 out of 11

Top Features for negative contribution

Fingerprint	Bit/Smiles	Feature Structure	Score	Carcinogen in training set
SCFP_8	-2103400817	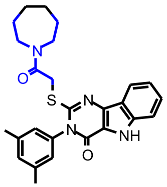 [*]CC(=O)N1CC[*]CC1	-0.889	0 out of 3
SCFP_8	306578635	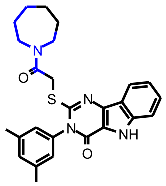 [*]C(=[*])N1C[*]CCC1	-0.889	0 out of 3
SCFP_8	240509252	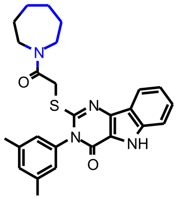 [*]N1[*]CCCC1	-0.879	1 out of 8

#UNDEFINED

TOPKAT_Weight_of_Evidence_Rodent_Carcinogenicity

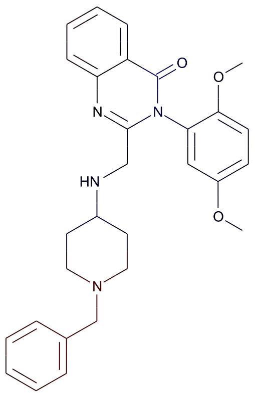

$C_{29}H_{32}N_4O_3$

Molecular Weight: 484.58938

ALogP: 3.743

Rotatable Bonds: 8

Acceptors: 6

Donors: 1

Model Prediction

Prediction: Non-Carcinogen

Probability: 0.469

Enrichment: 0.912

Bayesian Score: -1.8

Mahalanobis Distance: 10.6

Mahalanobis Distance p-value: 4.81e-006

Prediction: Positive if the Bayesian score is above the estimated best cutoff value from minimizing the false positive and false negative rate.
Probability: The esimated probability that the sample is in the positive category. This assumes that the Bayesian score follows a normal distribution and is different from the prediction using a cutoff.
Enrichment: An estimate of enrichment, that is, the increased likelihood (versus random) of this sample being in the category.
Bayesian Score: The standard Laplacian-modified Bayesian score.
Mahalanobis Distance: The Mahalanobis distance (MD) is the distance to the center of the training data. The larger the MD, the less trustworthy the prediction.
Mahalanobis Distance p-value: The p-value gives the fraction of training data with an MD greater than or equal to the one for the given sample, assuming normally distributed data. The smaller the p-value, the less trustworthy the prediciton. For highly non-normal X properties (e.g., fingerprints), the MD p-value is wildly inaccurate.

Structural Similar Compounds

Name	Emetine	Felodipine	Cisapride
Structure	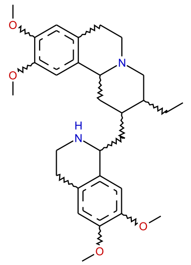	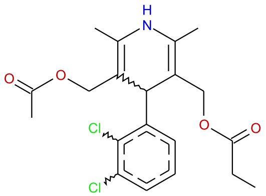	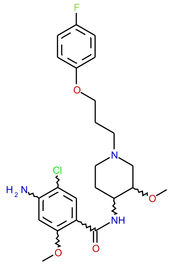
Actual Endpoint	Non-Carcinogen	Non-Carcinogen	Non-Carcinogen
Predicted Endpoint	Non-Carcinogen	Non-Carcinogen	Non-Carcinogen
Distance	0.588	0.619	0.637
Reference	US FDA (Centre for Drug Eval.& Res./Off. Testing & Res.) Sept. 1997	US FDA (Centre for Drug Eval.& Res./Off. Testing & Res.) Sept. 1997	US FDA (Centre for Drug Eval.& Res./Off. Testing & Res.) Sept. 1997

Model Applicability

Unknown features are fingerprint features in the query molecule, but not found or appearing too infrequently in the training set.

1. OPS PC13 out of range. Value: -3.0317. Training min, max, SD, explained variance: -2.3055, 3.3147, 1.058, 0.0228.

Feature Contribution

Top features for positive contribution

Fingerprint	Bit/Smiles	Feature Structure	Score	Carcinogen in training set
SCFP_8	-205766035	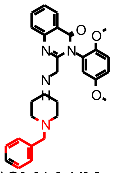 [*]N([*])C[c]1:[cH]:[cH]:[*]:[cH]:[cH]:1	0.498	3 out of 3

SCFP_8	-1377141613	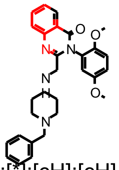 [*][c]1:[*]:[cH]:[cH]:[cH]:[c]:1N=[*]	0.444	8 out of 10
SCFP_8	1274421524	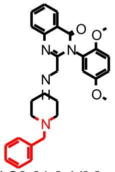 [*]N([*])C[c]1:[cH]:[cH]:[cH]:[cH]:[cH]:1	0.428	2 out of 2
**Top Features for negative contribution**				
**Fingerprint**	**Bit/Smiles**	**Feature Structure**	**Score**	**Carcinogen in training set**
SCFP_8	1257084377	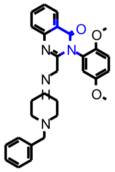 [*]N([*])C(=O)[c](:[*]):[*]	-0.443	7 out of 24
SCFP_8	-627385064	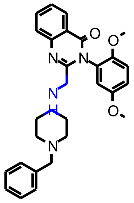 [*]CNC([*])[*]	-0.43	13 out of 43
SCFP_8	-1430588017	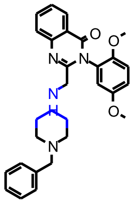 [*]CC(C[*])N[*]	-0.392	10 out of 32

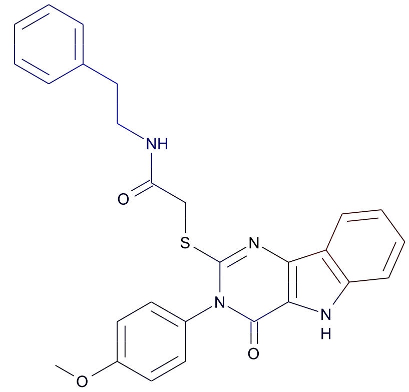

$C_{27}H_{24}N_4O_3S$
Molecular Weight: 484.56946
ALogP: 5.346
Rotatable Bonds: 8
Acceptors: 5
Donors: 2

Model Prediction

Prediction: Non-Carcinogen
Probability: 0.341
Enrichment: 0.663
Bayesian Score: -5.7
Mahalanobis Distance: 8.9
Mahalanobis Distance p-value: 0.00726

Prediction: Positive if the Bayesian score is above the estimated best cutoff value from minimizing the false positive and false negative rate.
Probability: The esimated probability that the sample is in the positive category. This assumes that the Bayesian score follows a normal distribution and is different from the prediction using a cutoff.
Enrichment: An estimate of enrichment, that is, the increased likelihood (versus random) of this sample being in the category.
Bayesian Score: The standard Laplacian-modified Bayesian score.
Mahalanobis Distance: The Mahalanobis distance (MD) is the distance to the center of the training data. The larger the MD, the less trustworthy the prediction.
Mahalanobis Distance p-value: The p-value gives the fraction of training data with an MD greater than or equal to the one for the given sample, assuming normally distributed data. The smaller the p-value, the less trustworthy the prediciton. For highly non-normal X properties (e.g., fingerprints), the MD p-value is wildly inaccurate.

Structural Similar Compounds

Name	Glyburide	Bitolterol	Glimepride
Structure	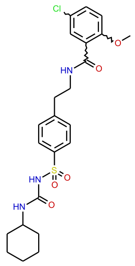	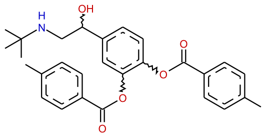	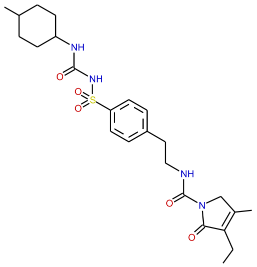
Actual Endpoint	Non-Carcinogen	Non-Carcinogen	Carcinogen
Predicted Endpoint	Non-Carcinogen	Non-Carcinogen	Non-Carcinogen
Distance	0.620	0.639	0.674
Reference	US FDA (Centre for Drug Eval.& Res./Off. Testing & Res.) Sept. 1997	US FDA (Centre for Drug Eval.& Res./Off. Testing & Res.) Sept. 1997	US FDA (Centre for Drug Eval.& Res./Off. Testing & Res.) Sept. 1997

Model Applicability

Unknown features are fingerprint features in the query molecule, but not found or appearing too infrequently in the training set.

1. All properties and OPS components are within expected ranges.

Feature Contribution

Top features for positive contribution				
**Fingerprint**	**Bit/Smiles**	**Feature Structure**	**Score**	**Carcinogen in training set**
SCFP_8	1651620003	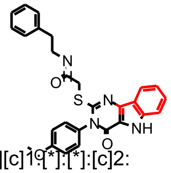 [*][c]1c[*]:[*]:[c]2:[cH]:[cH]:[cH]:[cH]:[c]:1:2	0.386	11 out of 15

SCFP_8	1851000357	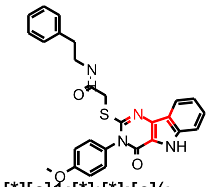 [*][c]1:[*]:[*]:[c](:[*]):[c]:1N=[*]	0.365	8 out of 11
SCFP_8	1205734667	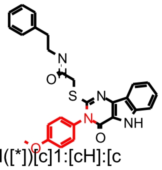 [*]N([*])[c]1:[cH]:[cH]:[c](OC):[cH]:[cH]:1	0.303	1 out of 1
**Top Features for negative contribution**				
**Fingerprint**	**Bit/Smiles**	**Feature Structure**	**Score**	**Carcinogen in training set**
SCFP_8	-1211866396	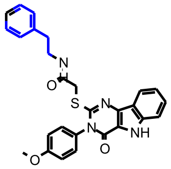 [*]CC[c]1:[cH]:[cH]:[*]:[cH]:[cH]:1	-0.685	6 out of 27
SCFP_8	-2056510245	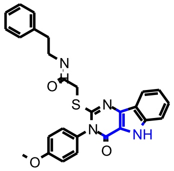 [*]C(=[*])[c]1:[nH]:[*]:[*]:[c]:1[*]	-0.651	4 out of 18
SCFP_8	1257084377	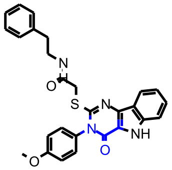 [*]N([*])C(=O)[c](:[*]):[*]	-0.443	7 out of 24

Molecule

TOPKAT_Carcinogenic_Potency_TD50_Mouse

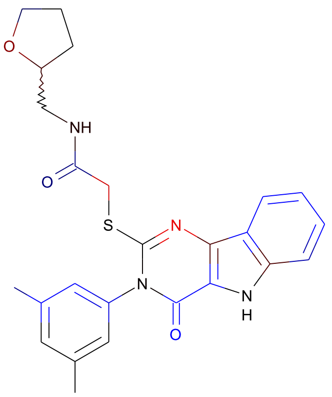

$C_{25}H_{26}N_4O_3S$
Molecular Weight: 462.56393
ALogP: 4.789
Rotatable Bonds: 6
Acceptors: 5
Donors: 2

Model Prediction

Prediction: 12
Unit: mg/kg_body_weight/day
Mahalanobis Distance: 12.8
Mahalanobis Distance p-value: 3.32e-007

Mahalanobis Distance: The Mahalanobis distance (MD) is a generalization of the Euclidean distance that accounts for correlations among the X properties. It is calculated as the distance to the center of the training data. The larger the MD, the less trustworthy the prediction.
Mahalanobis Distance p-value: The p-value gives the fraction of training data with an MD greater than or equal to the one for the given sample, assuming normally distributed data. The smaller the p-value, the less trustworthy the prediciton. For highly non-normal X properties (e.g., fingerprints), the MD p-value is wildly inaccurate.

Structural Similar Compounds

Name	Ochratoxin A	542	Phenolphthalein
Structure	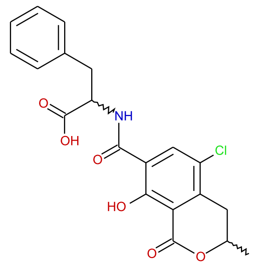	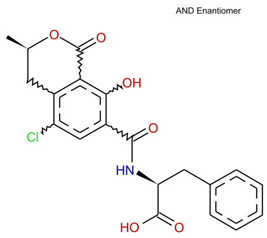	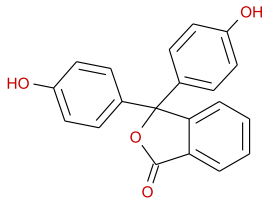
Actual Endpoint (-log C)	4.79932	4.79932	2.43468
Predicted Endpoint (-log C)	3.6353	3.6353	3.66084
Distance	0.795	0.795	0.798
Reference	CPDB	CPDB	CPDB

Model Applicability

Unknown features are fingerprint features in the query molecule, but not found or appearing too infrequently in the training set.

1. OPS PC23 out of range. Value: 3.3852. Training min, max, SD, explained variance: -2.6901, 3.3252, 1.05, 0.0138.
2. Unknown ECFP_2 feature: -782828288: [*]C(=[*])[c]1:[nH]:[*]:[*]:[c]:1[*]
3. Unknown ECFP_2 feature: -962771238: [*]C(=[*])N(C(=[*])[*])[c](:[*]):[*]
4. Unknown ECFP_2 feature: -962137479: [*][c]1:[*]:[*]:[c](:[*]):[c]:1N=[*]
5. Unknown ECFP_2 feature: 676970202: [*]S\C(=N\[*])\N([*])[*]
6. Unknown ECFP_2 feature: -84772164: [*]NCC([*])[*]

Feature Contribution

Top features for positive contribution

Fingerprint	Bit/Smiles	Feature Structure	Score
ECFP_6	655739385	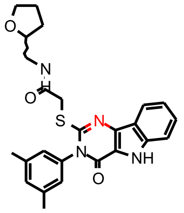 [*]N=[*]	0.229

ECFP_6	1559650422	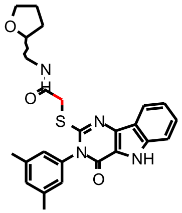 [*]C[*]	0.203
ECFP_6	683445015	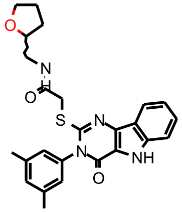 [*]O[*]	0.136
**Top Features for negative contribution**			
**Fingerprint**	**Bit/Smiles**	**Feature Structure**	**Score**
ECFP_6	2106656448	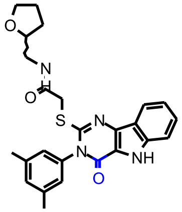 [*]C(=O)[*]	-0.275
ECFP_6	1996767644	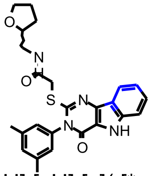 [*]:[cH]:[cH]:[c](:[*]):[*]	-0.251
ECFP_6	642810091	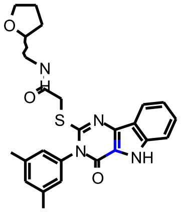 [*][c](:[*]):[*]	-0.247

#UNDEFINED

TOPKAT_Carcinogenic_Potency_TD50_Mouse

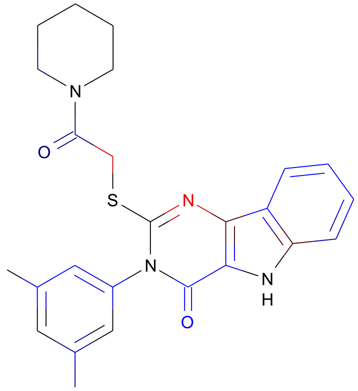

$C_{25}H_{26}N_4O_2S$
Molecular Weight: 446.56453
ALogP: 5.553
Rotatable Bonds: 4
Acceptors: 4
Donors: 1

Model Prediction

Prediction: 18.8
Unit: mg/kg_body_weight/day
Mahalanobis Distance: 12.9
Mahalanobis Distance p-value: 2.51e-007

Mahalanobis Distance: The Mahalanobis distance (MD) is a generalization of the Euclidean distance that accounts for correlations among the X properties. It is calculated as the distance to the center of the training data. The larger the MD, the less trustworthy the prediction.
Mahalanobis Distance p-value: The p-value gives the fraction of training data with an MD greater than or equal to the one for the given sample, assuming normally distributed data. The smaller the p-value, the less trustworthy the prediciton. For highly non-normal X properties (e.g., fingerprints), the MD p-value is wildly inaccurate.

Structural Similar Compounds

Name	646	Phenolphthalein	C.I. pigment red 3
Structure	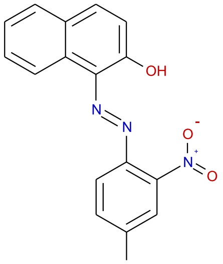	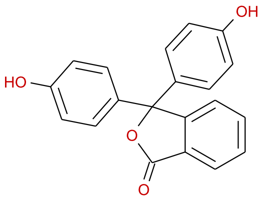	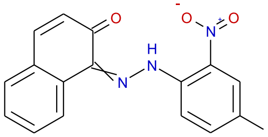
Actual Endpoint (-log C)	0.937339	2.43468	0.937339
Predicted Endpoint (-log C)	3.26294	3.66084	3.17837
Distance	0.745	0.750	0.810
Reference	CPDB	CPDB	CPDB

Model Applicability

Unknown features are fingerprint features in the query molecule, but not found or appearing too infrequently in the training set.

1. All properties and OPS components are within expected ranges.
2. Unknown ECFP_2 feature: -782828288: [*]C(=[*])[c]1:[nH]:[*]:[*]:[c]:1[*]
3. Unknown ECFP_2 feature: -962771238: [*]C(=[*])N(C(=[*])[*])[c](:[*]):[*]
4. Unknown ECFP_2 feature: -962137479: [*][c]1:[*]:[*]:[c](:[*]):[c]:1N=[*]
5. Unknown ECFP_2 feature: 676970202: [*]S\C(=N\[*])\N([*])[*]
6. Unknown ECFP_2 feature: 1341750291: [*]CC(=O)N([*])[*]
7. Unknown ECFP_2 feature: -1102925512: [*]CN(C[*])C(=[*])[*]

Feature Contribution

Top features for positive contribution			
**Fingerprint**	**Bit/Smiles**	**Feature Structure**	**Score**

ECFP_6	655739385	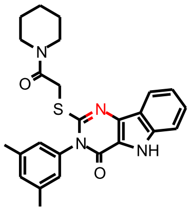 [*]N=[*]	0.229
ECFP_6	1559650422	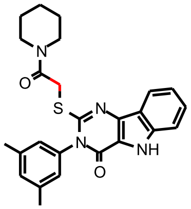 [*]C[*]	0.203
ECFP_6	1333660716	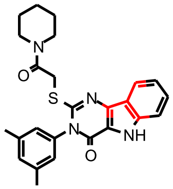 [*][c]1:[*]:[*]:[c](:[*]):[c]:1:[cH]:[*]	0.0746
**Top Features for negative contribution**			
**Fingerprint**	**Bit/Smiles**	**Feature Structure**	**Score**
ECFP_6	2106656448	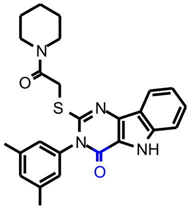 [*]C(=O)[*]	-0.275
ECFP_6	1996767644	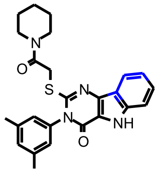 [*]:[cH]:[cH]:[c](:[*]):[*]	-0.251

ECFP_6	642810091	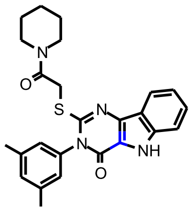<br>[*][c](:[*]):[*]	-0.247

#UNDEFINED

TOPKAT_Carcinogenic_Potency_TD50_Mouse

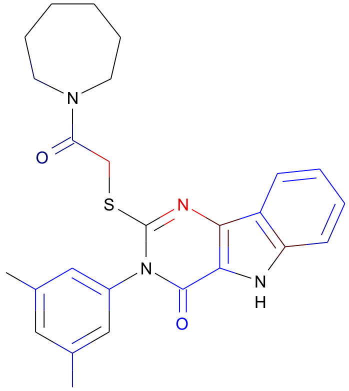

$C_{26}H_{28}N_4O_2S$

Molecular Weight: 460.59111

ALogP: 6.009

Rotatable Bonds: 4

Acceptors: 4

Donors: 1

Model Prediction

Prediction: 16.4

Unit: mg/kg_body_weight/day

Mahalanobis Distance: 13

Mahalanobis Distance p-value: 1.41e-007

Mahalanobis Distance: The Mahalanobis distance (MD) is a generalization of the Euclidean distance that accounts for correlations among the X properties. It is calculated as the distance to the center of the training data. The larger the MD, the less trustworthy the prediction.

Mahalanobis Distance p-value: The p-value gives the fraction of training data with an MD greater than or equal to the one for the given sample, assuming normally distributed data. The smaller the p-value, the less trustworthy the prediciton. For highly non-normal X properties (e.g., fingerprints), the MD p-value is wildly inaccurate.

Structural Similar Compounds

Name	646	Phenolphthalein	C.I. pigment red 3
Structure	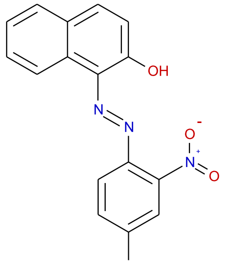	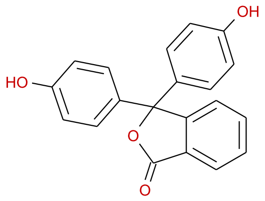	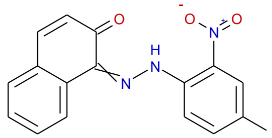
Actual Endpoint (-log C)	0.937339	2.43468	0.937339
Predicted Endpoint (-log C)	3.26294	3.66084	3.17837
Distance	0.758	0.772	0.829
Reference	CPDB	CPDB	CPDB

Model Applicability

Unknown features are fingerprint features in the query molecule, but not found or appearing too infrequently in the training set.

1. All properties and OPS components are within expected ranges.
2. Unknown ECFP_2 feature: -782828288: [*]C(=[*])[c]1:[nH]:[*]:[*]:[c]:1[*]
3. Unknown ECFP_2 feature: -962771238: [*]C(=[*])N(C(=[*])[*])[c](:[*]):[*]
4. Unknown ECFP_2 feature: -962137479: [*][c]1:[*]:[*]:[c](:[*]):[c]:1N=[*]
5. Unknown ECFP_2 feature: 676970202: [*]S\C(=N\[*])\N([*])[*]
6. Unknown ECFP_2 feature: 1341750291: [*]CC(=O)N([*])[*]
7. Unknown ECFP_2 feature: -1102925512: [*]CN(C[*])C(=[*])[*]

Feature Contribution

Top features for positive contribution

Fingerprint	Bit/Smiles	Feature Structure	Score

ECFP_6	655739385	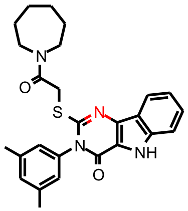 [*]N=[*]	0.229
ECFP_6	1559650422	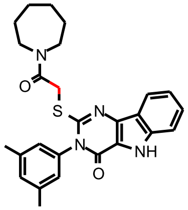 [*]C[*]	0.203
ECFP_6	1333660716	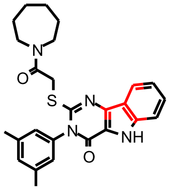 [*][c]1:[*]:[*]:[c](:[*]):[c]:1:[cH]:[*]	0.0746

Top Features for negative contribution

Fingerprint	Bit/Smiles	Feature Structure	Score
ECFP_6	2106656448	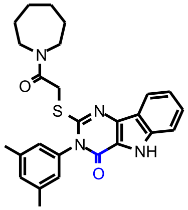 [*]C(=O)[*]	-0.275
ECFP_6	1996767644	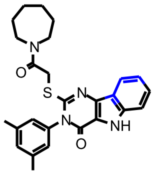 [*]:[cH]:[cH]:[c](:[*]):[*]	-0.251

ECFP_6	642810091	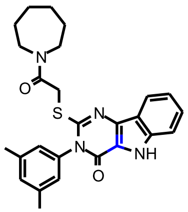<br>[*][c](:[*]):[*]	-0.247

#UNDEFINED

TOPKAT_Carcinogenic_Potency_TD50_Mouse

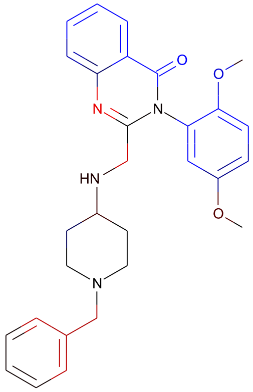

$C_{29}H_{32}N_4O_3$

Molecular Weight: 484.58938

ALogP: 3.743

Rotatable Bonds: 8

Acceptors: 6

Donors: 1

Model Prediction

Prediction: 17.8

Unit: mg/kg_body_weight/day

Mahalanobis Distance: 13.1

Mahalanobis Distance p-value: 5.59e-008

Mahalanobis Distance: The Mahalanobis distance (MD) is a generalization of the Euclidean distance that accounts for correlations among the X properties. It is calculated as the distance to the center of the training data. The larger the MD, the less trustworthy the prediction.

Mahalanobis Distance p-value: The p-value gives the fraction of training data with an MD greater than or equal to the one for the given sample, assuming normally distributed data. The smaller the p-value, the less trustworthy the prediciton. For highly non-normal X properties (e.g., fingerprints), the MD p-value is wildly inaccurate.

Structural Similar Compounds

Name	Estradiol mustard	223	Phenolphthalein
Structure	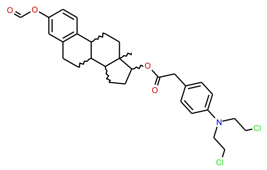	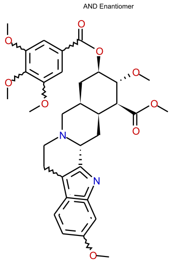	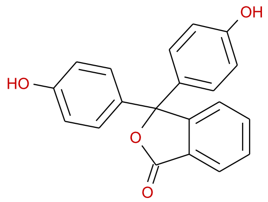
Actual Endpoint (-log C)	5.58568	5.08368	2.43468
Predicted Endpoint (-log C)	5.97715	5.08273	3.66084
Distance	0.827	0.842	0.879
Reference	CPDB	CPDB	CPDB

Model Applicability

Unknown features are fingerprint features in the query molecule, but not found or appearing too infrequently in the training set.

1. OPS PC7 out of range. Value: 6.4578. Training min, max, SD, explained variance: -5.1479, 5.5527, 1.707, 0.0363.
2. OPS PC22 out of range. Value: 4.6832. Training min, max, SD, explained variance: -3.1587, 3.8589, 1.086, 0.0147.
3. Unknown ECFP_2 feature: -962771238: [*]C(=[*])N(C(=[*])[*])[c](:[*]):[*]
4. Unknown ECFP_2 feature: -1073177635: [*]C\C(=N\[*])\N([*])[*]
5. Unknown ECFP_2 feature: -1236953626: [*]N([*])[c](:[cH]:[*]):[c]([*]):[*]
6. Unknown ECFP_2 feature: -597295171: [*][c](:[*]):[c](:[cH]:[*])N=[*]
7. Unknown ECFP_2 feature: 496787418: [*]CNC([*])[*]
8. Unknown ECFP_2 feature: -44121127: [*]N([*])C[c](:[*]):[*]

Feature Contribution

Top features for positive contribution			
**Fingerprint**	**Bit/Smiles**	**Feature Structure**	**Score**

ECFP_6	655739385	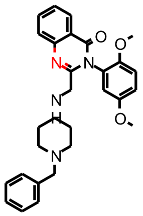 [*]N=[*]	0.229
ECFP_6	1559650422	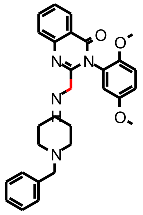 [*]C[*]	0.203
ECFP_6	-2024255407	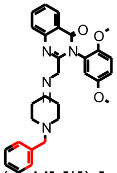 [*]C[c](:[cH]:[*]):[cH]:[*]	0.172
**Top Features for negative contribution**			
**Fingerprint**	**Bit/Smiles**	**Feature Structure**	**Score**
ECFP_6	2106656448	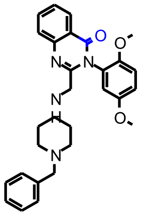 [*]C(=O)[*]	-0.275
ECFP_6	1996767644	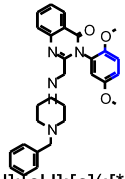 [*]:[cH]:[cH]:[c](:[*]):[*]	-0.251

ECFP_6	642810091	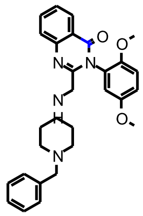 [*][c](:[*]):[*]	-0.247

#UNDEFINED

TOPKAT_Carcinogenic_Potency_TD50_Mouse

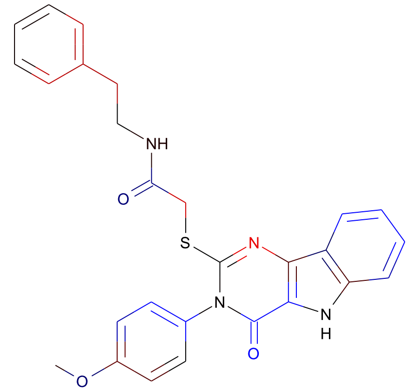

$C_{27}H_{24}N_4O_3S$
Molecular Weight: 484.56946
ALogP: 5.346
Rotatable Bonds: 8
Acceptors: 5
Donors: 2

Model Prediction

Prediction: 5.61
Unit: mg/kg_body_weight/day
Mahalanobis Distance: 15
Mahalanobis Distance p-value: 3.88e-013

Mahalanobis Distance: The Mahalanobis distance (MD) is a generalization of the Euclidean distance that accounts for correlations among the X properties. It is calculated as the distance to the center of the training data. The larger the MD, the less trustworthy the prediction.
Mahalanobis Distance p-value: The p-value gives the fraction of training data with an MD greater than or equal to the one for the given sample, assuming normally distributed data. The smaller the p-value, the less trustworthy the prediciton. For highly non-normal X properties (e.g., fingerprints), the MD p-value is wildly inaccurate.

Structural Similar Compounds

Name	223	Tamoxifen citrate	646
Structure	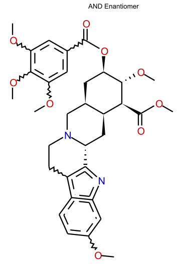	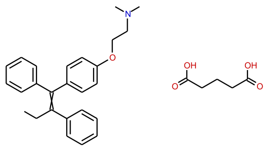	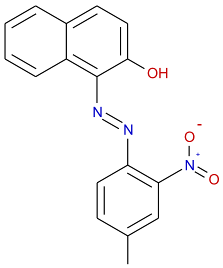
Actual Endpoint (-log C)	5.08368	5.05965	0.937339
Predicted Endpoint (-log C)	5.08273	4.24168	3.26294
Distance	0.896	0.900	0.905
Reference	CPDB	CPDB	CPDB

Model Applicability

Unknown features are fingerprint features in the query molecule, but not found or appearing too infrequently in the training set.

1. All properties and OPS components are within expected ranges.
2. Unknown ECFP_2 feature: -782828288: [*]C(=[*])[c]1:[nH]:[*]:[*]:[c]:1[*]
3. Unknown ECFP_2 feature: -962771238: [*]C(=[*])N(C(=[*])[*])[c](:[*]):[*]
4. Unknown ECFP_2 feature: -962137479: [*][c]1:[*]:[*]:[c](:[*]):[c]:1N=[*]
5. Unknown ECFP_2 feature: 676970202: [*]S\C(=N\[*])\N([*])[*]

Feature Contribution

Top features for positive contribution

Fingerprint	Bit/Smiles	Feature Structure	Score
ECFP_6	655739385	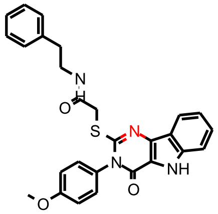 [*]N=[*]	0.229

ECFP_6	1559650422	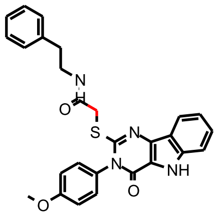 [*]C[*]	0.203
ECFP_6	-2024255407	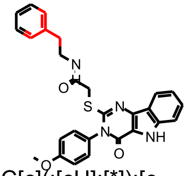 [*]C[c](:[cH]:[*]):[cH]:[*]	0.172
**Top Features for negative contribution**			
**Fingerprint**	**Bit/Smiles**	**Feature Structure**	**Score**
ECFP_6	2106656448	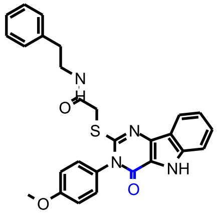 [*]C(=O)[*]	-0.275
ECFP_6	1996767644	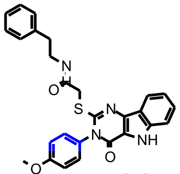 [*]:[cH]:[cH]:[c](:[*]):[*]	-0.251
ECFP_6	642810091	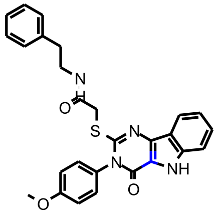 [*][c](:[*]):[*]	-0.247

Molecule

TOPKAT_Carcinogenic_Potency_TD50_Rat

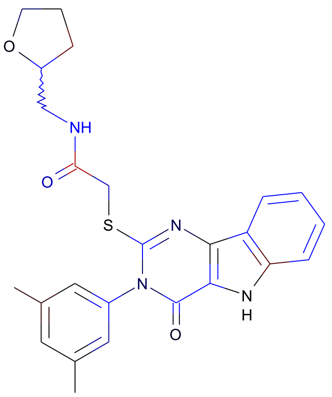

$C_{25}H_{26}N_4O_3S$
Molecular Weight: 462.56393
ALogP: 4.789
Rotatable Bonds: 6
Acceptors: 5
Donors: 2

Model Prediction

Prediction: 13.9
Unit: mg/kg_body_weight/day
Mahalanobis Distance: 17.1
Mahalanobis Distance p-value: 3.59e-018

Mahalanobis Distance: The Mahalanobis distance (MD) is a generalization of the Euclidean distance that accounts for correlations among the X properties. It is calculated as the distance to the center of the training data. The larger the MD, the less trustworthy the prediction.
Mahalanobis Distance p-value: The p-value gives the fraction of training data with an MD greater than or equal to the one for the given sample, assuming normally distributed data. The smaller the p-value, the less trustworthy the prediciton. For highly non-normal X properties (e.g., fingerprints), the MD p-value is wildly inaccurate.

Structural Similar Compounds

Name	Fluvastatin	913	FD & C violet no. 1
Structure	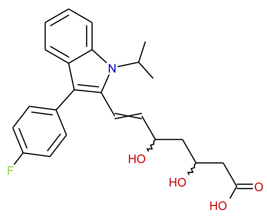	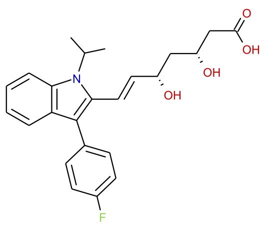	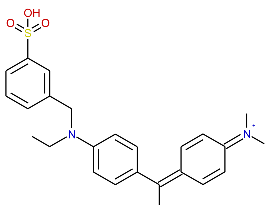
Actual Endpoint (-log C)	3.51742	3.51742	2.8543
Predicted Endpoint (-log C)	5.41573	5.41573	3.40838
Distance	0.747	0.747	0.753
Reference	CPDB	CPDB	CPDB

Model Applicability

Unknown features are fingerprint features in the query molecule, but not found or appearing too infrequently in the training set.

1. All properties and OPS components are within expected ranges.

Feature Contribution

Top features for positive contribution

Fingerprint	Bit/Smiles	Feature Structure	Score
FCFP_6	1	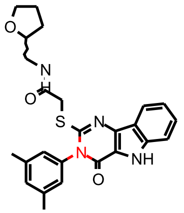 [*]N([*])[*]	0.234

FCFP_6	-885550502	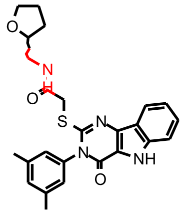 [*]CNC(=[*])[*]	0.229
FCFP_6	203677720	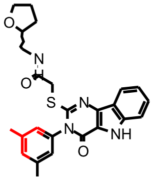 [*]:[cH]:[c](C):[cH]:[*]	0.137
**Top Features for negative contribution**			
**Fingerprint**	**Bit/Smiles**	**Feature Structure**	**Score**
FCFP_6	-1272709286	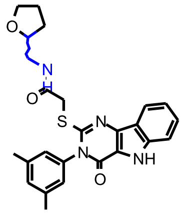 [*]NCC([*])[*]	-0.526
FCFP_6	991735244	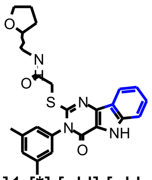 [*]:[c]1:[*]:[cH]:[cH]:[cH]:[cH]:1	-0.422
FCFP_6	16	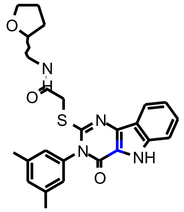 [*][c](:[*]):[*]	-0.354

#UNDEFINED

TOPKAT_Carcinogenic_Potency_TD50_Rat

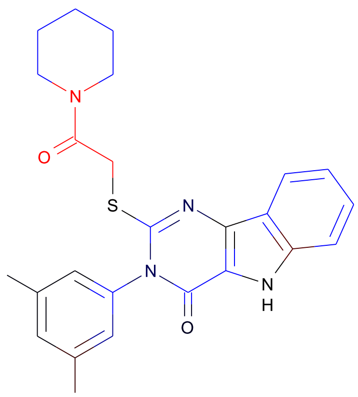

$C_{25}H_{26}N_4O_2S$
Molecular Weight: 446.56453
ALogP: 5.553
Rotatable Bonds: 4
Acceptors: 4
Donors: 1

Model Prediction

Prediction: 9.07
Unit: mg/kg_body_weight/day
Mahalanobis Distance: 16.1
Mahalanobis Distance p-value: 1.66e-014

Mahalanobis Distance: The Mahalanobis distance (MD) is a generalization of the Euclidean distance that accounts for correlations among the X properties. It is calculated as the distance to the center of the training data. The larger the MD, the less trustworthy the prediction.
Mahalanobis Distance p-value: The p-value gives the fraction of training data with an MD greater than or equal to the one for the given sample, assuming normally distributed data. The smaller the p-value, the less trustworthy the prediciton. For highly non-normal X properties (e.g., fingerprints), the MD p-value is wildly inaccurate.

Structural Similar Compounds

Name	FD & C violet no. 1	Indomethacin	646
Structure	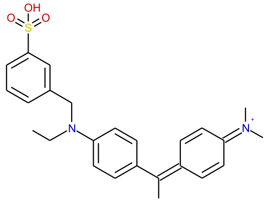	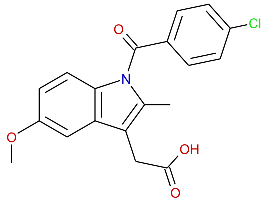	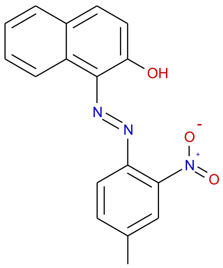
Actual Endpoint (-log C)	2.8543	5.49293	2.41938
Predicted Endpoint (-log C)	3.40838	4.9569	3.77987
Distance	0.714	0.731	0.737
Reference	CPDB	CPDB	CPDB

Model Applicability

Unknown features are fingerprint features in the query molecule, but not found or appearing too infrequently in the training set.

1. All properties and OPS components are within expected ranges.

Feature Contribution

Top features for positive contribution			
**Fingerprint**	**Bit/Smiles**	**Feature Structure**	**Score**
FCFP_6	565998553	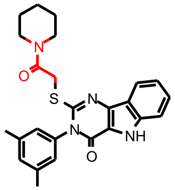 [*]CC(=O)N([*])[*]	0.357

FCFP_6	1	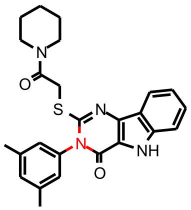 [*]N([*])[*]	0.234
FCFP_6	203677720	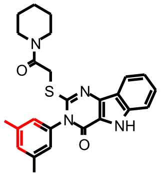 [*]:[cH]:[c](C):[cH]:<br>[*]	0.137
**Top Features for negative contribution**			
**Fingerprint**	**Bit/Smiles**	**Feature Structure**	**Score**
FCFP_6	1175638033	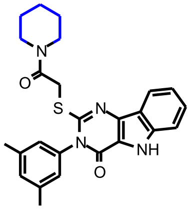 [*]1CCCCC1	-0.512
FCFP_6	991735244	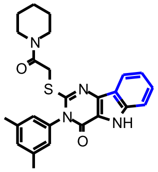 [*]:[c]1:[*]:[cH]:[cH<br>]:[cH]:[cH]:1	-0.422
FCFP_6	16	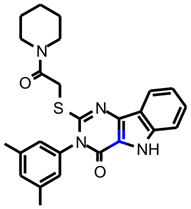 [*][c](:[*]):[*]	-0.354

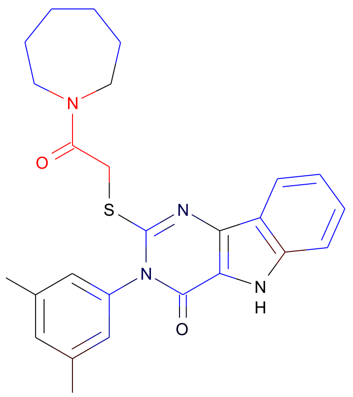

$C_{26}H_{28}N_4O_2S$
Molecular Weight: 460.59111
ALogP: 6.009
Rotatable Bonds: 4
Acceptors: 4
Donors: 1

Model Prediction

Prediction: 8.42
Unit: mg/kg_body_weight/day
Mahalanobis Distance: 16.1
Mahalanobis Distance p-value: 1.79e-014

Mahalanobis Distance: The Mahalanobis distance (MD) is a generalization of the Euclidean distance that accounts for correlations among the X properties. It is calculated as the distance to the center of the training data. The larger the MD, the less trustworthy the prediction.
Mahalanobis Distance p-value: The p-value gives the fraction of training data with an MD greater than or equal to the one for the given sample, assuming normally distributed data. The smaller the p-value, the less trustworthy the prediciton. For highly non-normal X properties (e.g., fingerprints), the MD p-value is wildly inaccurate.

Structural Similar Compounds

Name	FD & C violet no. 1	Indomethacin	646
Structure	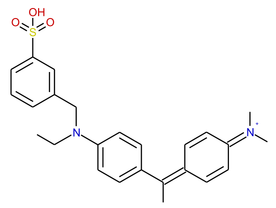	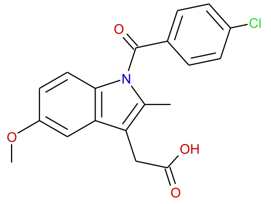	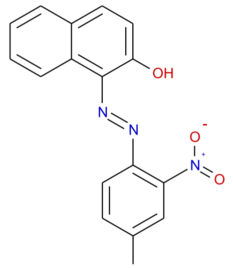
Actual Endpoint (-log C)	2.8543	5.49293	2.41938
Predicted Endpoint (-log C)	3.40838	4.9569	3.77987
Distance	0.722	0.753	0.755
Reference	CPDB	CPDB	CPDB

Model Applicability

Unknown features are fingerprint features in the query molecule, but not found or appearing too infrequently in the training set.

1. All properties and OPS components are within expected ranges.

Feature Contribution

Top features for positive contribution

Fingerprint	Bit/Smiles	Feature Structure	Score
FCFP_6	565998553	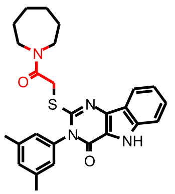 [*]CC(=O)N([*])[*]	0.357

FCFP_6	1	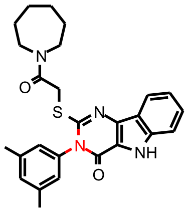 [*]N([*])[*]	0.234
FCFP_6	203677720	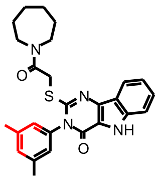 [*]:[cH]:[c](C):[cH]:[*]	0.137

Top Features for negative contribution

Fingerprint	Bit/Smiles	Feature Structure	Score
FCFP_6	1175638033	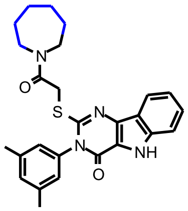 [*]1CCCCC1	-0.512
FCFP_6	991735244	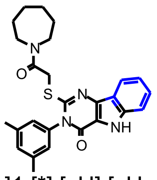 [*]:[c]1:[*]:[cH]:[cH]:[cH]:[cH]:1	-0.422
FCFP_6	16	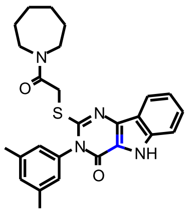 [*][c](:[*]):[*]	-0.354

#UNDEFINED

TOPKAT_Carcinogenic_Potency_TD50_Rat

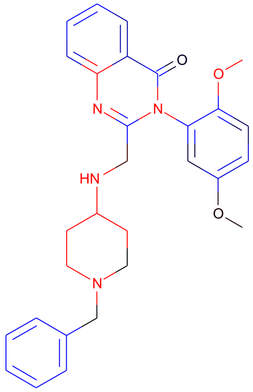

$C_{29}H_{32}N_4O_3$
Molecular Weight: 484.58938
ALogP: 3.743
Rotatable Bonds: 8
Acceptors: 6
Donors: 1

Structural Similar Compounds

Name	Loxtidine	FD & C violet no. 1	5,6-Dimethoxysterigmatocystin
Structure	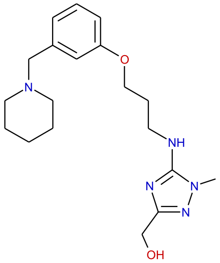	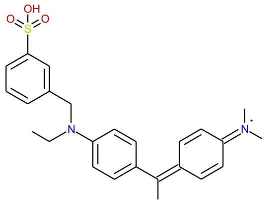	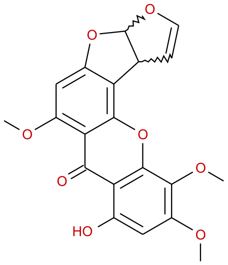
Actual Endpoint (-log C)	2.87532	2.8543	6.02361
Predicted Endpoint (-log C)	3.63996	3.40838	4.98771
Distance	0.747	0.777	0.792
Reference	CPDB	CPDB	CPDB

Model Prediction

Prediction: 0.0251
Unit: mg/kg_body_weight/day
Mahalanobis Distance: 16.8
Mahalanobis Distance p-value: 4.53e-017

Mahalanobis Distance: The Mahalanobis distance (MD) is a generalization of the Euclidean distance that accounts for correlations among the X properties. It is calculated as the distance to the center of the training data. The larger the MD, the less trustworthy the prediction.
Mahalanobis Distance p-value: The p-value gives the fraction of training data with an MD greater than or equal to the one for the given sample, assuming normally distributed data. The smaller the p-value, the less trustworthy the prediciton. For highly non-normal X properties (e.g., fingerprints), the MD p-value is wildly inaccurate.

Model Applicability

Unknown features are fingerprint features in the query molecule, but not found or appearing too infrequently in the training set.

1. All properties and OPS components are within expected ranges.

Feature Contribution

Top features for positive contribution

Fingerprint	Bit/Smiles	Feature Structure	Score
FCFP_6	-1043250487	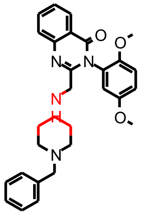 [*]CC(C[*])N[*]	1.15

FCFP_6	136627117	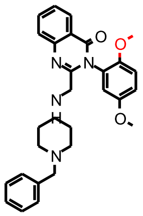 [*]OC	0.69
FCFP_6	9	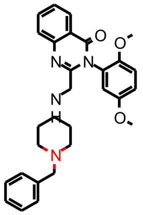 [*]N([*])[*]	0.385
**Top Features for negative contribution**			
**Fingerprint**	**Bit/Smiles**	**Feature Structure**	**Score**
FCFP_6	-1272709286	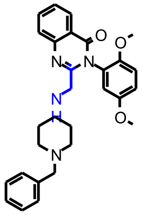 [*]NCC([*])[*]	-0.526
FCFP_6	991735244	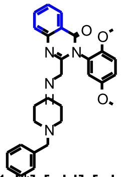 [*]:[c]1:[*]:[cH]:[cH]:[cH]:[cH]:1	-0.422
FCFP_6	-2093839777	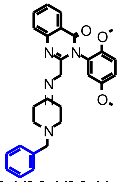 [*][c]1:[cH]:[cH]:[cH]:[cH]:[cH]:1	-0.378

#UNDEFINED

TOPKAT_Carcinogenic_Potency_TD50_Rat

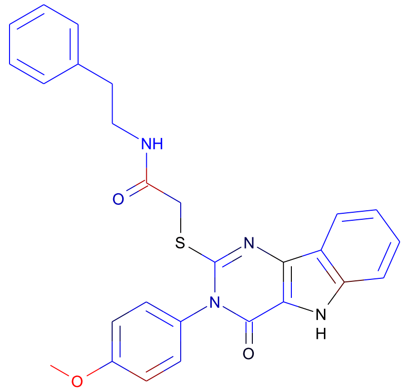

$C_{27}H_{24}N_4O_3S$

Molecular Weight: 484.56946

ALogP: 5.346

Rotatable Bonds: 8

Acceptors: 5

Donors: 2

Model Prediction

Prediction: 14.5

Unit: mg/kg_body_weight/day

Mahalanobis Distance: 17.7

Mahalanobis Distance p-value: 3.08e-020

Mahalanobis Distance: The Mahalanobis distance (MD) is a generalization of the Euclidean distance that accounts for correlations among the X properties. It is calculated as the distance to the center of the training data. The larger the MD, the less trustworthy the prediction.

Mahalanobis Distance p-value: The p-value gives the fraction of training data with an MD greater than or equal to the one for the given sample, assuming normally distributed data. The smaller the p-value, the less trustworthy the prediciton. For highly non-normal X properties (e.g., fingerprints), the MD p-value is wildly inaccurate.

Structural Similar Compounds

Name	Fluvastatin	913	C.I. direct brown 95
Structure	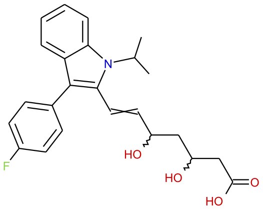	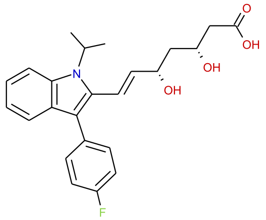	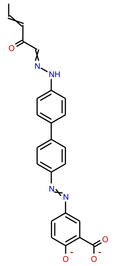
Actual Endpoint (-log C)	3.51742	3.51742	5.31387
Predicted Endpoint (-log C)	5.41573	5.41573	4.30266
Distance	0.784	0.784	0.808
Reference	CPDB	CPDB	CPDB

Model Applicability

Unknown features are fingerprint features in the query molecule, but not found or appearing too infrequently in the training set.

1. All properties and OPS components are within expected ranges.

Feature Contribution

Top features for positive contribution			
**Fingerprint**	**Bit/Smiles**	**Feature Structure**	**Score**
FCFP_6	136627117	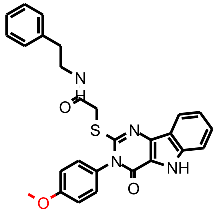 [*]OC	0.69

FCFP_6	1	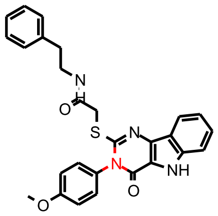 [*]N([*])[*]	0.234
FCFP_6	-885550502	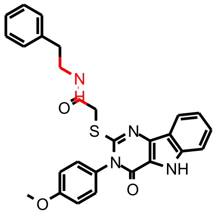 [*]CNC(=[*])[*]	0.229

Top Features for negative contribution

Fingerprint	Bit/Smiles	Feature Structure	Score
FCFP_6	-1272709286	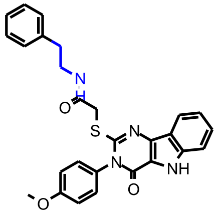 [*]NCC([*])[*]	-0.526
FCFP_6	991735244	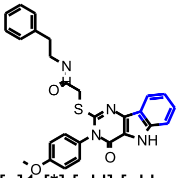 [*]:[c]1:[*]:[cH]:[cH]:[cH]:[cH]:1	-0.422
FCFP_6	-2093839777	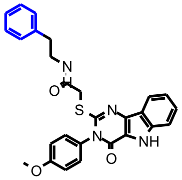 [*][c]1:[cH]:[cH]:[cH]:[cH]:[cH]:1	-0.378

Molecule

TOPKAT_Chronic_LOAEL

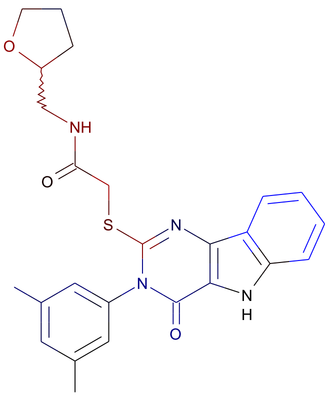

$C_{25}H_{26}N_4O_3S$
Molecular Weight: 462.56393
ALogP: 4.789
Rotatable Bonds: 6
Acceptors: 5
Donors: 2

Model Prediction

Prediction: 0.0179
Unit: g/kg_body_weight
Mahalanobis Distance: 45.5
Mahalanobis Distance p-value: 5.84e-051

Mahalanobis Distance: The Mahalanobis distance (MD) is a generalization of the Euclidean distance that accounts for correlations among the X properties. It is calculated as the distance to the center of the training data. The larger the MD, the less trustworthy the prediction.
Mahalanobis Distance p-value: The p-value gives the fraction of training data with an MD greater than or equal to the one for the given sample, assuming normally distributed data. The smaller the p-value, the less trustworthy the prediciton. For highly non-normal X properties (e.g., fingerprints), the MD p-value is wildly inaccurate.

Structural Similar Compounds

Name	GLYBURIDE	D & C RED 9	RHODAMINE 6G
Structure	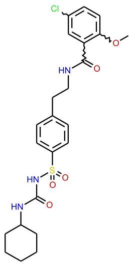	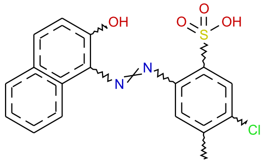	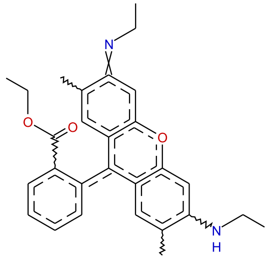
Actual Endpoint (-log C)	4.21661	3.87715	4.54906
Predicted Endpoint (-log C)	4.21035	3.6546	4.6787
Distance	0.647	0.675	0.715
Reference	UPJ-26452	NTP REPORT # 225	NTP 364 39

Model Applicability

Unknown features are fingerprint features in the query molecule, but not found or appearing too infrequently in the training set.

1. All properties and OPS components are within expected ranges.
2. Unknown ECFP_6 feature: -152683720: [*]:[nH]:[*]
3. Unknown ECFP_6 feature: -830332112: [*]S[*]
4. Unknown ECFP_6 feature: -782828288: [*]C(=[*])[c]1:[nH]:[*]:[*]:[c]:1[*]
5. Unknown ECFP_6 feature: -962771238: [*]C(=[*])N(C(=[*])[*])[c](:[*]):[*]
6. Unknown ECFP_6 feature: -962137479: [*][c]1:[*]:[*]:[c](:[*]):[c]:1N=[*]
7. Unknown ECFP_6 feature: 1945129186: [*]N([*])C(=O)[c](:[*]):[*]
8. Unknown ECFP_6 feature: 676970202: [*]S\C(=N\[*])\N([*])[*]
9. Unknown ECFP_6 feature: 2085698692: [*]C(=N[c](:[*]):[*])[*]
10. Unknown ECFP_6 feature: 558201926: [*][c]1:[*]:[*]:[c](:[*]):[nH]:1
11. Unknown ECFP_6 feature: 1333660716: [*][c]1:[*]:[*]:[c](:[*]):[c]:1:[cH]:[*]
12. Unknown ECFP_6 feature: -175021654: [*]N([*])[c](:[cH]:[*]):[cH]:[*]
13. Unknown ECFP_6 feature: 1099224616: [*]:[cH]:[c]1:[nH]:[*]:[*]:[c]:1:[*]
14. Unknown ECFP_6 feature: 1427820655: [*]CSC(=[*])[*]
15. Unknown ECFP_6 feature: 1731843802: [*]CC(=O)N[*]
16. Unknown ECFP_6 feature: 497523368: [*]CNC(=[*])[*]
17. Unknown ECFP_6 feature: -179515162: [*]:[cH]:[c](C):[cH]:[*]
18. Unknown ECFP_6 feature: -1939757055: [*]C1[*][*]CO1
19. Unknown ECFP_6 feature: -955816473: [*]SCC(=[*])[*]
20. Unknown ECFP_6 feature: -84772164: [*]NCC([*])[*]

21. Unknown ECFP_6 feature: -554480104: [*]CC1C[*][*]O1
22. Unknown ECFP_6 feature: -91640731: [*]1[*]OCC1
23. Unknown ECFP_6 feature: 1997021792: [*]:[cH]:[cH]:[cH]:[*]
24. Unknown ECFP_6 feature: -1332781180: [*]1[*]CCC1

Feature Contribution

Top features for positive contribution

Fingerprint	Bit/Smiles	Feature Structure	Score
ECFP_6	-167460056	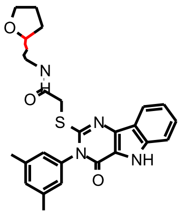 [*]C([*])[*]	0.136
FCFP_6	-1143715940	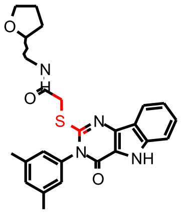 [*]CSC(=[*])[*]	0.13
ECFP_6	1559650422	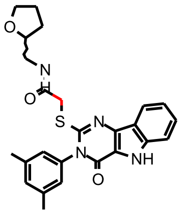 [*]C[*]	0.129

Top Features for negative contribution

Fingerprint	Bit/Smiles	Feature Structure	Score

FCFP_6	991735244	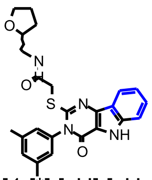 [*]:[c]1:[*]:[cH]:[cH]:[cH]:[cH]:1	-0.134
ECFP_6	1564392544	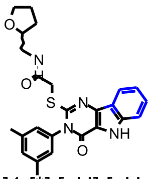 [*]:[c]1:[*]:[cH]:[cH]:[cH]:[cH]:1	-0.133
ECFP_6	2106656448	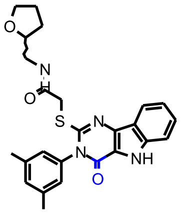 [*]C(=O)[*]	-0.11

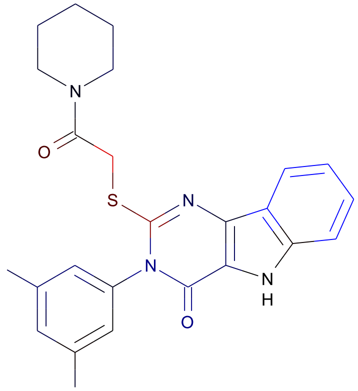

$C_{25}H_{26}N_4O_2S$
Molecular Weight: 446.56453
ALogP: 5.553
Rotatable Bonds: 4
Acceptors: 4
Donors: 1

Structural Similar Compounds

Name	C.I. PIGMENT RED 3	RHODAMINE 6G	D & C RED 9
Structure	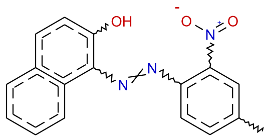	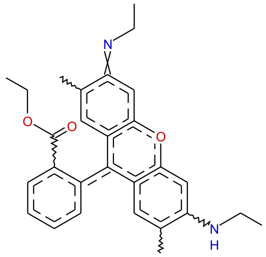	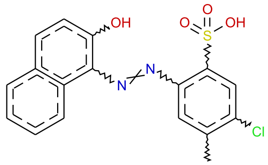
Actual Endpoint (-log C)	3.0252	4.54906	3.87715
Predicted Endpoint (-log C)	3.34768	4.6787	3.6546
Distance	0.675	0.682	0.693
Reference	NTP REPORT # 407	NTP 364 39	NTP REPORT # 225

Model Prediction

Prediction: 0.0817
Unit: g/kg_body_weight
Mahalanobis Distance: 36.8
Mahalanobis Distance p-value: 9.14e-037

Mahalanobis Distance: The Mahalanobis distance (MD) is a generalization of the Euclidean distance that accounts for correlations among the X properties. It is calculated as the distance to the center of the training data. The larger the MD, the less trustworthy the prediction.
Mahalanobis Distance p-value: The p-value gives the fraction of training data with an MD greater than or equal to the one for the given sample, assuming normally distributed data. The smaller the p-value, the less trustworthy the prediciton. For highly non-normal X properties (e.g., fingerprints), the MD p-value is wildly inaccurate.

Model Applicability

Unknown features are fingerprint features in the query molecule, but not found or appearing too infrequently in the training set.

1. All properties and OPS components are within expected ranges.
2. Unknown ECFP_6 feature: -152683720: [*]:[nH]:[*]
3. Unknown ECFP_6 feature: -830332112: [*]S[*]
4. Unknown ECFP_6 feature: -782828288: [*]C(=[*])[c]1:[nH]:[*]:[*]:[c]:1[*]
5. Unknown ECFP_6 feature: -962771238: [*]C(=[*])N(C(=[*])[*])[c](:[*]):[*]
6. Unknown ECFP_6 feature: -962137479: [*][c]1:[*]:[*]:[c](:[*]):[c]:1N=[*]
7. Unknown ECFP_6 feature: 1945129186: [*]N([*])C(=O)[c](:[*]):[*]
8. Unknown ECFP_6 feature: 676970202: [*]S\C(=N\[*])\N([*])[*]
9. Unknown ECFP_6 feature: 2085698692: [*]C(=N[c](:[*]):[*])[*]
10. Unknown ECFP_6 feature: 558201926: [*][c]1:[*]:[*]:[c](:[*]):[nH]:1
11. Unknown ECFP_6 feature: 1333660716: [*][c]1:[*]:[*]:[c](:[*]):[c]:1:[cH]:[*]
12. Unknown ECFP_6 feature: -175021654: [*]N([*])[c](:[cH]:[*]):[cH]:[*]
13. Unknown ECFP_6 feature: 1099224616: [*]:[cH]:[c]1:[nH]:[*]:[*]:[c]:1:[*]
14. Unknown ECFP_6 feature: 1427820655: [*]CSC(=[*])[*]
15. Unknown ECFP_6 feature: 1341750291: [*]CC(=O)N([*])[*]
16. Unknown ECFP_6 feature: -1102925512: [*]CN(C[*])C(=[*])[*]
17. Unknown ECFP_6 feature: -955816473: [*]SCC(=[*])[*]
18. Unknown ECFP_6 feature: -179515162: [*]:[cH]:[c](C):[cH]:[*]
19. Unknown ECFP_6 feature: -757679000: [*]CCN([*])[*]
20. Unknown ECFP_6 feature: 1997021792: [*]:[cH]:[cH]:[cH]:[*]

21.	Unknown ECFP_6 feature: -1332781180: [*]1[*]CCC1

Feature Contribution

Top features for positive contribution			
**Fingerprint**	**Bit/Smiles**	**Feature Structure**	**Score**
FCFP_6	-1143715940	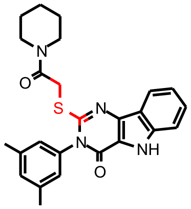 [*]CSC(=[*])[*]	0.13
ECFP_6	1559650422	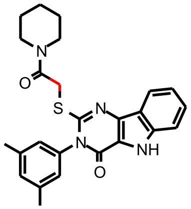 [*]C[*]	0.129
ECFP_6	2099970318	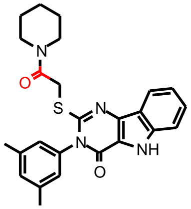 [*]C(=O)[*]	0.0766

Top Features for negative contribution			
**Fingerprint**	**Bit/Smiles**	**Feature Structure**	**Score**
FCFP_6	991735244	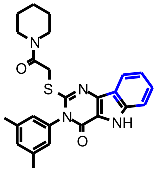 [*]:[c]1:[*]:[cH]:[cH]:[cH]:[cH]:1	-0.134

ECFP_6	1564392544	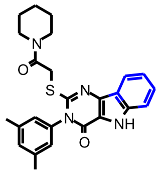 [*]:[c]1:[*]:[cH]:[cH]:[cH]:[cH]:1	-0.133
ECFP_6	2106656448	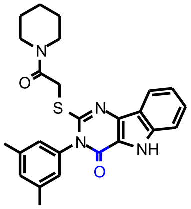 [*]C(=O)[*]	-0.11

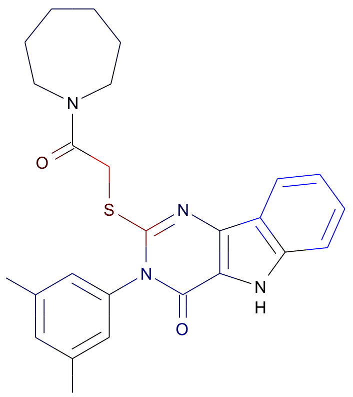

$C_{26}H_{28}N_4O_2S$
Molecular Weight: 460.59111
ALogP: 6.009
Rotatable Bonds: 4
Acceptors: 4
Donors: 1

Model Prediction

Prediction: 0.0713
Unit: g/kg_body_weight
Mahalanobis Distance: 36.8
Mahalanobis Distance p-value: 7.85e-037

Mahalanobis Distance: The Mahalanobis distance (MD) is a generalization of the Euclidean distance that accounts for correlations among the X properties. It is calculated as the distance to the center of the training data. The larger the MD, the less trustworthy the prediction.
Mahalanobis Distance p-value: The p-value gives the fraction of training data with an MD greater than or equal to the one for the given sample, assuming normally distributed data. The smaller the p-value, the less trustworthy the prediciton. For highly non-normal X properties (e.g., fingerprints), the MD p-value is wildly inaccurate.

Structural Similar Compounds

Name	RHODAMINE 6G	C.I. PIGMENT RED 3	D & C RED 9
Structure	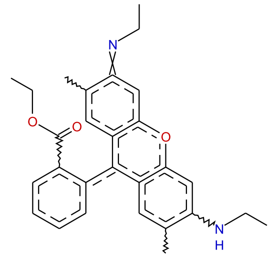	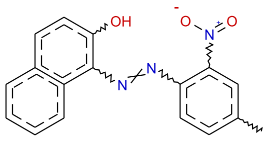	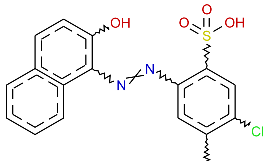
Actual Endpoint (-log C)	4.54906	3.0252	3.87715
Predicted Endpoint (-log C)	4.6787	3.34768	3.6546
Distance	0.685	0.707	0.715
Reference	NTP 364 39	NTP REPORT # 407	NTP REPORT # 225

Model Applicability

Unknown features are fingerprint features in the query molecule, but not found or appearing too infrequently in the training set.

1. All properties and OPS components are within expected ranges.
2. Unknown ECFP_6 feature: -152683720: [*]:[nH]:[*]
3. Unknown ECFP_6 feature: -830332112: [*]S[*]
4. Unknown ECFP_6 feature: -782828288: [*]C(=[*])[c]1:[nH]:[*]:[*]:[c]:1[*]
5. Unknown ECFP_6 feature: -962771238: [*]C(=[*])N(C(=[*])[*])[c](:[*]):[*]
6. Unknown ECFP_6 feature: -962137479: [*][c]1:[*]:[*]:[c](:[*]):[c]:1N=[*]
7. Unknown ECFP_6 feature: 1945129186: [*]N([*])C(=O)[c](:[*]):[*]
8. Unknown ECFP_6 feature: 676970202: [*]S\C(=N\[*])\N([*])[*]
9. Unknown ECFP_6 feature: 2085698692: [*]C(=N[c](:[*]):[*])[*]
10. Unknown ECFP_6 feature: 558201926: [*][c]1:[*]:[*]:[c](:[*]):[nH]:1
11. Unknown ECFP_6 feature: 1333660716: [*][c]1:[*]:[*]:[c](:[*]):[c]:1:[cH]:[*]
12. Unknown ECFP_6 feature: -175021654: [*]N([*])[c](:[cH]:[*]):[cH]:[*]
13. Unknown ECFP_6 feature: 1099224616: [*]:[cH]:[c]1:[nH]:[*]:[*]:[c]:1:[*]
14. Unknown ECFP_6 feature: 1427820655: [*]CSC(=[*])[*]
15. Unknown ECFP_6 feature: 1341750291: [*]CC(=O)N([*])[*]
16. Unknown ECFP_6 feature: -1102925512: [*]CN(C[*])C(=[*])[*]
17. Unknown ECFP_6 feature: -955816473: [*]SCC(=[*])[*]
18. Unknown ECFP_6 feature: -179515162: [*]:[cH]:[c](C):[cH]:[*]
19. Unknown ECFP_6 feature: -757679000: [*]CCN([*])[*]
20. Unknown ECFP_6 feature: 1997021792: [*]:[cH]:[cH]:[cH]:[*]

21. Unknown ECFP_6 feature: -1332781180: [*]1[*]CCC1

Feature Contribution

Top features for positive contribution			
**Fingerprint**	**Bit/Smiles**	**Feature Structure**	**Score**
FCFP_6	-1143715940	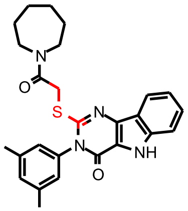 [*]CSC(=[*])[*]	0.13
ECFP_6	1559650422	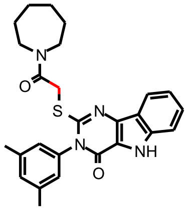 [*]C[*]	0.129
ECFP_6	2099970318	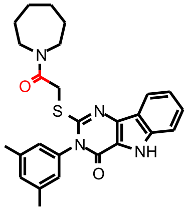 [*]C(=O)[*]	0.0766
**Top Features for negative contribution**			
**Fingerprint**	**Bit/Smiles**	**Feature Structure**	**Score**
FCFP_6	991735244	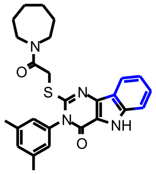 [*]:[c]1:[*]:[cH]:[cH]:[cH]:[cH]:1	-0.134

ECFP_6	1564392544	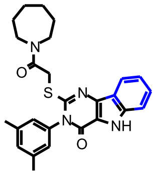 [*]:[c]1:[*]:[cH]:[cH]:[cH]:[cH]:1	-0.133
ECFP_6	2106656448	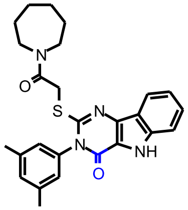 [*]C(=O)[*]	-0.11

#UNDEFINED

TOPKAT_Chronic_LOAEL

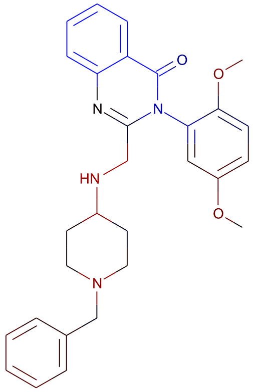

$C_{29}H_{32}N_4O_3$

Molecular Weight: 484.58938

ALogP: 3.743

Rotatable Bonds: 8

Acceptors: 6

Donors: 1

Model Prediction

Prediction: 0.0155

Unit: g/kg_body_weight

Mahalanobis Distance: 34.7

Mahalanobis Distance p-value: 3.66e-033

Mahalanobis Distance: The Mahalanobis distance (MD) is a generalization of the Euclidean distance that accounts for correlations among the X properties. It is calculated as the distance to the center of the training data. The larger the MD, the less trustworthy the prediction.
Mahalanobis Distance p-value: The p-value gives the fraction of training data with an MD greater than or equal to the one for the given sample, assuming normally distributed data. The smaller the p-value, the less trustworthy the prediciton. For highly non-normal X properties (e.g., fingerprints), the MD p-value is wildly inaccurate.

Structural Similar Compounds

Name	DILTIAZEM	RHODAMINE 6G	ASSURE
Structure	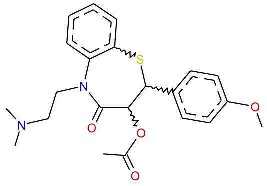	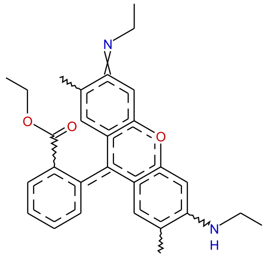	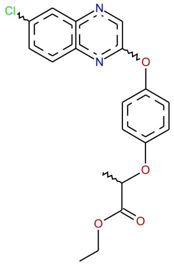
Actual Endpoint (-log C)	4.21961	4.54906	5.00328
Predicted Endpoint (-log C)	4.005	4.6787	4.27671
Distance	0.631	0.636	0.693
Reference	NDA-18602	NTP 364 39	EPA COVER SHEET 0335;891001;(1)

Model Applicability

Unknown features are fingerprint features in the query molecule, but not found or appearing too infrequently in the training set.

1. OPS PC19 out of range. Value: 5.7751. Training min, max, SD, explained variance: -5.9263, 5.2633, 1.805, 0.0142.
2. Unknown ECFP_6 feature: -962771238: [*]C(=[*])N(C(=[*])[*])[c](:[*]):[*]
3. Unknown ECFP_6 feature: 1945129186: [*]N([*])C(=O)[c](:[*]):[*]
4. Unknown ECFP_6 feature: -1073177635: [*]C\C(=N\[*])\N([*])[*]
5. Unknown ECFP_6 feature: 2085698692: [*]C(=N[c](:[*]):[*])[*]
6. Unknown ECFP_6 feature: -1236953626: [*]N([*])[c](:[cH]:[*]):[c]([*]):[*]
7. Unknown ECFP_6 feature: -597295171: [*][c](:[*]):[c](:[cH]:[*])N=[*]
8. Unknown ECFP_6 feature: -2041399277: [*]CN(C[*])C[*]
9. Unknown ECFP_6 feature: 769925792: [*]NCC(=[*])[*]
10. Unknown ECFP_6 feature: 496787418: [*]CNC([*])[*]
11. Unknown ECFP_6 feature: -44121127: [*]N([*])C[c](:[*]):[*]
12. Unknown ECFP_6 feature: -757679000: [*]CCN([*])[*]
13. Unknown ECFP_6 feature: -859078569: [*]CC(C[*])N[*]
14. Unknown ECFP_6 feature: 1307307440: [*]:[c](:[*])OC
15. Unknown ECFP_6 feature: 1997021792: [*]:[cH]:[cH]:[cH]:[*]

Feature Contribution

Top features for positive contribution

Fingerprint	Bit/Smiles	Feature Structure	Score
ECFP_6	-167460056	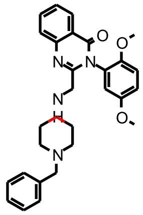 [*]C([*])[*]	0.136
ECFP_6	1559650422	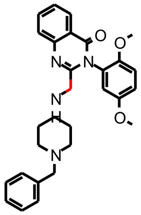 [*]C[*]	0.129
ECFP_6	-176455838	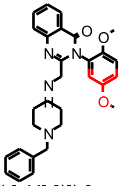 [*]O[c](:[cH]:[*]):[cH]:[*]	0.106

Top Features for negative contribution			
Fingerprint	Bit/Smiles	Feature Structure	Score
FCFP_6	991735244	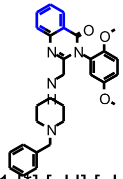 [*]:[c]1:[*]:[cH]:[cH]:[cH]:[cH]:1	-0.134

ECFP_6	1564392544	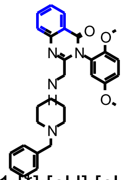 [*]:[c]1:[*]:[cH]:[cH]:[cH]:[cH]:1	-0.133
ECFP_6	2106656448	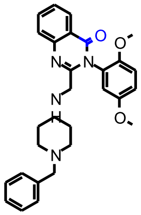 [*]C(=O)[*]	-0.11

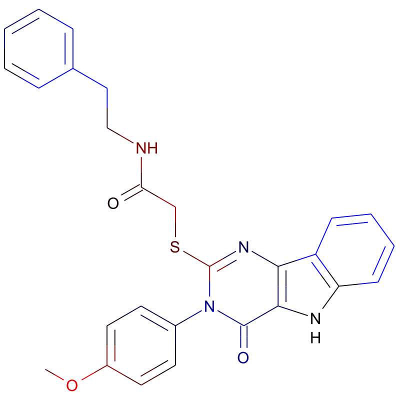

$C_{27}H_{24}N_4O_3S$
Molecular Weight: 484.56946
ALogP: 5.346
Rotatable Bonds: 8
Acceptors: 5
Donors: 2

Model Prediction

Prediction: 0.0171
Unit: g/kg_body_weight
Mahalanobis Distance: 42.9
Mahalanobis Distance p-value: 8.18e-047

Mahalanobis Distance: The Mahalanobis distance (MD) is a generalization of the Euclidean distance that accounts for correlations among the X properties. It is calculated as the distance to the center of the training data. The larger the MD, the less trustworthy the prediction.
Mahalanobis Distance p-value: The p-value gives the fraction of training data with an MD greater than or equal to the one for the given sample, assuming normally distributed data. The smaller the p-value, the less trustworthy the prediciton. For highly non-normal X properties (e.g., fingerprints), the MD p-value is wildly inaccurate.

Structural Similar Compounds

Name	FLUVALINATE	GLYBURIDE	D & C RED 9
Structure	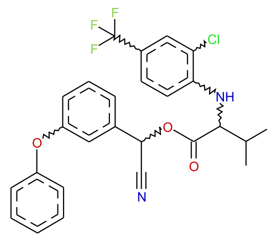	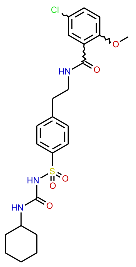	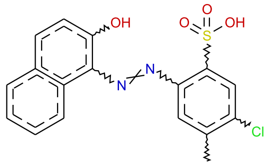
Actual Endpoint (-log C)	5.30356	4.21661	3.87715
Predicted Endpoint (-log C)	4.89944	4.21035	3.6546
Distance	0.734	0.751	0.810
Reference	EPA COVER SHEET 0281;880630;(1)	UPJ-26452	NTP REPORT # 225

Model Applicability

Unknown features are fingerprint features in the query molecule, but not found or appearing too infrequently in the training set.

1. OPS PC22 out of range. Value: -4.7826. Training min, max, SD, explained variance: -4.3287, 5.3383, 1.588, 0.0110.
2. Unknown ECFP_6 feature: -152683720: [*]:[nH]:[*]
3. Unknown ECFP_6 feature: -830332112: [*]S[*]
4. Unknown ECFP_6 feature: -782828288: [*]C(=[*])[c]1:[nH]:[*]:[*]:[c]:1[*]
5. Unknown ECFP_6 feature: -962771238: [*]C(=[*])N(C(=[*])[*])[c](:[*]):[*]
6. Unknown ECFP_6 feature: -962137479: [*][c]1:[*]:[*]:[c](:[*]):[c]:1N=[*]
7. Unknown ECFP_6 feature: 1945129186: [*]N([*])C(=O)[c](:[*]):[*]
8. Unknown ECFP_6 feature: 676970202: [*]S\C(=N\[*])\N([*])[*]
9. Unknown ECFP_6 feature: 2085698692: [*]C(=N[c](:[*]):[*])[*]
10. Unknown ECFP_6 feature: 558201926: [*][c]1:[*]:[*]:[c](:[*]):[nH]:1
11. Unknown ECFP_6 feature: 1333660716: [*][c]1:[*]:[*]:[c](:[*]):[c]:1:[cH]:[*]
12. Unknown ECFP_6 feature: 1099224616: [*]:[cH]:[c]1:[nH]:[*]:[*]:[c]:1:[*]
13. Unknown ECFP_6 feature: 1427820655: [*]CSC(=[*])[*]
14. Unknown ECFP_6 feature: -175021654: [*]N([*])[c](:[cH]:[*]):[cH]:[*]
15. Unknown ECFP_6 feature: 1731843802: [*]CC(=O)N[*]
16. Unknown ECFP_6 feature: -955816473: [*]SCC(=[*])[*]
17. Unknown ECFP_6 feature: 497523368: [*]CNC(=[*])[*]
18. Unknown ECFP_6 feature: -1791034651: [*]CCN[*]

19. Unknown ECFP_6 feature: 1307307440: [*]:[c](:[*])OC
20. Unknown ECFP_6 feature: -1795525632: [*]CC[c](:[*]):[*]
21. Unknown ECFP_6 feature: 1997021792: [*]:[cH]:[cH]:[cH]:[*]

Feature Contribution

Top features for positive contribution			
**Fingerprint**	**Bit/Smiles**	**Feature Structure**	**Score**
FCFP_6	-1143715940	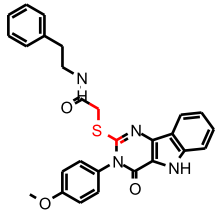 [*]CSC(=[*])[*]	0.13
ECFP_6	1559650422	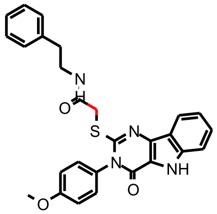 [*]C[*]	0.129
ECFP_6	-176455838	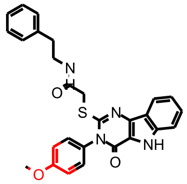 [*]O[c](:[cH]:[*]):[cH]:[*]	0.106

Top Features for negative contribution			
**Fingerprint**	**Bit/Smiles**	**Feature Structure**	**Score**

FCFP_6	991735244	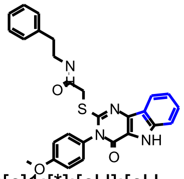 [*]:[c]1:[*]:[cH]:[cH]:[cH]:[cH]:1	-0.134
ECFP_6	1564392544	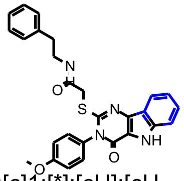 [*]:[c]1:[*]:[cH]:[cH]:[cH]:[cH]:1	-0.133
ECFP_6	2106656448	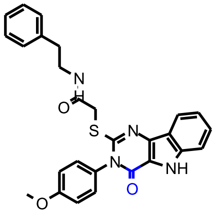 [*]C(=O)[*]	-0.11

Molecule

TOPKAT_Daphnia_EC50

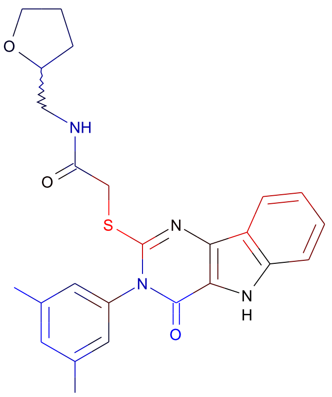

$C_{25}H_{26}N_4O_3S$

Molecular Weight: 462.56393

ALogP: 4.789

Rotatable Bonds: 6

Acceptors: 5

Donors: 2

Structural Similar Compounds

Name	Naphthaleneacetic acid	3-Methoxybenzeneamine	Bromadiolone
Structure	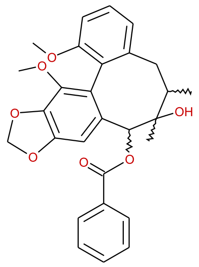	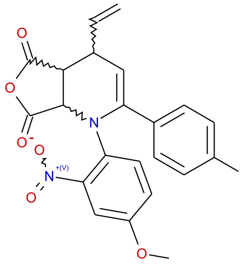	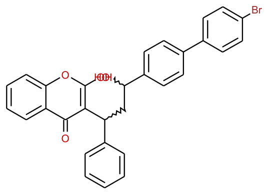
Actual Endpoint (-log C)	3.015	6.58436	5.421
Predicted Endpoint (-log C)	5.76302	5.62256	6.33935
Distance	0.655	0.691	0.780
Reference	Toropov and Benfenati, 2006, Bioorganic & Medicinal Chemistry, 14(8), 2779-2788	EPA EcoTox Database	Toropov and Benfenati, 2006, Bioorganic & Medicinal Chemistry, 14(8), 2779-2788

Model Prediction

Prediction: 0.469

Unit: mg/l

Mahalanobis Distance: 40.8

Mahalanobis Distance p-value: 1.08e-065

Mahalanobis Distance: The Mahalanobis distance (MD) is a generalization of the Euclidean distance that accounts for correlations among the X properties. It is calculated as the distance to the center of the training data. The larger the MD, the less trustworthy the prediction.
Mahalanobis Distance p-value: The p-value gives the fraction of training data with an MD greater than or equal to the one for the given sample, assuming normally distributed data. The smaller the p-value, the less trustworthy the prediciton. For highly non-normal X properties (e.g., fingerprints), the MD p-value is wildly inaccurate.

Model Applicability

Unknown features are fingerprint features in the query molecule, but not found or appearing too infrequently in the training set.

1. OPS PC24 out of range. Value: -4.2901. Training min, max, SD, explained variance: -4.1656, 7.024, 1.69, 0.0096.
2. Unknown FCFP_2 feature: 203707511: [*]C(=[*])[c]1:[nH]:[*]:[*]:[c]:1[*]
3. Unknown ECFP_6 feature: -152683720: [*]:[nH]:[*]
4. Unknown ECFP_6 feature: -782828288: [*]C(=[*])[c]1:[nH]:[*]:[*]:[c]:1[*]
5. Unknown ECFP_6 feature: -962771238: [*]C(=[*])N(C(=[*])[*])[c](:[*]):[*]
6. Unknown ECFP_6 feature: -962137479: [*][c]1:[*]:[*]:[c](:[*]):[c]:1N=[*]
7. Unknown ECFP_6 feature: 1945129186: [*]N([*])C(=O)[c](:[*]):[*]
8. Unknown ECFP_6 feature: 676970202: [*]S\C(=N\[*])\N([*])[*]
9. Unknown ECFP_6 feature: 2085698692: [*]C(=N[c](:[*]):[*])[*]
10. Unknown ECFP_6 feature: 558201926: [*][c]1:[*]:[*]:[c](:[*]):[nH]:1
11. Unknown ECFP_6 feature: 1333660716: [*][c]1:[*]:[*]:[c](:[*]):[c]:1:[cH]:[*]
12. Unknown ECFP_6 feature: -175021654: [*]N([*])[c](:[cH]:[*]):[cH]:[*]
13. Unknown ECFP_6 feature: 1099224616: [*]:[cH]:[c]1:[nH]:[*]:[*]:[c]:1:[*]
14. Unknown ECFP_6 feature: 1427820655: [*]CSC(=[*])[*]
15. Unknown ECFP_6 feature: 1731843802: [*]CC(=O)N[*]
16. Unknown ECFP_6 feature: 497523368: [*]CNC(=[*])[*]
17. Unknown ECFP_6 feature: -1939757055: [*]C1[*][*]CO1

18. Unknown ECFP_6 feature: -955816473: [*]SCC(=[*])[*]
19. Unknown ECFP_6 feature: -84772164: [*]NCC([*])[*]
20. Unknown ECFP_6 feature: -554480104: [*]CC1C[*][*]O1
21. Unknown ECFP_6 feature: -91640731: [*]1[*]OCC1
22. Unknown ECFP_6 feature: -1331450522: [*]C1[*][*]CC1
23. Unknown ECFP_6 feature: -1332781180: [*]1[*]CCC1

Feature Contribution

Top features for positive contribution			
**Fingerprint**	**Bit/Smiles**	**Feature Structure**	**Score**
FCFP_6	-1143715940	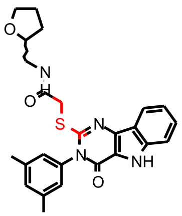 [*]CSC(=[*])[*]	0.218
ECFP_6	642810091	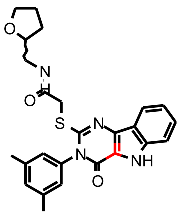 [*][c](:[*]):[*]	0.148
FCFP_6	-98332825	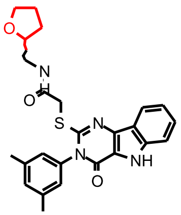 [*]C1CCCO1	0.0931

Top Features for negative contribution			
**Fingerprint**	**Bit/Smiles**	**Feature Structure**	**Score**

FCFP_6	0	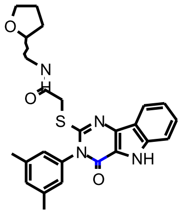 [*]C(=[*])[*]	-0.202
ECFP_6	-1100000244	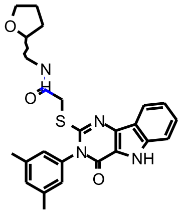 [*]C(=[*])[*]	-0.199
ECFP_6	-1074141656	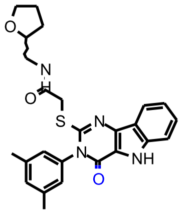 [*]=O	-0.158

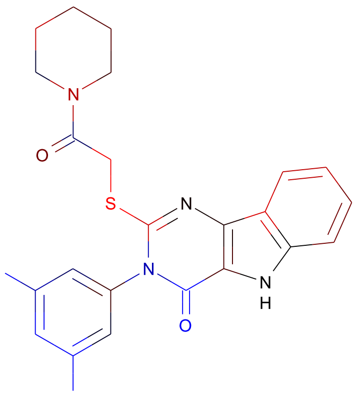

$C_{25}H_{26}N_4O_2S$
Molecular Weight: 446.56453
ALogP: 5.553
Rotatable Bonds: 4
Acceptors: 4
Donors: 1

Structural Similar Compounds

Name	Naphthaleneacetic acid	Difenoconazole	Rotenone
Structure	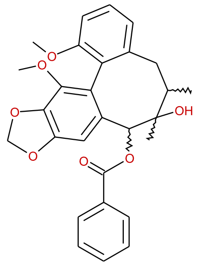	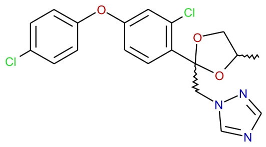	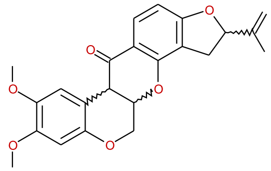
Actual Endpoint (-log C)	3.015	5.722	8.028
Predicted Endpoint (-log C)	5.76302	5.71868	5.51369
Distance	0.665	0.682	0.752
Reference	Toropov and Benfenati, 2006, Bioorganic & Medicinal Chemistry, 14(8), 2779-2788	Toropov and Benfenati, 2006, Bioorganic & Medicinal Chemistry, 14(8), 2779-2788	Toropov and Benfenati, 2006, Bioorganic & Medicinal Chemistry, 14(8), 2779-2788

Model Prediction

Prediction: 0.15
Unit: mg/l
Mahalanobis Distance: 41.6
Mahalanobis Distance p-value: 4.32e-068

Mahalanobis Distance: The Mahalanobis distance (MD) is a generalization of the Euclidean distance that accounts for correlations among the X properties. It is calculated as the distance to the center of the training data. The larger the MD, the less trustworthy the prediction.
Mahalanobis Distance p-value: The p-value gives the fraction of training data with an MD greater than or equal to the one for the given sample, assuming normally distributed data. The smaller the p-value, the less trustworthy the prediciton. For highly non-normal X properties (e.g., fingerprints), the MD p-value is wildly inaccurate.

Model Applicability

Unknown features are fingerprint features in the query molecule, but not found or appearing too infrequently in the training set.

1. All properties and OPS components are within expected ranges.
2. Unknown FCFP_2 feature: 203707511: [*]C(=[*])[c]1:[nH]:[*]:[*]:[c]:1[*]
3. Unknown ECFP_6 feature: -152683720: [*]:[nH]:[*]
4. Unknown ECFP_6 feature: -782828288: [*]C(=[*])[c]1:[nH]:[*]:[*]:[c]:1[*]
5. Unknown ECFP_6 feature: -962771238: [*]C(=[*])N(C(=[*])[*])[c](:[*]):[*]
6. Unknown ECFP_6 feature: -962137479: [*][c]1:[*]:[*]:[c](:[*]):[c]:1N=[*]
7. Unknown ECFP_6 feature: 1945129186: [*]N([*])C(=O)[c](:[*]):[*]
8. Unknown ECFP_6 feature: 676970202: [*]S\C(=N\[*])\N([*])[*]
9. Unknown ECFP_6 feature: 2085698692: [*]C(=N[c](:[*]):[*])[*]
10. Unknown ECFP_6 feature: 558201926: [*][c]1:[*]:[*]:[c](:[*]):[nH]:1
11. Unknown ECFP_6 feature: 1333660716: [*][c]1:[*]:[*]:[c](:[*]):[c]:1:[cH]:[*]
12. Unknown ECFP_6 feature: -175021654: [*]N([*])[c](:[cH]:[*]):[cH]:[*]
13. Unknown ECFP_6 feature: 1099224616: [*]:[cH]:[c]1:[nH]:[*]:[*]:[c]:1:[*]
14. Unknown ECFP_6 feature: 1427820655: [*]CSC(=[*])[*]
15. Unknown ECFP_6 feature: 1341750291: [*]CC(=O)N([*])[*]
16. Unknown ECFP_6 feature: -1102925512: [*]CN(C[*])C(=[*])[*]
17. Unknown ECFP_6 feature: -955816473: [*]SCC(=[*])[*]
18. Unknown ECFP_6 feature: -757679000: [*]CCN([*])[*]

19.	Unknown ECFP_6 feature: -1332781180: [*]1[*]CCC1

Feature Contribution

Top features for positive contribution

Fingerprint	Bit/Smiles	Feature Structure	Score
FCFP_6	-1143715940	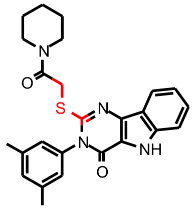 [*]CSC(=[*])[*]	0.218
ECFP_6	642810091	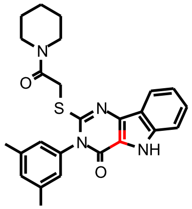 [*][c](:[*]):[*]	0.148
FCFP_6	565998553	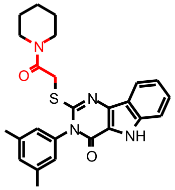 [*]CC(=O)N([*])[*]	0.114

Top Features for negative contribution

Fingerprint	Bit/Smiles	Feature Structure	Score
FCFP_6	0	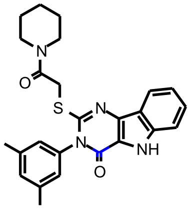 [*]C(=[*])[*]	-0.202

ECFP_6	-1100000244	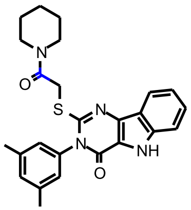 [*]C(=[*])[*]	-0.199
ECFP_6	-1074141656	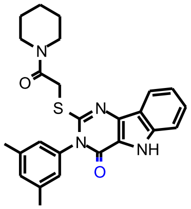 [*]=O	-0.158

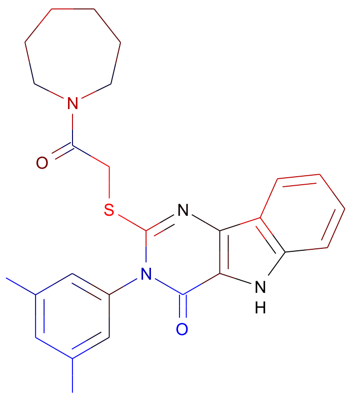

$C_{26}H_{28}N_4O_2S$
Molecular Weight: 460.59111
ALogP: 6.009
Rotatable Bonds: 4
Acceptors: 4
Donors: 1

Structural Similar Compounds

Name	Naphthaleneacetic acid	Difenoconazole	Bromadiolone
Structure	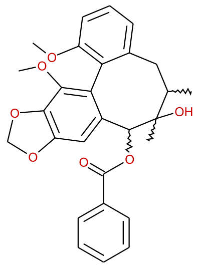	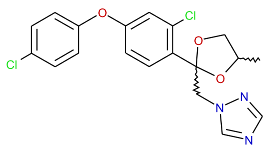	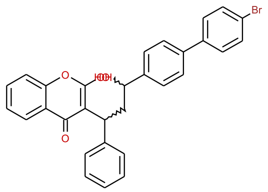
Actual Endpoint (-log C)	3.015	5.722	5.421
Predicted Endpoint (-log C)	5.76302	5.71868	6.33935
Distance	0.665	0.703	0.739
Reference	Toropov and Benfenati, 2006, Bioorganic & Medicinal Chemistry, 14(8), 2779-2788	Toropov and Benfenati, 2006, Bioorganic & Medicinal Chemistry, 14(8), 2779-2788	Toropov and Benfenati, 2006, Bioorganic & Medicinal Chemistry, 14(8), 2779-2788

Model Prediction

Prediction: 0.112
Unit: mg/l
Mahalanobis Distance: 41.6
Mahalanobis Distance p-value: 3.66e-068

Mahalanobis Distance: The Mahalanobis distance (MD) is a generalization of the Euclidean distance that accounts for correlations among the X properties. It is calculated as the distance to the center of the training data. The larger the MD, the less trustworthy the prediction.
Mahalanobis Distance p-value: The p-value gives the fraction of training data with an MD greater than or equal to the one for the given sample, assuming normally distributed data. The smaller the p-value, the less trustworthy the prediciton. For highly non-normal X properties (e.g., fingerprints), the MD p-value is wildly inaccurate.

Model Applicability

Unknown features are fingerprint features in the query molecule, but not found or appearing too infrequently in the training set.

1. All properties and OPS components are within expected ranges.
2. Unknown FCFP_2 feature: 203707511: [*]C(=[*])[c]1:[nH]:[*]:[*]:[c]:1[*]
3. Unknown ECFP_6 feature: -152683720: [*]:[nH]:[*]
4. Unknown ECFP_6 feature: -782828288: [*]C(=[*])[c]1:[nH]:[*]:[*]:[c]:1[*]
5. Unknown ECFP_6 feature: -962771238: [*]C(=[*])N(C(=[*])[*])[c](:[*]):[*]
6. Unknown ECFP_6 feature: -962137479: [*][c]1:[*]:[*]:[c](:[*]):[c]:1N=[*]
7. Unknown ECFP_6 feature: 1945129186: [*]N([*])C(=O)[c](:[*]):[*]
8. Unknown ECFP_6 feature: 676970202: [*]S\C(=N\[*])\N([*])[*]
9. Unknown ECFP_6 feature: 2085698692: [*]C(=N[c](:[*]):[*])[*]
10. Unknown ECFP_6 feature: 558201926: [*][c]1:[*]:[*]:[c](:[*]):[nH]:1
11. Unknown ECFP_6 feature: 1333660716: [*][c]1:[*]:[*]:[c](:[*]):[c]:1:[cH]:[*]
12. Unknown ECFP_6 feature: -175021654: [*]N([*])[c](:[cH]:[*]):[cH]:[*]
13. Unknown ECFP_6 feature: 1099224616: [*]:[cH]:[c]1:[nH]:[*]:[*]:[c]:1:[*]
14. Unknown ECFP_6 feature: 1427820655: [*]CSC(=[*])[*]
15. Unknown ECFP_6 feature: 1341750291: [*]CC(=O)N([*])[*]
16. Unknown ECFP_6 feature: -1102925512: [*]CN(C[*])C(=[*])[*]
17. Unknown ECFP_6 feature: -955816473: [*]SCC(=[*])[*]
18. Unknown ECFP_6 feature: -757679000: [*]CCN([*])[*]

19. Unknown ECFP_6 feature: -1332781180: [*]1[*]CCC1

Feature Contribution

Top features for positive contribution

Fingerprint	Bit/Smiles	Feature Structure	Score
FCFP_6	-1143715940	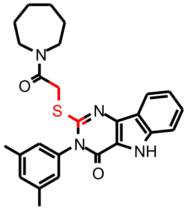 [*]CSC(=[*])[*]	0.218
ECFP_6	642810091	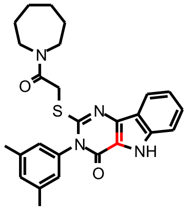 [*][c](:[*]):[*]	0.148
FCFP_6	565998553	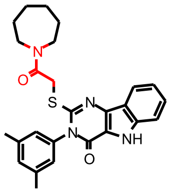 [*]CC(=O)N([*])[*]	0.114

Top Features for negative contribution

Fingerprint	Bit/Smiles	Feature Structure	Score
FCFP_6	0	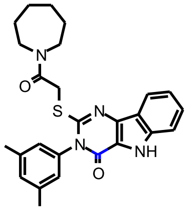 [*]C(=[*])[*]	-0.202

ECFP_6	-1100000244	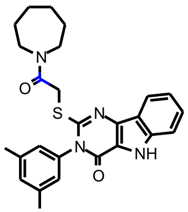 [*]C(=[*])[*]	-0.199
ECFP_6	-1074141656	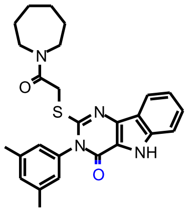 [*]=O	-0.158

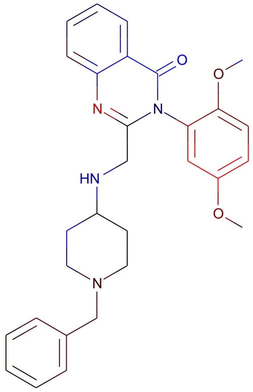

$C_{29}H_{32}N_4O_3$

Molecular Weight: 484.58938

ALogP: 3.743

Rotatable Bonds: 8

Acceptors: 6

Donors: 1

Structural Similar Compounds

Name	Naphthaleneacetic acid	Fenpyroximate	Difenoconazole
Structure	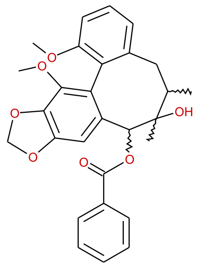	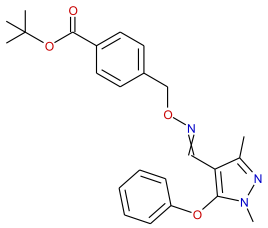	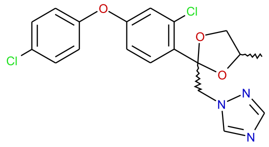
Actual Endpoint (-log C)	3.015	8.068	5.722
Predicted Endpoint (-log C)	5.76302	6.55124	5.71868
Distance	0.596	0.698	0.704
Reference	Toropov and Benfenati, 2006, Bioorganic & Medicinal Chemistry, 14(8), 2779-2788	Toropov and Benfenati, 2006, Bioorganic & Medicinal Chemistry, 14(8), 2779-2788	Toropov and Benfenati, 2006, Bioorganic & Medicinal Chemistry, 14(8), 2779-2788

Model Prediction

Prediction: 0.14

Unit: mg/l

Mahalanobis Distance: 38.2

Mahalanobis Distance p-value: 1e-057

Mahalanobis Distance: The Mahalanobis distance (MD) is a generalization of the Euclidean distance that accounts for correlations among the X properties. It is calculated as the distance to the center of the training data. The larger the MD, the less trustworthy the prediction.

Mahalanobis Distance p-value: The p-value gives the fraction of training data with an MD greater than or equal to the one for the given sample, assuming normally distributed data. The smaller the p-value, the less trustworthy the prediciton. For highly non-normal X properties (e.g., fingerprints), the MD p-value is wildly inaccurate.

Model Applicability

Unknown features are fingerprint features in the query molecule, but not found or appearing too infrequently in the training set.

1. All properties and OPS components are within expected ranges.
2. Unknown FCFP_2 feature: 906798516: [*]N([*])C[c](:[*]):[*]
3. Unknown ECFP_6 feature: -962771238: [*]C(=[*])N(C(=[*])[*])[c](:[*]):[*]
4. Unknown ECFP_6 feature: 1945129186: [*]N([*])C(=O)[c](:[*]):[*]
5. Unknown ECFP_6 feature: -1073177635: [*]C\C(=N\[*])\N([*])[*]
6. Unknown ECFP_6 feature: 2085698692: [*]C(=N[c](:[*]):[*])[*]
7. Unknown ECFP_6 feature: -1236953626: [*]N([*])[c](:[cH]:[*]):[c]([*]):[*]
8. Unknown ECFP_6 feature: -427397688: [*]C(=[*])[c](:[cH]:[*]):[c]([*]):[*]
9. Unknown ECFP_6 feature: -597295171: [*][c](:[*]):[c](:[cH]:[*])N=[*]
10. Unknown ECFP_6 feature: -2041399277: [*]CN(C[*])C[*]
11. Unknown ECFP_6 feature: 769925792: [*]NCC(=[*])[*]
12. Unknown ECFP_6 feature: 496787418: [*]CNC([*])[*]
13. Unknown ECFP_6 feature: -44121127: [*]N([*])C[c](:[*]):[*]
14. Unknown ECFP_6 feature: -1331450522: [*]C1[*][*]CC1
15. Unknown ECFP_6 feature: -757679000: [*]CCN([*])[*]
16. Unknown ECFP_6 feature: -859078569: [*]CC(C[*])N[*]
17. Unknown ECFP_6 feature: 1307307440: [*]:[c](:[*])OC

Feature Contribution

Top features for positive contribution

Fingerprint	Bit/Smiles	Feature Structure	Score
ECFP_6	-1059365320	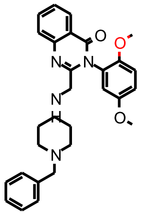 [*]O[*]	0.165
ECFP_6	642810091	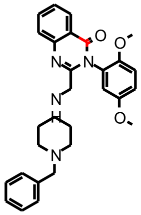 [*][c](:[*]):[*]	0.148
FCFP_6	565998553	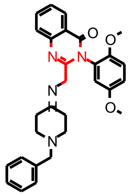 [*]CC(=O)N([*])[*]	0.114

Top Features for negative contribution

Fingerprint	Bit/Smiles	Feature Structure	Score
FCFP_6	0	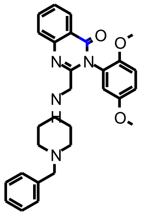 [*]C(=[*])[*]	-0.202

ECFP_6	-1074141656	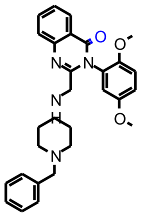 [*]=O	-0.158
ECFP_6	734603939	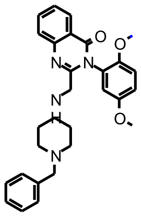 [*]C	-0.157

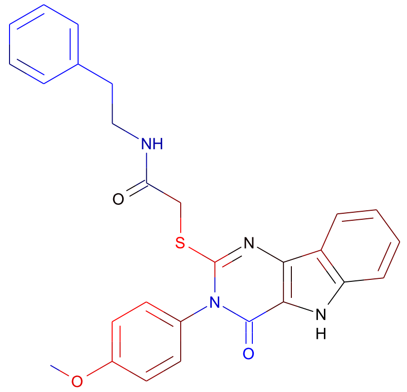

$C_{27}H_{24}N_4O_3S$

Molecular Weight: 484.56946

ALogP: 5.346

Rotatable Bonds: 8

Acceptors: 5

Donors: 2

Structural Similar Compounds

Name	Naphthaleneacetic acid	Bromadiolone	Fenpyroximate
Structure	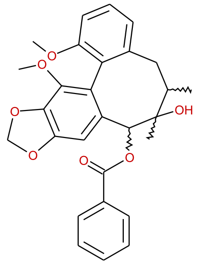	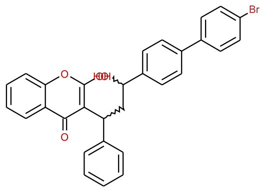	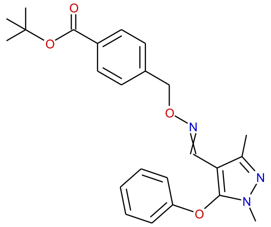
Actual Endpoint (-log C)	3.015	5.421	8.068
Predicted Endpoint (-log C)	5.76302	6.33935	6.55124
Distance	0.716	0.716	0.828
Reference	Toropov and Benfenati, 2006, Bioorganic & Medicinal Chemistry, 14(8), 2779-2788	Toropov and Benfenati, 2006, Bioorganic & Medicinal Chemistry, 14(8), 2779-2788	Toropov and Benfenati, 2006, Bioorganic & Medicinal Chemistry, 14(8), 2779-2788

Model Prediction

Prediction: 0.106

Unit: mg/l

Mahalanobis Distance: 39.3

Mahalanobis Distance p-value: 4.03e-061

Mahalanobis Distance: The Mahalanobis distance (MD) is a generalization of the Euclidean distance that accounts for correlations among the X properties. It is calculated as the distance to the center of the training data. The larger the MD, the less trustworthy the prediction.
Mahalanobis Distance p-value: The p-value gives the fraction of training data with an MD greater than or equal to the one for the given sample, assuming normally distributed data. The smaller the p-value, the less trustworthy the prediciton. For highly non-normal X properties (e.g., fingerprints), the MD p-value is wildly inaccurate.

Model Applicability

Unknown features are fingerprint features in the query molecule, but not found or appearing too infrequently in the training set.

1. All properties and OPS components are within expected ranges.
2. Unknown FCFP_2 feature: 203707511: [*]C(=[*])[c]1:[nH]:[*]:[*]:[c]:1[*]
3. Unknown ECFP_6 feature: -152683720: [*]:[nH]:[*]
4. Unknown ECFP_6 feature: -782828288: [*]C(=[*])[c]1:[nH]:[*]:[*]:[c]:1[*]
5. Unknown ECFP_6 feature: -962771238: [*]C(=[*])N(C(=[*])[*])[c](:[*]):[*]
6. Unknown ECFP_6 feature: -962137479: [*][c]1:[*]:[*]:[c](:[*]):[c]:1N=[*]
7. Unknown ECFP_6 feature: 1945129186: [*]N([*])C(=O)[c](:[*]):[*]
8. Unknown ECFP_6 feature: 676970202: [*]S\C(=N\[*])\N([*])[*]
9. Unknown ECFP_6 feature: 2085698692: [*]C(=N[c](:[*]):[*])[*]
10. Unknown ECFP_6 feature: 558201926: [*][c]1:[*]:[*]:[c](:[*]):[nH]:1
11. Unknown ECFP_6 feature: 1333660716: [*][c]1:[*]:[*]:[c](:[*]):[c]:1:[cH]:[*]
12. Unknown ECFP_6 feature: 1099224616: [*]:[cH]:[c]1:[nH]:[*]:[*]:[c]:1:[*]
13. Unknown ECFP_6 feature: 1427820655: [*]CSC(=[*])[*]
14. Unknown ECFP_6 feature: -175021654: [*]N([*])[c](:[cH]:[*]):[cH]:[*]
15. Unknown ECFP_6 feature: 1731843802: [*]CC(=O)N[*]
16. Unknown ECFP_6 feature: -955816473: [*]SCC(=[*])[*]
17. Unknown ECFP_6 feature: 497523368: [*]CNC(=[*])[*]
18. Unknown ECFP_6 feature: -1791034651: [*]CCN[*]

19. Unknown ECFP_6 feature: 1307307440: [*]:[c](:[*])OC
20. Unknown ECFP_6 feature: -1795525632: [*]CC[c](:[*]):[*]

Feature Contribution

Top features for positive contribution			
**Fingerprint**	**Bit/Smiles**	**Feature Structure**	**Score**
FCFP_6	-1143715940	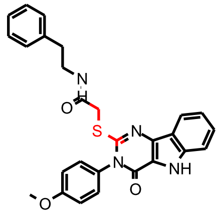 [*]CSC(=[*])[*]	0.218
ECFP_6	-1059365320	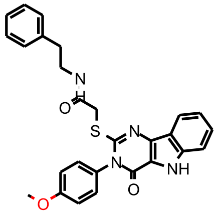 [*]O[*]	0.165
ECFP_6	642810091	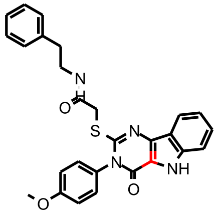 [*][c](:[*]):[*]	0.148
**Top Features for negative contribution**			
**Fingerprint**	**Bit/Smiles**	**Feature Structure**	**Score**
FCFP_6	0	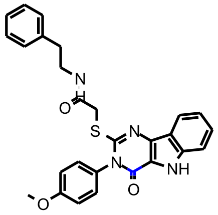 [*]C(=[*])[*]	-0.202

ECFP_6	-1100000244	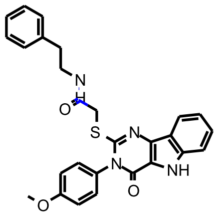<br>[*]C(=[*])[*]	-0.199
ECFP_6	-1074141656	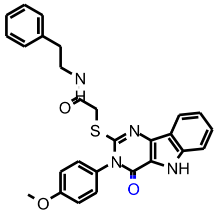<br>[*]=O	-0.158

Molecule

TOPKAT_Fathead_Minnow_LC50

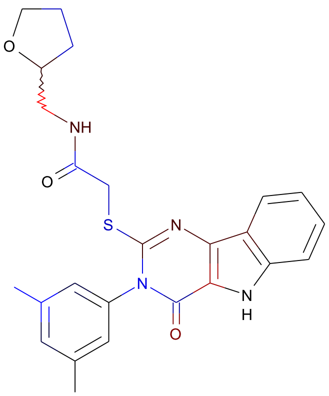

$C_{25}H_{26}N_4O_3S$
Molecular Weight: 462.56393
ALogP: 4.789
Rotatable Bonds: 6
Acceptors: 5
Donors: 2

Structural Similar Compounds

Name	Dicumarol	O-Ethyl O-(p-nitrophenyl phenyl)phosphonothioate	Fenvalerate (test 2)
Structure	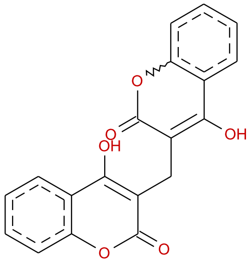	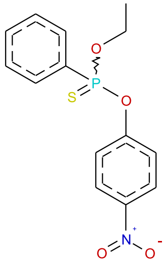	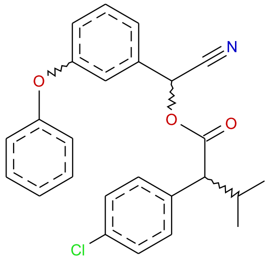
Actual Endpoint (-log C)	4.81816	6.61439	9
Predicted Endpoint (-log C)	5.081	6.0368	7.86616
Distance	0.927	1.042	1.095
Reference	DSSTox/EPAFHM	DSSTox/EPAFHM	ATOCFM Volume 4

Model Prediction

Prediction: 0.000208
Unit: g/l
Mahalanobis Distance: 14.1
Mahalanobis Distance p-value: 1.58e-016

Mahalanobis Distance: The Mahalanobis distance (MD) is a generalization of the Euclidean distance that accounts for correlations among the X properties. It is calculated as the distance to the center of the training data. The larger the MD, the less trustworthy the prediction.
Mahalanobis Distance p-value: The p-value gives the fraction of training data with an MD greater than or equal to the one for the given sample, assuming normally distributed data. The smaller the p-value, the less trustworthy the prediciton. For highly non-normal X properties (e.g., fingerprints), the MD p-value is wildly inaccurate.

Model Applicability

Unknown features are fingerprint features in the query molecule, but not found or appearing too infrequently in the training set.

1. All properties and OPS components are within expected ranges.
2. Unknown FCFP_2 feature: 203707511: [*]C(=[*])[c]1:[nH]:[*]:[*]:[c]:1[*]
3. Unknown FCFP_2 feature: 580453787: [*]C(=N[c](:[*]):[*])[*]

Feature Contribution

Top features for positive contribution

Fingerprint	Bit/Smiles	Feature Structure	Score
FCFP_2	-1272709286	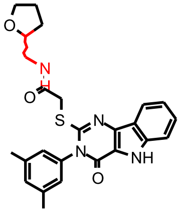 [*]NCC([*])[*]	0.237

FCFP_2	-1549163031	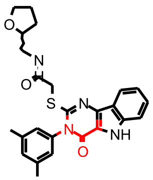 [*]N([*])C(=O)[c](:[*]):[*]	0.078
FCFP_2	332760439	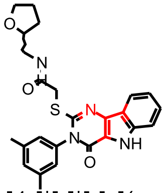 [*][c]1:[*]:[*]:[c](:[*]):[c]:1N=[*]	0.0709

Top Features for negative contribution

Fingerprint	Bit/Smiles	Feature Structure	Score
FCFP_2	1	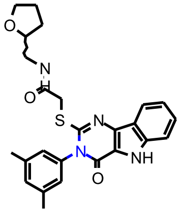 [*]N([*])[*]	-0.306
FCFP_2	0	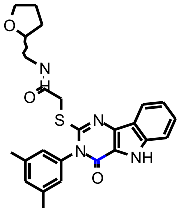 [*]C(=[*])[*]	-0.275
FCFP_2	-1272768868	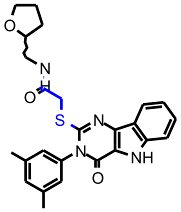 [*]SCC(=[*])[*]	-0.247

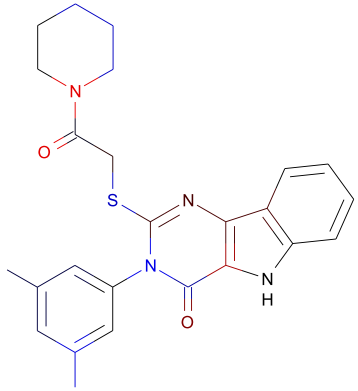

$C_{25}H_{26}N_4O_2S$
Molecular Weight: 446.56453
ALogP: 5.553
Rotatable Bonds: 4
Acceptors: 4
Donors: 1

Model Prediction

Prediction: 4.91e-005
Unit: g/l
Mahalanobis Distance: 13
Mahalanobis Distance p-value: 7.25e-013

Mahalanobis Distance: The Mahalanobis distance (MD) is a generalization of the Euclidean distance that accounts for correlations among the X properties. It is calculated as the distance to the center of the training data. The larger the MD, the less trustworthy the prediction.
Mahalanobis Distance p-value: The p-value gives the fraction of training data with an MD greater than or equal to the one for the given sample, assuming normally distributed data. The smaller the p-value, the less trustworthy the prediciton. For highly non-normal X properties (e.g., fingerprints), the MD p-value is wildly inaccurate.

Structural Similar Compounds

Name	Fenvalerate (test 2)	Diphenylphthalate	Triphenyl phosphate
Structure	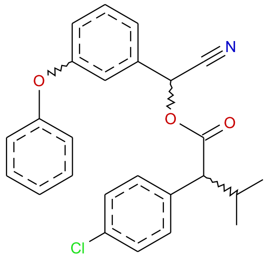	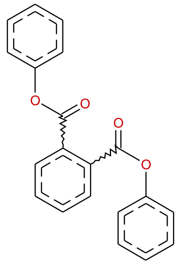	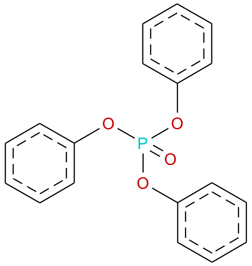
Actual Endpoint (-log C)	9	6.6	5.57512
Predicted Endpoint (-log C)	7.86616	6.4642	6.43312
Distance	0.821	0.839	0.899
Reference	ATOCFM Volume 4	ATOCFM Volume 2	DSSTox/EPAFHM

Model Applicability

Unknown features are fingerprint features in the query molecule, but not found or appearing too infrequently in the training set.

1. All properties and OPS components are within expected ranges.
2. Unknown FCFP_2 feature: 203707511: [*]C(=[*])[c]1:[nH]:[*]:[*]:[c]:1[*]
3. Unknown FCFP_2 feature: 580453787: [*]C(=N[c](:[*]):[*])[*]

Feature Contribution

Top features for positive contribution

Fingerprint	Bit/Smiles	Feature Structure	Score
FCFP_2	565998553	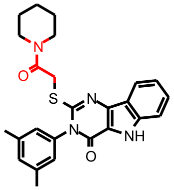 [*]CC(=O)N([*])[*]	0.224

FCFP_2	-1549163031	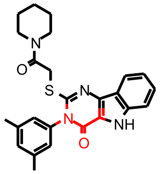 [*]N([*])C(=O)[c](:[*]):[*]	0.078
FCFP_2	332760439	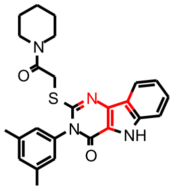 [*][c]1:[*]:[*]:[c](:[*]):[c]:1N=[*]	0.0709
**Top Features for negative contribution**			
**Fingerprint**	**Bit/Smiles**	**Feature Structure**	**Score**
FCFP_2	1	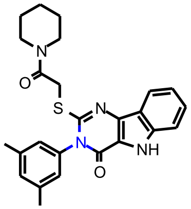 [*]N([*])[*]	-0.306
FCFP_2	0	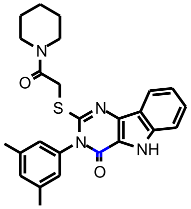 [*]C(=[*])[*]	-0.275
FCFP_2	-1272768868	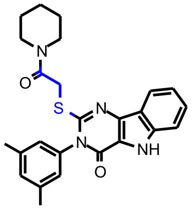 [*]SCC(=[*])[*]	-0.247

#UNDEFINED

TOPKAT_Fathead_Minnow_LC50

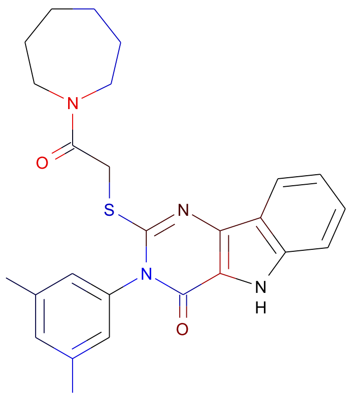

$C_{26}H_{28}N_4O_2S$

Molecular Weight: 460.59111

ALogP: 6.009

Rotatable Bonds: 4

Acceptors: 4

Donors: 1

Model Prediction

Prediction: 2.58e-005

Unit: g/l

Mahalanobis Distance: 13.2

Mahalanobis Distance p-value: 3.03e-013

Mahalanobis Distance: The Mahalanobis distance (MD) is a generalization of the Euclidean distance that accounts for correlations among the X properties. It is calculated as the distance to the center of the training data. The larger the MD, the less trustworthy the prediction.

Mahalanobis Distance p-value: The p-value gives the fraction of training data with an MD greater than or equal to the one for the given sample, assuming normally distributed data. The smaller the p-value, the less trustworthy the prediciton. For highly non-normal X properties (e.g., fingerprints), the MD p-value is wildly inaccurate.

Structural Similar Compounds

Name	Fenvalerate (test 2)	Diphenylphthalate	Flucythrinate
Structure	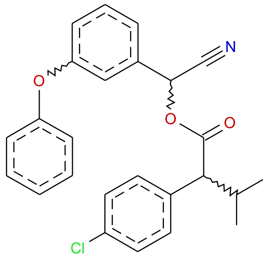	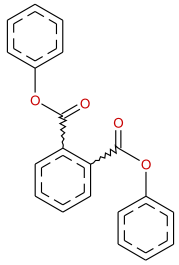	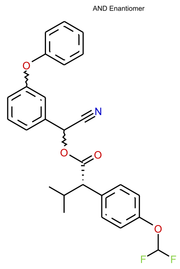
Actual Endpoint (-log C)	9	6.6	9.37572
Predicted Endpoint (-log C)	7.86616	6.4642	8.49266
Distance	0.815	0.872	0.896
Reference	ATOCFM Volume 4	ATOCFM Volume 2	DSSTox/EPAFHM

Model Applicability

Unknown features are fingerprint features in the query molecule, but not found or appearing too infrequently in the training set.

1. All properties and OPS components are within expected ranges.
2. Unknown FCFP_2 feature: 203707511: [*]C(=[*])[c]1:[nH]:[*]:[*]:[c]:1[*]
3. Unknown FCFP_2 feature: 580453787: [*]C(=N[c](:[*]):[*])[*]

Feature Contribution

Top features for positive contribution			
**Fingerprint**	**Bit/Smiles**	**Feature Structure**	**Score**
FCFP_2	565998553	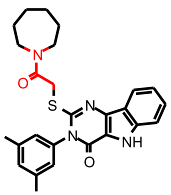 [*]CC(=O)N([*])[*]	0.224

FCFP_2	-1549163031	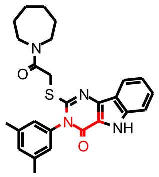 [*]N([*])C(=O)[c](:[*]):[*]	0.078
FCFP_2	332760439	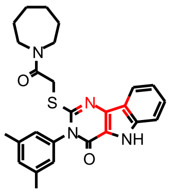 [*][c]1:[*]:[*]:[c](:[*]):[c]:1N=[*]	0.0709
**Top Features for negative contribution**			
**Fingerprint**	**Bit/Smiles**	**Feature Structure**	**Score**
FCFP_2	1	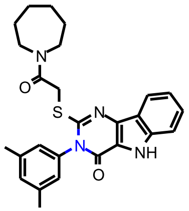 [*]N([*])[*]	-0.306
FCFP_2	0	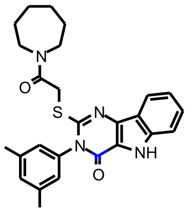 [*]C(=[*])[*]	-0.275
FCFP_2	-1272768868	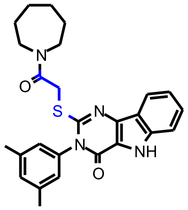 [*]SCC(=[*])[*]	-0.247

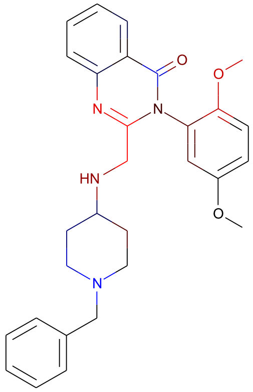

$C_{29}H_{32}N_4O_3$
Molecular Weight: 484.58938
ALogP: 3.743
Rotatable Bonds: 8
Acceptors: 6
Donors: 1

Model Prediction

Prediction: 0.000269
Unit: g/l
Mahalanobis Distance: 16.1
Mahalanobis Distance p-value: 5.57e-024

Mahalanobis Distance: The Mahalanobis distance (MD) is a generalization of the Euclidean distance that accounts for correlations among the X properties. It is calculated as the distance to the center of the training data. The larger the MD, the less trustworthy the prediction.
Mahalanobis Distance p-value: The p-value gives the fraction of training data with an MD greater than or equal to the one for the given sample, assuming normally distributed data. The smaller the p-value, the less trustworthy the prediciton. For highly non-normal X properties (e.g., fingerprints), the MD p-value is wildly inaccurate.

Structural Similar Compounds

Name	Flucythrinate	Diphenylphthalate	Rotenone
Structure	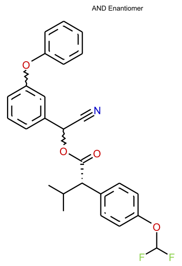	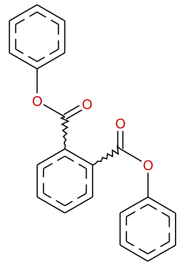	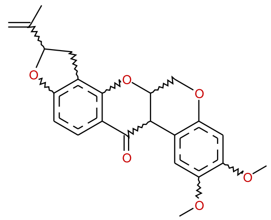
Actual Endpoint (-log C)	9.37572	6.6	7.942
Predicted Endpoint (-log C)	8.49266	6.4642	6.1742
Distance	0.849	0.852	0.889
Reference	DSSTox/EPAFHM	ATOCFM Volume 2	ATOCFM Volume 5

Model Applicability

Unknown features are fingerprint features in the query molecule, but not found or appearing too infrequently in the training set.

1. OPS PC9 out of range. Value: -5.0736. Training min, max, SD, explained variance: -4.4045, 4.4278, 1.418, 0.0346.
2. Unknown FCFP_2 feature: 580453787: [*]C(=N[c](:[*]):[*])[*]

Feature Contribution

Top features for positive contribution

Fingerprint	Bit/Smiles	Feature Structure	Score
FCFP_2	-1272709286	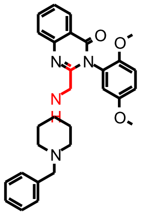 [*]NCC([*])[*]	0.237

FCFP_2	565998553	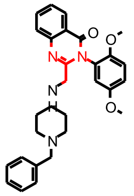 [*]CC(=O)N([*])[*]	0.224
FCFP_2	1036089772	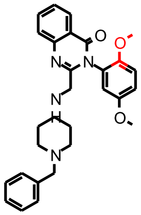 [*]:[c](:[*])OC	0.119

Top Features for negative contribution

Fingerprint	Bit/Smiles	Feature Structure	Score
FCFP_2	1	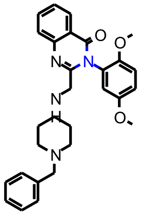 [*]N([*])[*]	-0.306
FCFP_2	0	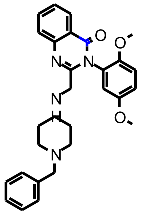 [*]C(=[*])[*]	-0.275
FCFP_2	9	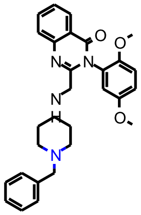 [*]N([*])[*]	-0.268

#UNDEFINED

TOPKAT_Fathead_Minnow_LC50

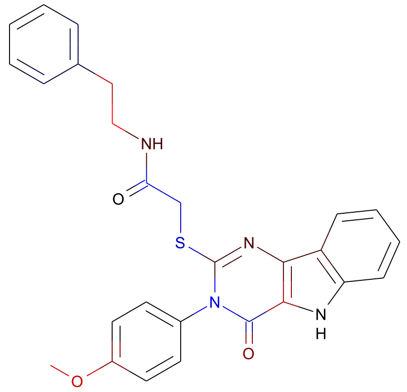

$C_{27}H_{24}N_4O_3S$
Molecular Weight: 484.56946
ALogP: 5.346
Rotatable Bonds: 8
Acceptors: 5
Donors: 2

Model Prediction

Prediction: 1.64e-005
Unit: g/l
Mahalanobis Distance: 15.9
Mahalanobis Distance p-value: 4.42e-023

Mahalanobis Distance: The Mahalanobis distance (MD) is a generalization of the Euclidean distance that accounts for correlations among the X properties. It is calculated as the distance to the center of the training data. The larger the MD, the less trustworthy the prediction.
Mahalanobis Distance p-value: The p-value gives the fraction of training data with an MD greater than or equal to the one for the given sample, assuming normally distributed data. The smaller the p-value, the less trustworthy the prediciton. For highly non-normal X properties (e.g., fingerprints), the MD p-value is wildly inaccurate.

Structural Similar Compounds

Name	Flucythrinate	Fenvalerate (test 2)	Diphenylphthalate
Structure	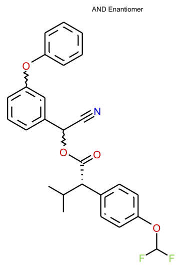	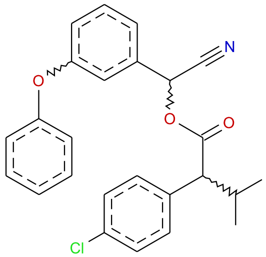	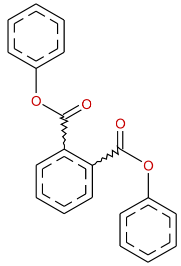
Actual Endpoint (-log C)	9.37572	9	6.6
Predicted Endpoint (-log C)	8.49266	7.86616	6.4642
Distance	1.075	1.121	1.161
Reference	DSSTox/EPAFHM	ATOCFM Volume 4	ATOCFM Volume 2

Model Applicability

Unknown features are fingerprint features in the query molecule, but not found or appearing too infrequently in the training set.

1. Num_AromaticRings out of range. Value: 4. Training min, max, mean, SD: 0, 3, 0.64948, 0.679.
2. OPS PC3 out of range. Value: -6.3899. Training min, max, SD, explained variance: -5.951, 6.9816, 2.181, 0.0819.
3. OPS PC9 out of range. Value: -5.4829. Training min, max, SD, explained variance: -4.4045, 4.4278, 1.418, 0.0346.
4. Unknown FCFP_2 feature: 203707511: [*]C(=[*])[c]1:[nH]:[*]:[*]:[c]:1[*]
5. Unknown FCFP_2 feature: 580453787: [*]C(=N[c](:[*]):[*])[*]

Feature Contribution

Top features for positive contribution

Fingerprint	Bit/Smiles	Feature Structure	Score
FCFP_2	-1272709286	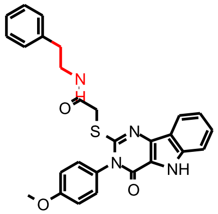 [*]NCC([*])[*]	0.237

FCFP_2	1036089772	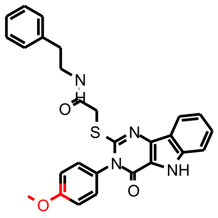 [*]:[c](:[*])OC	0.119
FCFP_2	136627117	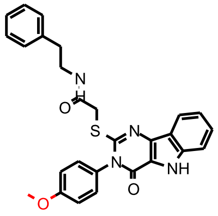 [*]OC	0.0814
**Top Features for negative contribution**			
**Fingerprint**	**Bit/Smiles**	**Feature Structure**	**Score**
FCFP_2	1	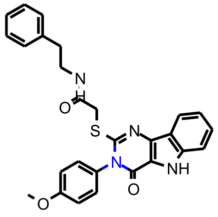 [*]N([*])[*]	-0.306
FCFP_2	0	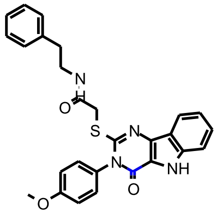 [*]C(=[*])[*]	-0.275
FCFP_2	-1272768868	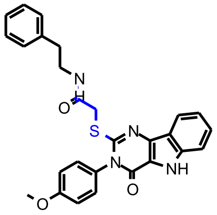 [*]SCC(=[*])[*]	-0.247

Molecule

TOPKAT_Rat_Inhalational_LC50

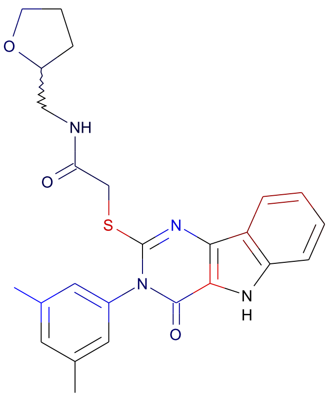

$C_{25}H_{26}N_4O_3S$

Molecular Weight: 462.56393

ALogP: 4.789

Rotatable Bonds: 6

Acceptors: 5

Donors: 2

Model Prediction

Prediction: 5.58e+003

Unit: mg/m3/h

Mahalanobis Distance: 12.8

Mahalanobis Distance p-value: 3.85e-008

Mahalanobis Distance: The Mahalanobis distance (MD) is a generalization of the Euclidean distance that accounts for correlations among the X properties. It is calculated as the distance to the center of the training data. The larger the MD, the less trustworthy the prediction.
Mahalanobis Distance p-value: The p-value gives the fraction of training data with an MD greater than or equal to the one for the given sample, assuming normally distributed data. The smaller the p-value, the less trustworthy the prediciton. For highly non-normal X properties (e.g., fingerprints), the MD p-value is wildly inaccurate.

Structural Similar Compounds

Name	Benzoic acid; 5-(2-chloro-4-(trifluoromethyl)phenoxy)-2-nitro-	Benzamide; N-(((3;5-dichloro-4-(1;1;2;2-tetrafluoroethoxy)phenyl) am ino)carbonyl)-2;6-difluoro-	1H-1;2;4-Triazole-1-ethanamidothioic acid; N-(2;4-dichlorophenyl)-;
Structure	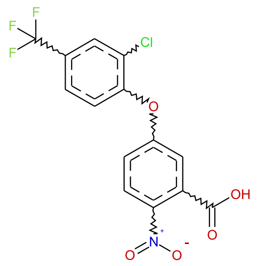	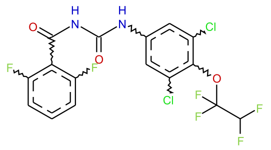	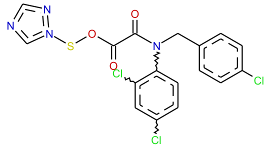
Actual Endpoint (-log C)	1.1174	1.6638	2.0499
Predicted Endpoint (-log C)	1.97855	1.71207	2.8772
Distance	0.819	0.826	0.848
Reference	PEMNDP Pesticide Manual. (The British Crop Protection Council; 20 Bridport R d.; Thornton Heath CR4 7QG; UK) V.1- 1968- Volume(issue)/page/year: 9;6;199 1	PEMNDP Pesticide Manual. (The British Crop Protection Council; 20 Bridport R d.; Thornton Heath CR4 7QG; UK) V.1- 1968- Volume(issue)/page/year: 9;471;1 991	NNGADV Nippon Noyaku Gakkaishi. Journal of the Pesticide Science Society of Japan. (Nippon Noyaku Gakkai; 1-43-11; Komagome; Toshima-ku; Tokyo 170; Japan) V.1- 1976- Volume(issue)/page/year: 20;373;1995

Model Applicability

Unknown features are fingerprint features in the query molecule, but not found or appearing too infrequently in the training set.

1. All properties and OPS components are within expected ranges.
2. Unknown ECFP_2 feature: -782828288: [*]C(=[*])[c]1:[nH]:[*]:[*]:[c]:1[*]
3. Unknown ECFP_2 feature: -962137479: [*][c]1:[*]:[*]:[c](:[*]):[c]:1N=[*]
4. Unknown ECFP_2 feature: -84772164: [*]NCC([*])[*]

Feature Contribution

Top features for positive contribution			
Fingerprint	Bit/Smiles	Feature Structure	Score

ECFP_2	642810091	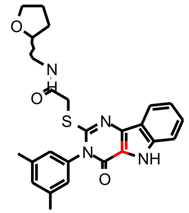 [*][c](:[*]):[*]	0.214
ECFP_2	-830332112	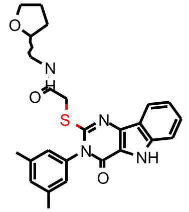 [*]S[*]	0.2
ECFP_2	1996767644	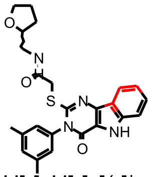 [*]:[cH]:[cH]:[c](:[*]):[*]	0.127

Top Features for negative contribution

**Fingerprint**	**Bit/Smiles**	**Feature Structure**	**Score**
ECFP_2	734603939	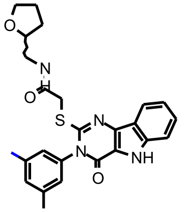 [*]C	-0.302
ECFP_2	655739385	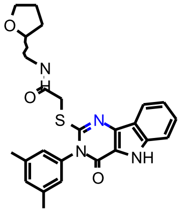 [*]N=[*]	-0.217

ECFP_2	-786013480	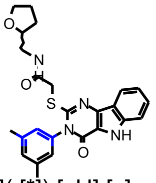 [*][c](:[*]):[cH]:[c]([*]):[*]	-0.206

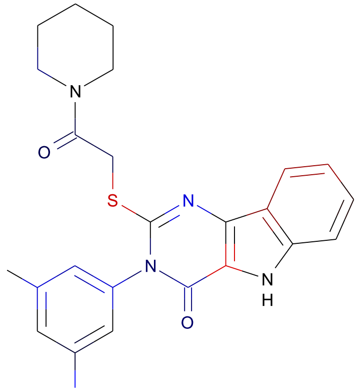

$C_{25}H_{26}N_4O_2S$
Molecular Weight: 446.56453
ALogP: 5.553
Rotatable Bonds: 4
Acceptors: 4
Donors: 1

Model Prediction

Prediction: 6.83e+003
Unit: mg/m3/h
Mahalanobis Distance: 12.5
Mahalanobis Distance p-value: 1.54e-007

Mahalanobis Distance: The Mahalanobis distance (MD) is a generalization of the Euclidean distance that accounts for correlations among the X properties. It is calculated as the distance to the center of the training data. The larger the MD, the less trustworthy the prediction.
Mahalanobis Distance p-value: The p-value gives the fraction of training data with an MD greater than or equal to the one for the given sample, assuming normally distributed data. The smaller the p-value, the less trustworthy the prediciton. For highly non-normal X properties (e.g., fingerprints), the MD p-value is wildly inaccurate.

Structural Similar Compounds

Name	1H-Benzimidazole; 5-chloro-6-(2;3-dichlorophenoxy)-2-(methylthio)-	Ethanone; 2-((4-(2;4-dichloro-3-methylbenzoyl)-1;3-dimethyl-1H-pyra zol-5-yl)oxy)- 1-(4-methylphenyl)-	1;3-Indandione; 2-((p-chlorophenyl)phenylacetyl)-
Structure	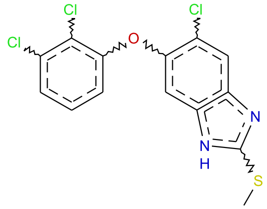	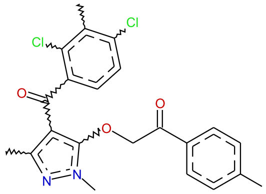	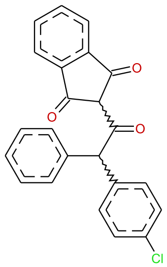
Actual Endpoint (-log C)	2.2548	1.7472	2.0967
Predicted Endpoint (-log C)	1.69815	2.15944	1.99523
Distance	0.651	0.695	0.761
Reference	MDACAP Medicamentos de Actualidad. (J.R. Prous; S.A.; Apartado de Correos 54 0; 08080 Barcelona; Spain) V.1-1965- Volume(issue)/page/year: 21;227;1985	NNGADV Nippon Noyaku Gakkaishi. Journal of the Pesticide Science Society of Japan. (Nippon Noyaku Gakkai; 1-43-11; Komagome; Toshima-ku; Tokyo 170; Japan) V.1-1976- Volume(issue)/page/year: 15;125;1990	85JFAN "Agrochemicals Handbook;" with updates; Hartley; D.; and H. Kidd; eds. ; Nottingham; Royal Soc. of Chemistry; 1983-86 Volume(issue)/page/year: A084;198 3

Model Applicability

Unknown features are fingerprint features in the query molecule, but not found or appearing too infrequently in the training set.

1. All properties and OPS components are within expected ranges.
2. Unknown ECFP_2 feature: -782828288: [*]C(=[*])[c]1:[nH]:[*]:[*]:[c]:1[*]
3. Unknown ECFP_2 feature: -962137479: [*][c]1:[*]:[*]:[c](:[*]):[c]:1N=[*]

Feature Contribution

Top features for positive contribution			
Fingerprint	Bit/Smiles	Feature Structure	Score

ECFP_2	642810091	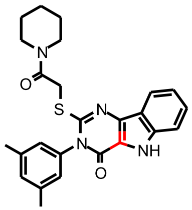 [*][c](:[*]):[*]	0.214
ECFP_2	-830332112	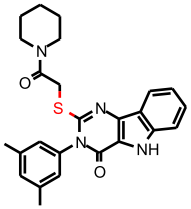 [*]S[*]	0.2
ECFP_2	1996767644	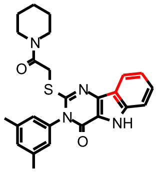 [*]:[cH]:[cH]:[c](:[*]):[*]	0.127

Top Features for negative contribution

Fingerprint	Bit/Smiles	Feature Structure	Score
ECFP_2	734603939	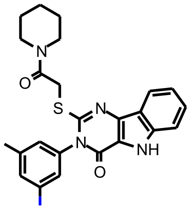 [*]C	-0.302
ECFP_2	655739385	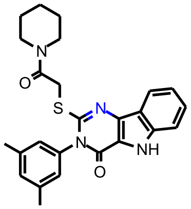 [*]N=[*]	-0.217

ECFP_2	-786013480	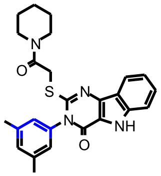 [*][c](:[*]):[cH]:[c]([*]):[*]	-0.206

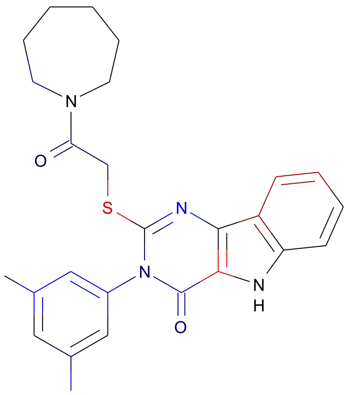

$C_{26}H_{28}N_4O_2S$

Molecular Weight: 460.59111

ALogP: 6.009

Rotatable Bonds: 4

Acceptors: 4

Donors: 1

Model Prediction

Prediction: 6.47e+003

Unit: mg/m3/h

Mahalanobis Distance: 12.6

Mahalanobis Distance p-value: 7.66e-008

Mahalanobis Distance: The Mahalanobis distance (MD) is a generalization of the Euclidean distance that accounts for correlations among the X properties. It is calculated as the distance to the center of the training data. The larger the MD, the less trustworthy the prediction.

Mahalanobis Distance p-value: The p-value gives the fraction of training data with an MD greater than or equal to the one for the given sample, assuming normally distributed data. The smaller the p-value, the less trustworthy the prediciton. For highly non-normal X properties (e.g., fingerprints), the MD p-value is wildly inaccurate.

Structural Similar Compounds

Name	1H-Benzimidazole; 5-chloro-6-(2;3-dichlorophenoxy)-2-(methylthio)-	Ethanone; 2-((4-(2;4-dichloro-3-methylbenzoyl)-1;3-dimethyl-1H-pyra zol-5-yl)oxy)- 1-(4-methylphenyl)-	1H-1;2;4-Triazole-1-ethanamidothioic acid; N-(2;4-dichlorophenyl)-;
Structure	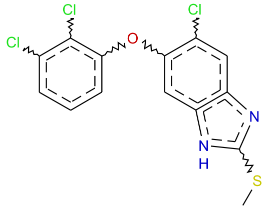	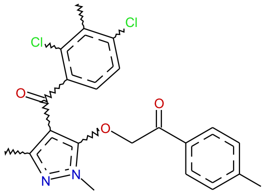	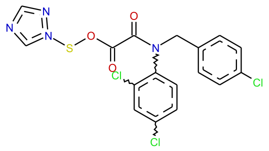
Actual Endpoint (-log C)	2.2548	1.7472	2.0499
Predicted Endpoint (-log C)	1.69815	2.15944	2.8772
Distance	0.662	0.697	0.777
Reference	MDACAP Medicamentos de Actualidad. (J.R. Prous; S.A.; Apartado de Correos 54 0; 08080 Barcelona; Spain) V.1-1965- Volume(issue)/page/year: 21;227;1985	NNGADV Nippon Noyaku Gakkaishi. Journal of the Pesticide Science Society of Japan. (Nippon Noyaku Gakkai; 1-43-11; Komagome; Toshima-ku; Tokyo 170; Japan) V.1-1976- Volume(issue)/page/year: 15;125;1990	NNGADV Nippon Noyaku Gakkaishi. Journal of the Pesticide Science Society of Japan. (Nippon Noyaku Gakkai; 1-43-11; Komagome; Toshima-ku; Tokyo 170; Japan) V.1-1976- Volume(issue)/page/year: 20;373;1995

Model Applicability

Unknown features are fingerprint features in the query molecule, but not found or appearing too infrequently in the training set.

1. All properties and OPS components are within expected ranges.
2. Unknown ECFP_2 feature: -782828288: [*]C(=[*])[c]1:[nH]:[*]:[*]:[c]:1[*]
3. Unknown ECFP_2 feature: -962137479: [*][c]1:[*]:[*]:[c](:[*]):[c]:1N=[*]

Feature Contribution

Top features for positive contribution

Fingerprint	Bit/Smiles	Feature Structure	Score

ECFP_2	642810091	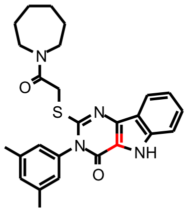 [*][c](:[*]):[*]	0.214
ECFP_2	-830332112	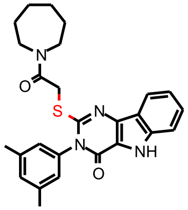 [*]S[*]	0.2
ECFP_2	1996767644	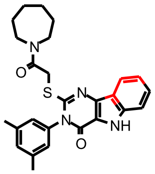 [*]:[cH]:[cH]:[c](:[*]):[*]	0.127

Top Features for negative contribution

Fingerprint	Bit/Smiles	Feature Structure	Score
ECFP_2	734603939	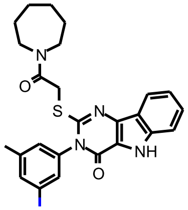 [*]C	-0.302
ECFP_2	655739385	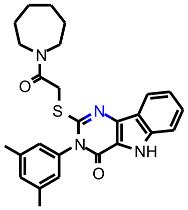 [*]N=[*]	-0.217

ECFP_2	-786013480	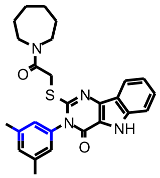 [*][c](:[*]):[cH]:[c]([*]):[*]	-0.206

#UNDEFINED

TOPKAT_Rat_Inhalational_LC50

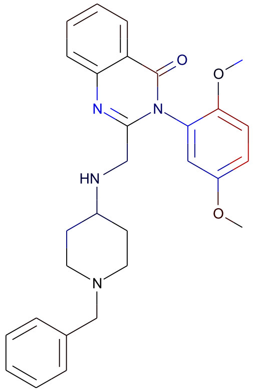

$C_{29}H_{32}N_4O_3$

Molecular Weight: 484.58938

ALogP: 3.743

Rotatable Bonds: 8

Acceptors: 6

Donors: 1

Model Prediction

Prediction: 7.08e+003

Unit: mg/m3/h

Mahalanobis Distance: 13.5

Mahalanobis Distance p-value: 2.75e-010

Mahalanobis Distance: The Mahalanobis distance (MD) is a generalization of the Euclidean distance that accounts for correlations among the X properties. It is calculated as the distance to the center of the training data. The larger the MD, the less trustworthy the prediction.
Mahalanobis Distance p-value: The p-value gives the fraction of training data with an MD greater than or equal to the one for the given sample, assuming normally distributed data. The smaller the p-value, the less trustworthy the prediciton. For highly non-normal X properties (e.g., fingerprints), the MD p-value is wildly inaccurate.

Structural Similar Compounds

Name	**Benzoic acid; 4-(((((1;3-dimethyl-5-phenoxy-1H-pyrazol-4-yl)methyle ne)amino)oxy)methyl)-; 1;1-dimethylethyl ester; (E)-**	**1H-1;2;4-Triazole; 1-((2-(2-chloro-4-(4-chlorophenoxy)phenyl)-4-met hyl-1;3-dioxolan-2-yl)methyl)-**	**Propanoic acid; 2-(4-((6-chloro-2-quinoxalinyl)oxy)phenoxy)-; 2-(((1-methylethylidene)amino) oxy)ethyl ester; (R)-**
Structure	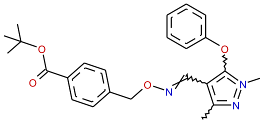	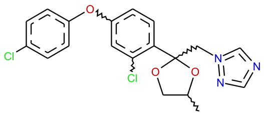	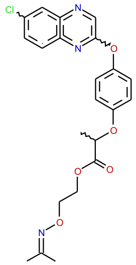
Actual Endpoint (-log C)	2.5042	3.3535	1.6473
Predicted Endpoint (-log C)	2.56205	1.62735	1.81634
Distance	0.687	0.735	0.768
Reference	NNGADV Nippon Noyaku Gakkaishi. Journal of the Pesticide Science Society of Japan. (Nippon Noyaku Gakkai; 1-43-11; Komagome; Toshima-ku; Tokyo 170; Japan) V.1- 1976- Volume(issue)/page/year: 17;S261;199	PEMNDP Pesticide Manual. (The British Crop Protection Council; 20 Bridport R d.; Thornton Heath CR4 7QG; UK) V.1- 1968- Volume(issue)/page/year: 9;277;1 991	PEMNDP Pesticide Manual. (The British Crop Protection Council; 20 Bridport R d.; Thornton Heath CR4 7QG; UK) V.1- 1968- Volume(issue)/page/year: 9;718;1 991

Model Applicability

Unknown features are fingerprint features in the query molecule, but not found or appearing too infrequently in the training set.

1. OPS PC19 out of range. Value: 3.7056. Training min, max, SD, explained variance: -3.716, 3.6677, 1.125, 0.0176.
2. Unknown ECFP_2 feature: -1073177635: [*]C\C(=N\[*])\N([*])[*]
3. Unknown ECFP_2 feature: -597295171: [*][c](:[*]):[c](:[cH]:[*])N=[*]
4. Unknown ECFP_2 feature: 769925792: [*]NCC(=[*])[*]
5. Unknown ECFP_2 feature: 496787418: [*]CNC([*])[*]

Feature Contribution

Top features for positive contribution

Fingerprint	Bit/Smiles	Feature Structure	Score
ECFP_2	642810091	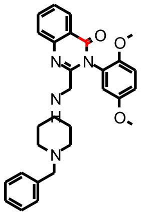 [*][c](:[*]):[*]	0.214
ECFP_2	1996767644	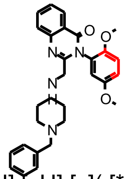 [*]:[cH]:[cH]:[c](:[*]):[*]	0.127
ECFP_2	-176455838	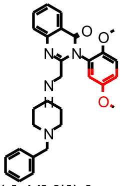 [*]O[c](:[cH]:[*]):[cH]:[*]	0.047

Top Features for negative contribution

Fingerprint	Bit/Smiles	Feature Structure	Score
ECFP_2	734603939	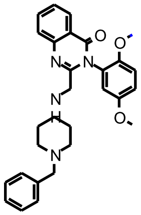 [*]C	-0.302

ECFP_2	655739385	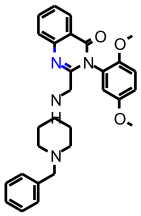 [*]N=[*]	-0.217
ECFP_2	-786013480	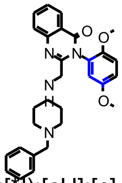 [*][c](:[*]):[cH]:[c]([*]):[*]	-0.206

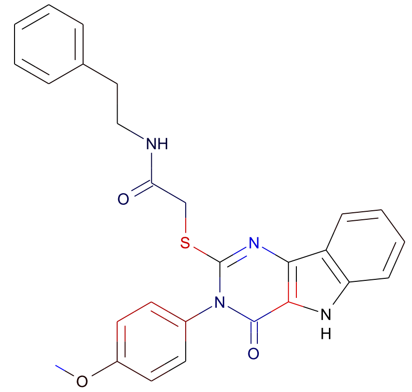

$C_{27}H_{24}N_4O_3S$

Molecular Weight: 484.56946

ALogP: 5.346

Rotatable Bonds: 8

Acceptors: 5

Donors: 2

Model Prediction

Prediction: 1.62e+003

Unit: mg/m3/h

Mahalanobis Distance: 14.1

Mahalanobis Distance p-value: 6.5e-012

Mahalanobis Distance: The Mahalanobis distance (MD) is a generalization of the Euclidean distance that accounts for correlations among the X properties. It is calculated as the distance to the center of the training data. The larger the MD, the less trustworthy the prediction.

Mahalanobis Distance p-value: The p-value gives the fraction of training data with an MD greater than or equal to the one for the given sample, assuming normally distributed data. The smaller the p-value, the less trustworthy the prediciton. For highly non-normal X properties (e.g., fingerprints), the MD p-value is wildly inaccurate.

Structural Similar Compounds

Name	DL-Valine; N-(2-chloro-4-(trifluoromethyl)phenyl)-; cyano(3-phenoxy phenyl)methyl ester	1H-1;2;4-Triazole-1-ethanamidothioic acid; N-(2;4-dichlorophenyl)-;	Benzoic acid; 4-(((((1;3-dimethyl-5-phenoxy-1H-pyrazol-4-yl)methyle ne)amino)oxy)methyl)-; 1;1-dimethylethyl ester; (E)-
Structure	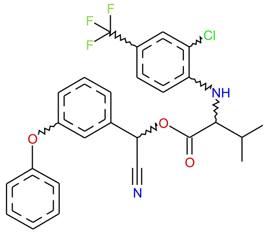	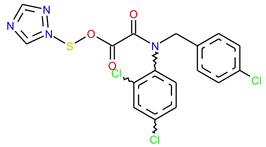	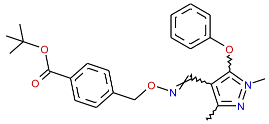
Actual Endpoint (-log C)	2.457	2.0499	2.5042
Predicted Endpoint (-log C)	1.74061	2.8772	2.56205
Distance	0.862	0.891	0.940
Reference	NNGADV Nippon Noyaku Gakkaishi. Journal of the Pesticide Science Society of Japan. (Nippon Noyaku Gakkai; 1-43-11; Komagome; Toshima-ku; Tokyo 170; Japan) V.1-1976- Volume(issue)/page/year: 15;121;1990	NNGADV Nippon Noyaku Gakkaishi. Journal of the Pesticide Science Society of Japan. (Nippon Noyaku Gakkai; 1-43-11; Komagome; Toshima-ku; Tokyo 170; Japan) V.1-1976- Volume(issue)/page/year: 20;373;1995	NNGADV Nippon Noyaku Gakkaishi. Journal of the Pesticide Science Society of Japan. (Nippon Noyaku Gakkai; 1-43-11; Komagome; Toshima-ku; Tokyo 170; Japan) V.1-1976- Volume(issue)/page/year: 17;S261;199

Model Applicability

Unknown features are fingerprint features in the query molecule, but not found or appearing too infrequently in the training set.

1. Num_AromaticRings out of range. Value: 4. Training min, max, mean, SD: 0, 3, 0.57958, 0.795.
2. Unknown ECFP_2 feature: -782828288: [*]C(=[*])[c]1:[nH]:[*]:[*]:[c]:1[*]
3. Unknown ECFP_2 feature: -962137479: [*][c]1:[*]:[*]:[c](:[*]):[c]:1N=[*]

Feature Contribution

Top features for positive contribution			
Fingerprint	Bit/Smiles	Feature Structure	Score

ECFP_2	642810091	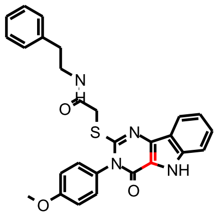 [*][c](:[*]):[*]	0.214
ECFP_2	-830332112	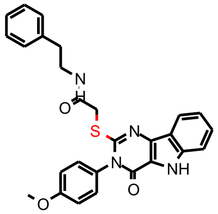 [*]S[*]	0.2
ECFP_2	1996767644	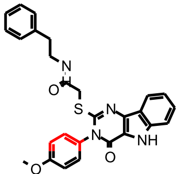 [*]:[cH]:[cH]:[c](:[*]):[*]	0.127
**Top Features for negative contribution**			
**Fingerprint**	**Bit/Smiles**	**Feature Structure**	**Score**
ECFP_2	734603939	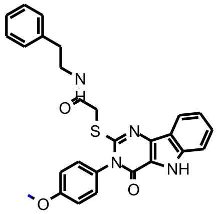 [*]C	-0.302
ECFP_2	655739385	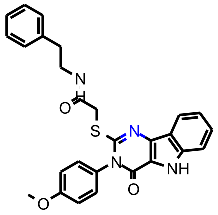 [*]N=[*]	-0.217

ECFP_2	2106656448	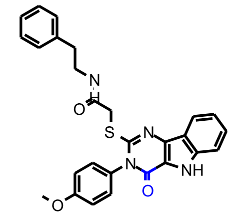<br>[*]C(=O)[*]	-0.151

Molecule

TOPKAT_Rat_Maximum_Tolerated_Dose_Feed

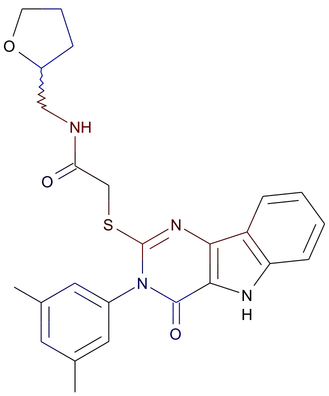

$C_{25}H_{26}N_4O_3S$

Molecular Weight: 462.56393

ALogP: 4.789

Rotatable Bonds: 6

Acceptors: 5

Donors: 2

Model Prediction

Prediction: 0.0644

Unit: g/kg_body_weight

Mahalanobis Distance: 11.6

Mahalanobis Distance p-value: 6.12e-008

Mahalanobis Distance: The Mahalanobis distance (MD) is a generalization of the Euclidean distance that accounts for correlations among the X properties. It is calculated as the distance to the center of the training data. The larger the MD, the less trustworthy the prediction.
Mahalanobis Distance p-value: The p-value gives the fraction of training data with an MD greater than or equal to the one for the given sample, assuming normally distributed data. The smaller the p-value, the less trustworthy the prediciton. For highly non-normal X properties (e.g., fingerprints), the MD p-value is wildly inaccurate.

Structural Similar Compounds

Name	C.I.PIGMENT RED 3	PHENOLPHTHALEIN	RESERPINE
Structure	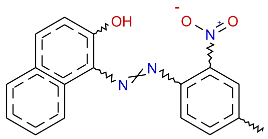	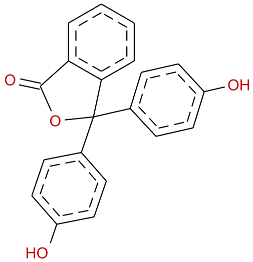	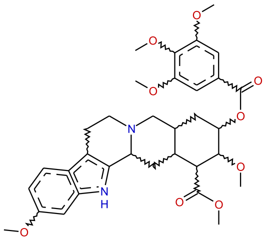
Actual Endpoint (-log C)	2.65635	2.20184	6.13118
Predicted Endpoint (-log C)	2.97957	2.8857	4.38304
Distance	0.705	0.764	0.810
Reference	NCI/NTP TR-407	NCI/NTP TR-465	NCI/NTP TR-193

Model Applicability

Unknown features are fingerprint features in the query molecule, but not found or appearing too infrequently in the training set.

1. OPS PC12 out of range. Value: -2.433. Training min, max, SD, explained variance: -2.364, 2.9228, 1.079, 0.0263.

Feature Contribution

Top features for positive contribution

Fingerprint	Bit/Smiles	Feature Structure	Score
FCFP_2	-885550502	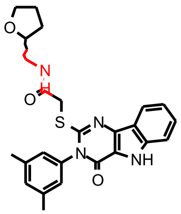 [*]CNC(=[*])[*]	0.115

FCFP_2	-1143715940	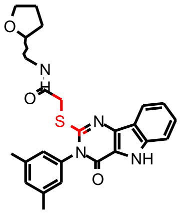 [*]CSC(=[*])[*]	0.095
FCFP_2	3	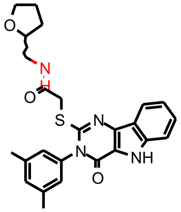 [*]N[*]	0.0737
**Top Features for negative contribution**			
**Fingerprint**	**Bit/Smiles**	**Feature Structure**	**Score**
FCFP_2	-1272798659	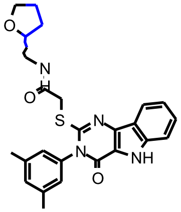 [*]C1[*][*]CC1	-0.111
FCFP_2	1872154524	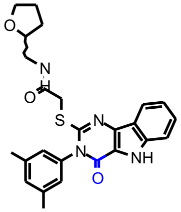 [*]C(=O)[*]	-0.105
FCFP_2	203677720	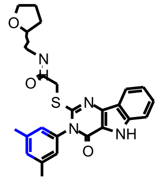 [*]:[cH]:[c](C):[cH]:[*]	-0.0829

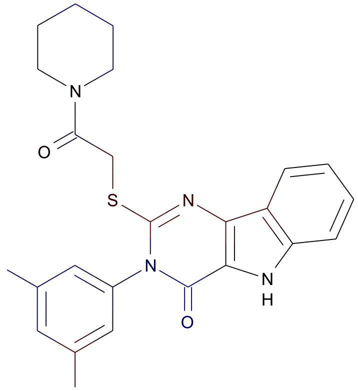

$C_{25}H_{26}N_4O_2S$
Molecular Weight: 446.56453
ALogP: 5.553
Rotatable Bonds: 4
Acceptors: 4
Donors: 1

Model Prediction

Prediction: 0.0445
Unit: g/kg_body_weight
Mahalanobis Distance: 11.2
Mahalanobis Distance p-value: 4.28e-007

Mahalanobis Distance: The Mahalanobis distance (MD) is a generalization of the Euclidean distance that accounts for correlations among the X properties. It is calculated as the distance to the center of the training data. The larger the MD, the less trustworthy the prediction.
Mahalanobis Distance p-value: The p-value gives the fraction of training data with an MD greater than or equal to the one for the given sample, assuming normally distributed data. The smaller the p-value, the less trustworthy the prediciton. For highly non-normal X properties (e.g., fingerprints), the MD p-value is wildly inaccurate.

Structural Similar Compounds

Name	C.I.PIGMENT RED 3	PHENOLPHTHALEIN	4,4'-THIOBIS(6-t-BUTYL-m-CRESOL)
Structure	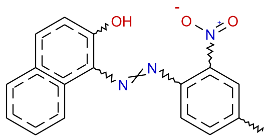	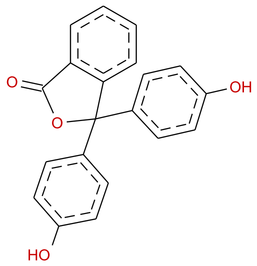	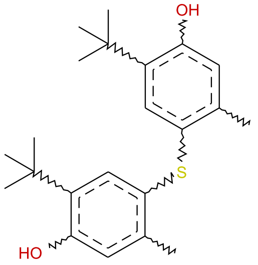
Actual Endpoint (-log C)	2.65635	2.20184	3.55454
Predicted Endpoint (-log C)	2.97957	2.8857	3.06707
Distance	0.595	0.682	0.737
Reference	NCI/NTP TR-407	NCI/NTP TR-465	NCI/NTP TR-435

Model Applicability

Unknown features are fingerprint features in the query molecule, but not found or appearing too infrequently in the training set.

1. All properties and OPS components are within expected ranges.

Feature Contribution

Top features for positive contribution

Fingerprint	Bit/Smiles	Feature Structure	Score
FCFP_2	-1143715940	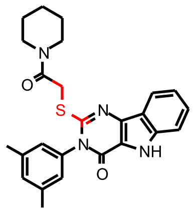 [*]CSC(=[*])[*]	0.095

FCFP_2	136120670	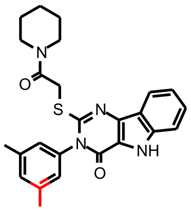 [*]:[c](:[*])C	0.064
FCFP_2	332760439	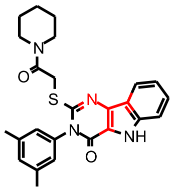 [*][c]1:[*]:[*]:[c](:[*]):[c]:1N=[*]	0.0611
**Top Features for negative contribution**			
**Fingerprint**	**Bit/Smiles**	**Feature Structure**	**Score**
FCFP_2	-1272798659	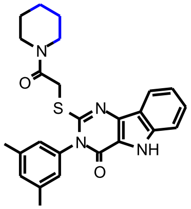 [*]C1[*][*]CC1	-0.111
FCFP_2	1872154524	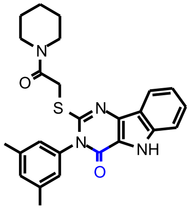 [*]C(=O)[*]	-0.105
FCFP_2	203677720	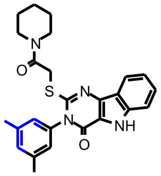 [*]:[cH]:[c](C):[cH]:[*]	-0.0829

#UNDEFINED

TOPKAT_Rat_Maximum_Tolerated_Dose_Feed

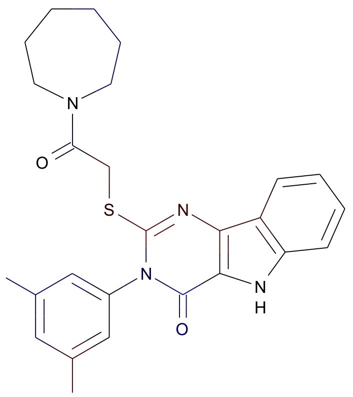

$C_{26}H_{28}N_4O_2S$
Molecular Weight: 460.59111
ALogP: 6.009
Rotatable Bonds: 4
Acceptors: 4
Donors: 1

Model Prediction

Prediction: 0.042
Unit: g/kg_body_weight
Mahalanobis Distance: 11.2
Mahalanobis Distance p-value: 3.83e-007

Mahalanobis Distance: The Mahalanobis distance (MD) is a generalization of the Euclidean distance that accounts for correlations among the X properties. It is calculated as the distance to the center of the training data. The larger the MD, the less trustworthy the prediction.
Mahalanobis Distance p-value: The p-value gives the fraction of training data with an MD greater than or equal to the one for the given sample, assuming normally distributed data. The smaller the p-value, the less trustworthy the prediciton. For highly non-normal X properties (e.g., fingerprints), the MD p-value is wildly inaccurate.

Structural Similar Compounds

Name	C.I.PIGMENT RED 3	PHENOLPHTHALEIN	4,4'-THIOBIS(6-t-BUTYL-m-CRESOL)
Structure	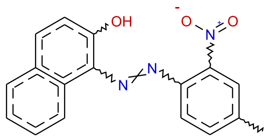	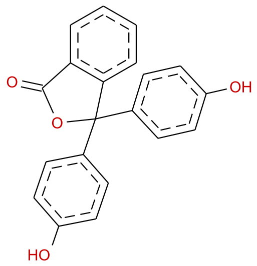	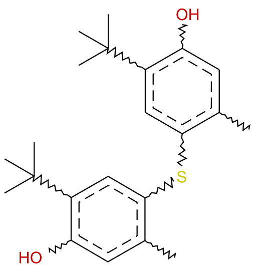
Actual Endpoint (-log C)	2.65635	2.20184	3.55454
Predicted Endpoint (-log C)	2.97957	2.8857	3.06707
Distance	0.623	0.715	0.733
Reference	NCI/NTP TR-407	NCI/NTP TR-465	NCI/NTP TR-435

Model Applicability

Unknown features are fingerprint features in the query molecule, but not found or appearing too infrequently in the training set.

1. All properties and OPS components are within expected ranges.

Feature Contribution

Top features for positive contribution

Fingerprint	Bit/Smiles	Feature Structure	Score
FCFP_2	-1143715940	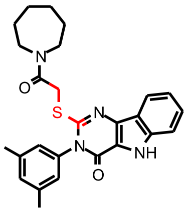 [*]CSC(=[*])[*]	0.095

FCFP_2	136120670	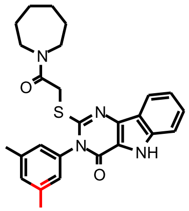 [*]:[c](:[*])C	0.064
FCFP_2	332760439	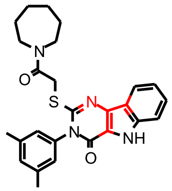 [*][c]1:[*]:[*]:[c](:[*]):[c]:1N=[*]	0.0611
**Top Features for negative contribution**			
**Fingerprint**	**Bit/Smiles**	**Feature Structure**	**Score**
FCFP_2	-1272798659	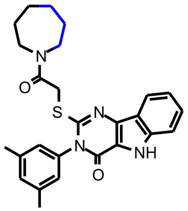 [*]C1[*][*]CC1	-0.111
FCFP_2	1872154524	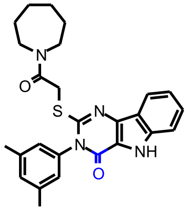 [*]C(=O)[*]	-0.105
FCFP_2	203677720	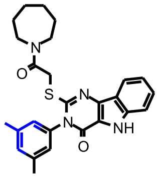 [*]:[cH]:[c](C):[cH]:[*]	-0.0829

#UNDEFINED

TOPKAT_Rat_Maximum_Tolerated_Dose_Feed

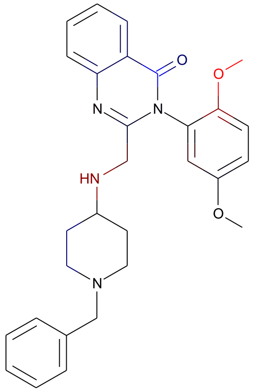

$C_{29}H_{32}N_4O_3$
Molecular Weight: 484.58938
ALogP: 3.743
Rotatable Bonds: 8
Acceptors: 6
Donors: 1

Structural Similar Compounds

Name	RESERPINE	ROTENONE	3,3'-DIMETHOXYBENZIDINE-4,4'-DIISOCYANATE
Structure	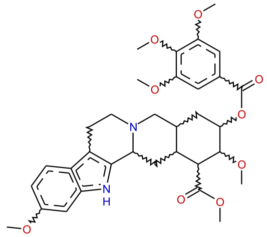	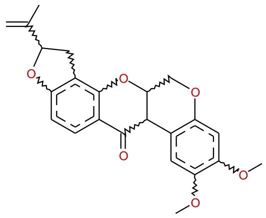	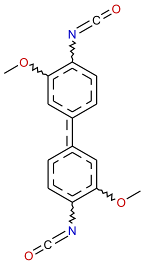
Actual Endpoint (-log C)	6.13118	5.06769	2.17504
Predicted Endpoint (-log C)	4.38304	4.11907	3.78717
Distance	0.752	0.756	0.765
Reference	NCI/NTP TR-193	NCI/NTP TR-320	NCI/NTP TR-128

Model Prediction

Prediction: 0.0371
Unit: g/kg_body_weight
Mahalanobis Distance: 8.72
Mahalanobis Distance p-value: 0.00338

Mahalanobis Distance: The Mahalanobis distance (MD) is a generalization of the Euclidean distance that accounts for correlations among the X properties. It is calculated as the distance to the center of the training data. The larger the MD, the less trustworthy the prediction.
Mahalanobis Distance p-value: The p-value gives the fraction of training data with an MD greater than or equal to the one for the given sample, assuming normally distributed data. The smaller the p-value, the less trustworthy the prediciton. For highly non-normal X properties (e.g., fingerprints), the MD p-value is wildly inaccurate.

Model Applicability

Unknown features are fingerprint features in the query molecule, but not found or appearing too infrequently in the training set.

1. OPS PC11 out of range. Value: 3.941. Training min, max, SD, explained variance: -3.8346, 3.8752, 1.233, 0.0343.
2. Unknown FCFP_2 feature: 906798516: [*]N([*])C[c](:[*]):[*]

Feature Contribution

Top features for positive contribution			
**Fingerprint**	**Bit/Smiles**	**Feature Structure**	**Score**
FCFP_2	136627117	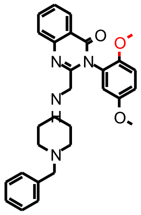 [*]OC	0.173

FCFP_2	-885550502	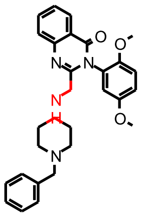 [*]CNC(=[*])[*]	0.115
FCFP_2	1036089772	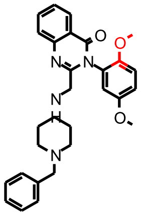 [*]:[c](:[*])OC	0.0749

Top Features for negative contribution

Fingerprint	Bit/Smiles	Feature Structure	Score
FCFP_2	-1272798659	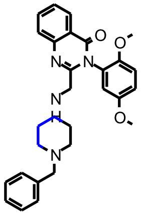 [*]C1[*][*]CC1	-0.111
FCFP_2	1872154524	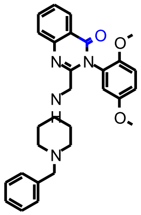 [*]C(=O)[*]	-0.105
FCFP_2	203677720	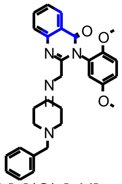 [*]:[cH]:[c](C):[cH]:[*]	-0.0829

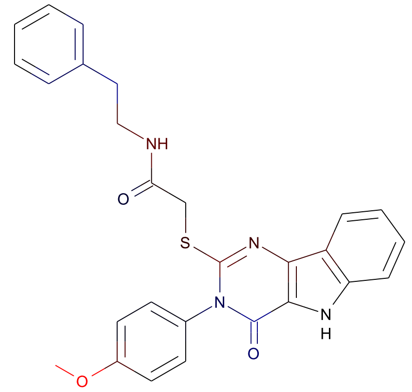

$C_{27}H_{24}N_4O_3S$
Molecular Weight: 484.56946
ALogP: 5.346
Rotatable Bonds: 8
Acceptors: 5
Donors: 2

Model Prediction

Prediction: 0.0473
Unit: g/kg_body_weight
Mahalanobis Distance: 12.2
Mahalanobis Distance p-value: 5.6e-009

Mahalanobis Distance: The Mahalanobis distance (MD) is a generalization of the Euclidean distance that accounts for correlations among the X properties. It is calculated as the distance to the center of the training data. The larger the MD, the less trustworthy the prediction.
Mahalanobis Distance p-value: The p-value gives the fraction of training data with an MD greater than or equal to the one for the given sample, assuming normally distributed data. The smaller the p-value, the less trustworthy the prediciton. For highly non-normal X properties (e.g., fingerprints), the MD p-value is wildly inaccurate.

Structural Similar Compounds

Name	RESERPINE	C.I.PIGMENT RED 3	C.I.PIGMENT RED 23
Structure	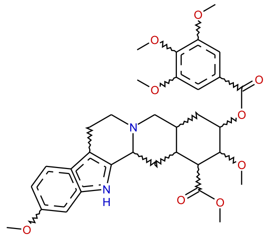	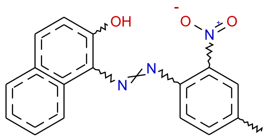	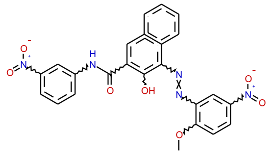
Actual Endpoint (-log C)	6.13118	2.65635	2.30052
Predicted Endpoint (-log C)	4.38304	2.97957	3.55333
Distance	0.786	0.858	0.868
Reference	NCI/NTP TR-193	NCI/NTP TR-407	NCI/NTP TR-411

Model Applicability

Unknown features are fingerprint features in the query molecule, but not found or appearing too infrequently in the training set.

1. All properties and OPS components are within expected ranges.

Feature Contribution

Top features for positive contribution

Fingerprint	Bit/Smiles	Feature Structure	Score
FCFP_2	136627117	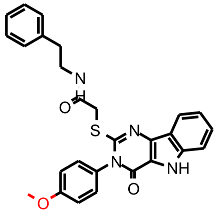 [*]OC	0.173

FCFP_2	-885550502	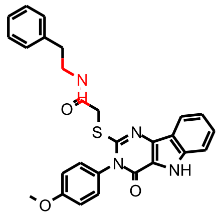 [*]CNC(=[*])[*]	0.115
FCFP_2	-1143715940	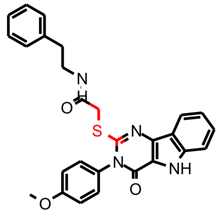 [*]CSC(=[*])[*]	0.095

Top Features for negative contribution

Fingerprint	Bit/Smiles	Feature Structure	Score
FCFP_2	1872154524	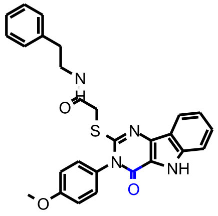 [*]C(=O)[*]	-0.105
FCFP_2	203677720	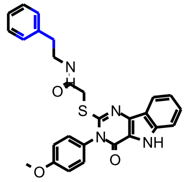 [*]:[cH]:[c](C):[cH]:[*]	-0.0829
FCFP_2	1	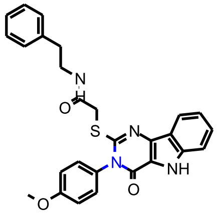 [*]N([*])[*]	-0.0796

Molecule

TOPKAT_Rat_Maximum_Tolerated_Dose_Gavage

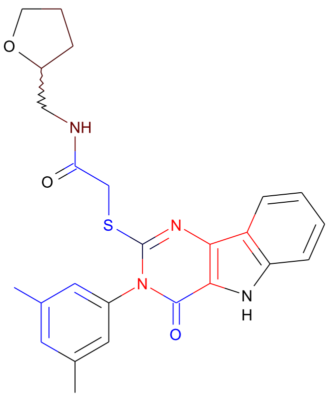

$C_{25}H_{26}N_4O_3S$

Molecular Weight: 462.56393

ALogP: 4.789

Rotatable Bonds: 6

Acceptors: 5

Donors: 2

Model Prediction

Prediction: 0.00445

Unit: g/kg_body_weight

Mahalanobis Distance: 15.4

Mahalanobis Distance p-value: 1.66e-013

Mahalanobis Distance: The Mahalanobis distance (MD) is a generalization of the Euclidean distance that accounts for correlations among the X properties. It is calculated as the distance to the center of the training data. The larger the MD, the less trustworthy the prediction.
Mahalanobis Distance p-value: The p-value gives the fraction of training data with an MD greater than or equal to the one for the given sample, assuming normally distributed data. The smaller the p-value, the less trustworthy the prediciton. For highly non-normal X properties (e.g., fingerprints), the MD p-value is wildly inaccurate.

Structural Similar Compounds

Name	OCHRATOXIN	PENICILLIN VK	SULFISOOXAZOLE
Structure	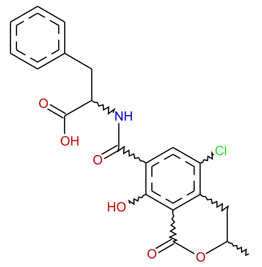	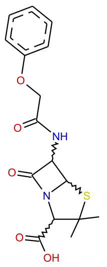	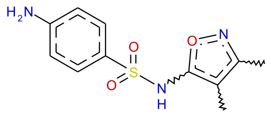
Actual Endpoint (-log C)	6.28396	2.54455	2.82494
Predicted Endpoint (-log C)	5.12358	3.9702	3.0705
Distance	0.786	1.052	1.065
Reference	NCI/NTP TR-358	NCI/NTP TR-336	NCI/NTP TR-138

Model Applicability

Unknown features are fingerprint features in the query molecule, but not found or appearing too infrequently in the training set.

1. Molecular_Weight out of range. Value: 462.56. Training min, max, mean, SD: 68.074, 434.63, 171.13, 85.06.
2. Num_AromaticRings out of range. Value: 3. Training min, max, mean, SD: 0, 2, 0.5625, 0.693.
3. OPS PC6 out of range. Value: -2.7218. Training min, max, SD, explained variance: -2.4321, 2.9885, 1.256, 0.0488.
4. Unknown FCFP_2 feature: 19: [*]:[nH]:[*]
5. Unknown FCFP_2 feature: 203707511: [*]C(=[*])[c]1:[nH]:[*]:[*]:[c]:1[*]
6. Unknown FCFP_2 feature: -1986158408: [*]S\C(=N\[*])\N([*])[*]
7. Unknown FCFP_2 feature: 580453787: [*]C(=N[c](:[*]):[*])[*]
8. Unknown FCFP_2 feature: 2005402822: [*][c]1:[*]:[*]:[c](:[*]):[nH]:1
9. Unknown FCFP_2 feature: 307448885: [*]:[cH]:[c]1:[nH]:[*]:[*]:[c]:1:[*]

Feature Contribution

Top features for positive contribution			
Fingerprint	Bit/Smiles	Feature Structure	Score

FCFP_2	332760439	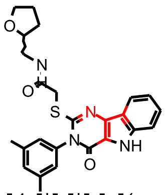 [*][c]1:[*]:[*]:[c](:[*]):[c]:1N=[*]	0.672
FCFP_2	1	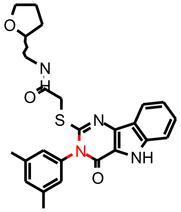 [*]N([*])[*]	0.511
FCFP_2	3	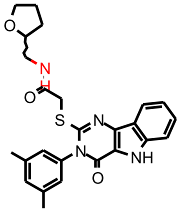 [*]N[*]	0.104
**Top Features for negative contribution**			
**Fingerprint**	**Bit/Smiles**	**Feature Structure**	**Score**
FCFP_2	203677720	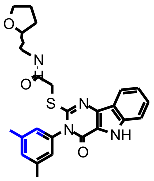 [*]:[cH]:[c](C):[cH]:[*]	-0.406
FCFP_2	1872154524	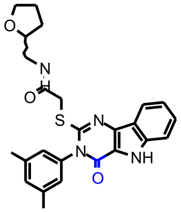 [*]C(=O)[*]	-0.307

FCFP_2	0	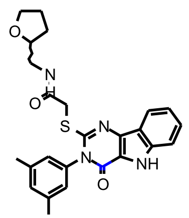 [*]C(=[*])[*]	-0.29

#UNDEFINED

TOPKAT_Rat_Maximum_Tolerated_Dose_Gavage

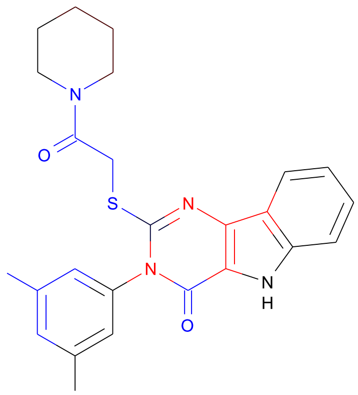

$C_{25}H_{26}N_4O_2S$

Molecular Weight: 446.56453

ALogP: 5.553

Rotatable Bonds: 4

Acceptors: 4

Donors: 1

Model Prediction

Prediction: 0.0126

Unit: g/kg_body_weight

Mahalanobis Distance: 13

Mahalanobis Distance p-value: 3.54e-010

Mahalanobis Distance: The Mahalanobis distance (MD) is a generalization of the Euclidean distance that accounts for correlations among the X properties. It is calculated as the distance to the center of the training data. The larger the MD, the less trustworthy the prediction.
Mahalanobis Distance p-value: The p-value gives the fraction of training data with an MD greater than or equal to the one for the given sample, assuming normally distributed data. The smaller the p-value, the less trustworthy the prediciton. For highly non-normal X properties (e.g., fingerprints), the MD p-value is wildly inaccurate.

Structural Similar Compounds

Name	PHENYLBUTAZONE	OCHRATOXIN	PROMETHAZINE.HCL
Structure	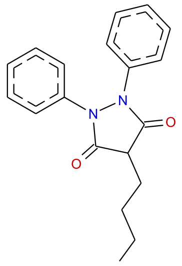	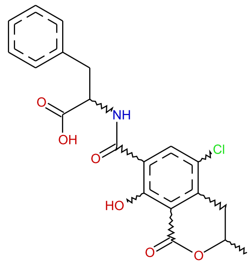	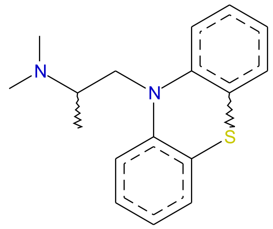
Actual Endpoint (-log C)	3.48909	6.28396	3.93152
Predicted Endpoint (-log C)	3.17333	5.12358	4.72433
Distance	0.972	1.047	1.068
Reference	NCI/NTP TR-367	NCI/NTP TR-358	NCI/NTP TR-425

Model Applicability

Unknown features are fingerprint features in the query molecule, but not found or appearing too infrequently in the training set.

1. Molecular_Weight out of range. Value: 446.56. Training min, max, mean, SD: 68.074, 434.63, 171.13, 85.06.
2. Num_AromaticRings out of range. Value: 3. Training min, max, mean, SD: 0, 2, 0.5625, 0.693.
3. OPS PC6 out of range. Value: -2.7619. Training min, max, SD, explained variance: -2.4321, 2.9885, 1.256, 0.0488.
4. Unknown FCFP_2 feature: 19: [*]:[nH]:[*]
5. Unknown FCFP_2 feature: 203707511: [*]C(=[*])[c]1:[nH]:[*]:[*]:[c]:1[*]
6. Unknown FCFP_2 feature: -1986158408: [*]S\C(=N\[*])\N([*])[*]
7. Unknown FCFP_2 feature: 580453787: [*]C(=N[c](:[*]):[*])[*]
8. Unknown FCFP_2 feature: 2005402822: [*][c]1:[*]:[*]:[c](:[*]):[nH]:1
9. Unknown FCFP_2 feature: 307448885: [*]:[cH]:[c]1:[nH]:[*]:[*]:[c]:1:[*]

Feature Contribution

Top features for positive contribution			
Fingerprint	Bit/Smiles	Feature Structure	Score

FCFP_2	332760439	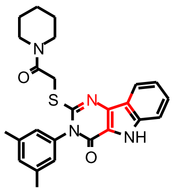 [*][c]1:[*]:[*]:[c](:[*]):[c]:1N=[*]	0.672
FCFP_2	1	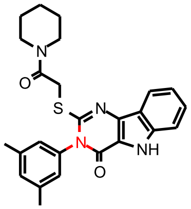 [*]N([*])[*]	0.511
FCFP_2	-1272798659	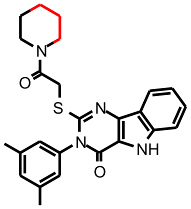 [*]C1[*][*]CC1	0.0703
**Top Features for negative contribution**			
**Fingerprint**	**Bit/Smiles**	**Feature Structure**	**Score**
FCFP_2	203677720	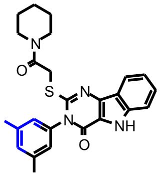 [*]:[cH]:[c](C):[cH]:[*]	-0.406
FCFP_2	565998553	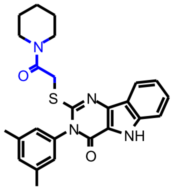 [*]CC(=O)N([*])[*]	-0.348

FCFP_2	1872154524	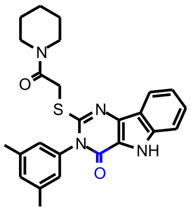<br>[*]C(=O)[*]	-0.307

#UNDEFINED

TOPKAT_Rat_Maximum_Tolerated_Dose_Gavage

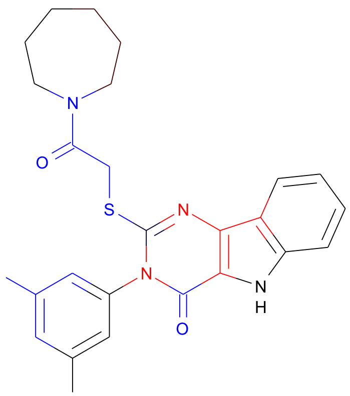

$C_{26}H_{28}N_4O_2S$
Molecular Weight: 460.59111
ALogP: 6.009
Rotatable Bonds: 4
Acceptors: 4
Donors: 1

Model Prediction

Prediction: 0.00975
Unit: g/kg_body_weight
Mahalanobis Distance: 13.3
Mahalanobis Distance p-value: 1.44e-010

Mahalanobis Distance: The Mahalanobis distance (MD) is a generalization of the Euclidean distance that accounts for correlations among the X properties. It is calculated as the distance to the center of the training data. The larger the MD, the less trustworthy the prediction.
Mahalanobis Distance p-value: The p-value gives the fraction of training data with an MD greater than or equal to the one for the given sample, assuming normally distributed data. The smaller the p-value, the less trustworthy the prediciton. For highly non-normal X properties (e.g., fingerprints), the MD p-value is wildly inaccurate.

Structural Similar Compounds

Name	PHENYLBUTAZONE	OCHRATOXIN	PROMETHAZINE.HCL
Structure	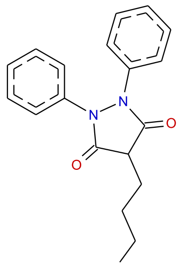	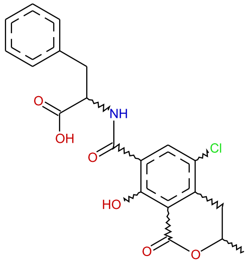	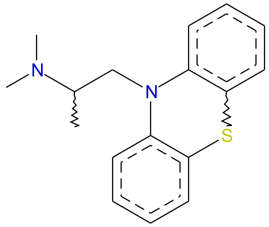
Actual Endpoint (-log C)	3.48909	6.28396	3.93152
Predicted Endpoint (-log C)	3.17333	5.12358	4.72433
Distance	1.001	1.072	1.094
Reference	NCI/NTP TR-367	NCI/NTP TR-358	NCI/NTP TR-425

Model Applicability

Unknown features are fingerprint features in the query molecule, but not found or appearing too infrequently in the training set.

1. Molecular_Weight out of range. Value: 460.59. Training min, max, mean, SD: 68.074, 434.63, 171.13, 85.06.
2. Num_AromaticRings out of range. Value: 3. Training min, max, mean, SD: 0, 2, 0.5625, 0.693.
3. OPS PC6 out of range. Value: -2.8236. Training min, max, SD, explained variance: -2.4321, 2.9885, 1.256, 0.0488.
4. Unknown FCFP_2 feature: 19: [*]:[nH]:[*]
5. Unknown FCFP_2 feature: 203707511: [*]C(=[*])[c]1:[nH]:[*]:[*]:[c]:1[*]
6. Unknown FCFP_2 feature: -1986158408: [*]S\C(=N\[*])\N([*])[*]
7. Unknown FCFP_2 feature: 580453787: [*]C(=N[c](:[*]):[*])[*]
8. Unknown FCFP_2 feature: 2005402822: [*][c]1:[*]:[*]:[c](:[*]):[nH]:1
9. Unknown FCFP_2 feature: 307448885: [*]:[cH]:[c]1:[nH]:[*]:[*]:[c]:1:[*]

Feature Contribution

Top features for positive contribution

Fingerprint	Bit/Smiles	Feature Structure	Score

FCFP_2	332760439	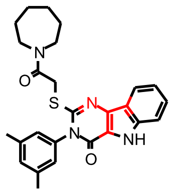 [*][c]1:[*]:[*]:[c](:[*]):[c]:1N=[*]	0.672
FCFP_2	1	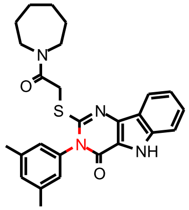 [*]N([*])[*]	0.511
FCFP_2	-1272798659	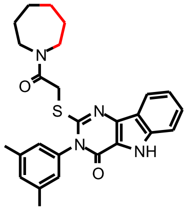 [*]C1[*][*]CC1	0.0703
**Top Features for negative contribution**			
**Fingerprint**	**Bit/Smiles**	**Feature Structure**	**Score**
FCFP_2	203677720	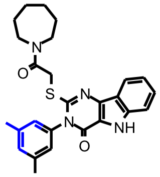 [*]:[cH]:[c](C):[cH]:[*]	-0.406
FCFP_2	565998553	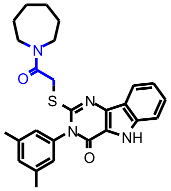 [*]CC(=O)N([*])[*]	-0.348

FCFP_2	1872154524	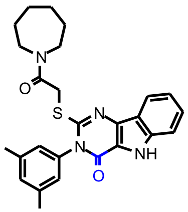 [*]C(=O)[*]	-0.307

#UNDEFINED

TOPKAT_Rat_Maximum_Tolerated_Dose_Gavage

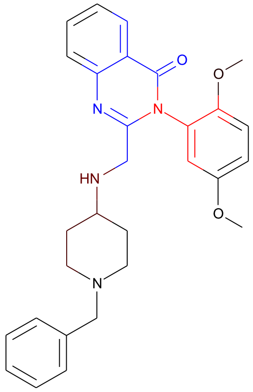

$C_{29}H_{32}N_4O_3$

Molecular Weight: 484.58938

ALogP: 3.743

Rotatable Bonds: 8

Acceptors: 6

Donors: 1

Model Prediction

Prediction: 0.00144

Unit: g/kg_body_weight

Mahalanobis Distance: 15

Mahalanobis Distance p-value: 5.88e-013

Mahalanobis Distance: The Mahalanobis distance (MD) is a generalization of the Euclidean distance that accounts for correlations among the X properties. It is calculated as the distance to the center of the training data. The larger the MD, the less trustworthy the prediction.

Mahalanobis Distance p-value: The p-value gives the fraction of training data with an MD greater than or equal to the one for the given sample, assuming normally distributed data. The smaller the p-value, the less trustworthy the prediciton. For highly non-normal X properties (e.g., fingerprints), the MD p-value is wildly inaccurate.

Structural Similar Compounds

Name	PHENYLBUTAZONE	PROBENECID	OCHRATOXIN
Structure	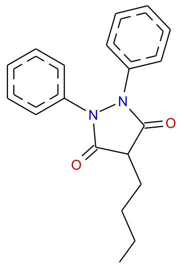	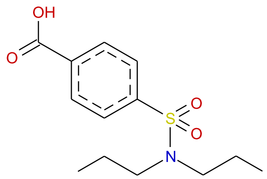	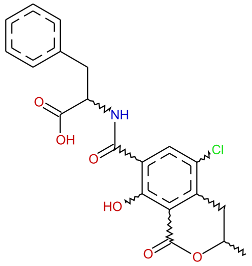
Actual Endpoint (-log C)	3.48909	2.85333	6.28396
Predicted Endpoint (-log C)	3.17333	2.4258	5.12358
Distance	1.042	1.070	1.085
Reference	NCI/NTP TR-367	NCI/NTP TR-395	NCI/NTP TR-358

Model Applicability

Unknown features are fingerprint features in the query molecule, but not found or appearing too infrequently in the training set.

1. Molecular_Weight out of range. Value: 484.59. Training min, max, mean, SD: 68.074, 434.63, 171.13, 85.06.
2. Num_AromaticRings out of range. Value: 3. Training min, max, mean, SD: 0, 2, 0.5625, 0.693.
3. OPS PC6 out of range. Value: -3.4054. Training min, max, SD, explained variance: -2.4321, 2.9885, 1.256, 0.0488.
4. Unknown FCFP_2 feature: 580453787: [*]C(=N[c](:[*]):[*])[*]
5. Unknown FCFP_2 feature: 906798516: [*]N([*])C[c](:[*]):[*]

Feature Contribution

Top features for positive contribution

Fingerprint	Bit/Smiles	Feature Structure	Score
FCFP_2	332760439	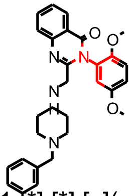 [*][c]1:[*]:[*]:[c](:[*]):[c]:1N=[*]	0.672

FCFP_2	1	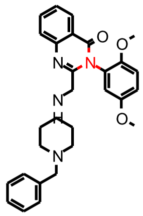 [*]N([*])[*]	0.511
FCFP_2	3	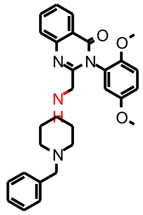 [*]N[*]	0.104
**Top Features for negative contribution**			
**Fingerprint**	**Bit/Smiles**	**Feature Structure**	**Score**
FCFP_2	203677720	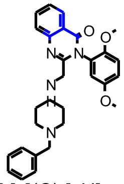 [*]:[cH]:[c](C):[cH]:[*]	-0.406
FCFP_2	565998553	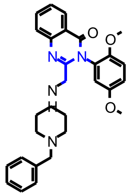 [*]CC(=O)N([*])[*]	-0.348
FCFP_2	1872154524	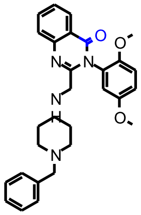 [*]C(=O)[*]	-0.307

#UNDEFINED

TOPKAT_Rat_Maximum_Tolerated_Dose_Gavage

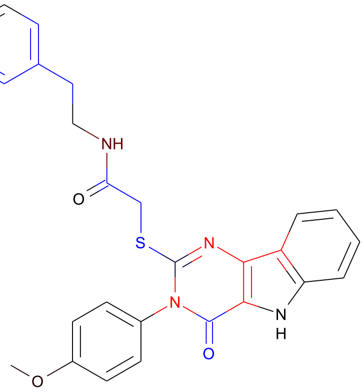

$C_{27}H_{24}N_4O_3S$
Molecular Weight: 484.56946
ALogP: 5.346
Rotatable Bonds: 8
Acceptors: 5
Donors: 2

Model Prediction

Prediction: 0.00705
Unit: g/kg_body_weight
Mahalanobis Distance: 14.9
Mahalanobis Distance p-value: 7.17e-013

Mahalanobis Distance: The Mahalanobis distance (MD) is a generalization of the Euclidean distance that accounts for correlations among the X properties. It is calculated as the distance to the center of the training data. The larger the MD, the less trustworthy the prediction.
Mahalanobis Distance p-value: The p-value gives the fraction of training data with an MD greater than or equal to the one for the given sample, assuming normally distributed data. The smaller the p-value, the less trustworthy the prediciton. For highly non-normal X properties (e.g., fingerprints), the MD p-value is wildly inaccurate.

Structural Similar Compounds

Name	OCHRATOXIN	SULFISOOXAZOLE	PENICILLIN VK
Structure	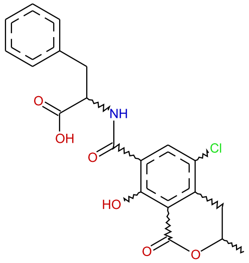	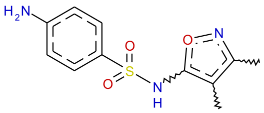	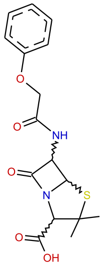
Actual Endpoint (-log C)	6.28396	2.82494	2.54455
Predicted Endpoint (-log C)	5.12358	3.0705	3.9702
Distance	1.026	1.307	1.309
Reference	NCI/NTP TR-358	NCI/NTP TR-138	NCI/NTP TR-336

Model Applicability

Unknown features are fingerprint features in the query molecule, but not found or appearing too infrequently in the training set.

1. Molecular_Weight out of range. Value: 484.57. Training min, max, mean, SD: 68.074, 434.63, 171.13, 85.06.
2. Num_AromaticRings out of range. Value: 4. Training min, max, mean, SD: 0, 2, 0.5625, 0.693.
3. OPS PC6 out of range. Value: -3.1462. Training min, max, SD, explained variance: -2.4321, 2.9885, 1.256, 0.0488.
4. Unknown FCFP_2 feature: 19: [*]:[nH]:[*]
5. Unknown FCFP_2 feature: 203707511: [*]C(=[*])[c]1:[nH]:[*]:[*]:[c]:1[*]
6. Unknown FCFP_2 feature: -1986158408: [*]S\C(=N\[*])\N([*])[*]
7. Unknown FCFP_2 feature: 580453787: [*]C(=N[c](:[*]):[*])[*]
8. Unknown FCFP_2 feature: 2005402822: [*][c]1:[*]:[*]:[c](:[*]):[nH]:1
9. Unknown FCFP_2 feature: 307448885: [*]:[cH]:[c]1:[nH]:[*]:[*]:[c]:1:[*]

Feature Contribution

Top features for positive contribution			
Fingerprint	Bit/Smiles	Feature Structure	Score

FCFP_2	332760439	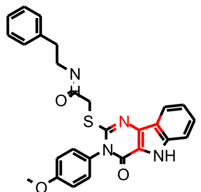 [*][c]1:[*]:[*]:[*]:[c](:[*]):[c]:1N=[*]	0.672
FCFP_2	1	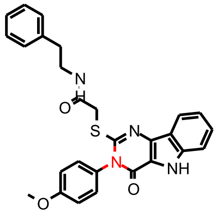 [*]N([*])[*]	0.511
FCFP_2	3	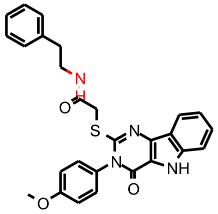 [*]N[*]	0.104
**Top Features for negative contribution**			
**Fingerprint**	**Bit/Smiles**	**Feature Structure**	**Score**
FCFP_2	203677720	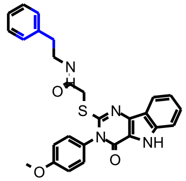 [*]:[cH]:[c](C):[cH]:[*]	-0.406
FCFP_2	1872154524	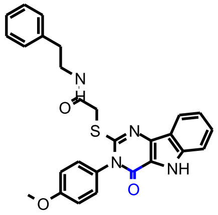 [*]C(=O)[*]	-0.307

FCFP_2	0	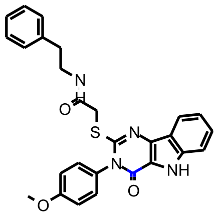 [*]C(=[*])[*]	-0.29

Molecule

TOPKAT_Rat_Oral_LD50

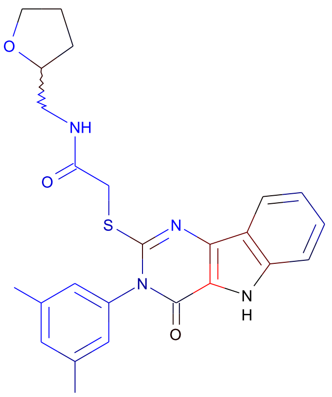

$C_{25}H_{26}N_4O_3S$

Molecular Weight: 462.56393

ALogP: 4.789

Rotatable Bonds: 6

Acceptors: 5

Donors: 2

Model Prediction

Prediction: 31.1

Unit: g/kg_body_weight

Mahalanobis Distance: 29.4

Mahalanobis Distance p-value: 9.65e-060

Mahalanobis Distance: The Mahalanobis distance (MD) is a generalization of the Euclidean distance that accounts for correlations among the X properties. It is calculated as the distance to the center of the training data. The larger the MD, the less trustworthy the prediction.
Mahalanobis Distance p-value: The p-value gives the fraction of training data with an MD greater than or equal to the one for the given sample, assuming normally distributed data. The smaller the p-value, the less trustworthy the prediciton. For highly non-normal X properties (e.g., fingerprints), the MD p-value is wildly inaccurate.

Structural Similar Compounds

Name	ACEMETACIN	bis-OXATIN ACETATE	TALNIFLUMATE
Structure	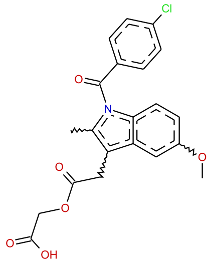	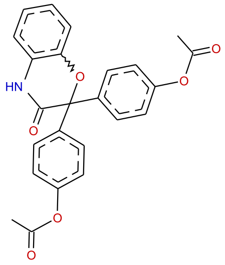	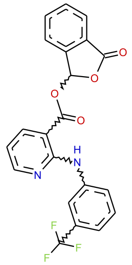
Actual Endpoint (-log C)	4.235	1.717	1.538
Predicted Endpoint (-log C)	3.39415	2.40947	2.82541
Distance	0.643	0.673	0.688
Reference	ARZNAD 30;1398;80	NIIRDN 6;609;82	FRPSAX 36;372;81

Model Applicability

Unknown features are fingerprint features in the query molecule, but not found or appearing too infrequently in the training set.

1. All properties and OPS components are within expected ranges.
2. Unknown ECFP_2 feature: -962137479: [*][c]1:[*]:[*]:[c](:[*]):[c]:1N=[*]
3. Unknown FCFP_6 feature: 16: [*][c](:[*]):[*]
4. Unknown FCFP_6 feature: 19: [*]:[nH]:[*]
5. Unknown FCFP_6 feature: 203707511: [*]C(=[*])[c]1:[nH]:[*]:[*]:[c]:1[*]
6. Unknown FCFP_6 feature: 580453787: [*]C(=N[c](:[*]):[*])[*]
7. Unknown FCFP_6 feature: 2005402822: [*][c]1:[*]:[*]:[c](:[*]):[nH]:1
8. Unknown FCFP_6 feature: 307448885: [*]:[cH]:[c]1:[nH]:[*]:[*]:[c]:1:[*]
9. Unknown FCFP_6 feature: 1618154665: [*][c](:[*]):[cH]:[c]([*]):[*]

Feature Contribution

Top features for positive contribution			
Fingerprint	Bit/Smiles	Feature Structure	Score

ECFP_6	642810091	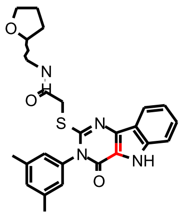 [*][c](:[*]):[*]	0.281
ECFP_6	-1897341097	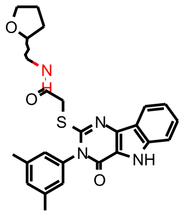 [*]N[*]	0.216
FCFP_6	-1549163031	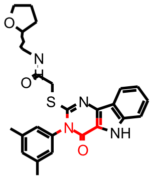 [*]N([*])C(=O)[c](:[*]):[*]	0.171
**Top Features for negative contribution**			
**Fingerprint**	**Bit/Smiles**	**Feature Structure**	**Score**
ECFP_6	2106656448	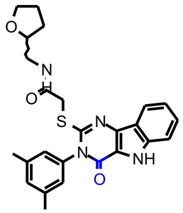 [*]C(=O)[*]	-0.352
ECFP_6	497523368	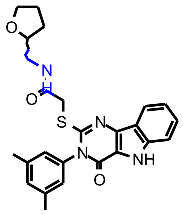 [*]CNC(=[*])[*]	-0.301

ECFP_6	683445015	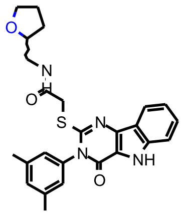 [*]O[*]	-0.266

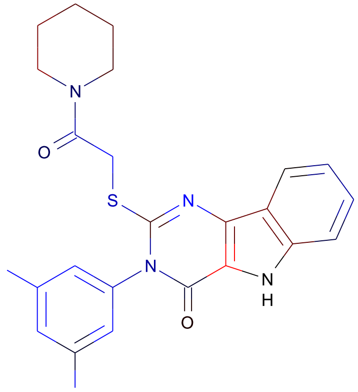

$C_{25}H_{26}N_4O_2S$

Molecular Weight: 446.56453

ALogP: 5.553

Rotatable Bonds: 4

Acceptors: 4

Donors: 1

Model Prediction

Prediction: 3.92

Unit: g/kg_body_weight

Mahalanobis Distance: 27.6

Mahalanobis Distance p-value: 3.38e-047

Mahalanobis Distance: The Mahalanobis distance (MD) is a generalization of the Euclidean distance that accounts for correlations among the X properties. It is calculated as the distance to the center of the training data. The larger the MD, the less trustworthy the prediction.
Mahalanobis Distance p-value: The p-value gives the fraction of training data with an MD greater than or equal to the one for the given sample, assuming normally distributed data. The smaller the p-value, the less trustworthy the prediciton. For highly non-normal X properties (e.g., fingerprints), the MD p-value is wildly inaccurate.

Structural Similar Compounds

Name	2-BENZIMIDAZOLINONE; 1-[1-(3-CYANO-3;3-DIPHENYLPROPYL)-4-PIPERIDYL]-3-PROPIONYL-	TALNIFLUMATE	1-BENZENESULFONYL-5;5-DIPHENYLHYDANTOIN
Structure	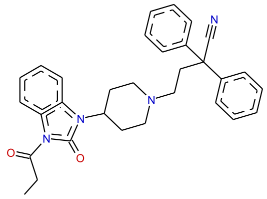	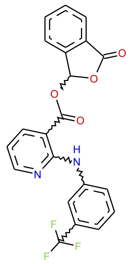	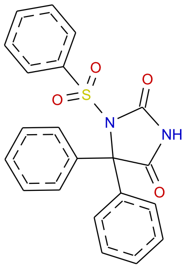
Actual Endpoint (-log C)	3.543	1.538	2.363
Predicted Endpoint (-log C)	3.00532	2.82541	2.34793
Distance	0.630	0.637	0.639
Reference	ARZNAD 21;862;71	FRPSAX 36;372;81	ARZNAD 20;1579;70

Model Applicability

Unknown features are fingerprint features in the query molecule, but not found or appearing too infrequently in the training set.

1. All properties and OPS components are within expected ranges.
2. Unknown ECFP_2 feature: -962137479: [*][c]1:[*]:[*]:[c](:[*]):[c]:1N=[*]
3. Unknown FCFP_6 feature: 16: [*][c](:[*]):[*]
4. Unknown FCFP_6 feature: 19: [*]:[nH]:[*]
5. Unknown FCFP_6 feature: 203707511: [*]C(=[*])[c]1:[nH]:[*]:[*]:[c]:1[*]
6. Unknown FCFP_6 feature: 580453787: [*]C(=N[c](:[*]):[*])[*]
7. Unknown FCFP_6 feature: 2005402822: [*][c]1:[*]:[*]:[c](:[*]):[nH]:1
8. Unknown FCFP_6 feature: 307448885: [*]:[cH]:[c]1:[nH]:[*]:[*]:[c]:1:[*]
9. Unknown FCFP_6 feature: 1618154665: [*][c](:[*]):[cH]:[c]([*]):[*]

Feature Contribution

Top features for positive contribution			
**Fingerprint**	**Bit/Smiles**	**Feature Structure**	**Score**

ECFP_6	642810091	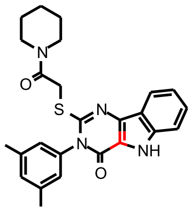 [*][c](:[*]):[*]	0.281
FCFP_6	-1549163031	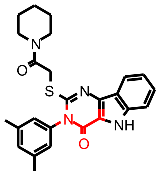 [*]N([*])C(=O)[c](:[*]):[*]	0.171
FCFP_6	675769755	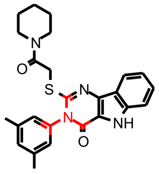 [*]C(=[*])N(C(=[*])[*])[c](:[*]):[*]	0.155

Top Features for negative contribution

Fingerprint	Bit/Smiles	Feature Structure	Score
ECFP_6	2106656448	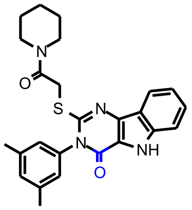 [*]C(=O)[*]	-0.352
FCFP_6	1676877079	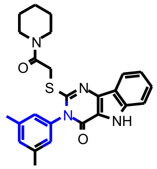 [*]N([*])[c]1:[cH]:[*]:[cH]:[c](C):[cH]:1	-0.254

ECFP_6	655739385	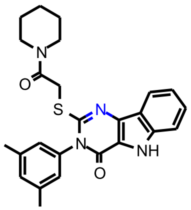<br>[*]N=[*]	-0.239

#UNDEFINED

TOPKAT_Rat_Oral_LD50

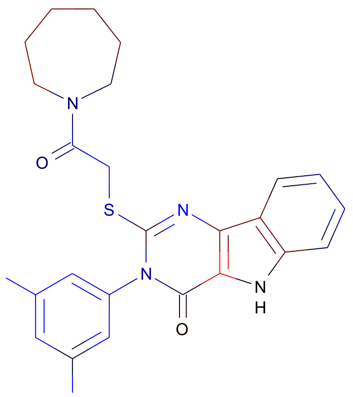

$C_{26}H_{28}N_4O_2S$

Molecular Weight: 460.59111

ALogP: 6.009

Rotatable Bonds: 4

Acceptors: 4

Donors: 1

Structural Similar Compounds

Name	2-BENZIMIDAZOLINONE; 1-[1-(3-CYANO-3;3-DIPHENYLPROPYL)-4-PIPERIDYL]-3-PROPIONYL-	TALNIFLUMATE	INDOMETHAZINE
Structure	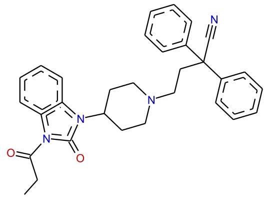	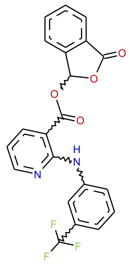	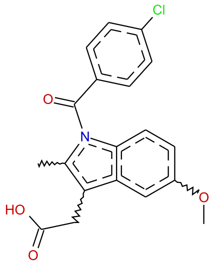
Actual Endpoint (-log C)	3.543	1.538	5.17
Predicted Endpoint (-log C)	3.00532	2.82541	3.33605
Distance	0.639	0.659	0.685
Reference	ARZNAD 21;862;71	FRPSAX 36;372;81	ARZNAD 25;1526;75

Model Prediction

Prediction: 3.86

Unit: g/kg_body_weight

Mahalanobis Distance: 28.1

Mahalanobis Distance p-value: 1.51e-050

Mahalanobis Distance: The Mahalanobis distance (MD) is a generalization of the Euclidean distance that accounts for correlations among the X properties. It is calculated as the distance to the center of the training data. The larger the MD, the less trustworthy the prediction.

Mahalanobis Distance p-value: The p-value gives the fraction of training data with an MD greater than or equal to the one for the given sample, assuming normally distributed data. The smaller the p-value, the less trustworthy the prediciton. For highly non-normal X properties (e.g., fingerprints), the MD p-value is wildly inaccurate.

Model Applicability

Unknown features are fingerprint features in the query molecule, but not found or appearing too infrequently in the training set.

1. All properties and OPS components are within expected ranges.
2. Unknown ECFP_2 feature: -962137479: [*][c]1:[*]:[*]:[c](:[*]):[c]:1N=[*]
3. Unknown FCFP_6 feature: 16: [*][c](:[*]):[*]
4. Unknown FCFP_6 feature: 19: [*]:[nH]:[*]
5. Unknown FCFP_6 feature: 203707511: [*]C(=[*])[c]1:[nH]:[*]:[*]:[c]:1[*]
6. Unknown FCFP_6 feature: 580453787: [*]C(=N[c](:[*]):[*])[*]
7. Unknown FCFP_6 feature: 2005402822: [*][c]1:[*]:[*]:[c](:[*]):[nH]:1
8. Unknown FCFP_6 feature: 307448885: [*]:[cH]:[c]1:[nH]:[*]:[*]:[c]:1:[*]
9. Unknown FCFP_6 feature: 1618154665: [*][c](:[*]):[cH]:[c]([*]):[*]

Feature Contribution

Top features for positive contribution			
Fingerprint	Bit/Smiles	Feature Structure	Score

ECFP_6	642810091	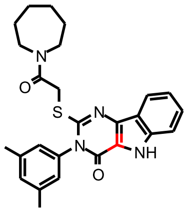 [*][c](:[*]):[*]	0.281
FCFP_6	-1549163031	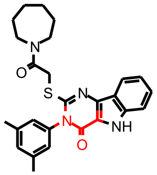 [*]N([*])C(=O)[c](:[*]):[*]	0.171
FCFP_6	675769755	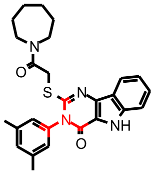 [*]C(=[*])N(C(=[*])[*])[c](:[*]):[*]	0.155
**Top Features for negative contribution**			
**Fingerprint**	**Bit/Smiles**	**Feature Structure**	**Score**
ECFP_6	2106656448	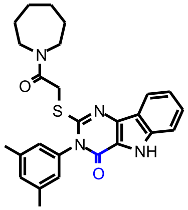 [*]C(=O)[*]	-0.352
FCFP_6	1676877079	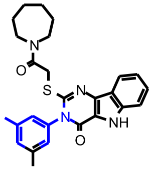 [*]N([*])[c]1:[cH]:[*]:[cH]:[c](C):[cH]:1	-0.254

ECFP_6	655739385	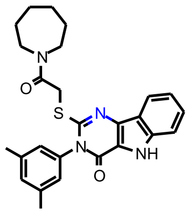 [*]N=[*]	-0.239

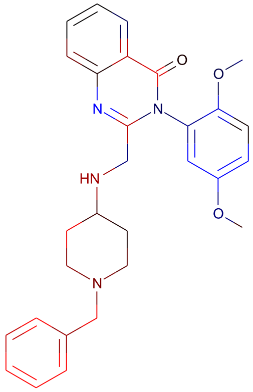

$C_{29}H_{32}N_4O_3$
Molecular Weight: 484.58938
ALogP: 3.743
Rotatable Bonds: 8
Acceptors: 6
Donors: 1

Structural Similar Compounds

Name	KETOCONAZOLE	DIXYRAZINE .HCl (HCl STRIPPED)	2-BENZIMIDAZOLINONE; 1-[1-(3-CYANO-3;3-DIPHENYLPROPYL)-4-PIPERIDYL]-3-PROPIONYL-
Structure	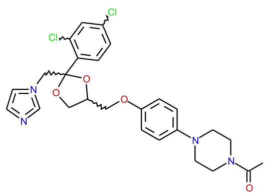	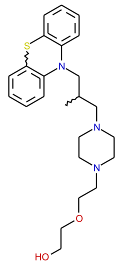	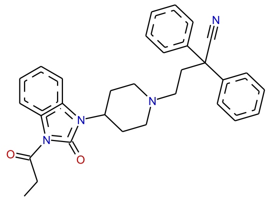
Actual Endpoint (-log C)	3.505	3.029	3.543
Predicted Endpoint (-log C)	2.65464	2.47585	3.00532
Distance	0.565	0.613	0.629
Reference	MDACAP 17;373;81	ANPBAZ 61;669;61	ARZNAD 21;862;71

Model Prediction

Prediction: 0.873
Unit: g/kg_body_weight
Mahalanobis Distance: 23.1
Mahalanobis Distance p-value: 2.53e-021

Mahalanobis Distance: The Mahalanobis distance (MD) is a generalization of the Euclidean distance that accounts for correlations among the X properties. It is calculated as the distance to the center of the training data. The larger the MD, the less trustworthy the prediction.
Mahalanobis Distance p-value: The p-value gives the fraction of training data with an MD greater than or equal to the one for the given sample, assuming normally distributed data. The smaller the p-value, the less trustworthy the prediciton. For highly non-normal X properties (e.g., fingerprints), the MD p-value is wildly inaccurate.

Model Applicability

Unknown features are fingerprint features in the query molecule, but not found or appearing too infrequently in the training set.

1. All properties and OPS components are within expected ranges.
2. Unknown FCFP_6 feature: 16: [*][c](:[*]):[*]
3. Unknown FCFP_6 feature: 580453787: [*]C(=N[c](:[*]):[*])[*]
4. Unknown FCFP_6 feature: 1618154665: [*][c](:[*]):[cH]:[c]([*]):[*]
5. Unknown FCFP_6 feature: 906798516: [*]N([*])C[c](:[*]):[*]

Feature Contribution

Top features for positive contribution

Fingerprint	Bit/Smiles	Feature Structure	Score

ECFP_6	642810091	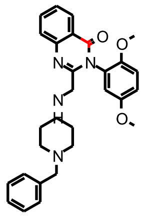 [*][c](:[*]):[*]	0.281
ECFP_6	-1897341097	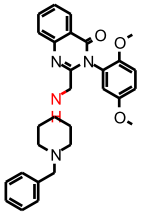 [*]N[*]	0.216
ECFP_6	1571214559	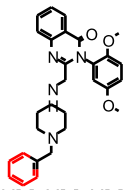 [*]1:[cH]:[cH]:[cH]:[cH]:[cH]:1	0.19
**Top Features for negative contribution**			
**Fingerprint**	**Bit/Smiles**	**Feature Structure**	**Score**
ECFP_6	2106656448	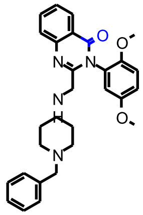 [*]C(=O)[*]	-0.352
ECFP_6	-176455838	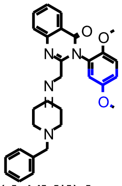 [*]O[c](:[cH]:[*]):[cH]:[*]	-0.257

ECFP_6	2077607946	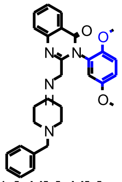 [*]O[c]1:[cH]:[cH]:[c]([*]):[*]:[c]:1[*]	-0.252

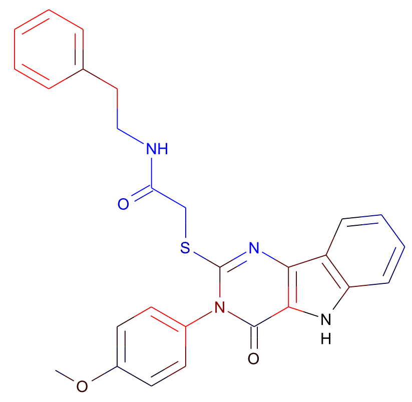

$C_{27}H_{24}N_4O_3S$
Molecular Weight: 484.56946
ALogP: 5.346
Rotatable Bonds: 8
Acceptors: 5
Donors: 2

Structural Similar Compounds

Name	BENZENESULFONIC ACID; 2;2'-(4;4'-BIPHENYLYLENEDIVINYLENE)DI-; DISODIUM SALT (Na STRIPPED)	ACEMETACIN	TALNIFLUMATE
Structure	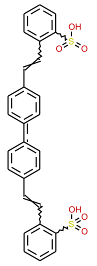	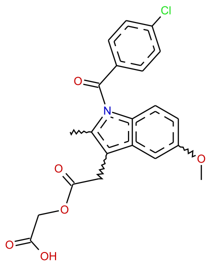	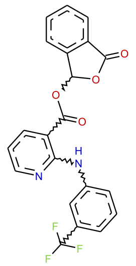
Actual Endpoint (-log C)	1.968	4.235	1.538
Predicted Endpoint (-log C)	1.72109	3.39415	2.82541
Distance	0.712	0.733	0.782
Reference	MVCRB3 2;193;73	ARZNAD 30;1398;80	FRPSAX 36;372;81

Model Prediction

Prediction: 2.46
Unit: g/kg_body_weight
Mahalanobis Distance: 28.2
Mahalanobis Distance p-value: 5.14e-051

Mahalanobis Distance: The Mahalanobis distance (MD) is a generalization of the Euclidean distance that accounts for correlations among the X properties. It is calculated as the distance to the center of the training data. The larger the MD, the less trustworthy the prediction.
Mahalanobis Distance p-value: The p-value gives the fraction of training data with an MD greater than or equal to the one for the given sample, assuming normally distributed data. The smaller the p-value, the less trustworthy the prediciton. For highly non-normal X properties (e.g., fingerprints), the MD p-value is wildly inaccurate.

Model Applicability

Unknown features are fingerprint features in the query molecule, but not found or appearing too infrequently in the training set.

1. All properties and OPS components are within expected ranges.
2. Unknown ECFP_2 feature: -962137479: [*][c]1:[*]:[*]:[c](:[*]):[c]:1N=[*]
3. Unknown FCFP_6 feature: 16: [*][c](:[*]):[*]
4. Unknown FCFP_6 feature: 19: [*]:[nH]:[*]
5. Unknown FCFP_6 feature: 203707511: [*]C(=[*])[c]1:[nH]:[*]:[*]:[c]:1[*]
6. Unknown FCFP_6 feature: 580453787: [*]C(=N[c](:[*]):[*])[*]
7. Unknown FCFP_6 feature: 2005402822: [*][c]1:[*]:[*]:[c](:[*]):[nH]:1
8. Unknown FCFP_6 feature: 307448885: [*]:[cH]:[c]1:[nH]:[*]:[*]:[c]:1:[*]
9. Unknown FCFP_6 feature: 1618154665: [*][c](:[*]):[cH]:[c]([*]):[*]

Feature Contribution

Top features for positive contribution			
Fingerprint	Bit/Smiles	Feature Structure	Score

ECFP_6	642810091	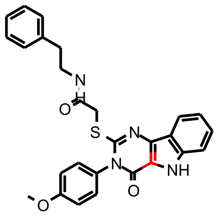 [*][c](:[*]):[*]	0.281
ECFP_6	-1897341097	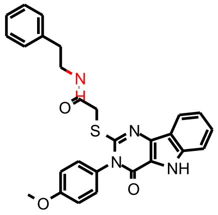 [*]N[*]	0.216
ECFP_6	1571214559	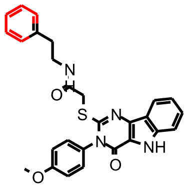 [*]1:[cH]:[cH]:[cH]:[cH]:[cH]:1	0.19
**Top Features for negative contribution**			
**Fingerprint**	**Bit/Smiles**	**Feature Structure**	**Score**
ECFP_6	2106656448	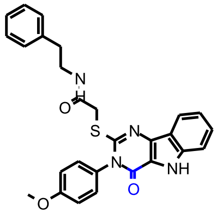 [*]C(=O)[*]	-0.352
ECFP_6	497523368	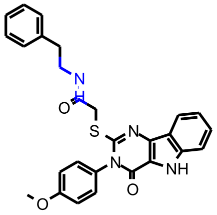 [*]CNC(=[*])[*]	-0.301

ECFP_6	-176455838	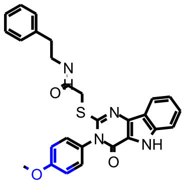 [*]O[c](:[cH]:[*]):[cH]:[*]	-0.257
